# Chemical diversification of polyprenyl quinones for mechanistic studies on menaquinone-binding peptide antibiotics†

**DOI:** 10.1039/d5sc03363b

**Published:** 2025-07-03

**Authors:** Eilidh J. Matheson, Roy A. M. van Beekveld, Paolo Innocenti, Nathaniel I. Martin, Markus Weingarth, Stephen A. Cochrane

**Affiliations:** a School of Chemistry and Chemical Engineering, Queen's University Belfast David Keir Building, Stranmillis Road Belfast BT9 5AG UK s.cochrane@qub.ac.uk; b NMR Spectroscopy, Bijvoet Center for Biomolecular Research, Department of Chemistry, Utrecht University Padualaan 8 3584 CH Utrecht The Netherlands; c Biological Chemistry Group, Institute of Biology Leiden, Leiden University Sylviusweg 72 2333 BE Leiden The Netherlands

## Abstract

Polyprenyl quinones, such as ubiquinone and menaquinone, are essential membrane-embedded redox cofactors that are involved in electron transport and found across all domains of life. However, their highly hydrophobic structure, which includes a quinone head-group and long polyprenyl tail, has limited their chemical derivatization for biological studies. Here, we report a versatile synthetic approach for the chemical diversification of natural polyprenyl quinones, enabling the introduction of various reporter groups including fluorophores, quenchers, NMR-active nuclei, and photoaffinity and bioaffinity tags. These functionalized analogues retain their membrane-associating properties and enable new applications in antibiotic discovery. We show that fluorescently labelled menaquinone analogues retain their strong binding affinity to the menaquinone-binding peptide antibiotics lysocin E and lysomeb (MBA2). Incorporation of BODIPY–quinones into vesicles allowed visualization of the peptide–quinone interaction, revealing their effects on membrane integrity and quinone aggregation. This study expands the chemical toolbox for polyprenyl quinones, enabling targeted functionalization of these essential biomolecules and facilitating further exploration of their roles in biological systems.

## Abstract 2

Chemically labelled polyprenyl quinones (PPQs) have been synthesized by direct modification of native PPQs. These chemical probes offer new tools for studying redox cofactors involved in bacterial respiration and antimicrobial targeting.

## Graphical abstract

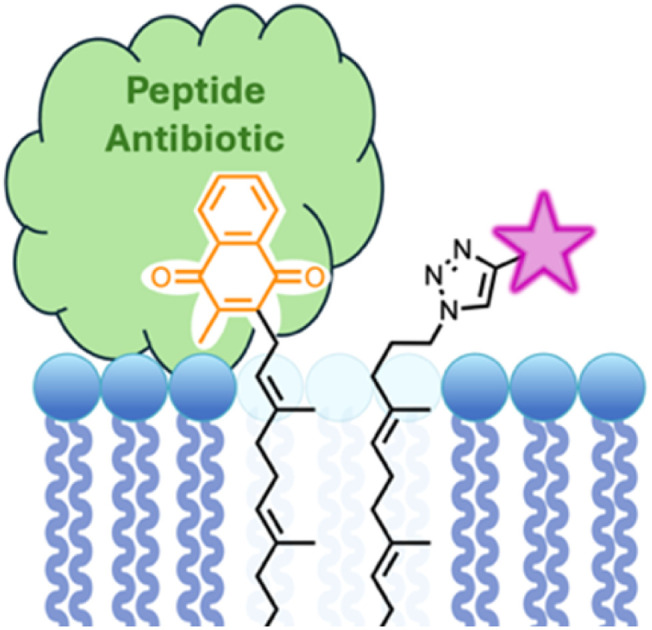

## Introduction

**Fig. 1:**
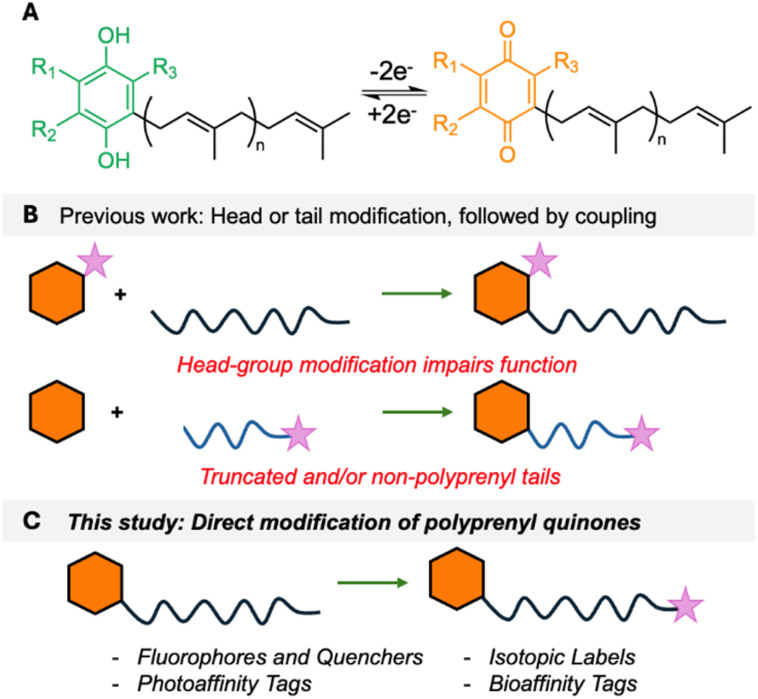
(A) Redox process of PPQs. (B) Previous approaches to attaching reporter groups. Hexagon = head group, wiggly line = polyprenyl tail and purple star = reporter group. (C) This study.

Polyprenyl quinones (PPQs), such as ubiquinone/coenzyme Q (UQ/CoQ) and menaquinone (MK), are essential membrane-bound redox cofactors that function as electron carriers in cellular respiration and photosynthesis.^1,2^ Their role in energy production is critical across all domains of life, with CoQ serving as a key component of the eukaryotic respiratory chain, while MK is required for anaerobic respiration in bacteria (but not found in humans).^3,4^ Structurally, PPQs consist of a redox-active quinone head group linked to a hydrophobic polyprenyl tail, which anchors them to the cell membrane and allows efficient electron transfer between protein complexes (Fig. 1A). Enzymes that synthesize PPQs or use them as cofactors, and even PPQs themselves, are emerging as attractive therapeutic targets, particularly in antibiotic development.^5^ Atovaquone is an antimalarial drug that inhibits the cytochrome bc1 complex of *Plasmodium falciparum* by mimicking CoQ.^6^ Lotilibcin (WAP-8294A2), a cyclic lipopeptide, was recently in phase II clinical trials for treatment of methicillin-resistant *Staphylococcus aureus*.^7^ Additionally, bicyclic MenA inhibitors are being developed by Otsuka Pharmaceuticals to target *Mycobacterium tuberculosis*.^8^ PPQs also play roles in oxidative stress regulation, making them potential targets for anti-cancer and neuroprotective therapies.^9^ Despite their biological significance, installation of reporter groups such as fluorophores, isotopic labels, or affinity tags onto PPQs has remained challenging. Such chemical probes would open new possibilities in bioenergetics, drug development, and mechanism-of-action studies. The challenge in the chemical labelling of PPQs is that there are few exploitable attachment points. Their quinone head groups are highly specific to their function. For example, the redox potentials of PPQs are fine-tuned, with MK and rhodoquinone (RQ) facilitating anaerobic respiration, whereas CoQ is optimized for aerobic electron transport.^1^ Furthermore, the peptide antibiotics lotilibcin,^7^ lysocin E^10^ and lysomeb (MBA2)^11^ kill bacteria by binding to MK, but do not interact with CoQ. Therefore, small headgroup modifications can severely impair function. The polyprenyl lipid tail is extremely hydrophobic, offering few site-selective modification points. As a consequence, previous chemical labelling approaches for PPQs have been mostly limited to separate head/tail labelling, followed by fragment coupling (Fig. 1B),^12–16^ or direct reduction or isotopic labelling of the polyprenyl tail.^17,18^ In previous work, we developed a concise approach that allowed installation of reporter groups on to the ω-terminus of undecaprenol, an essential bacterial lipid involved in cell wall assembly.^19^ Here, we describe a streamlined approach to the chemical diversification of PPQs, enabling the synthesis of a wide array of labelled analogues (Fig. 1C), and use fluorescently labelled menaquinone to provide new insights into how lysocin E and lysomeb (MBA2) interact with menaquinone in membranes.

## Results and discussion

### Direct PPQ labelling *via* oxidation and reductive amination

**Scheme 1:**
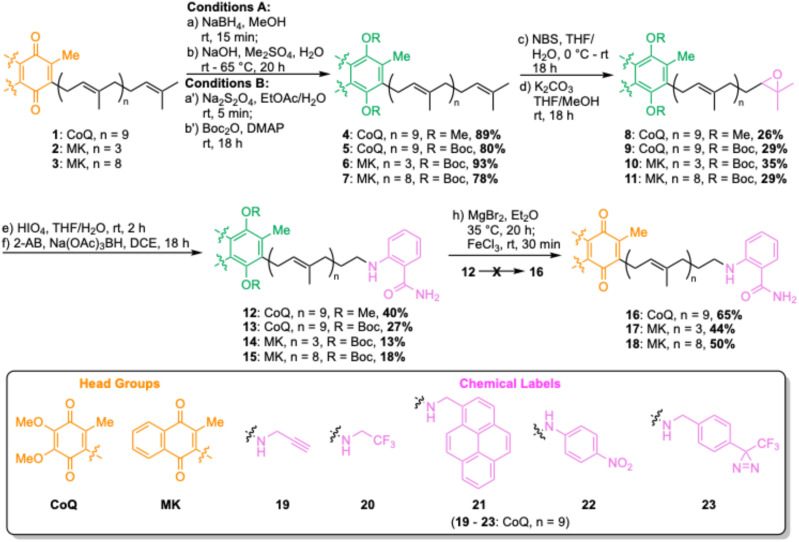
Direct labelling of polyprenyl quinones *via* ω-oxidation and reductive amination. DCE = dichloroethane, DMAP = 4-dimethylaminopyridine, NBS = *N*-bromosuccinimide, THF = tetrahydrofuran.

For direct labelling of PPQs, we envisaged ω-epoxidation and oxidative cleavage to an aldehyde, which could then undergo reductive amination with functionalized amines (Scheme 1). However, this proved more complicated than simply translating our previously reported methodology,^19^ due to the sensitivities of the quinone head group. Attempts to perform direct ω-oxidation (*via* NBS, then K_2_CO_3_/MeOH) on CoQ10 (1), MK4 (2) or MK9 (3) were unsuccessful due to decomposition under these conditions. Therefore, PPQs needed to be reduced to their quinol form and protected as either Me (CoQ) or Boc groups (CoQ and MK). Two-pot reduction/protection reactions proceeded in good yields to provide protected quinols 4–7. Subsequent treatment with NBS in THF/H_2_O, followed by K_2_CO_3_ in THF/MeOH, gave ω-epoxides 8–11 in moderate yields. These yields are in line with other polyprenyl systems we have worked with, and the two-pot reaction yields only desired product and unreacted starting material. Quite remarkable selectivity given that there are ten alkenes in CoQ10, and nine in MK9. Oxidative cleavage with HIO_4_ proceeded smoothly to yield ω-aldehydes, which were directly used in a test reductive amination with fluorophore 2-aminobenzamide (2-AB) yielding polyprenyls 12–15. 2-AB is frequently used along with dinitro-benzene (DNB) in FRET pairings.^20,21^ A problematic side reaction encountered during this step was double reduction amination, resulting in lower yields and we were unable to significantly suppress this reaction. We also encountered problems with the use of Me-ether protecting groups, as standard deprotection conditions^22^ resulted in cleavage of 2-AB from polyprenyl 12 (see Fig. S1†). Gratifyingly, Boc protecting groups could be removed by refluxing with MgBr_2_ in Et_2_O,^23^ followed by addition of FeCl_3_ to oxidize back to the quinone form, yielding 2-AB-PPQs 16–18. The strategy was extended to label CoQ10 with an alkyne click tag (19), ^19^F (20), pyrene (21) and 2-nitrophenyl (22) fluorophores, and a photoaffinity tag (23), highlighting the versatility of this method for ubiquinone modifications.

### Azido-PPQs allow diverse chemical label attachment

**Scheme 2:**
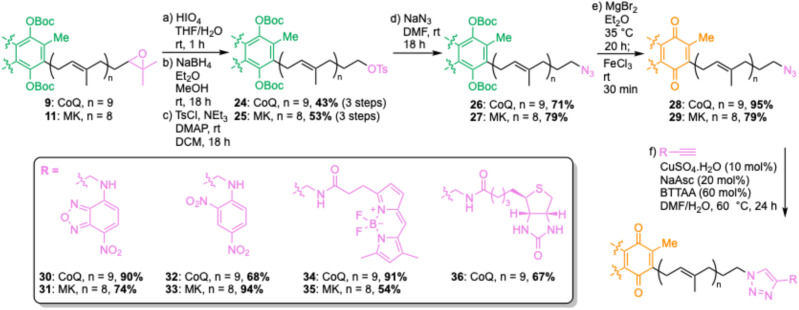
Copper-catalyzed azide–alkyne cycloaddition (CuAAC) approach for chemically labelling polyprenyl quinones. Asc = ascorbate, DCM = dichloromethane, DMAP = 4-dimethylaminopyridine, DMF = dimethylformamide, THF = tetrahydrofuran, Ts = tosyl.

The reductive amination strategy has two limitations – yields of reductive amination reactions were particularly low for menaquinone derivatives, and the Lewis acid conditions used to remove Boc protecting groups would not be compatible with many reporter groups (*e.g.*, acid-labile moieties). To overcome these limitations, we envisaged synthesis of ω-azides, which could be used as functional tags for attachment of virtually any reporter group through Cu-catalyzed azide–alkyne cycloadditions (CuAAC) (Scheme 2).^24,25^ Starting from ω-epoxides 9 and 11, oxidative cleavage was performed by treatment with HIO_4_, followed by NaBH_4_-mediated aldehyde reductions to provide ω-alcohols. These alcohol intermediates were used directly in the next step, providing ω-tosylates 24 and 25 in good yields over three steps. As well as ω-tosylates, we trialled ω-mesylates but found yields of subsequent nucleophilic displacement reactions to be highly variable. In contrast, ω-tosylates can be purified by column chromatography and fully characterized. The azido group was then installed by nucleophilic displacement with NaN_3_, yielding ω-azides 26 and 27, which were subsequently deprotected and oxidized to give azido-PPQs 28 and 29. With azido-CoQ10 (30) and azido-MK9 (31) in hand, we then explored CuAAC conditions for attachment of a quenchable nitrobenzodiazole (NBD) fluorophore (30 and 31), DNB quencher (32 and 33), BODIPY fluorophore (34 and 35) and biotin affinity tag (36). At first some reactions were sluggish and/or low yielding but upon addition of 60 mol% BTTAA, a ligand known to improve the efficiency of CuAAC reactions,^26^ good yields were obtained for all reactions, highlighting the power of this approach to provide a variety of different labelled PPQs. Having established a robust method to chemically label PPQs, we next proceeded to check that these modifications do not impair their function.

### Synthesis of menaquinone-binding antimicrobial peptides

**Scheme 3:**
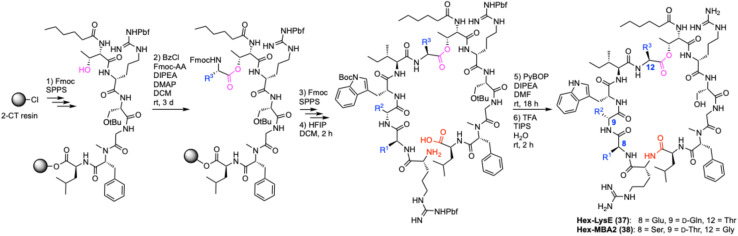
Synthesis of menaquinone-binding peptide antibiotics. AA = amino acid, Bz = benzoyl, CT = chlorotrityl, DCM = dichloromethane, DIPEA = diisopropylethylamine, DMAP = 4-dimethylaminopyridine, HFIP = hexafluoroisopropanol, SPPS = solid-phase peptide synthesis, TFA = trifluoroacetic acid, TIPS = triisopropylsilane.

The first menaquinone-binding peptide antibiotic lysocin E was reported in 2015.^10^ Since then, additional classes have been characterized including the natural product lotilibcin (WAP-8294A2)^7^ and synthetic peptides like lysomeb (MBA2).^11^ Little is known about their mechanism of action beyond their binding to menaquinone and disruption of cell membranes.^10^ We postulated that some of the labelled PPQs described above could be used as mechanistic probes. To investigate this, we synthesized novel analogues Hex-LysE (37) and Hex-MBA2 (38) (Scheme 3). We chose analogues with achiral hexanoyl N-terminal lipids as this removed the need for a multistep synthesis of the chiral lipid tails found in natural LysE and MBA2. Substitution of chiral lipid tails in non-ribosomal peptide antibiotics is commonly performed and does not impair activity.^27–29^ Starting from 2-CT resin, Fmoc-SPPS was used to build out linear peptides up to the acylated N-terminal threonine. A Yamaguchi esterification was then used to install the ester motif,^11,30^ before further Fmoc-SPPS completed the peptide chain. Cleavage of protected peptides from resin was performed using HFIP in DCM, followed by a macrolactamization with PyBOP as the activating agent. Finally, global deprotection and purification by preparative reversed-phase HPLC yielded Hex-LysE (37) and Hex-MBA2 (38) in 12% and 16% overall yields respectively. These analogues retained full activity displaying MICs of 1.56–3.13 μg mL^−1^ against *S. aureus*.

### Antagonization assays with labelled PPQs

To confirm that ω-modifications to MK9 do not affect binding to LysE or MBA2, we performed antagonization assays, wherein peptides were mixed with increasing quantities of PPQ (up to two-fold excess), and their MICs against *S. aureus* tested (Table 1).^31^ This pre-mixing of peptide and target results in complexation if target binding occurs, sequestering antimicrobial activity. Only NBD-PPQs 30 and 31, and BODIPY–PPQs 32 and 33, were used in these (and subsequent) assays as we intended to use them for fluorescence microscopy. However, it is reasonable to assume that the other ω-modifications do not impact binding or activity, as we have found with other ω-modified polyprenyl systems.^19,25^ When peptides were premixed with increasing concentrations of CoQ10, no effect on antimicrobial activity was observed. In contrast, premixing peptides with menaquinone analogues MK4 or MK9, as well as labelled menaquinone derivatives NBD-MK9 or BODIPY-MK9, resulted in a dose-dependent loss in antimicrobial activity, with activity abolished (50 μg mL^−1^ or greater) in the presence of one or more equivalents of PPQ. These results were qualitative confirmation that labelled MK-analogues still retain peptide-binding, but we next sought to measure this binding strength quantitatively.

**Table 1:** Antagonization assays with menaquinone-binding peptides and PPQs. Peptides premixed with PPQ and activity then tested against *S. aureus*. Green = MIC change within margin of error (±two-fold), amber = four-fold change, and red = eight-fold or greater

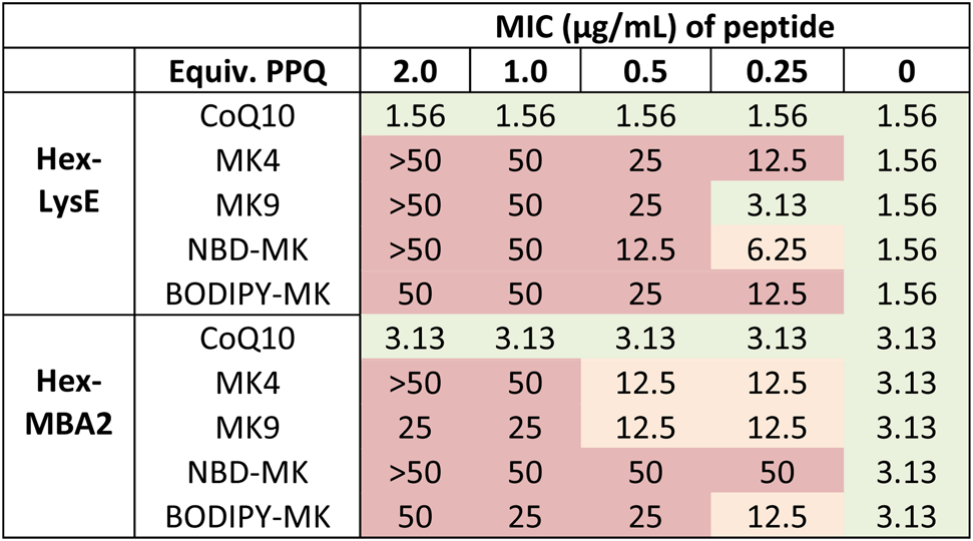

### Isothermal titration calorimetry with labelled PPQs

**Fig. 2:**
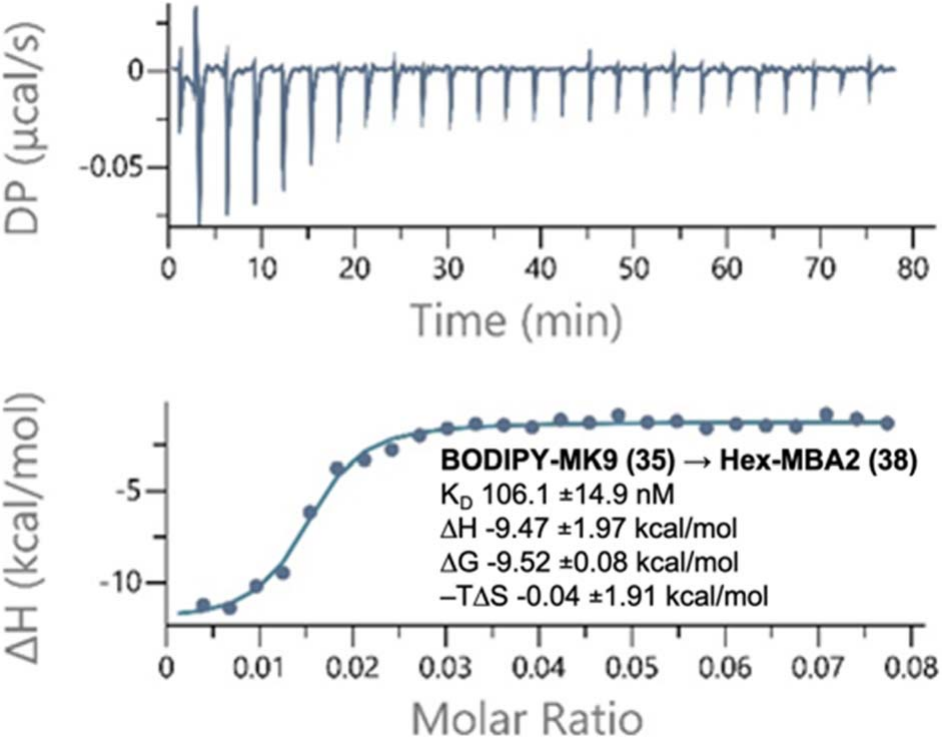
Representative binding thermogram and thermodynamic parameters generated by ITC experiments. BODIPY-MK9 DOPC/DOPG LUVs titrated into a solution of Hex-MBA2. Experiments were conducted in 20 mM HEPES and 50 mM NaCl at pH 7.5 at 25 °C. Top panel: raw signal as a function of time; bottom panel: normalized heat as a function of target : peptide molar ratio and fitted curve in the one set of sites model. Plots are representative of three experiments and results are presented as the average with the standard deviation. See Tables S1 and S2† for all thermodynamic parameters, Fig. S2–S16† for all other thermograms.

To quantify the strength of these binding interactions, we used isothermal titration calorimetry (ITC) (Fig. 2 and Tables S1, S2 and Fig. S2–S16†). Given that PPQs are found naturally embedded in cell membranes, we incorporated them into large unilamellar vesicles (LUVs, 100 nm) for these studies. LUVs composed of dioleoylphosphatidylglycerol (DOPG) and dioleoylphosphatidyl choline (DOPC) in a 1 : 1 ratio, and with 1.25% PPQ, were titrated into peptide solution, as has previously been performed for other antimicrobial peptides that bind to polyprenyl membrane targets (*e.g.*, lipid II).^32–34^ The 1 : 1 ratio of DOPG : DOPC was previously used for ITC with MBA-2.^11^ Extensive optimization was performed and the peptide concentrations that give the best signal/noise ratios were determined to be 20 μM for Hex-LysE and 160 μM for Hex-MBA2. As expected, no binding was detected between peptides and LUVs containing CoQ10. In contrast, strong binding affinities (*k*_D_) were found between Hex-LysE and MK4 (59 ± 10 nM), Hex-LysE and MK9 (80 ± 8 nM), Hex-MBA2 and MK4 (57 ± 9 nM) and Hex-MBA2 and MK9 (61 ± 7 nM). These binding affinities are consistent with previous work on MBA2 (ref. 11) but cannot be compared to previous LysE ITC data, which were determined in a non-membrane environment (DMSO).^10^ The strong binding affinity of these peptides for MK *vs.* CoQ is remarkable and may in part be due to an interaction between the essential d-Trp10 residue,^11^ and the MK benzene ring. We next looked at the binding affinities of peptides to NBD-MK9 and BODIPY-MK9. Consistent with the antagonization assays, peptides retained strong binding affinities (84–106 nM) to labelled PPQs, confirming that ω-modification does not disrupt headgroup binding. Analysis of the binding isotherms of both peptides to menaquinone derivatives suggested that rapid aggregation of peptide was occurring in the presence of menaquinone-containing LUVs. The saturation point, the point at which the isotherm levels out, indicating all available binding sites are occupied, was reached very quickly in these experiments. For Hex-LysE, this occurred at ∼0.2 : 1 MK9 : peptide, and at ∼0.04 : 1 MK9 : peptide for Hex-MBA2, suggesting that rapid peptide aggregation occurs in the presence of just a small amount of menaquinone. With fluorescently labelled CoQ10 and MK9 analogues in hand, we next sought to visualize this aggregation using fluorescence microscopy and giant unilamellar vesicles (GUVs).^25^

### Fluorescence microscopy with labelled PPQs

**Fig. 3:**
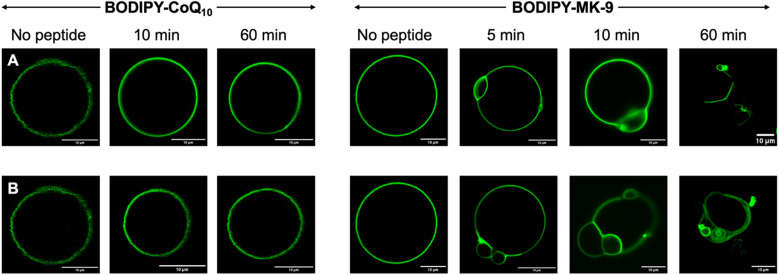
Fluorescence microscopy of GUVs containing BODIPY–PPQs. Row (A) shows effects of Hex-MBA2 addition and row (B) shows effects of Hex-LysE addition. Laser excitation wavelength = 488 nm, emission detection wavelength = 496–669 nm. Scale bar 10 μm in all panels.

GUVs constructed of 98% DOPC, 2% unlabelled target, and 0.1% labelled target were prepared to visualize how menaquinone-binding peptides interact with membranes. The 20 : 1 ratio of unlabelled : labelled PPQ was selected based on previous work with peptide antibiotic plectasin.^25^ Higher concentrations of labelled PPQs can result in image saturation, self-quenching and/or photobleaching, therefore unlabelled PPQs are added so the antibiotic target is present in sufficient concentration to visualize peptide effects on membranes. Imaging showed uniform distribution of NBD (Fig. S17†) and BODIPY (Fig. 3) PPQs in GUVs. BODIPY–PPQs proved superior for imaging, as for NBD-PPQs, trying to minimize the fluorophore's exposure to the laser to avoid photobleaching sometimes made it difficult to find and record quality images of GUVs. Therefore, BODIPY–PPQs were taken forward for mechanism of action studies on peptide antibiotics. First, to demonstrate selective peptide binding of MK9 over CoQ10, and to show that the effects observed are not because of a non-specific binding interaction to the polyprenyl chain, or the fluorophore unit of the probes, peptides were added to BODIPY-CoQ10 GUVs. No effect was observed upon addition of either Hex-LysE or Hex-MBA2, even after 60 min. Upon addition of Hex-LysE or Hex-MBA2 to BODIPY-MK9 GUVs, effects were seen almost instantly. Within five min, accumulation and localization of BODIPY-MK9 is observed. As time progresses, the accumulation of BODIPY-MK9 led to distortion of membrane curvature and vesiculation. As the vesiculation continues, the end result is whole membrane collapse. By 60 min, significant aggregation and membrane rupture was observed. These results are consistent with the antagonization assays and ITC measurements and without labelled PPQs, this experiment would not have been possible. Placement of a fluorophore on LysE or MBA2 would likely disrupt quinone-binding and would not reliably show quinone aggregation in membranes as peptide will still be fluorescent even when unbound. Alternatively, fluorescent phospholipids would provide no information on PPQ position within membranes. This work therefore highlights the utility of our labelled PPQs in mechanism of action studies of menaquinone-binding peptide antibiotics.

## Conclusions

In summary, we here report a concise and versatile synthetic strategy for the direct labelling of native polyprenyl quinones (PPQs), enabling the installation of a wide array of reporter groups at the ω-terminus of the polyprenyl tail. The method proceeds through ω-epoxidation and oxidative cleavage and can be applied to both menaquinone and ubiquinone scaffolds. Two routes were developed to introduce functionality: reductive amination, and Cu-catalyzed alkyne–azide cycloaddition (CuAAC). The reductive amination approach required seven steps, with overall yields 2–6%. Comparable overall yields were found for the CuAAC process, which required ten steps in total. The CuAAC approach proved superior for late-stage modification as azido-quinones can be synthesized first, with CuAAC the final step for label attachment. These analogues also open the possibility of strain-promoted azide–alkyne cycloaddition in native bacterial membranes. Using these strategies, we prepared a panel of PPQ analogues bearing fluorophores, quenchers, ^19^F nuclei, and affinity tags. The resulting compounds retain their native redox headgroups, and hydrophobic tail, and are compatible with incorporation into model membranes. Fluorescently labelled menaquinone analogues NBD-MK9 and BODIPY-MK9 were prepared in good yields and used as probes to investigate how menaquinone-binding antimicrobial peptides interact with their target. Two novel peptide analogues, Hex-LysE and Hex-MBA2, were synthesized for this purpose and retained full antimicrobial activity against *S. aureus*.

To confirm that ω-modification did not disrupt PPQ function, we performed antagonization assays, wherein labelled and unlabelled PPQs were pre-mixed with peptide prior to MIC testing. While ubiquinone analogues showed no effect, both natural and labelled menaquinones abolished antimicrobial activity, consistent with strong and specific target binding. These findings were supported by isothermal titration calorimetry, which revealed nanomolar binding affinities between MK9 (labelled and unlabelled) and both peptides. Furthermore, the binding isotherms indicated peptide aggregation occurred at low PPQ : peptide ratios, suggesting a clustering-type interaction. To visualize these interactions, we incorporated labelled PPQs into giant unilamellar vesicles (GUVs) and performed fluorescence microscopy. In the presence of BODIPY-CoQ10, no changes in vesicle morphology were observed following peptide addition. In contrast, BODIPY-MK9 was rapidly recruited to specific membrane regions, followed by membrane curvature, budding, and eventual rupture. These observations were consistent for both Hex-LysE and Hex-MBA2 and provide new insights into how these peptide antibiotics kill bacteria at a macromolecular level.

Our approach offers a direct, modular route to labelled PPQs that is broadly applicable across different redox quinone systems. The resulting probes retain their biological function and enable mechanistic studies that would otherwise be inaccessible. We demonstrate how fluorescently labelled menaquinones can be used to track quinone behaviour in membranes and reveal the dynamic effects of quinone-targeting antibiotics. Further applications of these probes are expected to include live-cell imaging, mechanistic dissection of antibiotic mechanism, and identification of novel quinone-binding partners.

## Author contributions

EJM performed all experimental work described, including PPQ synthesis, peptide synthesis, antagonization assays, isothermal titration calorimetry (ITC) and fluorescence microscopy (FM). RAMvB and MW provided training and supervision for FM experiments. PI and NIM provided training and supervision for ITC experiments. SAC and EJM designed and lead the study. SAC and EJM prepared the manuscript and ESI† with input from all other authors.

## Conflicts of interest

No conflicts of interest to declare.

## Supplementary Material

SC-OLF-D5SC03363B-s001

## Data Availability

The data supporting this article have been included as part of the ESI.†

## References

[cit1] Franza T., Gaudu P. (2022). Res. Microbiol..

[cit2] Nowicka B., Kruk J. (2010). Biochim. Biophys. Acta, Bioenerg..

[cit3] Gould K. (2016). J. Antimicrob. Chemother..

[cit4] Hutchings M. I., Truman A. W., Wilkinson B. (2019). Curr. Opin. Microbiol..

[cit5] Kurosu M., Begari E. (2010). Molecules.

[cit6] Fry M., Pudney M. (1992). Biochem. Pharmacol..

[cit7] Itoh H., Tokumoto K., Kaji T., Paudel A., Panthee S., Hamamoto H., Sekimizu K., Inoue M. (2018). J. Org. Chem..

[cit8] Kurosu M., Crick D. C. (2009). Med. Chem..

[cit9] Bagheri S., Haddadi R., Saki S., Kourosh-Arami M., Rashno M., Mojaver A., Komaki A. (2023). Front. Neurosci..

[cit10] Hamamoto H., Urai M., Ishii K., Yasukawa J., Paudel A., Murai M., Kaji T., Kuranaga T., Hamase K., Katsu T. (2015). et al.. Nat. Chem. Biol..

[cit11] Li L., Koirala B., Hernandez Y., MacIntyre L. W., Ternei M. A., Russo R., Brady S. F. (2022). Nat. Microbiol..

[cit12] Greene L. E., Godin R., Cosa G. (2016). J. Am. Chem. Soc..

[cit13] Komatsu H., Shindo Y., Oka K., Hill J. P., Ariga K. (2014). Angew. Chem., Int. Ed..

[cit14] Suhara Y., Abe S., Murakami A., Shimomura Y., Nakagawa K., Kamao M., Tsugawa N., Okano T. (2008). Tetrahedron.

[cit15] Murai M., Yamashita T., Senoh M., Mashimo Y., Kataoka M., Kosaka H., Matsuno-Yagi A., Yagi T., Miyoshi H. (2010). Biochemistry.

[cit16] Suhara Y., Murakami A., Kamao M., Mimatsu S., Nakagawa K., Tsugawa N., Okano T. (2007). Bioorg. Med. Chem. Lett..

[cit17] Goto M., Nishiyama A., Yamaguchi T., Watanabe K., Fujii K., Watanabe Y., Doi H. (2019). J. Labelled Compd. Radiopharm..

[cit18] Bu M.-J., Cai C., Gallou F., Lipshutz B. H. (2018). Green Chem..

[cit19] Cochrane R. V. K., Alexander F. M., Boland C., Fetics S. K., Caffrey M., Cochrane S. A. (2020). Chem. Commun..

[cit20] Craven T. W., Nolan M. D., Bailey J., Olatunji S., Bann S. J., Bowen K., Ostrovitsa N., Da Costa T. M., Ballantine R. D., Weichert D. (2024). et al.. ACS Chem. Biol..

[cit21] Wiktor M., Weichert D., Howe N., Huang C. Y., Olieric V., Boland C., Bailey J., Vogeley L., Stansfeld P. J., Buddelmeijer N. (2017). et al.. Nat. Commun..

[cit22] Yamakoshi H., Dodo K., Palonpon A., Ando J., Fujita K., Kawata S., Sodeoka M. (2012). J. Am. Chem. Soc..

[cit23] Sie C. J., Patteti V., Yang Y. R., Mong K. K. T. (2018). Chem. Commun..

[cit24] Cochrane S. A., Li X., He S., Yu M., Wu M., Vederas J. C. (2015). J. Med. Chem..

[cit25] Jekhmane S., Derks M. G. N., Maity S., Slingerland C. J., Tehrani K. H. M. E., Medeiros-Silva J., Charitou V. J., Ammerlaan D., Fetz C., Consoli N. A. (2024). et al.. Nat. Microbiol..

[cit26] Besanceney-Webler C., Jiang H., Zheng T., Feng L., Soriano Del Amo D., Wang W., Klivansky L. M., Marlow F. L., Liu Y., Wu P. (2011). Angew. Chem., Int. Ed..

[cit27] Ballantine R. D., Al Ayed K., Bann S. J., Hoekstra M., Martin N. I., Cochrane S. A. (2022). RSC Med. Chem..

[cit28] Cochrane S. A., Surgenor R. R., Khey K. M. W., Vederas J. C. (2015). Org. Lett..

[cit29] Cochrane S. A., Lohans C. T., Brandelli J. R., Mulvey G., Armstrong G. D., Vederas J. C. (2014). J. Med. Chem..

[cit30] Al Ayed K., Ballantine R. D., Hoekstra M., Bann S. J., Wesseling C. M. J., Bakker A. T., Zhong Z., Li Y., Brüchle N. C., van der Stelt M., Cochrane S. A., Martin N. I. (2022). Chem. Sci..

[cit31] Bann S. J., Ballantine R. D., Li Y.-X., Qian P.-Y., Cochrane S. A. (2019). J. Med. Chem..

[cit32] Buijs N. P., Vlaming H. C., Kotsogianni I., Arts M., Willemse J., Duan Y., Alexander F. M., Cochrane S. A., Schneider T., Martin N. I. (2024). Proc. Natl. Acad. Sci. U. S. A..

[cit33] Kotsogianni I., Wood T. M., Alexander F. M., Cochrane S. A., Martin N. I. (2021). ACS Infect. Dis..

[cit34] Chiorean S., Antwi I., Carney D. W., Kotsogianni I., Giltrap A. M., Alexander F. M., Cochrane S. A., Payne R. J., Martin N. I., Henninot A., Vederas J. C. (2019). ChemBioChem.

