# Supplementary material for: Chemical diversification of polyprenyl quinones for mechanistic studies on menaquinone-binding peptide antibiotics
Source: Chem Sci. 2025 Jul 3;16(30):13629–35. doi: 10.1039/d5sc03363b (PMC12243021; doi:10.1039/d5sc03363b)

Supplementary Information (SI) for Chemical Science.
This journal is © The Royal Society of Chemistry 2025

Supplementary Information

FOR

Chemical Diversification of Polyprenyl Quinones for Mechanistic Studies on Menaquinone-Binding Peptide Antibiotics

TABLE OF CONTENTS

S3 Supplemental Figures, Tables and Schemes

S14 Experimental Details

S22 Synthetic Chemistry Procedures and Compound Characterization

S83 References

S84 NMR Spectra

S298 Analytical HPLC Spectra

S300 IR Spectra

Supplemental Figures, Tables and Schemes

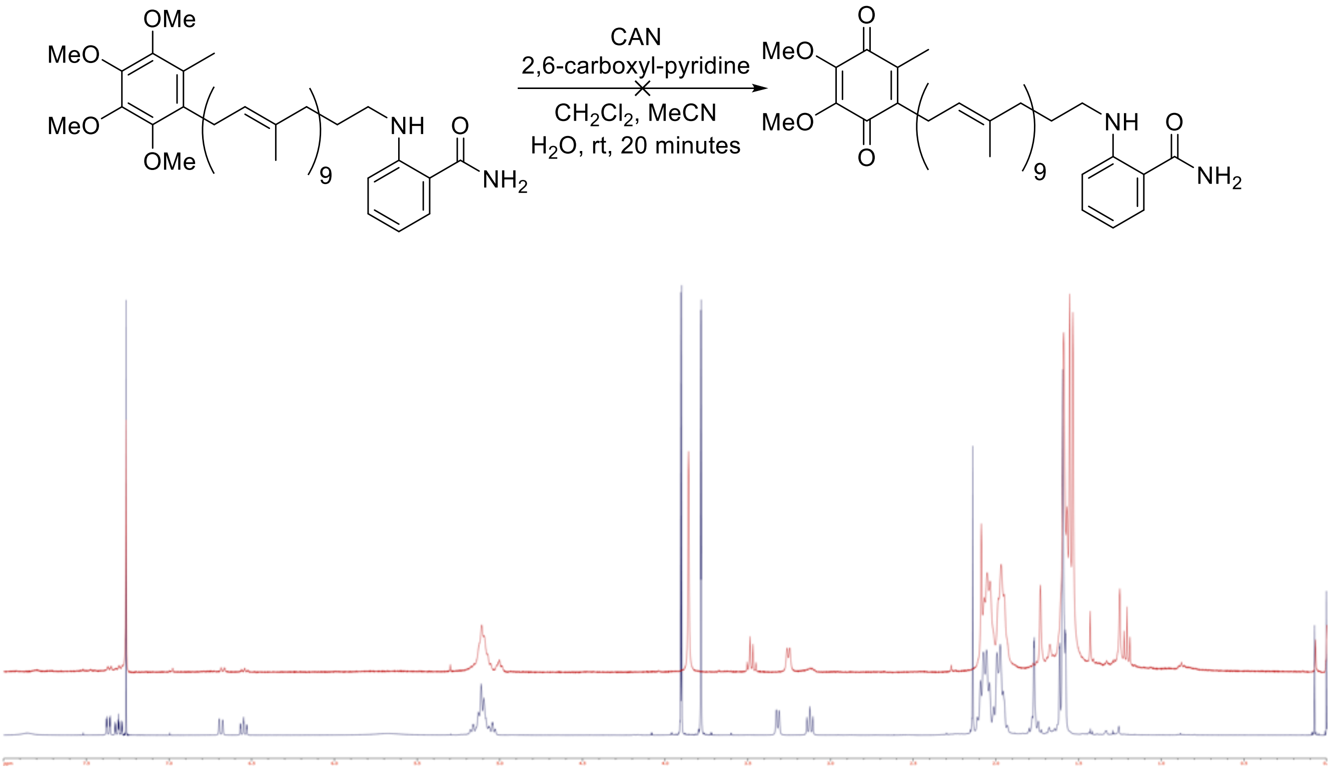

Figure S1. Unsuccessful attempt at deprotection of Me_2-CoQ10-ω-2AB (**12**) with ceric ammonium nitrate and 2,6-carboxyl-pyridine. ^1H NMR spectrum (in $CDCl_3$) of Me2-CoQ10-ω-2AB (**12**) before (blue) and after (red) reaction shows significant loss of 2AB group.

Table S1. Thermodynamic parameters for Hex-MBA2 (**38**) binding to different PPQs. Standard deviation in brackets, NB = no binding detected. Results are presented as the average of three experiments, with the standard deviation shown in parentheses.

**PPQ**	**Hex-MBA2**				
	**K_D (nM)**	**N ($x10^{-2}$ Sites)**	**ΔH (kcal mol^{-1})**	**–TΔS (kcal mol^{-1})**	**ΔG (kcal mol^{-1})**
MK4	56.9	2.47	– 5.63	– 4.26	– 9.89
	(± 8.50)	(± 0.06)	(± 1.08)	(± 0.99)	(± 0.09)
MK9	61.1	2.33	– 5.02	– 4.83	– 9.85
	(± 7.05)	(± 0.15)	(± 1. 34)	(± 1.38)	(± 0.07)
MK9-NBD	84.3	1.93	– 8.43	– 1.23	– 9.65
	(± 4.67)	(± 0.25)	(± 0.69)	(± 0.70)	(0.03)
MK9-BODIPY	106.1	1.37	– 9.47	– 0.04	– 9.52
	(± 14.9)	(± 0.15)	(± 1.97)	(± 1.91)	(± 0.08)
CoQ10	NB	NB	NB	NB	NB

Table S2. Thermodynamic parameters for Hex-LysE (**37**) binding to different PPQs. Standard deviation in brackets, NB = no binding detected, ND = not determined due to insufficient quantities of required materials. Results are presented as the average of three experiments, with the standard deviation shown in parentheses.

**PPQ**	**Hex-LysE**				
	**K_D (nM)**	**N (Sites)**	**ΔH (kcal mol^{-1})**	**–TΔS (kcal mol^{-1})**	**ΔG (kcal mol^{-1})**
MK4	59.2 (± 9.65)	0.18 (± 0.03)	– 8.49 (± 1.48)	– 1.37 (± 1.46)	– 9.86 (± 0.10)
MK9	80.3 (± 7.60)	0.30 (± 0.09)	– 8.99 (± 1.33)	– 0.68 (± 1.30)	– 9.68 (± 0.06)
MK9-NBD	101.4 (±8.47)	0.15 (± 0.04)	– 5.02 (± 1.05)	– 4.52 (±1.02)	– 9.54 (± 0.05)
MK9-BODIPY	ND	ND	ND	ND	ND
CoQ10	NB	NB	NB	NB	NB

*ND = not determined due to insufficient quantities of materials

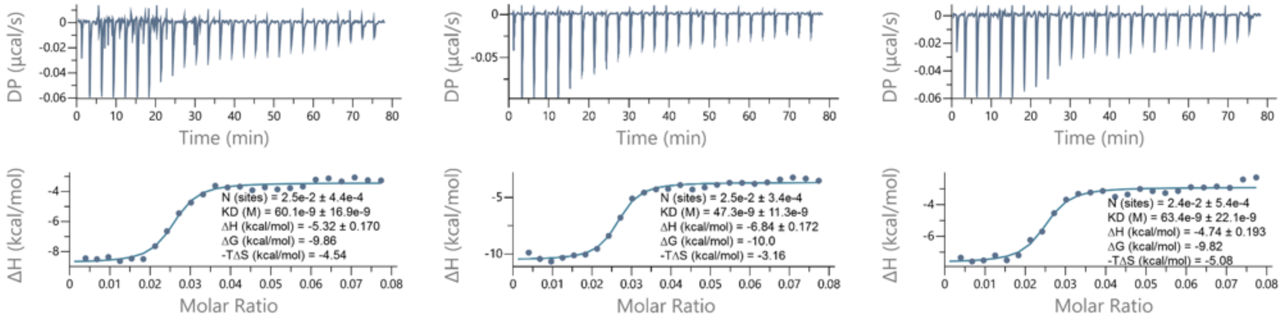

Figure S2. Titration of 1.25 mol% MK4 (**2**)-containing 1:1 DOPC:DOPG (5 mM, HEPES buffer) LUVs into Hex-LysE (**37**) (20 μM, HEPES buffer) at pH 7.5, 25 °C. Top panel: raw signal as a function of time; Bottom panel: normalized heat as a function of PPQ:peptide molar ratio and fitted curve in the one set of sites model. Three experimental repeats are shown.

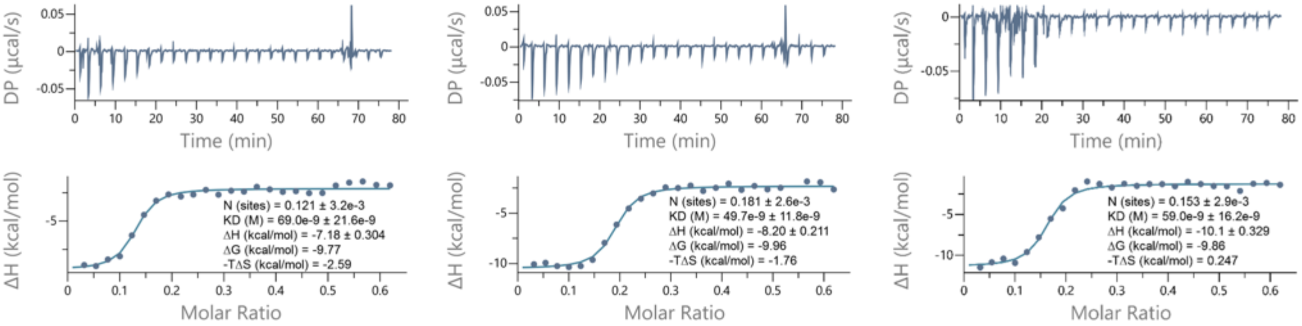

Figure S3. Titration of 1.25 mol% MK4 (**2**)-containing 1:1 DOPC:DOPG (5 mM, HEPES buffer) LUVs into Hex-MBA2 (**38**) (160 μM, HEPES buffer) at pH 7.5, 25 °C. Top panel: raw signal as a function of time; Bottom panel: normalized heat as a function of PPQ:peptide molar ratio and fitted curve in the one set of sites model. Three experimental repeats are shown.

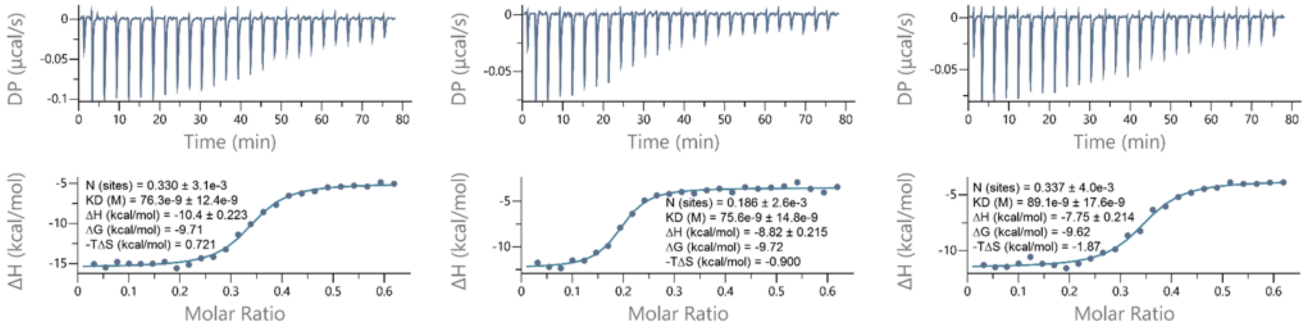

Figure S4. Titration of 1.25 mol% MK9 (**3**)-containing 1:1 DOPC:DOPG (5 mM, HEPES buffer) LUVs into Hex-LysE (**37**) (20 µM, HEPES buffer) at pH 7.5, 25 °C. Top panel: raw signal as a function of time; Bottom panel: normalized heat as a function of PPQ:peptide molar ratio and fitted curve in the one set of sites model. Three experimental repeats are shown.

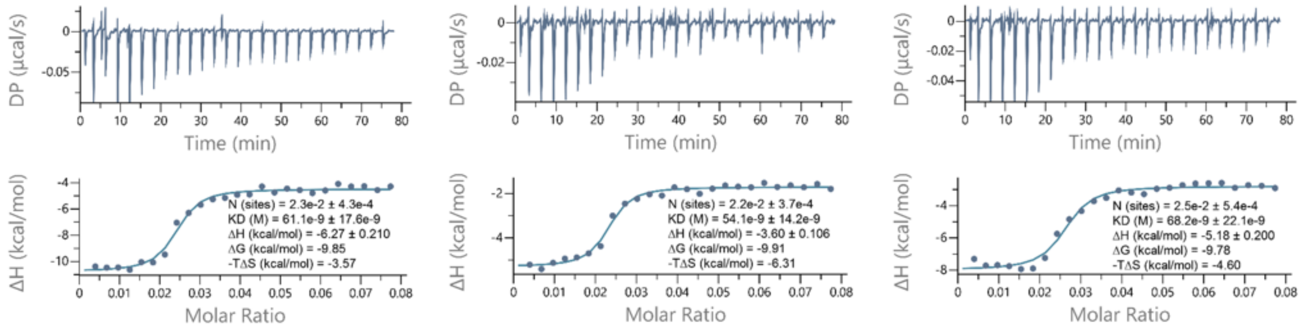

Figure S5. Titration of 1.25 mol% MK9 (**3**)-containing 1:1 DOPC:DOPG (5 mM, HEPES buffer) LUVs into Hex-MBA2 (**38**) (160 µM, HEPES buffer) at pH 7.5, 25 °C. Top panel: raw signal as a function of time; Bottom panel: normalized heat as a function of PPQ:peptide molar ratio and fitted curve in the one set of sites model.

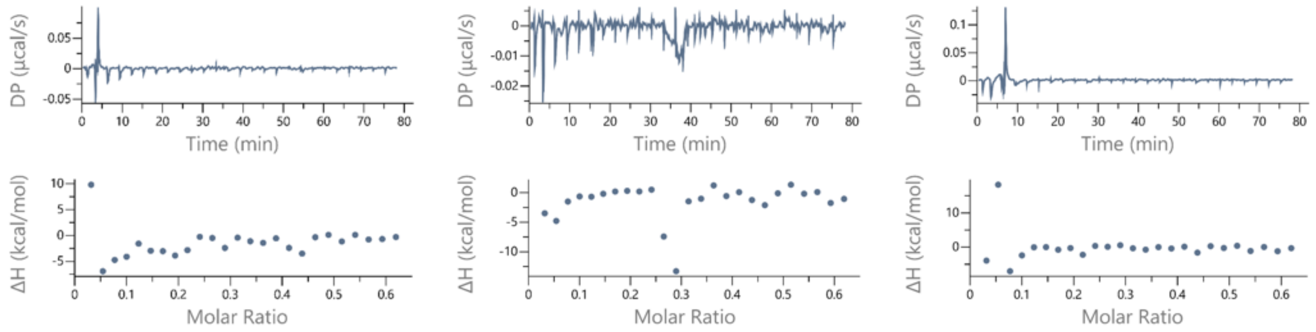

Figure S6. Titration of 1.25 mol% CoQ10 (**1**)-containing 1:1 DOPC:DOPG (5 mM, HEPES buffer) LUVs into Hex-LysE (**37**) (20 µM, HEPES buffer) at pH 7.5, 25 °C. Top panel: raw signal as a function of time; Bottom panel: normalized heat as a function of PPQ:peptide molar ratio and fitted curve in the one set of sites model. Three experimental repeats are shown.

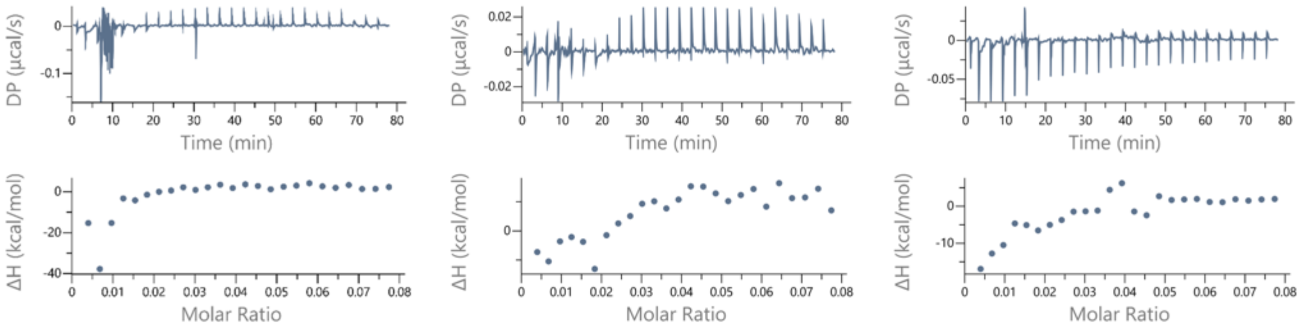

Figure S7. Titration of 1.25 mol% CoQ10 (**1**)-containing 1:1 DOPC:DOPG (5 mM, HEPES buffer) LUVs into Hex-MBA2 (**38**) (160 µM, HEPES buffer) at pH 7.5, 25 °C. Top panel: raw signal as a function of time; Bottom panel: normalized heat as a function of PPQ:peptide molar ratio and fitted curve in the one set of sites model. Three experimental repeats are shown.

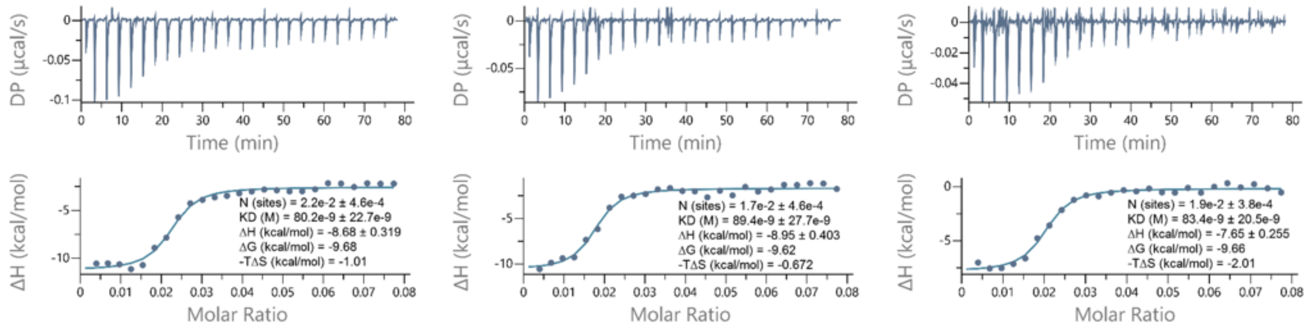

Figure S8. Titration of 1.25 mol% MK9-ω-NBD (**31**)-containing 1:1 DOPC:DOPG (5 mM, HEPES buffer) LUVs into Hex-LysE (**37**) (20 µM, HEPES buffer) at pH 7.5, 25 °C. Top panel: raw signal as a function of time; Bottom panel: normalized heat as a function of PPQ:peptide molar ratio and fitted curve in the one set of sites model. Three experimental repeats are shown.

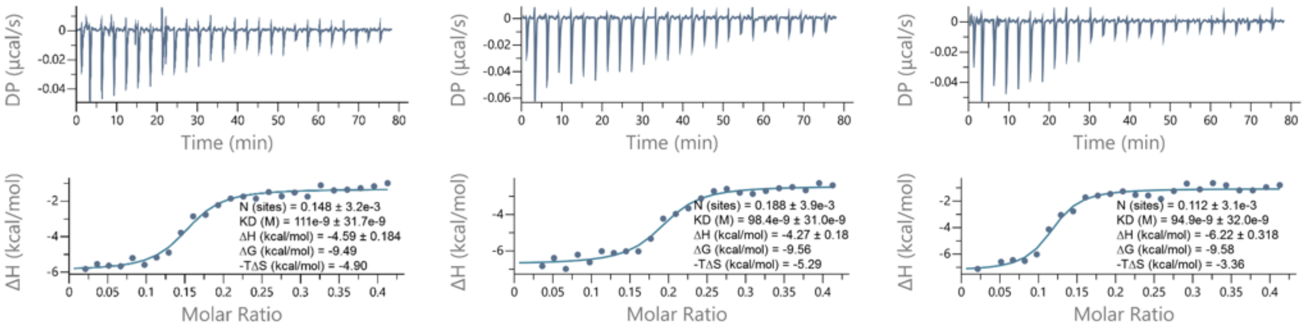

Figure S9. Titration of 1.25 mol% MK9-ω-NBD (**31**)-containing 1:1 DOPC:DOPG (5 mM, HEPES buffer) LUVs into Hex-MBA2 (**38**) (160 µM, HEPES buffer) at pH 7.5, 25 °C. Top panel: raw signal as a function of time; Bottom panel: normalized heat as a function of PPQ:peptide molar ratio and fitted curve in the one set of sites model. Three experimental repeats are shown.

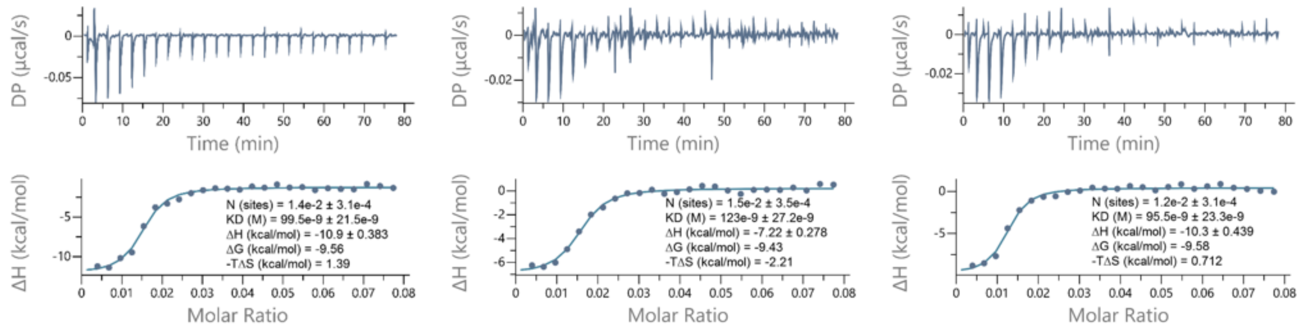

Figure S10. Titration of 1.25 mol% MK9-ω-BODIPY (**35**)-containing 1:1 DOPC:DOPG (5 mM, HEPES buffer) LUVs into Hex-MBA2 (**38**) (160 µM, HEPES buffer) at pH 7.5, 25 °C. Top panel: raw signal as a function of time; Bottom panel: normalized heat as a function of PPQ:peptide molar ratio and fitted curve in the one set of sites model. Three experimental repeats are shown.

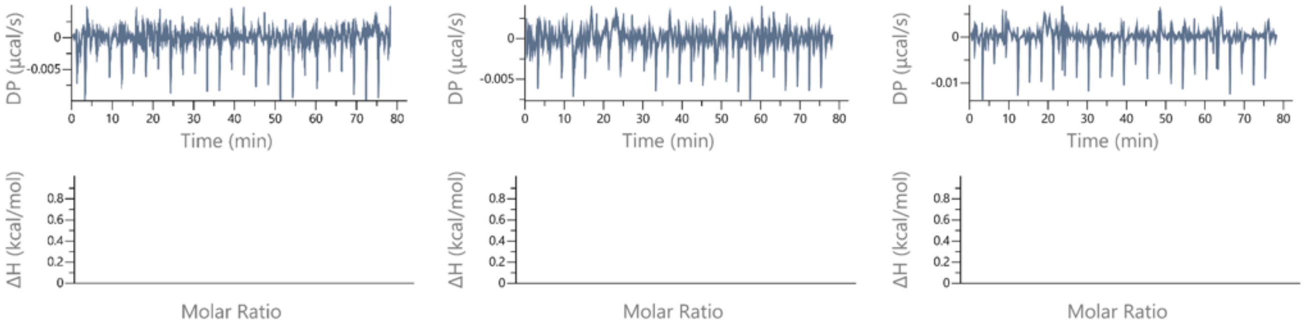

Figure S11. ITC control. Titration of 1.25 mol% MK4 (**2**)-containing 1:1 DOPC:DOPG (5 mM, HEPES buffer) LUVs into HEPES buffer at pH 7.5, 25 °C. Top panel: raw signal as a function of time. Three experimental repeats are shown.

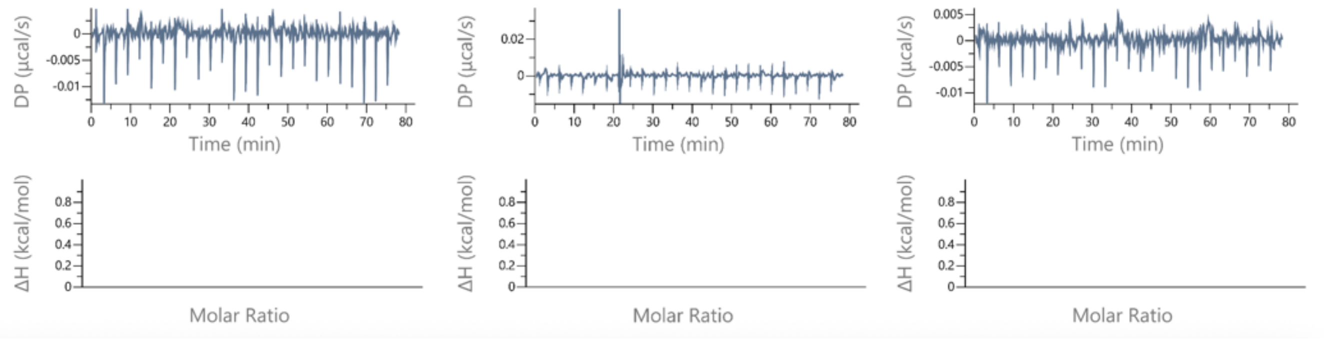

Figure S12. ITC control. Titration of 1.25 mol% MK9 (**3**)-containing 1:1 DOPC:DOPG (5 mM, HEPES buffer) LUVs into HEPES buffer at pH 7.5, 25 °C. Top panel: raw signal as a function of time. Three experimental repeats are shown.

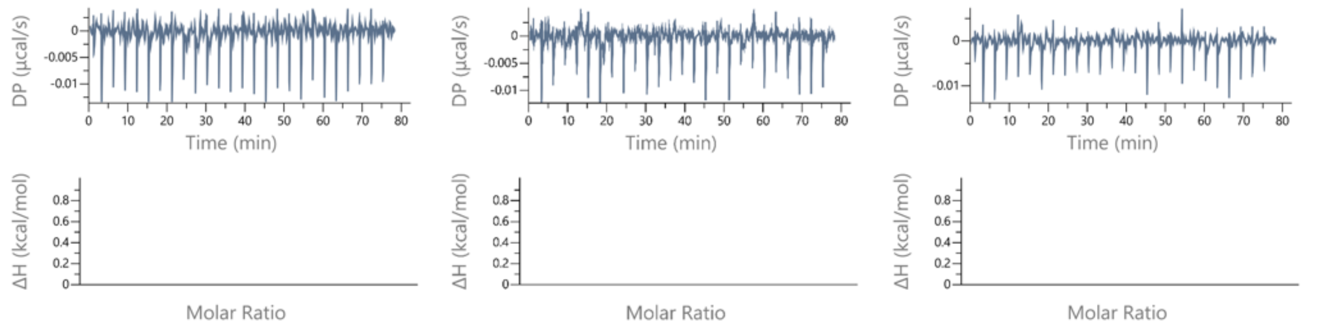

Figure S13. ITC control. Titration of 1.25 mol% MK9-ω-NBD (**31**)-containing 1:1 DOPC:DOPG (5 mM, HEPES buffer) LUVs into HEPES buffer at pH 7.5, 25 °C. Top panel: raw signal as a function of time. Three experimental repeats are shown.

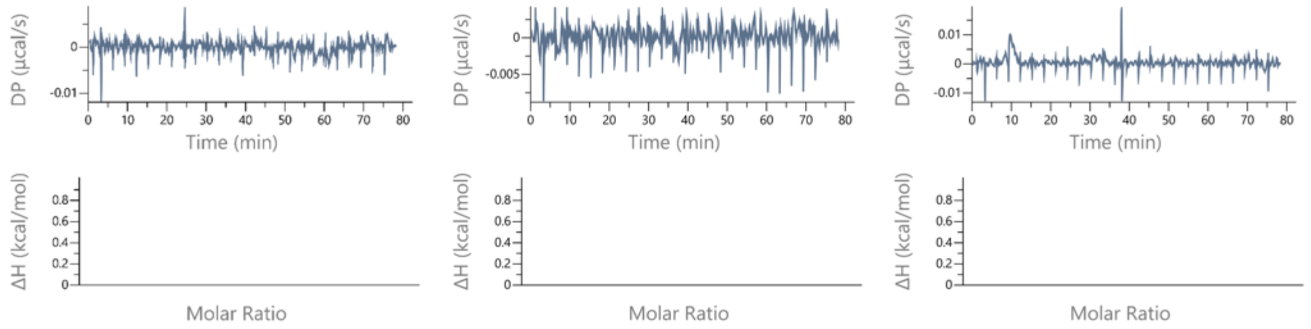

Figure S14. ITC control. Titration of 1.25 mol% MK9-ω-BODIPY (**35**)-containing 1:1 DOPC:DOPG (5 mM, HEPES buffer) LUVs into HEPES buffer at pH 7.5, 25 °C. Top panel: raw signal as a function of time. Three experimental repeats are shown.

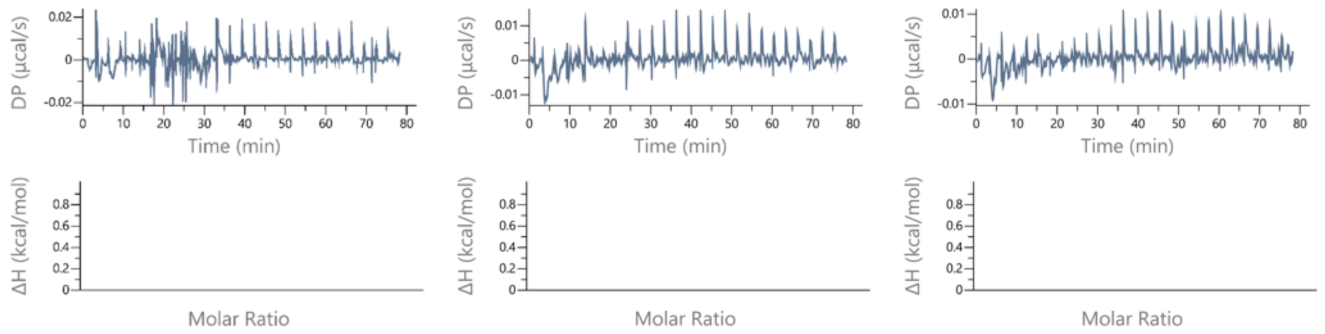

Figure S15. ITC control. Titration of 1:1 DOPC:DOPG (5 mM, HEPES buffer) LUVs into Hex-LysE (**37**) (20 µM, HEPES buffer) at pH 7.5, 25 °C. Top panel: raw signal as a function of time. Three experimental repeats are shown.

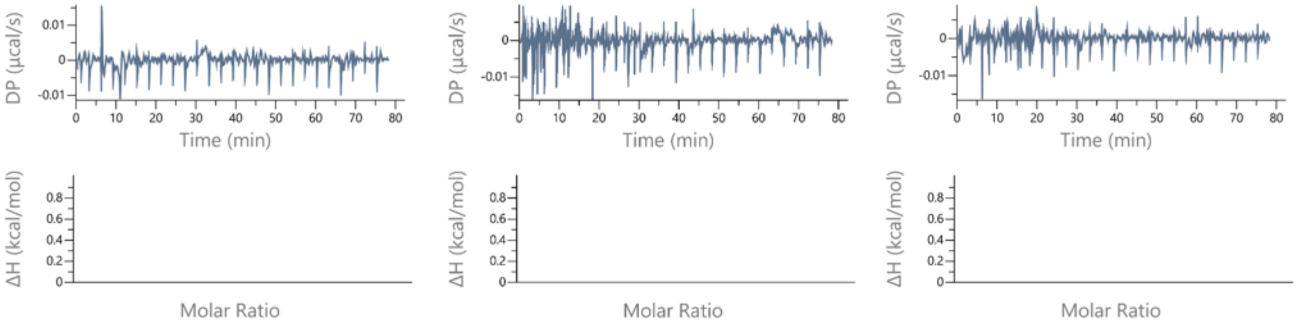

Figure S16. ITC control. Titration of 1:1 DOPC:DOPG (5 mM, HEPES buffer) LUVs into Hex-MBA2 (**38**) (160 µM, HEPES buffer) at pH 7.5, 25 °C. Top panel: raw signal as a function of time. Three experimental repeats are shown.

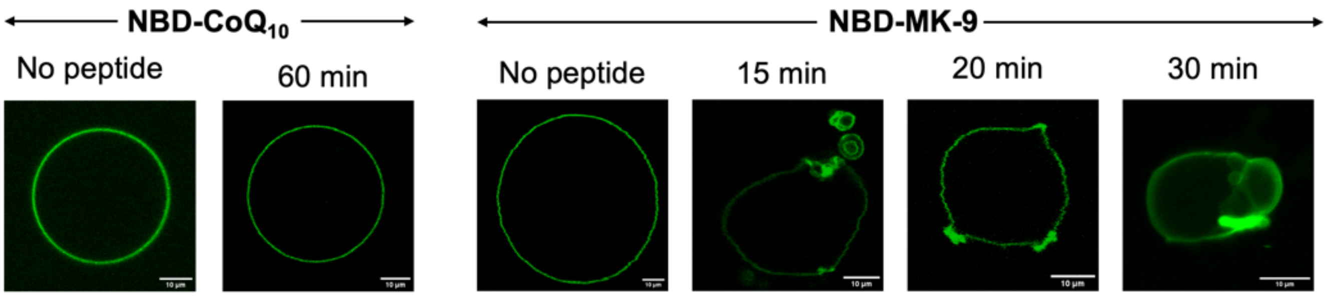

Figure S17. Fluorescence microscopy of GUVs containing NBD-PPQs, showing effects of Hex-LysE addition. Laser excitation wavelength = 488 nm, emission detection wavelength = 496 – 669 nm. Scalebar 10 µm in all panels.

Experimental Details

General Experimental Details

Nuclear Magnetic Resonance Spectroscopy (NMR)

^{1}H NMR were recorded at 600 MHz and 400 MHz using a Bruker 600 or Bruker 400 spectrometer, processed by TopSpin software. Residual solvent peaks were used as an internal reference for ^{1}H NMR spectra ($CDCl_3$ δ 7.26 ppm). Coupling constants are quoted to the nearest 0.1 Hz. ^{13}C NMR spectra were recorded at 101 MHz using a Bruker AVANCE 600, Bruker or Bruker AVANCE 400 spectrometer, processed by TopSpin software. Solvent peaks were used as an internal reference for ^{13}C NMR spectra ($CDCl_3$ δ 77.16 ppm). ssNMR experiments were recorded on at 1.2 gHz or 800 mHz using a Bruker Ascend 1.2 gHz or Bruker 800 spectrometer, processed by TopSpin software. iNMR, TopSpin or POKY software was used for processing and viewing NMR data. Due to the extremely hydrophobic nature of the described polyprenyls, removal of trace solvents used during purification and hydrocarbon impurities from these solvents proved very difficult, even under high vacuum for several days. Such impurities appear below 1.5 ppm and are present in most 1H-NMR spectra

Mass Spectroscopy (MS)

High-resolution mass spectra were recorded on a Waters LCT Premier ToF mass spectrometer instrument using Electrospray Ionisation (ESI+) or on a Shimadzu LCMS system. The LCMS system contained a Shimadzu Nexera X2 UHPLC system with a Waters Acquity HSS C18 column (2.1 × 100 mm, 1.8 µm) at 30 °C and equipped with a diode array detector. The following solvent system, at a flow rate of 0.5 mL/min, was used: solvent A, 0.1 % formic acid in water; solvent B, 0.1 % formic acid in acetonitrile. Gradient elution was as follows: 95:5 (A/B) for 1 min, 95:5 to 15:85 (A/B) over 6 min, 15:85 to 0:100 (A/B) over 1 min, 0:100 (A/B) for 3 min, then reversion back to 95:5 (A/B) for 3 min. This system was connected to a Shimadzu

9030 QTOF mass spectrometer (ESI ionisation) calibrated internally with Agilent's API-TOF reference mass solution kit (5.0 mM purine, 100.0 mM ammonium trifluoroacetate and 2.5 mM hexakis(1H,1H,3H-tetrafluoropropoxy)phosphazine) diluted for synthesis of a mass count of 10000.

Analytical Thin Layer Chromatography (TLC)

Analytical TLC was performed with Merck silica plates, precoated with silica gel 60 F254 (0.2 mm). Visualisation was effected by one or more of the following methods: under UV fluorescence (254nm), potassium permanganate stain (1.5 g $KMnO_4$, 10 g K_2CO_3, 1.25 mL 10% NaOH (aq), 200 mL water), ninhydrin stain (1.5 g, ninhydrin, 100 mL *n*-butanol, 3.0 mL acetic acid) and/or phosphomolybdic acid (PMA) stain (10 g phosphomolybdic acid, 100 mL absolute ethanol).

Spectrofluorometry

Resin loading and OD_{600} values were determined using a DeNovix DS 11FX+ spectrophotometer/fluorometer.

Experimental Procedures, Reagents and Glassware

All commercially available reagents and solvents were purchased from Merck Ltd., Fisher Scientific Ltd., Fluorochem Ltd., Alfa Aesar Ltd., ABCR Ltd., Jenabioscience Ltd and Cambridge Isotopes Ltd. Air/moisture sensitive reactions were conducted under a positive pressure of dry argon and the glassware was kept overnight in an oven or flame dried prior to use. All dry solvents were prepared from HPLC grade solvents by addition of 20% w/v freshly activated 3 Å molecular sieves under an inert atmosphere and standing for 24 h. The yields reported in this paper are shown as isolated yields, unless stated otherwise. Concentration

under reduced pressure/vacuum was performed using a Bϋchi rotary evaporator with a water bath temperature of 40 °C.

Flash Chromatography

Flash chromatography was employed by Fluorochem 60 40-63 micron silica gel.

N-Bromosuccinimide (NBS) Purification

N-Bromosuccinimide (10 g) was recrystallised from H_2O (100 mL) to give *N*-bromosuccinimide as a colourless crystalline solid.

Tosyl chloride (TsCl) Purification

Tosyl chloride (10 g) was dissolved in $CHCl_3$ (25 mL) and filtered *in vacuo*. The filtrate was diluted in hexane (125 mL) and the precipitate filtered and dried *in vacuo* to give tosyl chloride as a colourless crystalline solid.

General Procedures for Solid-Phase Peptide Synthesis (SPPS)

Resin loading

2-Chlorotrityl (2-CT) resin (1 equiv.) was swollen in CH_2Cl_2 (2 mL for every 0.1 g resin) and Fmoc-AA-OH (4 equiv.) and DIPEA (16 equiv.) was added and the reaction mixture stirred at ambient temperature under argon for 2 h. After which, the resin was drained and washed with CH_2Cl_2 (3 x 5 mL). The resin capping solution (85:10:5 CH_2Cl_2:MeOH:DIPEA, 1 mL for every 0.1 g resin) was added and the reaction mixture stirred at ambient temperature for 1 h. The resin was then drained and washed with CH_2Cl_2 (3 x 5 mL) and dried under a stream of air to give loaded resin.

Resin loading test

A small portion of resin (10 – 15 mg) was suspended in 2% DBU in DMF (2 mL) and agitated for 30 min. This solution was diluted to 10 mL with MeCN. A portion of this solution (2 mL) was further diluted to 25 mL with MeCN. A reference solution without resin was also prepared in the same way. Optical density at 304 nm was recorded thrice for the reference and the sample (blank sample = MeCN). The resin loading was calculated according to the following equation, the average of the 3 values taken as the resin loading.

$$Fmoc\ loading\ (mmol/g) = \left(Abs_{sample} - Abs_{reference}\right)x\ (16.4/resin\ mass\ (mg))$$

Peptide Synthesis General Methods

Manual solid phase peptide synthesis was performed in a 20 mL glass fritted column with a B24 adaptor, fitted with a T-joint and three-way T-bore Teflon stopcock. The pre-swollen 2-CT resin was pre-swollen by stirring in a suspension of CH_2Cl_2 (2 mL for every 100 mg resin) for 20 min. The resin was then drained and washed twice withCH_2Cl_2 (2 mL for every 100 mg resin). Fmoc deprotection were performed by stirring the resin at ambient temperature in 20% 4-methyl-piperidine in DMF (2 mL for every 100 mg resin) for two rounds of 1 min, followed by one round of 5 min. The resin was drained and rinsed with DMF (2 mL for every 100 mg resin) between each deprotection round. The deprotection was monitored by spotting the deprotection solution filtrate on a silica TLC plate and visualising the benzofulvene by-product with a 254 nm UV lamp. After the final deprotection round, the resin was washed with DMF (3 x 4 mL for every 100 mg resin). The amino acid coupling solution was prepared by dissolving the asymmetrically protected amino acid (5 equiv.) and HATU (5 equiv.) in DMF (2 mL for every 100 mg resin) before adding DIPEA (10 equiv.). The coupling solution was then added to the deprotected resin and stirred at ambient temperature for 1 h (2 h to overnight for sterically

hindered couplings). Following coupling, the remaining coupling solution was discharged, and the resin washed with DMF (3 x 4 mL for every 100 mg resin). Following elongation to the desired peptide, if necessary, one further Fmoc deprotection was performed.

Peptide Purification Conditions

Peptides were purified using an automatic Gilson HPLC, consisting of a Gilson 322 pump, Gilson 159 UV/Vis detector, Gilson GX-271 liquid handler equipped with 5 mL sample loop and Gilson Verity 4020 syringe pump. The system was operated, and data processed on Trilution Liquid Chromatography software. HPLC Method: Flow rate = 10 mL min^{-1}, gradient starting from 20% MeCN and 80% water (0.1% TFA) for 6 min, ramping up to 45% MeCN over 13 min, ramping up to 55% MeCN over 23 min, ramping up to 95% MeCN over 1 min, remaining at 95% MeCN for 3 min, ramping down to 20% MeCN over 3 min and staying at 20% MeCN for 3 min. Analytical HPLC traces were obtained on a Perkin Elmer HPLC system composed of a 200 series binary pump, UV/Vis detector, vacuum degasser, Rheodyne 7725i injector equipped with 100 µL sample loop and a Phenomenex C18 Luna column (5 µm, 150 x 4.60 mm). Traces were obtained using the following method: flow rate = 2 mL min^{-1}, gradient starting from 5% acetonitrile and 95% water (0.1 % TFA) for 2 min, ramping up to 95% acetonitrile over 18 min, ramping back down to 5% acetonitrile for 0.1 min and remaining at 5% acetonitrile for 3.9 min.

General Microbiology Procedures

General MicrobiologyInformation

All microbiology procedures were carried out in the presence of a flame from a Bunsen burner and the bench disinfected with 70% ethanol solution to minimise cross-contamination. All waste was autoclaved (121 °C) before disposal. *Staphylococcus aureus* RN4220 was used in all assays. Mueller-Hinton broth (MHB) and agar were purchased from VWR chemicals. MHB was

prepared by dissolving the broth powder (21 g) in Milli-Q water (1 L) and autoclaved. Mueller-Hinton agar was prepared by dissolving the agar powder (38 g) in Milli-Q water (1 L) and autoclaving. While still hot, but cool enough to handle, the agar solution was poured into sterile culture dishes, left to cool to room temperature and kept at 5 °C until use. Inoculum were steaked onto the agar plates and incubated at 37 °C for 24 h to give single colonies. Streaked plates were kept at 5 °C until use. For MIC testing, single colonies were suspended in a 2 mL disposable, sterile cuvette filled with MHB broth and aspirated with a pipette to give an even dispersion. The OD_{600} value was determined to be 0.08 – 0.13 and the concentration adjusted if necessary to give the necessary OD_{600}. 200 µL of this solution was diluted with MHB (20 mL).

Determination of Minimum Inhibitory Concentrations

Minimum inhibitory concentration (MIC) values were determined by microbroth dilution assays according to Clinical & Laboratory Standards (CLSI) guidelines. Peptides were dissolved in 5% DMSO in Milli-Q water (1 mg mL^{-1}). 50 µL of these solutions were diluted with MHB (200 µL) to give a concentration of 200 µ mL^{-1}. To a 96-well plate, 100 µL of MHB broth was added wells in columns 1 – 12. 100 µL of the peptide solution was added to column 1 and serial diluted across wells in columns 1 – 10 using a Sartorius 200 µL Proline Plus multichannel pipette. 100 µL of the inoculum solution was added to wells in columns 1 – 10 and 12. Column 11 is a negative control to ensure no contamination of the MHB. Column 12 is a positive control to ensure sufficient inoculum growth and to act as a reference for full growth. The MIC testing was performed in triplicate for each peptide. The 96-well plate was incubated at 37 °C for 16 – 20 h. And MIC values obtained through visual inspection. The MIC was determined as the lowest concentration of peptide where no inoculum growth had occurred.

Microbroth Antagonisation Assays

Quinone was dissolved in DMSO (1 mL) to give a concentration of double that of the equivalent peptide solution. 400 μL of this solution was diluted by MHB (1.6 mL). 100 μL MHB was added to all wells of a 96-well plate. 100 μL of the quinone solution was added to columns 1 – 12 of row A. This was serial diluted down rows B – D to give the equivalencies of quinone to peptide 2.0, 1.0, 0.5, 0.25 and 0 in rows A – E respectfully. The MIC testing then set up as described above. After the peptide solution was added and diluted, the 96-well plate was left for 10 min before adding the inoculum solution to allow time for a binding equilibrium to be reached. Column 11 acts as the negative control to ensure no contamination of the quinone MHB solution and column 12 acts as a positive control and visual reference. Row E acts as a reference MIC assay without any quinone present. Each antagonisation MIC assay was performed in duplicate. Antagonisation, and hence binding, was determined to be if the MIC value was higher in the presence of quinone and if increasing the quinone concentration increased the MIC value.

General Procedure for Preparation of Large Unilamellar Vesicles (LUVs)

DOPG and DOPC solutions were mixed in a 1:1 molar ratio. Quinone solution ($CHCl_3$) was added to the lipid solution to give 1.25% molar ration (to total lipid concentration). The solvent was removed under a stream of argon. The resulting lipid film was then suspended in HEPES buffer (20 mM HEPES and 50 mM NaCl in Milli-Q water) to give a final lipid concentration of 5 mM. The lipid suspension was frozen in liquid nitrogen and thawed in a 40 °C water bath. This freeze-thaw cycle was completed a further 4 times. The lipids were filtered using an Avanti extruder through Whatman 0.1 μm polycarbonate filters 11 times. LUVs were used the same day.

Isothermal Titration Calorimetry (ITC)

All ITC binding experiments were performed on a MicroCal PEAQ-ITC Automated microcalorimeter (Malvern Panalytical Ltd). LUVs were prepared according to method B. Peptides were dissolved in HEPES buffer (20 mM HEPES and 50 mM NaCl in Milli-Q water). Samples were equilibrated to 25 °C prior to titration. All titrations were performed at 25 °C with stirring at 1000 rpm. Each titration started with an initial injection of 0.5 µL followed by 25 injections of 1.5 µL with a time delay of 180 s between each injection. The reference power cell was set to 5 µcal s^{-1} and the feedback mode set to 'low'. All data was analysed on MicroCal PEAQ-ITC Analysis Software Version 1.21. The relevant blank titrations and dilution data were compared to, but not subtracted from binding data. Data fitting was performed using the 'one set of sites' model. Best fit was defined by the lowest chi-squared value possible for that given binding experiment. Binding and thermodynamic parameters are reported as the mean of three experiments with standard deviation.

General Procedure for Preparation of Giant Unilamellar Vesicles (GUVs)

GUVs were prepared using electroformation in a Teflon chamber equipped with two titanium wires as electrodes. A prepared lipid solution (98% DOPC, 2% unlabelled quinone lipid, 0.1% fluorescently labelled quinone lipid, total lipid concentration = 0.5 mM in $CHCl_3$) (10 µL) was dripped on the two electrodes and dried *in vacuo* for 20 min. Sucrose solution (350 µL, 125 mM) was added to the chamber and the chamber closed, suspending the electrodes in the solution. The electrodes were attached to a power source and an oscillating sine wave voltage (2.5 V, 10.0 Hz) applied for 1.5 h. GUV were then displaced from the electrodes by applying a square wave voltage (2.0 V, 2.0 Hz) for 15 min. The microscopy slide (µ-slide 8 well) was preincubated with bovine serum albumin (350 µL, 5% w/v, Aq.) for 20 min, followed by washing

with micellar water 5 times and drying under nitrogen. Each slide was filled with glucose solution (250 µL, 125 mM) and GUV suspension (100 µL).

Microscopy

Microscopy was performed Zeiss LSM 880 confocal microscope with a ×100/1.4NA oil objective lens. BODIPY and NBD fluorescence (496 – 669 nm) was detected after excitation with a 488 nm laser. Images were processed using FIJI ImageJ2 v.2.15.1.

Synthetic Chemistry Procedures and Compound Characterization

Me_2-CoQ10 (4)

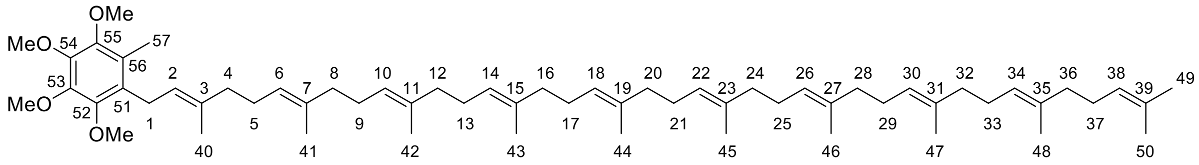

Me_2-CoQ10 (**4**) was prepared according to literature.[1] CoQ10 (**1**) (5.0 g, 5.79 mmol) was dissolved in acetone (200 mL) and $NaBH_4$ (1.01 g, 29.0 mmol) in MeOH (20 mL) was added, the solution turned from a bright orange to pale yellow. The reaction mixture was stirred for 15 min at ambient temperature under argon. NaOH (579 mg, 14.5 mmol) in H_2O (3 mL) and dimethyl sulfate (2.74 mL, 29.0 mol) were added dropwise simultaneously. The reaction mixture was stirred for one h then heated to reflux (56 °C) and stirred for 20 h. Reaction completion was confirmed through TLC. The reaction mixture was quenched with HCl (1M, 200 mL) and the mixture concentrated under reduced pressure. The quenched reaction mixture was extracted with EtOAc (3 x 200 mL), the organic extracts were dried over Na_2SO_4 and solvent removed under reduced pressure. Flash chromatography (2:98 EtOAc/hexane) afforded Me_2-

CoQ10 (**4**) as a viscous clear oil (4.59 g, 5.14 mmol, 89%). NMR data matched that of literature.[1]

^{1}H NMR (400 MHz; $CDCl_3$): δ 5.13 – 5.07 (10H, m, H6, H10, H14, H18, H22, H26, H30, H34, H38), 5.06 – 5.03 (1H, m, H2) 3.91 (3H, s, MeO), 3.90 (3H, s, MeO), 3.783 (3H, s, MeO), 3.781 (3H, s, MeO), 3.32 (2H, d, *J* = 6.5 Hz, H1), 2.14 (3H, s, H57), 2.10 – 1.93 (32H, m, H4, H5, H8, H9, H12, H13, H16, H17, H20, H21, H24, H25, H28, H29, H32, H33, H36, H37), 1.77 (3H, s, H40), 1.68 (3H, s, H49), 1.60 – 1.56 (27H, m, H41, H42, H43, H44, H45, H45, H46, H47, H48) ppm.

^{13}C-NMR (101 MHz, $CDCl_3$): δ 169.1, 169.0, 143.6, 143.4, 140.9, 140.6, 136.0, 135.3, 135.11, 135.09, 135.07, 135.05, 135.0, 131.4, 128.6, 125.1, 124.6, 124.43, 124.40, 124.37, 124.1, 121.4, 60.84, 60.82, 39.90, 39.88, 39.8, 26.93, 26.90, 26.88, 26.83, 26.78, 26.4, 25.7, 20.64, 20.61, 17.8, 16.4, 16.20, 16.18, 16.16, 12.3 ppm.

HRMS–ESI (*m/z*): $[M+H]^+$ calculated 893.7381; found 893.7369.

Boc_2-CoQ10 (5)

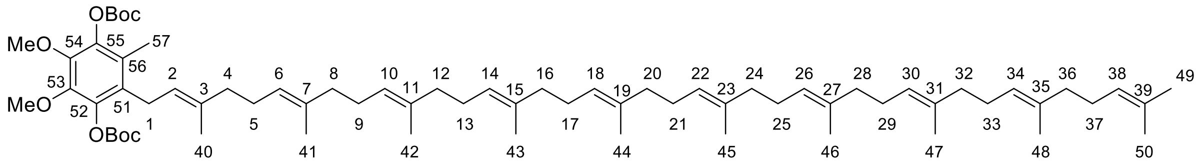

Boc_2-CoQ10 (**5**) was prepared according to a modified literature procedure.[2] CoQ10 (**1**) (10.00 g, 11.58 mmol) was dissolved in Et_2O (100 mL) and added into a separating funnel with H_2O (100 mL) and $Na_2S_2O_4$ (16.83 g, 492.66 mmol). The separating funnel was shaken for 10 min until the organic layer turned orange to colourless. The aqueous layer was separated and the

organic layer washed with brine (100 mL). The organic layer was dried over Na_2SO_4 and the solvent removed under reduced pressure. The resulting pale yellow oil was dissolved in dry THF (50 mL) and *tert*-butyloxycarbonyl anhydride (5.30g, 24.32 mmol) and DMAP (141 mg, 1.16 mmol) was added. The reaction was stirred at ambient temperature for 18 h at which point TLC indicated reaction completion. The solvent was removed *in vacuo* and the crude resuspended in EtOAc (150 mL), the organic layer was washed with HCl (1 M, 100 mL) and brine (100 mL), dried over Na_2SO_4 and solvent removed under reduced pressure. This afforded Boc$_2$-CoQ10 (**5**) (11.0 g, 10.33 mmol, 89%) as a pale-yellow oil. No further purification was necessary.

^{1}H NMR (400 MHz; $CDCl_3$): δ 5.13 – 5.05 (9H, m, H6, H10, H14, H18, H22, H26, H30, H34, H38), 5.05 – 4.99 (1H, m, H2), 3.865 (3H, s, MeO), 3.862 (3H, s, MeO), 3.26 (2H, d, J = 6.6 Hz, H1), 2.08 – 1.88 (39H, m, H4, H5, H8, H9, H12, H13, H16, H17, H20, H21, H24, H25, H28, H29, H32, H33, H36, H37, H57), 1.73 (3H, s, 40), 1.69 (3H, s, H49), 1.62–1.57 (27H, m, H41, H42, H43, H44, H45, H46, H47, H48, H50), 1.56 (9H, s, *t*Bu), 1.54 (9H, s, *t*Bu) ppm.

^{13}C NMR (101 MHz, $CDCl_3$): δ 151.5, 151.4, 143.6, 143.4, 140.9, 140.6, 135.7, 135.0, 134.9, 134.83, 134.80, 131.2, 128.3, 124.8, 124.4, 124.21, 124.18, 124.0, 121.1, 83.2, 83.0, 60.7, 60.63, 60.60, 39.69, 39.67, 39.6, 27.63, 27.59, 27.58, 26.72, 26.67, 26.6, 26.0, 25.6, 17.6, 16.2, 16.0, 11.9 ppm.

*Due to the close equivalence of multiple carbons, several signals overlap.

HRMS–ESI (*m/z*): $[M+NH_4]^+$ calculated 1082.8382; found 1082.8508.

IR (neat, cm^{-1}): 2976, 2917, 2850, 1757, 1448, 1358, 1277, 1234, 1144, 1107, 1083. 855, 779.

Rf = 0.32 (10% EtOAc in hexane).

General Procedure for Synthesis of Boc$_2$-MKs (6 – 7)

MK (**2** or **3**) was dissolved in dry THF (0.13 M) and $NaBH_4$ (2 equiv.) in dry MeOH (2.5 M) added to the reaction mixture. The reaction mixture was left to stir under argon at room temperature for 10 min until the reaction mixture turned yellow to colourless. *tert*-butyloxycarbonyl anhydride (Boc_2O) (2.5 equiv.) was added in 5 portions over 1 h, followed by DMAP (0.1 equiv.). The reaction mixture was left to stir for 3 h. Further $NaBH_4$ (1 equiv.) and Boc_2O (2 equiv.) was added, and the reaction mixture was left to stir at room temperature for a further 18 h. The reaction mixture was concentrated *in vacuo* and redissolved in EtOAc, washed with HCl (1M, Aq.) and brine. The organic layer was dried over Na_2SO_4 and the solvent removed under reduced pressure. Flash chromatography afforded Boc$_2$-MK derivatives (**6** or **7**).

Boc$_2$-MK4 (6)

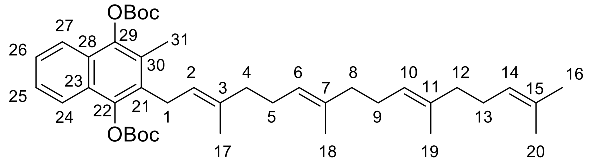

Flash chromatography (0:100 – 5:95 EtOAc/hexane) afforded Boc$_2$-MK4 (**6**) as a pale yellow oil (810 mg, 01.25 mmol, 93%).

^{1}H-NMR (400 MHz, $CDCl_3$): δ 7.83 – 7.76 (2 H, m, H24, H27), 7.49 – 7.46 (2H, m, H25, H26), 5.11 – 5.06 (4H, m, H2, H6, H10, H14), 3.47 (2H, d, J = 6.1 Hz, H1), 2.29 (3H, s, H31), 2.07 – 1.93 (12H, m, H4, H5, H8, H9, H12, H13), 1.79 (3H, d, J = 1.1 Hz, H17), 1.67 (3H, d, J = 1.1 Hz, H19), 1.59 – 1.56 (27 H, m, H18, H19, H20, 2 x *t*Bu) ppm.

^{13}C-NMR (101 MHz, $CDCl_3$):δ 152.1, 151.7, 146.9, 142.7, 142.4, 136.4, 135.3, 135.0, 131.4, 130.7, 127.3, 126.7, 126.6, 126.5, 126.4, 124.6, 124.4, 124.2, 121.3, 121.20, 121.17, 85.3, 83.64, 83.63, 39.9, 39.84, 39.81, 27.9, 27.8, 27.6, 26.9, 26.83, 26.79, 25.8, 17.8, 16.6, 16.1, 12.9 ppm.

HRMS–ESI (*m/z*): $[M+NH_4]^+$ calculated 664.4571; found 664.4590.

IR (neat, cm^{-1}): 2976, 2920, 1757, 1552, 1370, 1241, 1139, 1068, 744.

Rf : 0.73 (20% EtOAc in hexane)

Boc_2-MK-9 (7)

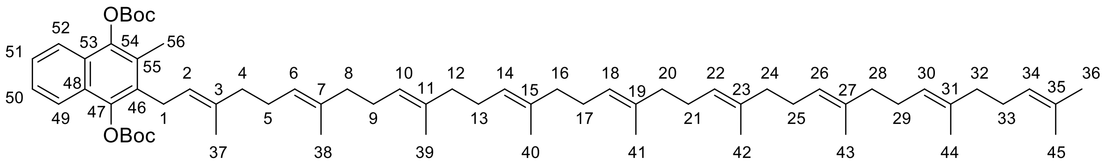

Flash chromatography (0:100 – 5:95 EtOAc/hexane) afforded Boc_2-MK9 (**7**) as a yellow crystalline solid (973 mg, 0.986 mmol, 78%). This was deemed to be of sufficient purity to take on to the next step.

^{1}H-NMR (400 MHz; $CDCl_3$): δ 7.83 – 7.77 (2H, m, H49, H52), 7.49 – 7.47 (2H, m, H50, H51), 5.13 – 5.08 (9H, m, H2, H10, H14, H18, H22, H26, H30, H34), 3.48 (2H, d, *J* = 6.2 Hz, H1, 2.29 (s, 3H, H56), 2.06 – 1.97 (32H, m, H4, H5, H8, H9, H12, H13, H16, H17, H20, H21, H24, H25, H28, H29, H32, H33) 1.79 (3H, d, *J* = 1.1 Hz, H37), 1.68 (3H, d, *J* = 1.1 Hz, H36), 1.60 – 1.55 (24H, m, H38, H39, H40, H41, H42, H43, H44, H45), 1.55 (9H, s, *t*Bu), 1.53 (9H, s, *t*Bu) ppm.

^{13}C-NMR (101 MHz, $CDCl_3$): δ 152.1, 151.7, 146.9, 142.8, 142.4, 136.4, 135.3, 135.07, 135.06, 135.0, 131.4, 130.7, 127.3, 126.7, 126.6, 126.5, 126.4, 124.6, 124.42, 124.36, 124.2, 121.3,

121.21, 121.18, 85.3, 83.6, 39.90, 39.88, 39.8, 31.8, 27.9, 27.83, 27.79, 27.6, 27.5, 26.93, 26.90, 26.86, 26.8, 25.9, 22.8, 17.8, 16.6, 16.18, 16.16, 14.3, 12.9 ppm.

*Due to the close equivalence of multiple carbons, several signals overlap.

HRMS–ESI (*m/z*): $[M+H]^+$ calculated 987.7436 ; found 987.7437.

IR (neat, cm^{-1}): 2960, 2917, 2851, 1761, 1446, 1370, 1241, 1140, 1101.

Rf : 0.46 (10% Et_2O in hexane)

Me_2-CoQ10-ω-epoxide (8)

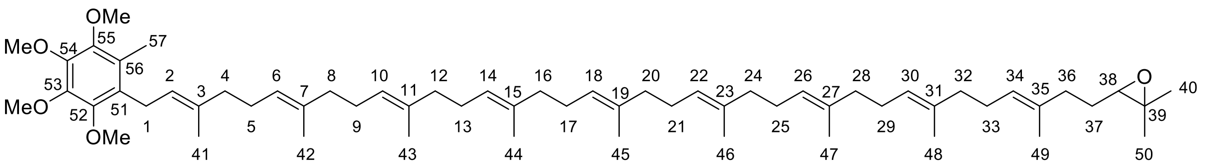

Me_2-CoQ10-ω-epoxide (**8**) was prepared according to a modified literature procedure.[3] Me_2-CoQ10 (**4**) (4.59 g, 5.14 mmol) was dissolved in THF (90 mL) and cooled to 0 °C. H_2O (10 mL) was added until the solution became cloudy. N-bromosuccinimide (1.00 g, 5.65 mmol) was added in 3 portions over 30 min. The reaction was stirred at 0 °C for 5 h. The reaction was concentrated *in vacuo* and diluted with H_2O (100 mL) and extracted with EtOAc (3 x 100 mL). The organic extracts were dried over Na_2SO_4 and the solvent removed under reduced pressure. The resulting oil was dissolved in THF:MeOH (3:2, 50 mL) and K_2CO_3 (2.13 g, 15.4 mmol) was added, the reaction was stirred at atmospheric temperature for 18 h. The reaction mixture was concentrated under reduced pressure, quenched with brine:H_2O (2:1, 100 mL) and extracted with EtOAc (3 x 100 mL). The organic extracts were dried over Na_2SO_4 and solvent removed under reduced pressure. Flash chromatography (2:98 to 7:93 EtOAc/hexane) afforded Me_2-CoQ10-ω-epoxide (**8**) as a viscous clear oil (1.23 g, 1.35 mmol, 26%).

^{1}H-NMR (600 MHz, $CDCl_3$): δ 5.17 – 5.15 (1H, m, H34), 5.12 – 5.07 (7H, m, H6, H10, H14, H18, H22, H26, H30), 5.05 – 5.03 (1H, m, H2), 3.91 (3H, s, MeO), 3.90 (3H, s, MeO), 3.783 (3H, s, MeO), 3.781 (3H, s, MeO), 3.32 (2H, d, J = 6.3 Hz, H1), 2.70 (1H, t, J = 6.3 Hz, H38), 2.14 (3H, s, H57), 2.10 – 1.94 (33H, m, H4, H5, H8, H9, H12, H13, H16, H17, H20, H21, H24, H25, H28, H29, H32, H33, H36), 1.77 (3H, s, H41), 1.69 – 1.63 (2H, m, H37), 1.62 – 1.58 (24H, m, H42, H43, H44, H45, H46, H47, H48, H49), 1.30 (s, 3H), 1.26 (s, 3H) ppm.

^{13}C-NMR (151 MHz, $CDCl_3$): δ 148.0, 147.8, 145.1, 144.8, 135.3, 135.2, 135.10, 135.08, 135.05, 134.9, 134.1, 129.4, 125.5, 125.1, 124.5, 124.42, 124.39, 124.34, 124.26, 121.0, 64.4, 61.3, 61.24, 61.22, 60.8, 58.5, 39.90, 39.88, 39.8, 36.5, 27.6, 26.88, 26.87, 26.84, 26.83, 26.79, 25.9, 25.1, 18.9, 16.4, 16.19, 16.17, 16.16, 11.9 ppm.

*Due to the close equivalence of multiple carbons, several signals overlap.

HRMS–ESI (*m/z*): $[M+Na]^+$ calculated 931.715018; found 931.7129.

IR (neat, cm^{-1}): 2958, 2920, 2850, 1465, 1446, 1407, 1351, 1258, 1195, 1103, 1068, 1040, 1012, 978, 870, 732.

Rf = 0.81 (10% EtOAc in hexane).

General Procedure for Synthesis of Boc_2-PPQ-ω-epoxides (9 – 11)

Boc_2-PPQ-ω-epoxides (**9** – **11**) were synthesized according to a previously reported procedure.[3] Boc_2-PPQ (**5**, **6**, or **7**) was dissolved in THF (0.12 M) and cooled to 0 °C. H_2O was added until the solution became cloudy. N-bromosuccinimide (1.1 equiv.) was added and the reaction mixture stirred for 5 h at 0 °C. The reaction mixture was warmed to room temperature and the THF removed under reduced pressure. The reaction mixture was diluted with H_2O and extracted with EtOAc thrice. The combine organic extracts were dried over Na_2SO_4 and the solvent removed under reduced pressure. The crude solid was then dissolved in THF:MeOH (3:2) and K_2CO_3 (2 equiv.) added. The reaction mixture was stirred at room temperature

overnight. The solvent was removed *in vacuo* and reaction mixture quenched with H_2O. This was extracted with EtOAc thrice, the combined organic layers dried over Na_2SO_4 and solvent removed *in vacuo*. Flash chromatography afforded Boc$_2$-PPQ-ω-epoxides (**9** – **11**).

Boc$_2$-CoQ10-ω-epoxide (9)

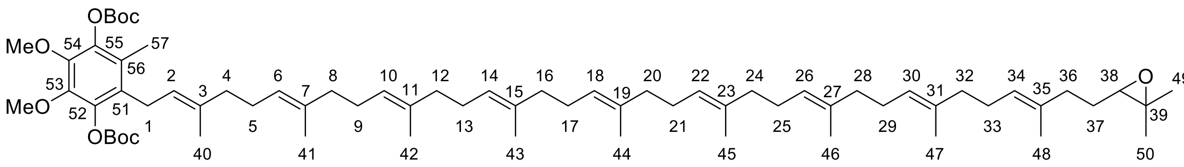

Flash chromatography (2:98 to 10:90 EtOAc/hexane) afforded Boc$_2$-CoQ10-ω-epoxide (**9**) as a viscous clear oil (1.45 g, 1.34 mmol, 29%).

^{1}H NMR (400 MHz; $CDCl_3$): δ 5.17 – 5.04 (8H, m, H6, H10, H14, H18, H22, H26, H30, H34), 5.02 – 4.98 (1H, m, H2) 3.852 (3H, s, MeO), 3.850 (3H, s, MeO), 3.25 (2H, d, J = 6.4 Hz, H1), 2.69 (1H, t, J = 6.0 Hz, H38), 2.08 – 1.96 (37H, m, H4, H5, H8, H9, H12, H13, H16, H17, H20, H21, H24, H25, H28, H29, H32, H33, H36, H57), 1.74 (3H, d, J = 1.1 Hz, H40), 1.70 – 1.64 (2H, m, H37) 1.62 – 1.56 (24H, m, H41, H42, H43, H44, H45, H46, H47, H48) 1.55 (9 H, s, *t*Bu) 1.53 (9 H, s, *t*Bu) 1.29 (3H, s, H49/50) 1.25 (3H, s, H49/50) ppm.

^{13}C NMR (101 MHz; $CDCl_3$): δ 151.6, 143.7, 140.7, 135.6, 135.1, 135.0, 134.0, 128.4, 125.0, 124.3, 83.3, 83.1, 64.2, 60.8, 58.3, 39.79, 39.77, 36.4, 27.69, 27.67, 24.9, 18.8, 16.06, 16.05, 12.0 ppm.

*Due to the close equivalence of multiple carbons, several signals overlap.

HRMS–ESI (*m/z*): $[M+NH_4]^+$ calculated 1098.8331; found 1098.8342.

IR (neat, cm^{-1}): 2976, 2917, 2851, 1757, 1448, 1368, 1277, 1232, 1144, 855.

Rf = 0.70 (30:70 EtOAc:hexane).

Boc$_2$-MK4-ω-epoxide (10)

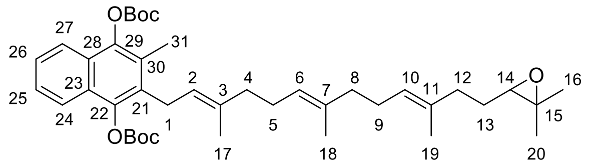

Flash chromatography (2:98 – 10:90 EtOAc/hexane) afforded Boc$_2$-MK4-ω-epoxide (**10**) as a colourless oil (62 mg, 0.094 mmol, 35%).

^{1}H-NMR (400 MHz, $CDCl_3$): δ 7.84 – 7.75 (2H, m, H24, H27), 7.50 – 7.46 (2H, m, H25, H26), 5.14 – 5.05 (3H, m, H2, H6, H10), 3.47 (2H, d, *J* = 5.9 Hz, H1), 2.69 (1H, t, *J* = 6.3 Hz, H14), 2.29 (3H, s, H31), 2.09 – 1.93 (10H, m, H4, H5, H8, H9, H12), 1.79 (3H, s, H17), 1.65 – 1.61 (2H, m, H13), 1.60 – 1.57 (24H, m, H18, H19, 2 x *t*Bu), 1.28 (3H, s, H16), 1.24 (3, s, H20) ppm.

^{13}C-NMR (101 MHz, $CDCl_3$): δ 152.1, 151.7, 142.7, 142.4, 136.3, 135.1, 134.1, 130.7, 127.3, 126.7, 126.6, 126.5, 126.4, 125.0, 124.3, 121.3, 121.23, 121.17, 83.64, 83.63, 64.4, 58.5, 39.80, 39.75, 36.4, 29.9, 27.9, 27.8, 27.6, 26.9, 26.82, 26.77, 25.0, 18.9, 16.6, 16.1, 12.9 ppm

*Due to the close equivalence of multiple carbons, several signals overlap.

HRMS–ESI (m/z): $[M+H]^+$ calculated 663.4256; found 663.4197.

IR (neat, cm^{-1}): 2976, 2920, 2850, 1757, 1370, 1239, 1138, 1100, 885, 800, 744.

Rf : 0.50 (20% EtOAc in hexane)

Boc_2-MK9-ω-epoxide (11)

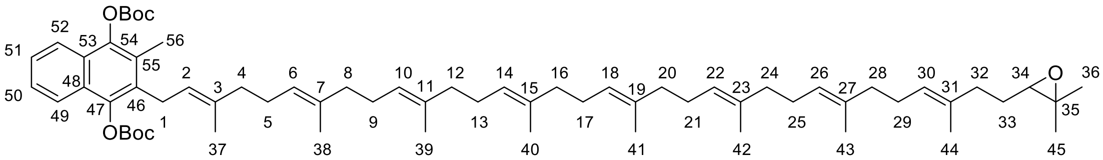

Flash chromatography (2:98 – 6:94 EtOAc/hexane) afforded Boc_2-MK9-ω-epoxide (**11**) as a colourless oil (264 mg, 0.26 mmol, 29%).

^{1}H-NMR (400 MHz, $CDCl_3$): δ 7.84 – 7.76 (2H, m, H49, H52), 7.50 – 7.46 (2H, m, H50, H51), 5.17 – 5.07 (8H, m, H2, H6, H10, H14, H18, H22, H26, H30), 3.47 (2H, d, *J* = 5.9 Hz, H1), 2.70 1H, (t, *J* = 6.3 Hz, H34), 2.29 (3H, s, H56), 2.09 – 1.97 (30 H, m,H4, H5, H8, H9, H12, H13, H16, H17, H24, H25, H28, H29, H32), 1.79 (3H, d, *J* = 1.0 Hz, H37), 1.69 – 1.65 (2H, m, H33), 1.62 – 1.56 (39 H, m, H38, H39, H40, H41, H42, H43, H44, H, 2 x *t*Bu), 1.30 (3H, s, H36), 1.26 (3H, s, H45) ppm.

^{13}C NMR (101 MHz; $CDCl_3$): δ 152.1, 151.7, 142.8, 142.4, 136.3, 135.3, 135.1, 135.0, 134.9, 134.1, 130.7, 127.3, 126.7, 126.6, 126.5, 126.4, 125.1, 124.6, 124.5, 124.41, 124.36, 124.2, 121.3, 121.21, 121.17, 83.60, 83.59, 64.3, 58.4, 39.90, 39.88, 39.8, 36.5, 27.9, 27.8, 27.6, 26.93, 26.90, 26.88, 26.86, 26.8, 25.1, 18.9, 16.6, 16.18, 16.16, 12.9 ppm.

*Due to the close equivalence of multiple carbons, several signals overlap.

HRMS–ESI (*m/z*): $[M+NH_4]^+$ calculated 1020.7651; found 1020.7604.

IR (neat, cm^{-1}): 2961, 2920, 2851, 1759, 1370, 1101, 885, 779, 744.

Rf : 0.83 (20% EtOAc in hexane)

Me$_2$-CoQ10-ω-2-AB (12)

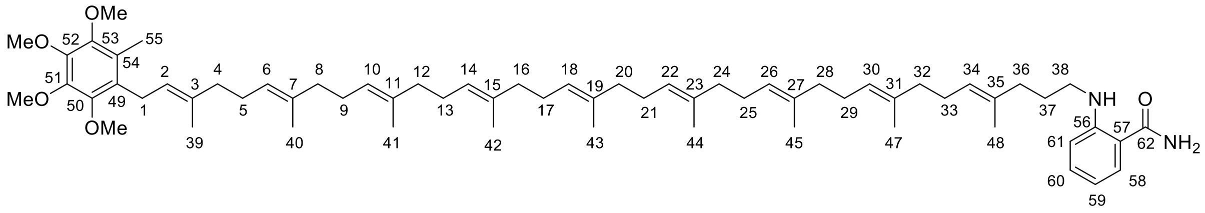

Me$_2$-CoQ10-ω-2-AB (**12**) was synthesized according to a previously reported procedure.[3] Me$_2$-CoQ10-ω-epoxide (**8**) (1.23 g, 1.35 mmol) was dissolved in THF:H_2O (9:1, 40 mL) and HIO_4 (369 mg) was added. The reaction was stirred at atmospheric temperature for 2 h at which point TLC confirmed reaction completion. The reaction was concentrated under reduced pressure, diluted with brine:H_2O (1:1, 50 mL) and extracted with EtOAc (3 x 50 mL). The organic extracts were dried over Na_2SO_4 and solvent removed *in vacuo* to afford crude ω-aldehyde (1.02 g, 1.18 mmol, 88%) as a clear viscous oil. A portion of ω-aldehyde (481 mg, 0.56 mmol) was dissolved in DCE (5 mL) and anthranilamide (2-AB) (93 mg, 0.67 mmol) added, followed by AcOH (57 µL in 0.5 mL DCE). This was stirred under argon at ambient temperature for 5 min. $NaBH(OAc)_3$ (237 mg, 1.11 mmol) was added and the reaction mixture stirred at ambient temperature for 18 h, at which point, TLC confirmed reaction completion. The reaction mixture was quenched with $NaHCO_3$ (10 mL) and extracted with Et_2O (2 x 10 mL). The organic layers were dried over Na_2SO_4 and solvent removed under reduced pressure. Flash chromatography (20:80 EtOAc/hexane + 0.1% TEA) afforded Me$_2$-CoQ10-ω-2-AB (**12**) as a pale yellow viscous oil (219 mg, 0.22 mmol, 40%).

^{1}H-NMR (400 MHz, $CDCl_3$): δ 7.37 (1H, dd, *J* = 7.9, 1.5 Hz, H58), 7.31 (1H, ddd, *J* = 8.5, 7.1, 1.5 Hz, H60), 6.69 (1H, d, *J* = 8.5 Hz, H61), 6.57 – 6.53 (1H, m, H59), 5.18 – 5.04 (9H, m, H2, H6, H10, H14, H18, H22, H26, H30, H34), 3.91 (3H, s, MeO), 3.90 (3H, s, MeO), 3.783 (3H, s,

MeO), 3.781 (3H, s, MeO), 3.32 (2H, d, *J* = 6.4 Hz, H1), 3.12 (2H, t, *J* = 7.2 Hz, H38), 2.14 (3H, s, H55), 2.09 – 1.95 (34H, m,H4, H5, H8, H9, H12, H13, H16, H17, H20, H21, H24, H25, H28, H29, H32, H33, H36), 1.78 – 1.74 (5H, m, H37, H39), 1.61 – 1.58 (24H, m,H40, H41, H42, H43, H44, H45, H46, H47, H48) ppm.

^{13}C-NMR (101 MHz, $CDCl_3$): δ 172.2, 150.5, 148.0, 147.8, 145.1, 144.8, 135.3, 135.2, 135.1, 135.0, 134.1, 133.7, 129.4, 128.4, 125.5, 125.1, 124.5, 124.43, 124.38, 124.34, 124.25, 123.0, 77.0, 61.3, 61.2, 60.8, 42.6, 39.90, 39.88, 39.85, 39.83, 39.81, 37.2, 27.4, 26.88, 26.85, 26.84, 26.79, 26.76, 25.9, 16.4, 16.19, 16.18, 16.0, 11.9 ppm.

*Due to the close equivalence of multiple carbons, several signals overlap.

HRMS–ESI (*m/z*): $[M+H]^+$ calculated 987.7549 found 987.7217.

IR (neat, cm^{-1}): 2922, 2848, 1655, 1614, 1580, 1521, 1465, 1405, 1282, 1197, 1103, 1066, 1040, 910, 842, 801, 731.

Rf = 0.80 (50% EtOAc in hexane).

Boc$_2$-CoQ10-ω-2-AB (13)

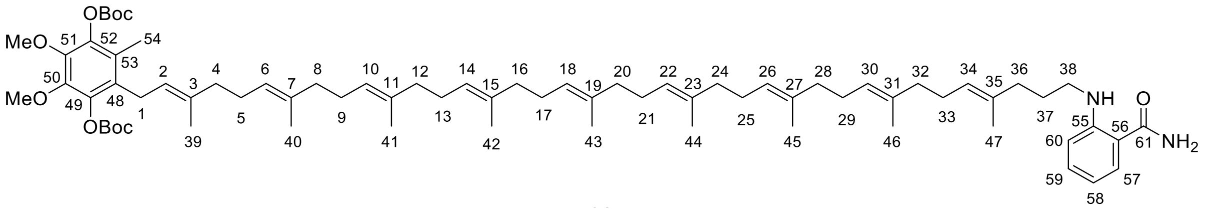

Boc$_2$-CoQ10-ω-2-AB (**13**) was synthesized according to a previously reported procedure.[3] Boc$_2$-CoQ10-ω-epoxide (**9**) (1.06 g, 0.981 mmol) was dissolved in THF:H_2O (9:1, 10 mL) and HIO_4 (268 mg) was added. The reaction was stirred at atmospheric temperature for 2 h at which point TLC confirmed reaction completion. The reaction was concentrated *in vacuo.*, diluted with

brine:H_2O (1:1, 20 mL) and extracted with EtOAc (3 x 20 mL). The organic extracts were dried over Na_2SO_4 and solvent removed *in vacuo* to afford Boc$_2$-CoQ10-ω-aldehyde (799 mg, 0.769 mmol, 78%) as a clear viscous oil. This material was carried forward without further purification. A portion of ω-aldehyde (110 mg, 0.107 mmol) was dissolved in DCE (1.5 mL) and anthranilamide (2-AB) (18 mg, 0.67 mmol) added, followed by AcOH (11 µL in 0.5 mL DCE) and stirred at ambient temperature under argon for 5 min. NaBH(OAc)$_3$ (45 mg, 0.214 mmol) was added and the reaction mixture stirred at ambient temperature under argon for 18 h, at which point TLC confirmed reaction completion. The solvent was removed *in vacuo,* quenched with $NaHCO_3$ (*sat*., 2 mL) and extracted with EtOAc (3 x 2 mL). The organic extracts were dried over Na_2SO_4 and solvent removed under reduced pressure. Flash chromatography (10:90 to 20:80 EtOAc/hexane) afforded Boc$_2$-CoQ10-ω-2-AB (**13**) as a viscous clear oil (34 mg, 0.029 mmol, 27%).

^{1}H NMR (400 MHz; $CDCl_3$): δ 7.85 – 7.78 (1H, m, NH), 7.36 (1H, dd, *J* = 8, 1.6 Hz, H57), 7.30 (1H, ddd, *J* = 8.5, 7.1, 1.5, H59), 6.67 (1H, dd, *J* = 8.5, 0.6 Hz, H60), 6,54 (1H, ddd, *J* = 7.9, 7.1, 1.0 Hz, H58) 5.17 – 5.05 (8H, m, H6, H10, H14, H18, H22, H26, H30, H34), 5.03 – 4.99 (1H, m, H2), 3.863 (3H, s, MeO), 3.861 (3H, s, MeO), 3.26 (2H, d, *J* = 6.4 Hz, H1), 3.12 (2H, q, *J* = 6.2 Hz, H38), 2.11 – 1.95 (37H, m, H4, H5, H8, H9, H12, H13, H16, H17, H20, H21, H24, H25, H28, H29, H32, H33, H36, H55), 1.80 – 1.73 (5H, m, H37, H39), 1.63 – 1.58 (24H, m, H40, H41, H42, H43, H44, H45, H46, H47), 1.56 (9 H, s, *t*Bu) 1.54 (9 H, s, *t*Bu) ppm.

^{13}C NMR (101 MHz; $CDCl_3$): δ 172.1, 151.6, 151.5, 150.5, 143.7, 143.5, 141.0, 140.7, 135.8, 135.1, 134.98, 134.97, 134.96, 134.9, 134.1, 133.5, 128.4, 128.3, 125.0, 124.9, 124.4, 124.3, 124.1, 121.2, 114.1, 112.6, 111.8, 83.3, 83.2, 60.8, 42.5, 39.80, 39.78, 39.7, 37.1, 27.69, 27.68, 27.3, 26.8, 16.1, 12.0 ppm.

*Due to the close equivalence of multiple carbons, several signals overlap.

HRMS–ESI (*m/z*): $[M+H]^+$ calculated 1159.8284; found 1159.8230.

IR (neat, cm^{-1}): 2976, 2917, 2850, 1757, 1657, 1579, 1519, 1478, 1452, 1368, 1277, 1234, 1142, 1107, 1083, 1049, 854, 744.

Rf = 0.85 (50:50 EtOAc:hexane).

General Procedure for Synthesis of Boc$_2$-MK-ω-2-AB Analogues (14 or **15)**

Boc$_2$-MK-ω-2-AB analogues (**14** or **15**) were prepared according to a modified literature procedures.[3,4] Boc$_2$-MK-ω-epoxides (**10** or **11**) were dissolved in THF:H_2O (9:1, 0.1 M) and HIO_4 (1.2 equiv.) added. The reaction mixture was stirred at room temperature for 1.5 h, at which point TLC indicated reaction completion. The solvent was removed *in vacuo* and the reaction mixture quenched with brine:H_2O (1:1). This was extracted with Et_2O three times, the combined organic extracts dried over Na_2SO_4, and solvent removed *in vacuo* to afford crude ω-aldehyde. The crude aldehyde was then dissolved in dry CH_2Cl_2 (0.1 M) with anthranilamide (2 equiv.) and AcOH (1.8 equiv.). This was stirred at ambient temperature for 15 min. $NaBH(OAc)_3$ (2 equiv.) was then added and the mixture stirred at ambient temperature for 3h. The reaction mixture was diluted with CH_2Cl_2, washed with $NaHCO_3$ (sat., Aq), brine and dried over Na_2SO_4. Flash column chromatography afforded Boc$_2$-MK-ω-2-AB analogues (**14** or **15**).

Boc$_2$-MK4-ω-2-AB (14)

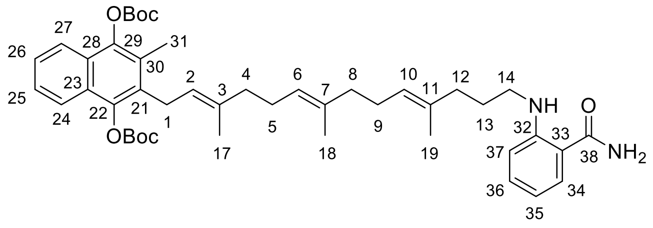

Flash column chromatography (20:80 – 40:60) afforded Boc$_2$-MK4-ω-2-AB (**14**) as a viscous colourless oil (10.6 mg, 0.0143 mmol, 13%).

^{1}H-NMR (400 MHz, $CDCl_3$): δ 7.82 – 7.78 (2H, m, H24, H27), 7.49 – 7.47 (2H, m, H25, H26), 7.32 (1H, dd, *J* = 7.9, 1.5 Hz, H34), 7.30 – 7.27 (1H, m, H36), 6.66 (1H, dd, *J* = 8.5, 0.8 Hz, H37), 6.50 (1H, ddd, *J* = 7.9, 7.1, 1.1 Hz, H35), 5.14 – 5.04 (3H, m, H2, H6, H10), 3.46 (2H, d, *J* = 6.1 Hz, H1), 3.10 (2H, td, *J* = 7.0, 5.5 Hz, H14), 2.29 (3H, s, H31), 2.09 – 1.93 (10H, m, H4, H5, H8, H9, H12), 1.78 (3H, d, *J* = 1.1 Hz, H17), 1.76 – 1.70 (2H, m, H13), 1.59 – 1.55 (24H, m, H18, H19, 2 x *t*Bu) ppm.

^{13}C-NMR (151 MHz, $CDCl_3$): δ 172.2, 152.1, 151.7, 150.6, 142.7, 142.4, 136.4, 135.0, 134.1, 133.6, 130.7, 128.5, 127.3, 126.64, 126.55, 126.4, 125.0, 124.3, 121.3, 121.2, 121.1, 114.1, 112.7, 111.8, 83.68, 83.66, 42.4, 39.77, 39.75, 37.2, 29.8, 27.9, 27.8, 27.3, 26.93, 26.86, 26.6, 16.5, 16.1, 16.0, 12.9 ppm.

HRMS–ESI (*m/z*): $[M+H]^+$ calculated 741.4473; found 741.4571.

IR (neat, cm^{-1}): 3355, 2980. 2928, 2851, 1757, 1653, 1579, 1612, 1519, 1454, 1370, 1277, 1239, 1137, 1101. 1075, 908, 883, 779, 729.

Rf : 0.54 (50% EtOAc in hexane)

Boc₂-MK9-ω-2-AB (15)

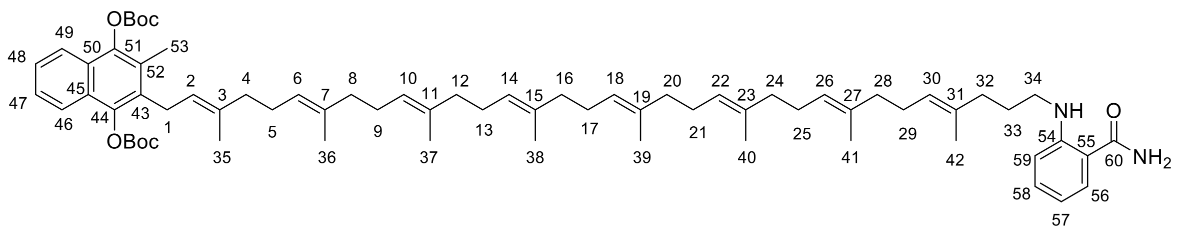

Flash chromatography (20:80 – 40:60 EtOAc:hexane) afforded Boc$_2$-MK9-ω-2-AB (**115**) as a viscous colourless oil (9.1 mg, 0.008 mmol, 18%).

^{1}H-NMR (400 MHz, $CDCl_3$): δ 7.82 – 7.76 (2H, m, H46, H49), 7.49 – 7.46 (2H, m, H47, H48), 7.35 (1H, dd, *J* = 7.9, 1.5 Hz, H56), 7.32 – 7.28 (1H, m, H58), 6.69 – 6.66 (1H, m, H59), 6.54 (1H, ddd, *J* = 8.0, 7.1, 1.0 Hz, H57), 5.17 – 5.06 (8H, m, H2, H6, H10, H14, H18, H22, H26, H30), 3.47 (2H, d, *J* = 5.7 Hz, H1), 3.16 – 3.12 (2H, m, H34), 2.29 (3H, s, H53), 2.09 – 1.94 (30H, m, H4, H5, H8, H9, H12, H13, H16, H17, H20, H21, H24, H25, H28, H29, H32), 1.79 (3H, d, *J* = 1.0 Hz, H35), 1.77 – 1.74 (2H, m, H33), 1.61 – 1.55 (39H, m, H36, H37, H38, H39, H40, H41, H42, 2 x *t*Bu) ppm.

^{13}C-NMR (101 MHz, $CDCl_3$): δ 152.1, 150.7, 144.3, 142.8, 136.4, 135.3, 135.1, 135.0, 134.2, 133.7, 130.7, 128.5, 128.4, 127.3, 126.7, 126.6, 126.5, 126.4, 125.1, 121.3, 121.2, 114.2, 112.7, 111.9, 83.6, 77.4, 42.6, 39.9, 39.8, 39.7, 37.2, 29.9, 29.8, 27.8, 27.4, 26.9, 16.6, 16.2, 16.1, 12.9 ppm.

*Due to the close equivalence of multiple carbons, several signals overlap.

HRMS–ESI (*m/z*): $[M+MeOH+H]^+$ calculated 1113.7870; found 1113.8058.

IR (neat, cm^{-1}): 2919, 2848, 1759, 1655, 1614, 1580, 1521, 1450, 1372, 1275, 1241, 1141, 1100, 883, 820, 779, 746.

Rf : 0.61 (30% EtOAc in hexane)

General Procedure for Boc Deprotection and Quinol Oxidation

Boc_2-PPQ (1 equiv.) was dissolved in Et_2O (0.1 M) and $MgBr_2$ (2 equiv.) was added. The reaction was refluxed (35 °C) for 18 h and then cooled to ambient temperature. $FeCl_3$ (2 equiv.) was added and the reaction mixture stirred for 20 min. The reaction mixture was then quenched with EDTA (0.1 M) and the organic layer separated. The aqueous layer was then further extracted with Et_2O x 2. The pooled organic extracts were dried over Na_2SO_4, concentrated *in vacuo* and purified by flash column chromatography.

CoQ10-ω-2-AB (16)

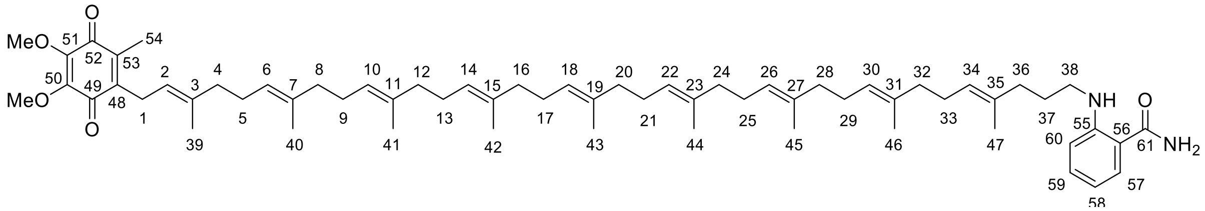

Flash chromatography (30:70 to 50:50 EtOAc/hexane) afforded CoQ10-ω-2-AB (**16**) as an orange oil (16 mg, 0.016 mmol, 65%).

^{1}H NMR (400 MHz; $CDCl_3$): δ 7.36 (1H, dd, *J* = 7.9, 1.5 Hz, H57), 7.30 (1H, ddd, *J* = 8.5, 7.1, 1.5, H59), 6.67 (1H, dd, *J* = 8.5, 0.6 Hz, H60), 6,54 (1H, ddd, *J* = 7.9, 7.1, 1.0 Hz, H58) 5.18 – 5.04 (8H, m, H6, H10, H14, H18, H22, H26, H30, H34), 4.95 – 4.91 (1H, m, H2) 3.99 (3H, s, MeO), 3.97 (3H, s, MeO), 3.18 (2H, d, *J* = 7.0 Hz, H1), 3.12 (2H, t, *J* = 8.0 Hz, H38), 2.17 – 1.92 (37H, m, H4, H5, H8, H9, H12, H13, H16, H17, H20, H21, H24, H25, H28, H29, H32, H33, H36, H54), 1.78 – 1.73 (5H, m, H37, H39), 1.65 – 1.54 (24H, m, H40, H41, H42, H43, H44, H45, H46, H47) ppm.

^{13}C NMR (101 MHz; $CDCl_3$): δ184.9,184.1, 172.2, 150.6, 144.5, 144.4, 141.8, 139.0, 137.8, 135.4, 135.14, 135.09, 135.0, 134.2, 133.7, 128.4, 125.1, 124.44, 124.37, 124.3, 124.0, 119.0, 114.2, 112.7, 111.9, 61.3, 61.3, 42.6, 39.89, 39.87, 39.85, 39.8, 37.2, 29.8 , 27.4, 26.9, 26.8, 26.7, 25.5, 16.5, 16.18, 16.17, 16.1, 12.1 ppm.

*Due to the close equivalence of multiple carbons, several signals overlap.

HRMS–ESI (*m/z*): $[M+H]^+$ calculated 879.6400 ; found 879.6394.

IR (neat, cm^{-1}): 2917, 2848, 1648, 1610, 1579, 1448, 1381, 1262, 1150, 1098, 744.

Rf = 0.64 (50:50 EtOAc:hexane).

MK4-ω-2-AB (17)

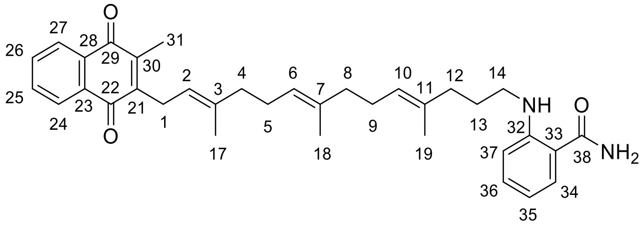

Flash chromatography (30:70 EtOAc/hexane) afforded di-*tert*-butyloxycarbonyl MK4-ω-2-AB (**17**) as an orange oil (3.3 mg, 0.0061 mmol, 44%).

^{1}H-NMR (400 MHz, $CDCl_3$): δ 8.09 – 8.06 (2H, m, H24, H27), 7.80 – 7.78 (1H, m, NH), 7.70 – 7.67 (2H, m, H25, H26), 7.36 – 7.34 (1H, m, H34), 7.31 – 7.27 (1H, m, H36), 6.68 – 6.65 (1H, m, H37), 6.52 (1H, ddd, *J* = 8.0, 7.0, 1.0 Hz, H35), 5.12 – 5.07 (1H, m, H2), 5.07 – 4.97 (2H, m, H6, H10), 3.36 (2H, d, *J* = 6.9 Hz, H1), 3.13 – 3.08 (2H, m, H14), 2.18 (3H, s, H31), 2.08 – 1.92 (10H, m, H4, H5, H8, H9, H12), 1.78 (3H, d, *J* = 1.2 Hz, H17), 1.75 – 1.72 (2H, m, H13), 1.59 – 1.55 (6H, m, H18, H19) ppm.

^{13}C-NMR (151 MHz, $CDCl_3$): δ 185.6, 184.7, 172.2, 146.3, 143.5, 137.7, 135.3, 134.2, 133.6, 133.5, 133.4, 132.33, 132.29, 128.4, 126.3, 124.99, 124.97, 124.1, 119.2, 114.2, 112.7, 111.9, 42.5, 39.8, 39.7, 37.2, 29.9, 27.4, 26.7, 26.6, 26.2, 16.6, 16.2, 16.0, 12.8 ppm.

HRMS–ESI (*m/z*): $[M+H]^+$ calculated 539.3275; found 539.3245.

IR (neat, cm^{-1}): 3466, 3347, 2915, 2848, 1655M 1618M 1580, 1521, 1454, 1379, 1331, 1293, 969, 790, 746.

Rf : 0.41 (50% EtOAc in hexane)

MK9-ω-2-AB (18)

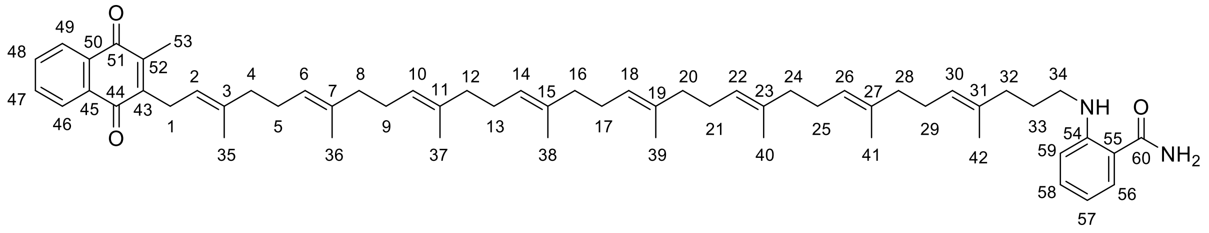

Flash chromatography (20:80 EtOAc:hexane) afforded MK9-ω-2-AB (**18**) as a yellow oil (2.2 mg,0.003 mmol, 60%).

^{1}H-NMR (400 MHz, $CDCl_3$): δ 8.09 – 8.06 (2H, m, H46, H49), 7.82 – 7.79 (1H, m, NH), 7.73 – 7.67 (2H, m, H47, H48), 7.37 – 7.35 (1H, m, H56), 7.32 – 7.28 (1H, m, H58), 6.67 (1H, dd, *J* = 8.1, 0.4 Hz, H59), 6.56 – 6.52 (1H, m, H57), 5.15 – 5.01 (8H, m, H2, H6, H10, H14, H18, H22, H26, H30), 3.37 (2H, d, *J* = 7.2 Hz, H1), 3.13 –3.11 (2H, m, H34), 2.19 (3H, s, H53), 2.07 – 1.94 (30H, m, H4, H5, H8, H9, H12, H13, H16, H17, H20, H21, H24, H25, H28, H29, H32), 1.79 – 1.74 (5H, m, H33, H35), 1.62 – 1.52 (21H, m, H36, H37, H38, H39, H40, H41, H42) ppm.

^{13}**C-NMR** (101 MHz, $CDCl_3$): δ 185.6, 150.6, 147.9, 146.3, 143.5, 143.0, 137.7, 135.10, 135.08, 135.0, 134.2, 133.7, 133.5, 133.4, 132.3, 128.4, 126.5, 126.3, 125.1, 124.5, 124.4, 124.34, 124.29, 124.28, 124.0, 119.2, 114.2, 111.9, 42.6, 39.90, 39.89, 39.8, 37.2, 29.9, 29.8, 27.4, 26.88, 26.86, 26.8, 26.8, 26.7, 26.2, 16.6, 16.20, 16.16, 16.1, 12.8 ppm.

*Due to the close equivalence of multiple carbons, several signals overlap.

HRMS–ESI (*m/z*): $[M+H]^+$ calculated 879.6398 ; found 879.6394.

IR (neat, cm^{-1}): 3353, 2917, 2850, 1659, 1616, 1579, 1519, 1437, 1405, 1381, 1329, 1292, 1258, 1150, 1094, 1018, 800, 744, 714, 691.

Rf : 0.40 (30% EtOAc in hexane)

General procedure for synthesis of ω-labelled Boc_2-CoQ10 via reductive amination

Me_2-CoQ10-ω-epoxide (**8**) (1.23 g, 1.35 mmol) was dissolved in THF:H_2O (9:1, 40 mL) and HIO_4 (369 mg) was added. The reaction was stirred at atmospheric temperature for 2 h at which point TLC confirmed reaction completion. The reaction was concentrated under reduced pressure, diluted with brine:H_2O (1:1, 50 mL) and extracted with EtOAc (3 x 50 mL). The organic extracts were dried over Na_2SO_4 and solvent removed *in vacuo* to afford crude ω-aldehyde (1.02 g, 1.18 mmol, 88%) as a clear viscous oil. ω-Aldehyde (1 equiv.) was dissolved in DCE or CH_2Cl_2 (1.5 mL) and amine (1.2 equiv.) was added, followed by AcOH (1.8 equiv.) in DCE or CH_2Cl_2 and stirred at ambient temperature under argon for 5 min. $NaBH(OAc)_3$ (2 equiv.) was added and the reaction mixture stirred at ambient temperature under argon for 3 – 18 h, at which point TLC confirmed reaction completion. The solvent was removed *in vacuo,* quenched with $NaHCO_3$ (*sat.*, 2 mL) and extracted with EtOAc (3 x 2 mL). The organic extracts were dried over Na_2SO_4, concentrated *in vacuo* and purified by flash chromatography to yield amines (**19** – **23**).

Boc$_2$-CoQ10-ω-alkyne (19a)

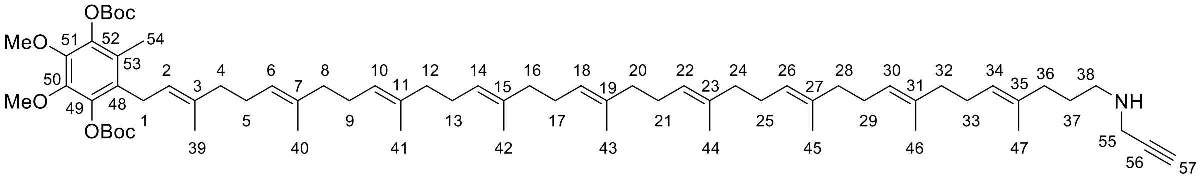

Solvent = CH_2Cl_2, reaction time = 18 h. Flash chromatography (10:90 to 40:60 EtOAc/hexane) afforded Boc$_2$-CoQ10-ω-alkyne (**19a**) as a viscous clear oil (24 mg, 0.023 mmol, 25%).

^{1}H NMR (400 MHz; $CDCl_3$): δ 5.15 – 5.05 (8H, m, H6, H10, H14, H18, H22, H26, H30, H34), 5.03 – 4.99 (1H, m, H2), 3.862 (3H, s, MeO), 3.860 (3H, s, MeO), 3.41 (2H, d, *J* = 2.4 Hz, H55) 3.26 (2H, d, *J* = 6.4 Hz, H1), 2.66 (2H, t, *J* = 7.2 Hz, H38), 2.20 (1H, t, *J* = 2.4 Hz, H57) 2.11 – 1.93 (39H, m, H4, H5, H8, H9, H12, H13, H16, H17, H20, H21, H24, H25, H28, H29, H32, H33, H36, H37, H54), 1.74 (3H, s, H39), 1.60 – 1.57 (24H, m, H40 H41, H42, H43, H44, H45, H46, H47) 1.56 (9 H, s, *t*Bu) 1.53 (9 H, s, *t*Bu) ppm.

^{13}C NMR (101 MHz; $CDCl_3$): δ 151.6, 143.7, 143.5, 140.7, 135.8, 135.1, 134.94, 134.85, 128.4, 124.9, 124.6, 124.3, 124.1, 121.2, 83.3, 83.1, 82.4, 71.1, 60.7, 60.7, 48.4, 39.77, 39.75, 39.7, 38.2, 37.3, 26.8, 26.1, 16.3, 16.1, 16.0, 11.9 ppm.

*Due to the close equivalence of multiple carbons, several signals overlap.

HRMS–ESI (*m/z*): $[M+H]^+$ calculated 1078.8070; found 1078.7983.

IR (neat, cm^{-1}): 2978, 2922, 2851, 1759, 1454, 1368, 1275, 1234, 1144, 1111, 1047, 910, 857, 731.

Rf = 0.60 (20:80 EtOAc:hexane).

Boc$_2$-CoQ10-ω-CF$_3$ (20a)

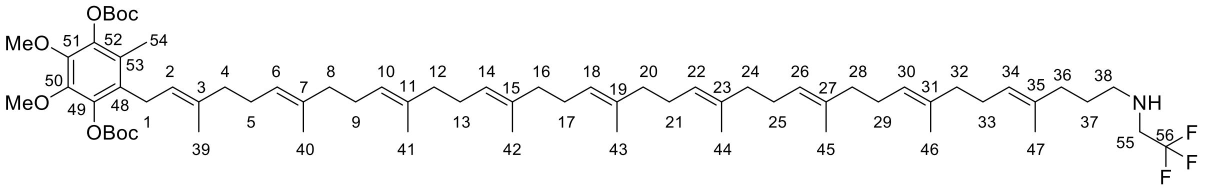

2,2,2-Trifluoroethylamine was prepared from 2,2,2-trifluoroethylamine hydrochloride (18 mg, 0.129 mmol) in CH_2Cl_2 (1 mL) with DIPEA (22 µL, 0.129 mmol). Solvent = CH_2Cl_2, reaction time = 3 h. Flash chromatography (5:95 to 10:90 EtOAc/hexane) afforded Boc$_2$-CoQ10-ω-CF$_3$ (**20a**) as a viscous clear oil (39 mg, 0.035 mmol, 32%).

^{1}H NMR (400 MHz; $CDCl_3$): δ 5.15 – 5.05 (8H, m, H6, H10, H14, H18, H22, H26, H30, H34), 5.03 – 4.99 (1H, m, H2), 3.86 (3H, s, MeO), 3.85 (3H, s, MeO), 3.26 (2H, d, J = 6.8 Hz, H1), 3.15 (2H, q, J = 9.5 Hz, H55) 2.67 (2H, t, J = 7.2 Hz, H38) 2.11 – 1.93 (37H, m, H4, H5, H8, H9, H12, H13, H16, H17, H20, H21, H24, H25, H28, H29, H32, H33, H36, H37, H54), 1.74 (3H, s, H39), 1.60 – 1.56 (24H, m, H40, H41, H42, H43, H44, H45, H46, H47) 1.55 (9 H, s, *t*Bu) 1.53 (9 H, s, *t*Bu) ppm.

^{13}C-NMR (101 MHz, $CDCl_3$): δ 151.7, 151.6, 143.8, 143.6, 141.1, 140.8, 135.9, 135.2, 135.1, 135.0, 134.9, 134.4, 128.5, 125.99, 124.96, 124.5, 124.41, 124.38, 124.36, 124.2, 121.3, 83.4, 83.23, 60.9, 60.8, 51.2, 50.9, 50.6, 50.3, 49.3, 39.9, 39.82, 39.80, 37.1, 28.3, 27.79, 27.77, 26.9, 26.8, 26.2, 16.4, 16.2, 16.1, 16.0, 12.1 ppm.

*Due to the close equivalence of multiple carbons, several signals overlap.

^{19}F NMR (373 MHz; $CDCl_3$): – 71.69 (t, J = 10.0 Hz) ppm.

HRMS–ESI (*m/z*): $[M+H]^+$ calculated 1122.7944; found 1122.8013.

IR (neat, cm^{-1}): 2917, 2848, 1649, 1610, 1448, 1260, 1189, 1090, 1012, 796.

Rf = 0.56 (20:80 EtOAc:hexane).

Boc$_2$-CoQ10-ω-pyrene (21a)

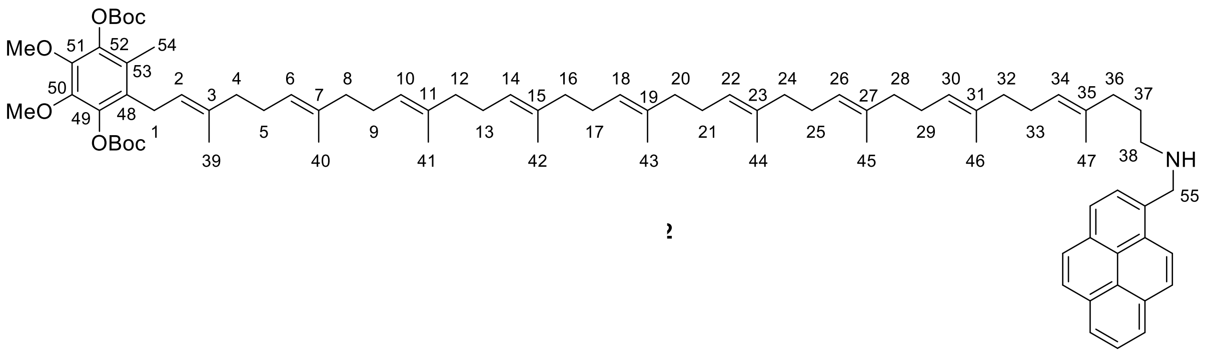

1-Pyrenemethylamine was prepared from 1-pyrenemethylamine hydrochloride (29 mg, 0.108 mmol) in CH_2Cl_2 (1 mL) and DIPEA (19 µL, 0.108 mmol). Solvent = CH_2Cl_2, reaction time = 18 h. Flash chromatography (10:90 to 50:50 EtOAc/hexane) afforded Boc$_2$-CoQ10-ω-pyrene (**21a**) as a viscous clear oil (29 mg, 0.023 mmol, 25%).

^{1}H NMR (400 MHz; $CDCl_3$): δ 8.37 (1H, d, *J* = 9.2 Hz, pyr-H) 8.18 – 8.11 (4H, m, 4 x pyr-H), 8.03 – 7.99 (4H, m, 4 x pyr-H), 5.16 – 5.05 (8H, m, H6, H10, H14, H18, H22, H26, H30, H34), 5.03 – 4.99 (1H, m, H2), 4.48 (2H, s, H55), 3.861 (3H, s, MeO), 3.859 (3H, s, MeO), 3.26 (2H, d, *J* = 6.4 Hz, H1), 2.77 (2H, t, *J* = 7.2 Hz, H38), 2.11 – 1.93 (37H, m, H4, H5, H8, H9, H12, H13, H16, H17, H20, H21, H24, H25, H28, H29, H32, H33, H36, H54), 1.74 (3H, d, *J* = 0.9 Hz, H39), 1.71 – 1.67 (2H, m, H37),1.60 – 1.57 (24H, m, H40, H41, H42, H43, H44, H45, H46, H47), 1.56 (9 H, s, *t*Bu) 1.53 (9 H, s, *t*Bu) ppm.

^{13}C NMR (101 MHz; $CDCl_3$): δ 151.7, 151.6, 143.8, 143.6, 141.1, 140.9, 135.9, 135.2, 135.1, 135.0, 134.7, 131.5, 131.0, 130.8, 128.5, 127.8, 127.6, 127.2, 127.1, 126.0, 125.2, 125.1,

125.0, 124.8, 124.4, 124.2, 123.4, 121.4, 83.4, 83.2, 60.9, 52.0, 49.8, 39.9, 39.8, 37.5, 28.5, 27.8, 26.9, 26.8, 16.18, 16.16, 16.15, 12.1 ppm.

*Due to the close equivalence of multiple carbons, several signals overlap.

HRMS–ESI (*m/z*): $[M+H]^+$ calculated 1254.8700; found 1254.8699.

IR (neat, cm^{-1}): 2920, 2850, 1757, 1448, 1368, 1277, 1234, 1144, 1107, 844, 800.

Rf = 0.53 (20:80 EtOAc:hexane).

Boc_2-CoQ10-ω-PNB (22a)

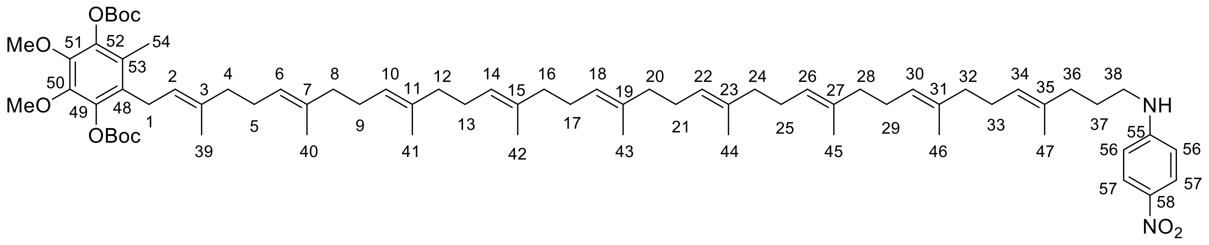

Solvent = DCE, reaction time = 3 h. Flash chromatography (10:90 EtOAc/hexane) afforded Boc_2-CoQ10-ω-PNB (**22a**) as a viscous yellow oil (34 mg, 0.029 mmol, 37%).

^{1}H NMR (400 MHz; $CDCl_3$): δ 8.08 (2H, d, *J* = 9.2 Hz, H57), 6.50 (2H, d, *J* = 9.2 Hz, H56) 5.20 – 5.05 (8H, m, H6, H10, H14, H18, H22, H26, H30, H34), 5.03 – 4.99 (1H, m, H2), 4.54 – 4.52 (1H, m, NH) 3.870 (3H, s, MeO), 3.876 (3H, s, MeO), 3.27 (2H, d, *J* = 6.5 Hz, H1), 3.18 (2H, q, *J* = 6.4 Hz, H38) 2.11 – 1.93 (35H, m, H4, H5, H8, H9, H12, H13, H16, H17, H20, H21, H24, H25, H28, H29, H32, H33, H36, H54), 1.78 – 1.73 (5H, m, H37, H39), 1.60 – 1.57 (24H, m, H40, H41, H42, H43, H44, H45, H46, H47) 1.56 (9 H, s, *t*Bu) 1.55 (9 H, s, *t*Bu) ppm.

^{13}C NMR (101 MHz; $CDCl_3$): δ 153.5, 151.7, 151.6, 143.8, 143.6, 141.1, 140.8, 138.0, 135.9, 135.2, 135.1, 135.04, 135.03, 135.0, 134.8, 133.7, 128.5, 126.6, 125.7, 125.0, 124.64, 124.55,

124.53, 124.52, 124.47, 124.42, 124.39, 124.2, 121.3, 111.0, 83.4, 83.3, 60.9, 60.83, 60.80, 43.0, 39.89, 39.88 39.82, 39.76, 37.0, 27.79, 27.78, 27.1, 26.9, 26.7, 26.2, 16.4, 16.2, 16.1, 16.0, 12.1 ppm.

*Due to the close equivalence of multiple carbons, several signals overlap.

HRMS–ESI (*m/z*): $[M+H]^+$ calculated 1161.8077; found 1161.8042.

IR (neat, cm^{-1}): 3390, 2976, 2920, 2850, 1757, 1601, 1478, 1368, 1321, 1305, 1277, 1234, 1142, 1109, 1049, 584, 833, 753.

Rf = 0.59 (30:70 EtOAc:hexane).

Boc₂-CoQ10-ω-diazirine (23a)

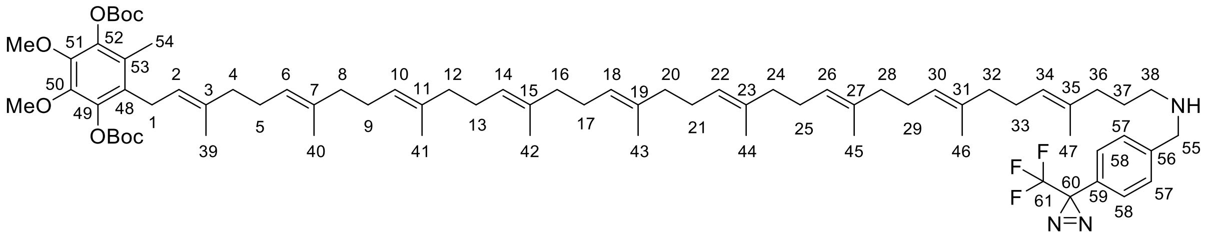

4-[3-(Trifluoromethyl)-3H-diazirin-3-yl]-benzylamine was prepared from 4-[3-(trifluoromethyl)-3H-diazirin-3-yl]-benzylamine hydrochloride (29 mg, 0.114 mmol) in CH_2Cl_2 (1 mL) with DIPEA (20 µL, 0.114 mmol). Solvent = CH_2Cl_2, reaction time = 3 h. The solvent was removed *in vacuo*., quenched with $NaHCO_3$ (*sat*., 2 mL) and extracted with EtOAc (3 x 2 mL). The organic extracts were dried over Na_2SO_4 and solvent removed *in vacuo*. Flash chromatography (5:95 to 10:90 EtOAc/hexane) afforded Boc₂-CoQ10-ω-diazirine (**23a**) as a viscous pale yellow oil (40 mg, 0.032mmol, 34%).

^{1}H NMR (400 MHz; $CDCl_3$): δ 7.35 (2H, d, *J* = 8.5 Hz, H57) 7.14 (2H, d, *J* = 7.9 Hz, H58) 5.15 – 5.05 (8H, m, H6, H10, H14, H18, H22, H26, H30, H34), 5.03 – 4.99 (2H, m, H2), 3.859 (3H,

s, MeO), 3.856 (3H, s, MeO), 3.78 (2H, s, H55) 3.26 (2H, d, J = 6.4 Hz, H1), 2.57 (2H, t, J = 7.2 Hz, H38) 2.11 – 1.93 (37H, m, H4, H5, H8, H9, H12, H13, H16, H17, H20, H21, H24, H25, H28, H29, H32, H33, H36, H37, H54), 1.74 (3H, s, H39), 1.60 – 1.56 (24H, m, H41, H41, H42, H43, H44, H45, H46, H47), 1.55 (9 H, s, *t*Bu) 1.53 (9 H, s, *t*Bu) ppm.

^{13}C NMR (101 MHz; $CDCl_3$): δ 151.8, 151.7, 143.9, 143.7, 141.2, 141.0, 136.0, 135.3, 135.2, 135.14, 135.05, 134.7, 128.8, 128.7, 127.9, 126.8, 126.7, 125.1, 124.9, 124.6, 124.52, 124.50, 124.3, 123.8, 121.5, 83.5, 83.3, 60.1, 60.94, 60.92, 60.91, 53.6, 49.3, 40.00, 39.98, 39.9, 37.6, 29.9, 27.90, 27.88, 27.0, 26.9, 26.3, 16.5, 16.27, 16.26, 16.25, 16.1, 12.2 ppm.

*Due to the close equivalence of multiple carbons, several signals overlap.

^{19}F NMR (376 MHz; $CDCl_3$): – 65.28 (s) ppm.

HRMS–ESI (*m/z*): $[M+H]^+$ calculated 1238.8318; found 1238.8397.

IR (neat, cm^{-1}): 2974, 2922, 2851, 1759, 1480, 1368, 1279, 1234, 1148, 1111, 973, 939, 857, 779, 734.

Rf = 0.75 (20:80 EtOAc:hexane).

CoQ10-ω-alkyne (19)

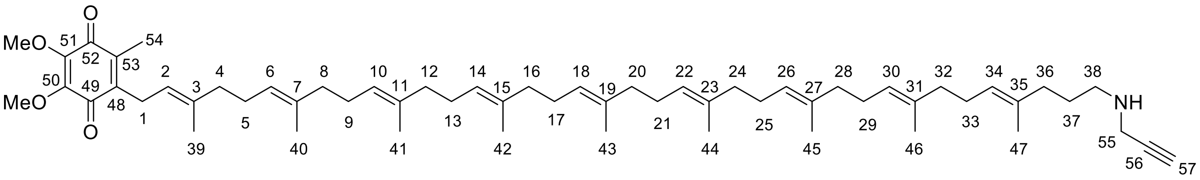

CoQ10-ω-alkyne (**19**) was synthesized from **19a** according to general procedure for Boc deprotection and quinol oxidation described above. Flash chromatography (10:90 to 30:70

EtOAc/hexane) afforded the CoQ10-ω-alkyne (**19**) as a viscous clear oil (9 mg, 0.012 mmol, 48%).

^{1}H NMR (400 MHz; $CDCl_3$): δ 5.12 – 5.04 (8H, m, H6, H10, H14, H18, H22, H26, H30, H34), 4.95 – 4.91 (1H, m, H2), 3.99 (3H, s, MeO), 3.98 (3H, s, MeO), 3.41 (2H, d, *J* = 2.4 Hz, H55) 3.18 (2H, d, *J* = 7.1 Hz, H1), 2.65 (2H, t, *J* = 7.2 Hz, H38), 2.20 (1H, t, *J* = 2.4 Hz, H57) 2.09 – 1.93 (39H, m, H4, H5, H8, H9, H12, H13, H16, H17, H20, H21, H24, H25, H28, H29, H32, H33, H36, H37, H54), 1.74 (3H, s, H39), 1.62 – 1.54 (24H, m, H40, H41, H42, H43, H44, H45, H46, H47) ppm.

^{13}C NMR (101 MHz; $CDCl_3$): δ 184.8, 183.9, 144.4, 144.2, 141.7, 138.9, 137.6, 135.3, 135.0, 134.94, 134.92, 134.8, 134.5, 124.6, 124.3, 124.24, 124.15, 123.8, 118.9, 71.1, 61.12, 61.08, 48.4, 39.8, 39.72, 39.68, 39.63, 39.60, 38.2, 37.3, 29.7, 28.1, 26.73, 26.66, 26.6, 26.5, 25.3, 16.4, 16.0, 15.93, 15.87, 11.9 ppm.

*Due to the close equivalence of multiple carbons, several signals overlap.

HRMS–ESI (*m/z*): $[M+H]^+$ calculated 876.6864; found 876.6871.

IR (neat, cm^{-1}): 2917, 2846, 1648, 1648, 1610, 1431, 1381, 1260, 1098, 1023, 837, 800, 734, 691.

Rf = 0.59 (10:90 MeOH:CH_2Cl_2).

CoQ10-ω-CF_3 (20)

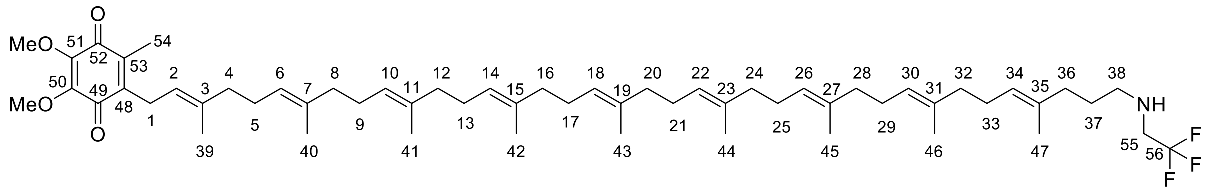

CoQ10-ω-CF_3 (**20**) was synthesized from **20a** according to general procedure for Boc deprotection and quinol oxidation described above. Flash chromatography (5:95 to 10:90 EtOAc/hexane) afforded CoQ10-ω-CF_3 (**20**) as an orange oil (13 mg, 0.035 mmol, 61%).

^{1}H NMR (400 MHz; $CDCl_3$): δ 5.15 – 5.04 (8H, m, H6, H10, H14, H18, H22, H26, H30, H34), 4.95 – 4.92 (1H, m, H2), 3.99 (3H, s, MeO), 3.98 (3H, s, MeO), 3.20 – 3.13 (4H, m, H1, H55), 2.67 (2H, t, *J* = 7.2 Hz, H38), 2.14 – 1.92 (37H, m, H4, H5, H8, H9, H12, H13, H16, H17, H20, H21, H24, H25, H28, H29, H32, H33, H36, H54), 1.74 (3H, s, H39), 1.66 – 1.48 (24H, m, H40, H41, H42, H43, H44, H45, H46, H47) ppm.

^{13}C NMR (101 MHz; $CDCl_3$): δ 184.8, 183.9, 144.4, 144.2, 141.7, 138.9, 137.6, 135.3, 135.0, 134.9, 134.8, 134.2, 124.8, 124.3, 124.3, 124.2, 123.8, 118.9, 61.1, 50.7, 50.4, 49.1, 39.8, 39.7, 39.6, 37.0, 28.2, 26.7, 26.6, 26.5, 25.3, 16.4, 16.0, 15.9, 15.8, 12.0 ppm.

*Due to the close equivalence of multiple carbons, several signals overlap.

^{19}F NMR (376 MHz; $CDCl_3$): – 71.68 (t, *J* = 2.5 Hz) ppm.

HRMS–ESI (*m/z*): $[M+H]^+$ calculated 920.6734; found 920.6646.

IR (neat, cm^{-1}): 2976, 2920, 2850, 1757, 1450, 1370, 1275, 1234, 1142, 1107, 1049, 855, 738.

Rf = 0.36 (20:80 EtOAc:hexane).

CoQ10-ω-pyrene (21)

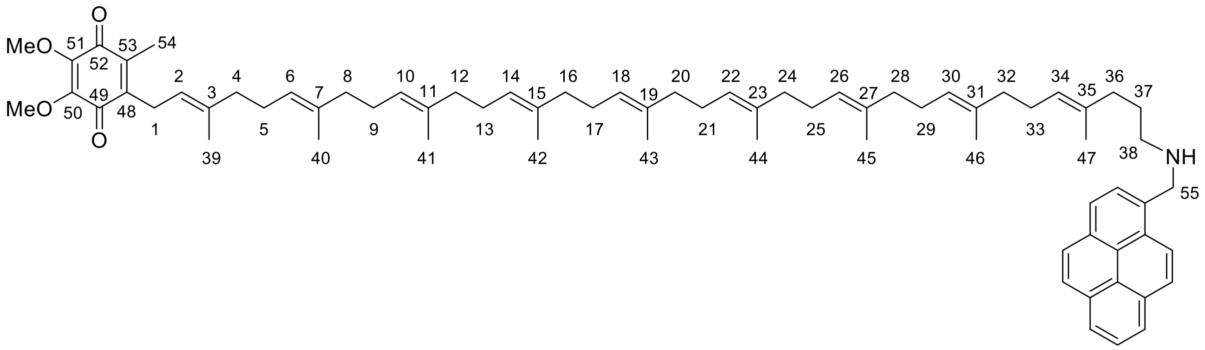

CoQ10-ω-pyrene (**21**) was synthesized from **21a** according to general procedure for Boc deprotection and quinol oxidation described above. Flash chromatography (0:100 to 10:100 MeOH/CH_2Cl_2) afforded CoQ10-ω-pyrene (**21**) as an orange oil (12 mg, 0.011 mmol, 55%).

^{1}H NMR (400 MHz; $CDCl_3$): δ 8.37 (1H, d, *J* = 9.2 Hz, pyr-H) 8.18 – 8.11 (4H, m, 4 x pyr-H), 8.05 – 7.98 (4H, m, 4 x pyr-H), 5.12 – 5.04 (8H, m, H6, H10, H14, H18, H22, H26, H30, H34), 4.95 – 4.91 (1H, m, H2) 4.49 (2H, s, H39), 3.98 (3H, s, MeO), 3.97 (3H, s, MeO), 3.17 (2H, d, *J* = 6.8 Hz, H1), 2.77 (2H, t, *J* = 7.0 Hz, H38), 2.12 – 1.86 (37H, m, H4, H5, H8, H9, H12, H13, H16, H17, H20, H21, H24, H25, H28, H29, H32, H33, H36, H54), 1.73 (3H, s, H39), 1.62 – 1.66 (2H, m, H37), 1.62 – 1.55 (24H, m, H40, H41, H42, H43, H44, H45, H46, H47) ppm.

^{13}C NMR (101 MHz; $CDCl_3$): δ 184.7, 183.9, 144.38, 144.27, 144.25, 144.24, 141.7, 138.8, 137.6, 135.2, 135.00, 134.94, 134.93, 134.84, 134.5, 131.3, 130.85, 130.65, 129.1, 127.63, 127.46, 127.0, 126.6, 125.9, 125.06, 124.97, 124.92, 124.69, 124.63, 124.24, 124.15, 123.8, 123.2, 118.9, 61.1, 49.6, 39.75, 39.73, 39.67, 37.4, 28.2, 26.73, 26.67, 26.53, 25.3, 16.3, 16.03, 15.87, 11.9 ppm.

*Due to the close equivalence of multiple carbons, several signals overlap.

HRMS–ESI (*m/z*): $[M+H]^+$ calculated 1052.7491 ; found 1052.7411.

IR (neat, cm^{-1}): 2907, 2842, 1607, 1448, 1426, 1377, 1260, 1105, 1016, 908, 844, 800, 731,

Rf = 0.67 (10:90 MeOH:CH_2Cl_2).

CoQ10-ω-PNB (22)

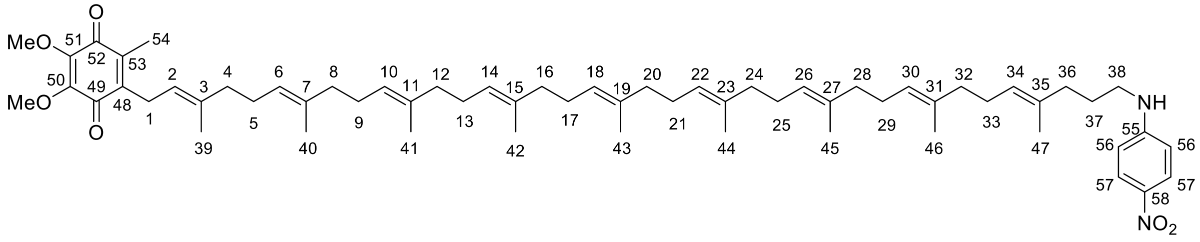

CoQ10-ω-PNB (**22**) was synthesized from **22a** according to general procedure for Boc deprotection and quinol oxidation described above. Flash chromatography (10:90 EtOAc/hexane) afforded CoQ10-ω-PNB (**22**) as a viscous yellow oil (21 mg, 0.022 mmol, 78%).

^{1}H NMR (400 MHz; $CDCl_3$): δ 8.08 (2H, d, J = 9.2 Hz, H57), 6.50 (2H, d, J = 9.2 Hz, H56) 5.18 – 5.04 (8H, m, H6, H10, H14, H18, H22, H26, H30, H34), 4.95 – 4.92 (1H, m, H2), 4.51 – 4.49 (1H, m, NH) 3.99 (3H, s, MeO), 3.97 (3H, s, MeO), 3.21 – 3.15 (4H, m, H1, H38), 2.10 – 1.93 (37H, m, H4, H5, H8, H9, H12, H13, H16, H17, H20, H21, H24, H25, H28, H29, H32, H33, H36, H54), 1.78 – 1.72 (5H, m, H37, H39), 1.63 – 1.57 (24H, m, H40, H41, H42, H43, H44, H45, H46, H47) ppm.

^{13}C NMR (101 MHz; $CDCl_3$): δ 184.8, 183.9, 153.3, 144.4, 141.7, 138.9, 137.6, 135.3, 135.00, 134.95, 134.93, 134.91, 134.85, 134.7, 133.6, 126.5, 125.6, 124.5, 124.4, 124.32, 124.27, 124.26, 124.24, 124.15, 123.9, 118.9, 110.9, 61.2, 61.14, 42.9, 39.8, 39.73, 39.72, 39.6, 36.9, 27.0, 26.73, 26.71, 26.67, 26.6, 26.5, 25.3, 16.4, 16.04, 16.03, 16.00, 15.9, 12.0 ppm.

*Due to the close equivalence of multiple carbons, several signals overlap.

HRMS–ESI (*m/z*): $[M+Cl]^-$ calculated 993.6493; found 993.6446.

IR (neat, cm^{-1}): 3379, 2913, 2846, 1648, 1599, 1448, 1320, 1286, 1262, 1185, 1109, 1021, 831, 753, 695.

Rf = 0.59 (30:70 EtOAc:hexane).

CoQ10-ω-diazirine (23)

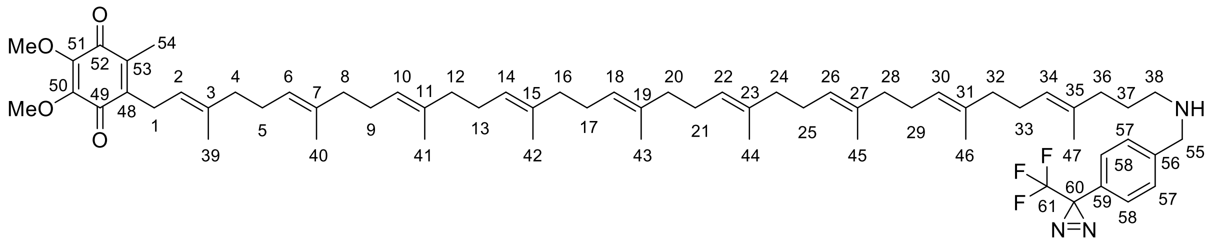

CoQ10-ω-diazirine (**23**) was synthesized from **23a** according to general procedure for Boc deprotection and quinol oxidation described above. Flash chromatography (0:100 to 30:90 EtOAc/hexane) afforded CoQ10-ω-diazirine (**23**) as an orange oil (21 mg, 0.020 mmol, 75%).

^{1}H-NMR (400 MHz, $CDCl_3$): δ 7.39 (2H, d, *J* = 8.2 Hz, H58), 7.15 (2H, d, *J* = 8.1 Hz, H57), 5.13 – 5.06 (8H, m, H6, H10, H14, H18, H22, H26, H30, H34), 4.95 – 4.91 (1H, m, H2), 3.99 (3H, s, MeO), 3.98 (3H, s, MeO), 3.83 (2H, s, H55), 3.18 (2H, d, *J* = 7.0 Hz, H1), 2.62 (2H, t, *J* = 7.4 Hz, H38), 2.07 – 1.95 (37H, m, H4, H5, H8, H9, H12, H13, H16, H17, H20, H21, H24, H25, H28, H29, H32, H33, H36, H54), 1.74 (3H, d, *J* = 0.5 Hz, H39), 1.64 – 1.53 (24H, m, H40, H41, H42, H43, H44, H45, H46, H47) ppm.

^{13}C-NMR (151 MHz, $CDCl_3$): δ 184.8, 183.9, 144.4, 144.2, 141.7, 138.9, 137.6, 135.3, 135.02, 135.01, 134.99, 134.95, 134.94, 134.93, 134.8, 129.12, 129.10, 126.7, 125.16, 125.15, 124.4, 124.27, 124.25, 124.2, 123.8, 118.8, 61.2, 61.1, 39.8, 39.73, 39.6, 37.0, 30.6, 29.70, 29.67, 26.74, 26.71, 26.67, 26.65, 26.6, 26.5, 25.3, 16.4, 16.04, 16.00, 15.96, 15.7, 12.0 ppm.

*Due to the close equivalence of multiple carbons, several signals overlap.

^{19}F-NMR (565 MHz, $CDCl_3$): δ – 65.24 (s) ppm.

HRMS–ESI (*m/z*): $[M+H]^+$ calculated 1036.7113; found 1036.6964.

IR (neat, cm^{-1}): 2920, 2850, 1649, 1610, 1448, 1381, 1344, 1262, 1230, 1183, 1154, 1100, 1025, 937, 835, 800, 734.

Rf = 0.44 (10% MeOH in CH_2Cl_2).

General Procedure for Synthesis of Boc_2-PPQ-ω-OTs (24 and 25)

ω-Tosylates (**24** and **25**) were prepared according to modified literature procedures.[4] Boc_2-PPQ-ω-epoxide (**9** or **11**) was dissolved in THF:H_2O (9:1, 0.1 M) and HIO_4 (1.2 equiv.) added. The reaction mixture was stirred at room temperature for 1.5 h, at which point TLC indicated reaction completion. The solvent was removed *in vacuo* and the reaction mixture quenched with brine:H_2O (1:1). This was extracted with Et_2O three times, the combined organic extracts dried over Na_2SO_4 and solvent removed *in vacuo* to afford crude ω-aldehyde. This was dissolved in Et_2O (0.1 M) and $NaBH_4$ (1.5 equiv.) in MeOH added. The reaction mixture was stirred for 2 h at room temperature, until TLC indicated reaction completion. The solvent was removed under reduced pressure, the reaction mixture quenched with brine:H_2O (1:1) and extracted with EtOAc. The combined organic extracts were dried over Na_2SO_4 and the solvent removed. The resulting crude alcohol was dissolved in CH_2Cl_2 (0.1 M) and tosyl chloride (3 equiv.), NEt_3 (4 equiv.) and DMAP (0.1 equiv.) were added. The reaction mixture was stirred at room temperature for 18 h, at which point TLC indicated reaction completion. The reaction mixture was diluted with CH_2Cl_2, washed with HCl, H_2O and brine. The organic layer was dried over Na_2SO_4 and solvent removed. Flash chromatography afforded ω-tosylate (**24** or **25**).

Boc$_2$-CoQ10-ω-OTs (24)

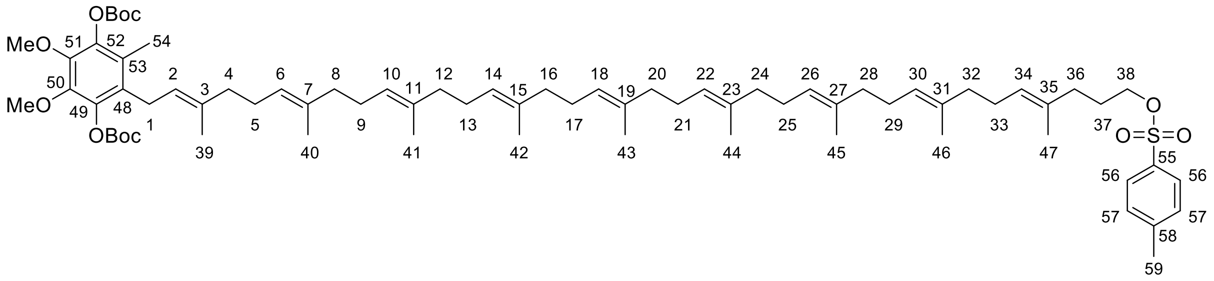

Boc$_2$-CoQ10-ω-OTs (**24**) was prepared according to the general procedure above and isolated as a viscous colourless oil (240 mg, 0.199 mmol, 74%).

^{1}H-NMR (600 MHz, $CDCl_3$): δ 7.79 (2H, d, *J* = 8.2 Hz, H56), 7.34 (2H, d, *J* = 8.1 Hz, H57), 5.12 – 5.08 (7H, m, H6, H10, H14, H18, H22, H26, H30), 5.02 – 5.00 (2H, m, H2, H34), 4.00 (2H, t, *J* = 6.5 Hz, H38), 3.86 (d, *J* = 1.4 Hz, 6H), 3.26 (2H, d, *J* = 6.5 Hz, H1), 2.44 (3H, s, H59), 2.07 – 1.96 (37H, m, H4, H5, H8, H9, H12, H13, H16, H17, H20, H21, H24, H25, H28, H29, H32, H33, H36, H54), 1.75 – 1.72 (5H, m, H37, H39), 1.60 – 1.57 (24H, m, H40, H41, H42, H43, H44, H45, H46, H47), 1.56 (9H, s, *t*Bu), 1.54 (9H, s, *t*Bu) ppm.

^{13}C-NMR (151 MHz, $CDCl_3$): δ 151.7, 151.6, 144.7, 143.8, 143.6, 141.1, 140.8, 135.9, 135.2, 135.1, 135.0, 134.8, 133.4, 133.0, 129.9, 128.5, 128.0, 125.8, 125.0, 124.5, 124.40, 124.37, 124.2, 121.3, 83.4, 83.3, 70.3, 60.9, 60.8, 39.90, 39.87, 39.8, 39.7, 35.2, 27.79, 27.77, 27.2, 26.88, 26.86, 26.8, 26.2, 21.8, 16.4, 16.18, 16.16, 16.15, 15.9, 12.1 ppm.

*Due to the close equivalence of multiple carbons, several signals overlap.

HRMS–ESI (*m/z*): $[M+Na]^+$ calculated 1217.7661 ; found 1217.7882.

IR (neat, cm^{-1}): 2976, 2917, 2850, 2757, 1426, 1366, 1277, 1234, 1144, 969, 924, 855, 814, 779, 664.

Rf = 0.56 (20:80 EtOAc:hexane).

Boc$_2$-MK9-ω-OTs (25)

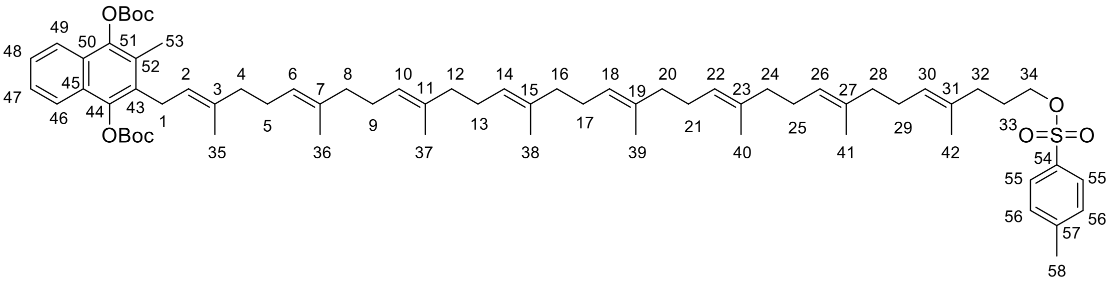

Flash chromatography (10:90 EtOAc/hexane) afforded Boc$_2$-MK9-ω-OTs (**25**) as a colourless oil (101 mg, 0.09 mmol, 53%).

^{1}H-NMR (400 MHz, $CDCl_3$): δ 7.83 – 7.77 (4H, m, H46, H49, H55), 7.49 – 7.47 (2H, m, H50, H51), 7.34 (2H, d, J = 7.9 Hz, H59), 5.13 – 5.09 (7H, m, H2, H6, H10, H14, H18, H22, H26), 5.04 – 5.00 (1H, m, H30), 4.00 (2H, t, J = 6.5 Hz, H34), 3.48 (2H, d, J = 6.2 Hz, H1), 2.44 (3H, s, H58), 2.30 (3H, s, H53), 2.07 – 1.96 (30H, m, H4, H5, H8, H9, H12, H13, H16, H17, H20, H21, H24, H25, H28, H29, H32), 1.80 (3H, d, J = 1.0 Hz, H35), 1.73 – 1.71 (2H, m, H33), 1.60 – 1.56 (39H, m, H36, H37, H39, H40, H41, H42, H43, 2 x *t*Bu) ppm.

^{13}C-NMR (101 MHz, $CDCl_3$): δ 152.1, 151.7, 144.7, 142.8, 142.4, 136.3, 135.3, 135.1, 135.0, 134.8, 133.0, 130.7, 129.9, 128.0, 127.3, 126.7, 126.6, 126.5, 126.4, 125.8, 124.5, 124.42, 124.39, 124.37, 124.2, 121.3, 121.22, 121.17, 83.6, 70.3, 39.90, 39.87, 39.8, 39.7, 35.2, 27.9, 27.8, 27.2, 26.93, 26.89, 26.86, 26.8, 21.8, 16.6, 16.18, 16.17, 16.15, 15.9, 12.9 ppm.

*Due to the close equivalence of multiple carbons, several signals overlap.

HRMS–ESI (*m/z*): $[M+H]^+$ calculated 1117.7161; found 1117.7037.

IR (neat, cm^{-1}): 2976, 2917, 2851, 1759, 1448, 1368, 1241, 1139, 1100, 1077, 814, 742.

Rf : 0.53 (20% EtOAc in hexane)

General Procedure for Synthesis of Boc$_2$-PPQ-ω-N$_3$ (26 and 27)

ω-Azides (**26** and **27**) were prepared using a modified literature procedure.[5] Boc$_2$-PPQ-ω-OTs was dissolved in DMF (0.1 M) and sodium azide (2 equiv.) added. The reaction mixture was stirred at room temperature for 18 h at which point TLC indicted reaction completion, and the solvent removed *in vacuo*. Flash chromatography afforded Boc$_2$-PPQ-ω-N$_3$ **(26** or **27)**.

Boc$_2$-CoQ10-ω-N$_3$ (26)

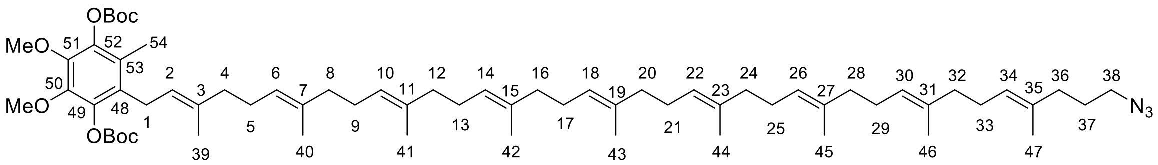

Flash chromatography (10:90 EtOAc/hexane) afforded Boc$_2$-CoQ10-ω-N$_3$ (**26**) as a viscous clear oil (127 mg, 0.119 mmol, 71%).

^{1}H NMR (400 MHz; CDCl$_3$): δ 5.16 – 5.09 (8H, m, H6, H10, H14, H18, H22, H26, H30, H34), 5.02 – 5.01 (1H, m, H2), 3.870 (3H, s, MeO), 3.868 (3H, s, MeO), 3.27 – 3.22 (4H, m, H1, H38), 2.11 – 1.96 (37H, m, H4, H5, H8, H9, H12, H13, H16, H17, H20, H21, H24, H25, H28, H29, H32, H33, H36, H54), 1.73 (3H, s, H39), 1.70 – 1.68 (2H, m , H37), 1.62 – 1.57 (24H, m, H40, H41, H42, H43, H44, H45, H46, H47), 1.55 (9 H, s, *t*Bu) 1.54 (9 H, s, *t*Bu) ppm.

^{13}C NMR (101 MHz; CDCl$_3$): δ 151.8, 151.7, 143.9, 143.7, 141.2, 140.9, 136.0, 135.3, 135.2, 135.1, 134.9, 133.5, 128.6, 125.8, 125.1, 124.7, 124.5, 124.5, 124.3, 121.4, 77.5, 83.5, 83.3, 61.0, 60.92, 60.90, 51.1, 39.98, 39.96, 39.9, 39.8, 36.72, 27.88, 27.86, 27.2, 27.0, 26.9, 26.8, 26.3, 16.5, 16.3, 16.2, 16.0, 12.2 ppm.

*Due to the close equivalence of multiple carbons, several signals overlap.

HRMS–ESI (*m/z*): $[M+H]^+$ calculated 1066.78178; found 1066.8155.

IR (neat, cm^{-1}): 2976, 2933, 2850, 2095, 1757, 1448, 1370, 1277, 1234, 1144, 1109, 1049, 855.

Rf = 0.74 (20:80 EtOAc:hexane).

Boc$_2$-MK9-ω-N$_3$ (27)

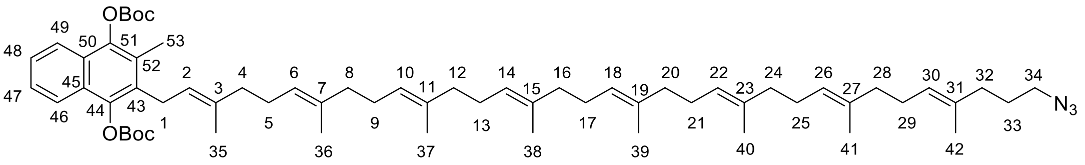

Flash chromatography (5:95 EtOAc:hexane) afforded Boc$_2$-MK9-ω-N$_3$ (**27**) as a viscous colourless oil (39.6 mg, 0.040 mmol, 79%).

^{1}H-NMR (400 MHz, $CDCl_3$): δ 7.84 – 7.76 (2H, m, H46, H49), 7.50 – 7.46 (2H, m, H47, H48), 5.16 – 5.07 (8H, m, H2, H6, H10, H14, H18, H22, H26, H30), 3.48 (2H, d, *J* = 6.1 Hz, H1), 3.23 (2H, t, *J* = 7.0 Hz, H34), 2.30 (3H, s, H53), 2.07 – 1.95 (30H, m, H4, H5, H8, H9, H12, H13, H16, H17, H20, H21, H24, H25, H28, H29, H32), 1.80 (3H, d, *J* = 0.9 Hz, H35), 1.70 – 1.67 (2H, m, H33), 1.60 – 1.56 (39H, m, H36, H37, H38, H39, H40, H41, H42, 2 x *t*Bu) ppm.

^{13}C-NMR (101 MHz, $CDCl_3$): δ 152.1, 151.7, 142.8, 142.4, 136.3, 135.3, 135.1, 135.0, 134.8, 133.4, 130.7, 127.3, 126.7, 126.6, 126.5, 126.4, 125.7, 124.6, 124.43, 124.38, 124.2, 121.3, 121.23, 121.18, 83.6, 51.0, 39.90, 39.88, 39.81, 39.76, 36.6, 29.9, 27.9, 27.8, 27.1, 26.94, 26.90, 26.88, 26.86, 26.8, 16.6, 16.17, 16.15, 16.1, 15.9, 12.9 ppm.

*Due to the close equivalence of multiple carbons, several signals overlap.

IR (neat, cm^{-1}): 2976, 2920, 2851, 2093, 1759, 1500, 1370, 1275, 1241, 1139, 1101, 885, 772, 747.

Rf : 0.74 (10% EtOAc in hexane)

CoQ$_{10}$-ω-azide (28)

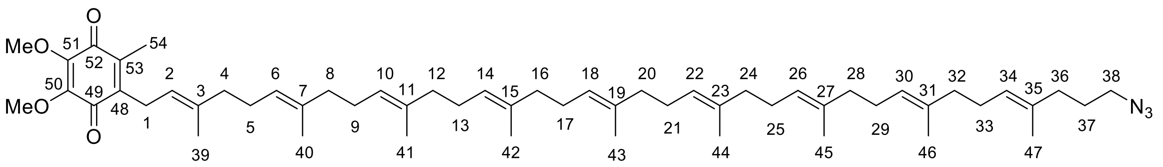

CoQ10-ω-azide (**28**) was synthesized from **26** according to general procedure for Boc deprotection and quinol oxidation described above. Flash chromatography (10:90 EtOAc/hexane) afforded CoQ10-ω-azide (**94**) as a viscous yellow oil (88 mg, 0.101 mmol, 95%).

^{1}H-NMR (400 MHz, $CDCl_3$): δ 5.16 – 5.04 (8H, m, H6, H10, H14, H18, H22, H26, H30, H34), 4.96 – 4.92 (1H, m, H2), 3.99 (3H, s, MeO), 3.98 (3H, s, MeO), 3.23 (2H, t, *J* = 7.0 Hz, H38), 3.18 (2H, d, *J* = 7.1 Hz, H1), 2.09 – 1.96 (37H, m, H4, H5, H8, H9, H12, H13, H16, H17, H20, H21, H24, H25, H28, H29, H32, H33, H36, H54), 1.74 (3H, d, *J* = 0.8 Hz, H39), 1.70 – 1.67 (2H, m, H37), 1.61 – 1.57 (24H, d, *J* = 0.6 Hz, H40, H41, H42, H43, H44, H45, H46, H47) ppm.

^{13}C-NMR (101 MHz, $CDCl_3$): δ 184.9, 184.0, 144.6, 144.4, 141.8, 139.0, 137.8, 135.4, 135.14, 135.08, 135.07, 135.0, 134.8, 133.4, 125.8, 125.7, 124.6, 124.44, 124.42, 124.41, 124.3, 124.0, 119.0, 77.4, 61.3, 51.1, 39.90, 39.88, 39.87, 39.8, 36.7, 27.1, 26.88, 26.86, 26.8, 26.7, 25.5, 16.5, 16.2, 16.13, 15.9, 12.1 ppm.

*Due to the close equivalence of multiple carbons, several signals overlap.

HRMS–ESI (*m/z*): $[M+H]^+$ calculated 886.6432; found 886.6423.

IR (neat, cm^{-1}): 2961, 2913, 2850, 2097. 1646, 1610, 1448, 1383, 1262, 1209, 1146, 1101, 1018, 874, 796, 742.

Rf = 0.74 (20:80 EtOAc:hexane).

MK9-ω-azide (29)

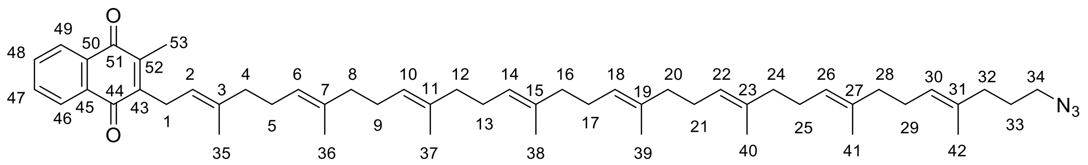

MK9-ω-azide (**29**) was synthesized from **27** according to general procedure for Boc deprotection and quinol oxidation described above. Flash chromatography (5:95 EtOAc:hexane) afforded MK9-ω-azide (**29**) as a yellow oil (46.5 mg, 0.058 mmol, 74%).

^{1}H-NMR (400 MHz, $CDCl_3$): δ 8.09 – 8.06 (2H, m, H46, H49), 7.70 – 7.67 (2H, m, H47, H48), 5.15 – 5.00 (H8, m, H2, H6, H10, H14, H18, H22, H26, H30), 3.37 (2H, d, *J* = 6.9 Hz, H1), 3.23 (2H, t, *J* = 7.0 Hz, H34), 2.19 (3H, s, H53), 2.12 – 1.92 (30H, m, H4, H5, H8, H9, H12, H13, H16, H17, H20, H21, H24, H25, H28, H29, H32), 1.79 (3H, d, *J* = 1.1 Hz, H35), 1.70 – 1.66 (2H, m, H33), 1.65 – 1.55 (21H, m, H36, H37, H38, H39, H40, H41, H42) ppm.

^{13}C-NMR (101 MHz, $CDCl_3$): δ 186.0, 185.1, 146.8, 143.9, 138.2, 135.8, 135.52, 135.51, 135.48, 135.3, 133.91, 133.85, 132.79, 132.75, 126.9, 126.8, 126.2, 125.0, 124.88, 124.85, 124.7, 124.4, 119.7, 51.5, 40.34, 40.32, 40.28, 40.2, 37.1, 27.6, 27.32, 27.30, 27.26, 27.2, 27.1, 26.6, 17.0, 16.62, 16.59, 16.57, 16.4, 13.3 ppm.

*Due to the close equivalence of multiple carbons, several signals overlap.

HRMS–ESI (*m/z*): $[M–N_2+H]^+$ calculated 758.5871 ; found 758.5826.

IR (neat, cm^{-1}): 2915, 2860, 2093, 1661, 1597, 1443, 1293, 1258, 714.

Rf : 0.93 (20% EtOAc in hexane)

Synthesis of Alkynes Required for CuAAC Reactions

Synthesis of NBD-alkyne (39)

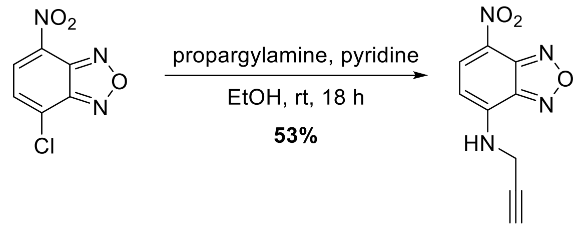

NBD-alkyne (39)

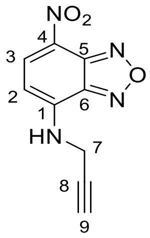

4-Propargylamine-7-nitrobenzofuran was prepared according to literature.[6] 4-Chloro-7-nitrobenzofuran (100 mg, 0.50 mmol) was dissolved in EtOH (5 mL). Propargylamine (80 µL, 1.25 mmol) and pyridine (8 µL, 0.1 mmol) was added. The reaction mixture was stirred at room temperature for 18 h, at which point TLC indicated reaction completion and the solvent was removed in vacuo. Flash chromatography (10:90 – 30:70 EtOAc/hexane) afforded NBD-alkyne (**39**) (58 mg, 0.27 mmol, 53%) as an orange crystalline solid. NMR data matched that of the literature.[6]

^{1}H-NMR (400 MHz; DMSO-d_6): δ 9.64 (1H, s, NH), 8.62 (1H, d, J = 8.8 Hz, H2), 6.48 (1H, d, J = 8.9 Hz, H3), 4.34 (2H, s, H7), 3.36 (1H, t, J = 2.5 Hz, H9) ppm.

^{13}C NMR (151 MHz; DMSO): δ 144.4, 144.1, 137.6, 122.2, 100.3, 78.6, 75.1, 32.4 ppm.

HRMS–ESI (*m/z*): $[M+H]^+$ calculated 219.0513; found 219.2145.

Synthesos of DNB-alkyne (40)

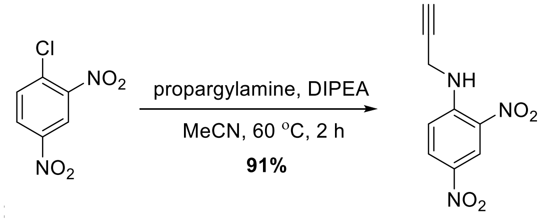

DNB-alkyne (40)

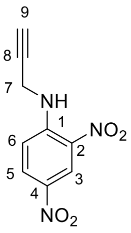

DNB-alkyne (**40**) was prepared according to a modified literature procedure.[7] 1-Chloro-2,4-dinitrobenzene (100 mg, 0.49 mmol) was dissolved in dry MeCN (2 mL) and DIPEA (0.25 mL, 1.47 mmol) and propargylamine (64 μL, 0.98 mmol) was added dropwise. The reaction mixture was heated to 60 °C and stirred under argon for 2 h, at which point TLC indicated reaction completion. The reaction was cooled to ambient temperature and H_2O (3 drops) added to form a precipitate. This was filtered and dried *in vacuo* to yield DNB-alkyne (**40**) (98.5 mg, 0.44 mmol, 91%) as bright yellow crystals. No further purification was necessary. NMR data matched that of literature.[7]

^{1}H-NMR (400 MHz, DMSO-d_6): δ 9.09 – 9.02 (1H, m, NH), 8.87 (1H, d, *J* = 2.7 Hz, H3), 8.38 (1H, dd, *J* = 9.5, 2.6 Hz, H5), 7.25 (1H, d, *J* = 9.6 Hz, H6), 4.35 (2H, dd, *J* = 5.8, 2.3 Hz, H7), 3.32 (1H, t, *J* = 2.4 Hz, H9) ppm.

^{13}C-NMR (101 MHz, DMSO-d$_6$): δ 147.3, 135.6, 130.5, 130.0, 123.4, 115.7, 79.3, 74.8, 32.4 ppm.

CHNS calculated C 48.88, H 3.19, N 19.00; found C 49.98, H 3.09, N 18.23.

Synthesis of BODIPY-alkyne (41)

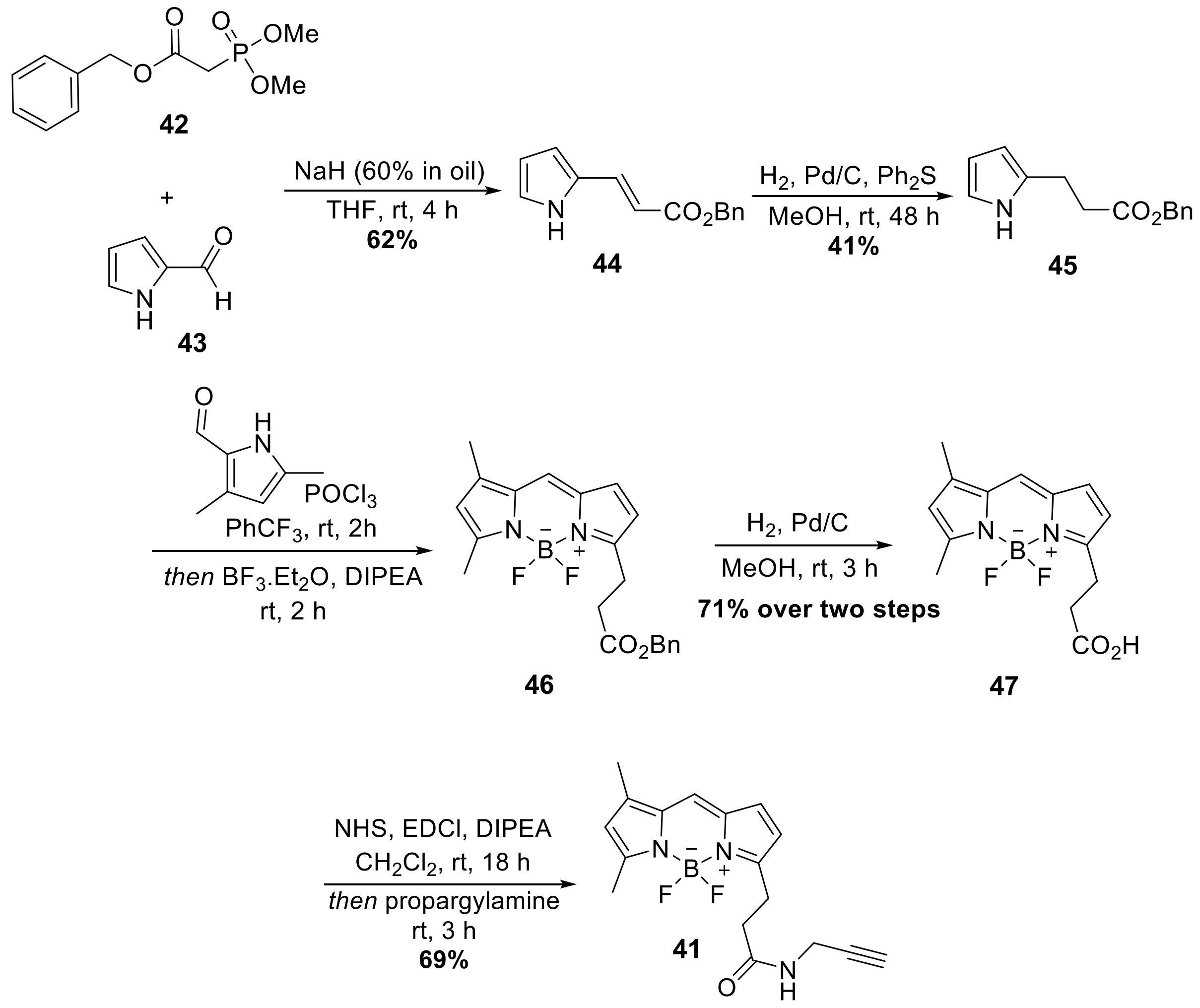

Benzyl (2*E*)-3-(1*H*-pyrrol-2-yl)prop-2-enoate (44)

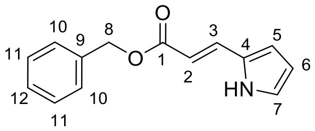

Benzyl (2*E*)-3-(1*H*-pyrrol-2-yl)prop-2-enoate (**44**) was prepared according to literature.[8] Benzyl (dimethoxyphosphoryl)acetate (**42**) (2.29 g, 8.86 mmol) was dissolved in dry THF (18 mL) and cooled to 0 °C. NaH (60 wt% in mineral oil, 276 mg, 11.5 mmol) was added in 5 portions over 10 min. This solution was stirred at 0 °C for 5 min. A solution of 1*H*-pyrrole-2-carbaldehyde (**43**) (927 mg, 9.76 mmol) in dry THF (2 mL) was added dropwise over 5 min. The reaction mixture was warmed to ambient temperature and stirred under argon for 3 h. The reaction mixture was cooled to 0 °C and quenched with citric acid (5% v/v aq, 5 mL). This was diluted with H_2O (15 mL) and extracted with EtOAc (40 mL). The organic layer was washed with brine (20 mL) and H_2O (20 mL) then dried over Na_2SO_4 and the solvent removed under reduced pressure. Flash chromatography (30:70 EtOAc:hexane) afforded benzyl (2*E*)-3-(1*H*-pyrrol-2-yl)prop-2-enoate (**44**) (1.26 g, 5.52 mmol, 62%) as a colourless solid. NMR data matched that of literature.[8]

^{1}H-NMR (400 MHz, DMSO-d$_6$): δ 11.53 (1H, s, NH), 7.50 (1H, d, *J* = 15.9 Hz, H3), 7.41 – 7.31 (5H, m, H10. H11, H12), 7.04 (1H, d, *J* = 1.3 Hz, H7), 6.61 – 6.57 (1H, m, H5), 6.26 (1H, d, *J* = 15.8 Hz, H2), 6.18 – 6.14 (1H, m, H6), 5.17 (2H, s, 2H, H8) ppm.

^{13}C-NMR (101 MHz, DMSO-d$_6$): δ 166.7, 136.6, 135.1, 128.4, 128.0, 127.9, 123.6, 115.1, 110.1, 109.7, 65.1 ppm.

Benzyl 3-(1*H*-pyrrol-2-yl)propanoate (45)

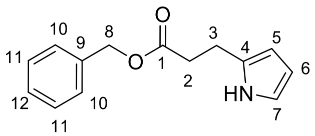

Benzyl 3-(1*H*-pyrrol-2-yl)propanoate (**45**) was prepared according to an adapted literature procedure.[8] Benzyl (2*E*)-3-(1*H*-pyrrol-2-yl)prop-2-enoate (**44**) (1.23 g, 5.41 mmol) was dissolved in MeOH (18 mL) and purged with argon. Pd/C (10% wt, 112 mg, 0.105 mmol) and diphenyl sulfide (4.5 µL, 0.027 mmol) added, followed by a H_2 ballon. The reaction mixture was stirred at ambient temperature for 48 h. The reaction mixture was filtered through celite and washed with MeOH (3 x 15 mL), the filtrate was concentrated under reduced pressure. Flash chromatography (10:90 EtOAc:hexane) afforded benzyl 3-(1*H*-pyrrol-2-yl)propanoate (**45**) (510 mg, 2.22 mmol, 41%) as a colourless solid. NMR data matched that of literature.[8]

^{1}H-NMR (400 MHz, DMSO-d_6): δ 10.52 (1H, s, NH), 7.39 – 7.31 (5H, m, H10, H11, H12), 6.58 – 6.54 (1H, m, H7), 5.88 – 5.85 (1H, m, H5), 5.74 – 5.71 (1H, m, H6), 5.09 (2H, s, H8), 2.81 (2H, t, *J* = 7.6 Hz, H3), 2.67 – 2.63 (2H, m, H2) ppm.

^{13}C-NMR (101 MHz, DMSO-d_6): δ 172.2, 136.2, 129.9, 128.4, 128.0, 127.9, 116.3, 107.1, 104.5, 65.4, 33.9, 22.7 ppm.

HRMS–ESI (*m/z*): $[2M+Na]^+$ calculated 481.2098; found 481.2153.

BODIPY-acid (47)

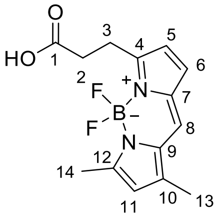

BODIPY-acid (**47**) was prepared according to literature.[8] Benzyl 3-(1*H*-pyrrol-2-yl)propanoate (**45**) (500 mg, 2.22 mmol) and 3,5-dimethyl-1*H*-pyrrole-2-carbaldehyde (287 mg, 2.33 mmol) were dissolved in anhydrous $PhCF_3$ (10 mL). $POCl_3$ (0.22 mL, 2.33 mmol) was added dropwise over 10 min. The reaction mixture was stirred at ambient temperature under argon for 2.5 h, at which point TLC indicated reaction completion. The reaction mixture was then cooled to 0 °C and $BF_3.Et_2O$ (1.1 mL, 8.88 mmol) added, followed by DIPEA (1.5 mL, 8.88 mmol) dropwise over 30 min. The reaction mixture was warmed to ambient temperature and stirred under argon for 1.75 h until TLC indicated reaction completion. The reaction mixture was then cooled to 0 °C and quenched with $NaHCO_3$ (5% wt Aq., 10 mL), diluted with EtOAc and filtered through celite, the celite was washed with EtOAc (2 x 10 mL). The filtrate was washed with brine (20 mL), H_2O (20 mL), dried over Na_2SO_4 and solvent removed under reduced pressure. Flash chromatography (0:100 – 20:80 EtOAc:hexane) afforded BODIPY-benzyl ester (**46**) as a red/green crystalline solid (629 mg, 1.64 mmol). This was dissolved in MeOH (6 mL) and Pd/C (10 wt., 62 mg) added. A H_2 ballon was attached, and the reaction mixture was stirred at ambient temperature for 2.5 h, at which point TLC indicated reaction completion. The reaction mixture was filtered through celite and washed with MeOH (3 x 5 mL) and EtOAc (3 x 5 mL). The filtrate was concentrated *in vacuo* to give BODIPY-acid (**47**) as a red-green solid (460 mg, 15.7 mmol, 71% over 2 steps). NMR data matched that of literature.[8]

^{1}H-NMR (400 MHz; DMSO-d$_6$): δ 7.70 (1H, s, H8), 7.09 (1H, d, *J* = 4.1 Hz, H6), 6.38 (1H, d, *J* = 4.1 Hz, H5), 6.31 (1H, s, H11), 3.07 (2H, t, *J* = 7.7 Hz, H3), 2.64 (2H, t, *J* = 7.7 Hz, H2), 2.47 (3H, s, H14), 2.26 (3H, s, H13) ppm.

^{13}C-NMR (101 MHz, DMSO-d$_6$): δ 173.4, 159.5, 156.9, 144.3, 134.6, 133.0, 128.8, 125.5, 120.4, 116.6, 32.3, 23.5, 14.5, 11.0 ppm.

LRMS–ESI (*m/z*): $[M–H]^–$ calculated 291.1; found 291.3.

BODIPY-alkyne (41)

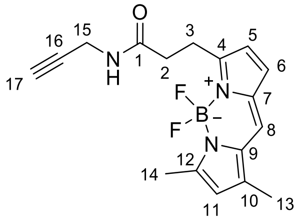

BODIPY-COOH (**47**) (100 mg, 0.34 mmol), N-hydroxyl succinimide (43 mg, 0.37 mmol) and EDC.HCl (71 mg, 0.37 mmol) were dissolved in CH_2Cl_2 (4 mL) and stirred at ambient temperature for 18 h. Propargylamine (32 µL, 0.51 mmol) was then added and the reaction mixture stirred at ambient temperature for 3 h, at which point TLC indicated reaction completion. The reaction mixture was diluted with CH_2Cl_2 (20 mL) and washed with brine (25 mL) and water (25 mL), the organic layer dried over Na_2SO_4 and the solvent removed under reduced pressure. Flash chromatography (30:70 – 50:50 EtOAc:hexane) afforded BODIPY-alkyne (**137**) (76.9 mg, 0.23 mmol, 69%) as a bright red solid.

^{1}H-NMR (400 MHz, $CDCl_3$): δ 7.11 (1H, s, H8), 6.90 (1H, d, *J* = 3.9 Hz, H6), 6.31 (1H, d, *J* = 4.0 Hz, H5), 6.15 (1H, s, H11), 5.86 – 5.83 (1H, m, NH), 4.04 (1H, d, *J* = 2.6 Hz, H15), 4.03

(1H, d, J = 2.6 Hz, H15') 3.30 (2H, t, J = 7.5 Hz, H3), 2.67 (2H, t, J = 7.6 Hz, H2), 2.59 (3H, s, H14), 2.28 (3H, s, H13), 2.21 (1H, t, J = 2.6 Hz, H17) ppm.

^{13}C-NMR (151 MHz, $CDCl_3$): δ 71.5, 160.6, 157.1, 144.1, 135.3, 133.6, 128.4, 124.0, 120.6, 117.6, 79.6, 71.6, 36.0, 29.3, 24.9, 15.1, 11.5 ppm.

^{19}F-NMR (376 MHz, $CDCl_3$): δ – 144.49 (d, J = 33.5 Hz), –144.67 (d, J = 33.5 Hz) ppm.

HRMS–ESI (*m/z*): $[M+Na]^+$ calculated 352.1403 ; found 352.1461.

IR (neat, cm^{-1}): 3286, 2920, 2853, 1653, 1603, 1532, 1485, 1424, 1241, 1194, 1139, 1083, 1057, 967, 826, 716.

Rf : 0.46 (40% EtOAc in hexane)

Synthesis of Biotin-alkyne (48)

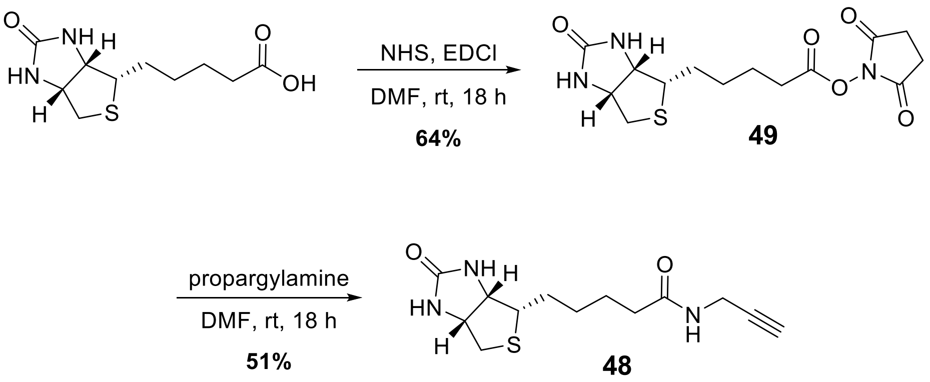

Biotin-NHS ester (49)

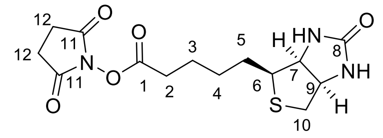

Biotin-NHS ester (**49**) was prepared according to literature.[13] Biotin (500 mg, 2.05 mmol) was dissolved in DMF (20 mL), N-hydroxy-succinimide (353 mg, 3.07 mmol) and EDCl (629 mg, 3.28 mmol) was added. The reaction mixture was stirred at room temperature for 18 h. The solvent was removed and the resulting white solid was washing MeOH (3 x 15 mL) and Et_2O (3 x 15 mL) and dried *in vacuo* to afford biotin-NHS ester (**48**) (446 mg, 1.31, 64%). No further purification was required. NMR data matched that of literature.[13]

^{1}H NMR: (400 MHz, DMSO-d_6): δ 6.42 (1H, s, NH), 6.36 (1H, s, NH), 4.30 (H1, dd, *J* = 7.4, 5.3 Hz, H7), 4.15 (1H, ddd, *J* = 7.4, 4.6, 1.7 Hz, H9), 3.13 – 3.08 (1H, m, H6), 2.89 – 2.73 (5H, m, H10, H12), 2.67 (2H, t, *J* = 7.4 Hz, H2), 2.58 (1H, d, *J* = 12.5 Hz, H10’), 1.68 – 1.38 (6H, m, H3, H4, H5) ppm.

^{13}C-NMR (101 MHz, DMSO-d_6): δ 170.3, 168.9, 162.6, 61.0, 59.2, 55.2, 30.0, 27.8, 27.6, 25.4, 24.3 ppm.

HRMS–ESI (*m/z*): $[M+H]^+$ calculated 342.1123; found 342.1124.

Biotin-alkyne (48)

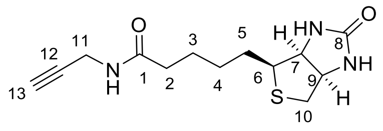

Biotin-alkyne (**48**) was prepared according to literature.[9] Biotin-NHS (**49**) (25 mg, 0.07 mmol) was dissolved in DMF (1 mL), propargyl amine (9 µL, 0.14 mmol) was added. The reaction mixture was stirred at ambient temperature for 18 h and the reaction mixture was concentrated under reduced pressure. Flash chromatography (10:90 MeOH:CH_2Cl_2) afforded biotin propargyl amide (**102**) (10.1 mg, 0.036 mmol, 51%) as a colourless crystalline solid.[9]

^{1}H-NMR (400 MHz, DMSO-d_6): δ 8.22 (t, *J* = 0.4 Hz, NH), 6.42 (s, NH), 6.36 (s, NH), 4.31 – 4.29 (1H, m, H7), 4.13 – 4.11 (1H, m, H9), 3.83 (2H, d, *J* = 1.7 Hz, H11), 3.11 – 3.07 (2H, m, H6, H13), 2.82 (1H, dd, *J* = 12.1, 4.4 Hz, H10), 2.57 (1H, d, *J* = 12.6 Hz, H10'), 2.08 (2H, t, *J* = 6.9 Hz, H2), 1.64 – 1.20 (6H, m, H3, H4, H5) ppm.

^{13}C-NMR (101 MHz, DMSO-d_6): δ 171.8, 162.7, 81.4, 72.8, 61.0, 59.2, 55.4, 34.9, 28.2, 28.0, 27.7, 25.1 ppm.

171.7, 162.6, 81.3, 72.7, 60.9, 59.1, 55.3, 34.8, 28.1, 27.9, 27.6, 25.1, 25.0

LRMS–ESI (*m/z*): $[M+Na]^+$ calculated 304.1; found 304.3.

Method A for CuAAC Reactions

CuAAC reactions were carried out according to a modified literature procedure.[10] PPQ-ω-azide (1 equiv.) and alkyne (1.1 equiv.) were dissolved in DMF (0.1 M). A solution of $Cu(SO_4).5H_2O$ (1 equiv.) and sodium ascorbate (2 equiv.) in H_2O (1mL) was prepared. A portion of this solution (0.1 mL) was added to BTTAA (0.6 equiv.) to give a colourless solution.

This solution was added to the reaction mixture and heated to 60 °C. The reaction mixture was stirred at 60 °C for 24 h. The reaction mixture was cooled to room temperature, solvent removed *in vacuo* and quenched with H_2O (2 mL). This was extracted with EtOAc (3 x 2 mL), the combine organic extracts were dried over Na_2SO_4 and solvent removed in vacuo. Flash chromatography afforded the desired product.

Method B for CuAAC Reactions

CuAAC reactions were carried out according to a modified literature procedure.[10] PPQ-ω-azide (1 equiv.) and alkyne (2 equiv.) were dissolved in DMF (0.3 mL). A solution of $Cu(SO_4).5H_2O$ (1 equiv.) and sodium ascorbate (2 equiv.) in H_2O (1mL) was prepared. A portion of this solution (0.1 mL) was added to BTTAA (0.6 equiv.) to give a colourless solution. This solution was added to the reaction mixture and heated to 60 °C. The reaction mixture was stirred at 60 °C for 24 h. The reaction mixture was cooled to room temperature, solvent removed *in vacuo*and quenched with H_2O (2 mL). This was extracted with EtOAc (3 x 2 mL), the combine organic extracts were dried over Na_2SO_4 and solvent removed in vacuo. Flash chromatography afforded the desired product.

CoQ10-ω-NBD (30)

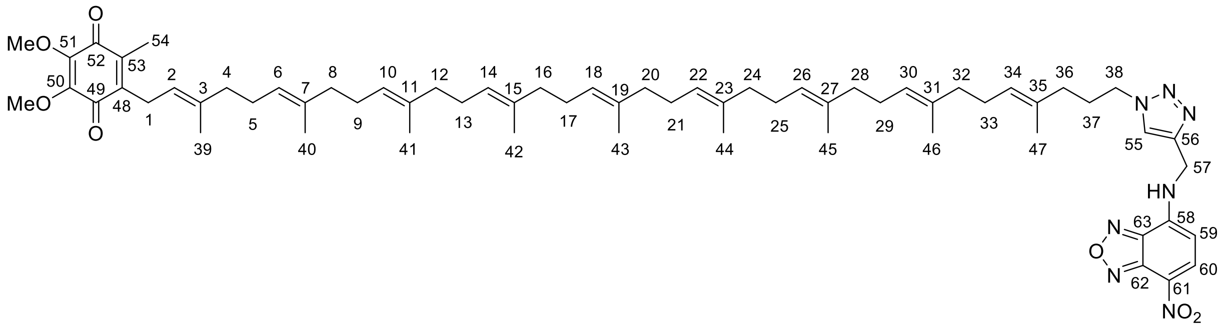

CoQ10-ω-NBD (**30**) was synthesised according to method A. Flash chromatography (20:80 – 50:50 EtOAc:hexane) afforded CoQ10-ω-NBD (**30**) (12.3 mg, 0.011 mmol, 95%).

^{1}H NMR (400 MHz; $CDCl_3$): δ 8.49 (1H, d, *J* = 8.5 Hz, H60), 7.58 (1H, s, H55), 6.37 (1H, d, *J* = 8.6 Hz, H41), 5.13 – 5.09 (8H, m, H6, H10, H14, H18, H22, H26, H30, H34), 4.93 (1H, t, *J* = 1.1 Hz, H2), 4.81 (2H, s, H57), 4.34 (2H, t, *J* = 7.1 Hz, H38), 4.00 (3H, s, OMe) 3.98 (3H, s, OMe) 3.18 (2H, d, *J* = 6.9 Hz, H1), 2.09 – 1.95 (39H, m, H4, H5, H8, H9, H12, H13, H16, H17, H20, H21, H24, H25, H28, H29, H32, H33, H36, H37, H54), 1.73 (3H, d, *J* = 0.9 Hz, H39), 1.64 – 1.58 (24 H, m, H40, H41, H42, H43, H44, H45, H46, H47) ppm.

^{13}C NMR (101 MHz; $CDCl_3$): δ 144.4, 144.3, 143.0, 141.9, 141.7, 138.9, 137.6, 136.0, 135.2, 135.0, 134.92, 134.89, 134.8, 134.6, 132.5, 126.3, 125.1, 124.5, 124.32, 124.28, 124.26, 124.2, 123.8, 121.9, 118.9, 99.5, 61.1, 50.1, 39.8, 39.6, 39.5, 36.2, 29.7, 29.7, 28.2, 26.7, 26.6, 26.5, 25.3, 16.3, 16.0, 16.0, 15.8, 11.9 ppm.

*Due to the close equivalence of multiple carbons, several signals overlap.

HRMS–ESI (*m/z*): $[M+H]^+$ calculated 1082.7053 ; found 1082.7040.

IR (neat, cm^{-1}): 2919, 2850, 1649, 1580, 1441, 1295, 1262, 1027, 811.

Rf = 0.16 (20% EtOAc in hexane).

MK-9-ω-NBD (31)

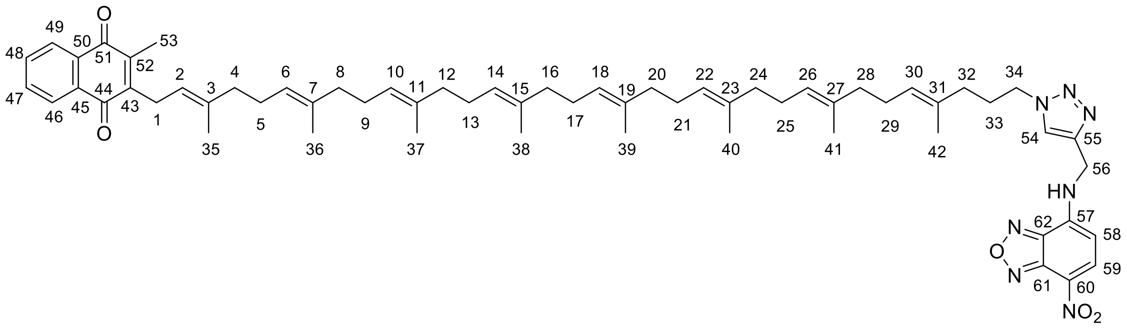

MK9-ω-NBD (**31**) was synthesised according to general method A. Flash chromatography (30:70 – 50:50 EtOAc:hexane) afforded MK9-ω- NBD (**31**) (12.3 mg, 0.0 mmol, 74%).

^{1}H-NMR (400 MHz, $CDCl_3$): δ 8.49 (1H, d, *J* = 8.5 Hz, H59), 8.09 – 8.06 (2H, m, H46, H49), 7.69 – 7.67 (2H, m, H47, H48), 7.57 (1H, s, H54), 6.81 (1H, s, NH), 6.36 (1H, d, *J* = 8.6 Hz, H58), 5.14 – 4.99 (8 H, m, H6, H10, H14, H18, H22, H26, H30, H34), 4.81 (2H, s, H55), 4.35 – 4.31 (2H, m, H34), 3.37 (2H, d, *J* = 6.9 Hz, H1), 2.18 (3H, s, H53), 2.08 – 1.91 (32H, m, H4, H5, H8, H9, H12, H13, H16, H17, H20, H21, H24, H25, H28, H29, H32, H33), 1.79 (3H, d, *J* = 1.2 Hz, H35), 1.60 – 1.56 (21 H, m, H36, H37, H38, H39, H40, H41, H42) ppm.

^{13}C-NMR (101 MHz, $CDCl_3$): δ 185.6, 184.7, 146.3, 144.4, 143.9, 143.5, 143.2, 142.0, 137.7, 136.2, 135.4, 135.2, 135.1, 135.0, 134.8, 133.5, 133.4, 132.7, 132.31, 132.27, 126.4, 126.3, 125.3, 124.6, 124.5, 124.42, 124.40, 124.3, 124.0, 122.0, 119.2, 77.2, 50.2, 39.9, 39.8, 39.71, 39.65, 36.3, 29.8, 28.3, 26.9, 26.80, 26.76, 26.7, 26.2, 16.6, 16.18, 16.15, 15.9, 12.8 ppm.

*Due to the close equivalence of multiple carbons, several signals overlap.

HRMS–ESI (*m/z*): $[M+H]^+$ calculated 1004.6371 ; found 1004.6357.

IR (neat, cm^{-1}): 2991, 2913, 1756, 1687, 1437, 1241, 1139, 1019, 952, 885, 697.

Rf : 0.08 (20% EtOAc in hexane).

CoQ10-ω-DNB (32)

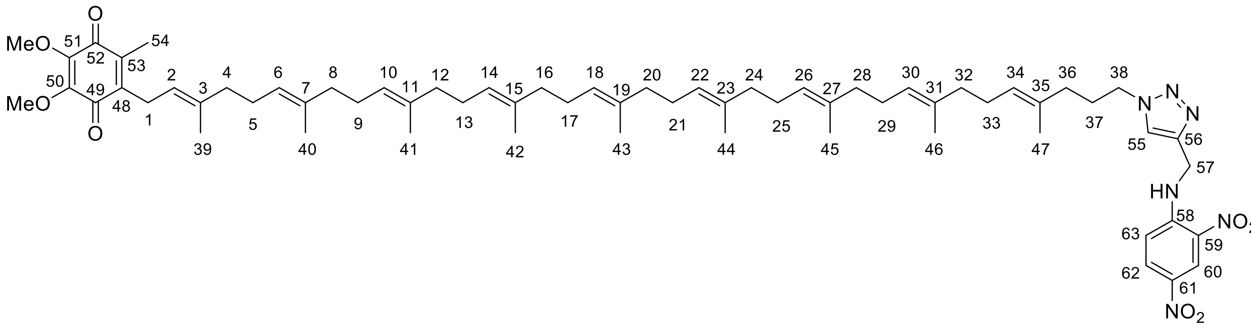

CoQ10-ω-DNB (**32**) was synthesised according to general method B. Flash chromatography (30:70 EtOAc:hexane) afforded CoQ10-ω-DNB (**32**) as a bright yellow oil (4.3 mg, 0.0040 mmol, 68%).

^{1}H-NMR (400 MHz; $CDCl_3$): δ 9.15 (1H, d, *J* = 2.6 Hz, H60), 8.95 – 8.92 (1H, m, NH), 8.29 (1H, ddd, *J* = 9.5, 2.7, 0.7 Hz, H62), 7.51 (1H, s, H55), 7.15 (1H, d, *J* = 9.5 Hz, H63), 5.12 – 5.04 (8H, m, H6, H10, H14, H18, H22, H26, H30, H34), 4.95 – 4.91 (1H, m, H2), 4.76 (2H, d, *J* = 5.6 Hz, H57), 4.31 (2H, t, *J* = 7.2 Hz, H38), 3.99 (3H, s, MeO), 3.98 (3H, s, MeO), 3.18 (2H, d, *J* = 6.8 Hz, H1), 2.07 – 1.95 (41H, m, H4, H5, H8, H9, H12, H13, H16, H17, H20, H21, H24, H25, H28, H29, H32, H33, H37, H54), 1.73 (3H, d, *J* = 1.2 Hz, H39), 1.60 – 1.57 (27H, m, H40, H41, H42, H43, H44, H45, H46, H47) ppm.

^{13}C-NMR (101 MHz, $CDCl_3$): δ 184.8, 183.9, 154.7, 147.9, 144.4, 144.2, 142.7, 141.7, 138.8, 137.6, 136.7, 135.3, 135.00, 134.95, 134.92, 134.86, 134.6, 132.57, 132.55, 131.0, 130.4, 129.9, 126.3, 124.5, 124.31, 124.27, 124.25, 124.24, 124.15, 124.1, 123.8, 121.7, 118.8, 115.9, 114.4, 61.2, 61.1, 50.0, 39.8, 39.73, 39.72, 39.6, 39.3, 36.2, 29.7, 28.2, 26.7, 26.70, 26.69, 26.6, 26.5, 25.3, 16.4, 16.04, 15.99, 15.8, 12.0 ppm.

*Due to the close equivalence of multiple carbons, several signals overlap.

HRMS–ESI (*m/z*): $[M+H]^+$ calculated 1085.7050; found 1085.7078.

IR (neat, cm^{-1}): 2919, 2850, 1648, 1612, 1590, 1523, 1429, 1381, 1334, 1262, 1204, 1049, 923, 833, 755.

Rf : 0.27 (30% EtOAc in hexane).

MK9-ω-DNB (33)

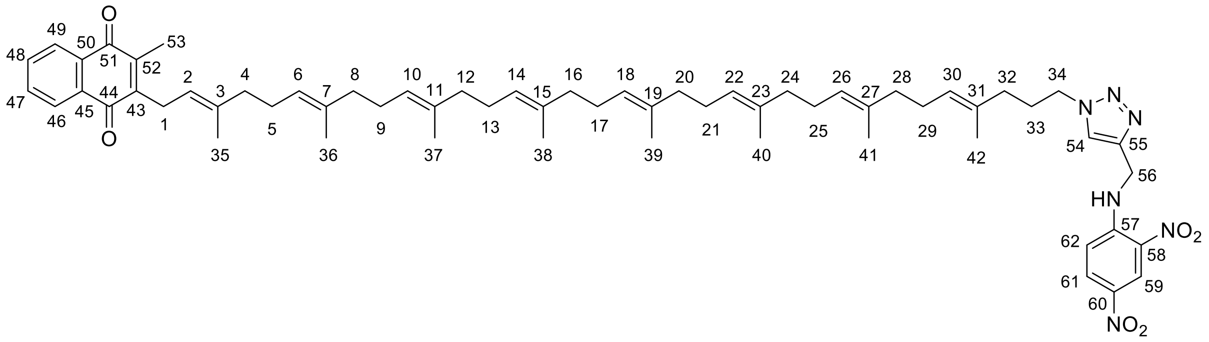

MK9-ω-DNB (**33**) was synthesised according to general method B. Flash chromatography (10:90 – 30:70 EtOAc:hexane) afforded MK9-ω-DNB (**33**) as a bright yellow oil (5.1 mg, 0.0051 mmol, 94%).

^{1}H-NMR (600 MHz, $CDCl_3$): δ 9.15 (1H, d, *J* = 2.6 Hz, H59), 8.94 – 8.92 (1H, m, NH), 8.29 (1H, dd, *J* = 9.6, 2.5 Hz, H61), 8.09 – 8.07 (2H, m, H46, H49), 7.69 – 7.68 (2H, m, H47, H48), 7.51 (1H, s, H54), 7.15 (1H, d, *J* = 9.4 Hz, H62), 5.14 – 5.03 (7H, m, H6, H10, H14, H18, H22, H26, H30), 5.03 – 5.00 (1H, m, H2), 4.76 (2H, d, *J* = 5.5 Hz, H56), 4.31 (2H, t, *J* = 7.0 Hz, H34), 3.37 (2H, d, *J* = 6.4 Hz, H1), 2.19 – 2.18 (3H, m, H53), 2.07 – 1.95 (32H, m, H4, H5, H8, H9,H12, H13, H16, H17, H20, H21, H24, H25, H28, H29, H32, H33), 1.79 (3H, s, H35), 1.59 – 1.55 (21H, m, H36, H37, H38, H39, H40, H41, H42) ppm.

^{13}C-NMR (151 MHz, $CDCl_3$): δ 185.6, 152.3, 148.0, 146.3, 142.9, 136.9, 135.4, 135.07, 135.06, 135.00, 134.8, 133.5, 133.4, 132.7, 130.6, 126.5, 126.4, 126.3, 124.6, 124.5, 124.42, 124.41,

124.30, 124.28, 124.0, 121.9, 119.2, 114.5, 50.2, 39.90, 39.89, 39.88, 39.7, 39.5, 36.4, 29.9, 28.3, 26.9, 26.82, 26.78, 26.7, 26.2, 23.2, 16.6, 16.19, 16.16, 16.1, 15.9, 12.8 ppm.

*Due to the close equivalence of multiple carbons, several signals overlap.

IR (neat, cm^{-1}): 3364, 2919, 2851, 1659, 1618, 1590, 1523, 1424, 1333, 1293, 1148, 1131, 1047, 971, 923, 833, 764, 744, 714.

Rf : 0.45 (50% EtOAc in hexane).

CoQ10-ω-BODIPY (91)

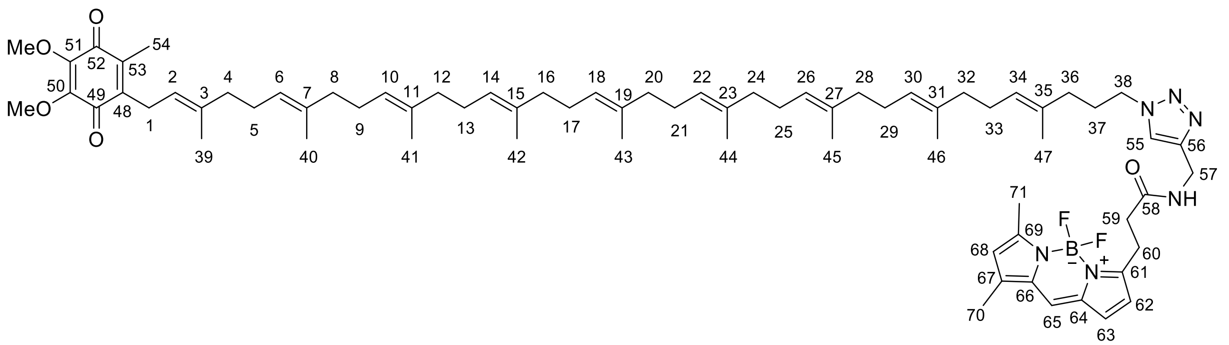

CoQ10-ω-BODIPY (**91**) was synthesised according to general method B. Flash chromatography (20:80 – 40:60 EtOAc:hexane) afforded CoQ10-ω-BODIPY (**91**) (10.1 mg, 0.0085 mmol, 91%).

^{1}H-NMR (400 MHz, $CDCl_3$): δ 7.46 (1H, s, H55), 7.07 (1H, s, H65), 6.84 (1H, d, *J* = 3.9 Hz, H63), 6.23 (1H, d, *J* = 4.0 Hz, H62), 6.11 (1H, s, H68), 5.16 – 5.04 (8H, m, H6, H10, H14, H18, H22, H26, H30, H34), 4.95 – 4.91 (1H, m, H2), 4.48 (2H, d, *J* = 5.8 Hz, H40), 4.25 (2H, t, *J* = 7.0 Hz, H38), 3.99 (3H, s, MeO), 3.98 (3H, s, MeO), 3.27 (3H, t, *J* = 7.6 Hz, H60), 3.18 (2H, d, *J* = 7.0 Hz, H1), 2.64 (2H, t, *J* = 7.6 Hz, H59), 2.55 (3H, s, H71), 2.25 (3H, s, H70), 2.07 – 1.95 (39H, m, H4, H5, H8, H9, H12, H16, H17, H20, H21, H24, H25, H28, H29, H32, H33, H36, H37,

H54), 1.74 (3H, d, J = 1.0 Hz, H39), 1.60 – 1.57 (24H, m, H40, H41, H42, H43, H44, H45, H46, H47) ppm.

^{13}C-NMR (151 MHz, $CDCl_3$): δ 184.8, 183.9, 171.8, 160.5, 157.1, 144.72, 144.71, 144.4, 144.2, 141.7, 138.9, 137.6, 135.3, 135.24, 135.22, 135.01, 134.96, 134.9, 134.7, 133.3, 132.7, 128.03, 128.02, 126.0, 124.5, 124.3, 124.24, 124.15, 123.84, 123.77, 123.76, 122.2, 120.52, 120.50, 120.48, 118.9, 117.24, 117.23, 117.21, 61.2, 61.1, 49.8, 39.8, 39.7, 39.6, 36.3, 35.82, 35.81, 35.1, 31.9, 29.7, 29.7, 29.4, 28.3, 26.7, 26.7, 26.6, 25.3, 24.8, 22.7, 16.4, 16.1, 15.8, 15.0, 14.1, 12.0, 11.3 ppm.

*Due to the close equivalence of multiple carbons, several signals overlap.

HRMS–ESI (*m/z*): $[M+H]^+$ calculated 1193.8124; found 1193.8062.

IR (neat, cm^{-1}): 2917, 2848, 1649, 1605, 1528, 1437, 1260, 1172, 1131, 1083, 1062, 1010, 796, 744, 669.

Rf : 0.06 (40% EtOAc in hexane).

MK9-ω-BODIPY (35)

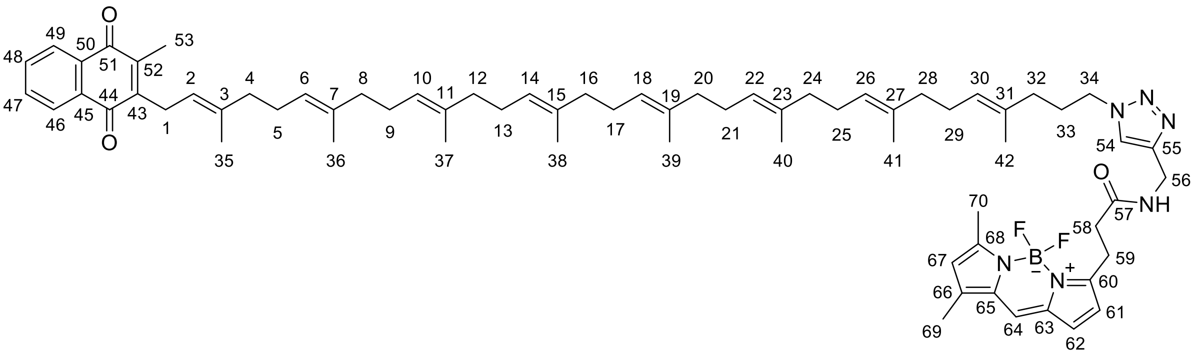

MK9-ω-BODIPY (**35**) was synthesised according to general method B. Flash chromatography (20:80 – 40:60 EtOAc:hexane) afforded MK9-ω-BODIPY (**35**) (4.5 mg, 0.0085 mmol, 54%).

^{1}H-NMR (400 MHz, $CDCl_3$): δ 8.09 – 8.06 (2H, m, H46, H49), 7.70 – 7.67 (2H, m, H47, H48), 7.07 (1H, s, H54), 7.07 (1H, s, H64), 6.84 (1H, d, *J* = 4.0 Hz, H62), 6.23 – 6.20 (2H, m, H62, NH), 6.12 (1H, s, H67), 5.14 – 5.00 (8H, m, H2, H6, H10, H14, H18, H22, H26, H30), 4.48 (2H, d, *J* = 5.8 Hz, H56), 4.25 (2H, t, J = 6.1 Hz, H34), 3.37 (2H, d, *J* = 6.9 Hz, H1), 3.27 (2H, t, *J* = 7.4 Hz, H59), 2.64 (2H, t, *J* = 7.6 Hz, H58), 2.55 (3H, s, H70), 2.25 (3H, s, H69), 2.19 (3H, s, H53), 2.09 – 1.93 (32H, m, H4, H5, H8, H9, H12, H13, H16, H17, H20, H21, H24, H25, H28, H29, H32, H33), 1.79 (3H, d, *J* = 1.2 Hz, H35), 1.60 – 1.54 (21H, m, H36, H37, H38, H39, H40, H41, H42) ppm.

^{13}C-NMR (151 MHz, $CDCl_3$): δ 185.6, 184.7, 171.9, 146.3, 144.7, 143.5, 137.7, 135.4, 135.1, 134.9, 133.51, 133.48, 133.4, 132.9, 128.2, 126.5, 126.3, 126.2, 124.6, 124.42, 124.39, 124.3, 124.0, 123.9, 122.3, 120.66, 120.65, 119.2, 117.4, 114.1, 49.9, 40.0, 39.9, 39.8, 39.7, 36.4, 36.0, 35.3, 29.9, 28.5, 26.88, 26.86, 26.85, 26.8, 26.7, 26.2, 24.9, 16.6, 16.2, 16.0, 15.1, 12.8, 11.5 ppm.

*Due to the close equivalence of multiple carbons, several signals overlap.

^{19}F-NMR (565 MHz, $CDCl_3$): δ – 144.56 (d, *J* = 80.6 Hz), – 144.68 (d, *J* = 80.6 Hz) ppm.

HRMS–ESI (*m/z*): $[M+H]^+$ calculated 1115.7460; found 1115.7746.

IR (neat, cm^{-1}): 2961, 2922, 2853, 1759, 1659, 1605, 1439, 1258, 1172, 1131, 1085, 1062, 1014, 794, 669.

Rf : 0.30 (40% EtOAc in hexane).

CoQ10-ω-biotin (36)

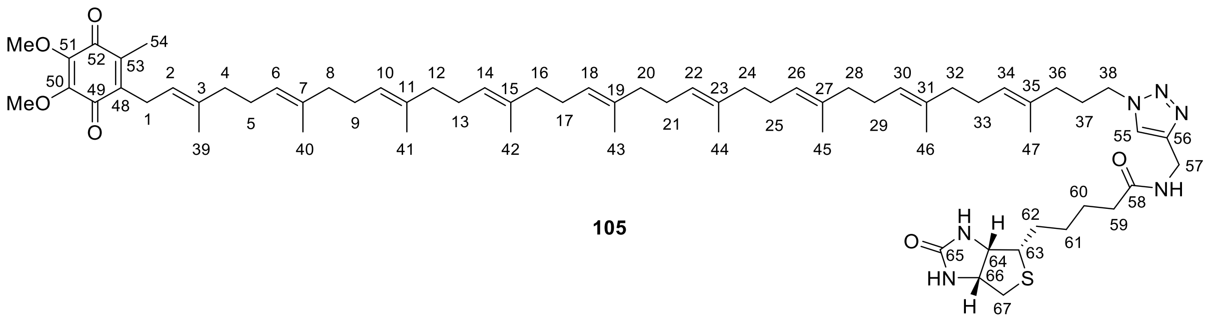

105

CoQ10-ω-biotin (**36**) was synthesised according to general method A. Flash chromatography (10:90 $MeOH:CH_2Cl_2$) afforded CoQ10-ω-biotin (**36**) (9.4 mg, 0.008 mmol, 67%).

^{1}H-NMR (400 MHz, $CDCl_3$): δ 7.74 – 7.71 (1H, m, NH), 7.56 (1H. s, H55), 6.98 (1H, s, NH), 6.39 (1H, s, NH), 5.15 – 5.04 (8H, m, H4, H6, H10, H14, H18, H22, H26, H30, H34), 4.93 (1H, tt, *J* = 7.0, 1.2 Hz, H2), 4.58 – 4.53 (2H, m, H57, H66), 4.37 – 4.32 (2H, m, H57’, H64), 4.25 (2H, t, *J* = 6.7 Hz, H38), 3.99 (3H, s, OMe), 3.97 (3H, s, OMe), 3.18 (2H, d, *J* = 7.0 Hz, H1), 3.15 – 3.10 (1H, m, H63), 2.93 (1H, dd, *J* = 12.8, 4.9 Hz, H67), 2.79 (H1, d, *J* = 12.8 Hz, H67’), 2.29 – 2.10 (2H, m, H59), 2.09 – 1.95 (37H, m, H4, H5, H8, H9, H12, H13, H16, H17, H20, H21, H24, H25, H28, H29, H32, H33, H36, H54), 1.73 – 1.62 (11H, m, H37, H39, H60, H61, H62), 1.60 – 1.57 (24H, m, H40, H41, H42, H43, H44, H45, H46, H47) ppm.

^{13}C-NMR (101 MHz, $CDCl_3$): δ 184.9, 184.09, 184.06, 173.3, 164.6, 145.1, 144.5, 144.4, 141.8, 139.0, 137.8, 135.4, 135.2, 135.10, 135.07, 135.05, 134.9, 132.9, 126.2, 124.6, 124.41, 124.39, 124.3, 124.0, 122.6, 122.5, 119.0, 61.7, 61.30, 61.29, 60.4, 55.6, 50.0, 40.8, 39.90, 39.88, 39.86, 39.8, 39.7, 36.4, 35.9, 34.4, 29.9, 28.4, 28.08, 28.06, 26.88, 26.85, 26.81, 26.80, 26.79, 26.7, 25.5, 25.4, 16.5, 16.20, 16.18, 16.1, 16.0, 12.1 ppm.

*Due to the close equivalence of multiple carbons, several signals overlap.

HRMS–ESI (*m/z*): $[M+Na]^+$ calculated 1167.7630; found 1167.7551.

IR (neat, cm^{-1}): 3289m 2917, 2850, 1702, 1646, 1610, 1541, 1437. 1325, 1286, 1262, 1202, 1150, 1023, 837, 742.

Rf = 0.10 (20% EtOAc in hexane).

Hex-LysE (37)

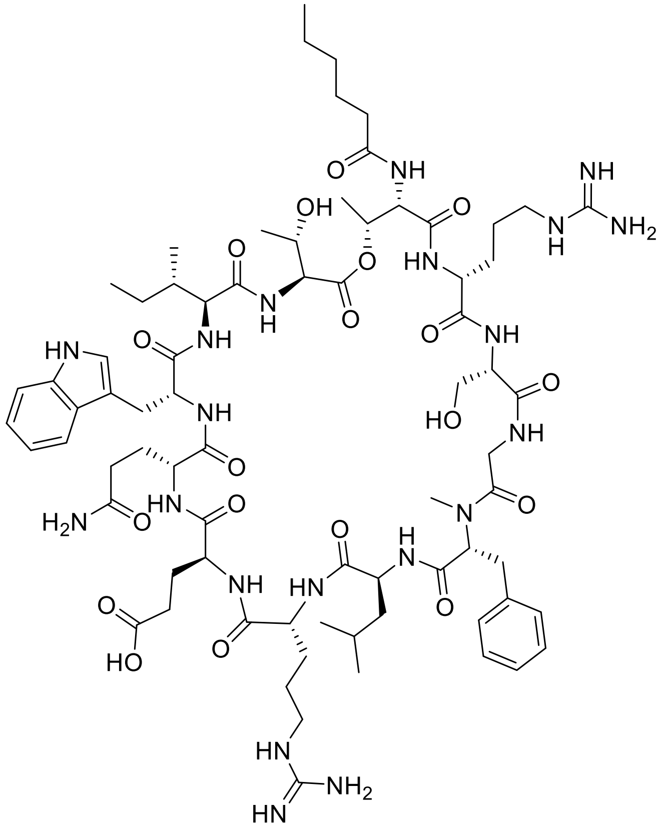

Hex-Lys (**37**) was synthesised according to modified literature procedures.[11,12] General SPPS method was applied to Fmoc-L-Leu-OH loaded CT resin (400 mg, 0.20 mmol). Esterification was performed by suspending the loaded resin and Fmoc-L-Thr(*t*Bu)-OH (795 mg, 2.0 mmol) in dry CH_2Cl_2/DMF (9:1, 8 mL). DIC (0.31 mL, 2.0 mmol) and DMAP (24 mg, 0.2 mmol) were added, and the reaction stirred under argon for 2 h. The resin was drained and washed with CH_2Cl_2 (3 x 6 mL) and DMF (3 x 6 mL). Further SPPS was performed using the general SPPS method. After one final Fmoc deprotection, the peptide was cleaved from resin by stirring in 20% HFIP in CH_2Cl_2 (8 mL) at ambient temperature for 6 h. The resin was filtered and washed with CH_2Cl_2 (3 x 8 mL), the filtrate was concentrated under reduced vacuum. The crude peptide was cyclised by dissolving in dry DMF (80 mL) with PyBOP (835 mg, 1.60 mmol) and DIPEA

(1.0 mL, 6.0 mmol) and stirred under argon at ambient temperature for 18 h. The reaction mixture was then diluted with CH_2Cl_2 (160 mL) and washed with formic acid (1% Aq., 5 x 15 mL). The organic fraction was concentrated *in vacuo* and dissolved in TFA:TIPS:H_2O (95:2.5:2.5, 8 mL). The reaction mixture was stirred at ambient temperature for 2 h then concentrated *in vacuo* and the peptide precipitated as described in Cleavage and Global Deprotection Method. Purification on C18 column using HPLC afforded Hex-LysE (**37**) as a colourless solid (49.5 mg, 0.031 mmol, 16%).

HRMS–ESI (*m/z*): $[M+2H]^{2+}$ calculated 794.4357; found 794.4363.

Retention time = 9.12 min.

Hex-MBA2 (38)

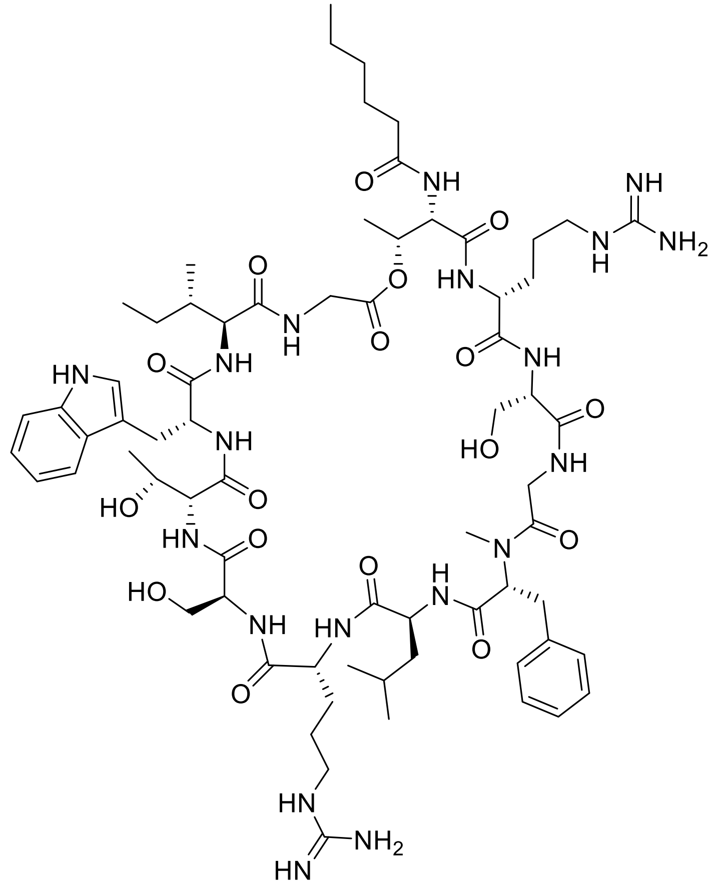

Hex-MBA2 (**38**) was synthesised according to modified literature procedures.[11,12] The General SPPS Method was applied to Fmoc-L-Leu-OH loaded CT resin (150 mg, 0.075 mmol). Esterification was performed by suspending the loaded resin and Fmoc-Gly-OH (223 mg, 0.75 mmol) in dry CH_2Cl_2/DMF (9:1, 3 mL). DIC (0.12 mL, 0.75 mmol) and DMAP (9 mg, 0.075 mmol) were added and the reaction stirred under argon for 2 h. The resin was drained and washed with CH_2Cl_2 (3 x 6 mL) and DMF (3 x 6 mL). Further SPPS was performed using the general SPPS method. After one final Fmoc deprotection, the peptide was cleaved from resin by stirring in 20% HFIP in CH_2Cl_2 (3 mL) at ambient temperature for 6 h. The resin was filtered and washed with CH_2Cl_2 (3 x 3 mL), the filtrate was concentrated under reduced vacuum. The

crude peptide was cyclised by dissolving in dry DMF (30 mL) with PyBOP (312 mg, 0.60 mmol) and DIPEA (0.4 mL, 2.25 mmol) and stirred under argon at ambient temperature for 18 h. The reaction mixture was then diluted with CH_2Cl_2 (60 mL) and washed with formic acid (1% Aq., 5 x 5 mL). The organic fraction was concentrated *in vacuo* and dissolved in TFA:TIPS:H_2O (95:2.5:2.5, 3 mL). The reaction mixture was stirred at ambient temperature for 2 h then concentrated *in vacuo* and the peptide precipitated as described in Cleavage and Global Deprotection Method. Purification on C18 column using HPLC afforded Hex-MBA2 (**38**) as a colourless solid (13.6 mg, 0.009 mmol, 12%).

HRMS–ESI (*m/z*): $[M+2H]^{2+}$ calculated 737.9119; found 737.9174.

Retention time = 9.15 min.

References

1. W. Ma, D. W. Li, T. C. Sutherland, Y. Li, Y. T. Long, H. Y. Chen, *J. Am. Chem. Soc.* **2011**, *133*, 12366–12369.

2. K. Okamoto, E. Mizuta, K. Kamiya, I. Imada, *Chem. Pharm. Bull.* **1985**, *33*, 3756–3765.

3. R. V. K. Cochrane, F. M. Alexander, C. Boland, S. K. Fetics, M. Caffrey, S. A. Cochrane, *Chem. Commun.* **2020**, *56*, 8603–8606.

4. Z. Guan, X. Chai, S. Yu, H. Hu, Y. Jiang, Q. Meng, Q. Wu, *Chem. Biol. Drug Des.* **2010**, *76*, 496–504.

5. W. Stadlbauer, G. Hojas, *J. Heterocycl. Chem.* **2003**, *40*, 753–756.

6. M. Albuszis, P. J. Roth, W. Pauer, H. U. Moritz, *Polym. Chem.* **2014**, *5*, 5689–5699.

7. B. Lin, X. Wu, H. Zhao, Y. Tian, J. Han, J. Liu, S. Han, *Chem. Sci.* **2016**, *7*, 3737–3741.

8. T. Katoh, M. Yoshikawa, T. Yamamoto, R. Arai, N. Nii, Y. Tomata, S. Suzuki, R. Koyama, N. Negoro, T. Yogo, *Bioorg. Med. Chem. Lett.* **2017**, *27*, 1145–1148.

9. C. C. Cosner, J. T. Markiewicz, P. Bourbon, C. J. Mariani, O. Wiest, M. Rujoi, A. I. Rosenbaum, A. Y. Huang, F. R. Maxfield, P. Helquist, *J. Med. Chem.* **2009**, *52*, 6494–6498.

10. C. Besanceney-Webler, H. Jiang, T. Zheng, L. Feng, D. Soriano Del Amo, W. Wang, L. M. Klivansky, F. L. Marlow, Y. Liu, P. Wu, *Angew. Chem. Int. Ed.* **2011**, *50*, 8051–8056.

11. L. Li, B. Koirala, Y. Hernandez, L. W. MacIntyre, M. A. Ternei, R. Russo, S. F. Brady, *Nat. Microbiol.* **2022**, *7*, 120–131.

12. M. Murai, T. Kaji, T. Kuranaga, H. Hamamoto, K. Sekimizu, M. Inoue, *Angew. Chem. Int. Ed.* **2015**, *54*, 1556–1560.

13. A. J. Burt, P. Ahmadvand, L. K. Opp, A. T. Ryan, C. H. Kang and R. J. Mancini, *ChemMedChem* **2020**, *15*, 2004–2009.

NMR Spectra

^{1}H NMR (400 MHZ, $CDCl_3$) for Me_2-CoQ10 (4)

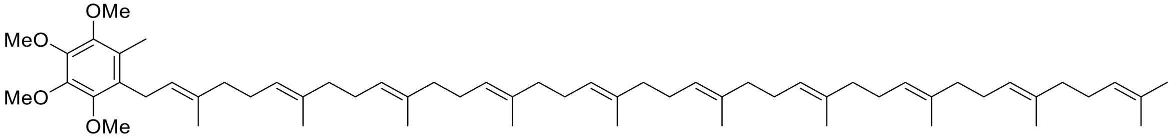

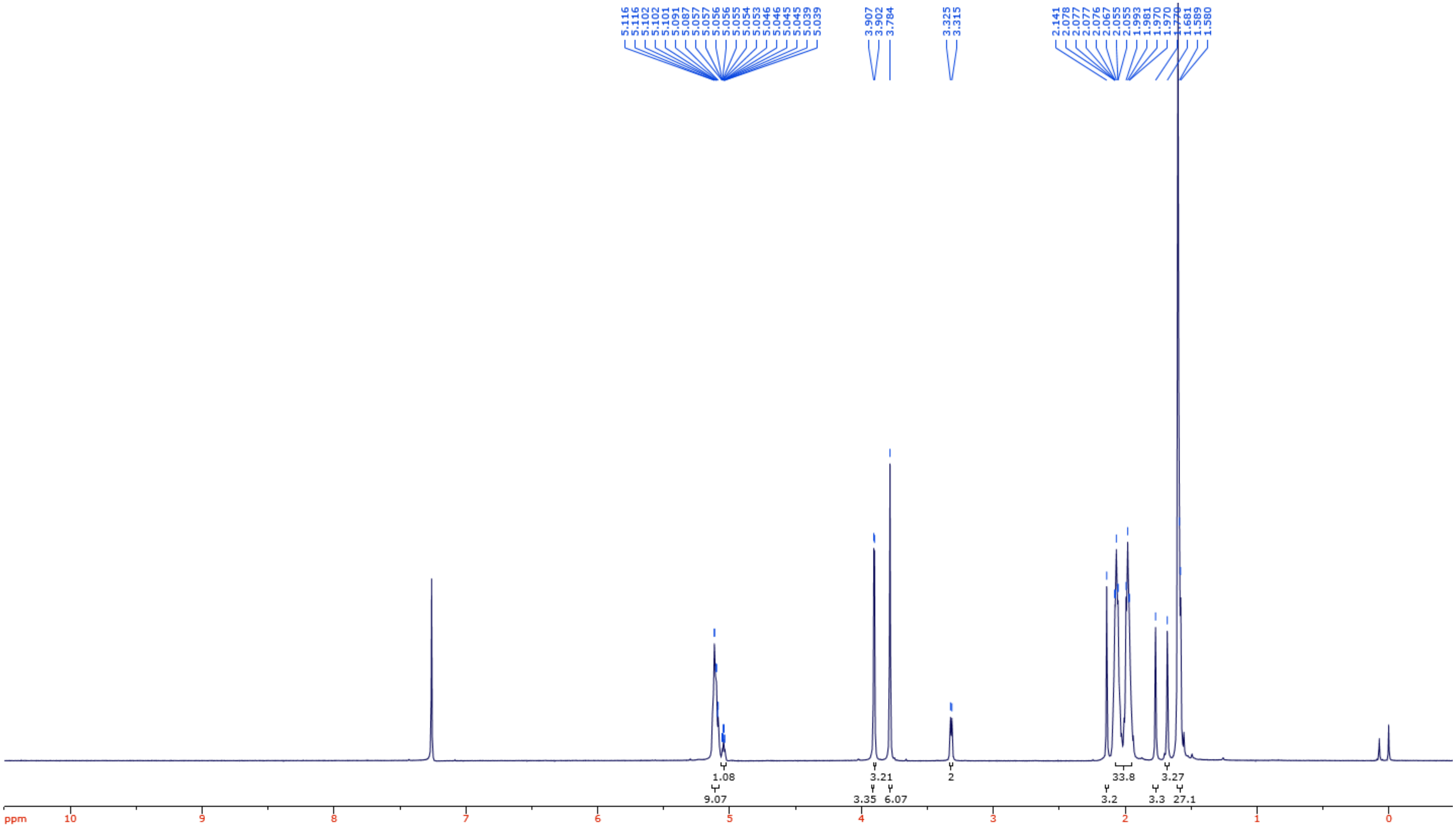

^{13}C NMR (151 MHZ, $CDCl_3$) for Me_2-CoQ10 (4)

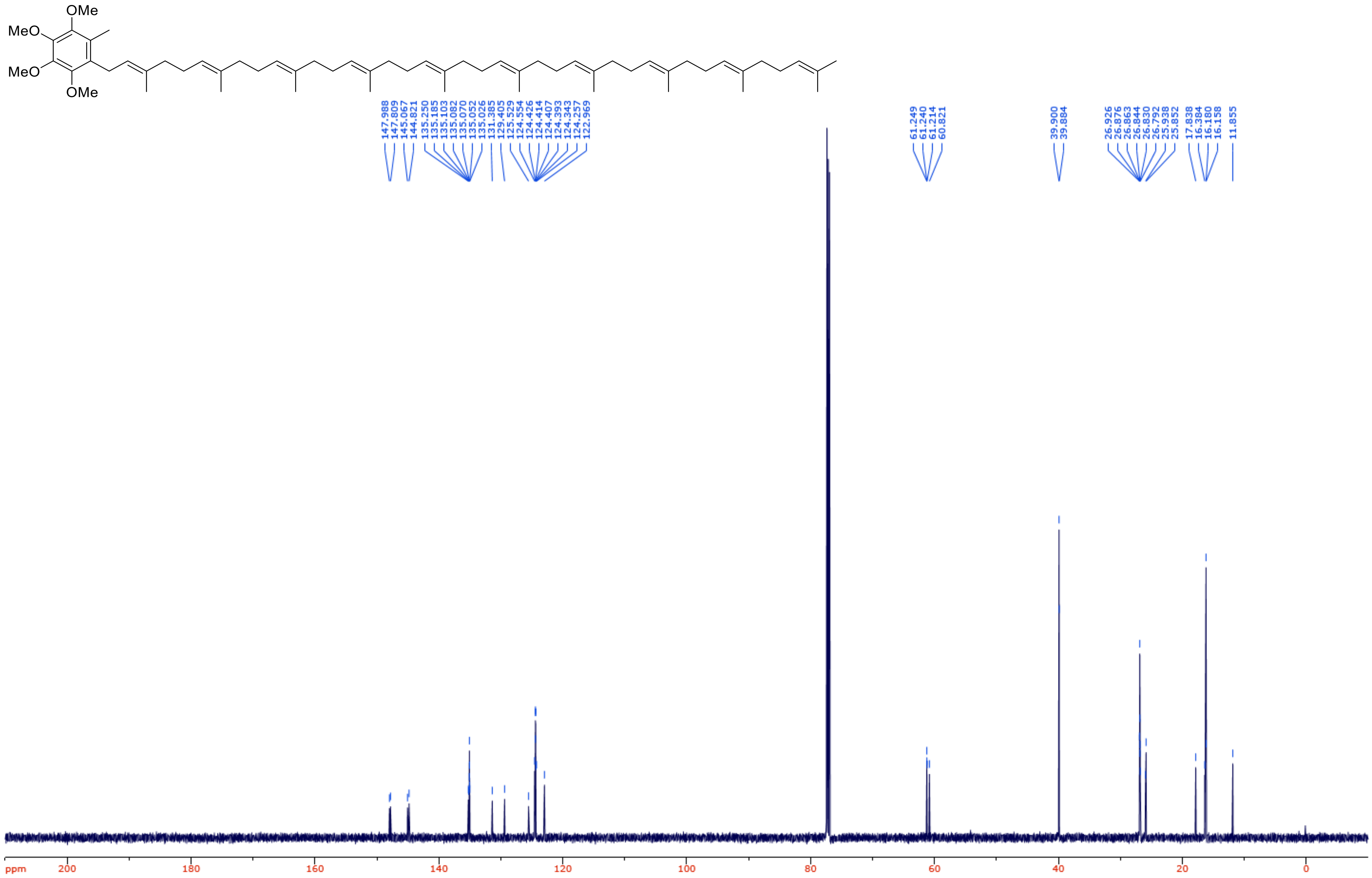

^{1}H NMR (400 MHZ, $CDCl_3$) for Boc_2-CoQ10 (5)

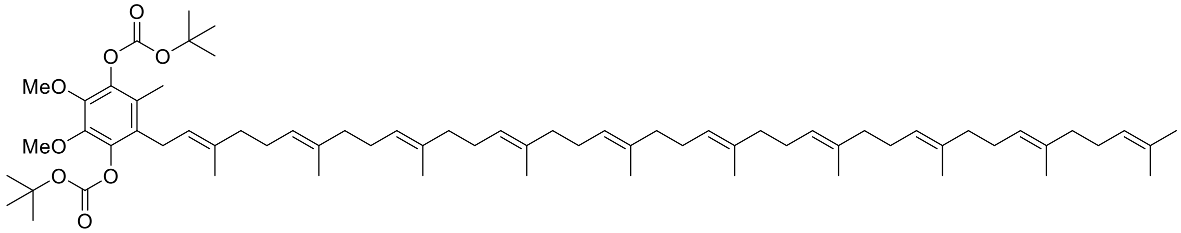

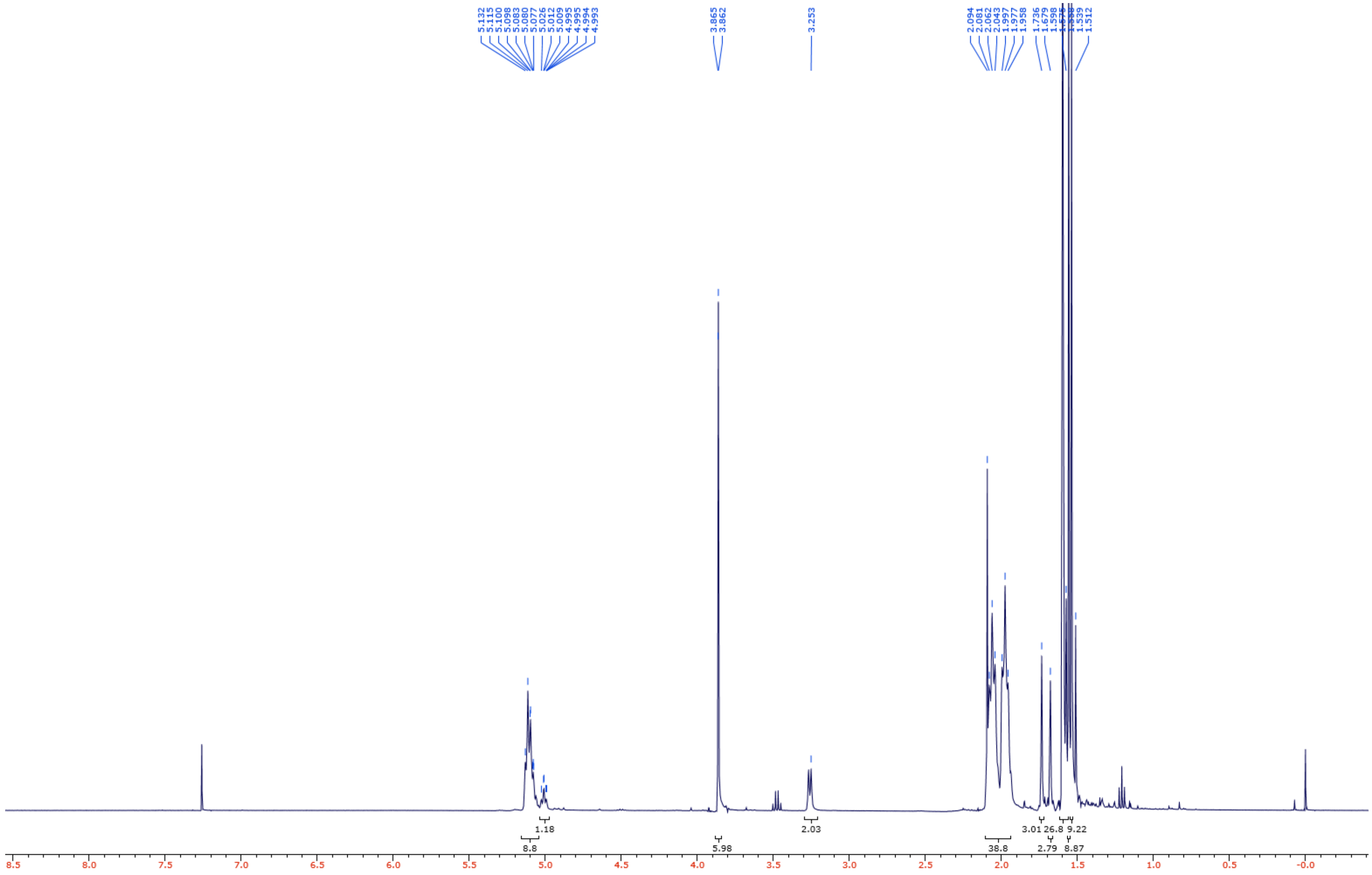

^{13}C NMR (101 MHZ, $CDCl_3$) for Boc_2-CoQ10 (5)

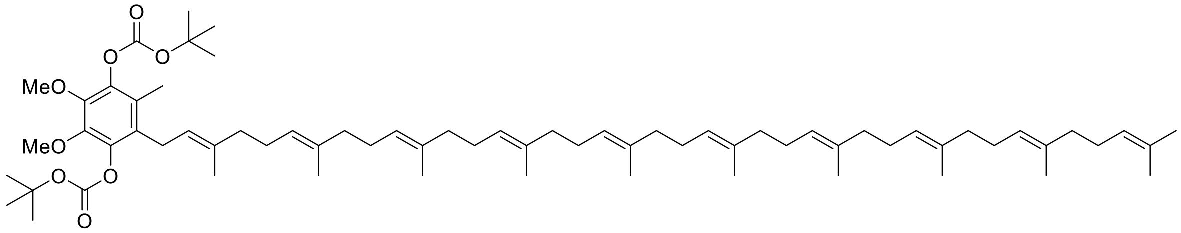

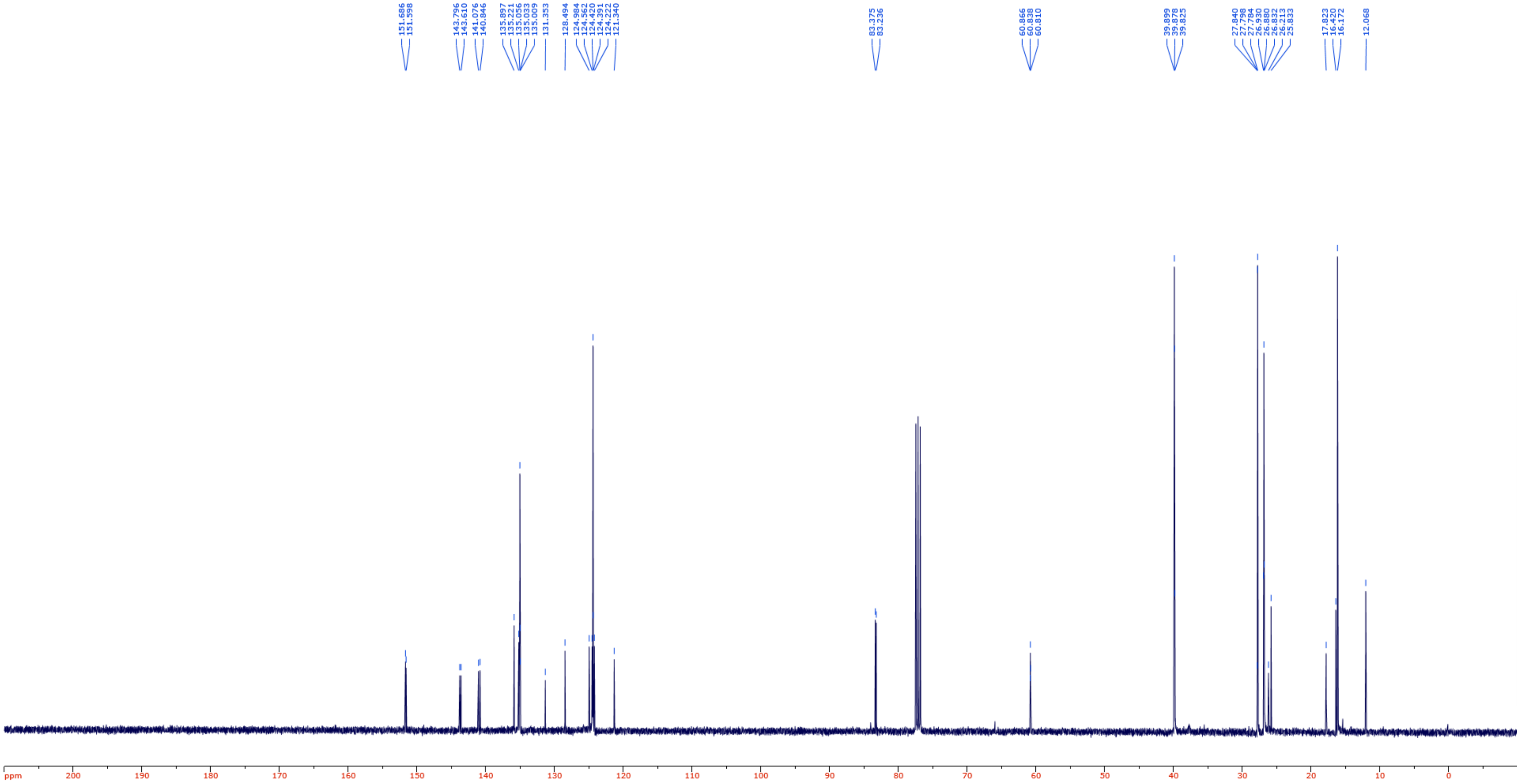

2D COSY NMR (400 MHZ, $CDCl_3$) for Boc_2-CoQ10 (5)

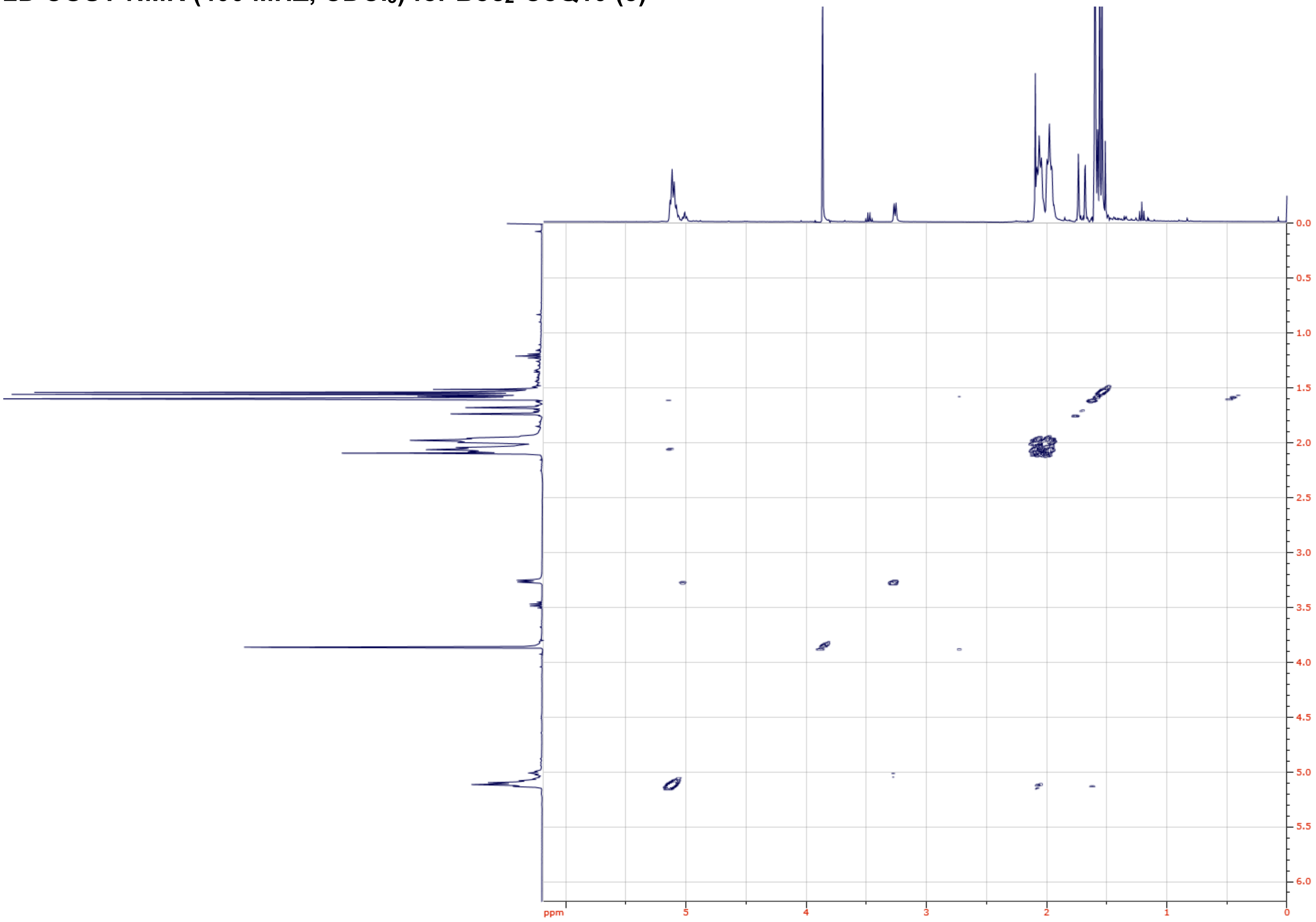

2D HSQC NMR (400 MHZ, $CDCl_3$) for Boc_2-CoQ10 (5)

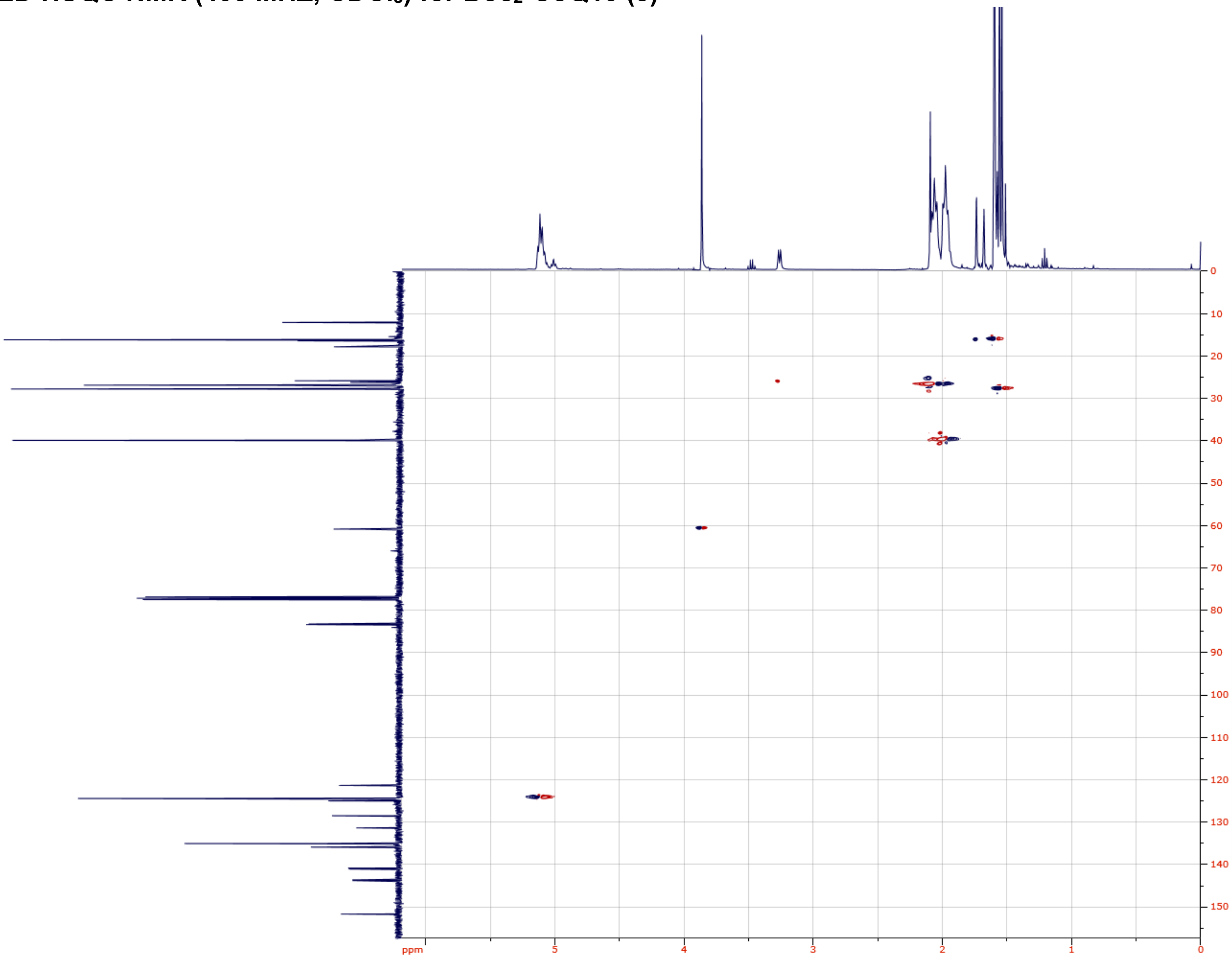

2D HMBC NMR (400 MHZ, $CDCl_3$) for Boc_2-CoQ10 (5)

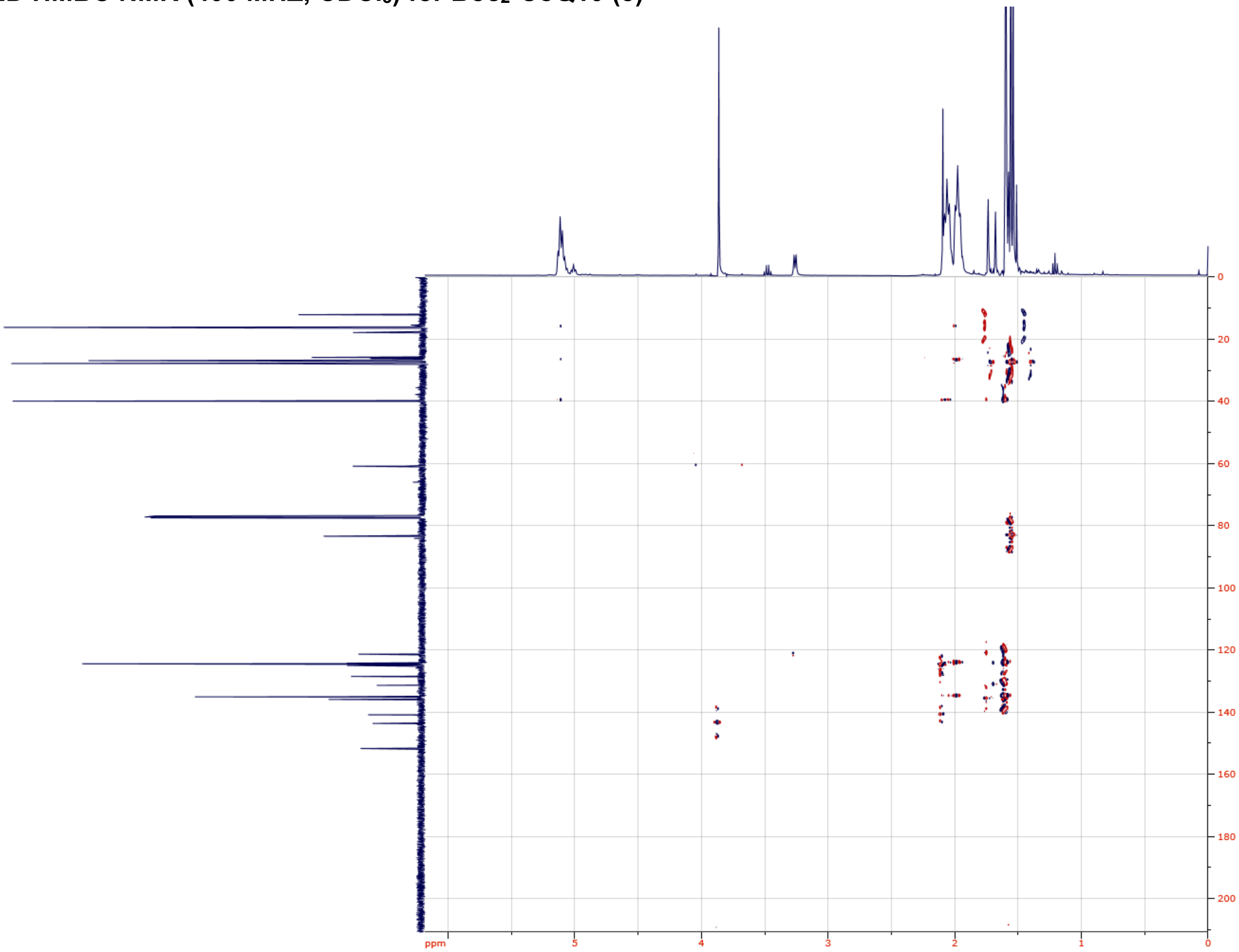

^{1}H NMR (400 MHZ, $CDCl_3$) for Boc_2-MK4 (6)

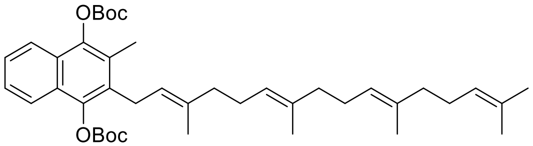

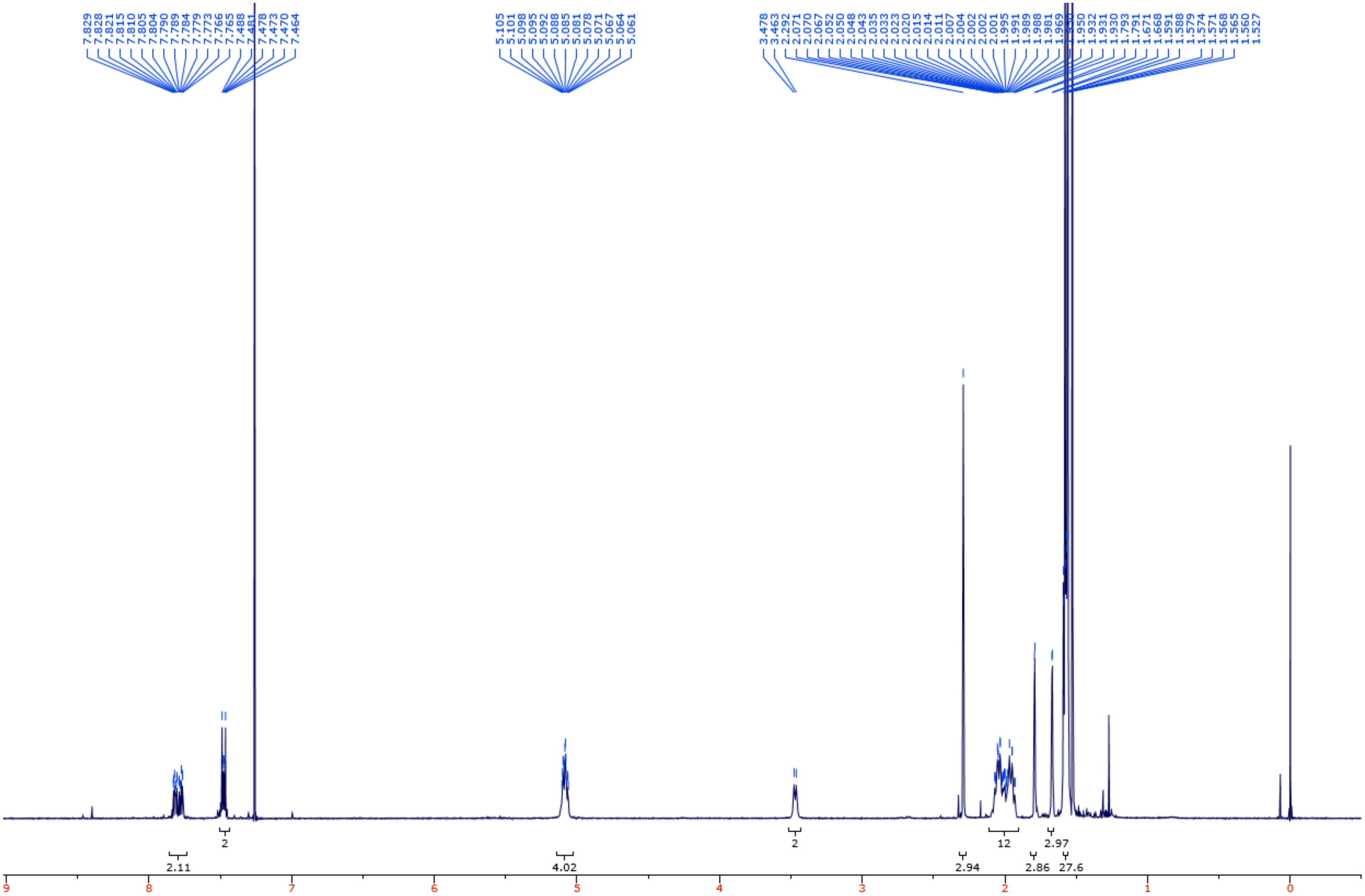

^{13}C NMR (101 MHZ, $CDCl_3$) for Boc_2-MK4 (6)

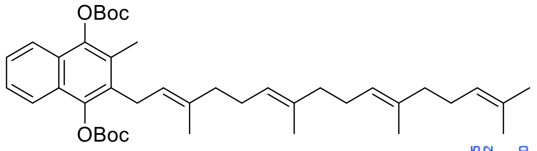

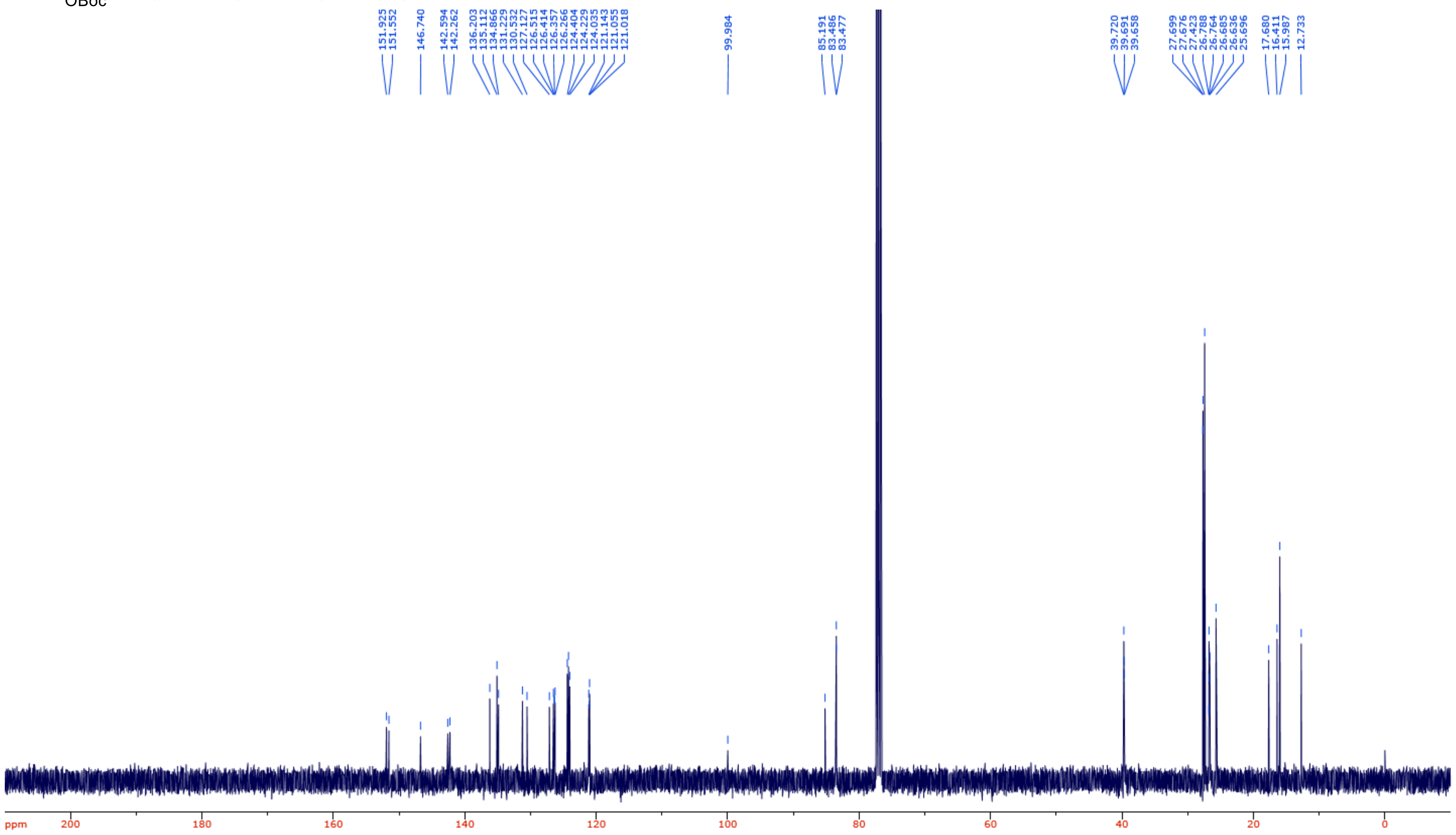

2D COSY (400 MHZ, $CDCl_3$) for Boc_2-MK4 (6)

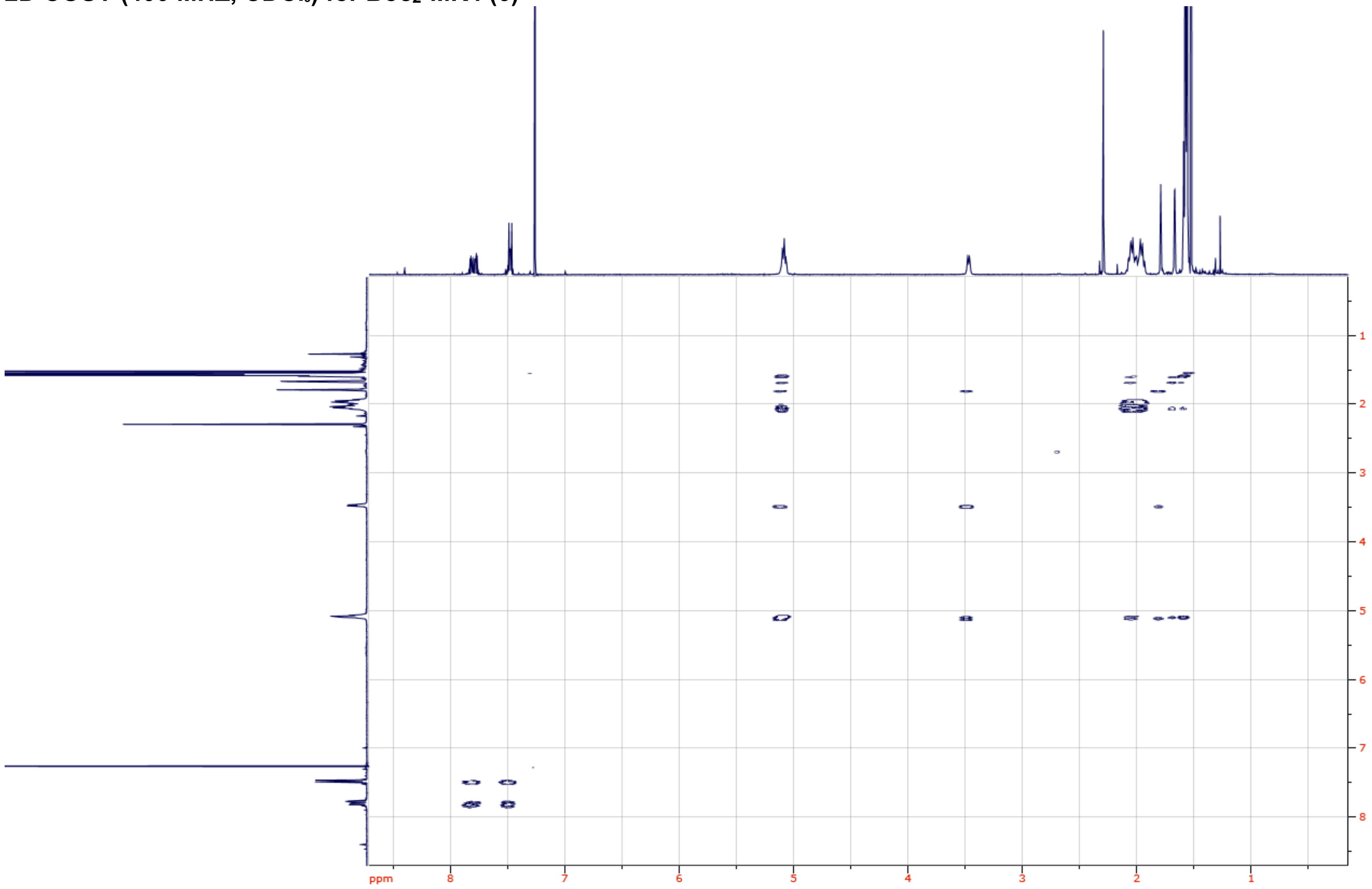

2D HSQC (400 MHZ, $CDCl_3$) for Boc_2-MK4 (6)

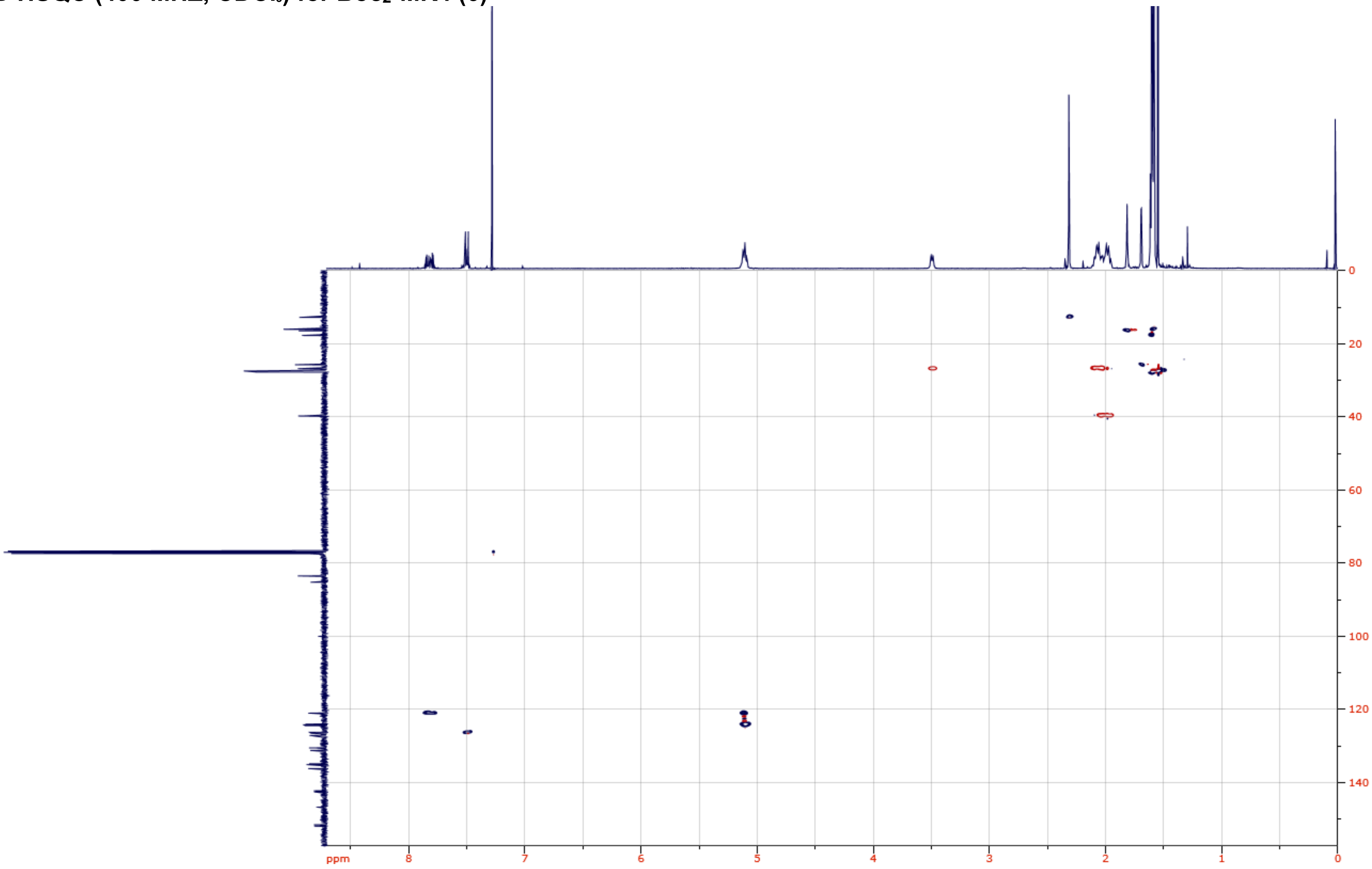

2D HMBC (400 MHZ, $CDCl_3$) for Boc_2-MK4 (6)

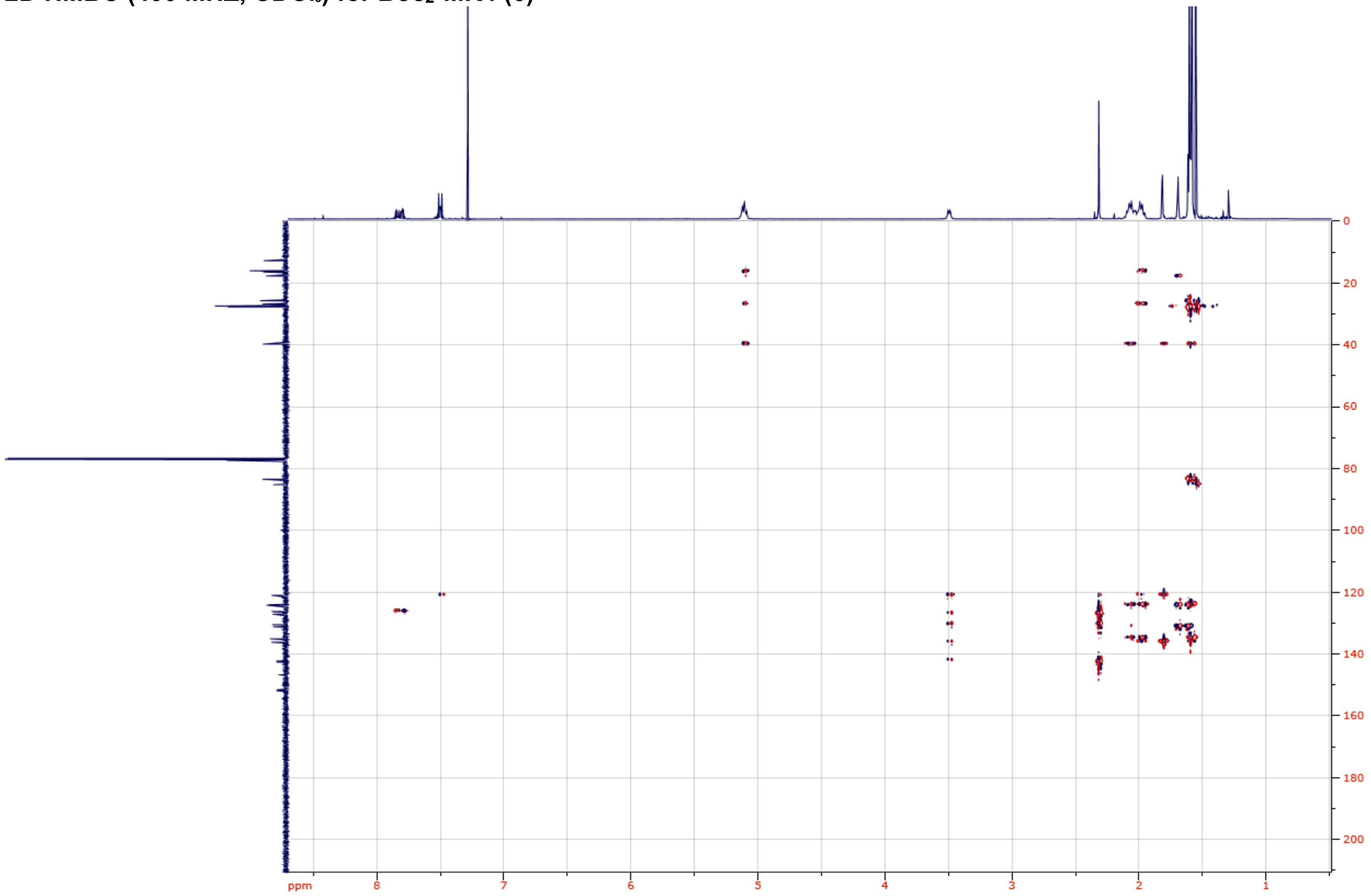

^{1}H NMR (400 MHZ, $CDCl_3$) for Boc_2-MK9 (7)

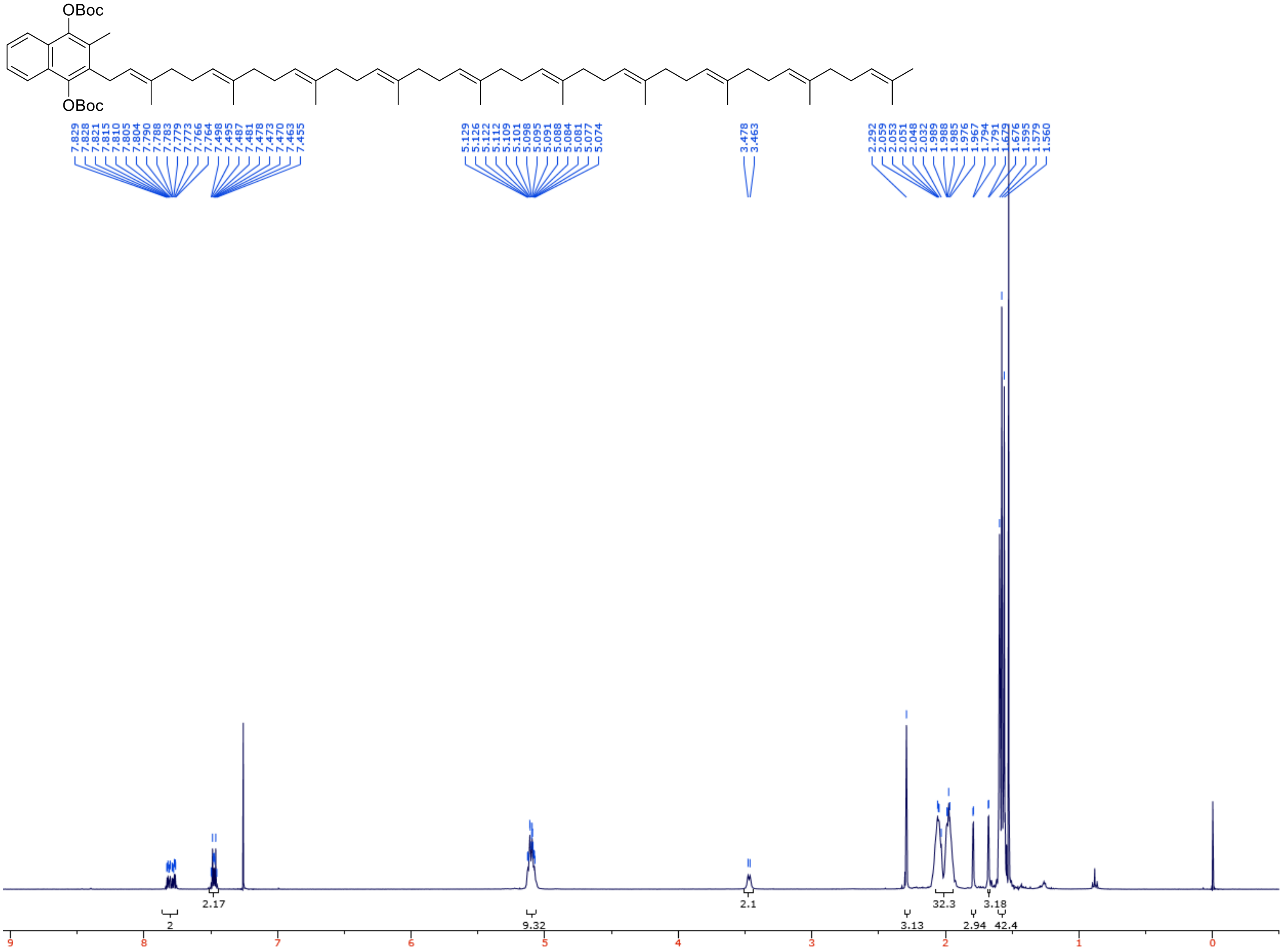

^{13}C NMR (101 MHZ, $CDCl_3$) for Boc_2-MK9 (7)

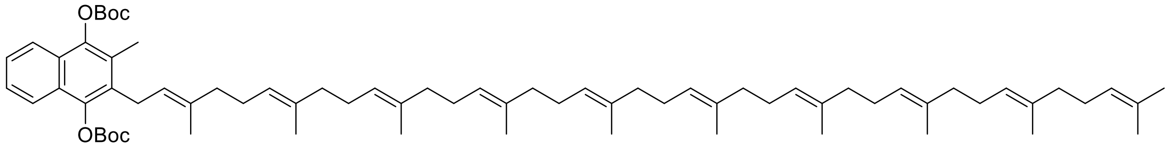

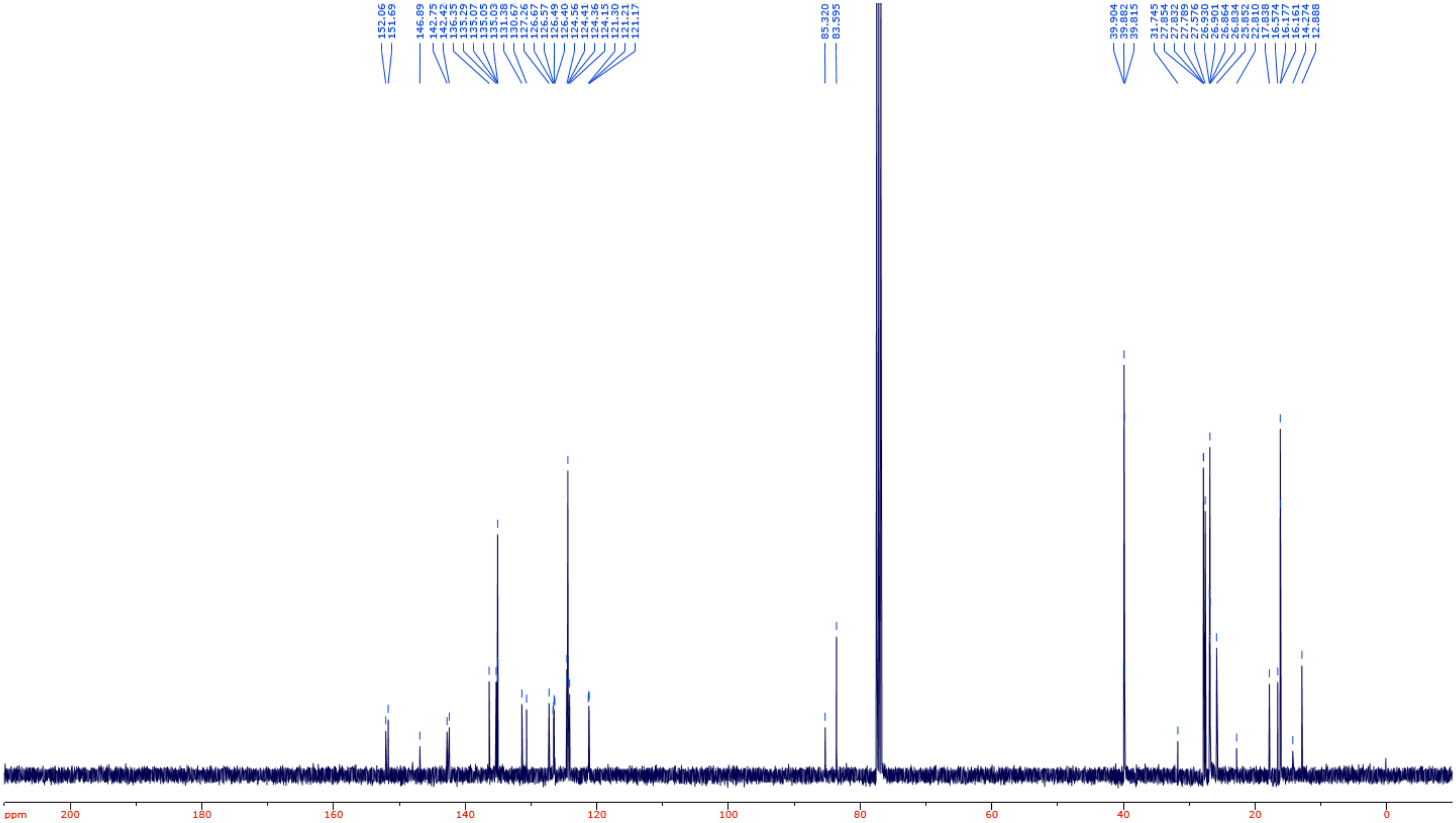

2D COSY (400 MHZ, $CDCl_3$) for Boc_2-MK9 (7)

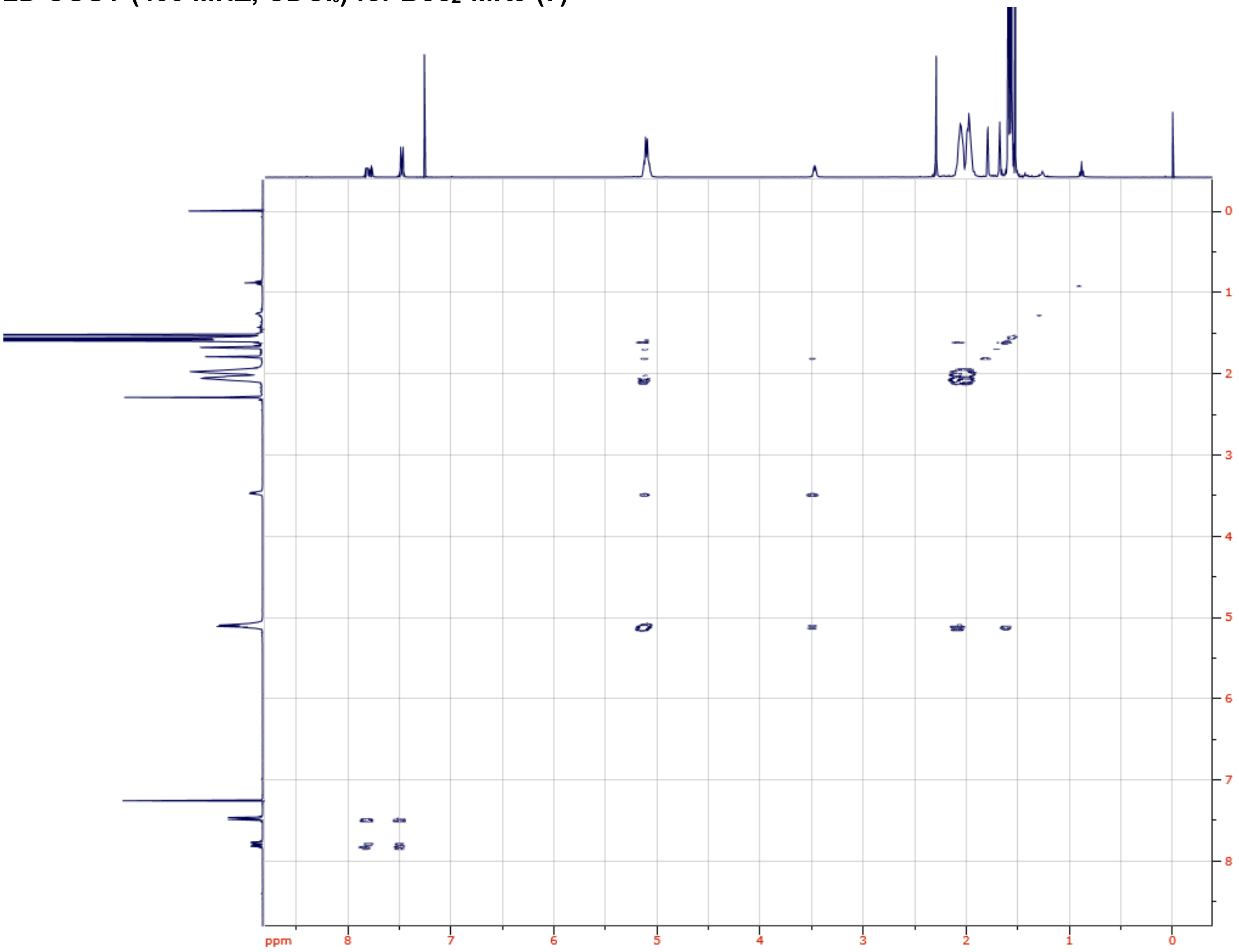

2D HSQC (400 MHZ, $CDCl_3$) for Boc_2-MK9 (7)

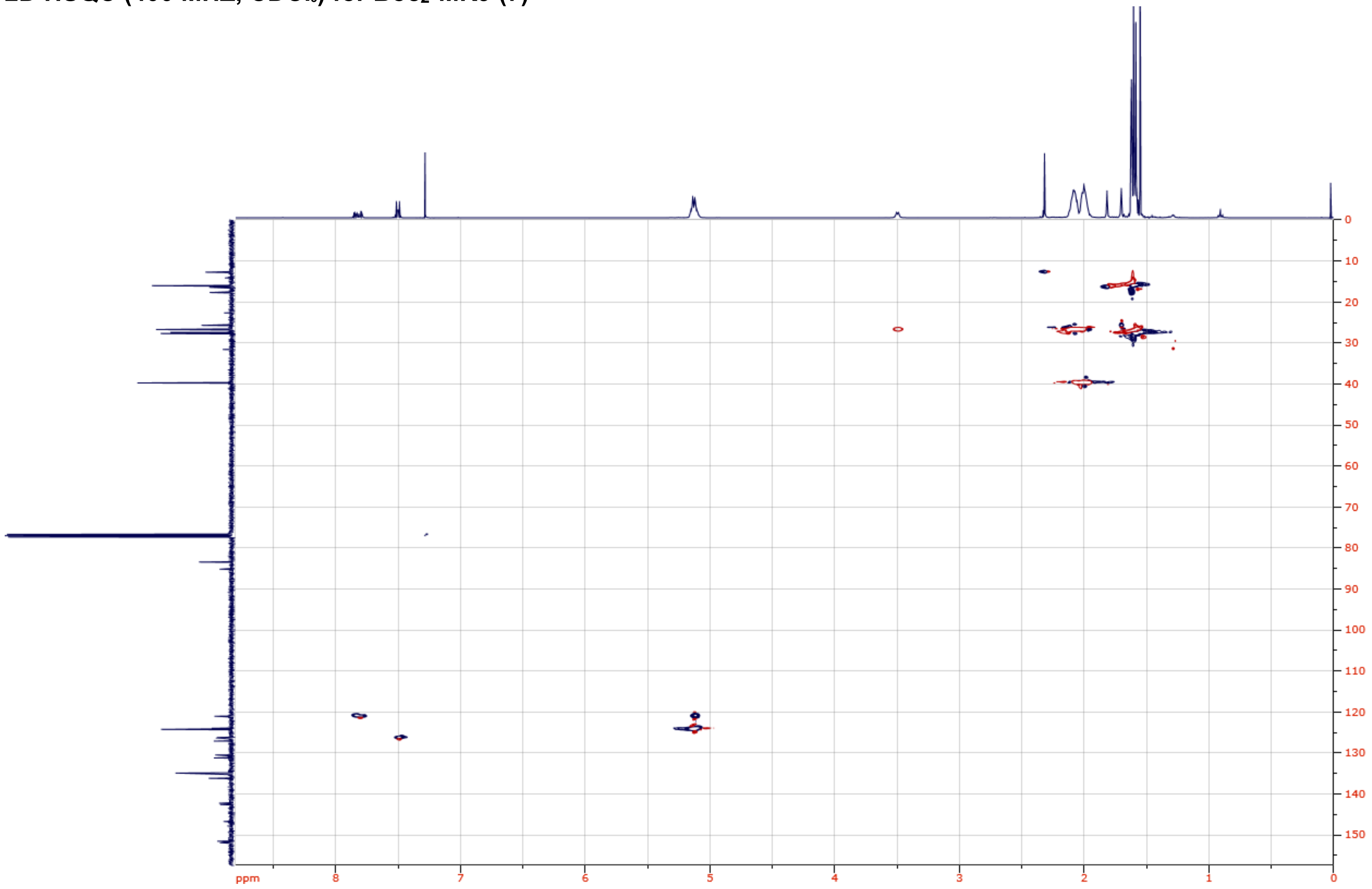

2D HMBC (400 MHZ, $CDCl_3$) for Boc_2-MK9 (7)

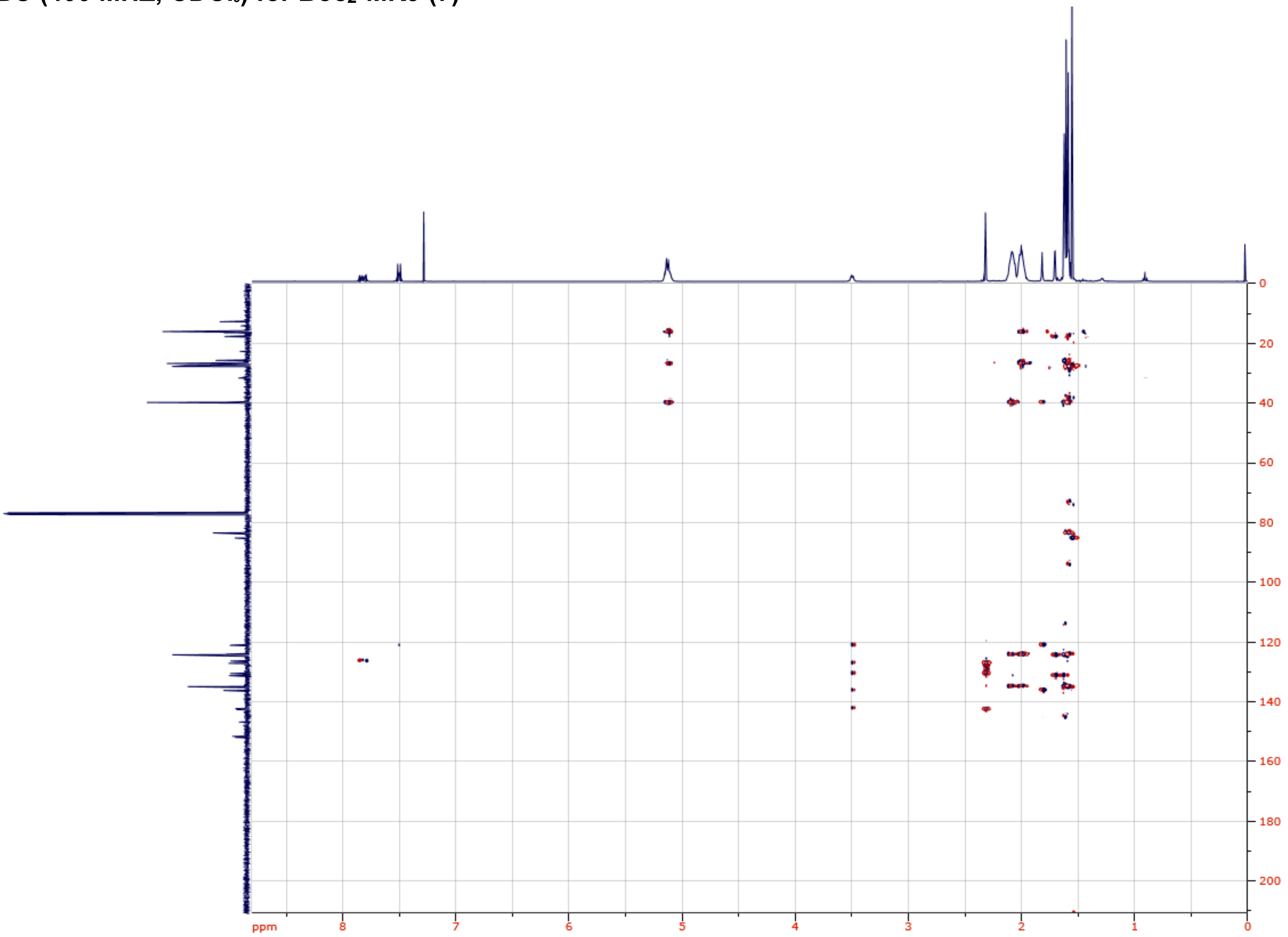

^{1}H NMR (600 MHZ, $CDCl_3$) for Me_2-CoQ10-ω-epoxide (8)

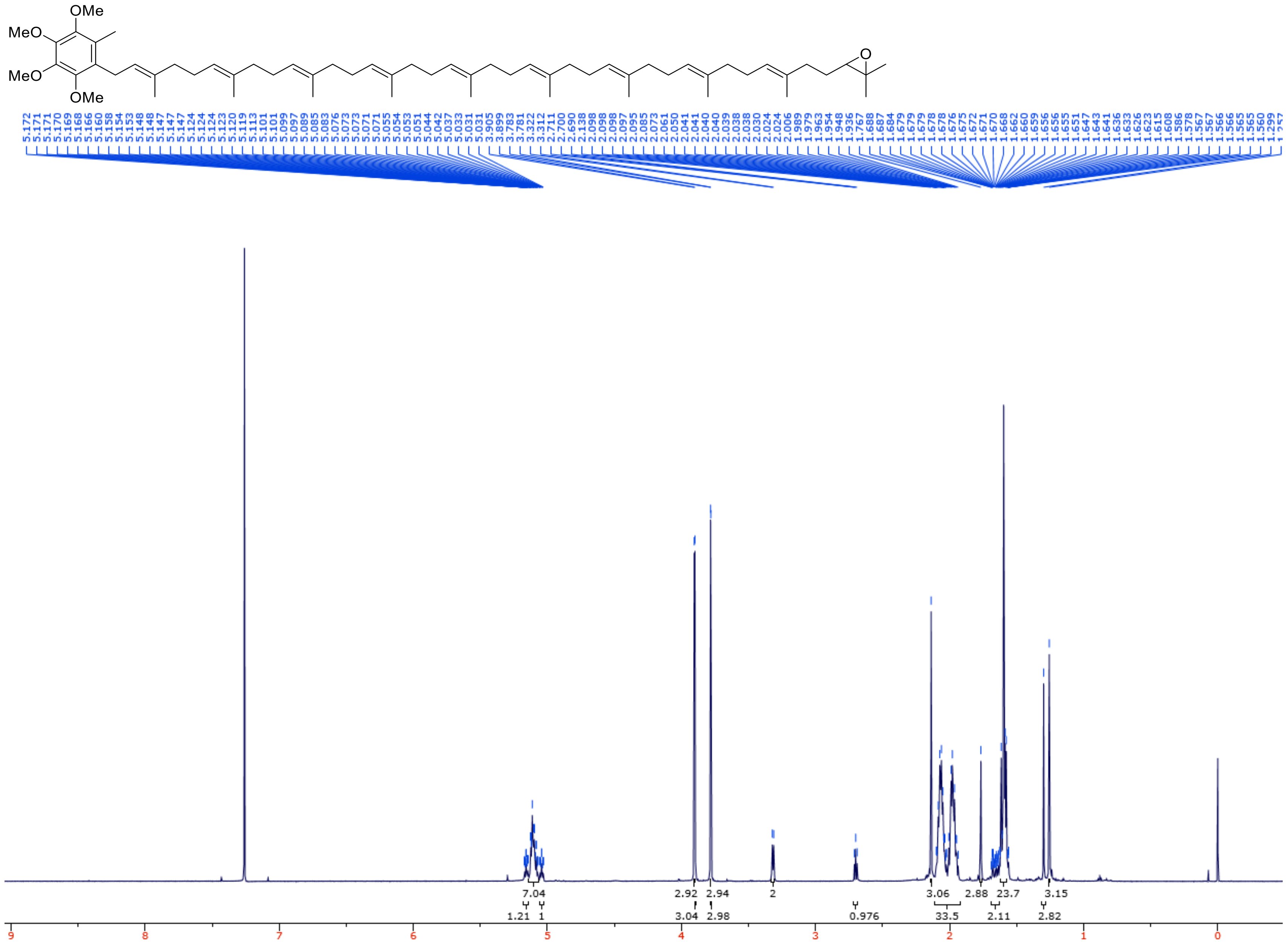

^{13}C NMR (101 MHZ, $CDCl_3$) for Me_2-CoQ10-ω-epoxide (8)

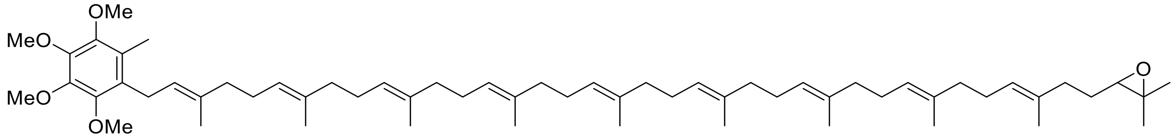

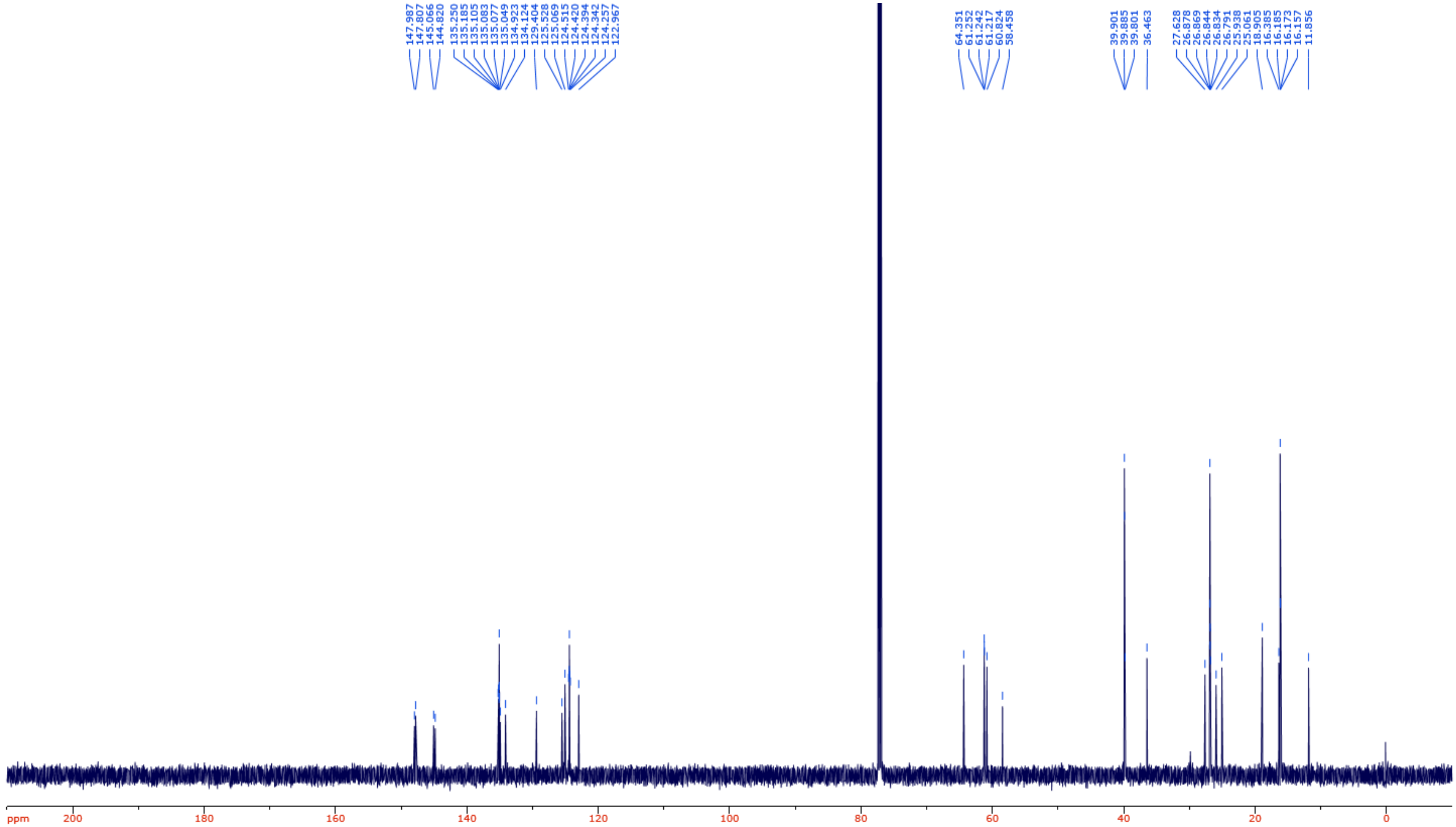

2D COSY (400 MHZ, $CDCl_3$) for Me_2-CoQ10-ω-epoxide (8)

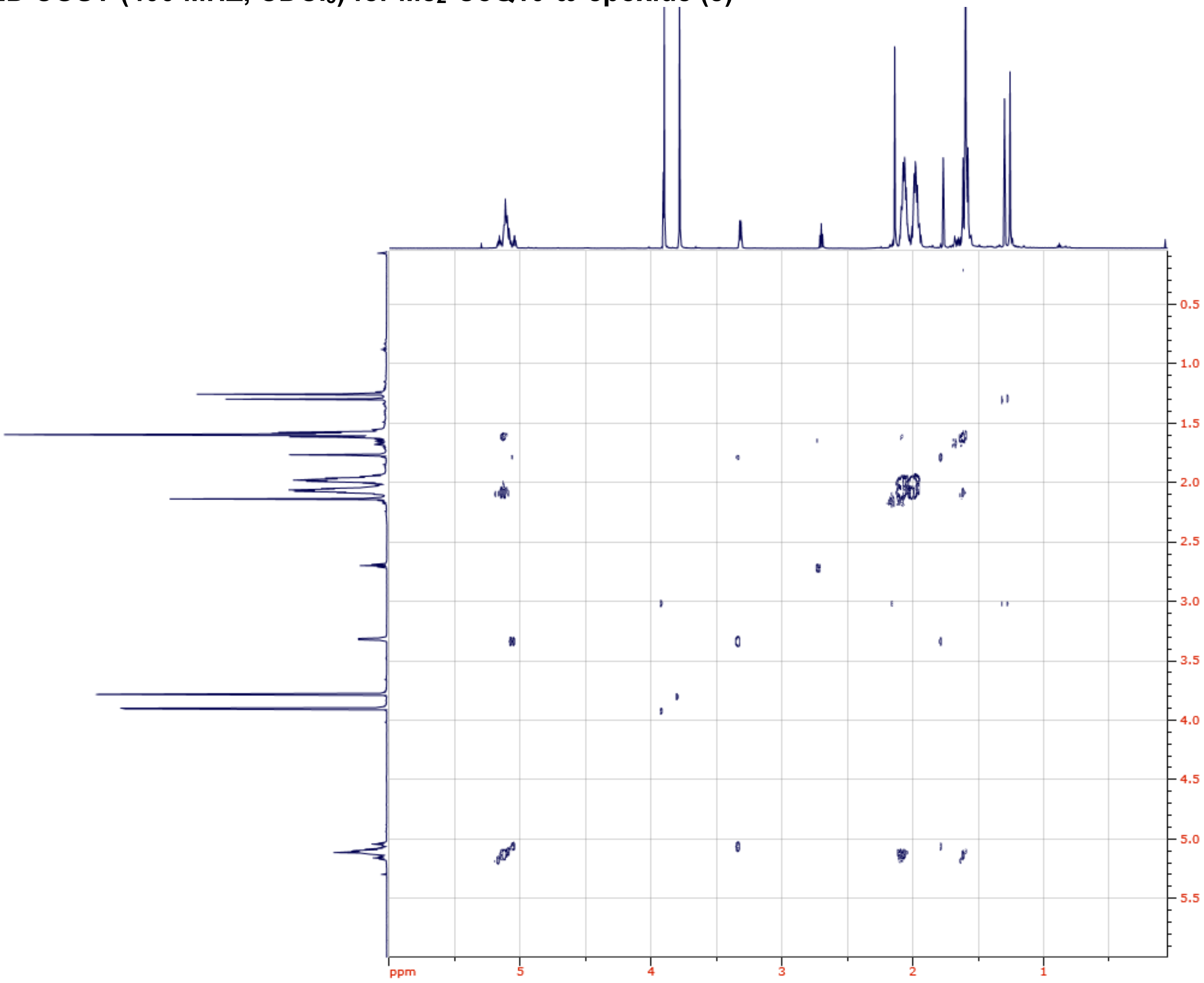

2D HSQC (400 MHZ, $CDCl_3$) for Me_2-CoQ10-ω-epoxide (8)

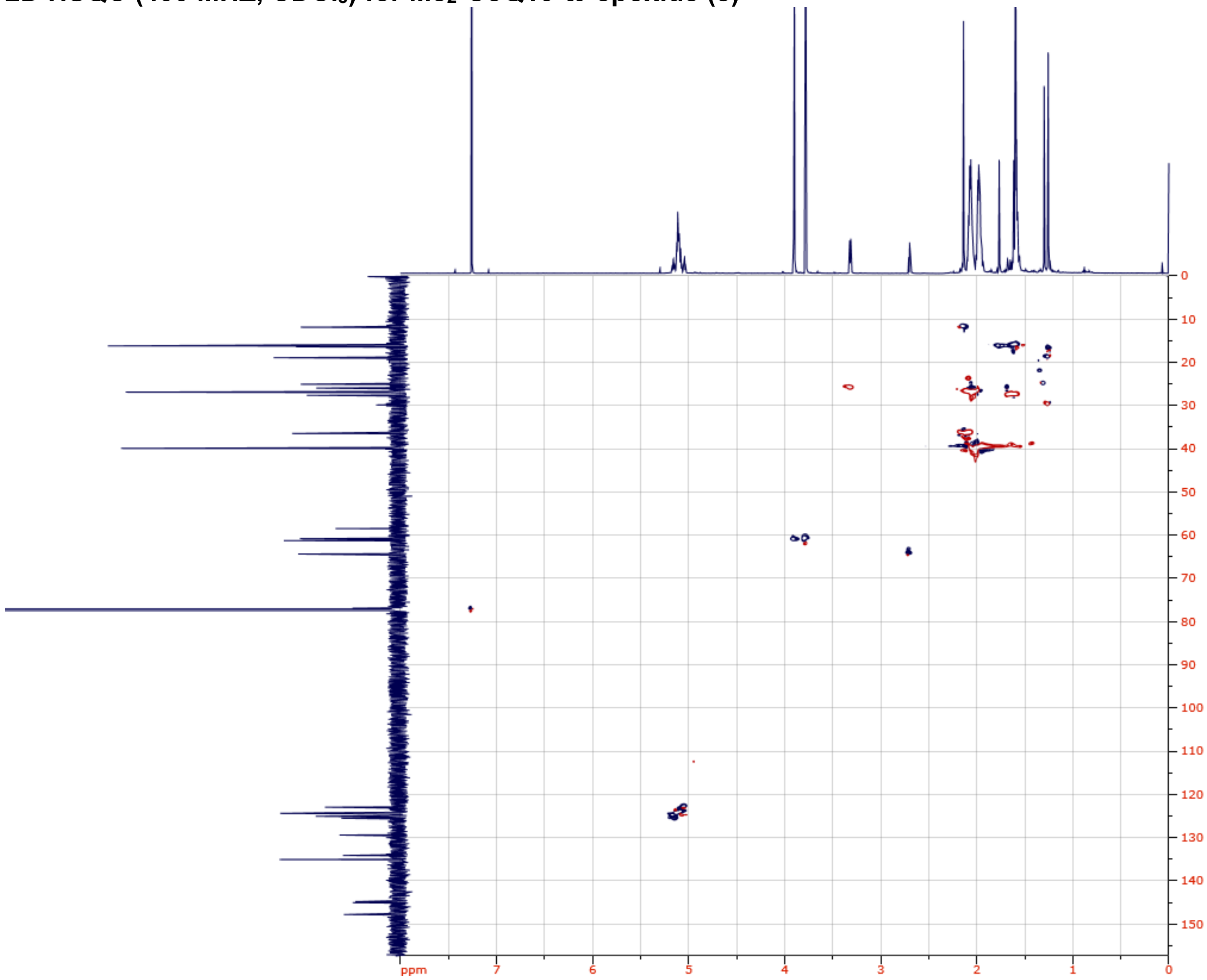

2D HMBC (400 MHZ, $CDCl_3$) for Me_2-CoQ10-ω-epoxide (8)

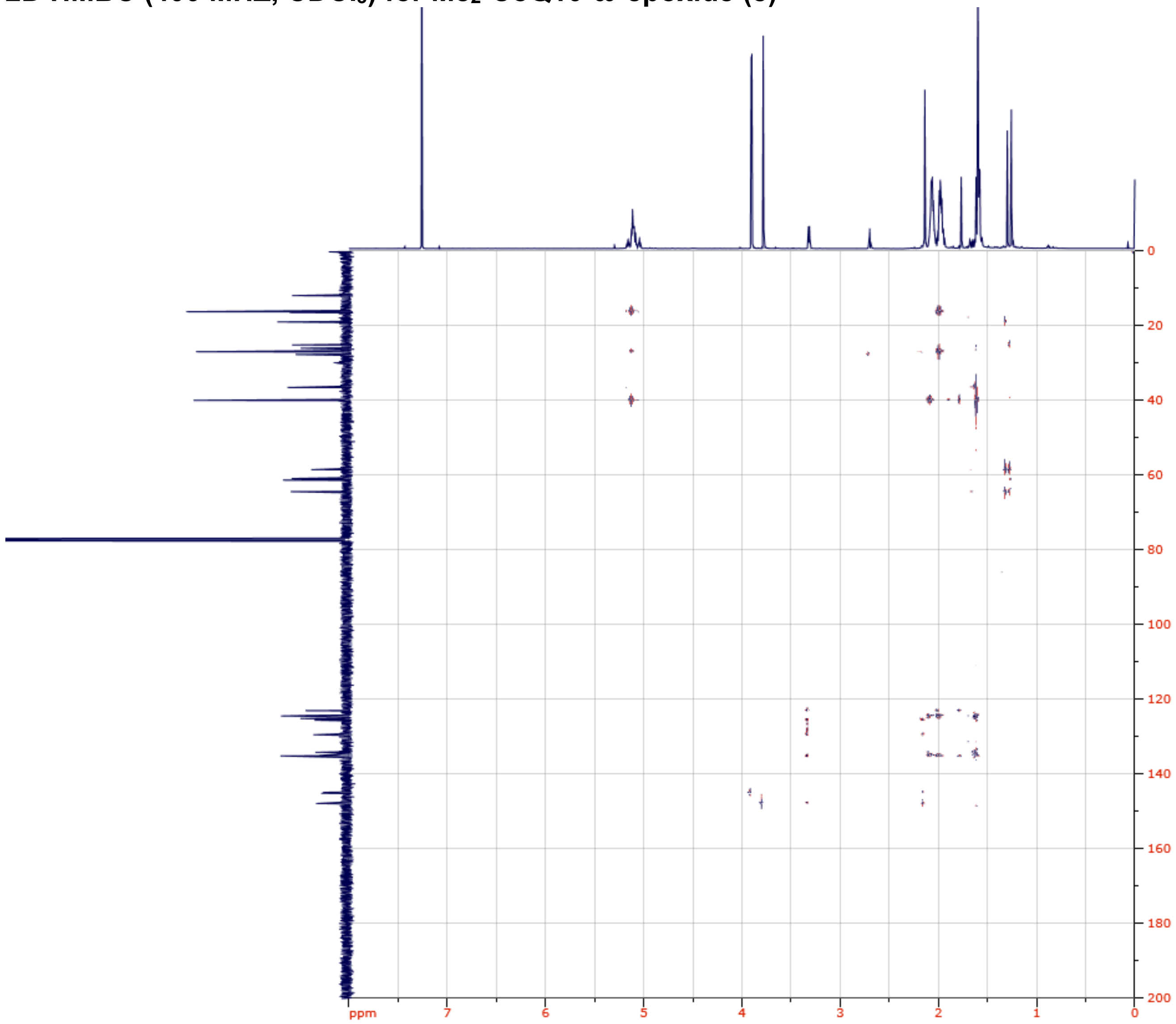

^{1}H NMR (400 MHZ, $CDCl_3$) for Boc_2-CoQ10-ω-epoxide (9)

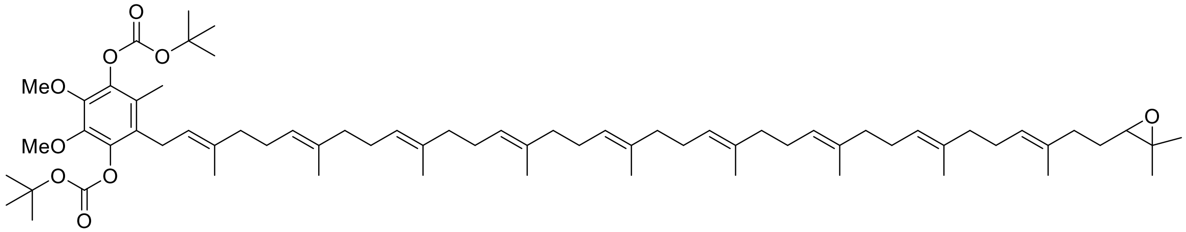

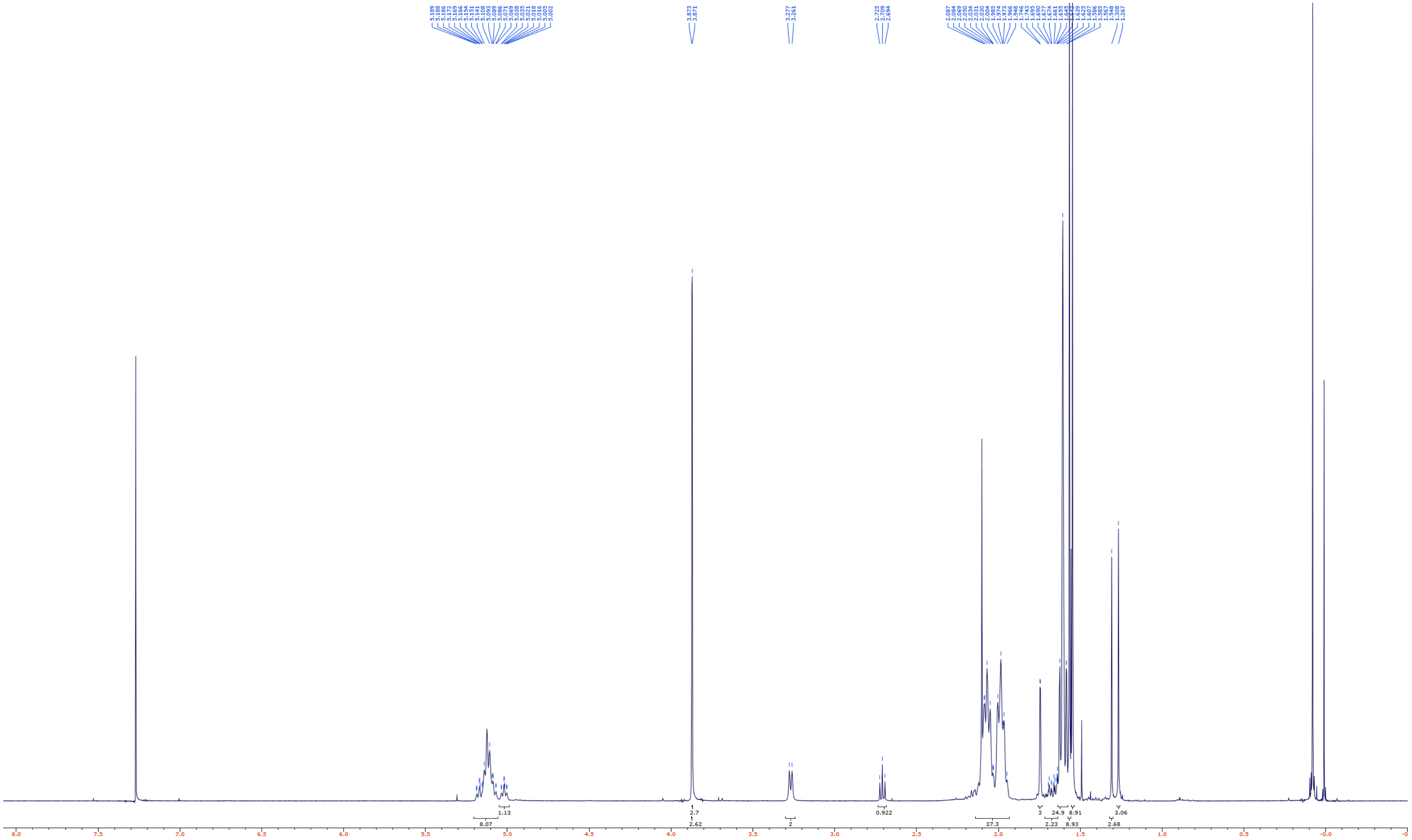

^{13}C NMR (101 MHZ, $CDCl_3$) for Boc$_2$-CoQ10-ω-epoxide (9)

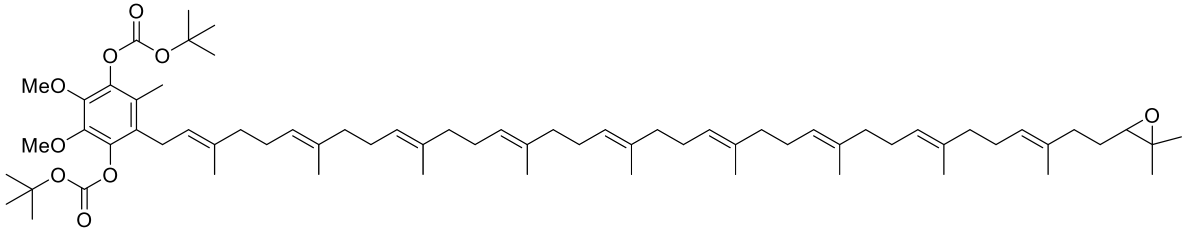

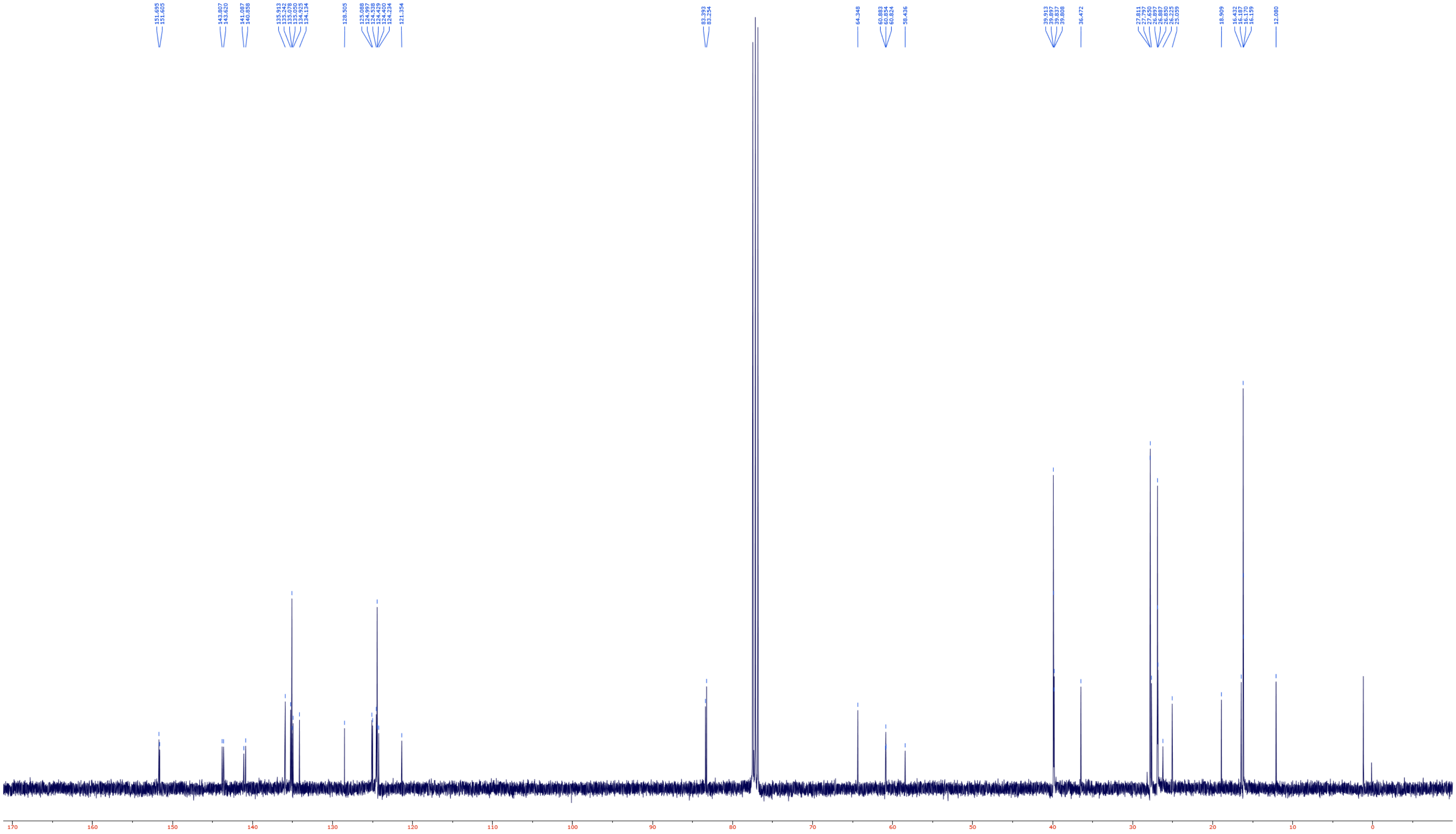

2D COSY (400 MHZ, $CDCl_3$) for Boc_2-CoQ10-ω-epoxide (9)

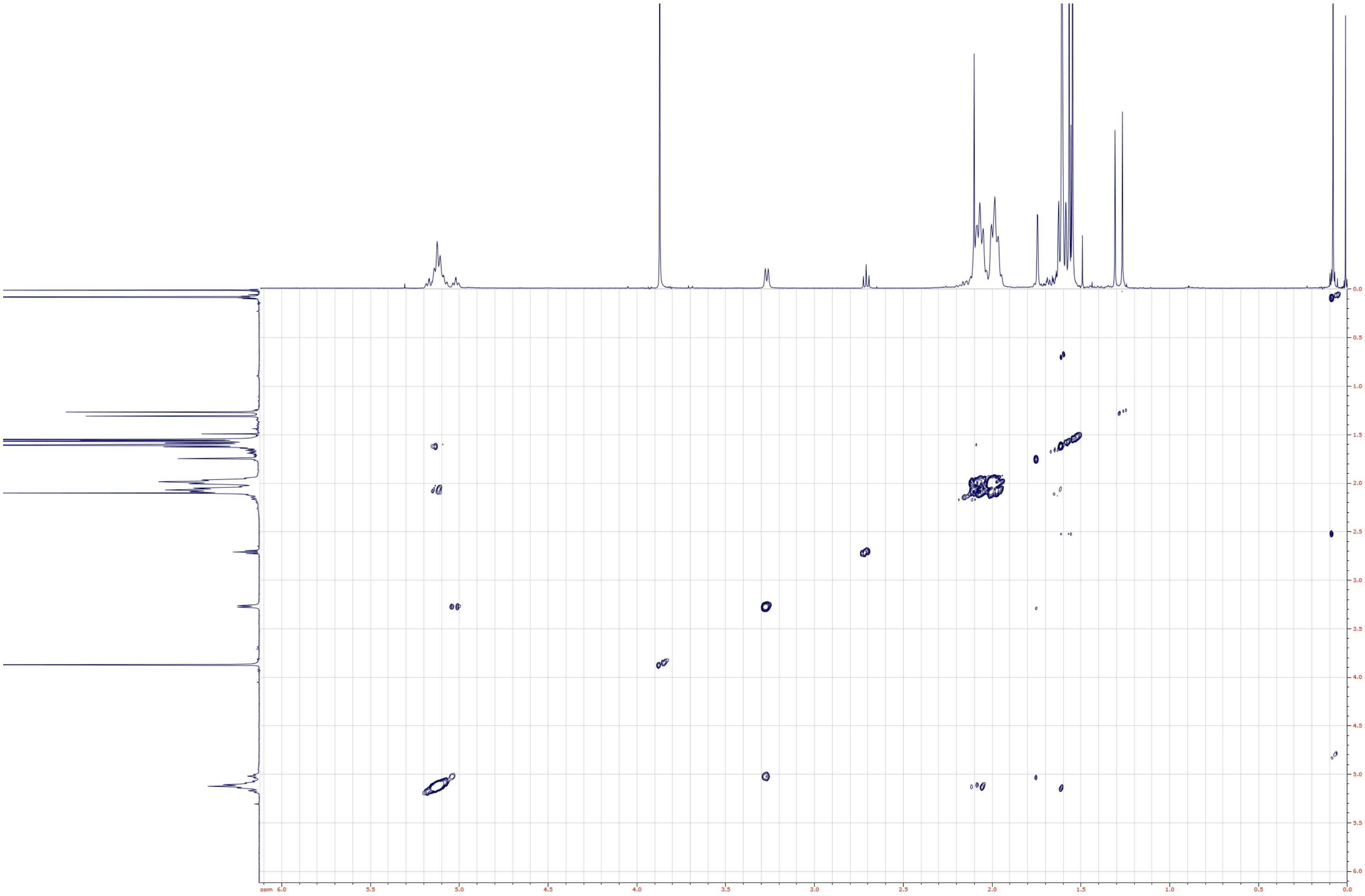

2D HSQC (400 MHZ, $CDCl_3$) for Boc_2-CoQ10-ω-epoxide (9)

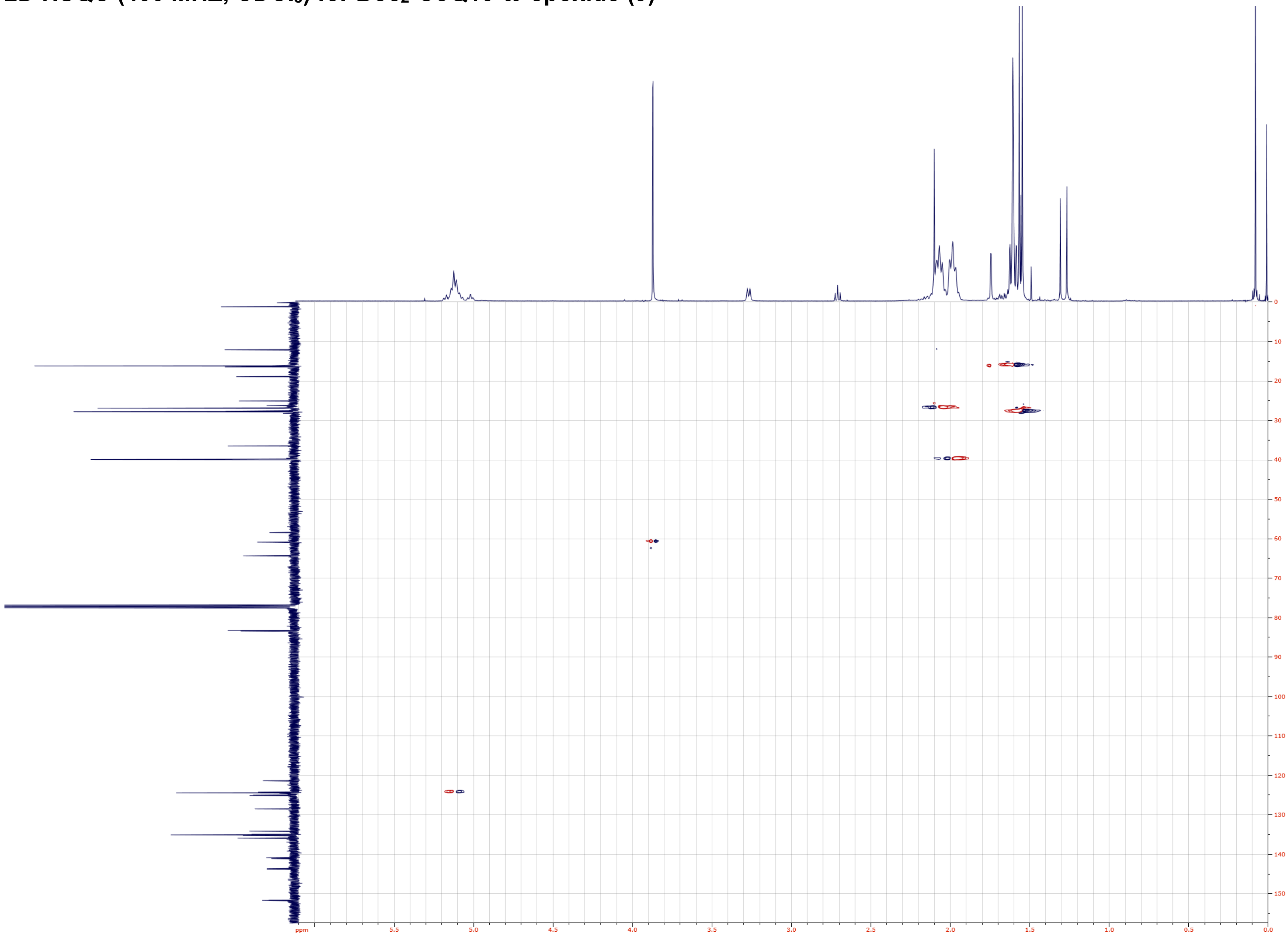

2D HMBC (400 MHZ, $CDCl_3$) for Boc_2-CoQ10-ω-epoxide (9)

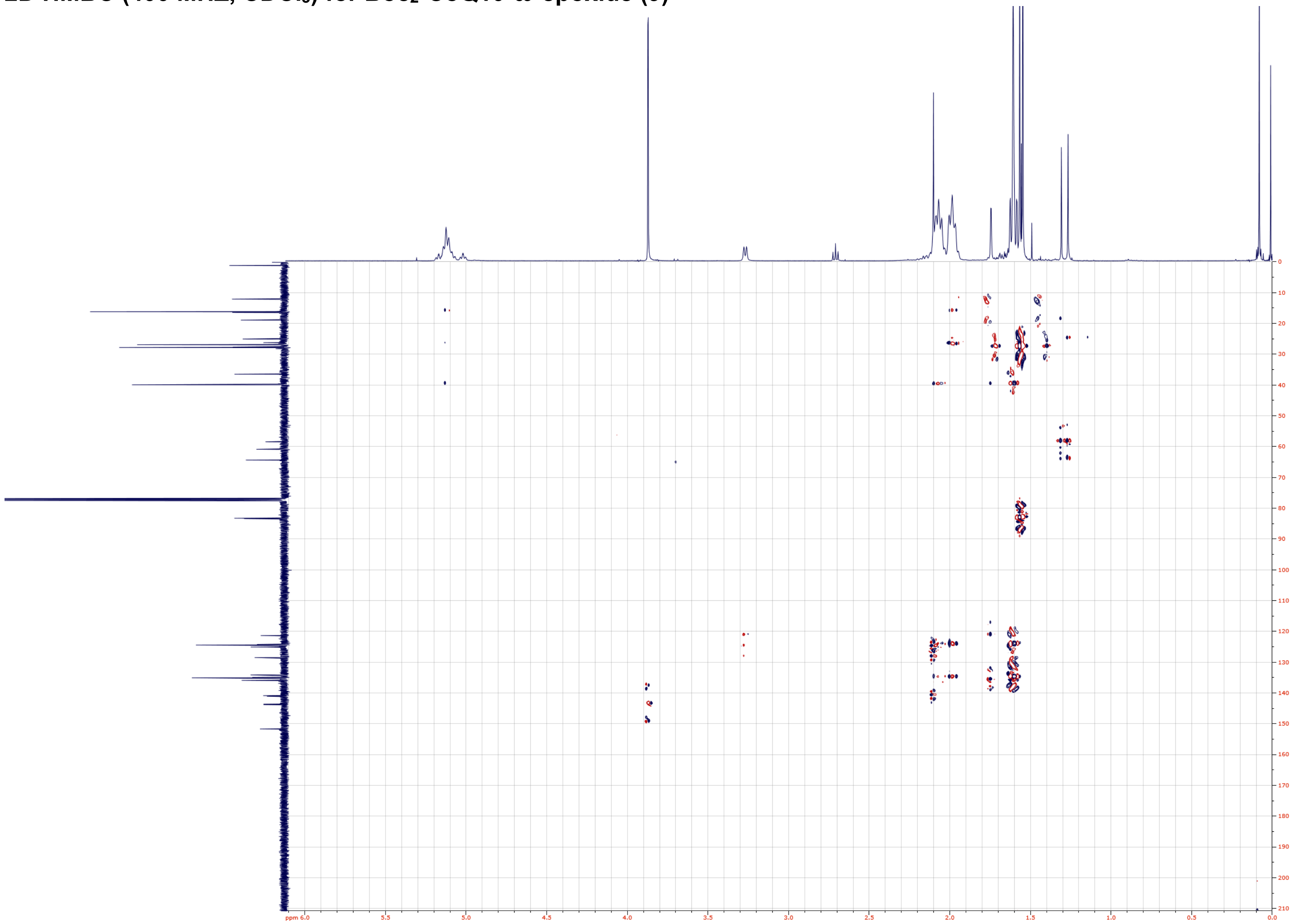

^{1}H NMR (400 MHZ, $CDCl_3$) for Boc_2-MK4-ω-epoxide (10)

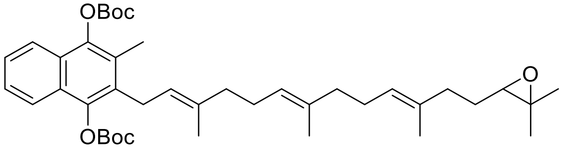

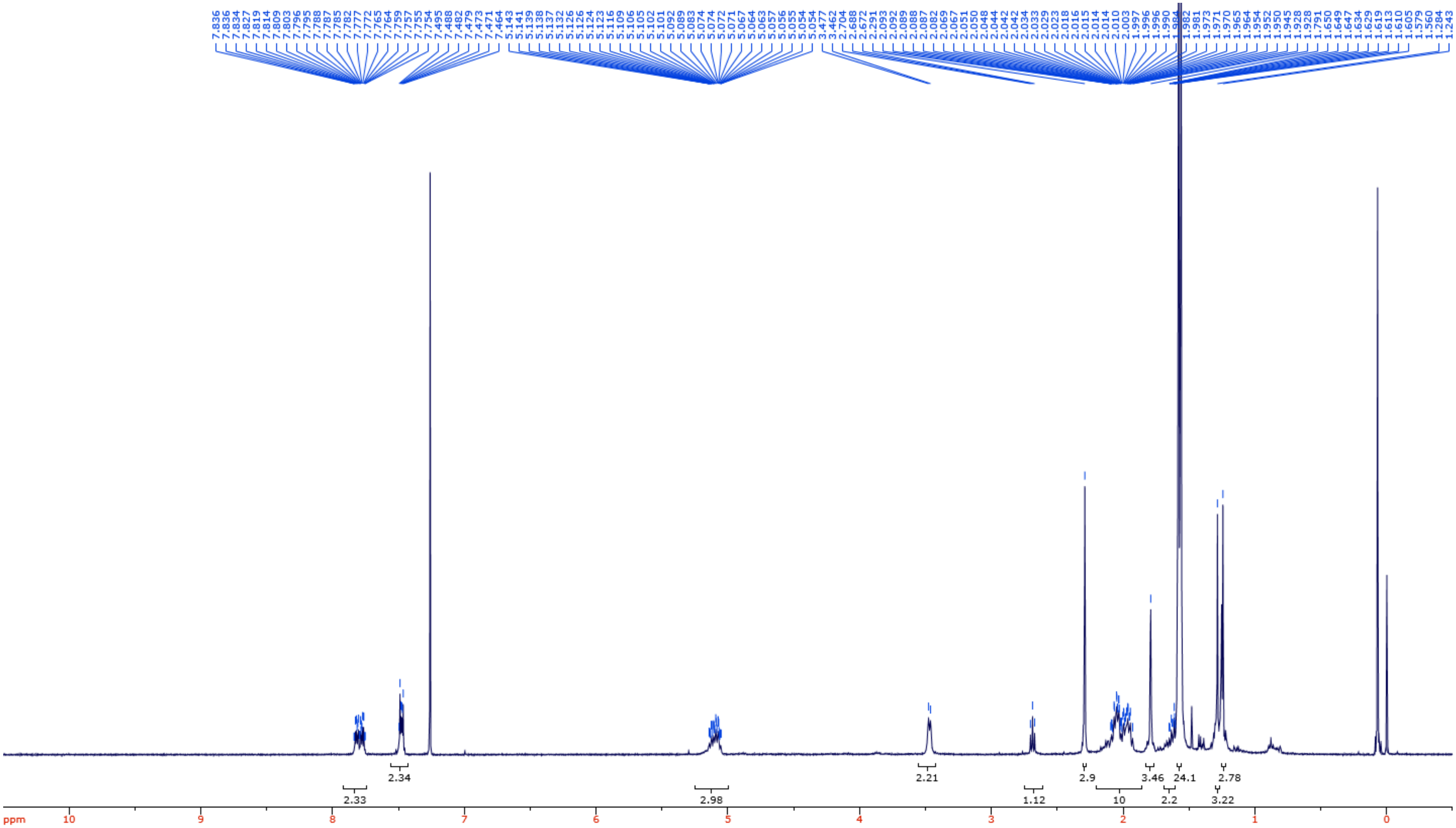

^{13}C NMR (101 MHZ, $CDCl_3$) for Boc_2-MK4-ω-epoxide (10)

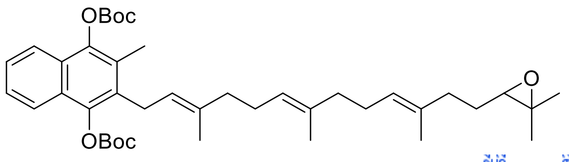

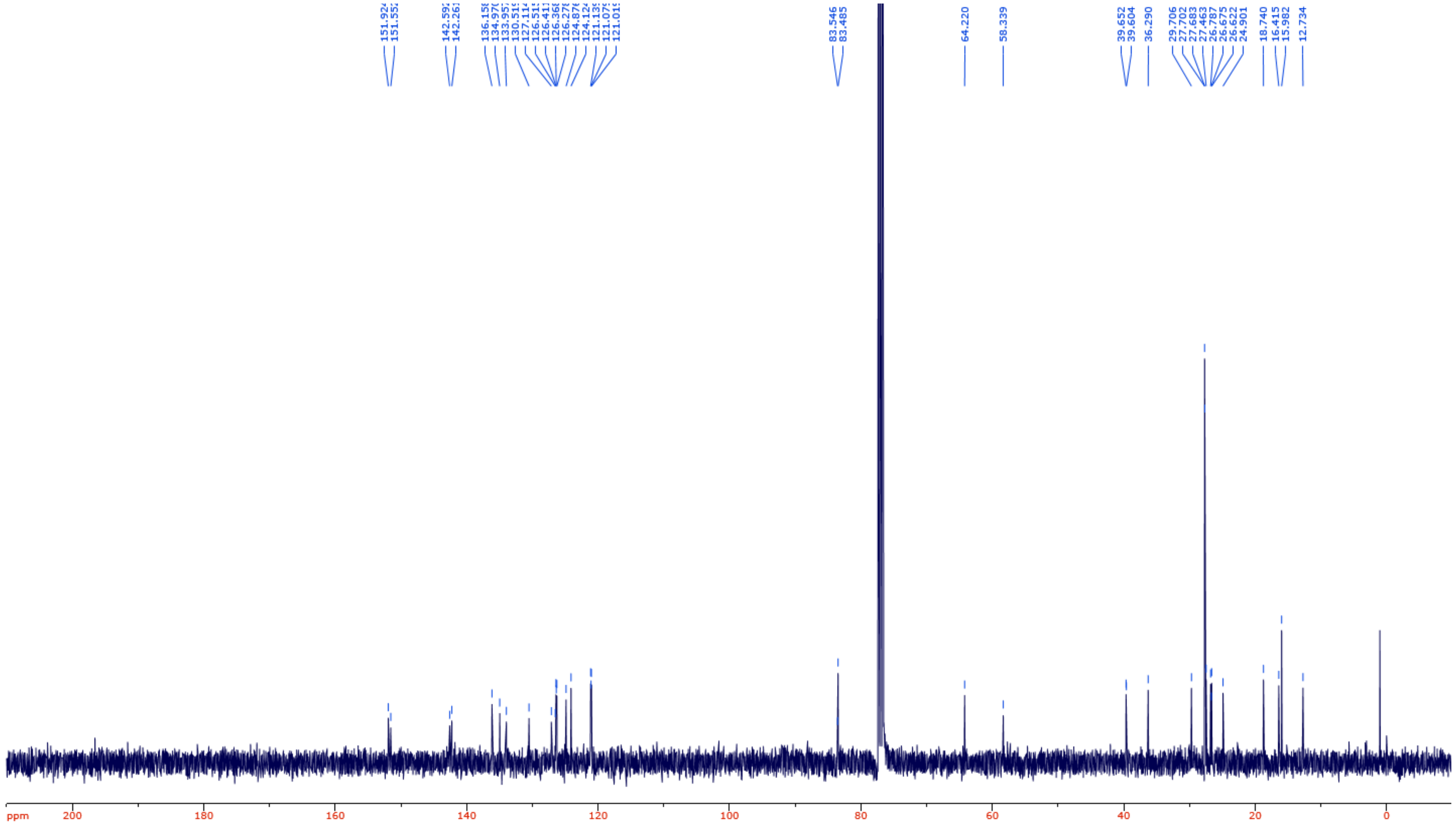

2D COSY (400 MHZ, $CDCl_3$) for Boc_2-MK4-ω-epoxide (10)

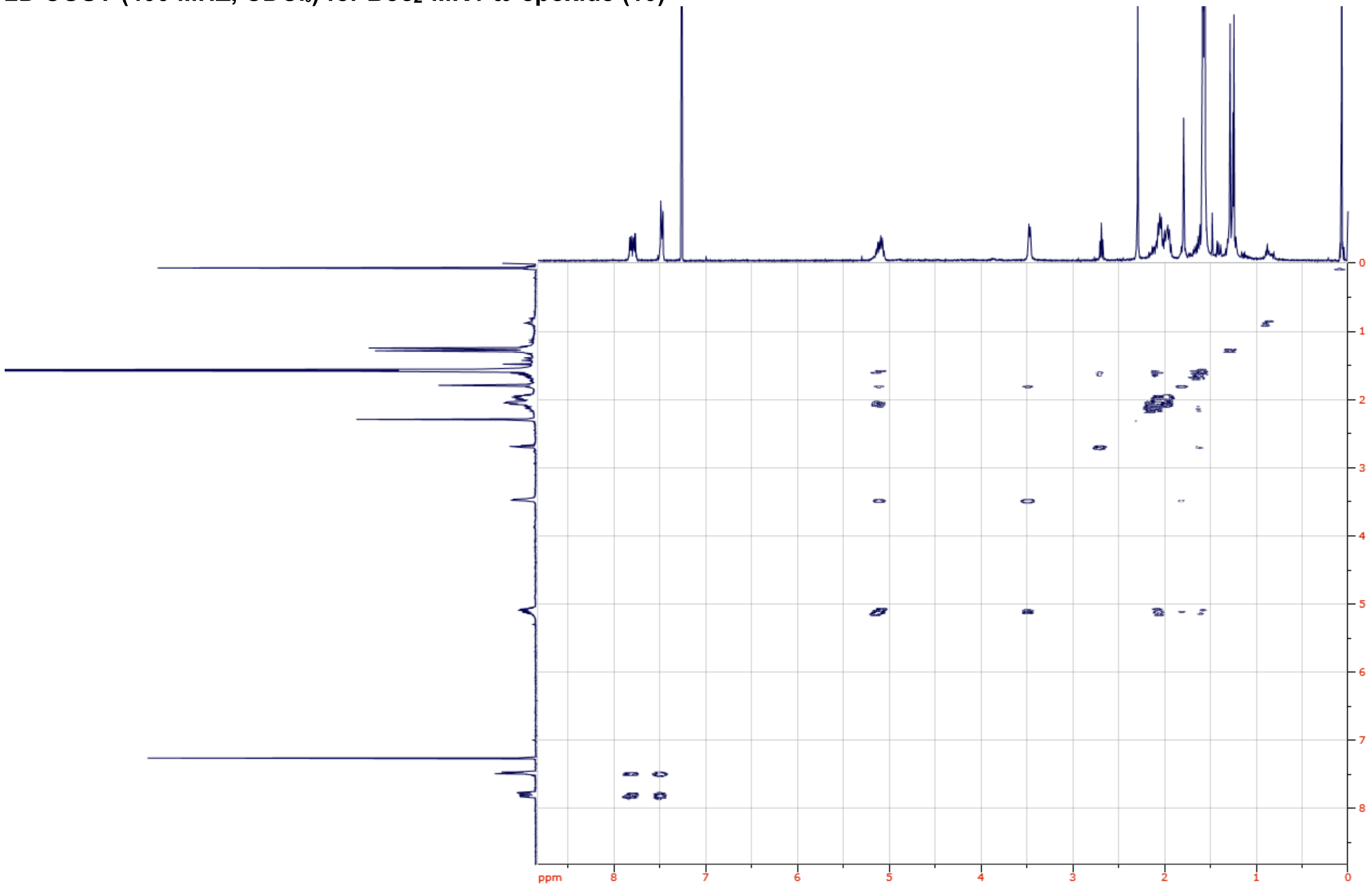

2D HSQC (400 MHZ, $CDCl_3$) for Boc_2-MK4-ω-epoxide (10)

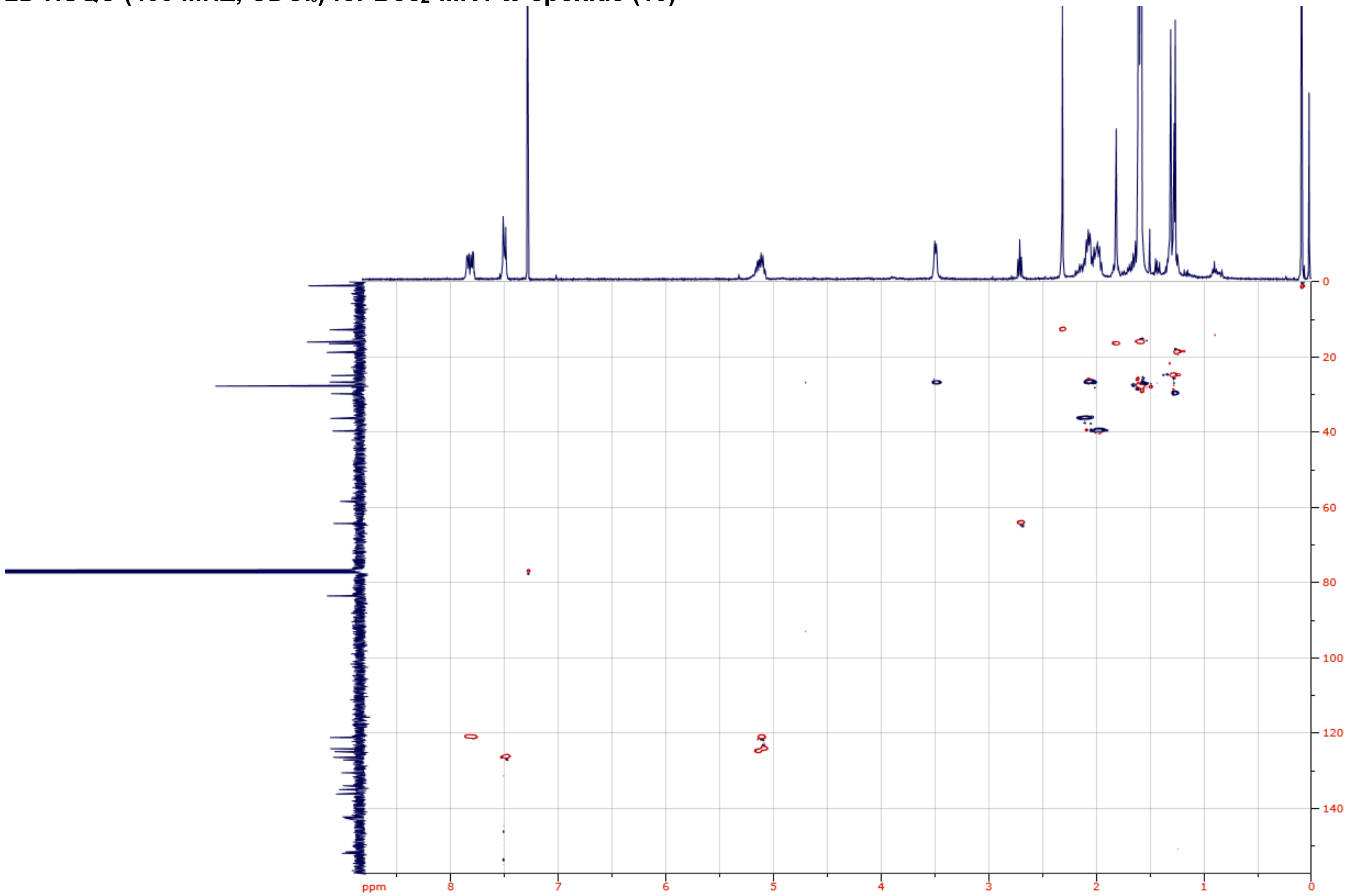

2D HMBC (400 MHZ, $CDCl_3$) for Boc_2-MK4-ω-epoxide (10)

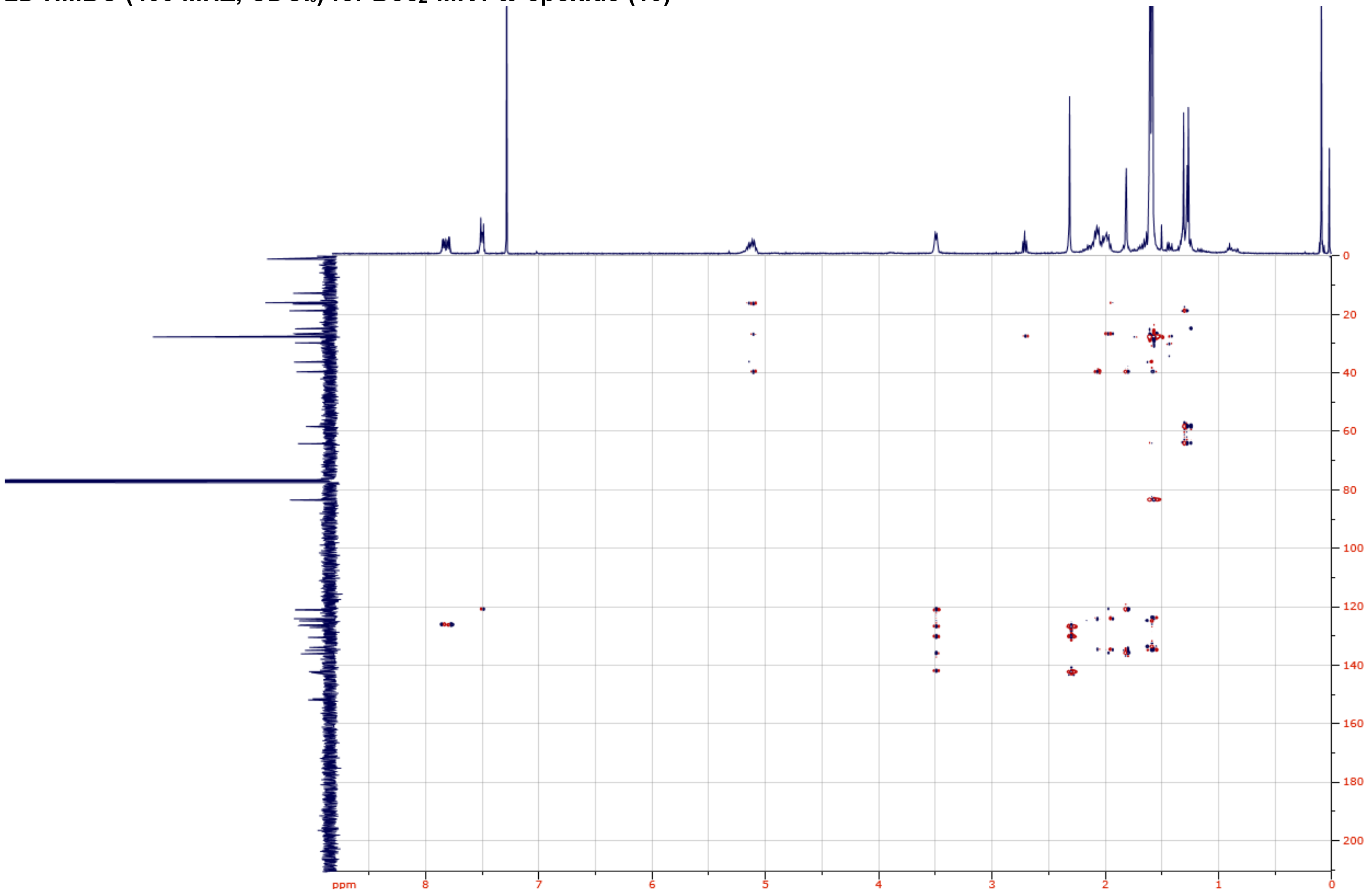

^{1}H NMR (400 MHZ, $CDCl_3$) for Boc_2-MK9-ω-epoxide (11)

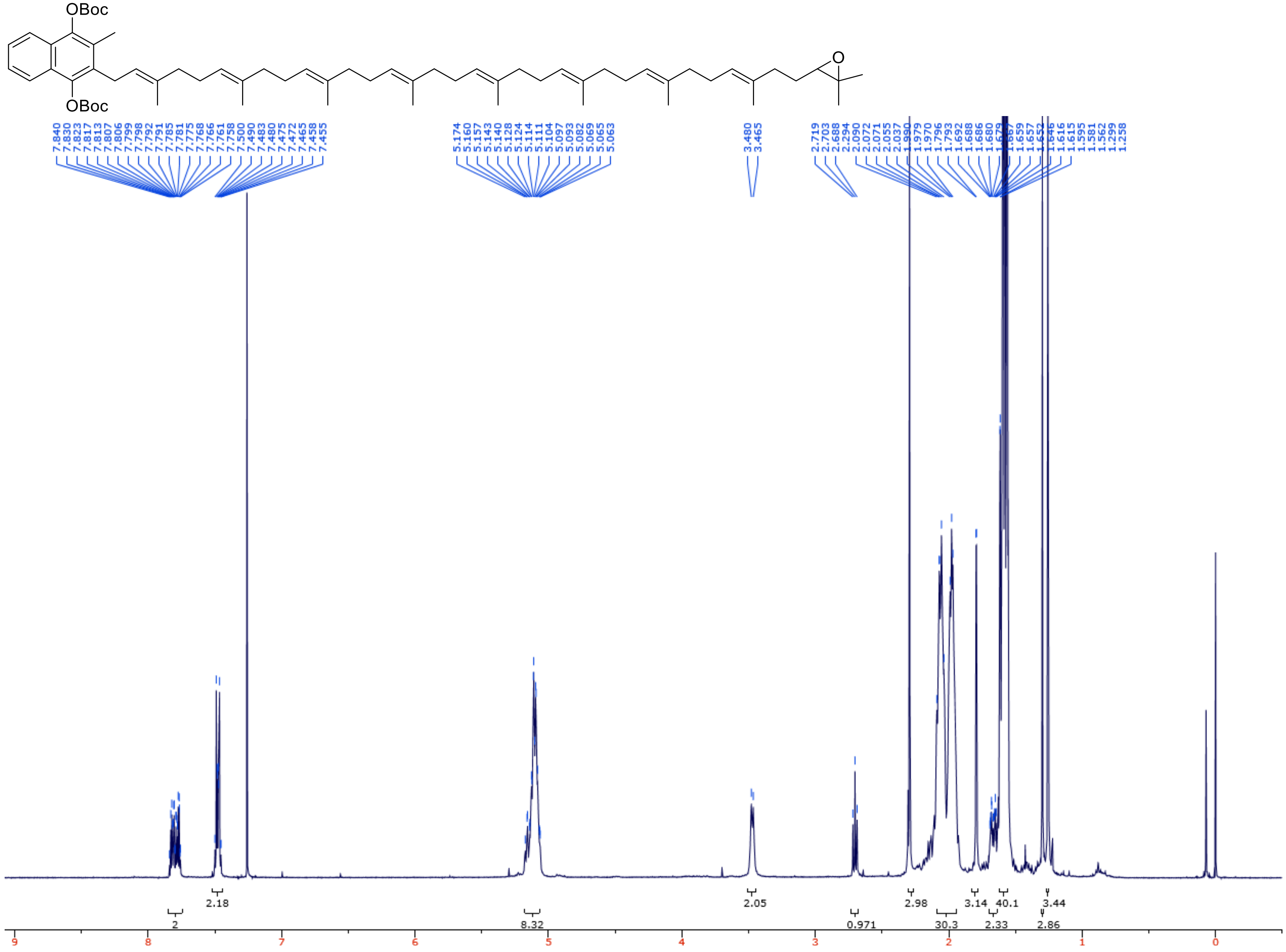

^{13}C NMR (101 MHZ, $CDCl_3$) for Boc_2-MK9-ω-epoxide (11)

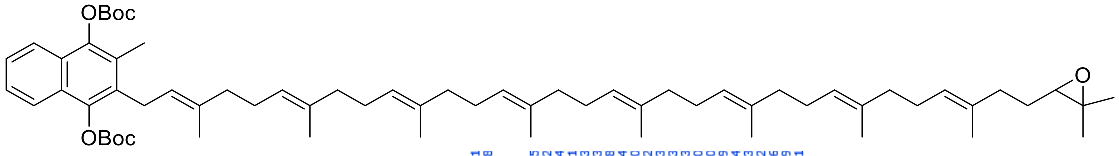

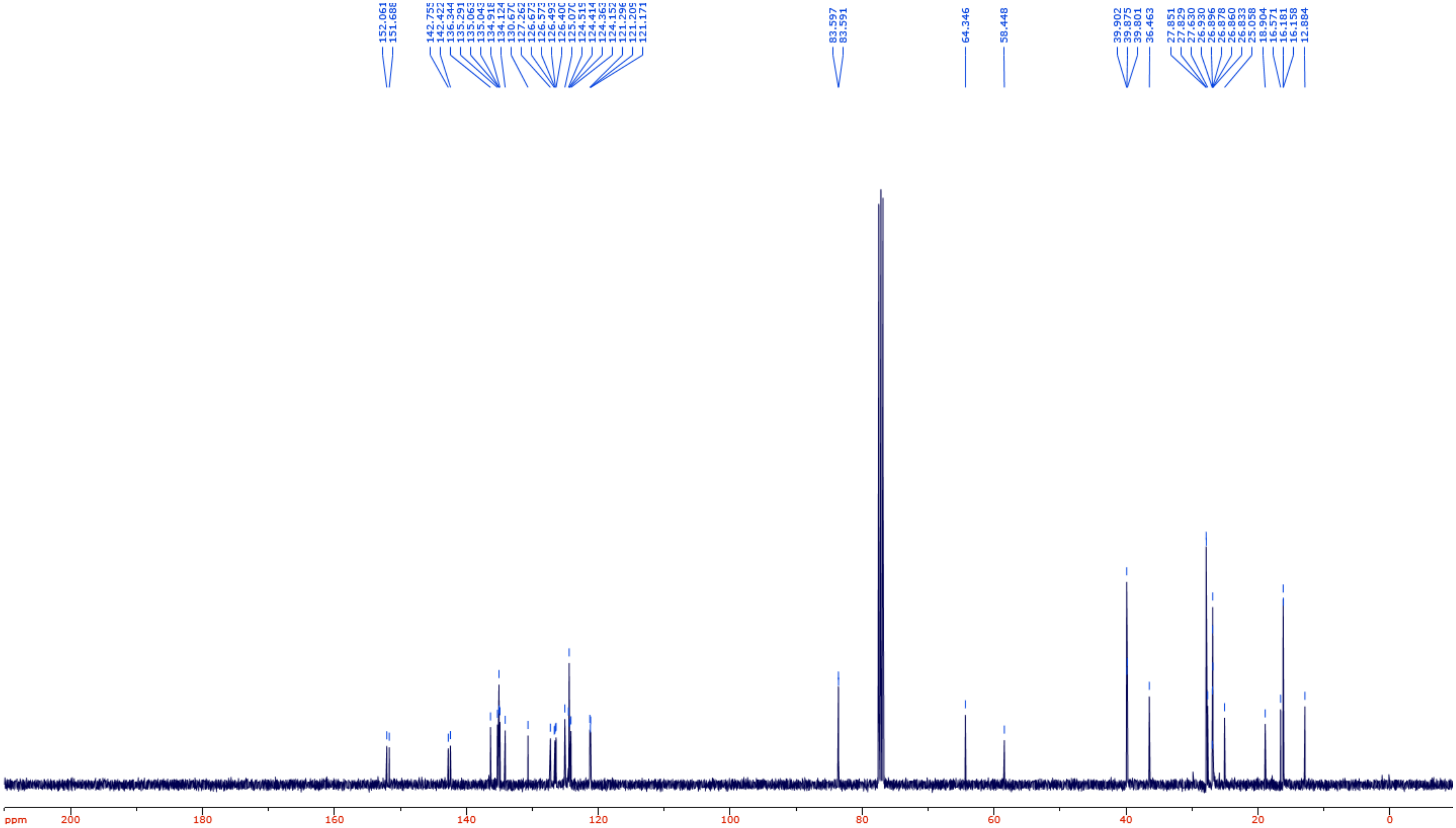

2D COSY (400 MHZ, $CDCl_3$) for Boc_2-MK9-ω-epoxide (11)

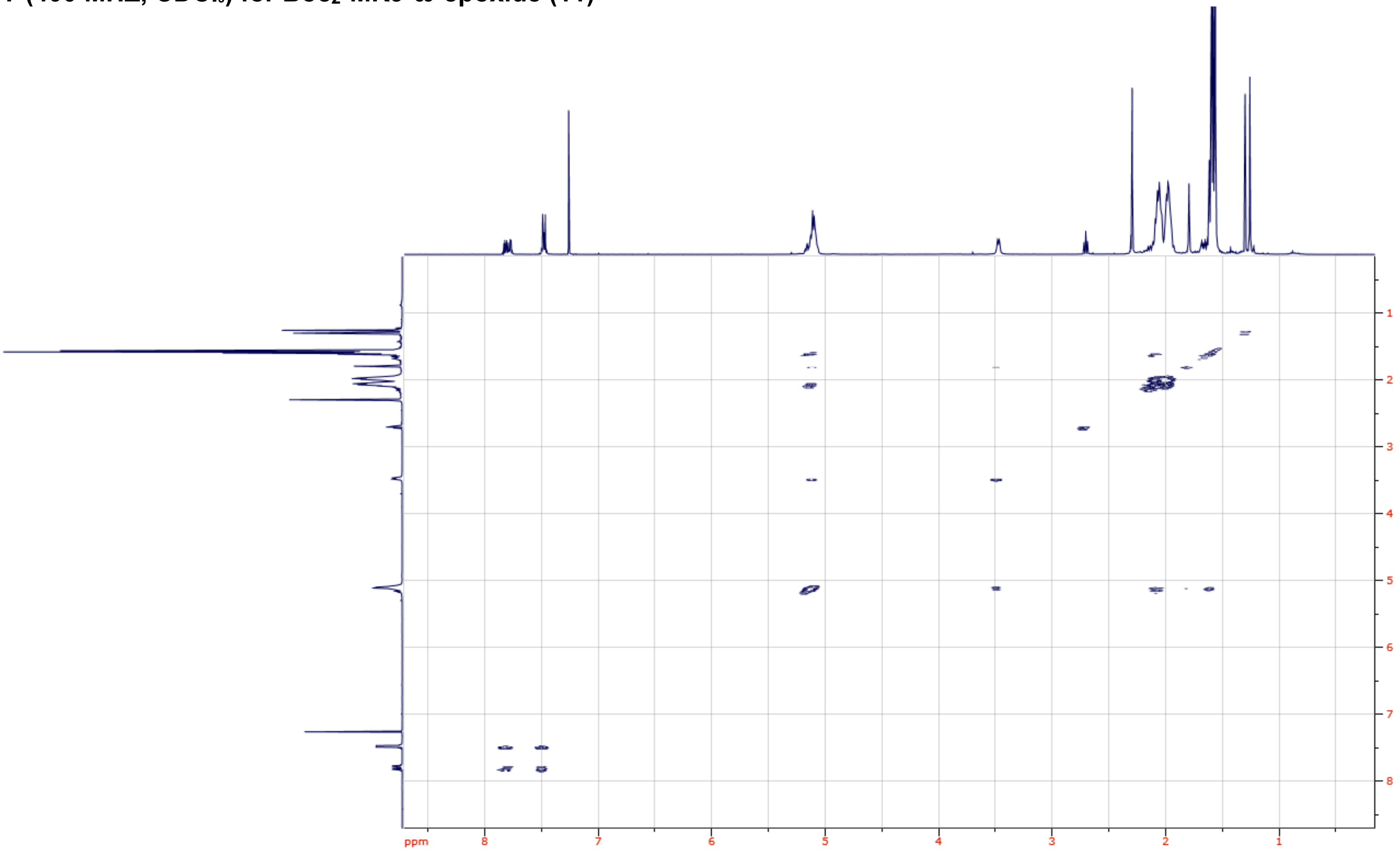

2D HSQC (400 MHZ, $CDCl_3$) for Boc_2-MK9-ω-epoxide (11)

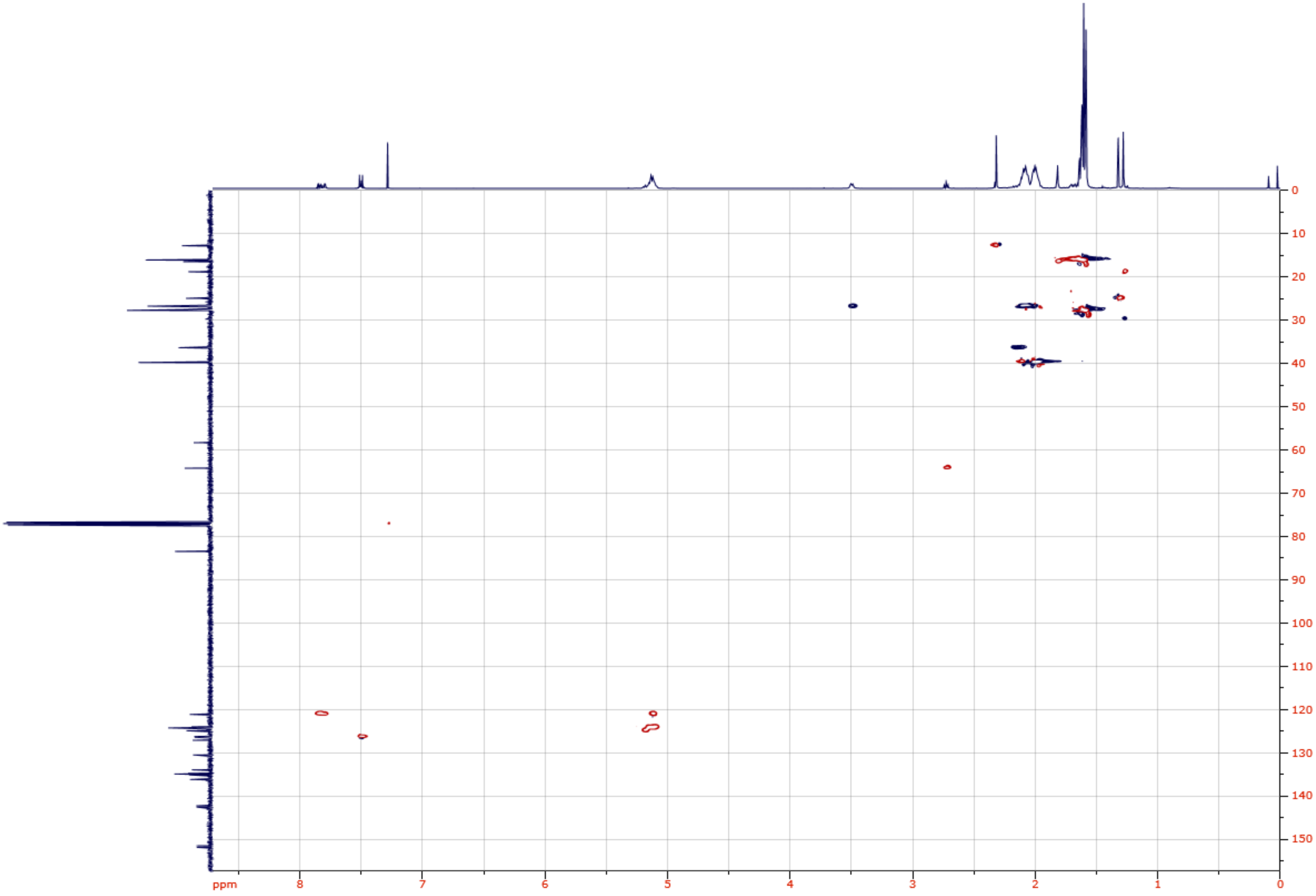

2D HMBC (400 MHZ, $CDCl_3$) for Boc_2-MK9-ω-epoxide (11)

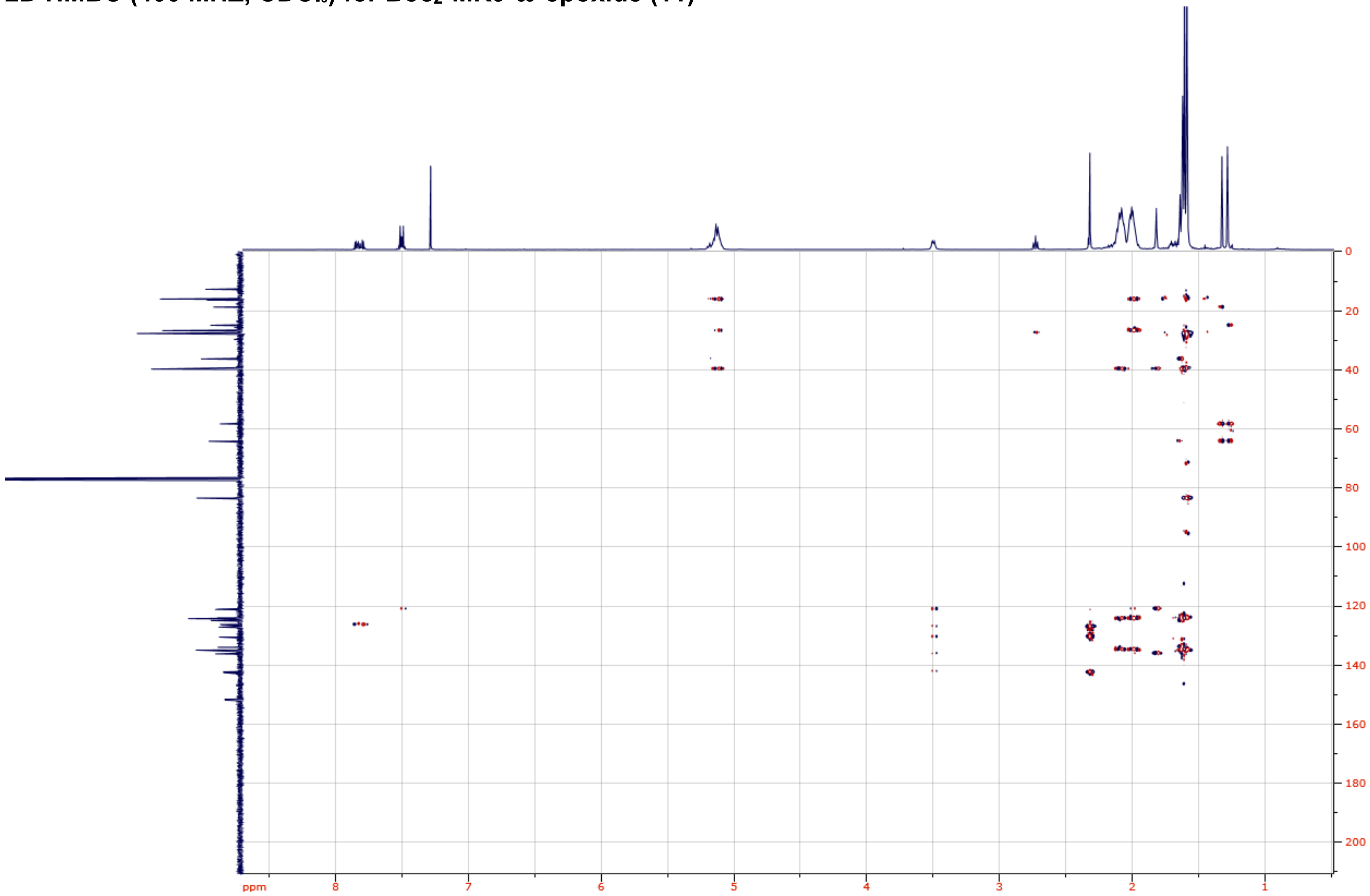

^{1}H NMR (400 MHZ, $CDCl_3$) for Me_2-CoQ10-ω-2-AB (12)

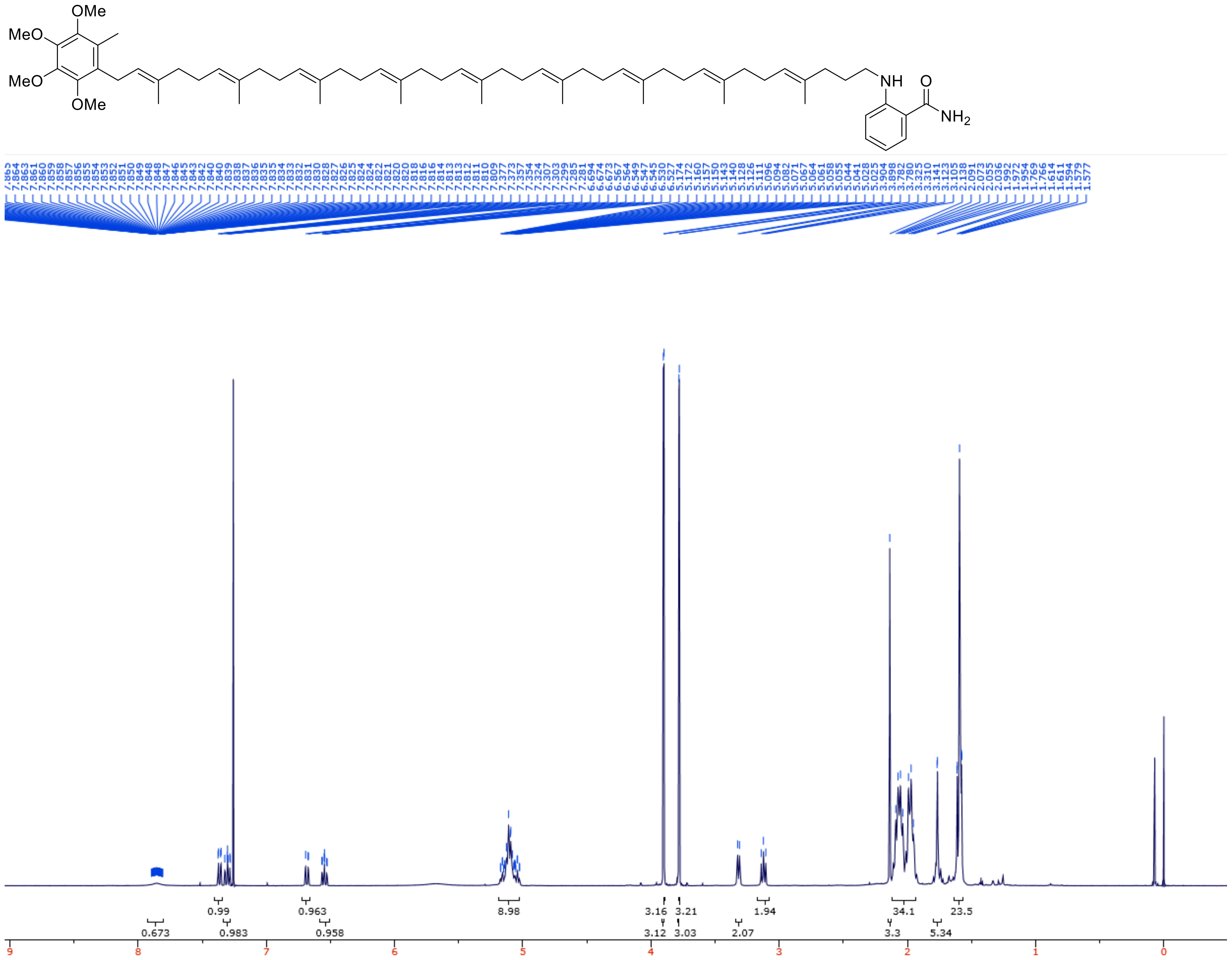

^{13}C NMR (101 MHZ, $CDCl_3$) for Me_2-CoQ10-ω-2-AB (12)

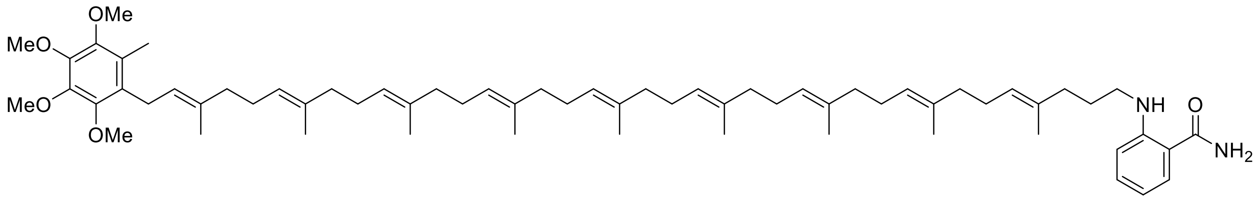

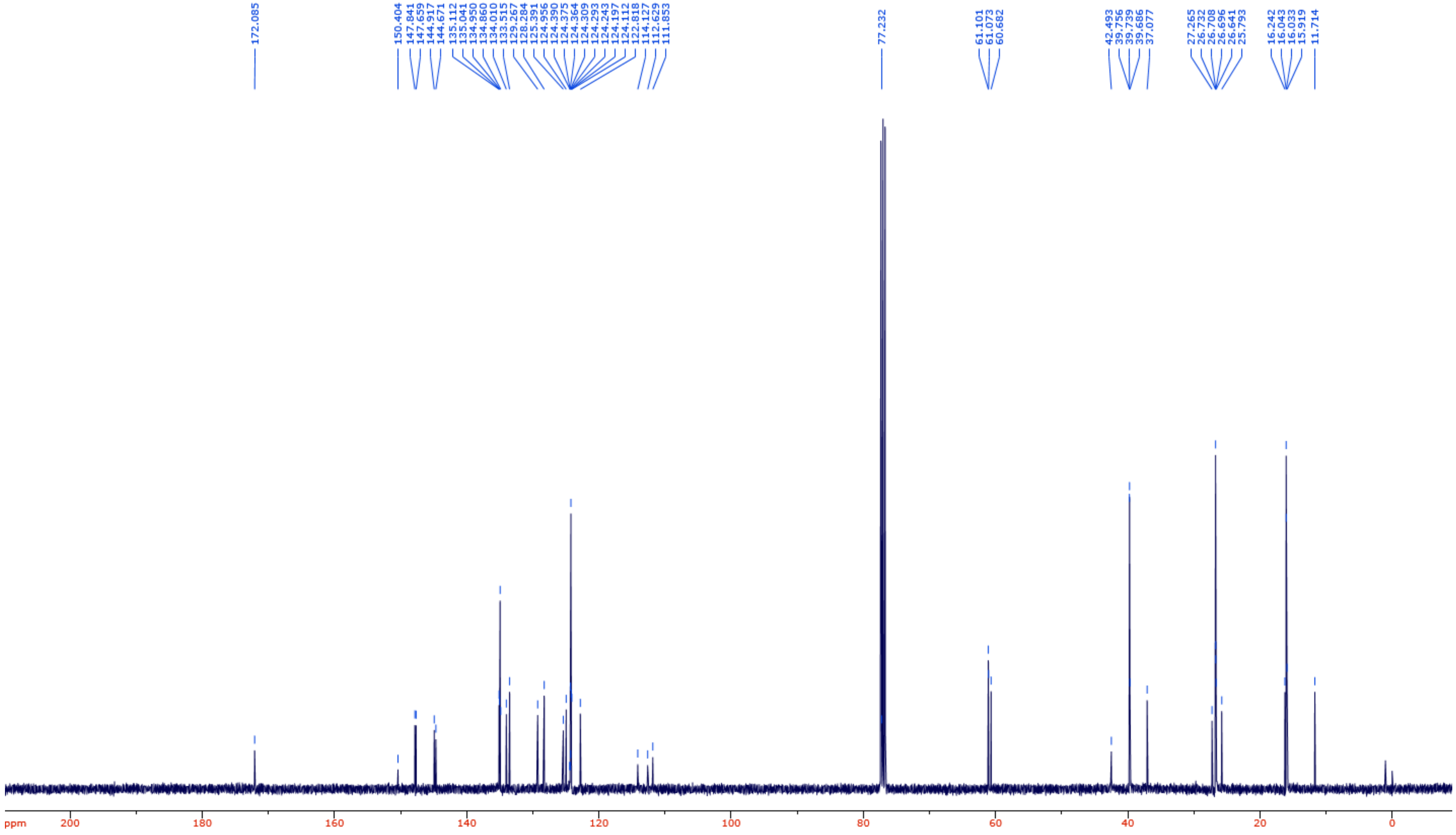

2D COSY (400 MHZ, $CDCl_3$) for Me_2-CoQ10-ω-2-AB (12)

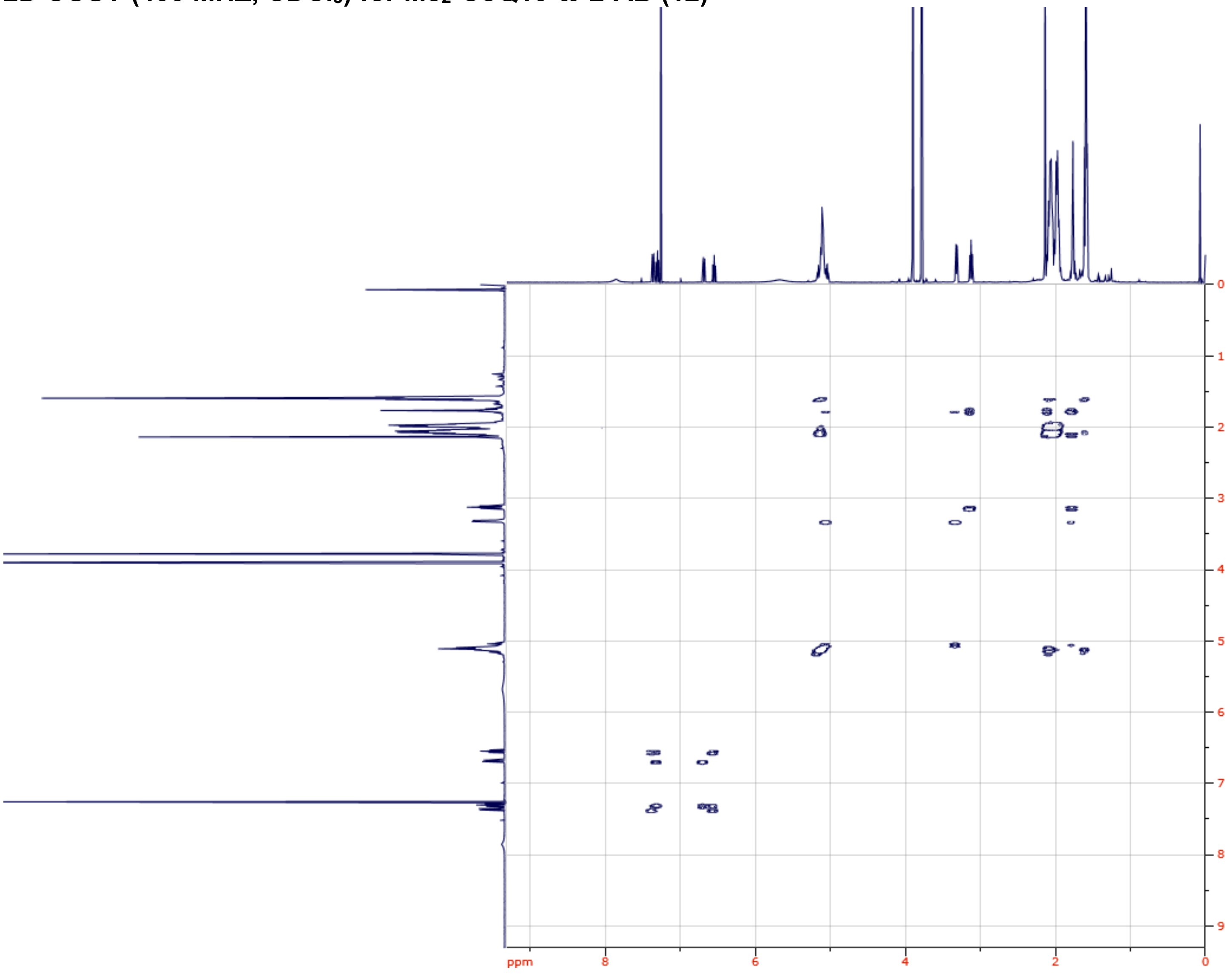

2D HSQC (400 MHZ, $CDCl_3$) for Me_2-CoQ10-ω-2-AB (12)

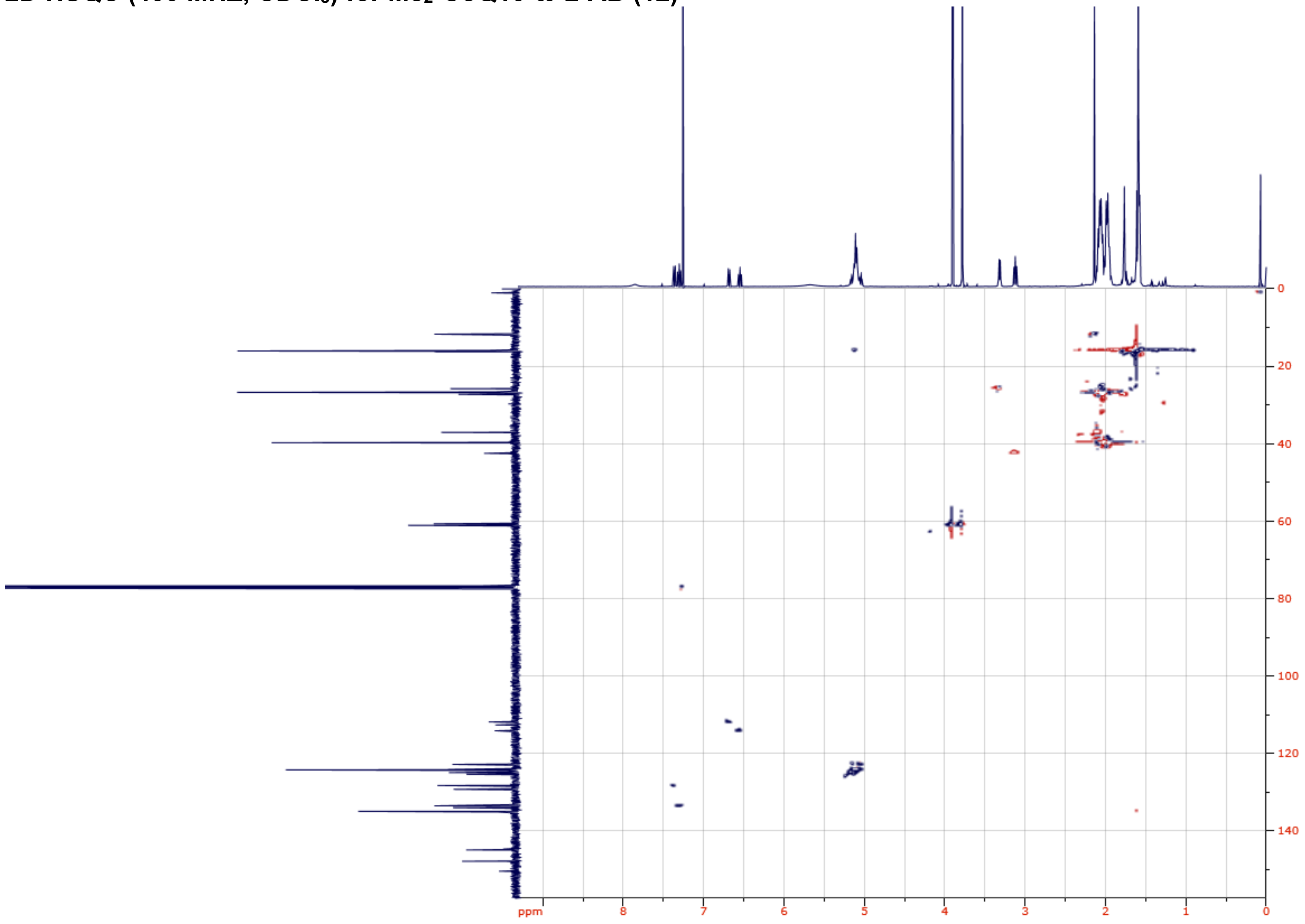

2D HMBC (400 MHZ, $CDCl_3$) for Me_2-CoQ10-ω-2-AB (12)

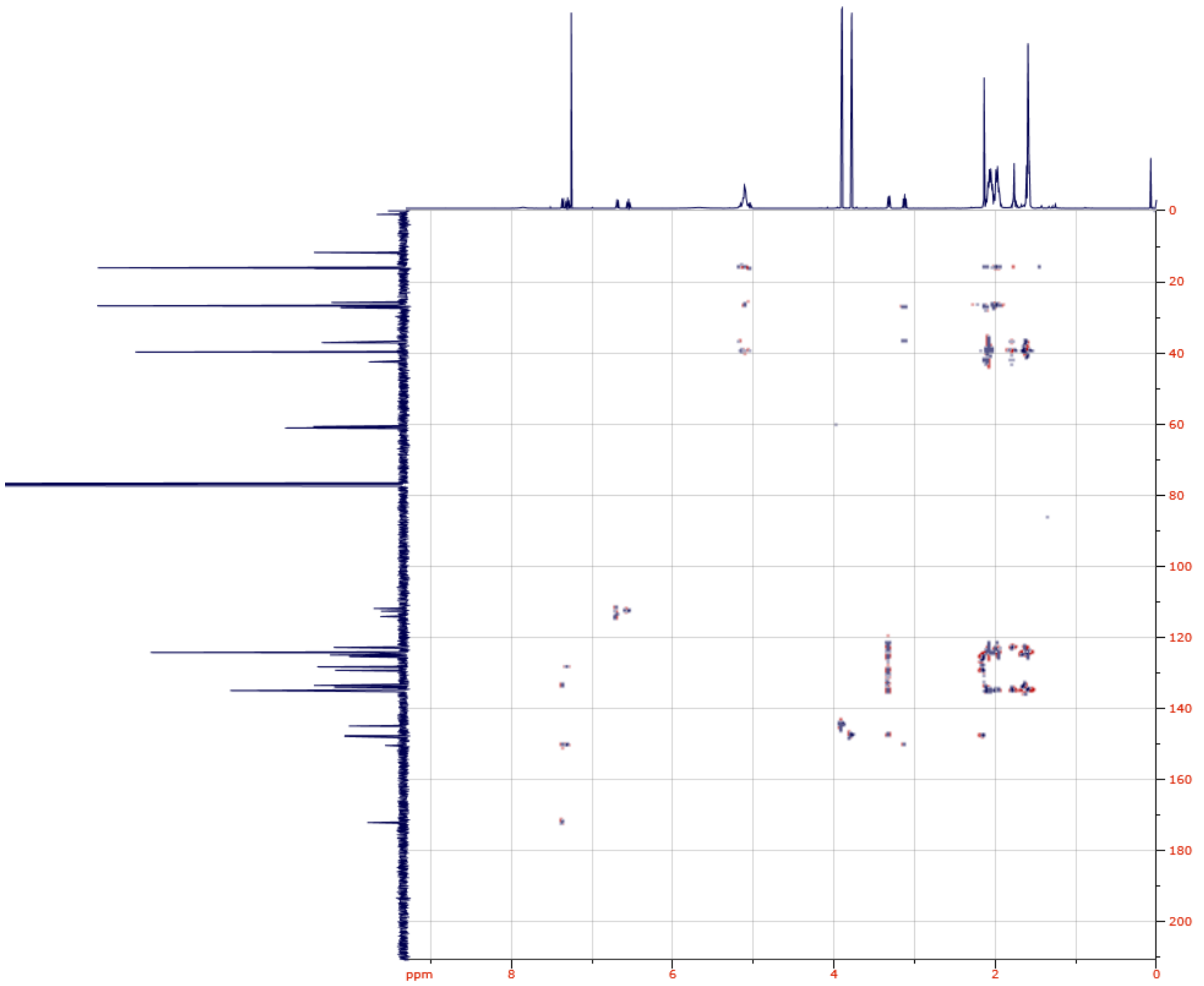

^{1}H NMR (400 MHZ, $CDCl_3$) for Boc_2-CoQ10-ω-2-AB (13)

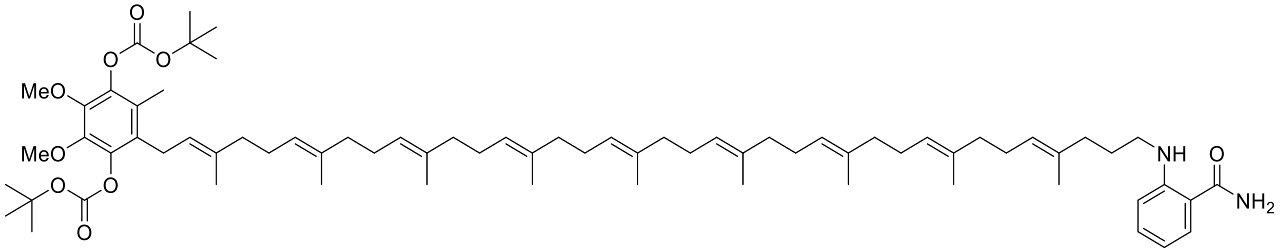

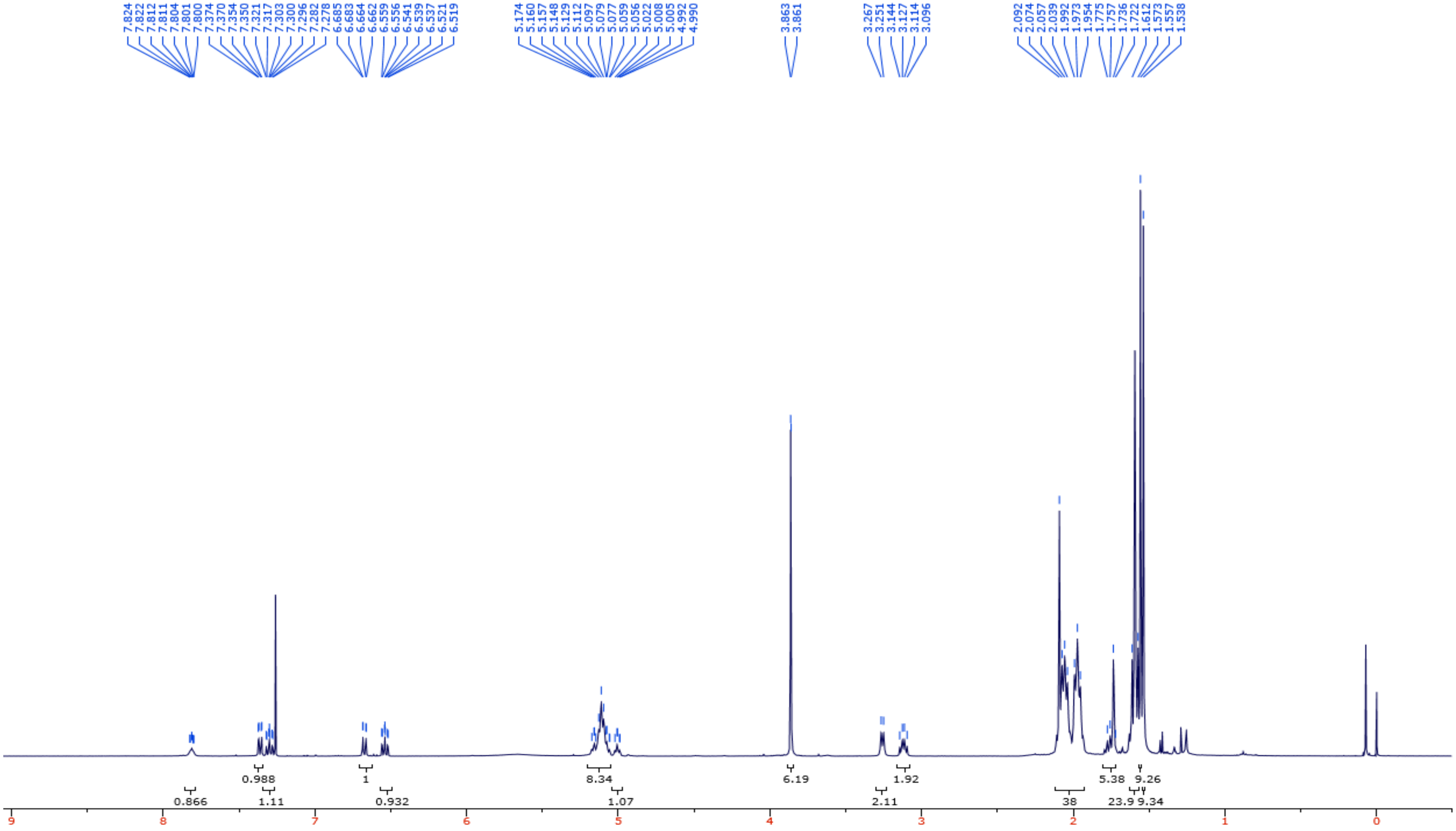

^{13}C NMR (101 MHZ, $CDCl_3$) for Boc_2-CoQ10-ω-2-AB (13)

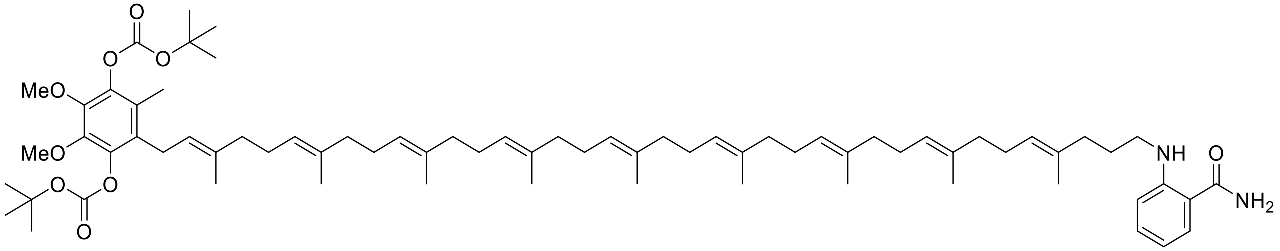

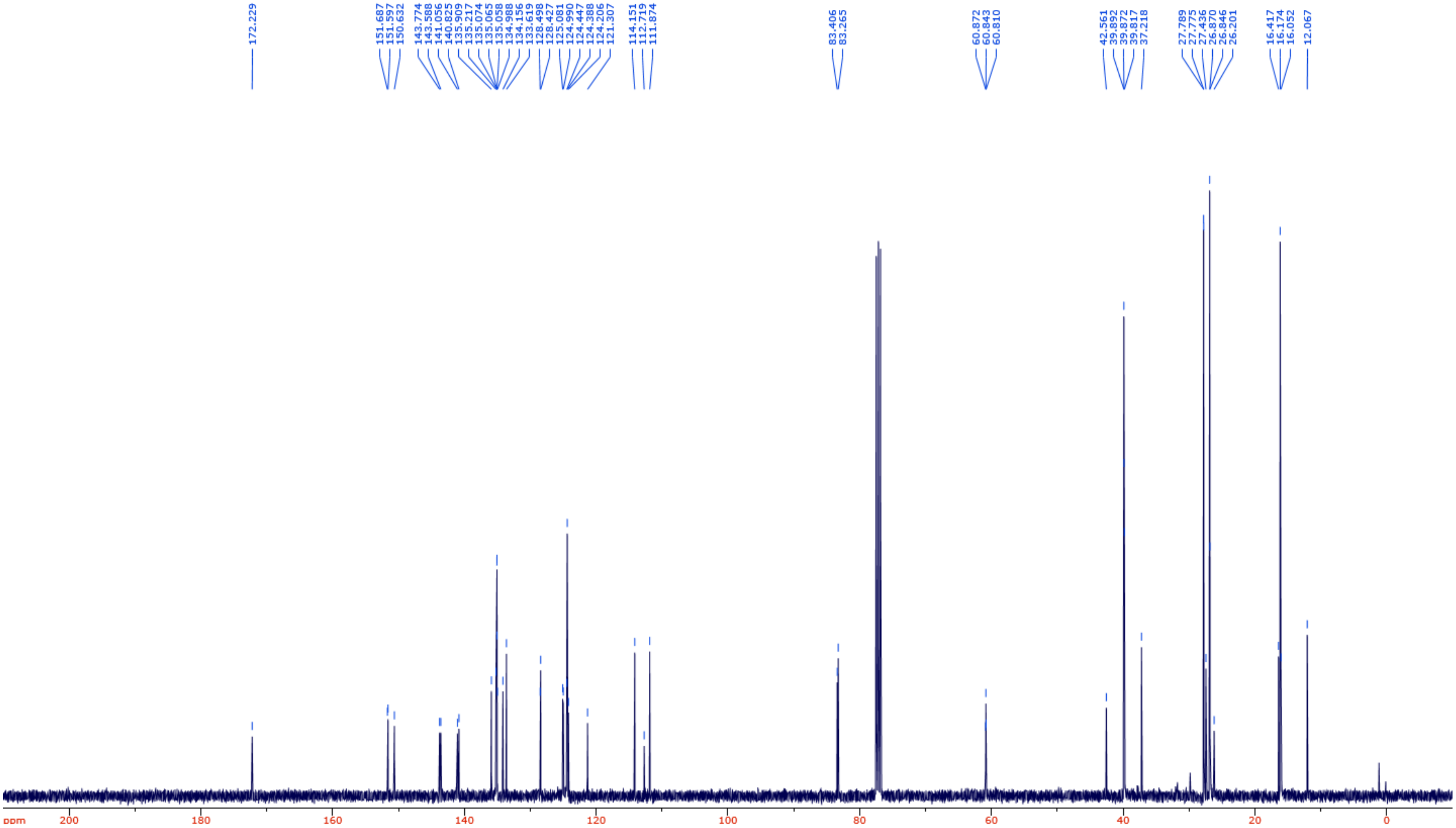

2D COSY (400 MHZ, $CDCl_3$) for Boc_2-CoQ10-ω-2-AB (13)

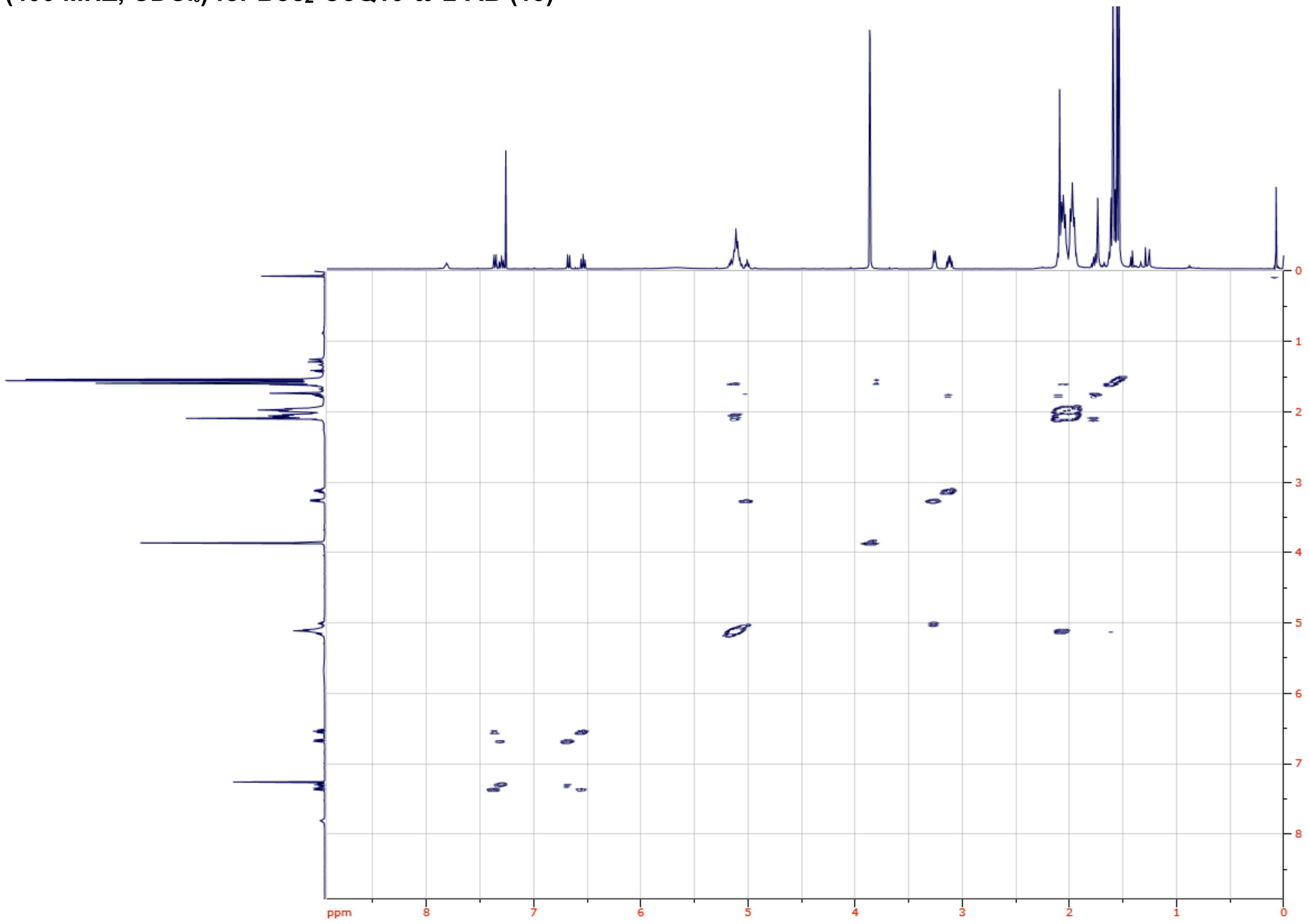

2D HSQC (400 MHZ, $CDCl_3$) for Boc_2-CoQ10-ω-2-AB (13)

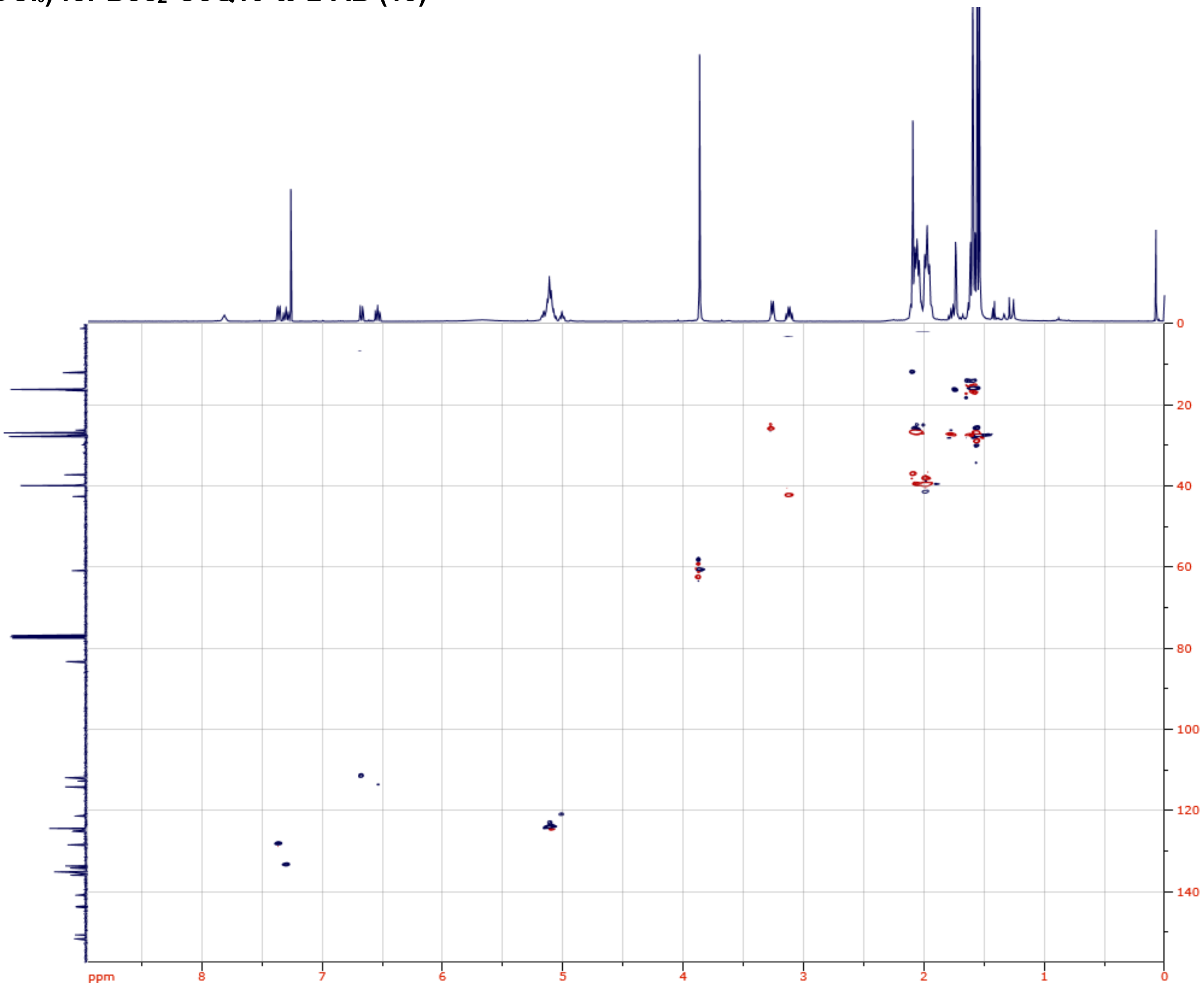

2D HMBC (400 MHZ, $CDCl_3$) for Boc_2-CoQ10-ω-2-AB (13)

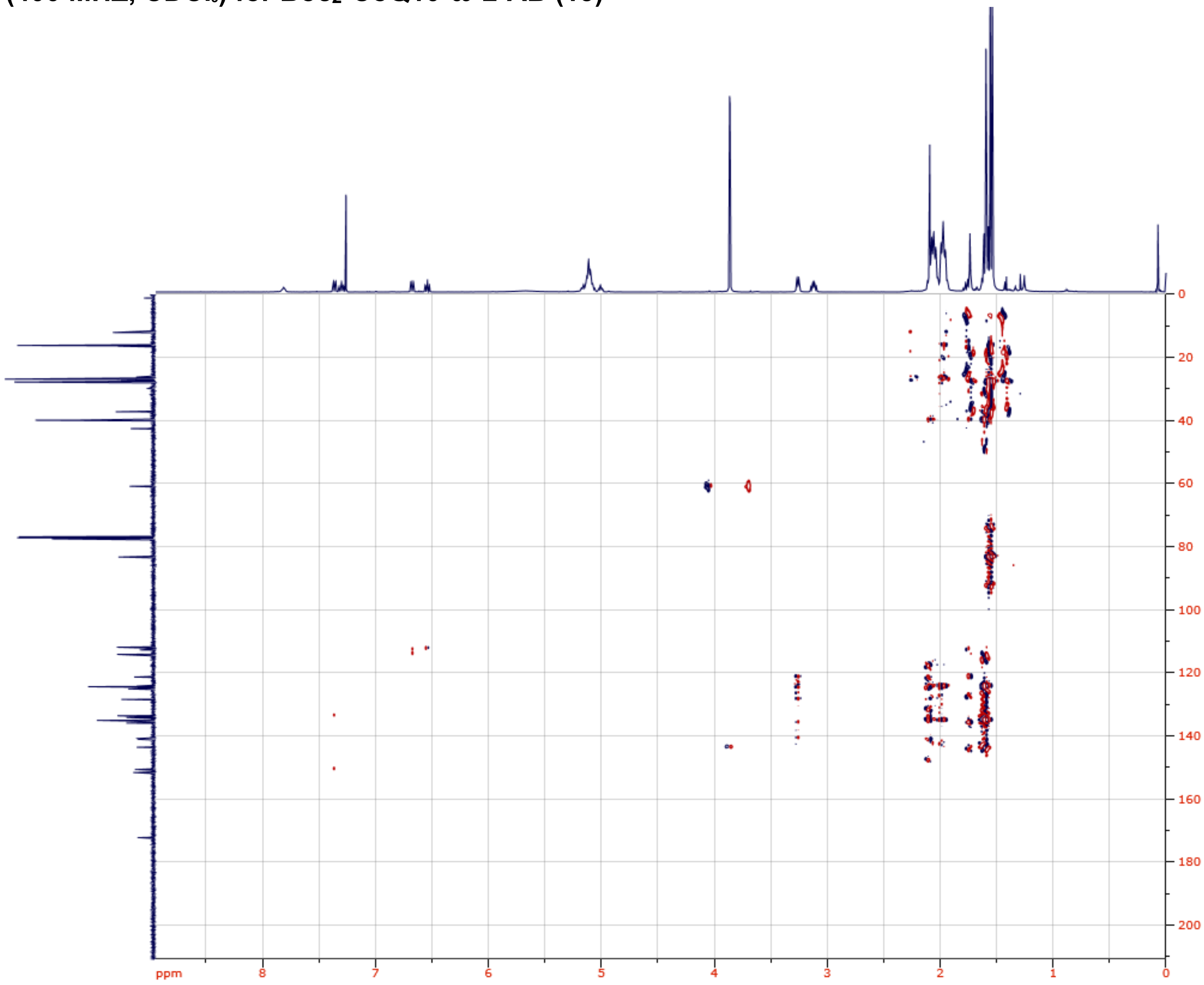

^{1}H-NMR (400 MHZ, $CDCl_3$) for Boc_2-MK4-ω-2-AB (14)

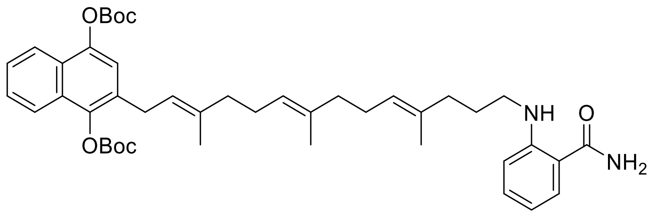

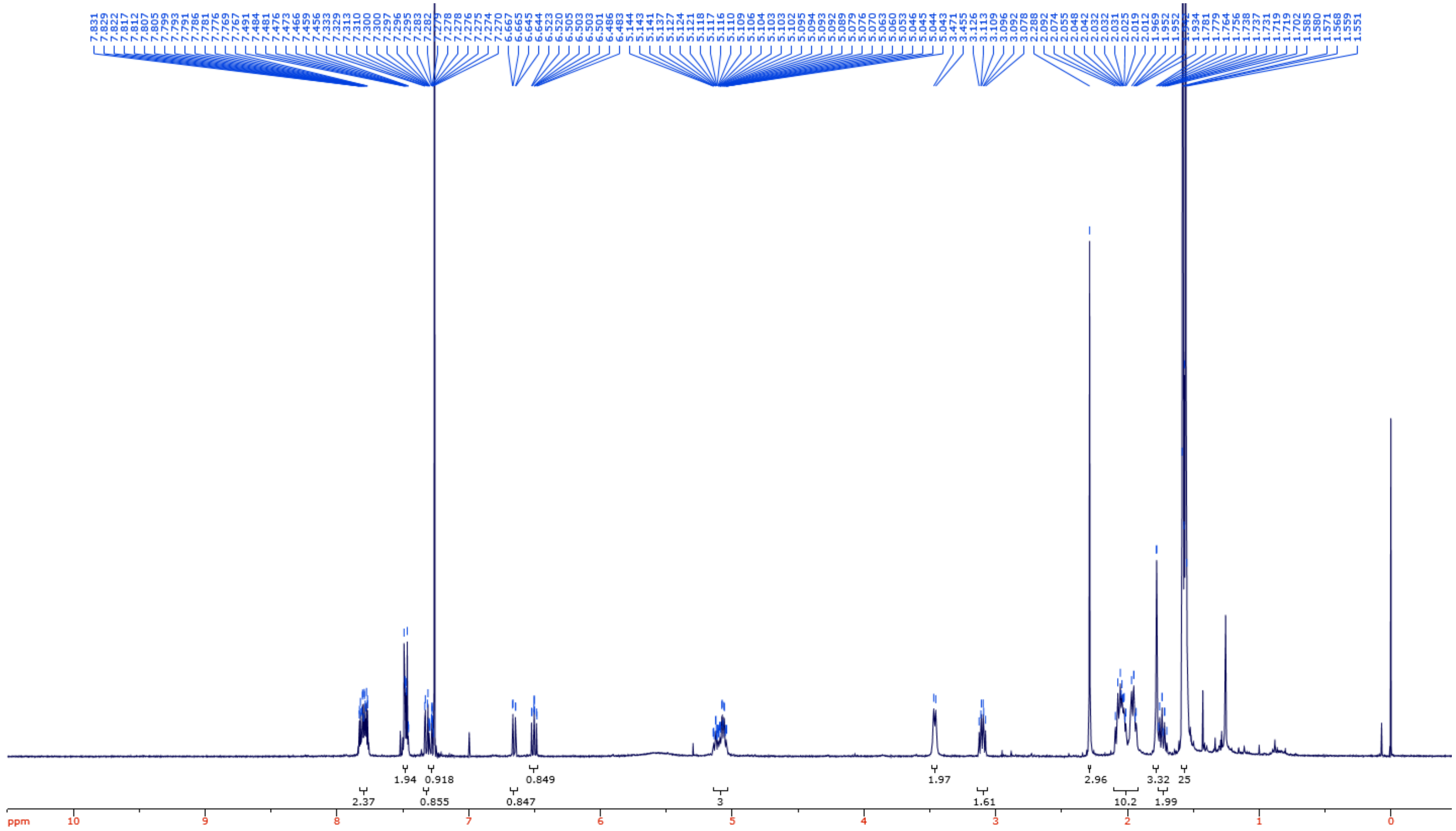

^{13}C NMR (400 MHZ, $CDCl_3$) for Boc_2-MK4-ω-2-AB (14)

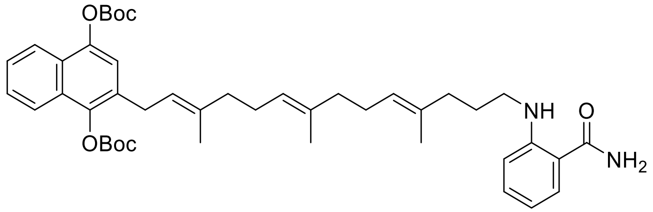

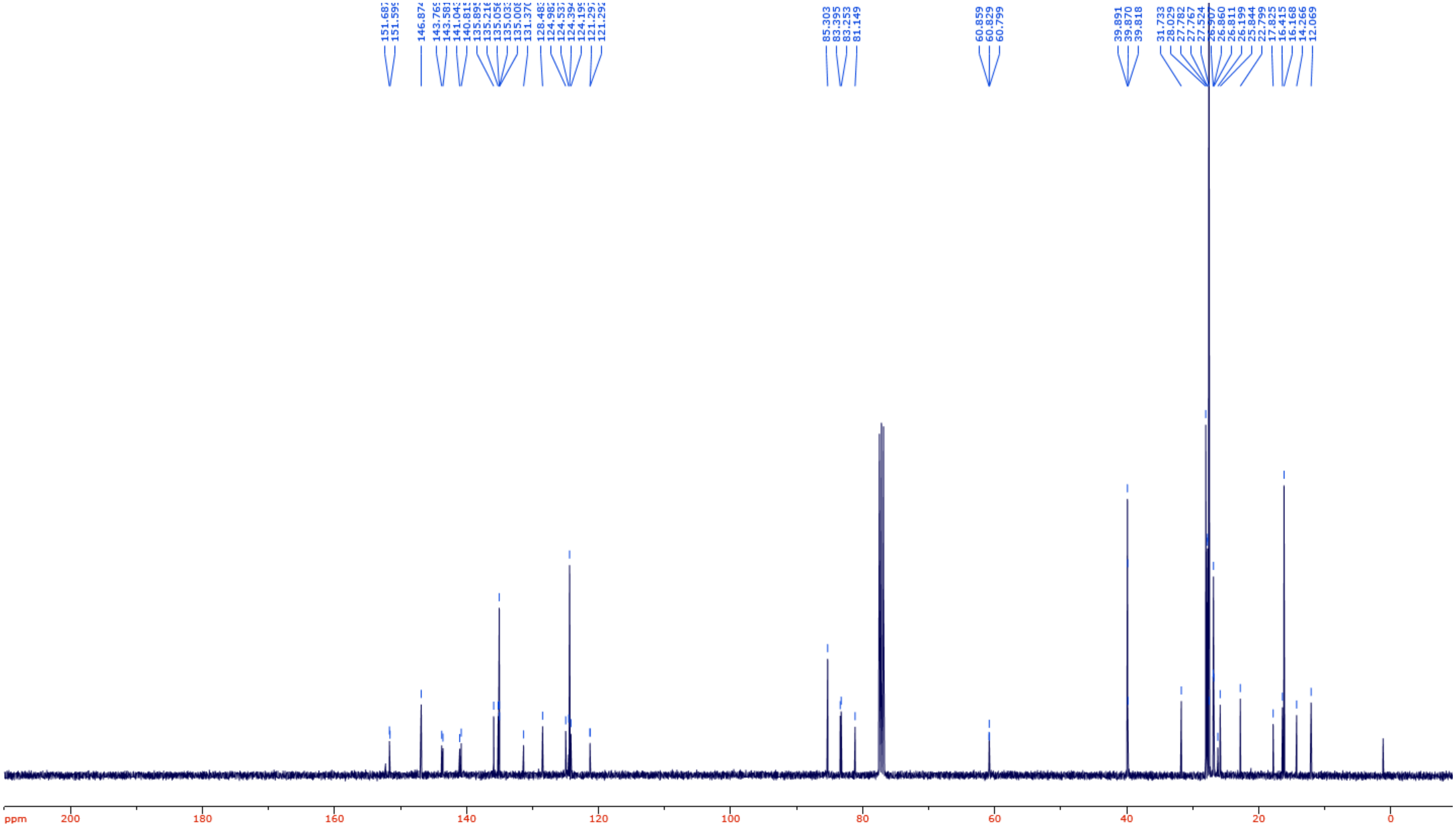

2D COSY (400 MHZ, $CDCl_3$) for for Boc_2-MK4-ω-2-AB (14)

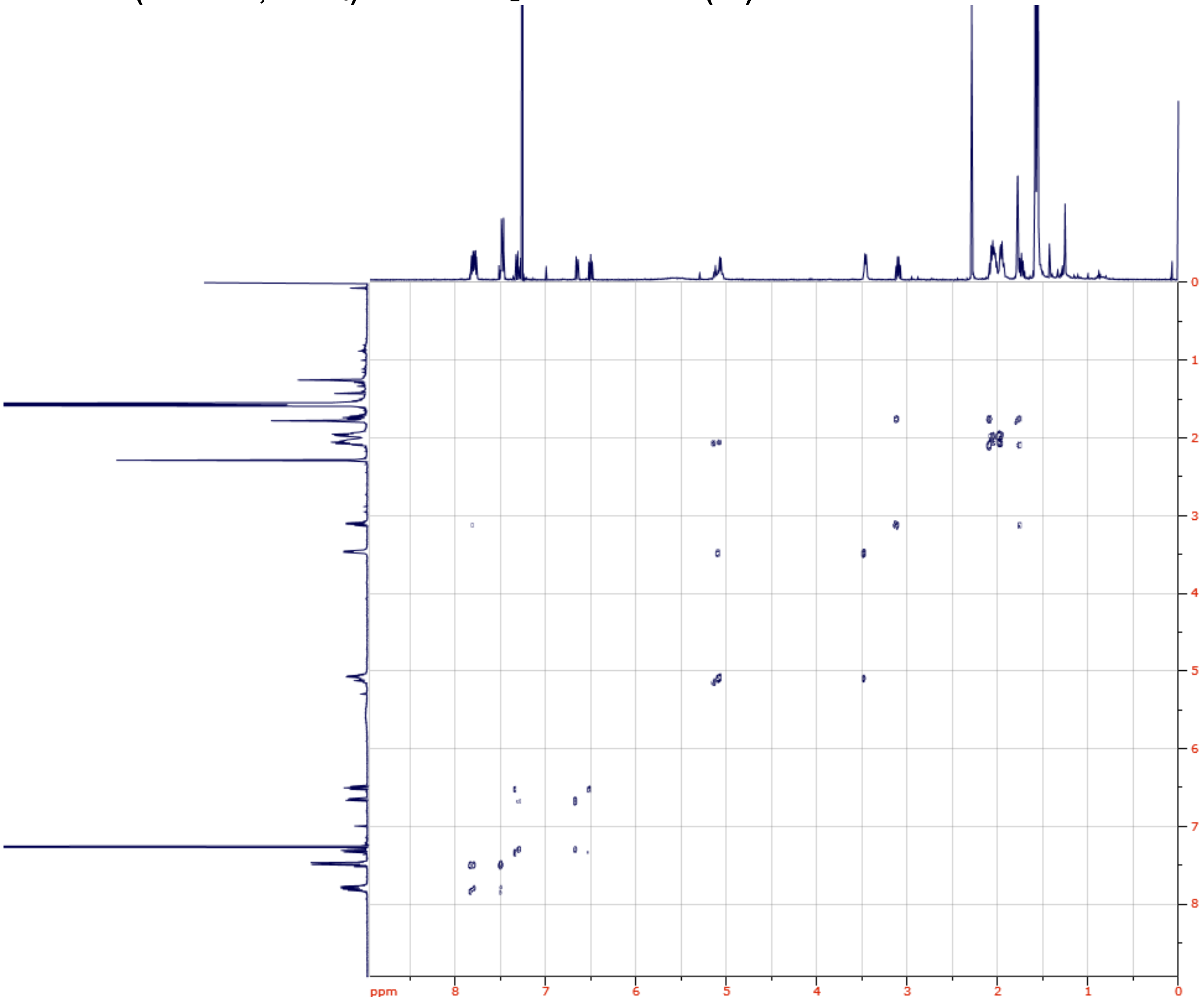

2D HSQC (400 MHZ, $CDCl_3$) for Boc_2-MK4-ω-2-AB (14)

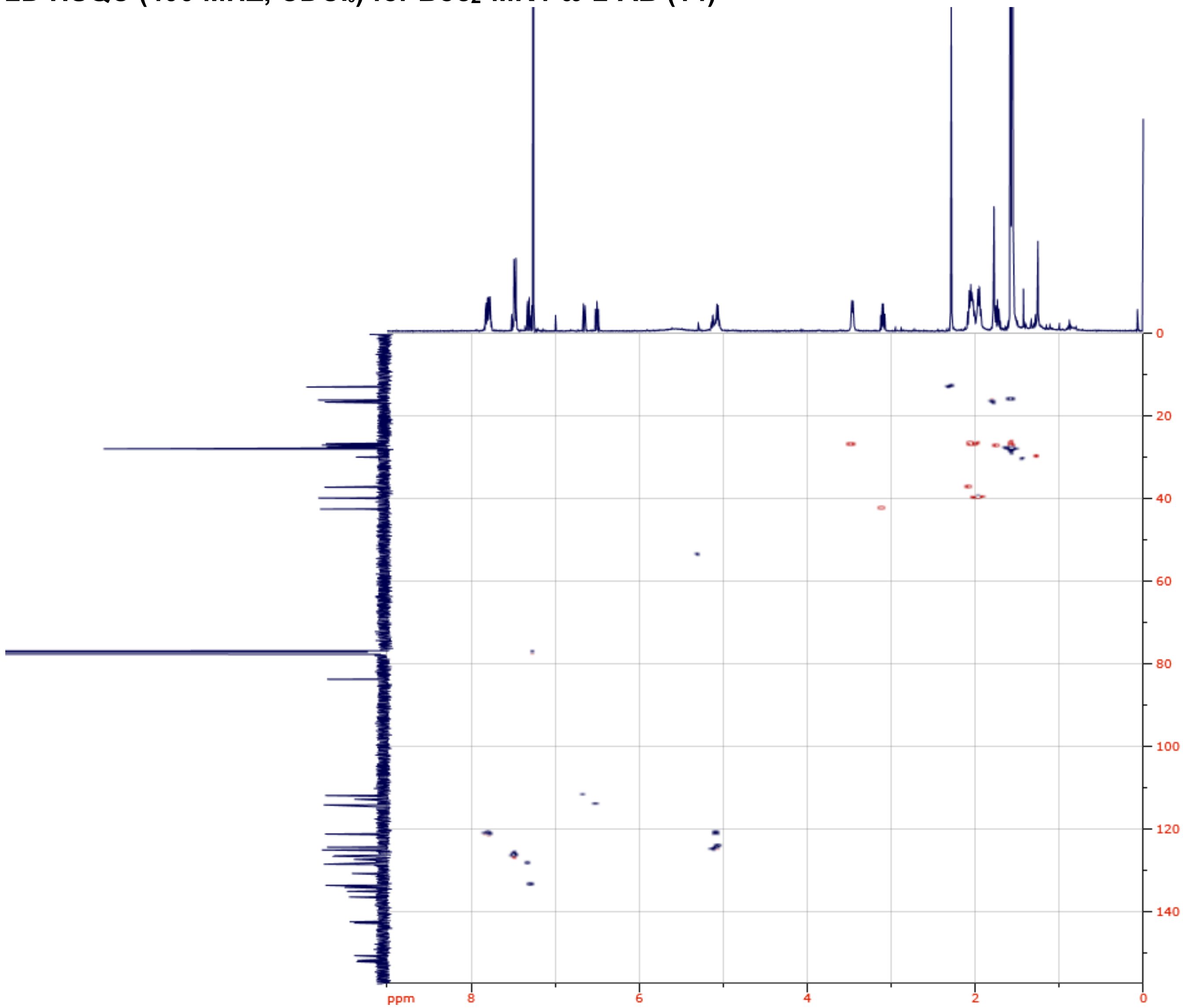

2D HMBC (400 MHZ, $CDCl_3$) for Boc_2-MK4-ω-2-AB (14)

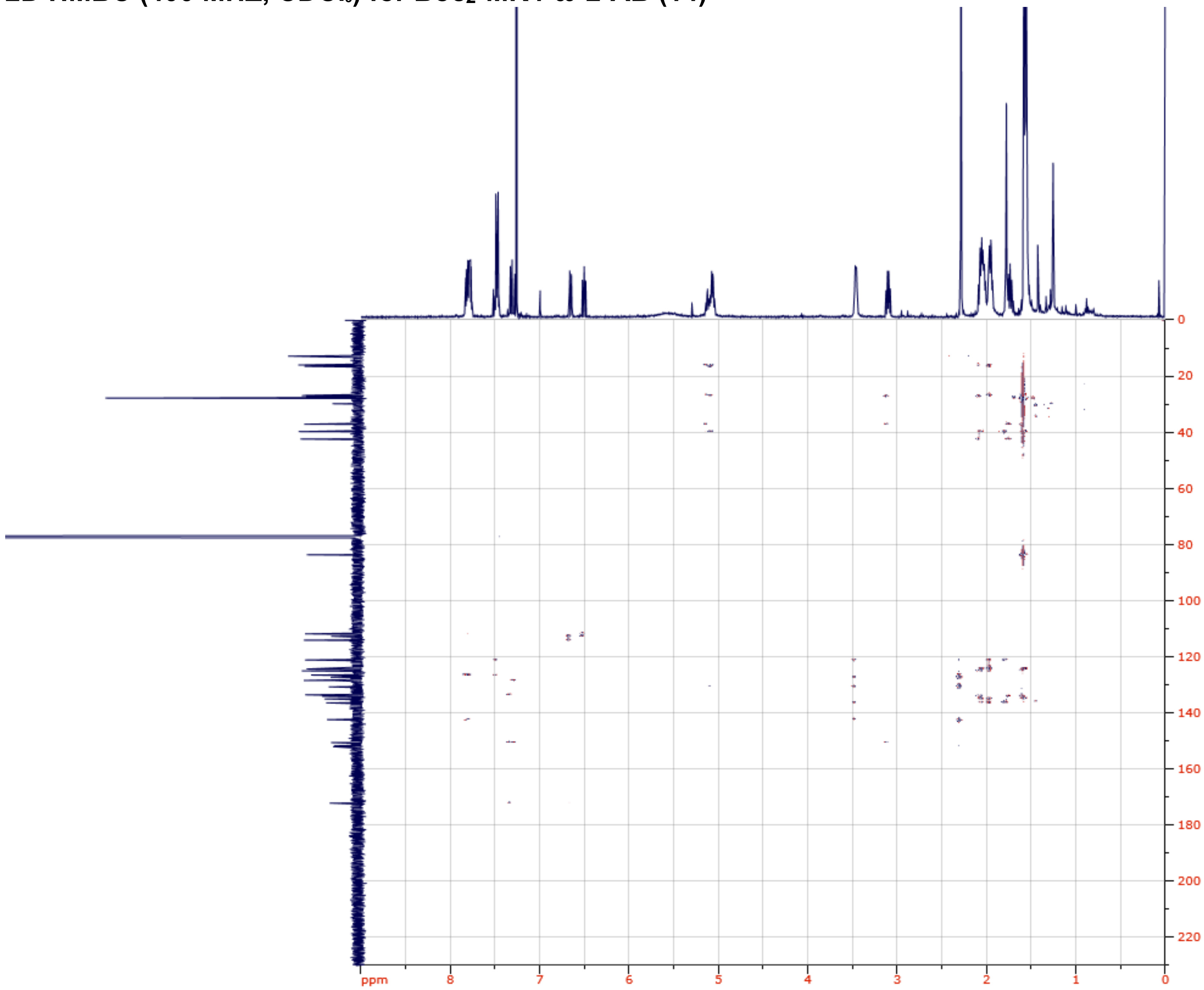

^{1}H NMR (400 MHZ, $CDCl_3$) for Boc_2-MK9-ω-2-AB (15)

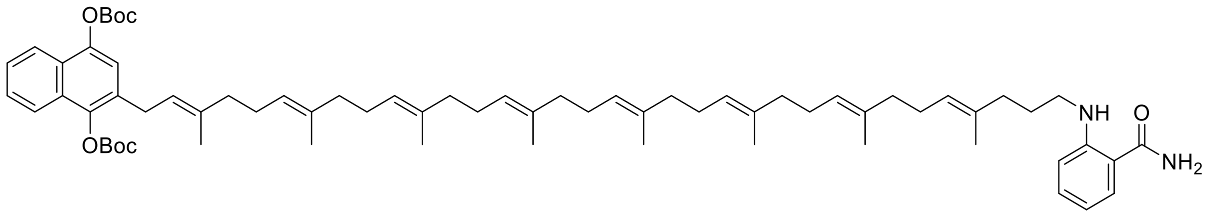

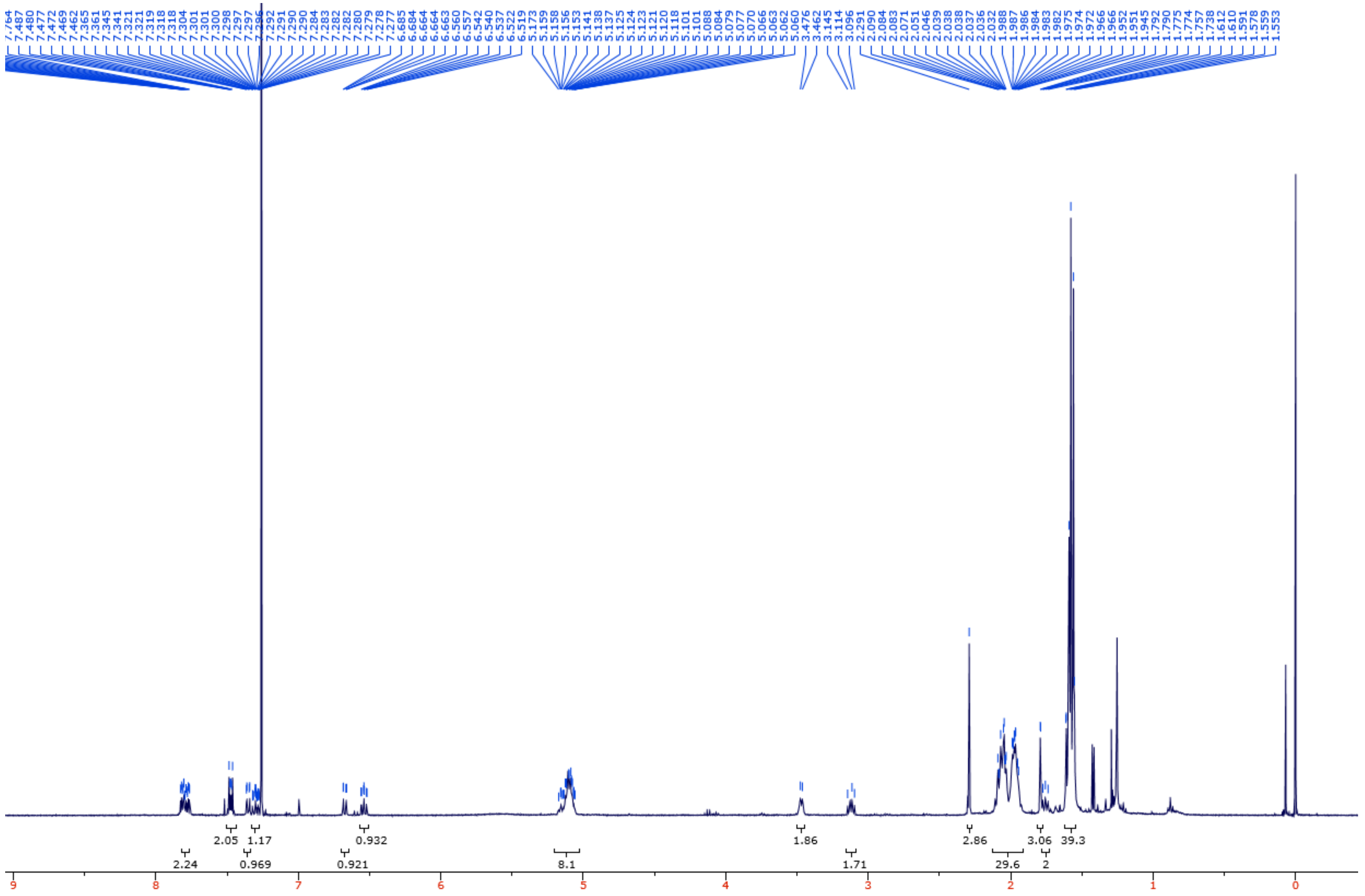

^{13}C NMR (101 MHZ, $CDCl_3$) for Boc$_2$-MK9-ω-2-AB (15)

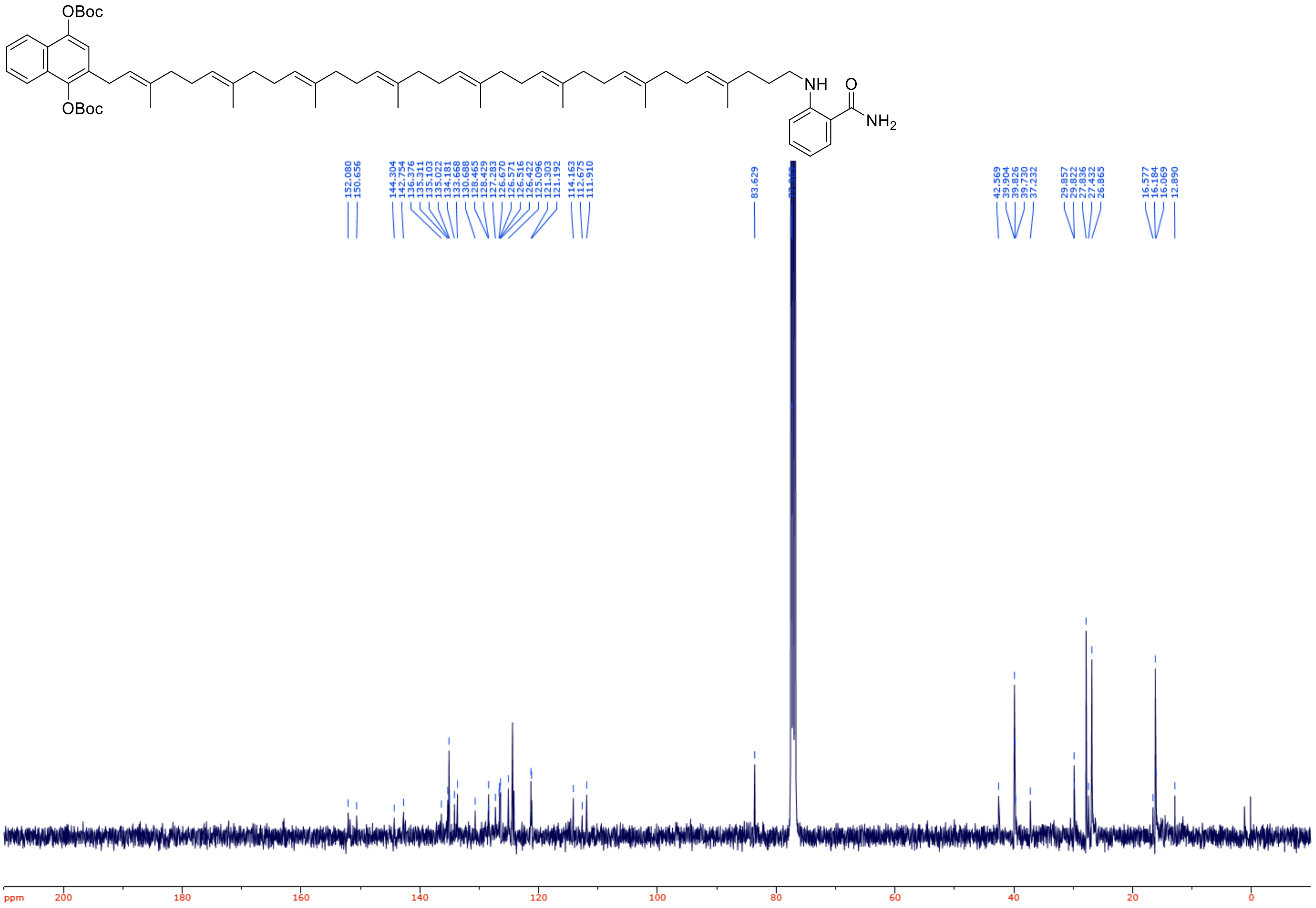

2D COSY (400 MHZ, $CDCl_3$) for Boc_2-MK9-ω-2-AB (15)

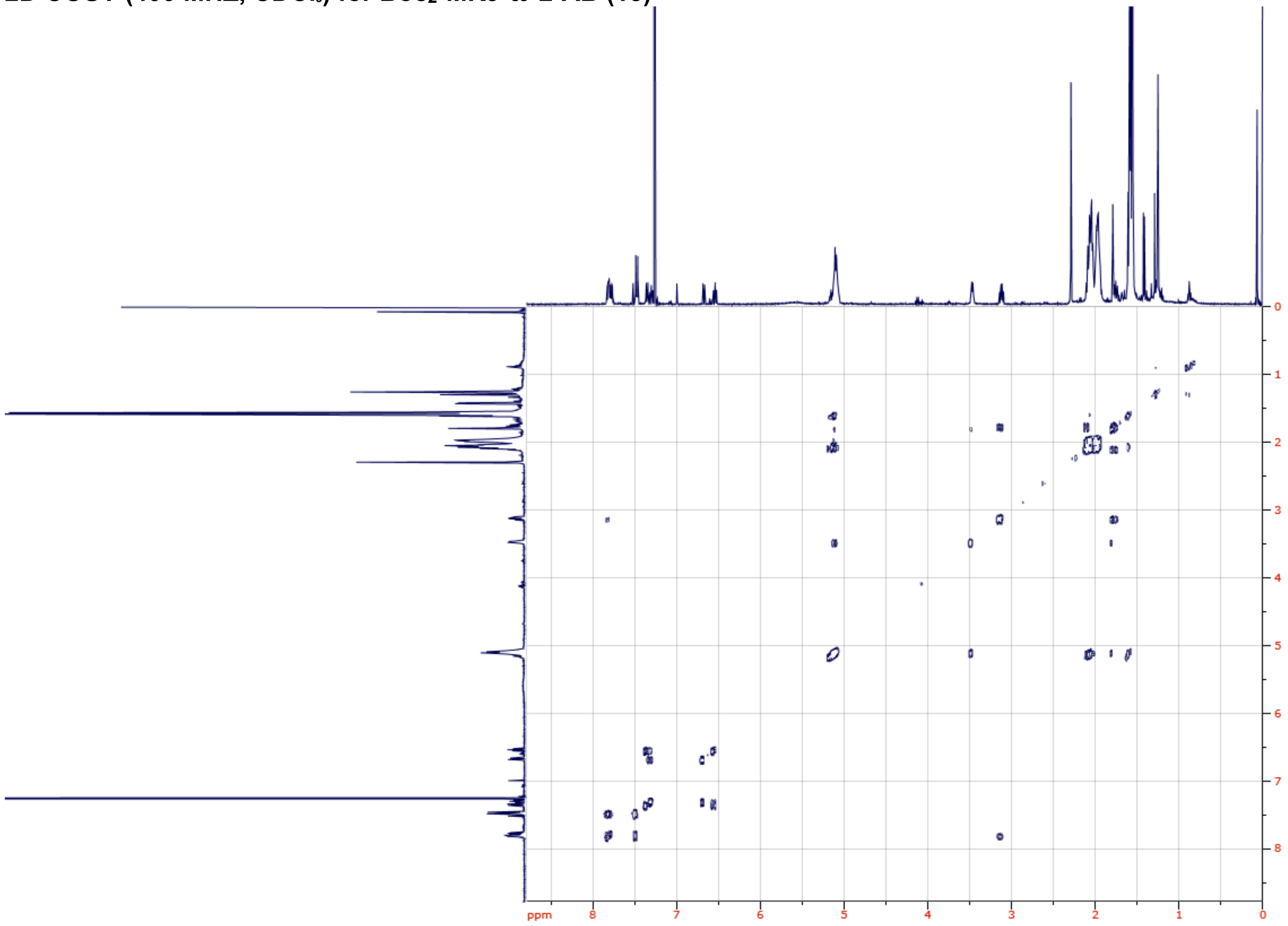

2D HSQC (400 MHZ, $CDCl_3$) for Boc_2-MK9-ω-2-AB (15)

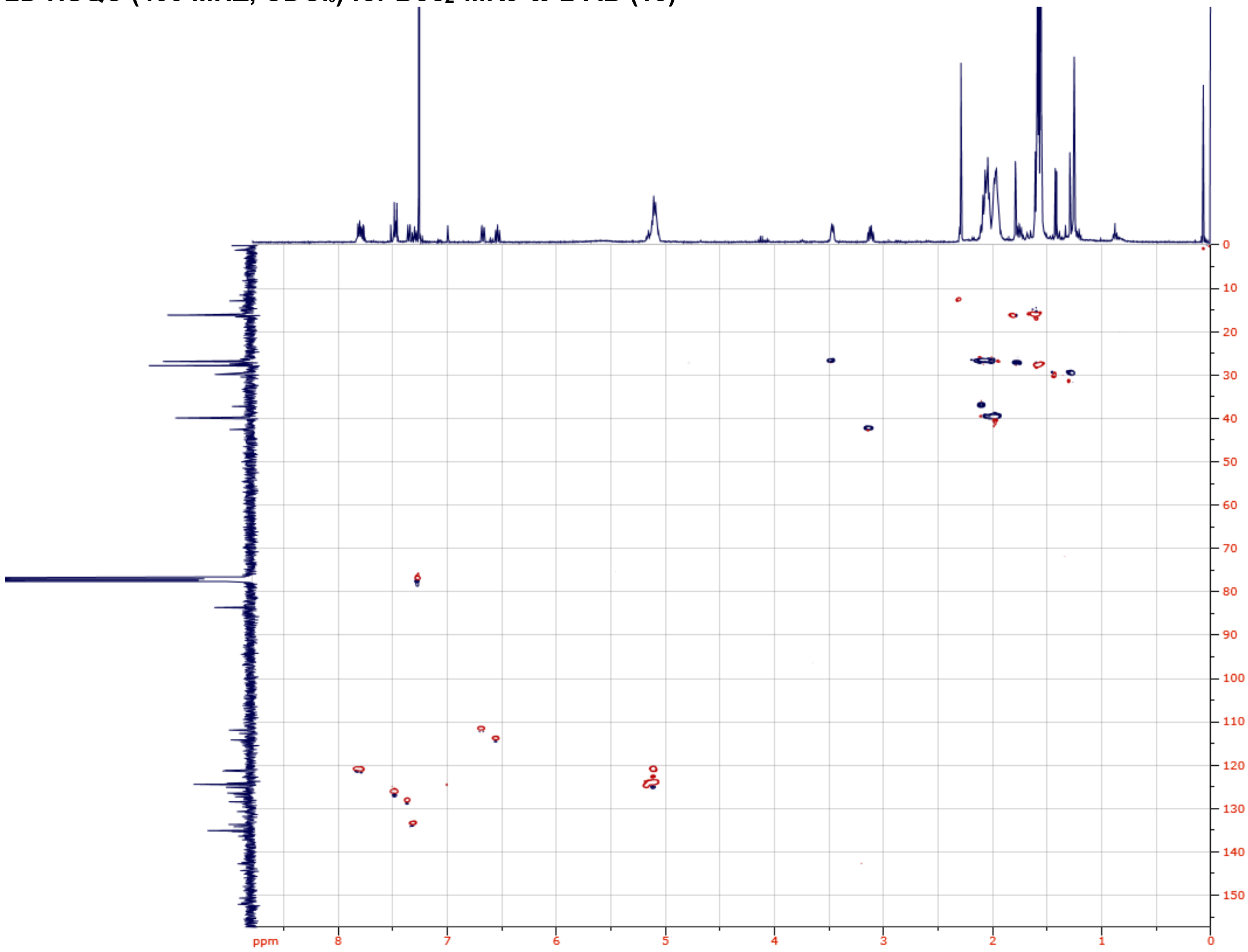

2D HMBC (400 MHZ, $CDCl_3$) for Boc_2-MK9-ω-2-AB (15)

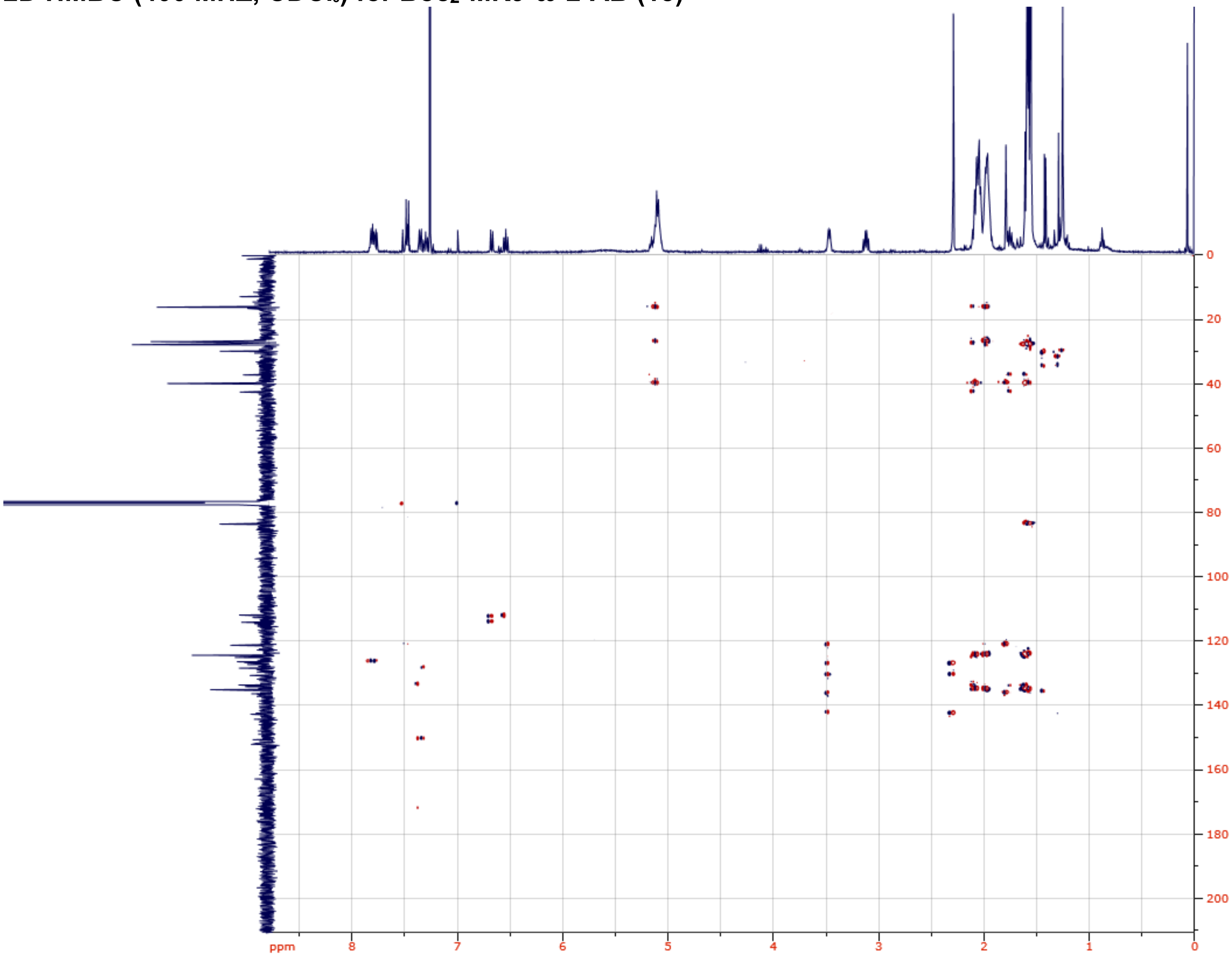

^{1}H NMR (400 MHZ, $CDCl_3$) for CoQ10-ω-2-AB (16)

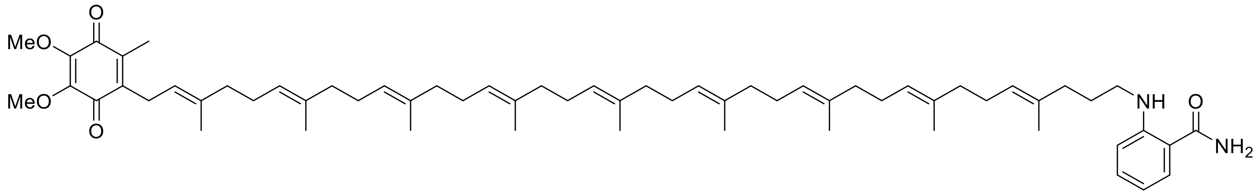

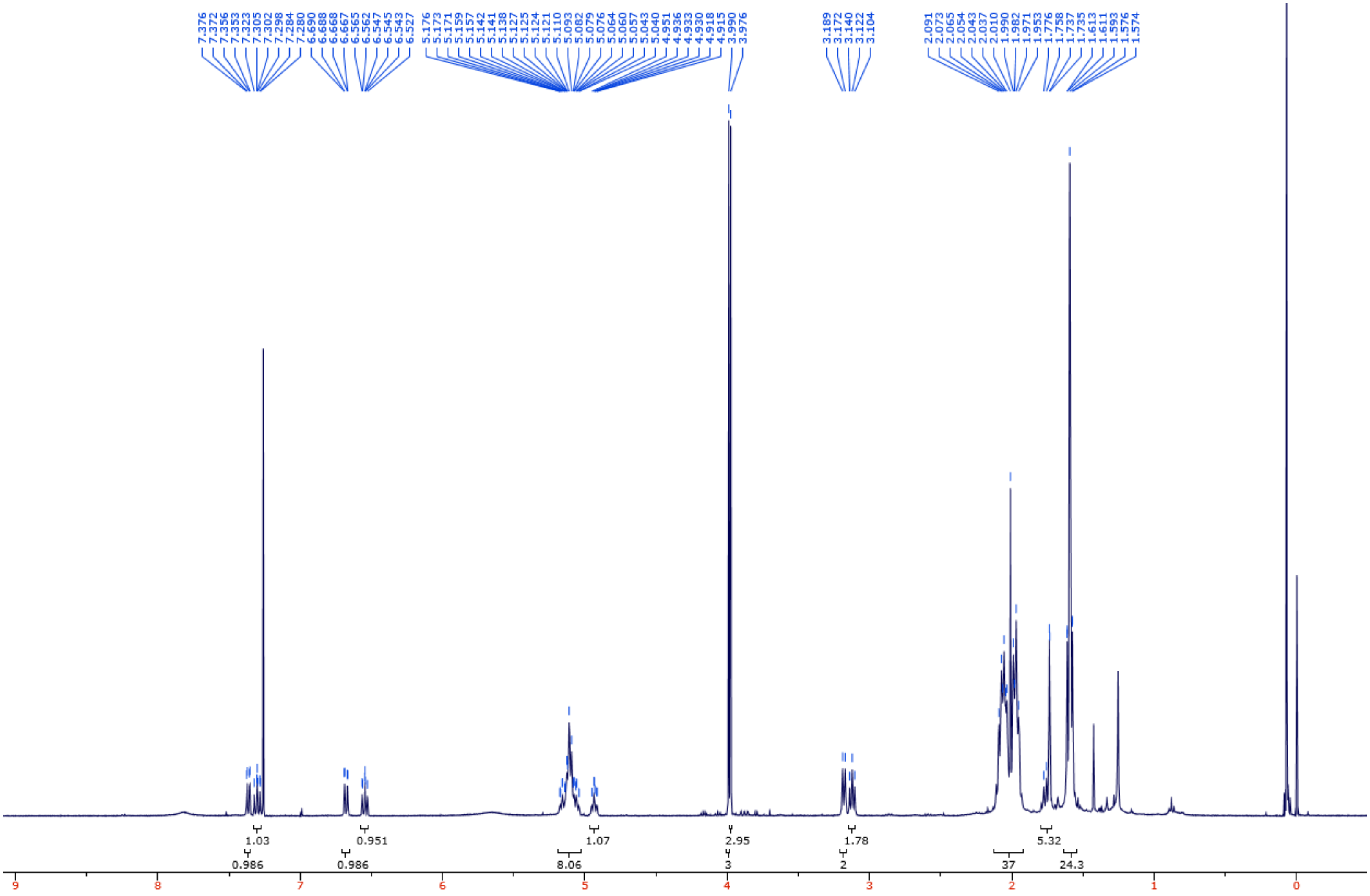

^{13}C NMR (101 MHZ, $CDCl_3$) for CoQ10-ω-2-AB (16)

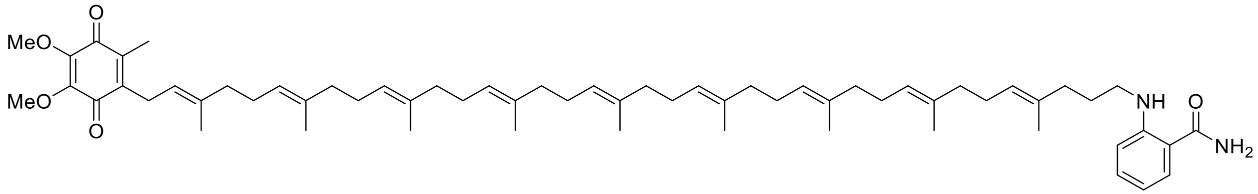

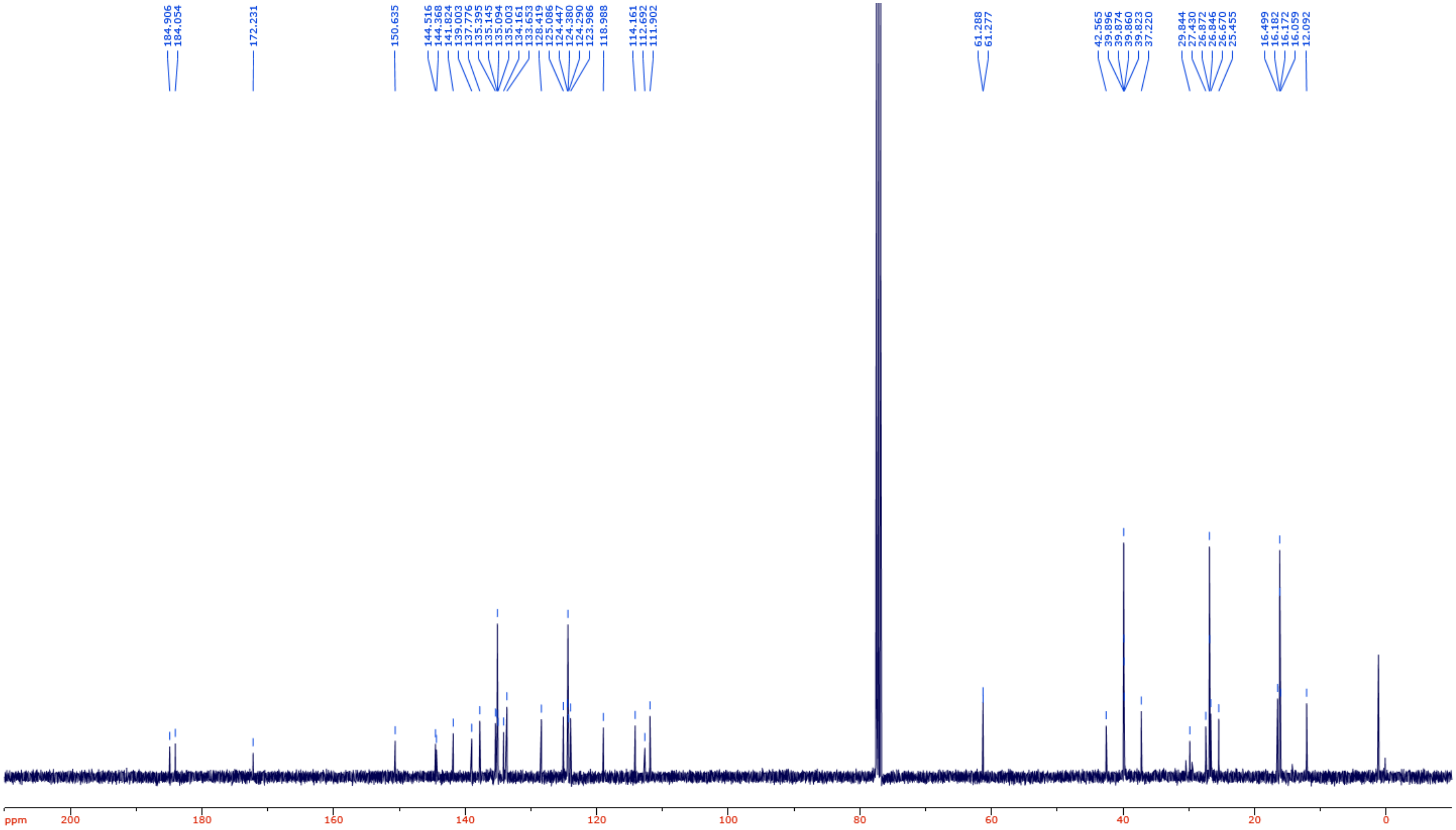

2D COSY (400 MHZ, $CDCl_3$) for CoQ10-ω-2-AB (16)

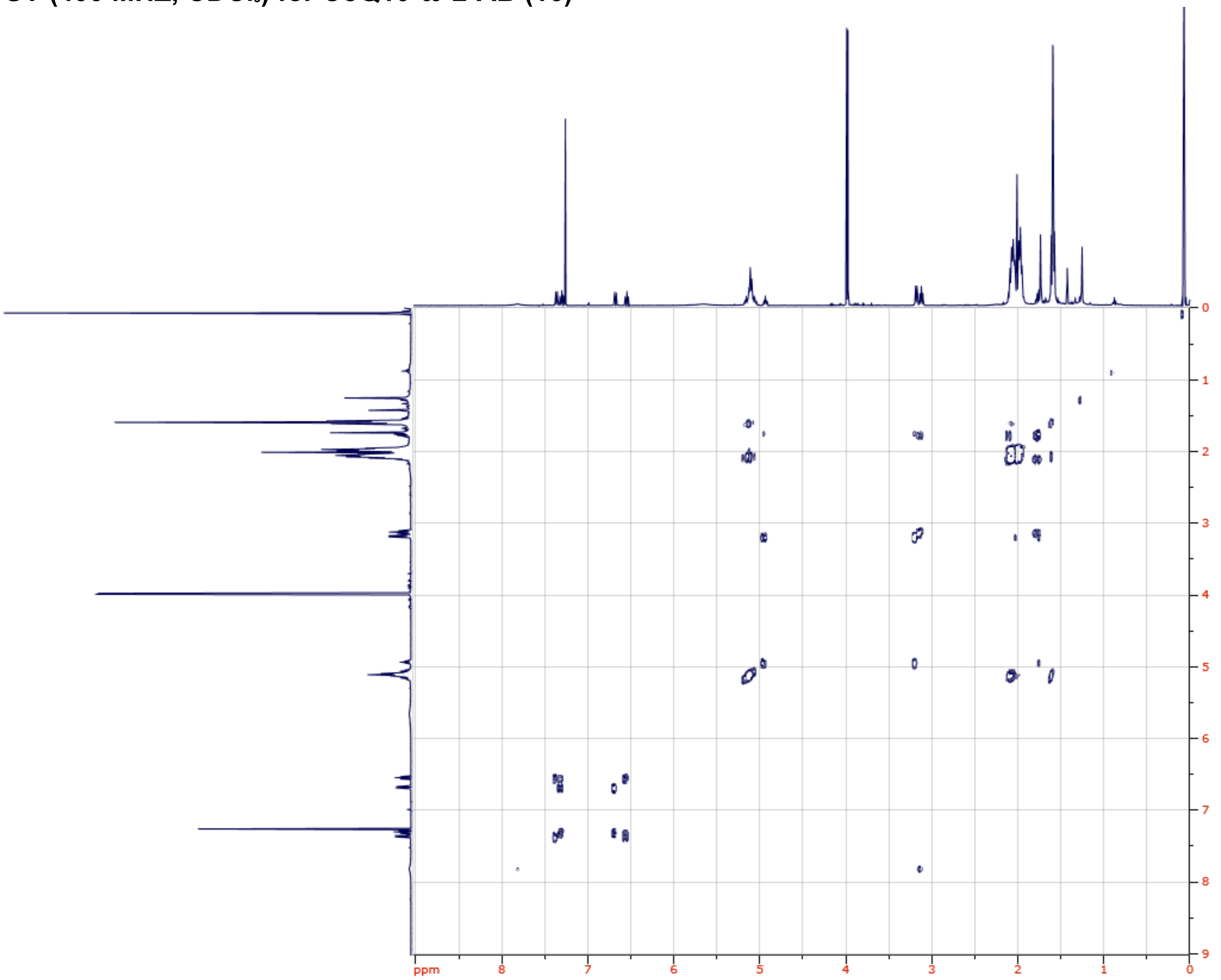

2D HSQC (400 MHZ, $CDCl_3$) for CoQ10-ω-2-AB (16)

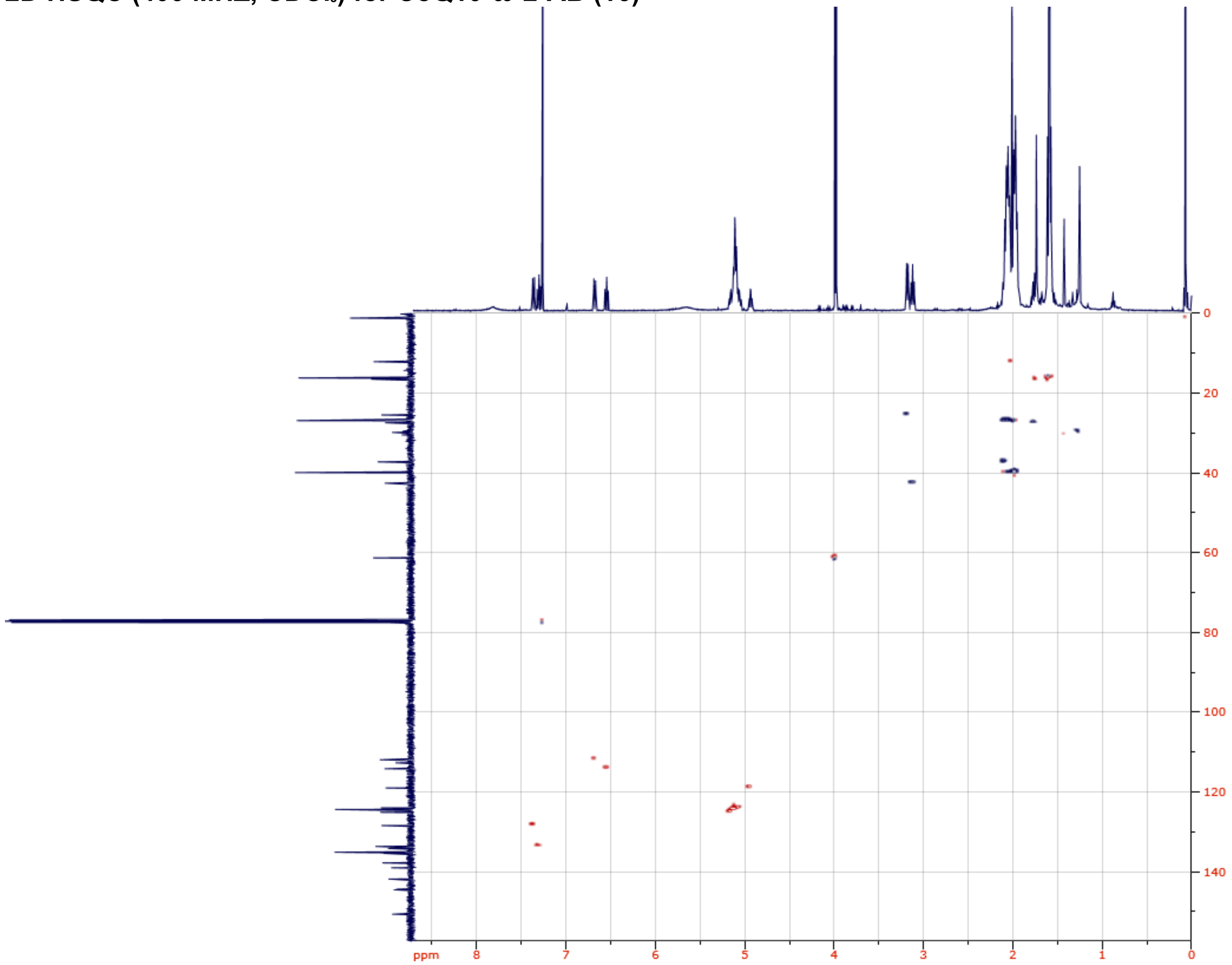

2D HMBC (400 MHZ, $CDCl_3$) for CoQ10-ω-2-AB (16)

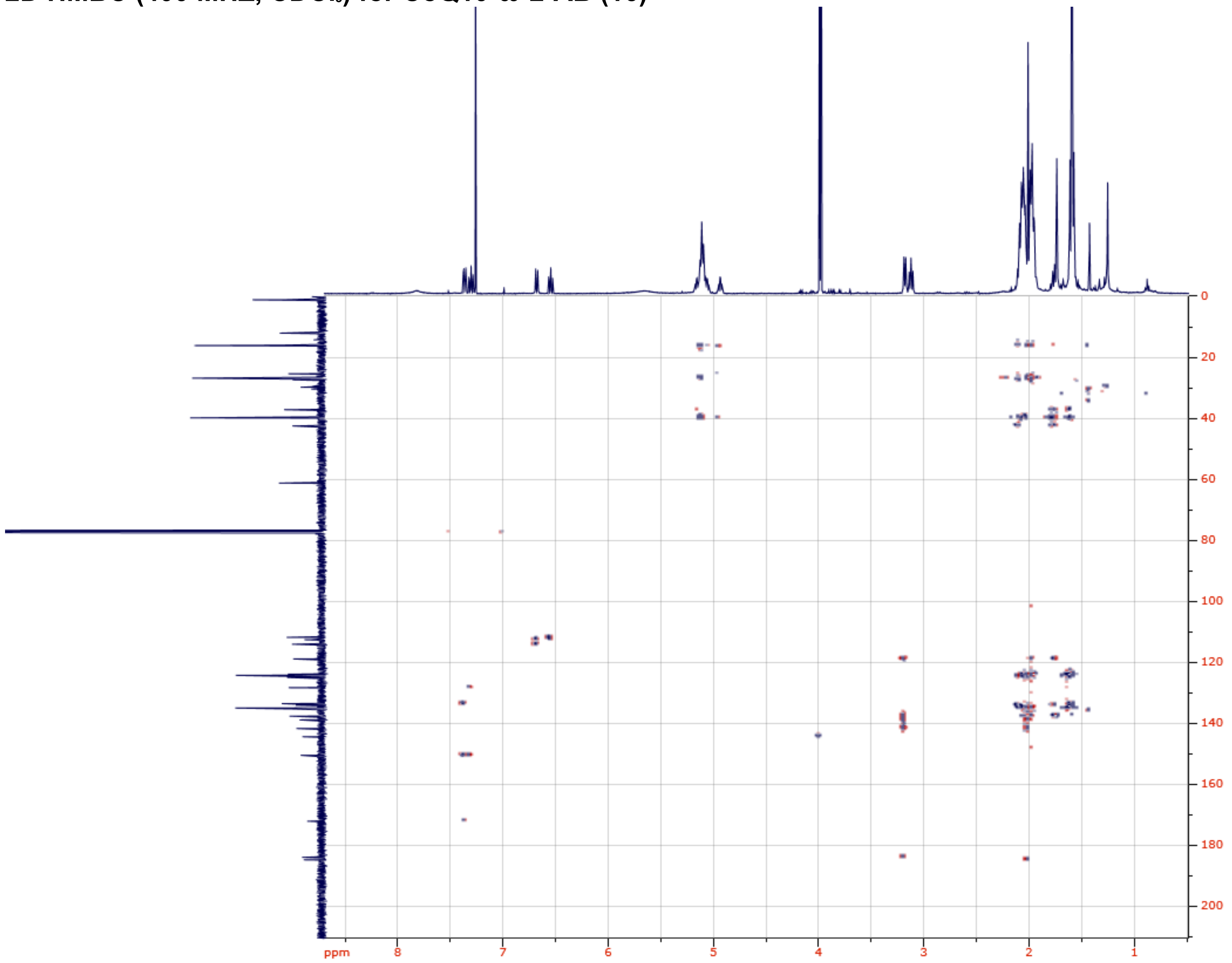

^{1}H NMR (400 MHZ, $CDCl_3$) for MK4-ω-2-AB (17)

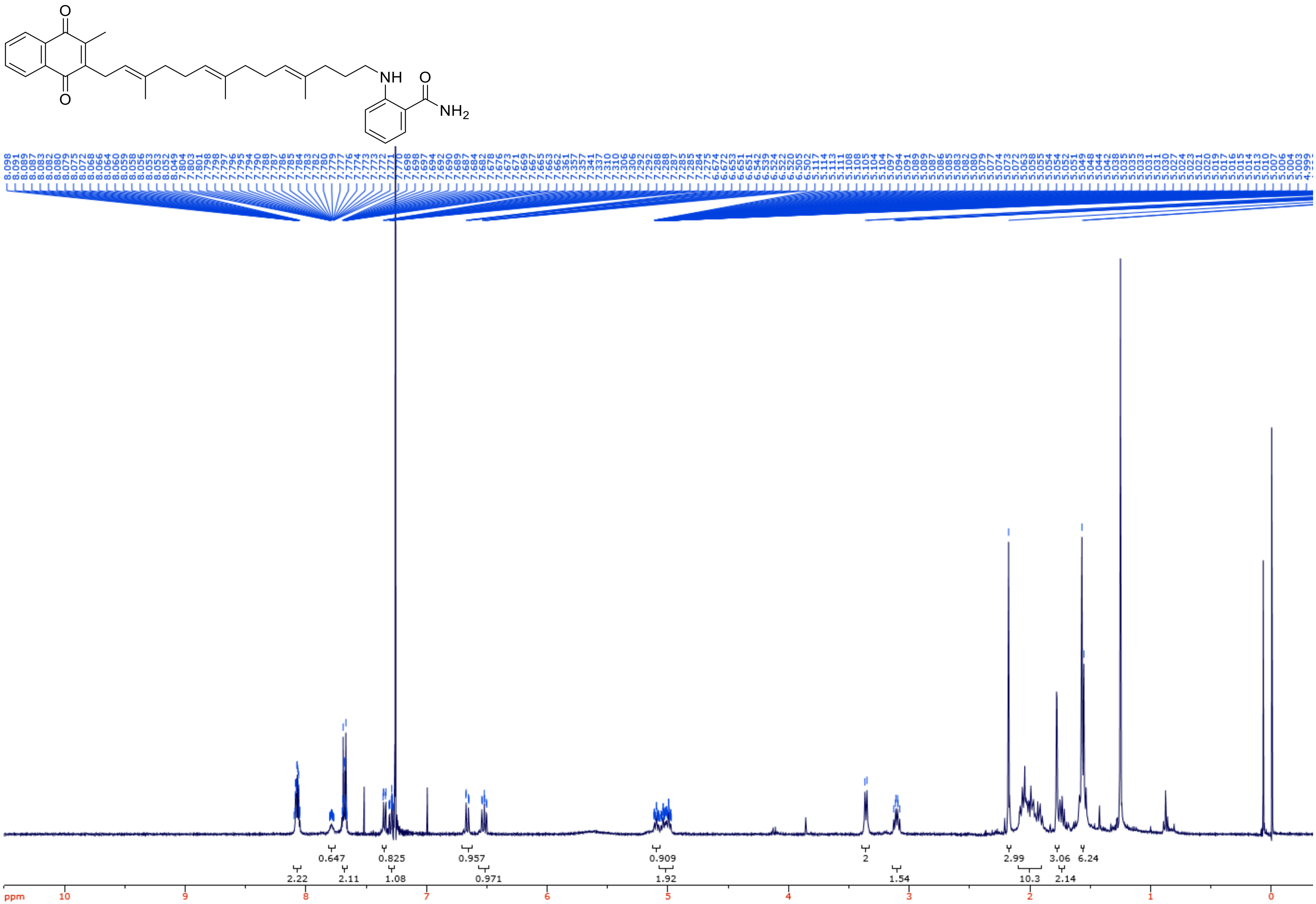

^{13}C NMR (151 MHZ, $CDCl_3$) for MK4-ω-2-AB (17)

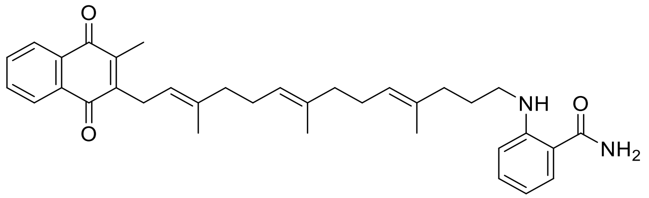

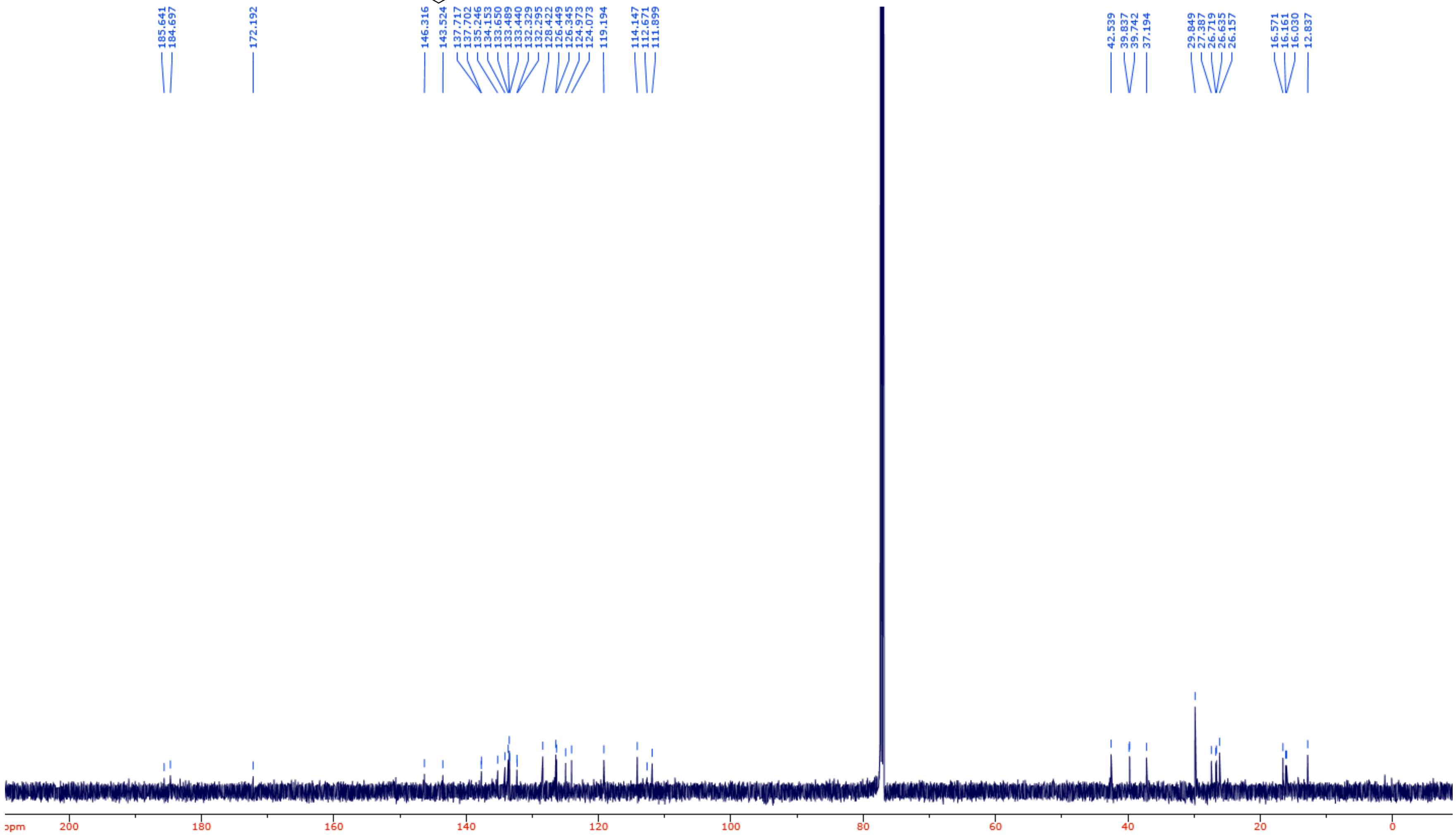

2D COSY (600 MHZ, $CDCl_3$) for MK4-ω-2-AB (17)

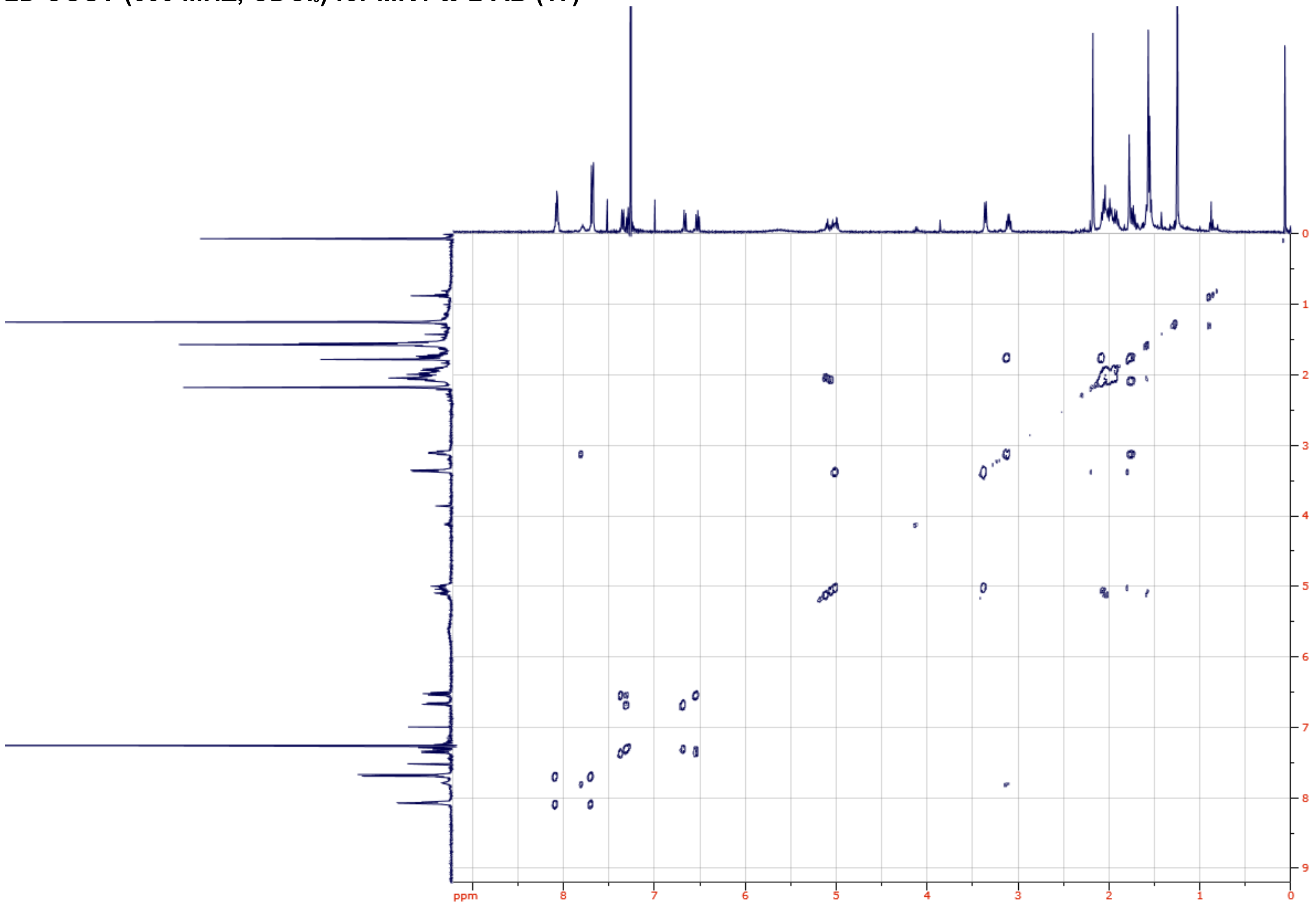

2D HSQC (600 MHZ, $CDCl_3$) for MK4-ω-2-AB (17)

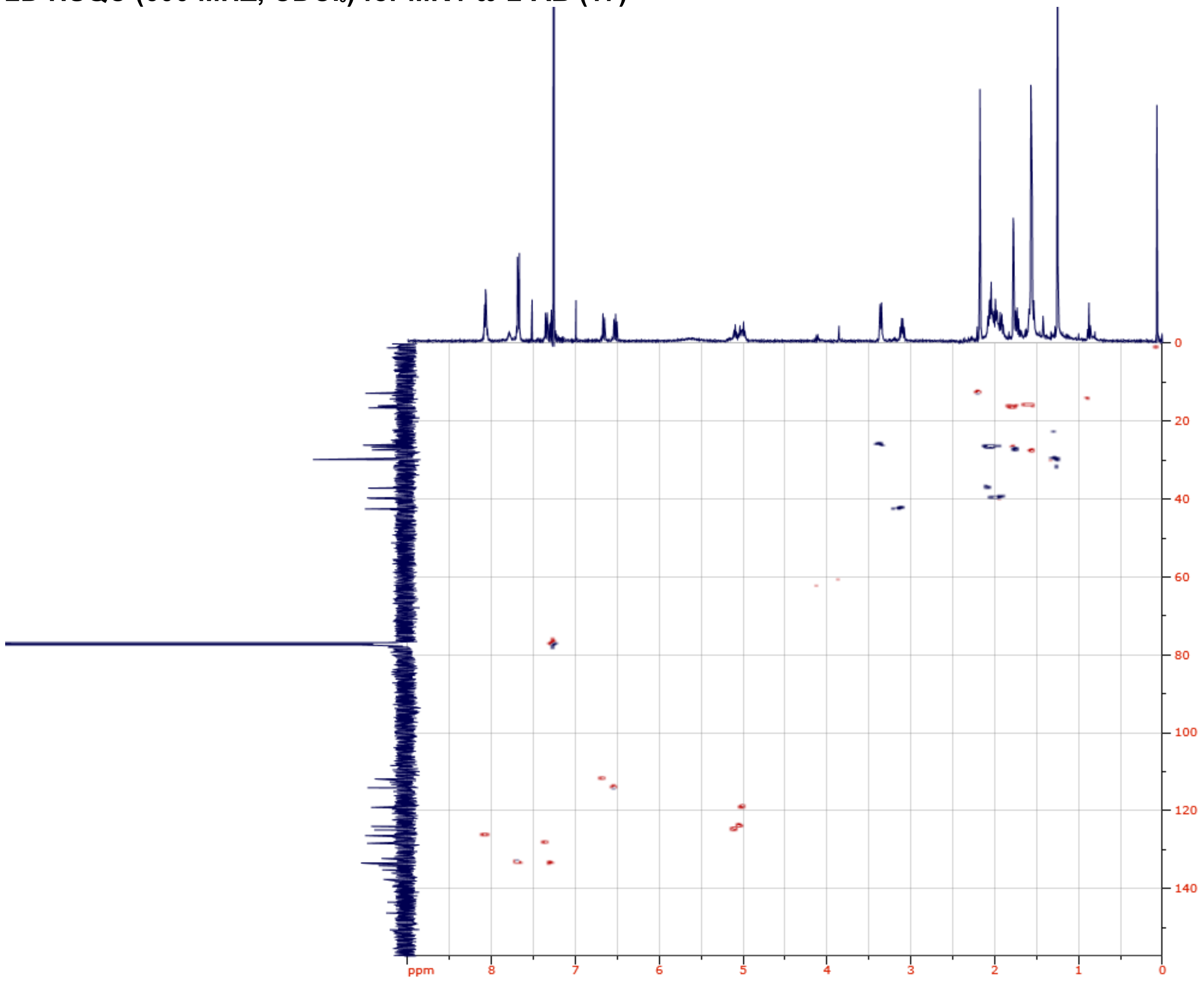

2D HMBC (600 MHZ, $CDCl_3$) for MK4-ω-2-AB (17)

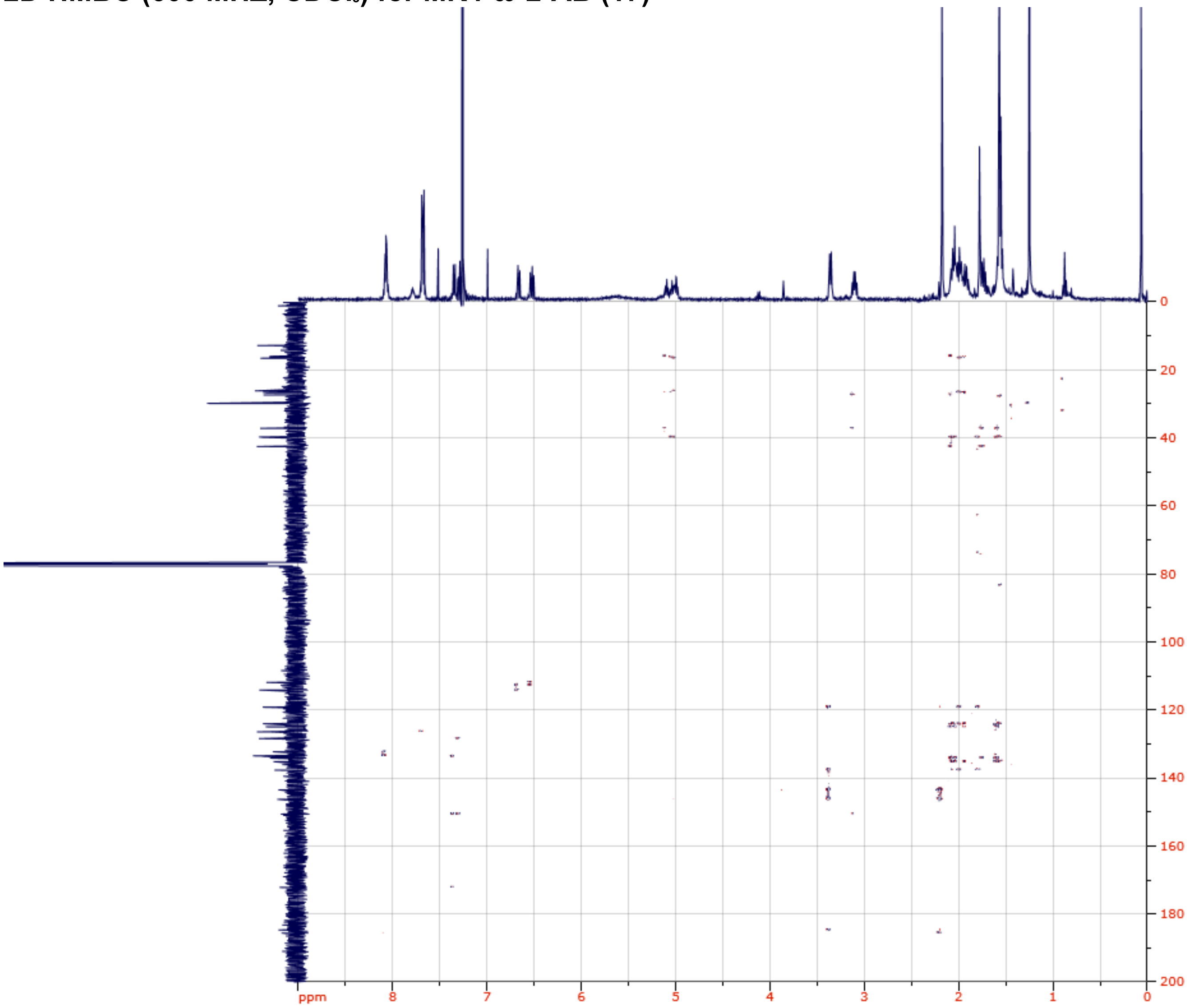

^{1}H NMR (400 MHZ, $CDCl_3$) for MK9-ω-2-AB (18)

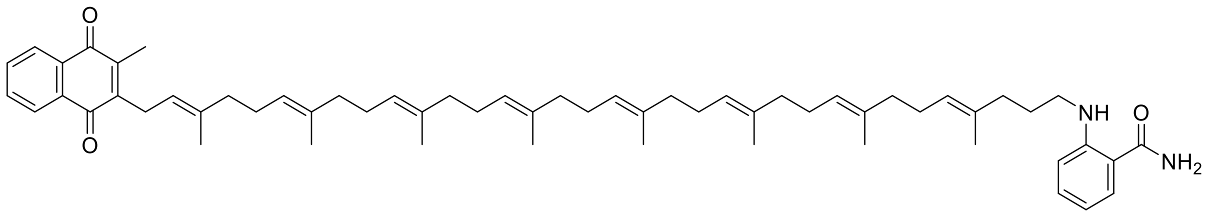

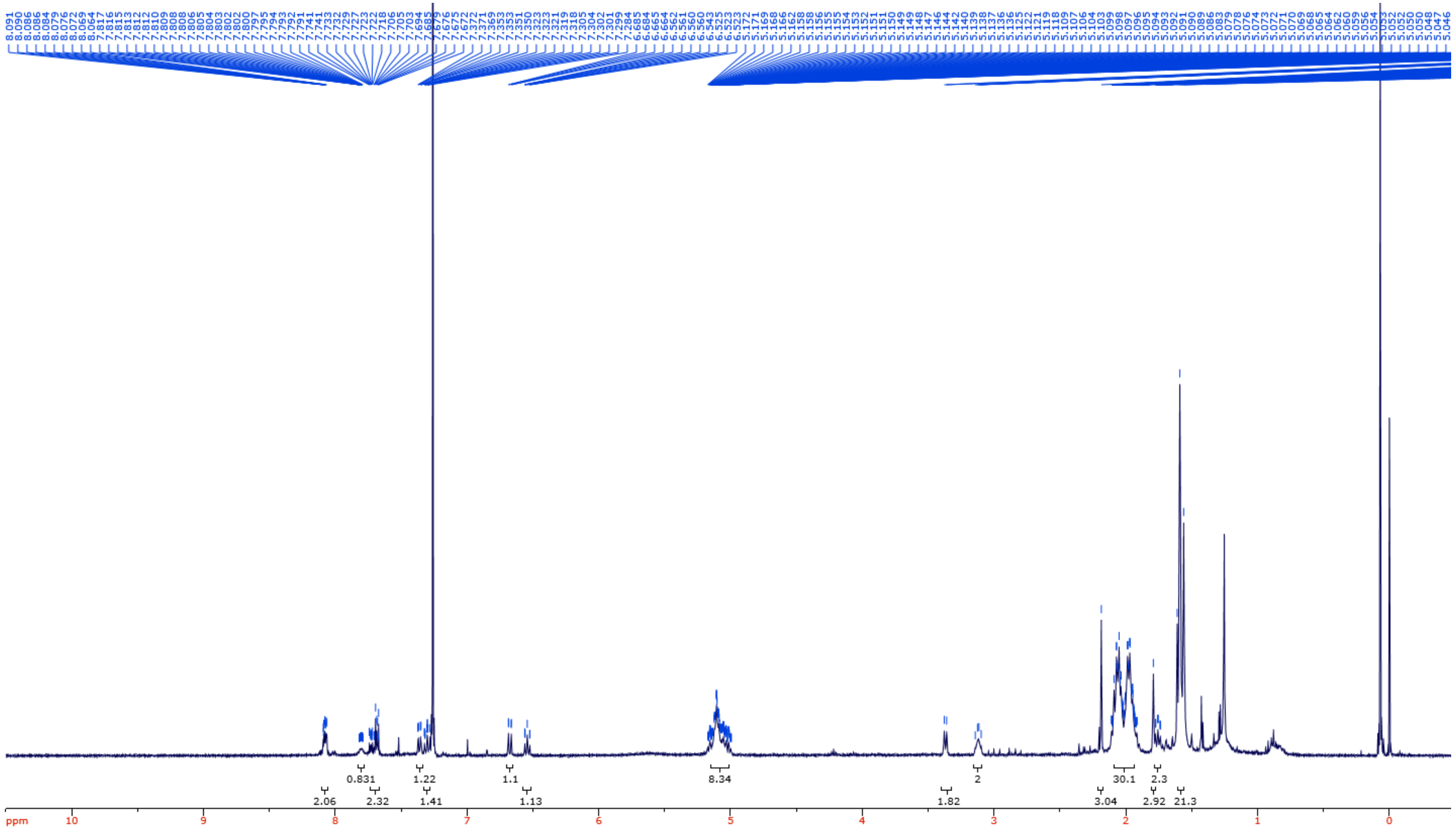

^{13}C NMR (101 MHZ, $CDCl_3$) for for MK9-ω-2-AB (18)

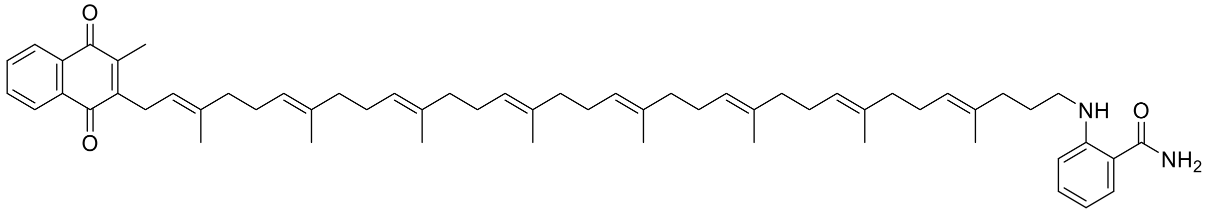

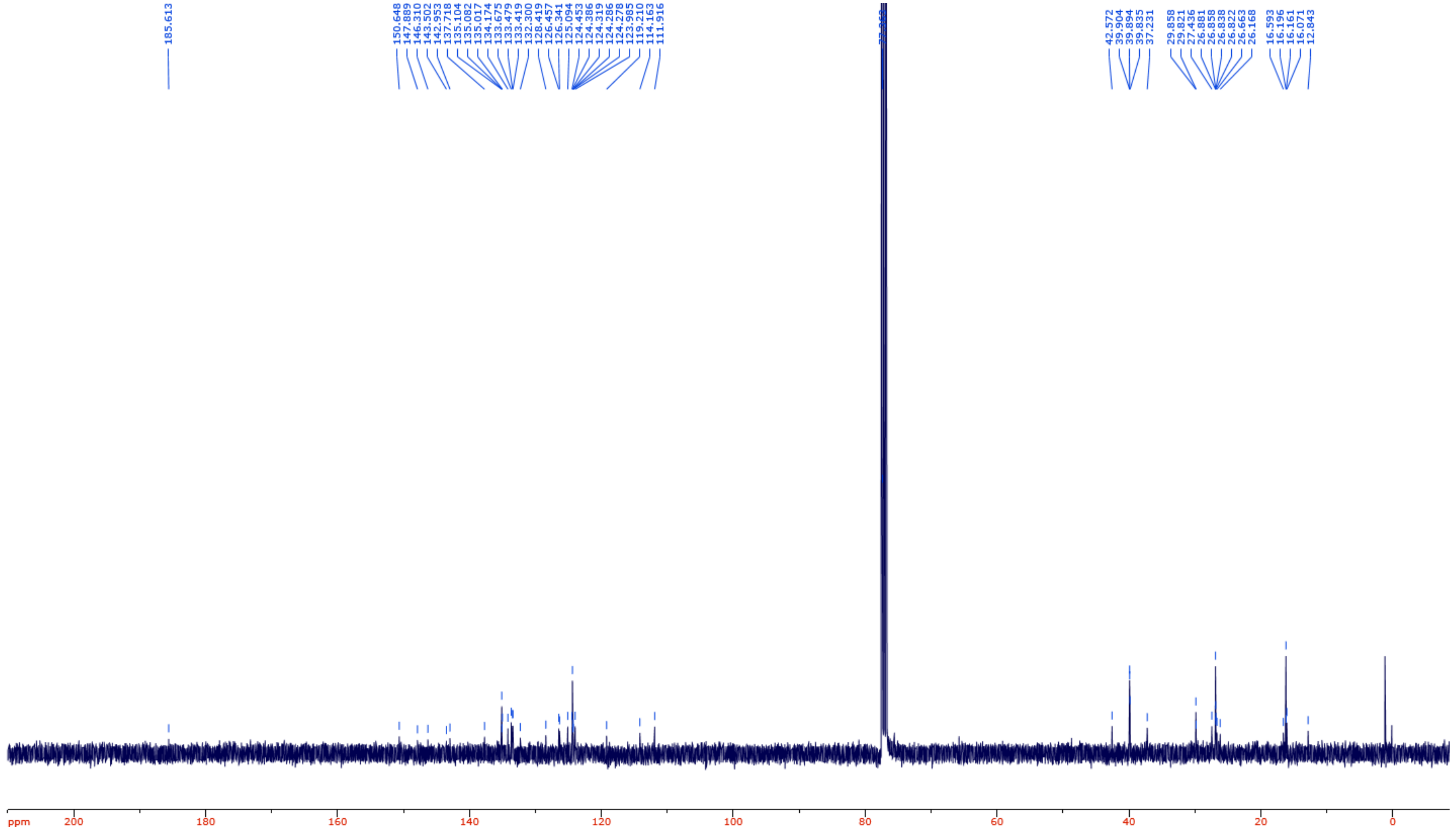

2D COSY (400 MHZ, $CDCl_3$) for MK9-ω-2-AB (18)

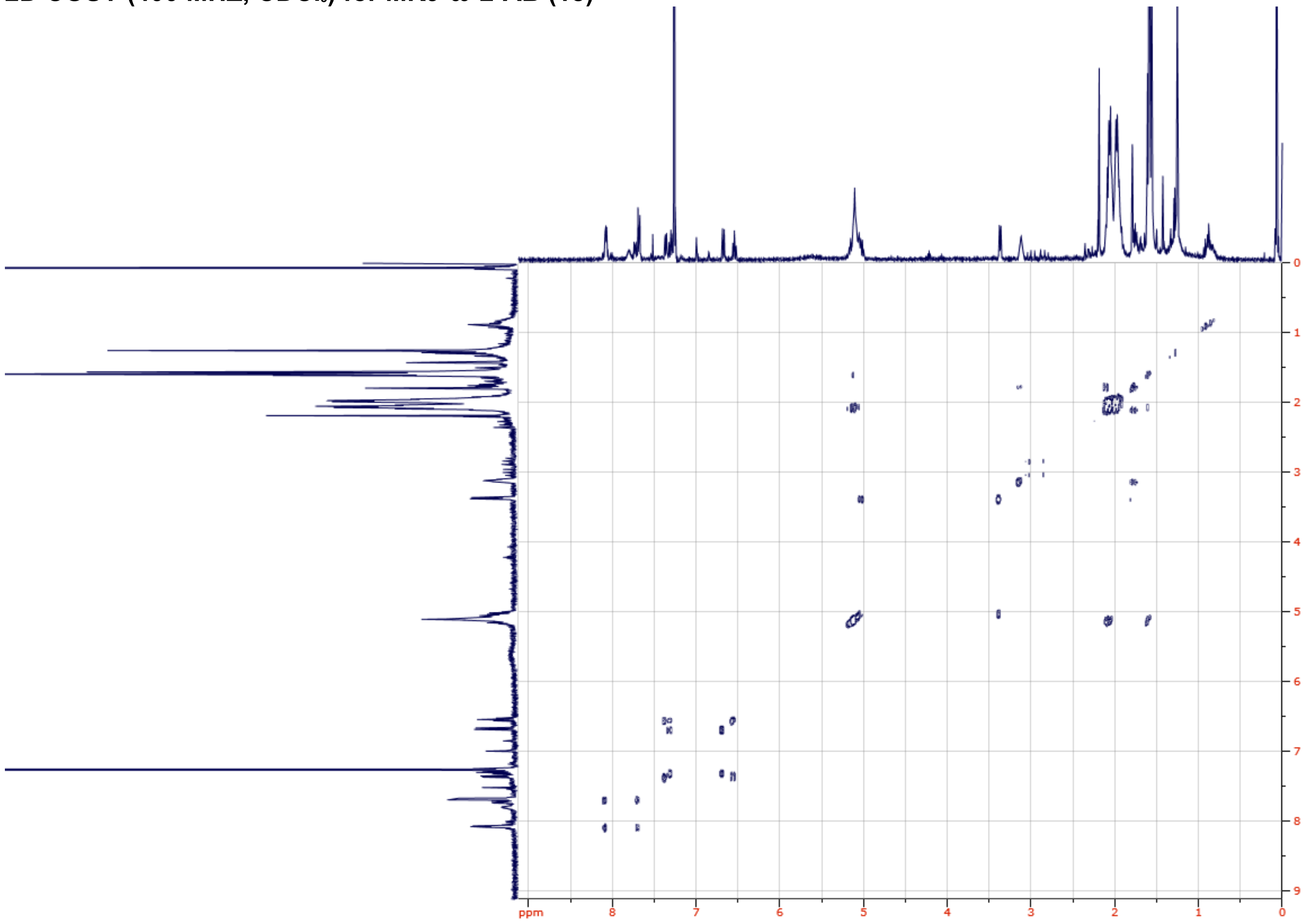

2D HSQC (400 MHZ, $CDCl_3$) for MK9-ω-2-AB (18)

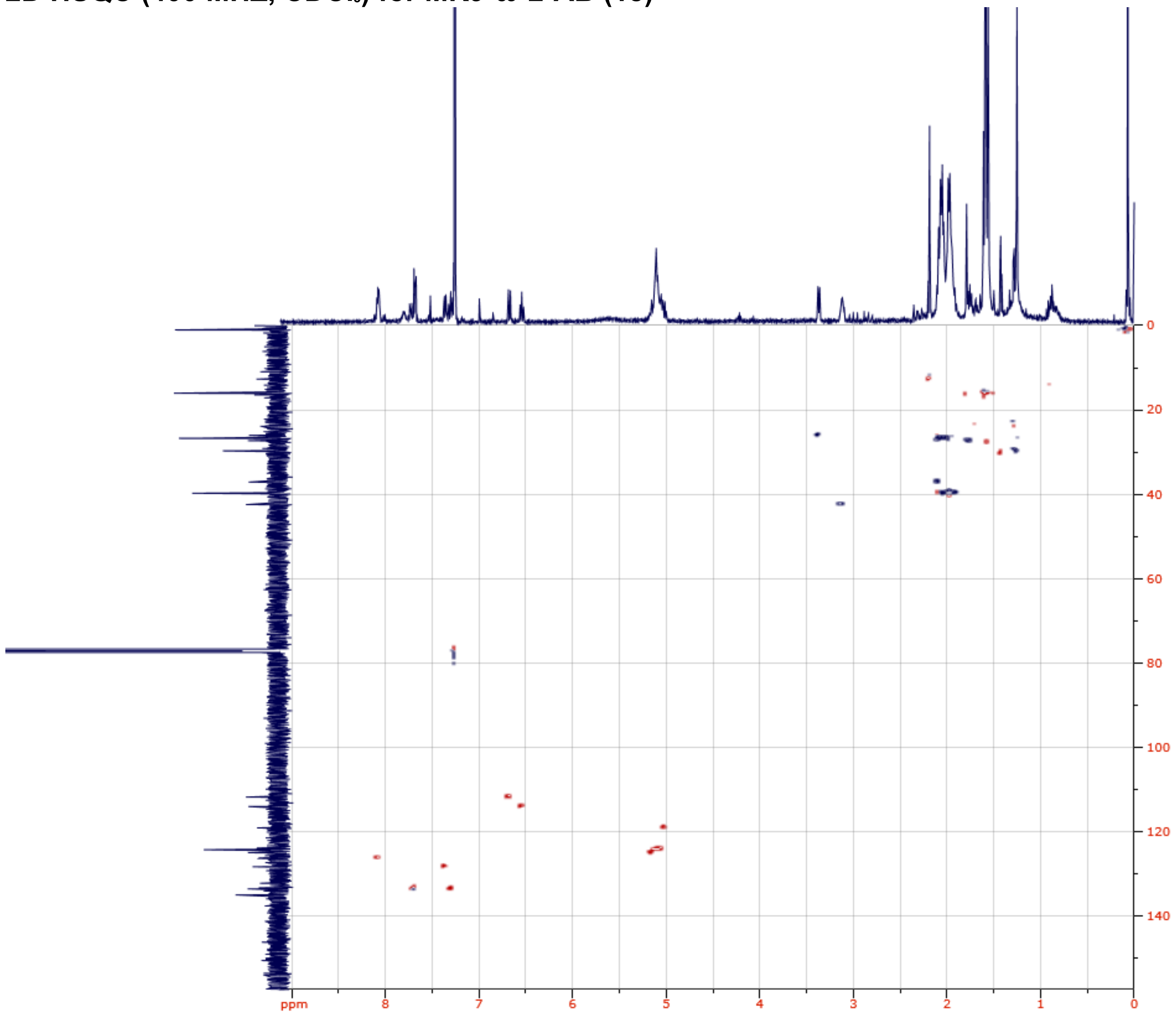

2D HSQC (400 MHZ, $CDCl_3$) for MK9-ω-2-AB (18)

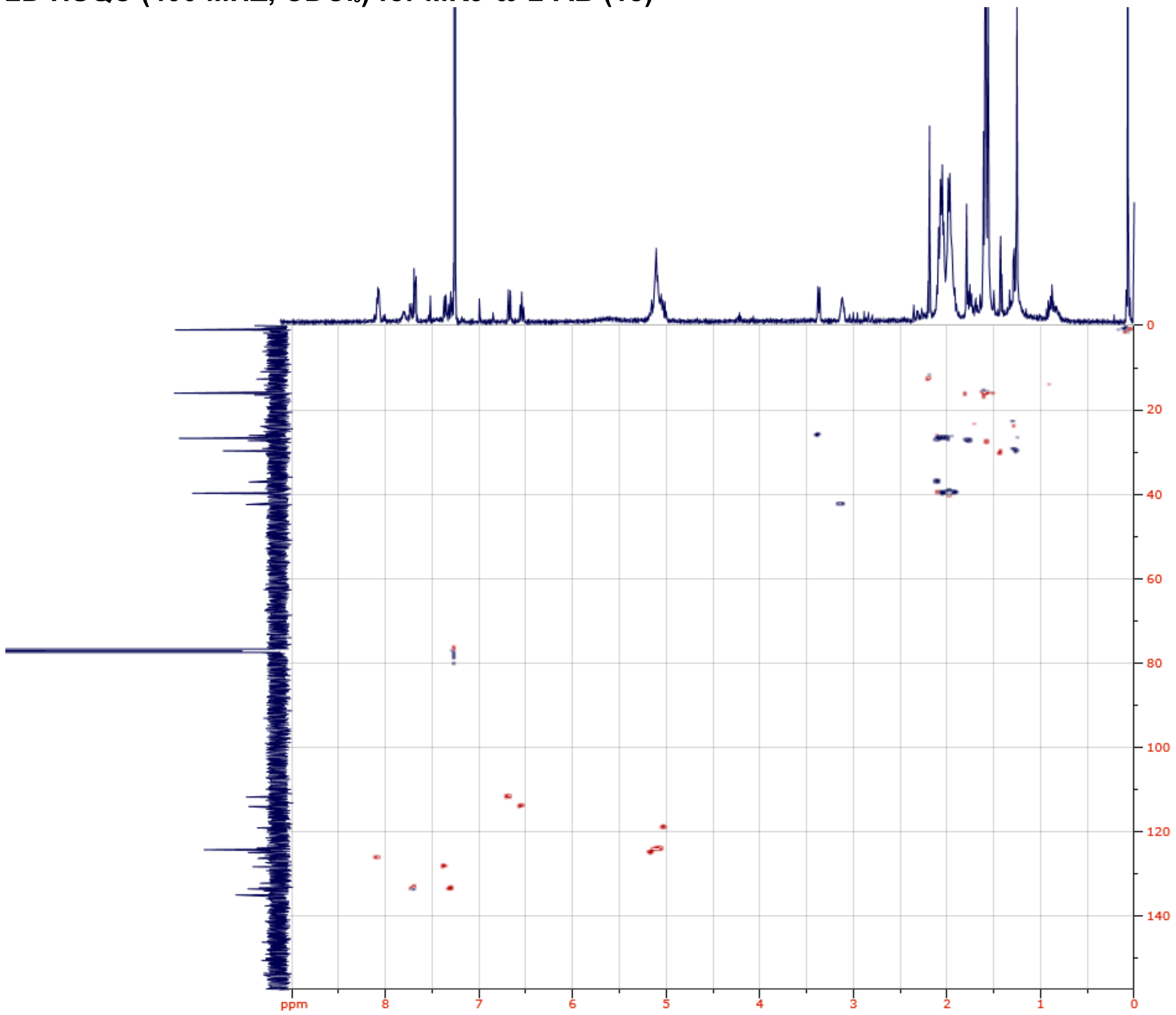

2D HMBC (400 MHZ, $CDCl_3$) for MK9-ω-2-AB (18)

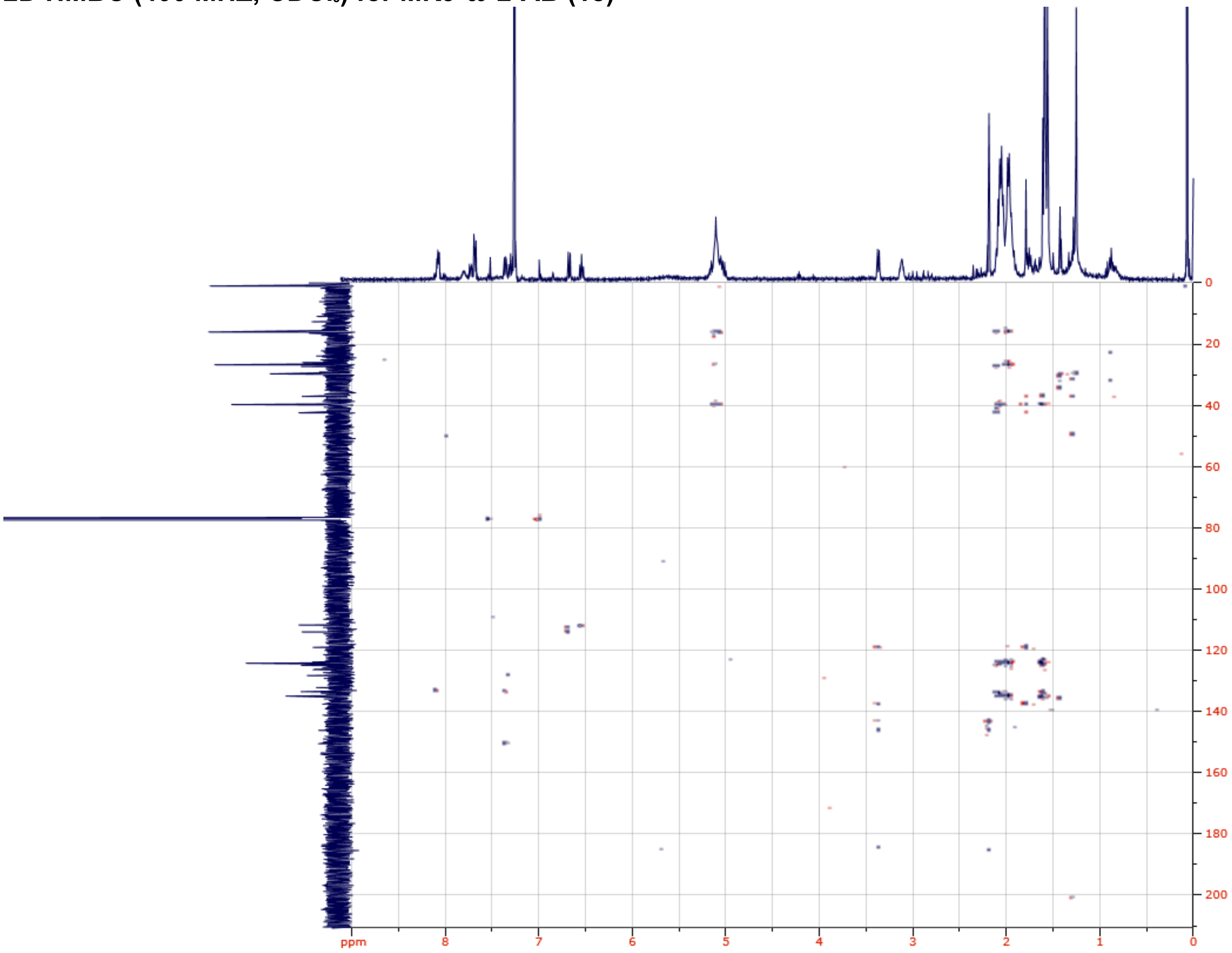

^{1}H NMR (400 MHZ, $CDCl_3$) for Boc_2-CoQ10-ω-alkyne (19a)

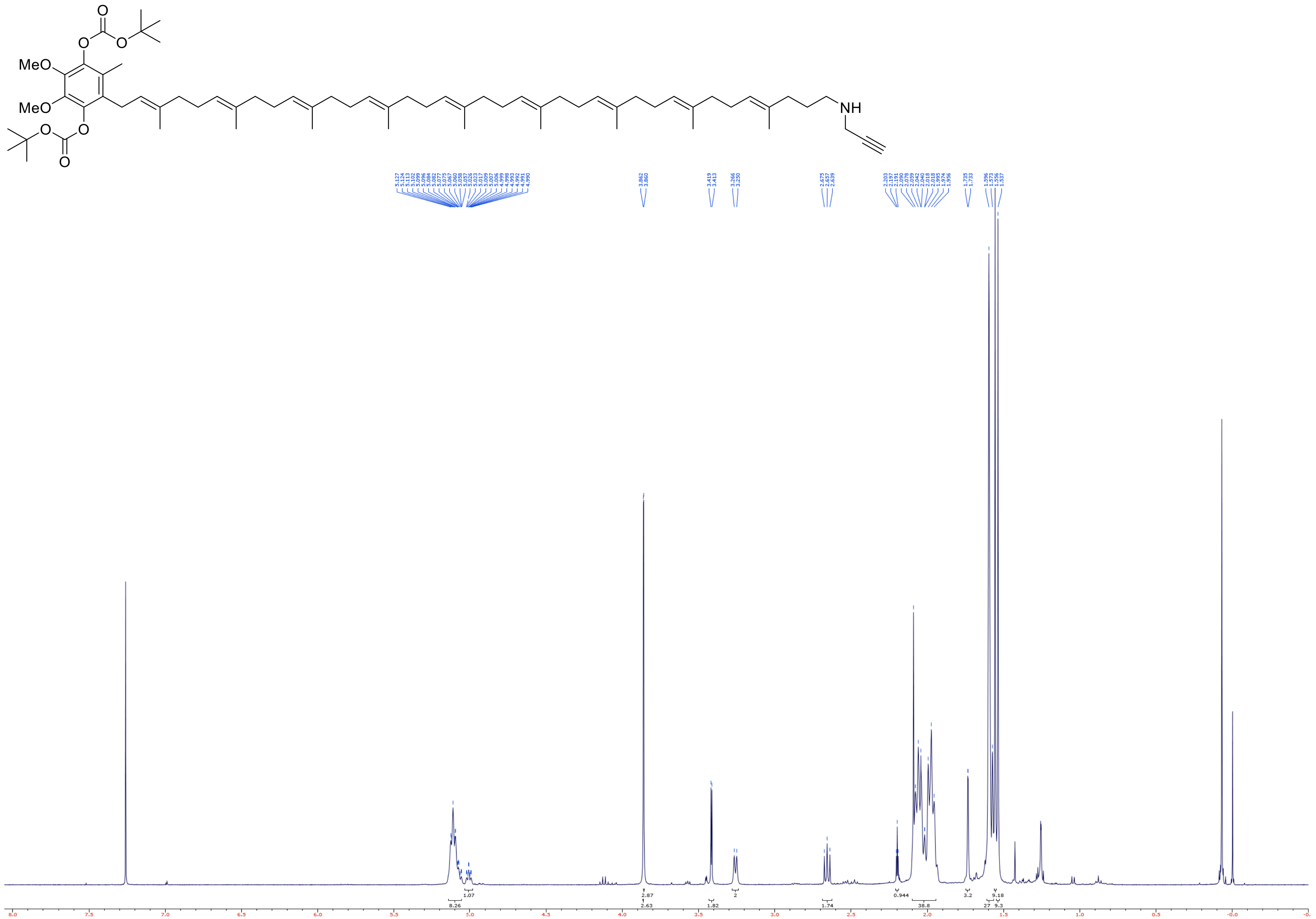

^{13}C NMR (101 MHZ, $CDCl_3$) for Boc_2-CoQ10-ω-alkyne (19a)

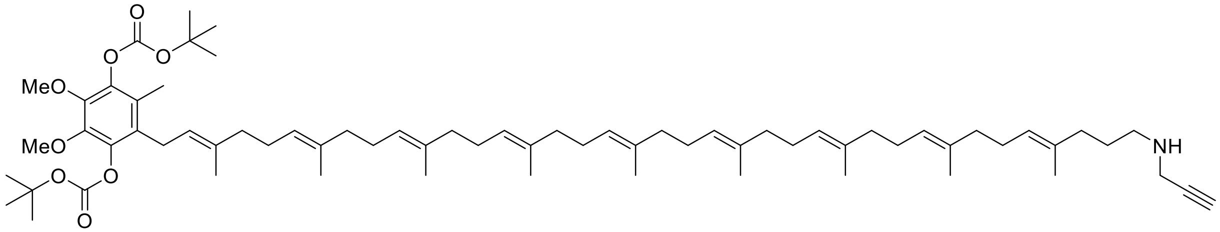

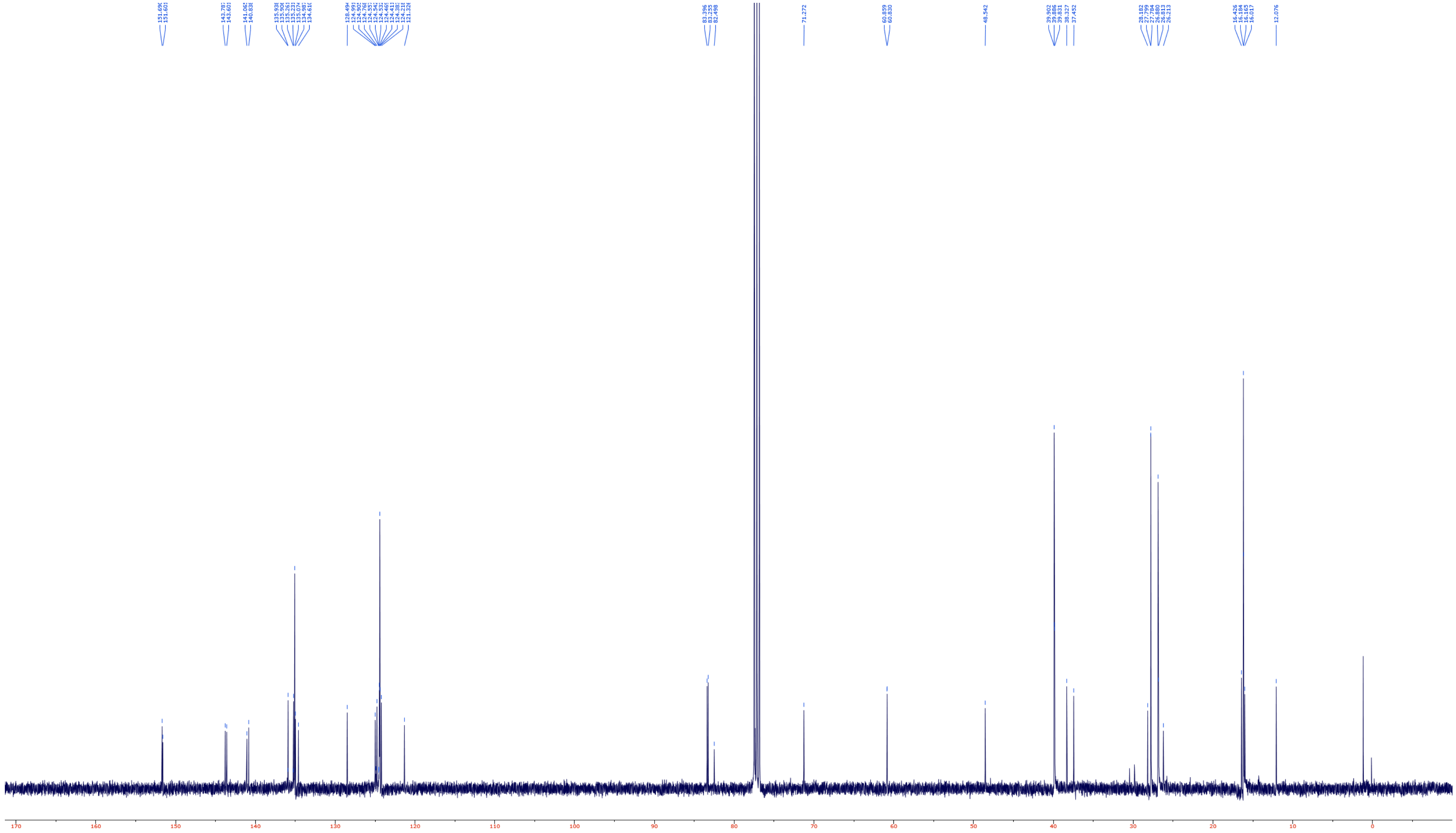

2D COSY (400 MHZ, $CDCl_3$) for Boc_2-CoQ10-ω-alkyne (19a)

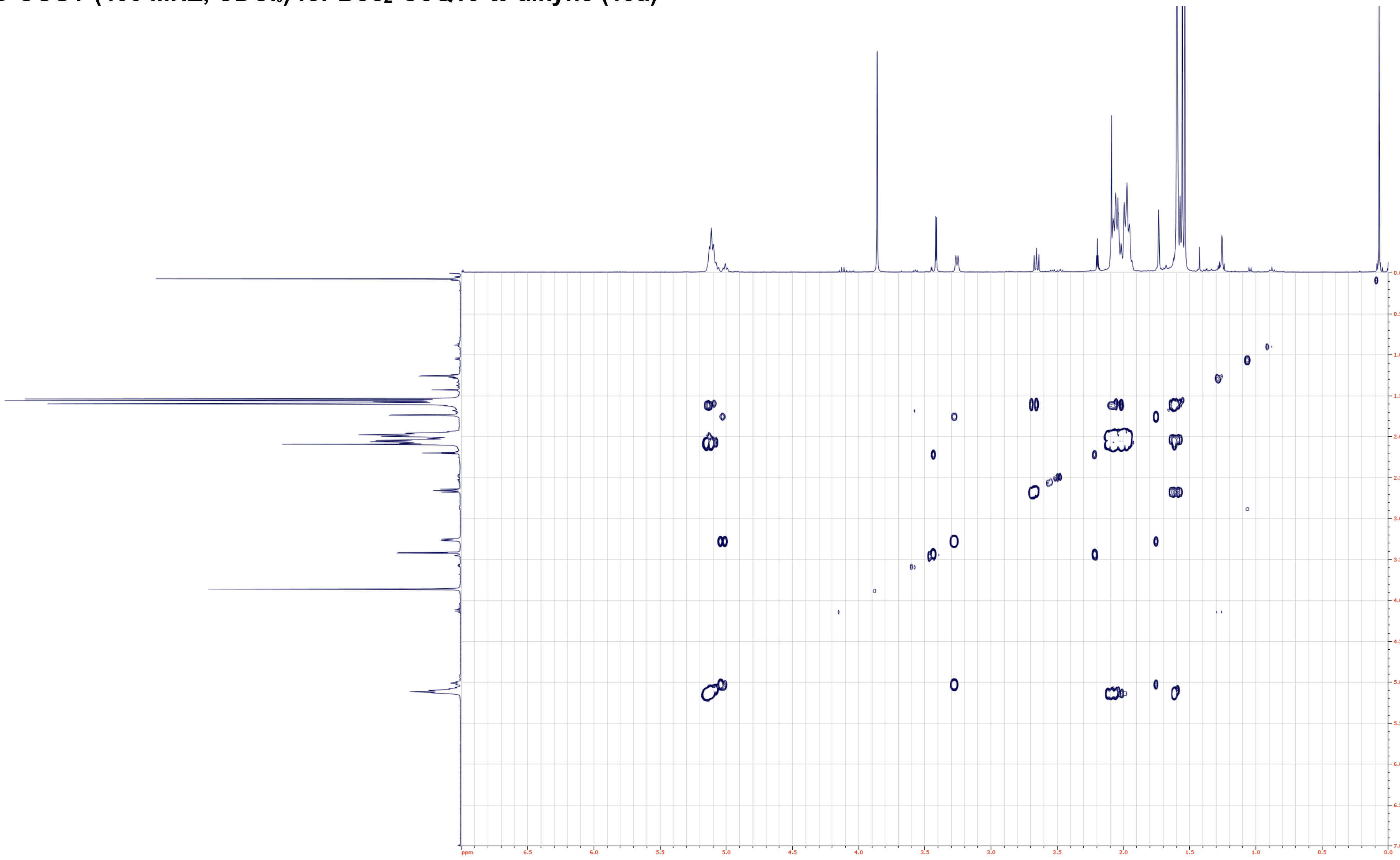

2D HSQC (400 MHZ, $CDCl_3$) for Boc_2-CoQ10-ω-alkyne (19a)

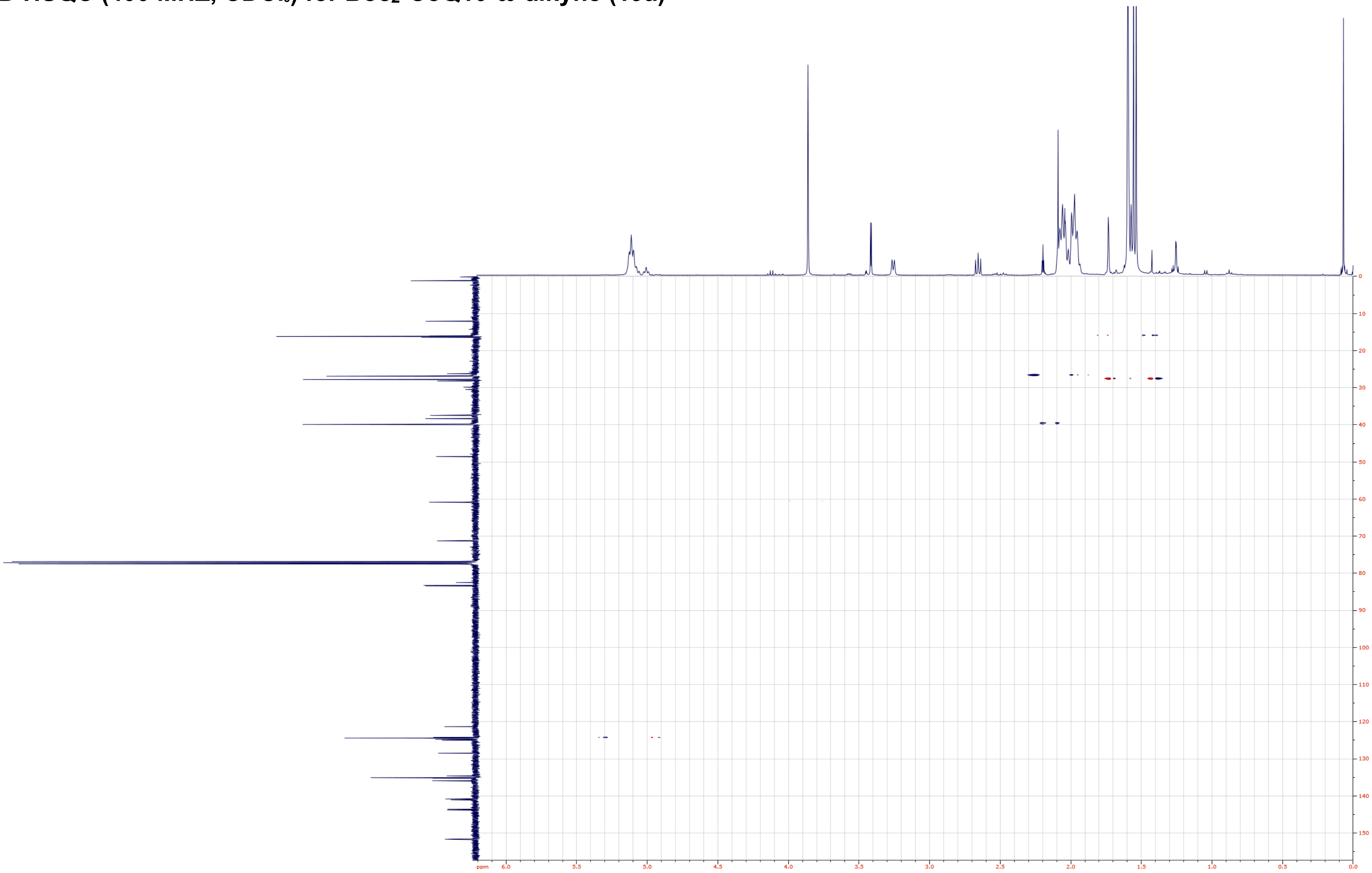

2D HMBC (400 MHZ, $CDCl_3$) for Boc_2-CoQ10-ω-alkyne (19a)

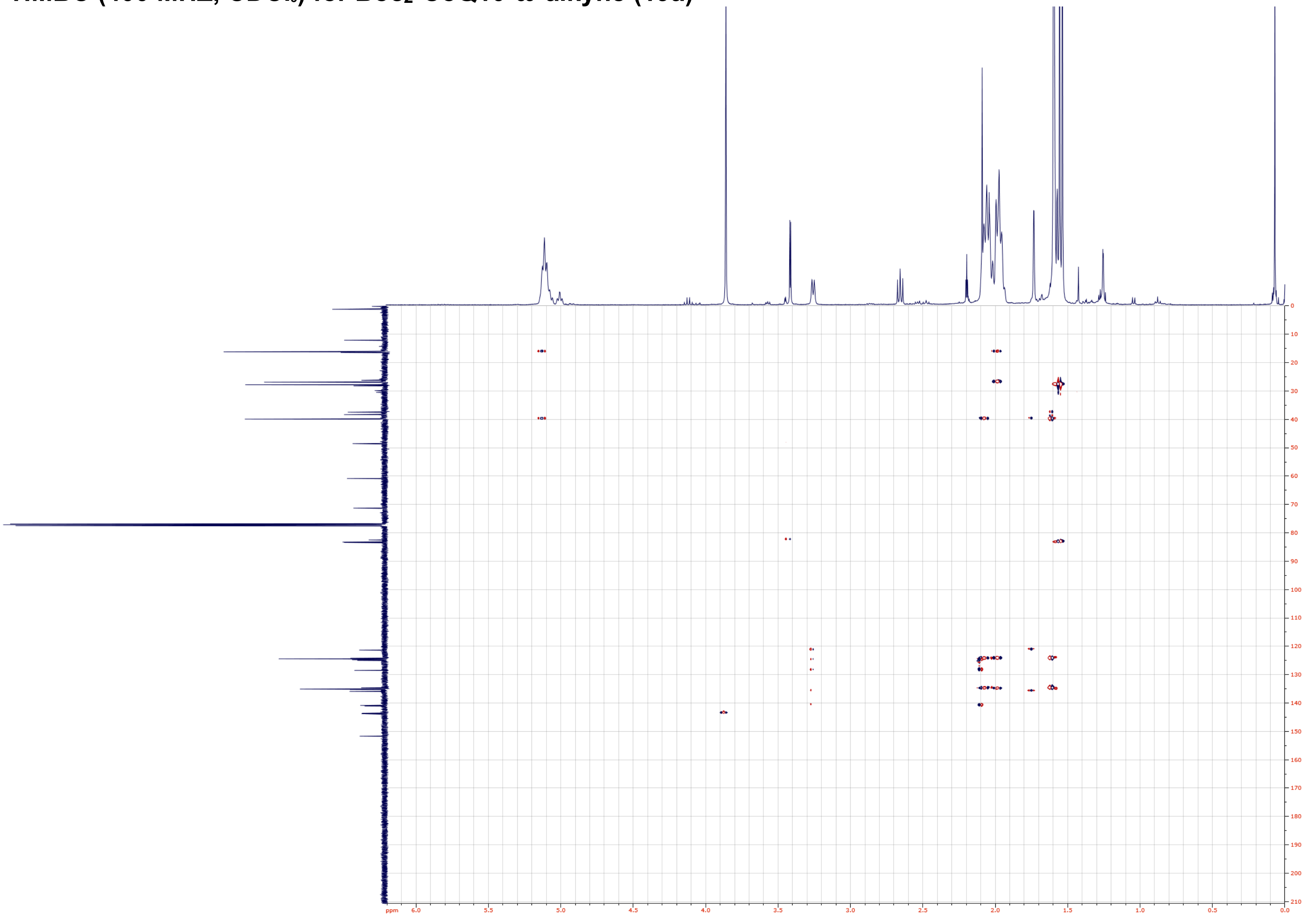

^{1}H NMR (101 MHZ, CDCl$_3$) for Boc$_2$-CoQ10-ω-CF$_3$ (20a)

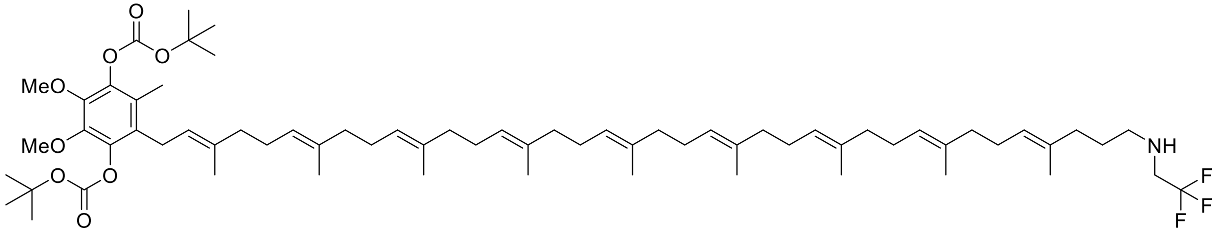

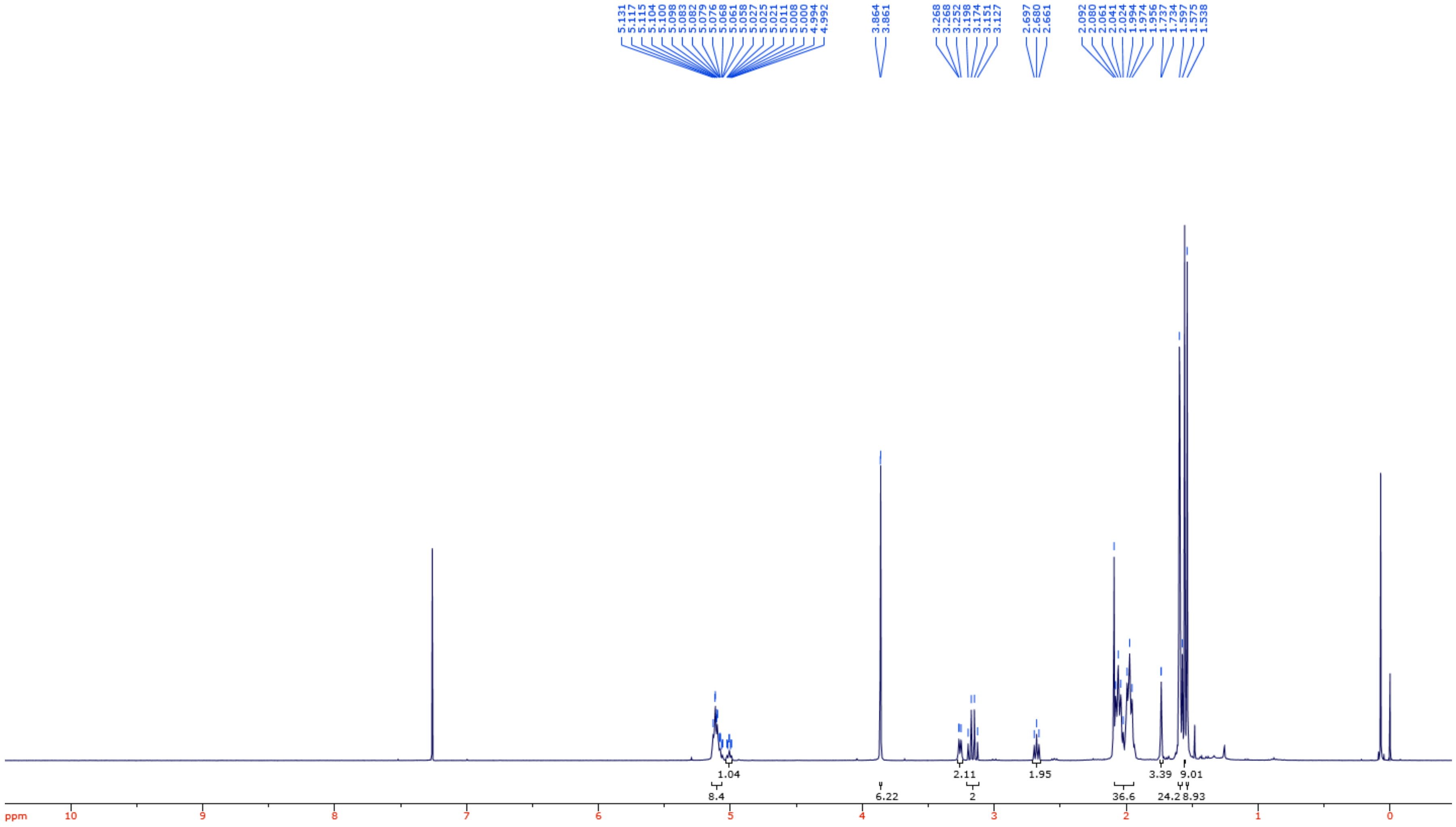

^{13}C NMR (101 MHZ, $CDCl_3$) for Boc_2-CoQ10-ω-CF_3 (20a)

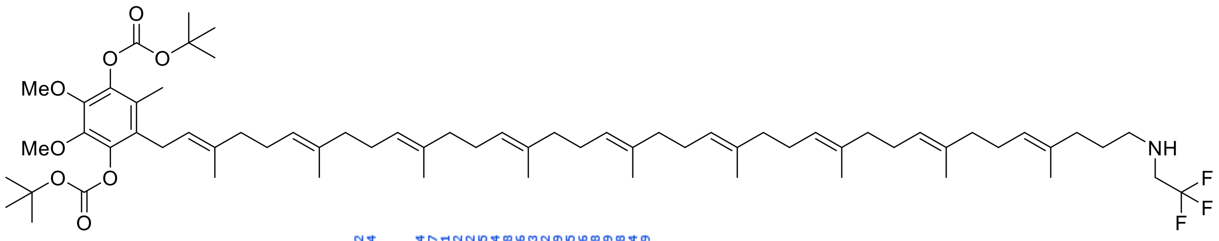

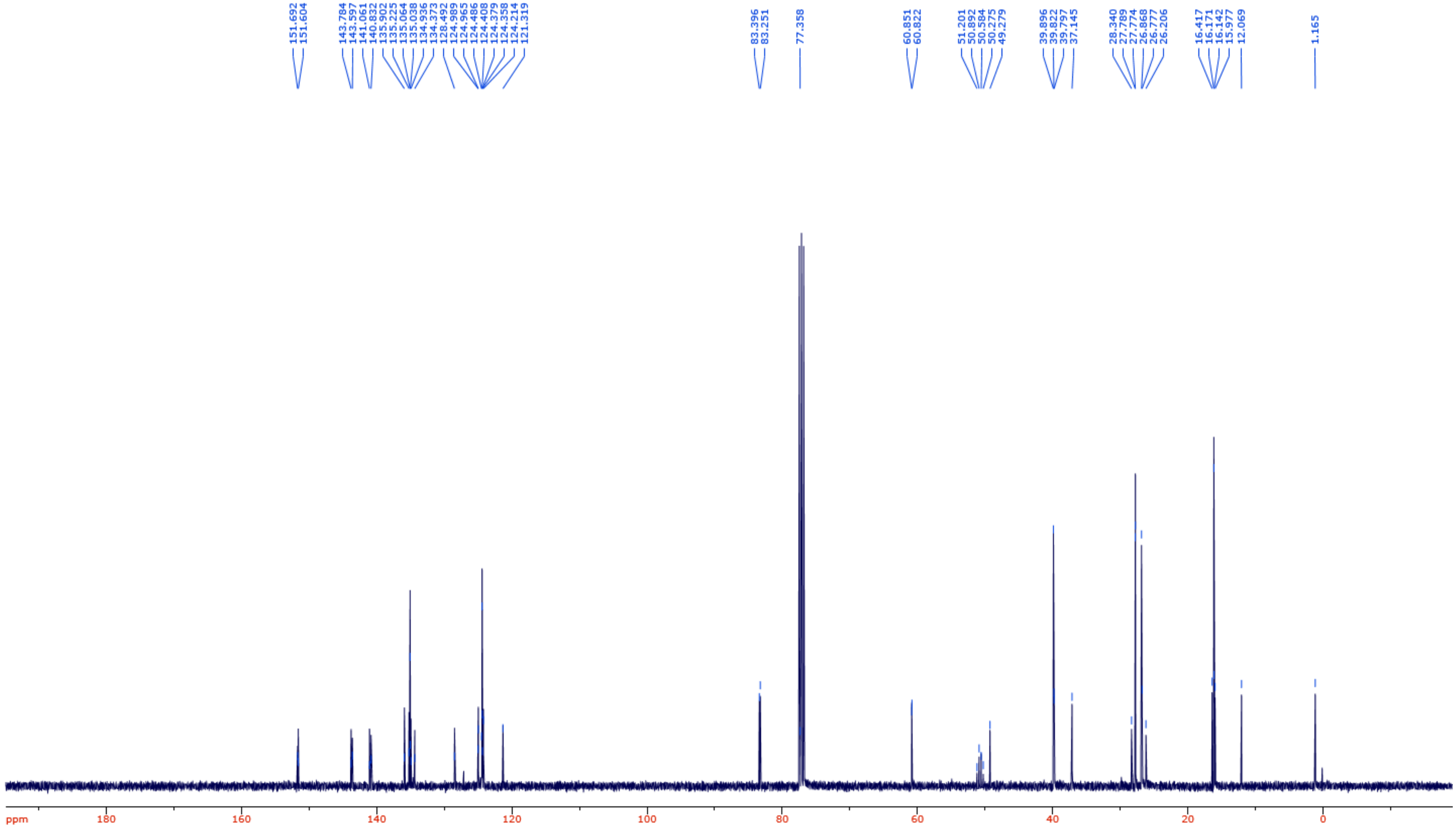

2D COSY (400 MHZ, $CDCl_3$) for Boc_2-CoQ10-ω-CF_3 (20a)

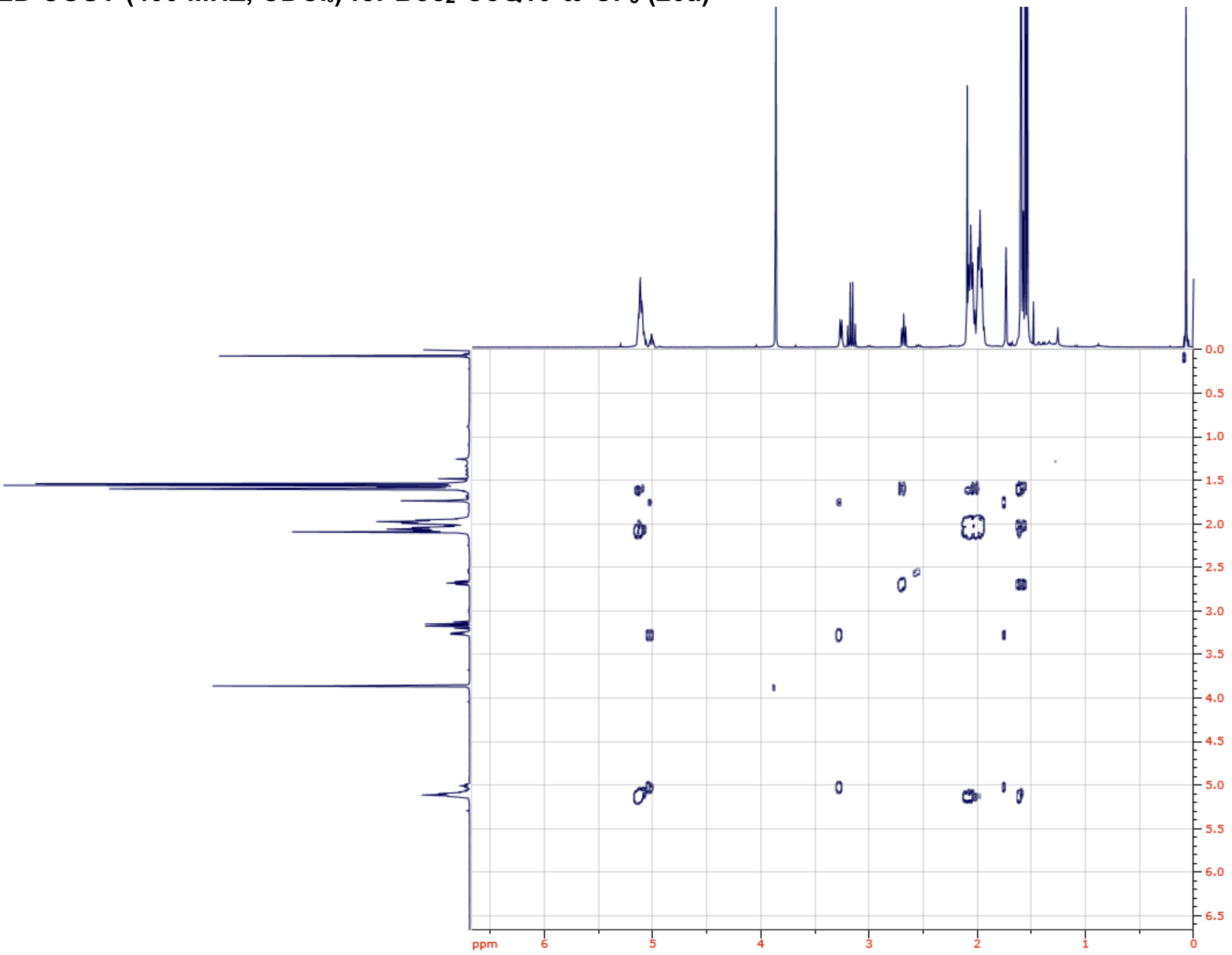

2D HSQC (400 MHZ, $CDCl_3$) for Boc_2-CoQ10-ω-CF_3 (20a)

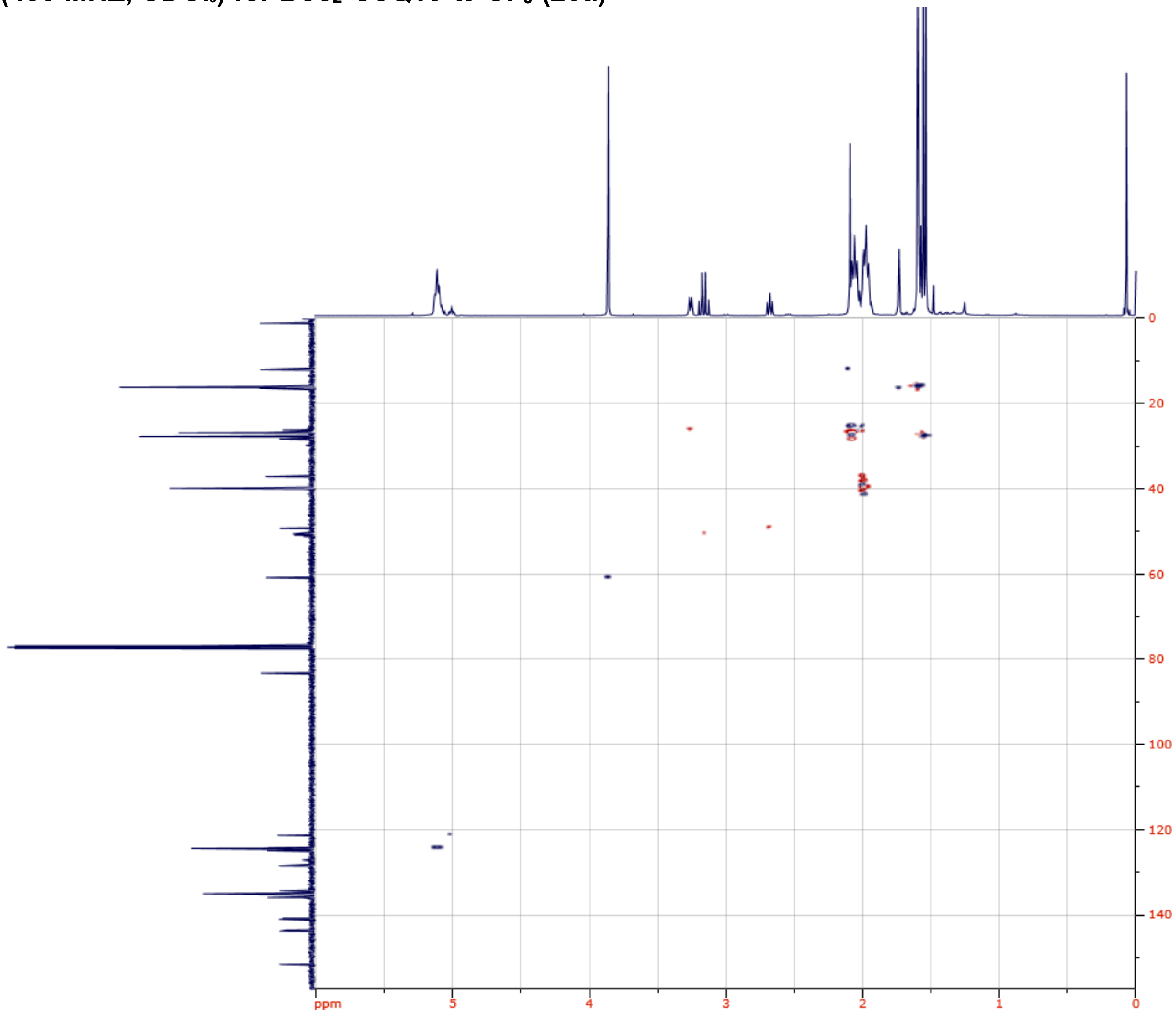

2D HMBC (400 MHZ, $CDCl_3$) for Boc_2-CoQ10-ω-CF_3 (20a)

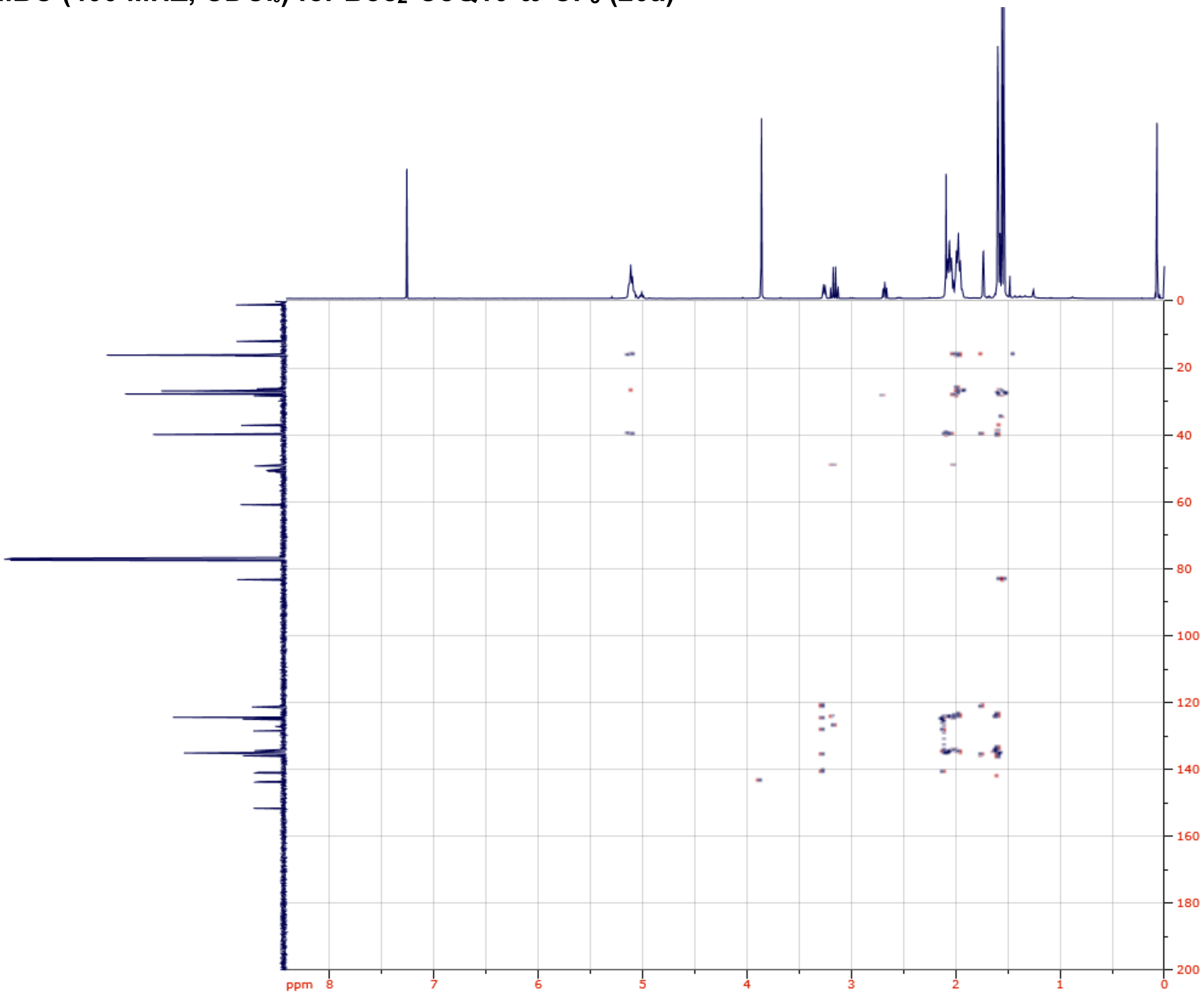

^{19}F NMR (376 MHZ, $CDCl_3$) for Boc_2-CoQ10-ω-CF_3 (20a)

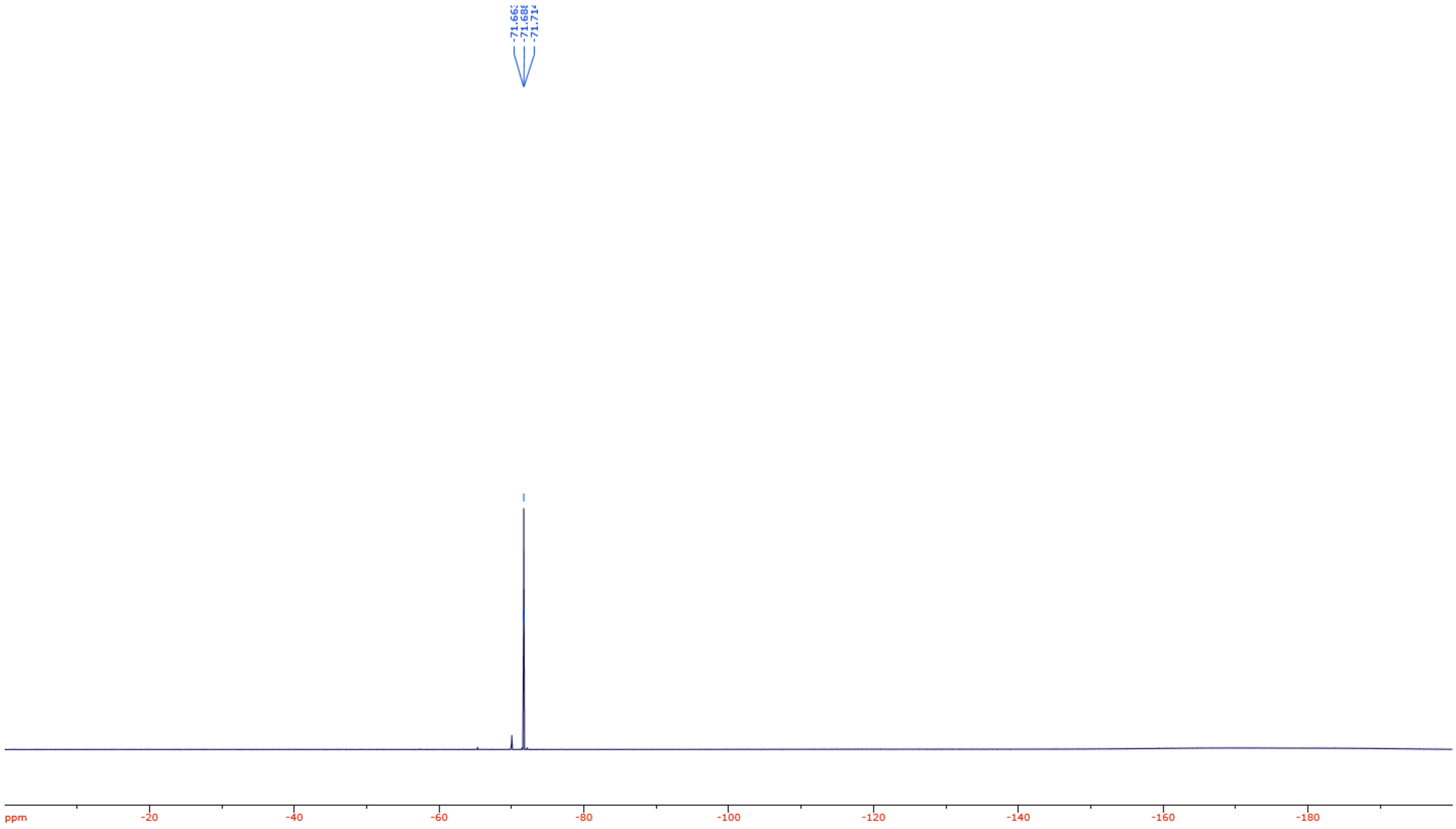

^{1}H NMR (400 MHZ, CDCl3) for Boc2-CoQ10-ω-pyrene (21a)

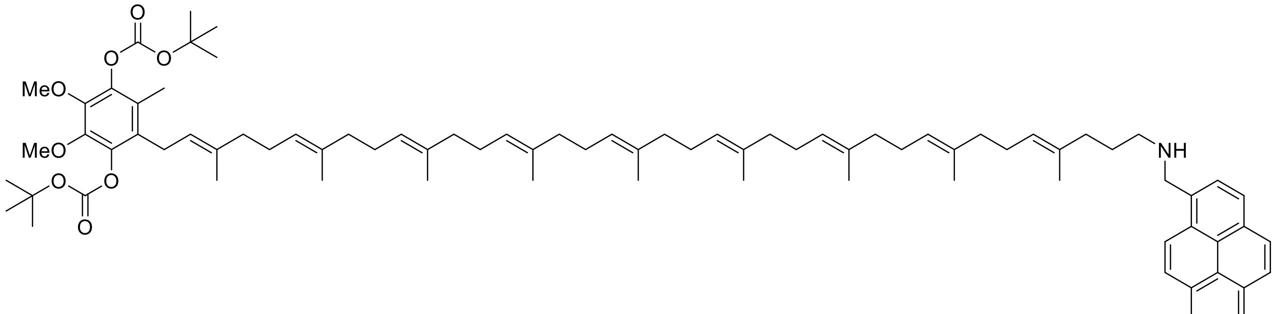

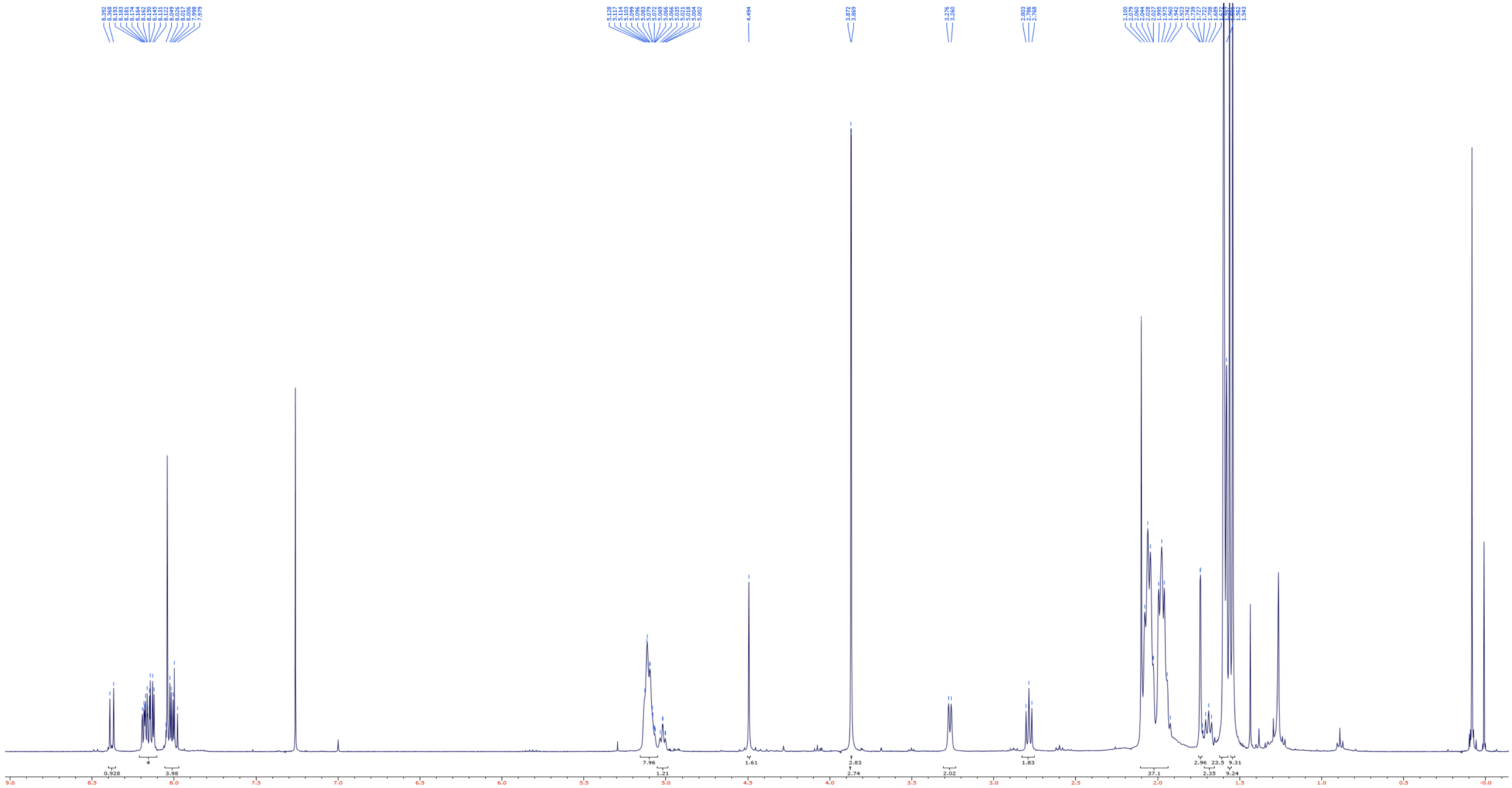

^{13}C NMR (101 MHZ, $CDCl_3$) for Boc_2-CoQ10-ω-pyrene (21a)

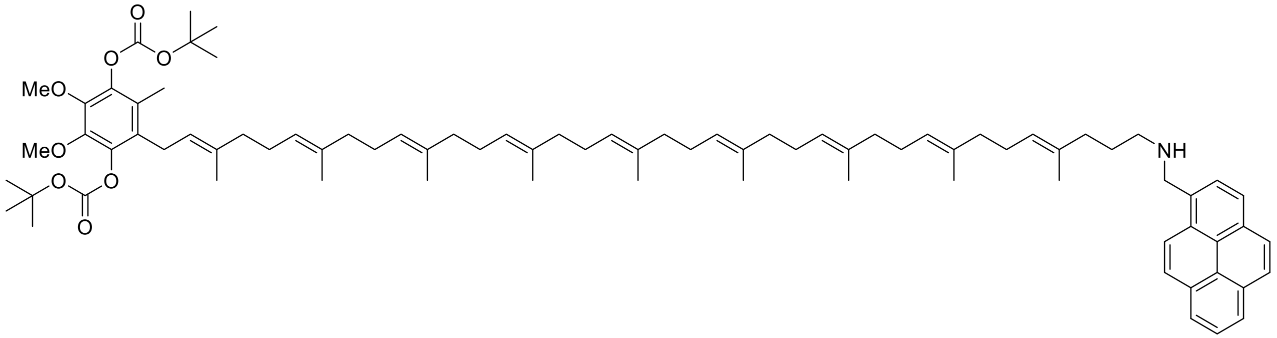

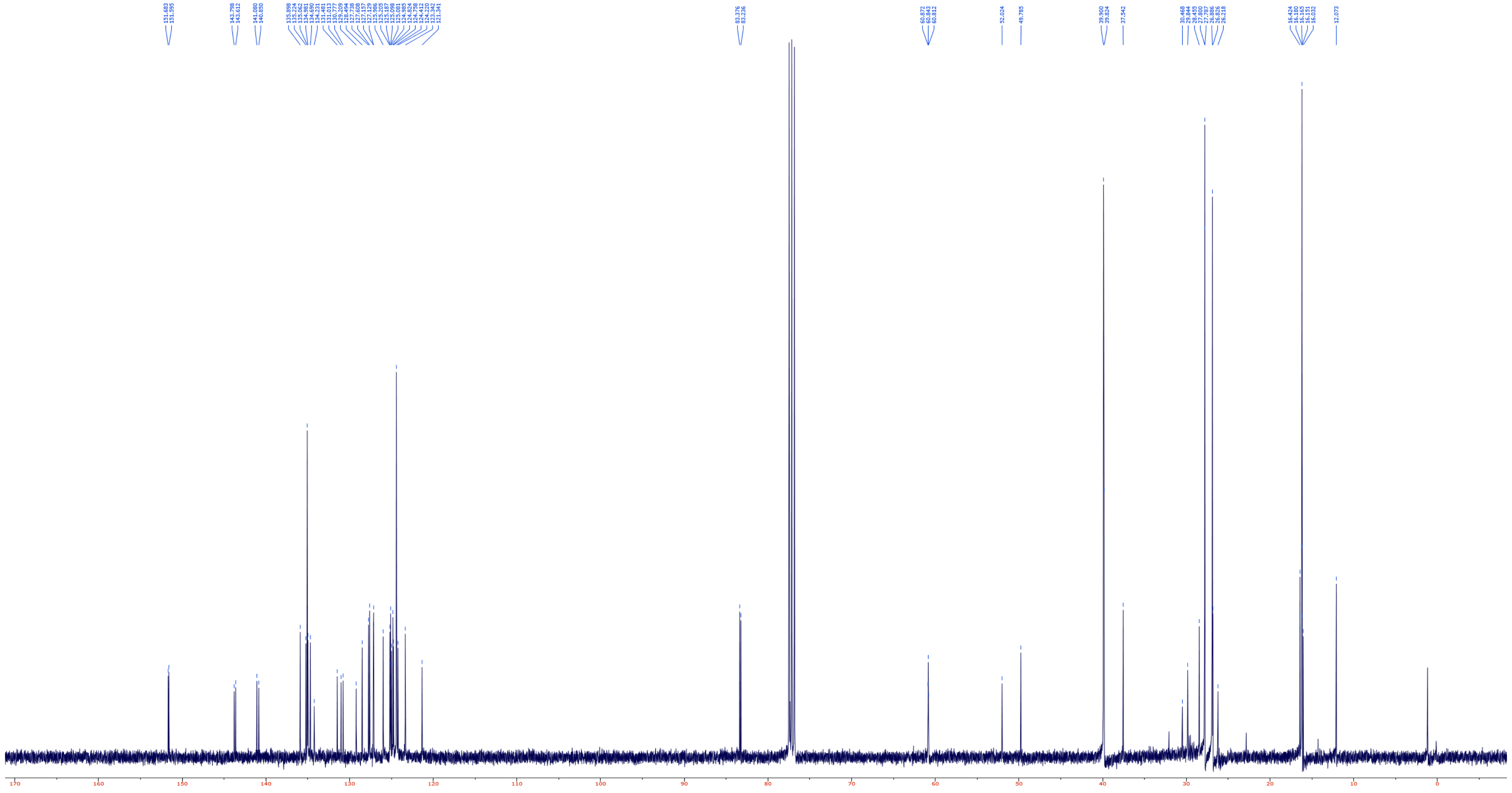

2D COSY (400 MHZ, $CDCl_3$) for Boc_2-CoQ10-ω-pyrene (21a)

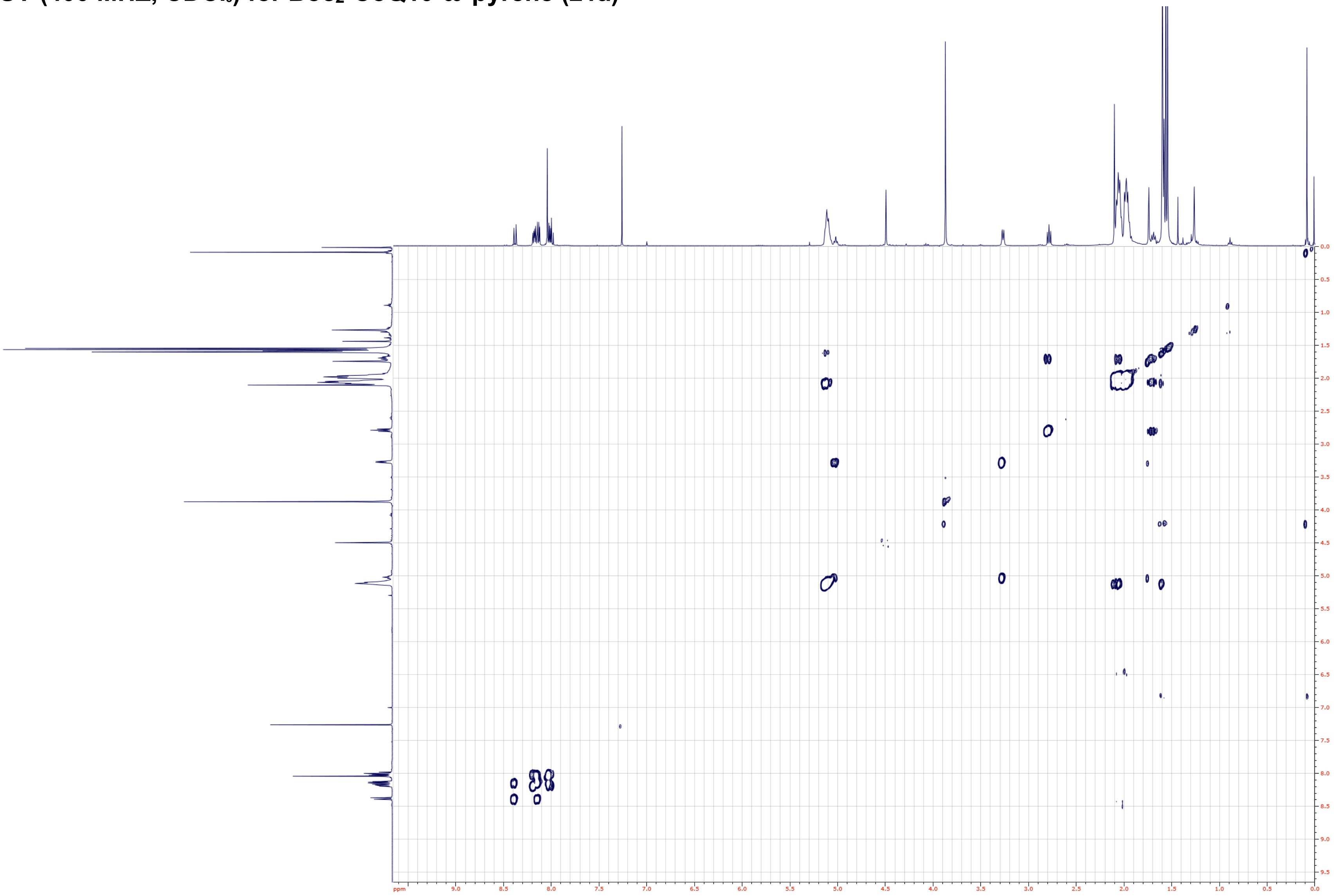

2D HSQC (400 MHZ, $CDCl_3$) for Boc_2-CoQ10-ω-pyrene (21a)

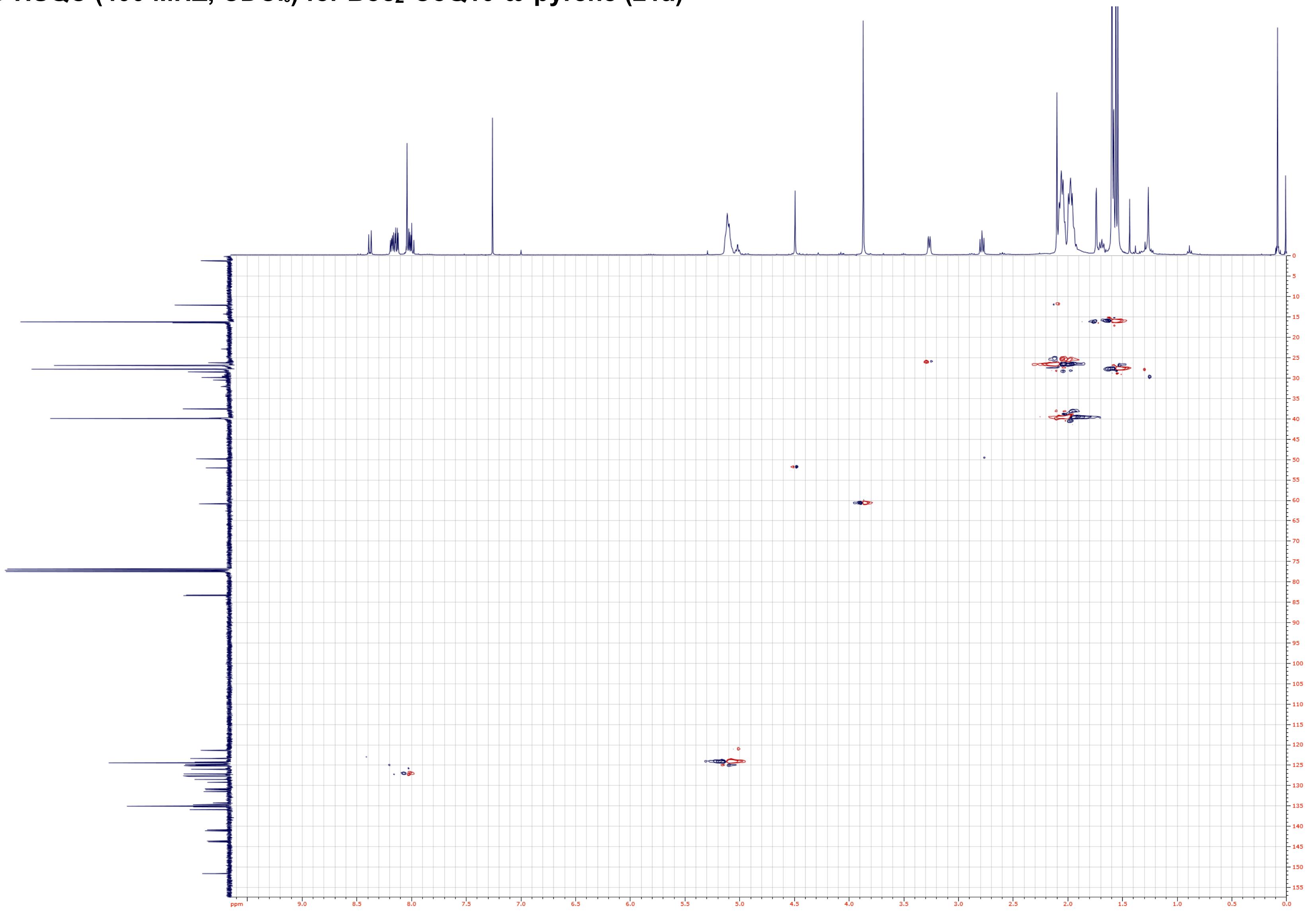

2D HMBC (400 MHZ, $CDCl_3$) for Boc_2-CoQ10-ω-pyrene (21a)

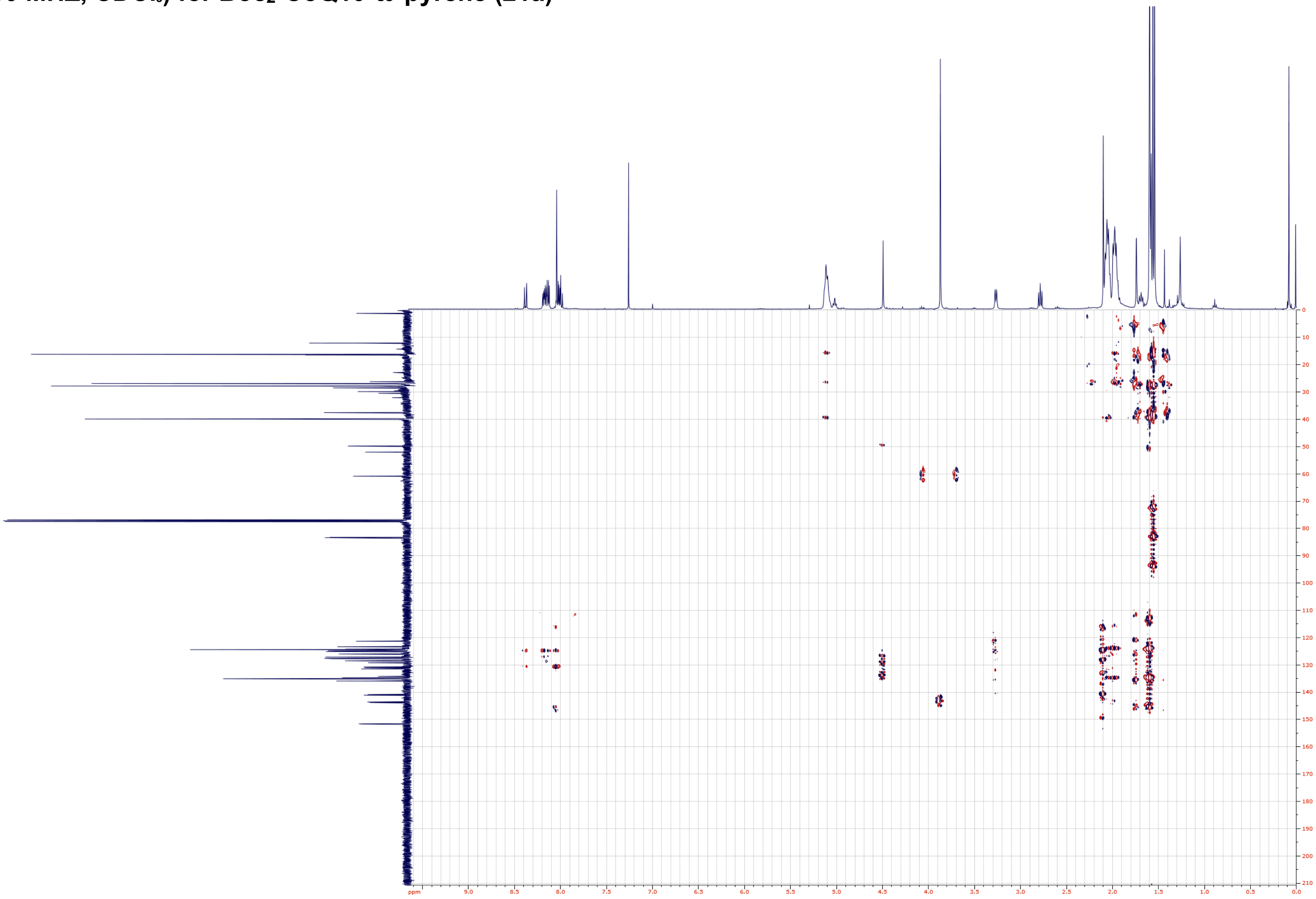

^{1}H NMR (400 MHZ, $CDCl_3$) for Boc_2-CoQ10-ω-PNB (22a)

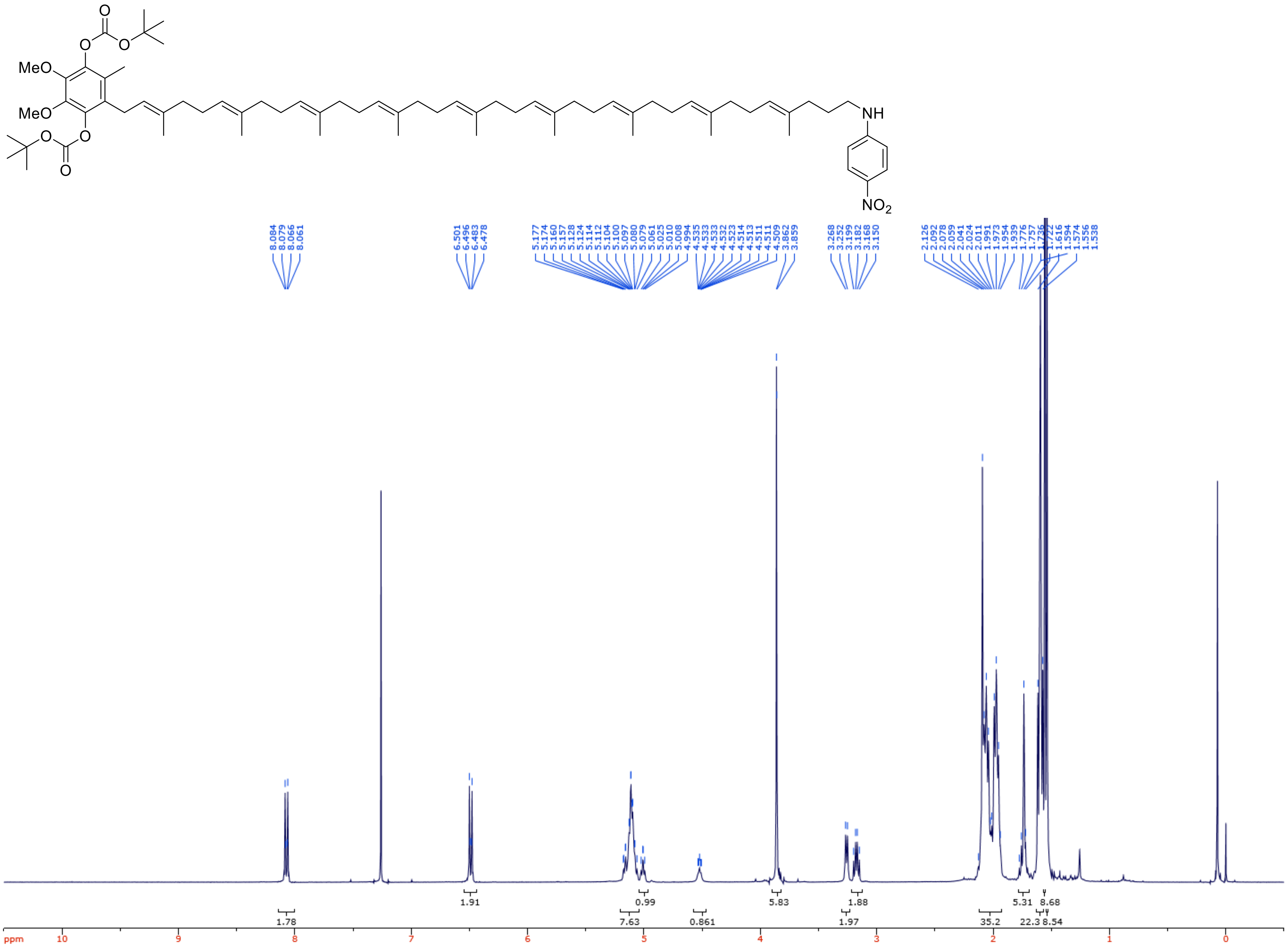

^{13}C NMR (101 MHZ, CDCl$_3$) for Boc$_2$-CoQ10-ω-PNB (22a)

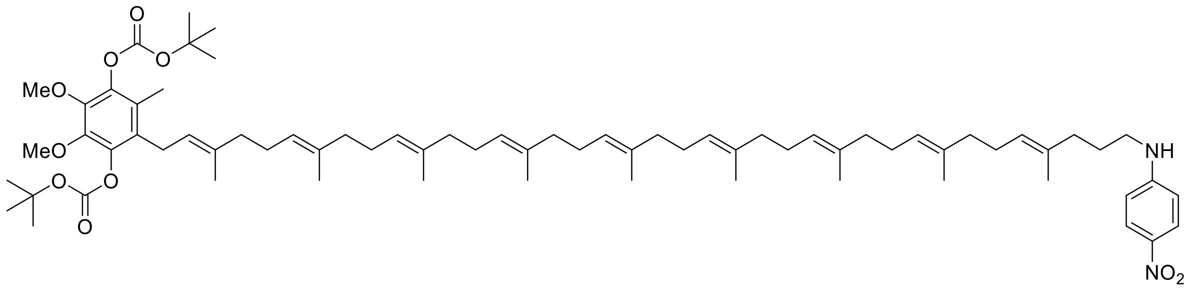

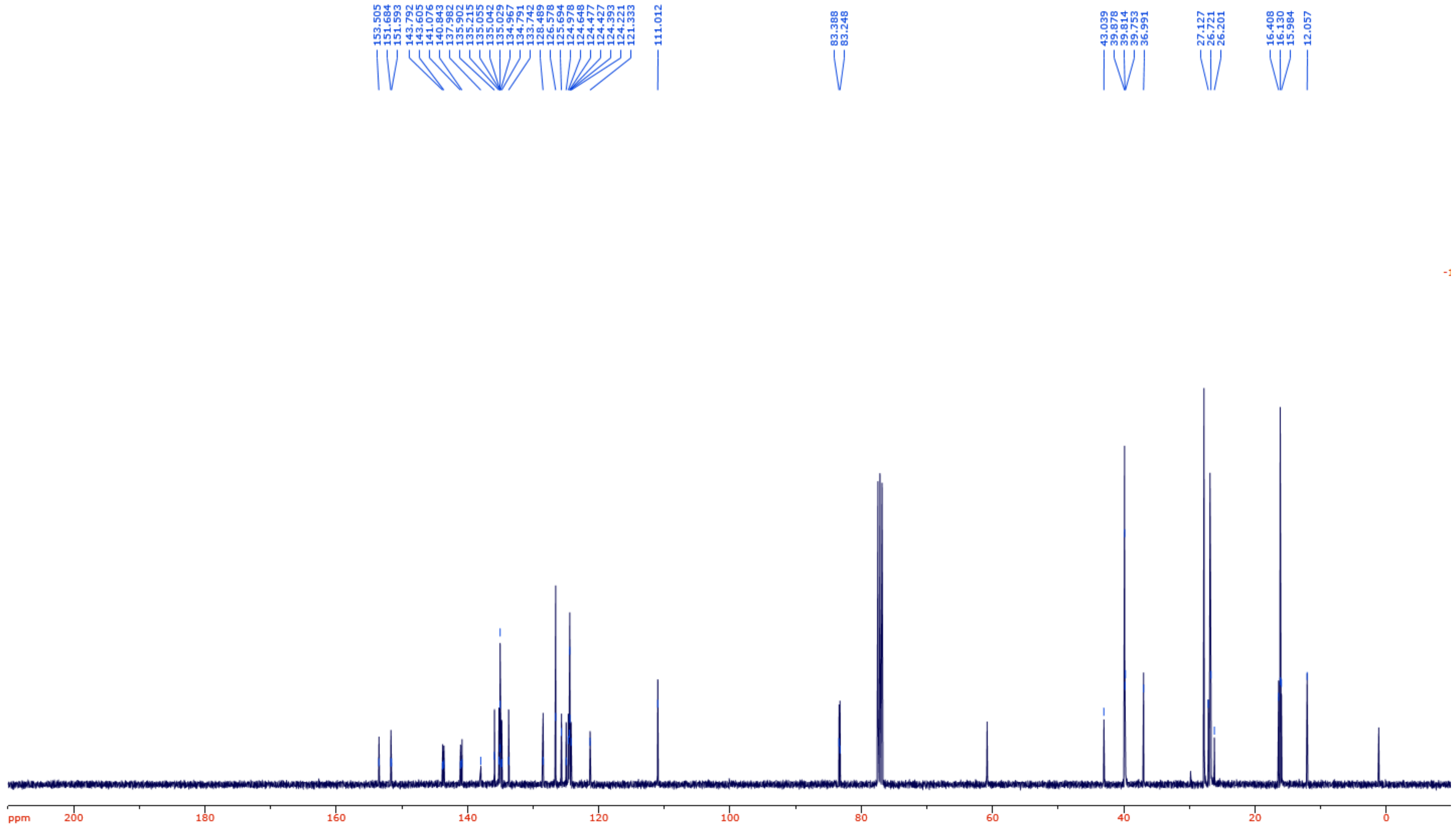

2D COSY (400 MHZ, $CDCl_3$) for Boc_2-CoQ10-ω-PNB (22a)

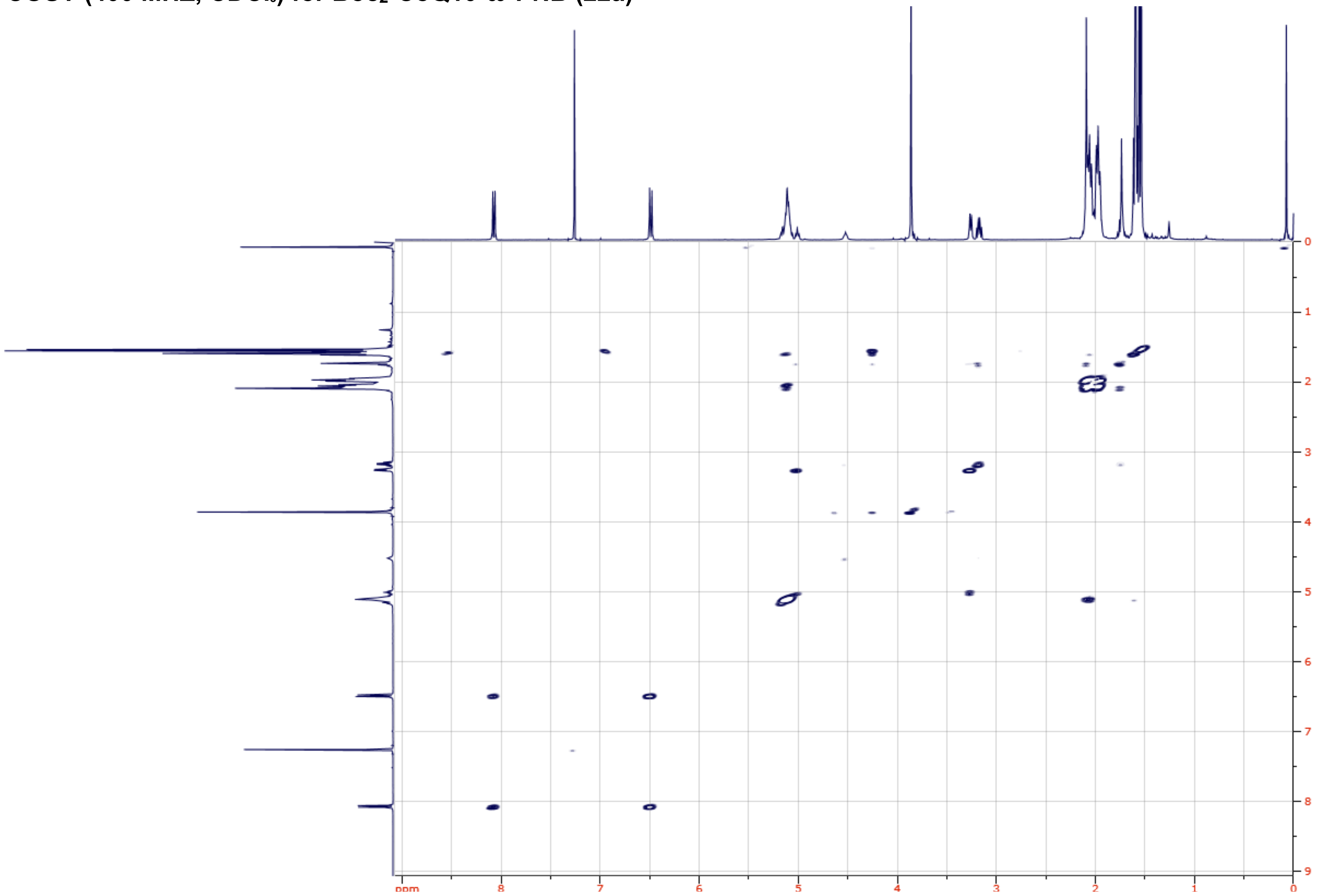

2D HSQC (400 MHZ, $CDCl_3$) for Boc_2-CoQ10-ω-PNB (22a)

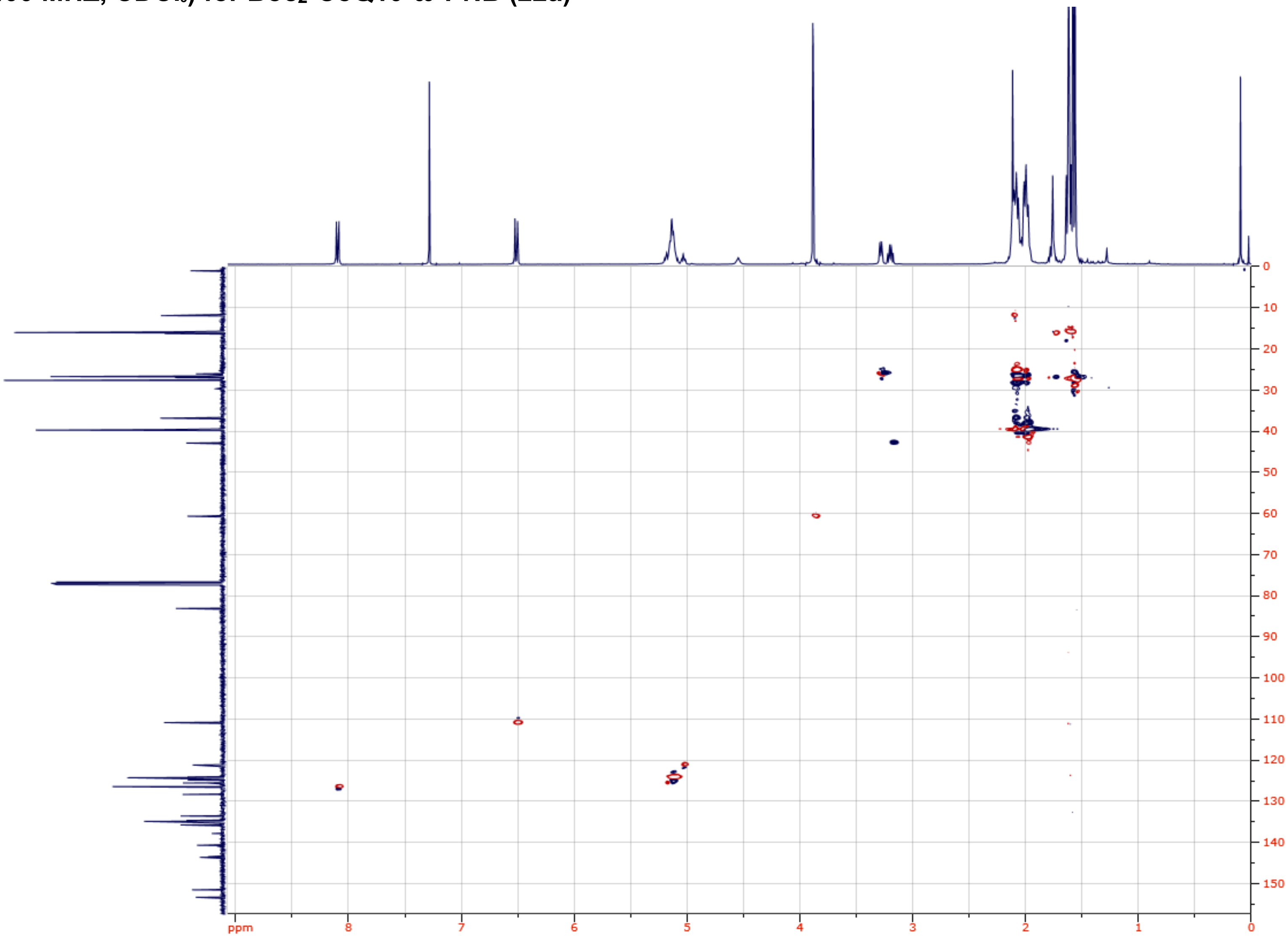

2D HMBC (400 MHZ, $CDCl_3$) for Boc_2-CoQ10-ω-PNB (22a)

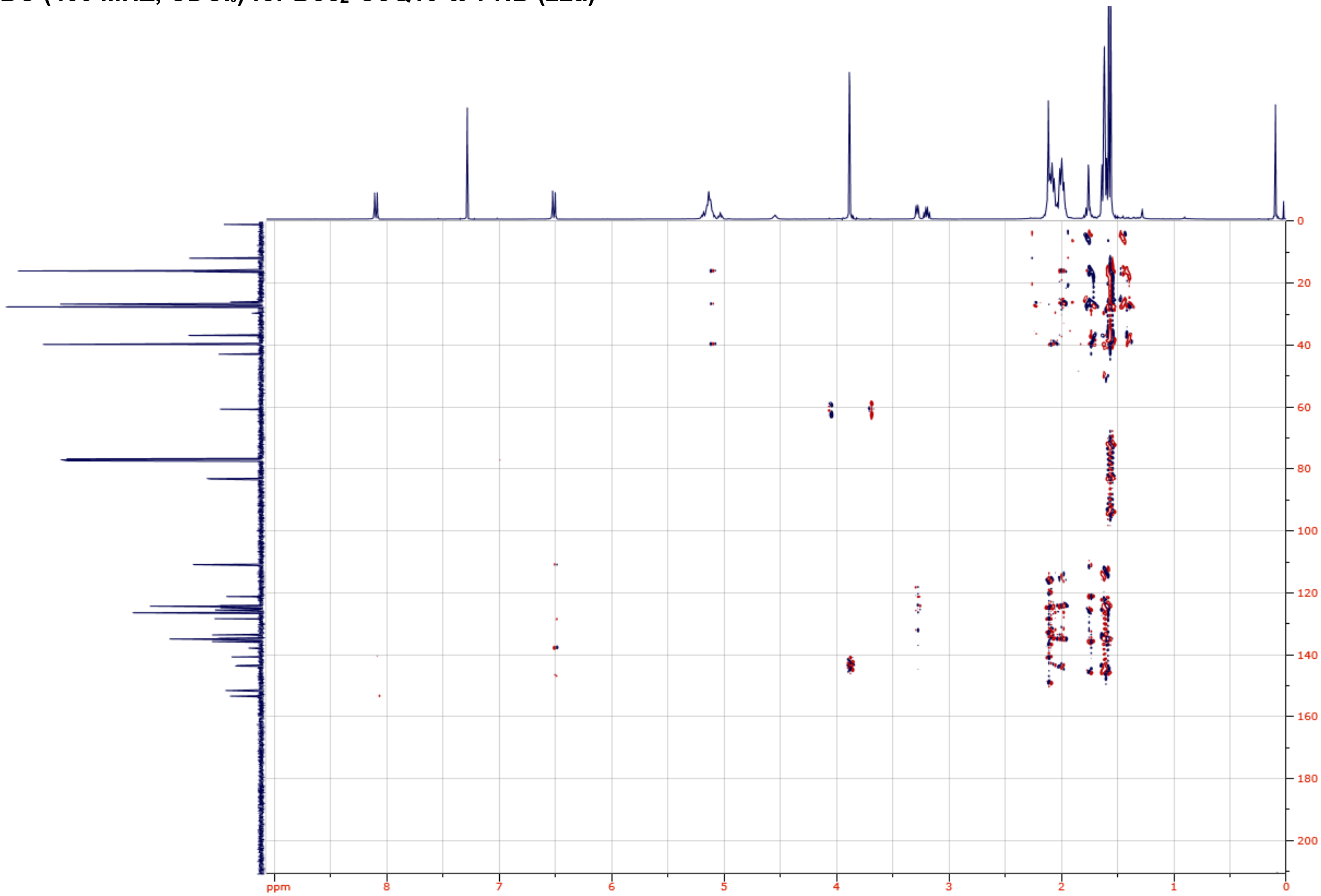

^{1}H NMR (400 MHZ, $CDCl_3$) for Boc_2-CoQ10-ω-diazirine (23a)

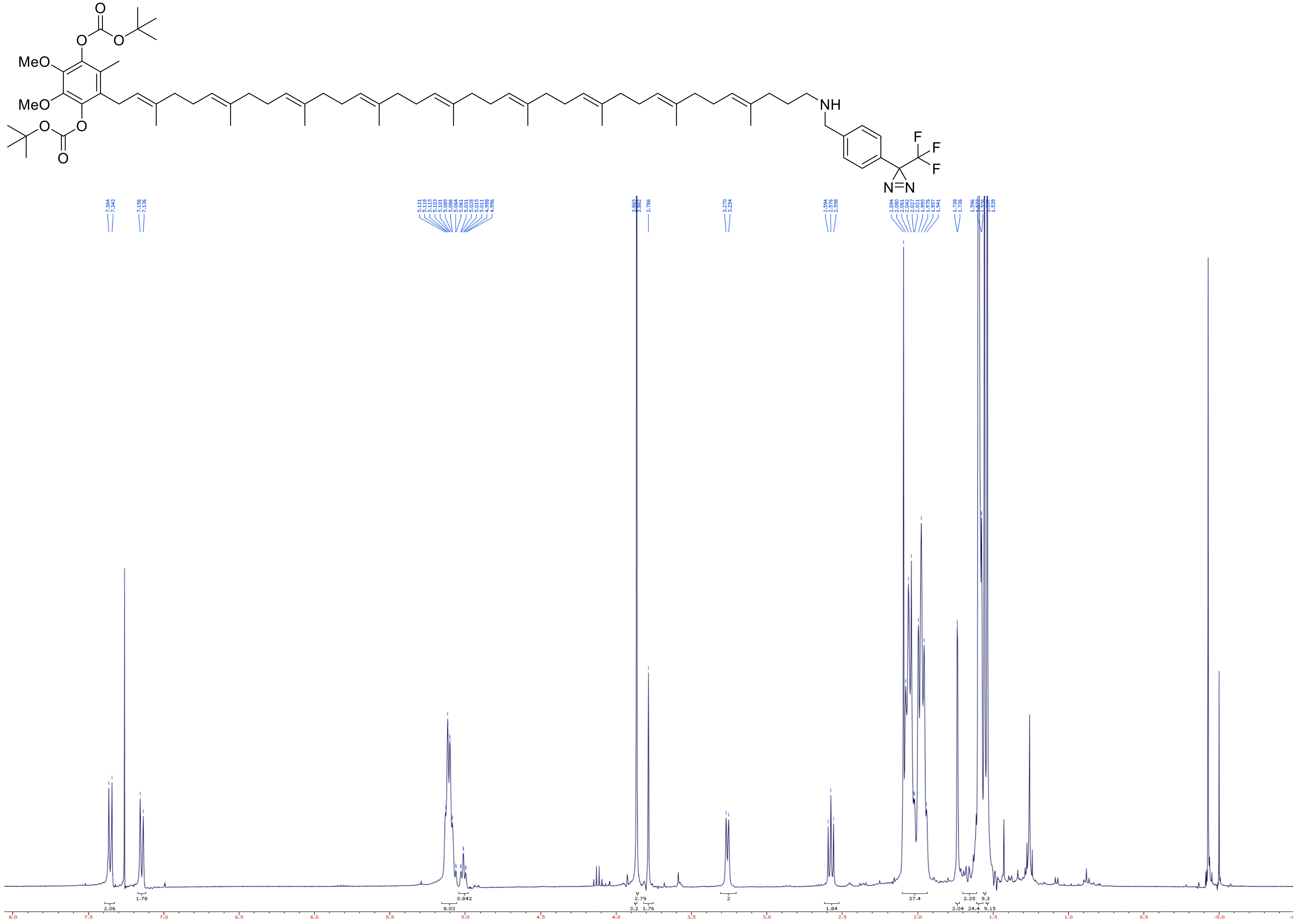

^{13}C NMR (101 MHZ, $CDCl_3$) Boc_2-CoQ10-ω-diazirine (23a)

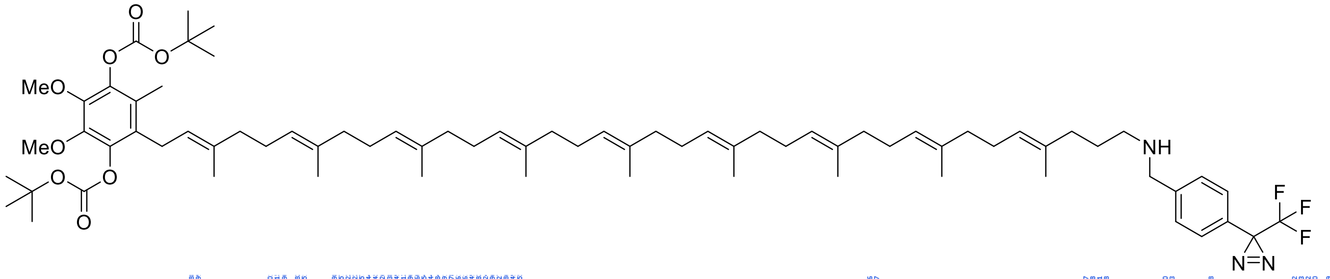

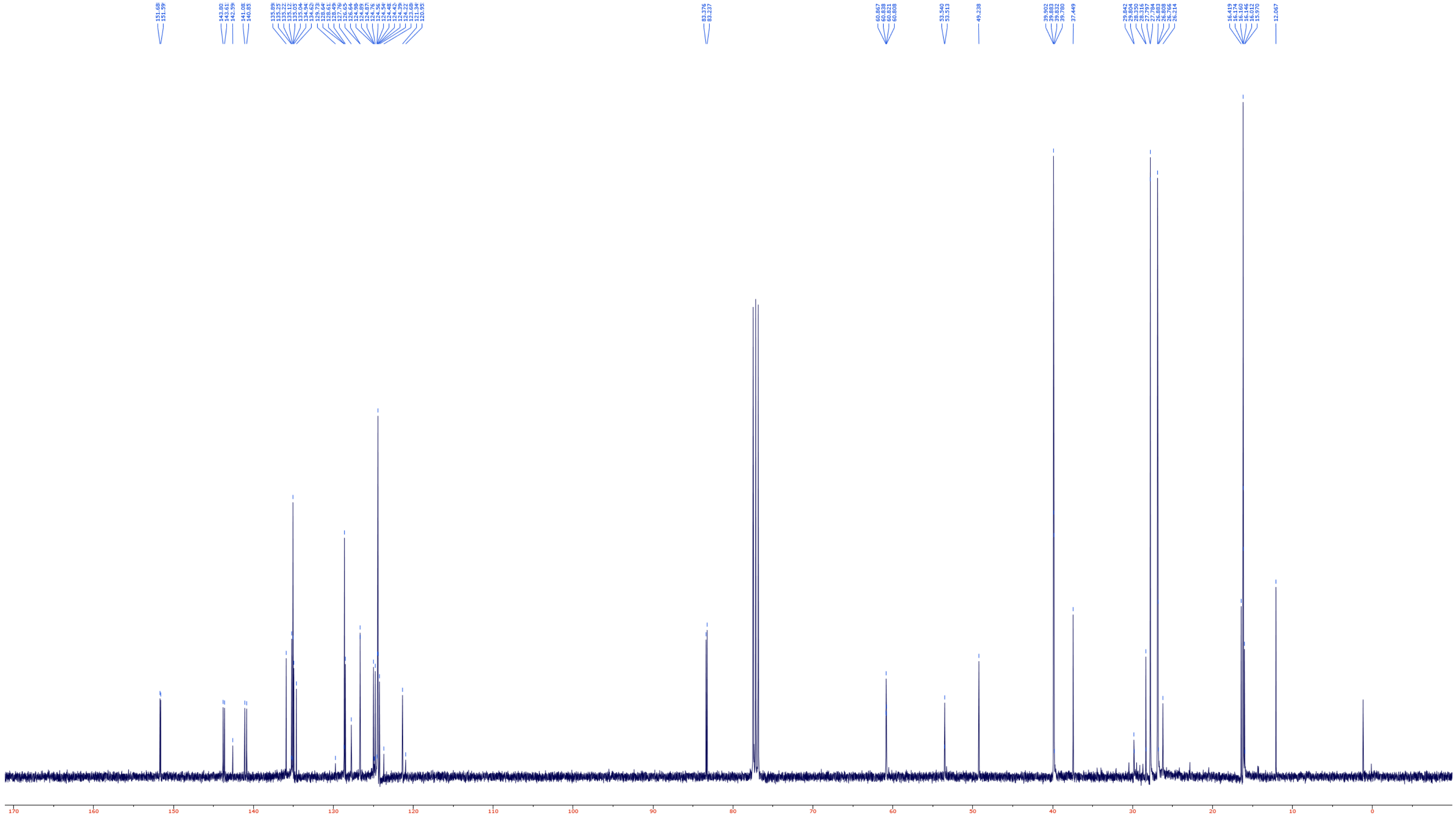

2D COSY (400 MHZ, $CDCl_3$) for Boc_2-CoQ10-ω-diazirine (23a)

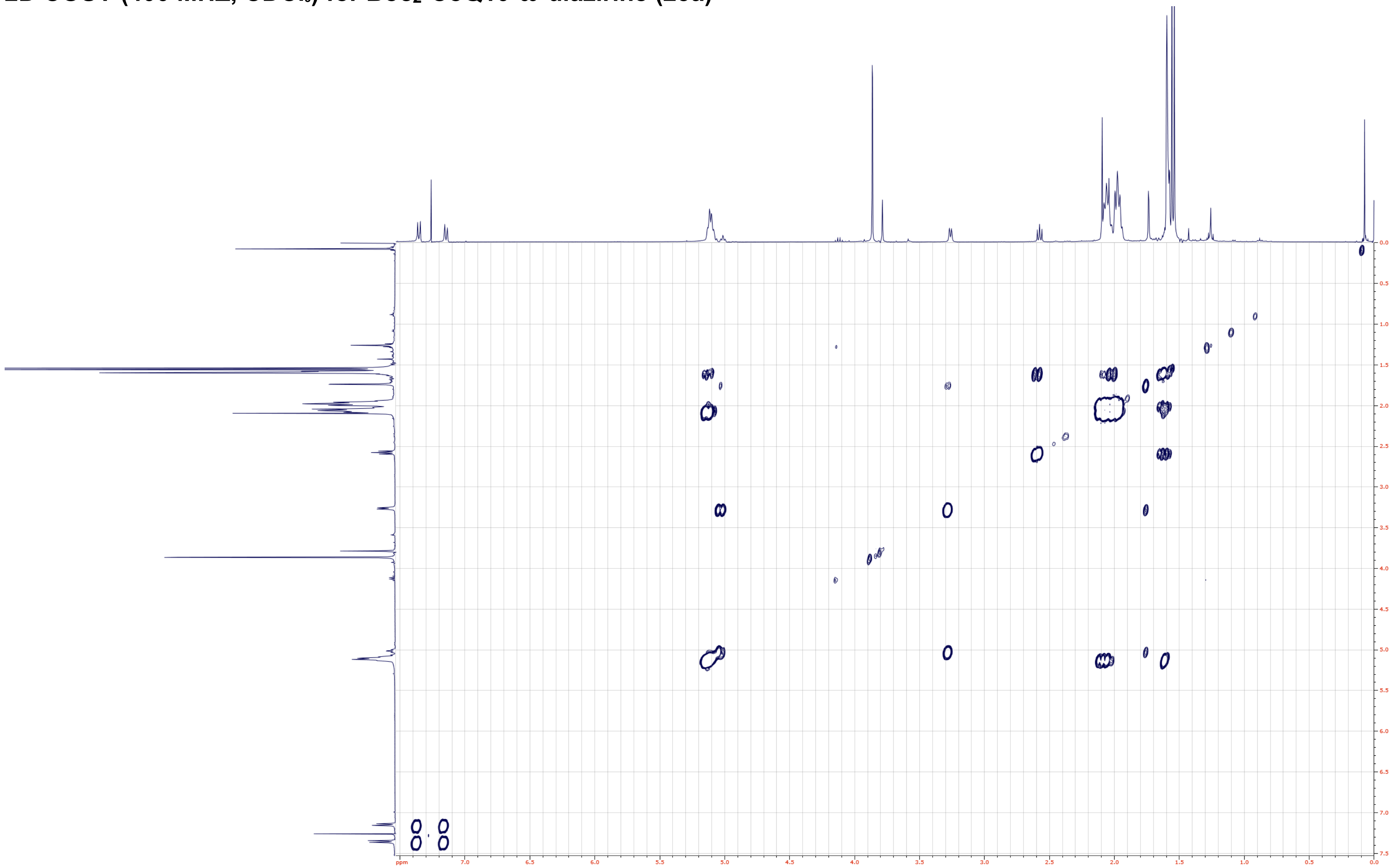

2D HSQC (400 MHZ, $CDCl_3$) for Boc_2-CoQ10-ω-diazirine (23a)

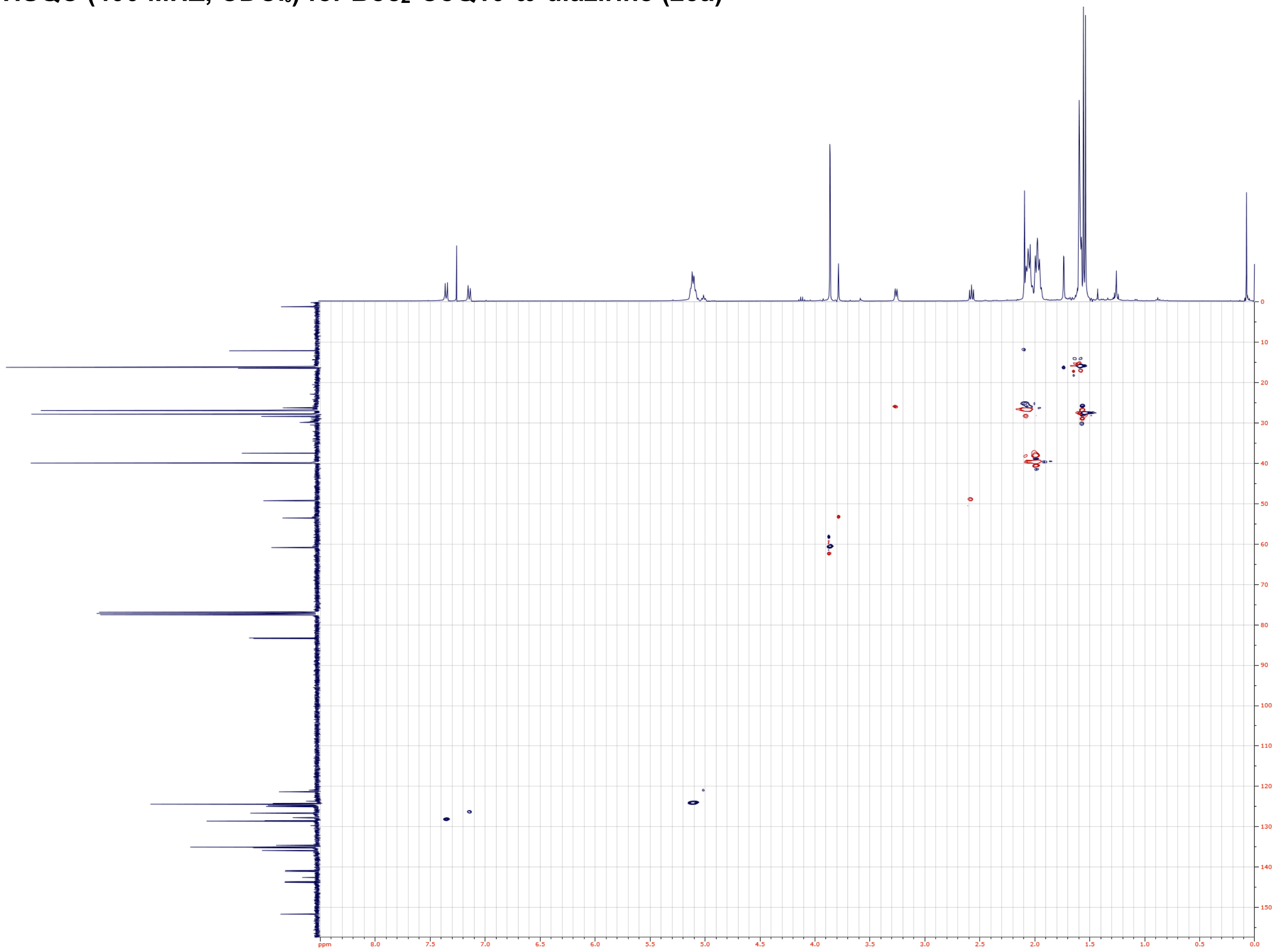

2D HMBC (400 MHZ, $CDCl_3$) for Boc_2-CoQ10-ω-diazirine (23a)

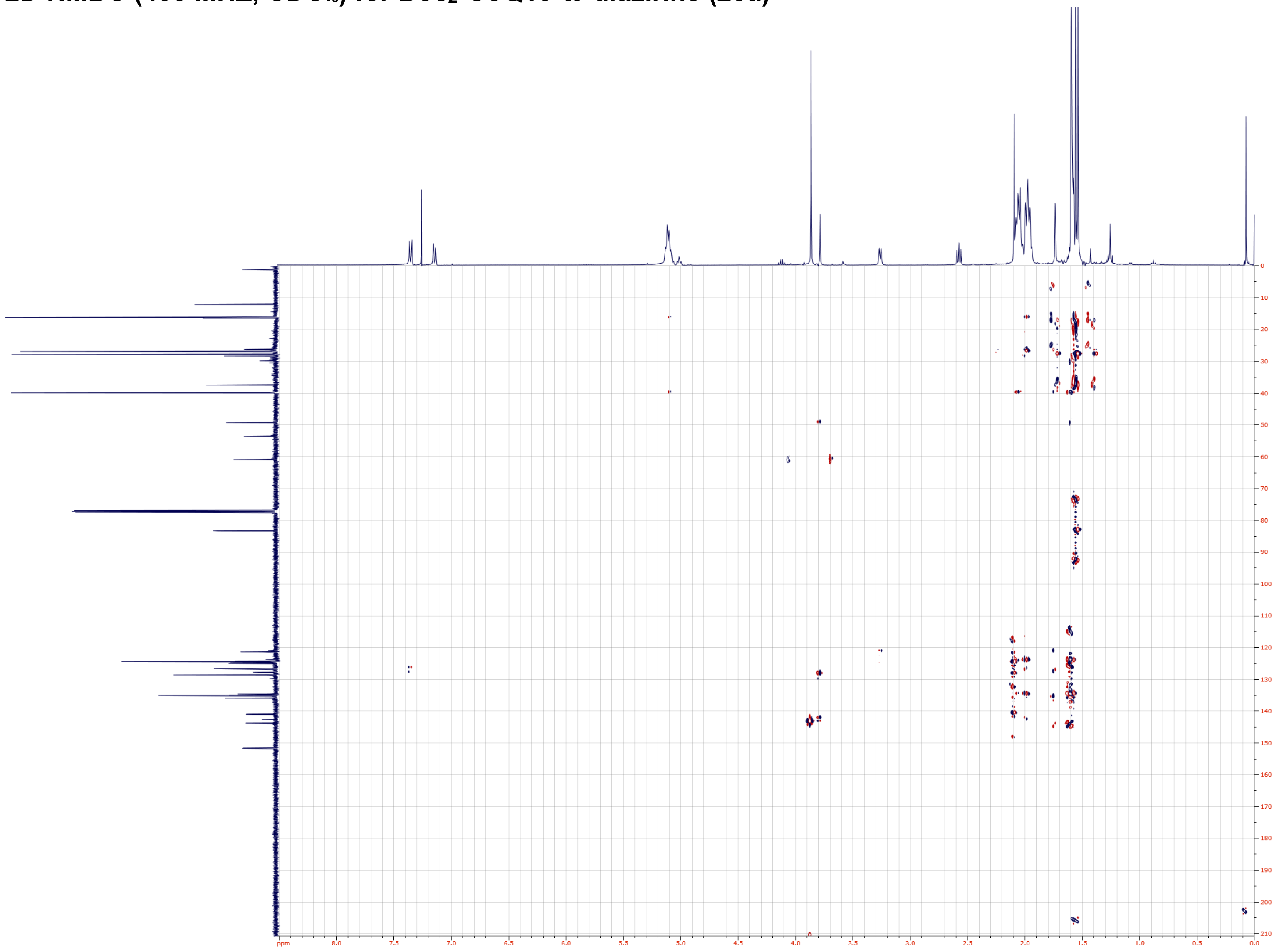

^{19}F NMR (376 MHZ, $CDCl_3$) for Boc_2-CoQ10-ω-diazirine (23a)

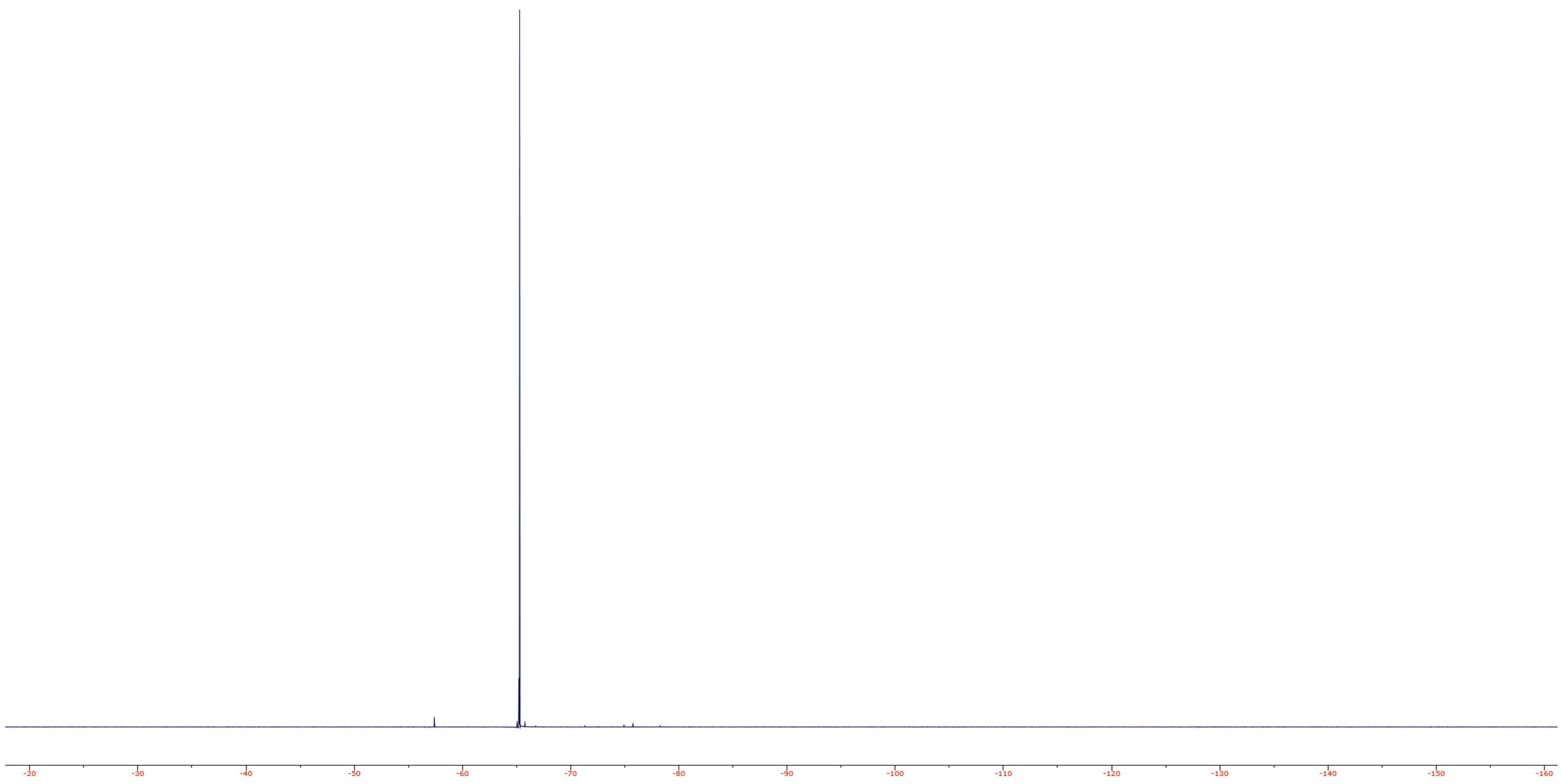

^{1}H NMR (400 MHZ, $CDCl_3$) for CoQ10-ω-alkyne (19)

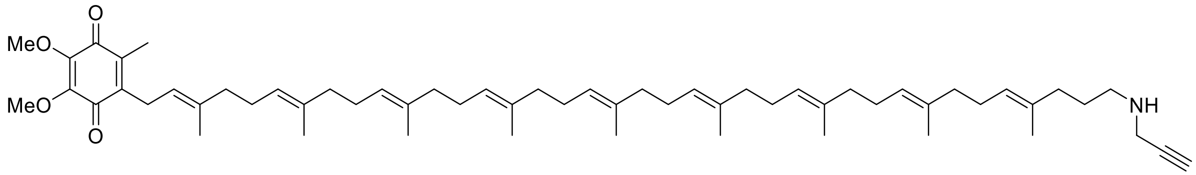

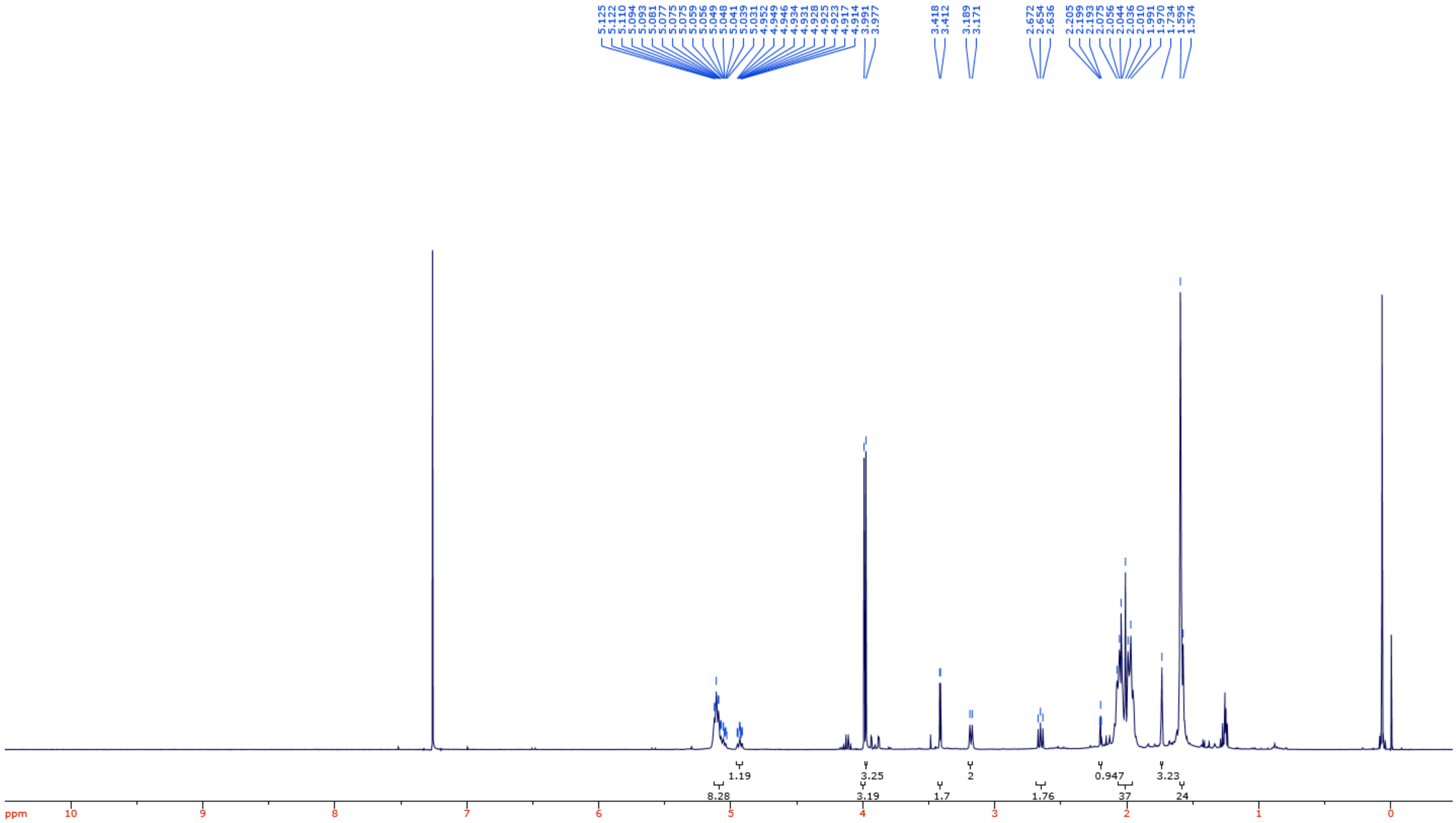

^{13}C NMR (101 MHZ, $CDCl_3$) for CoQ10-ω-alkyne (19)

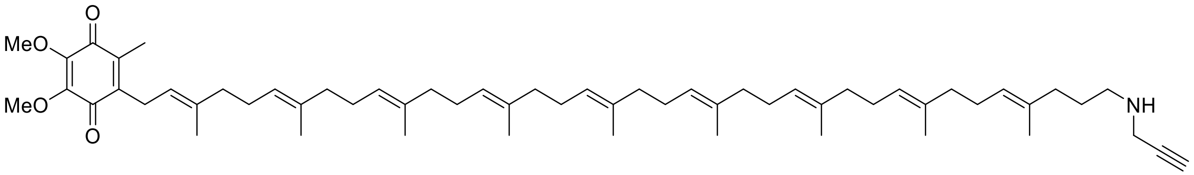

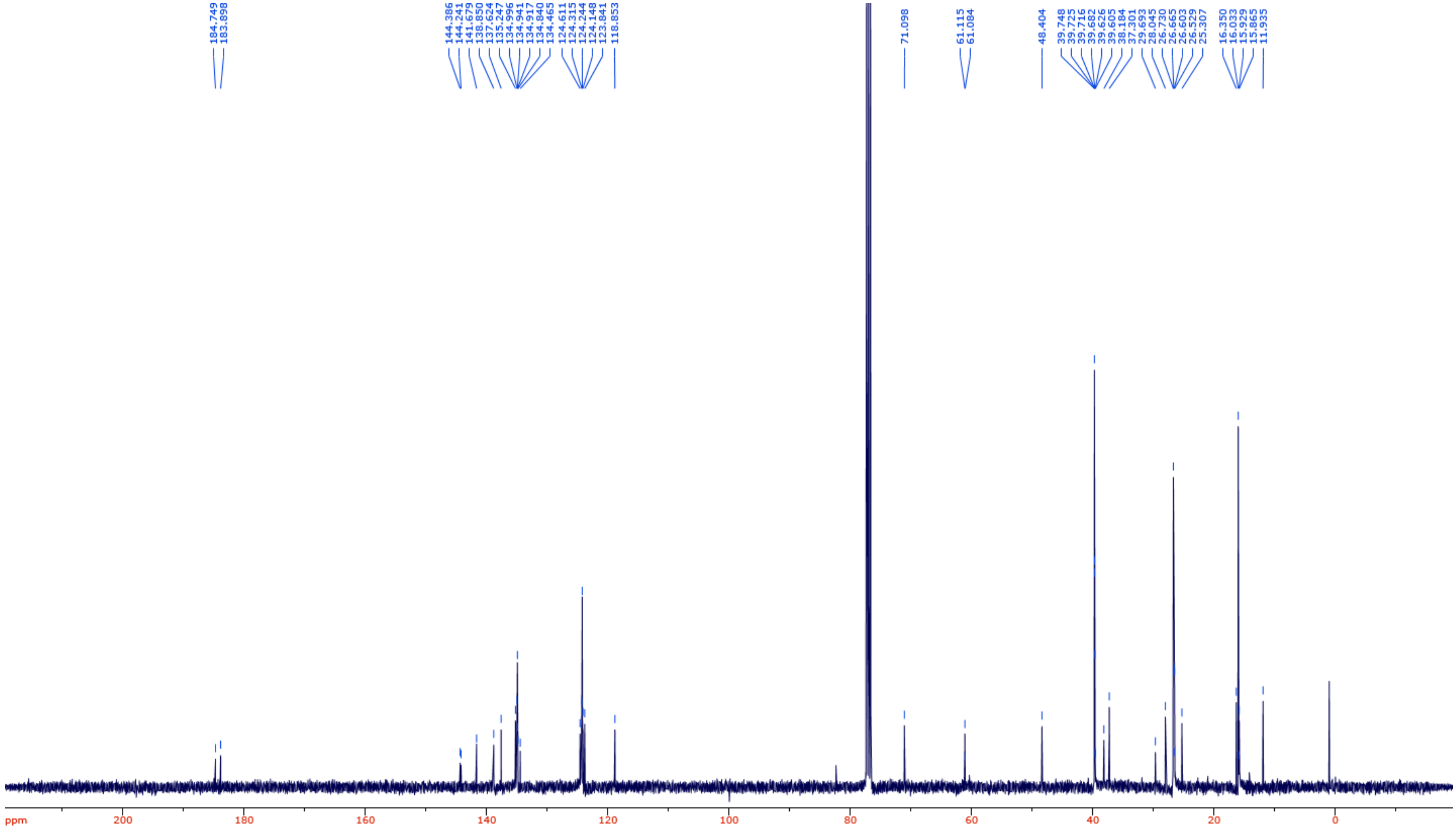

2D COSY (400 MHZ, $CDCl_3$) for CoQ10-ω-alkyne (19)

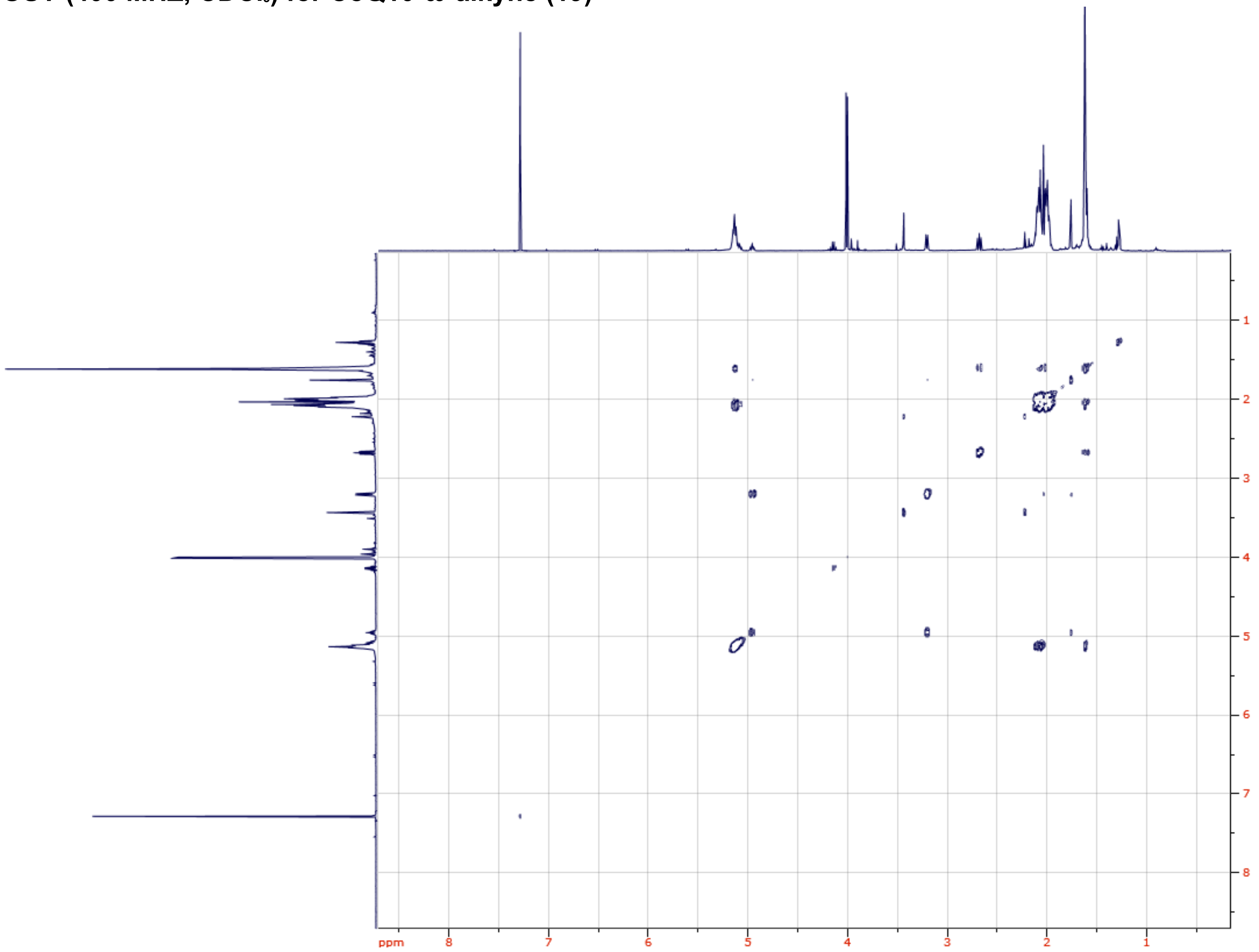

2D HSQC (400 MHZ, $CDCl_3$) for CoQ10-ω-alkyne (19)

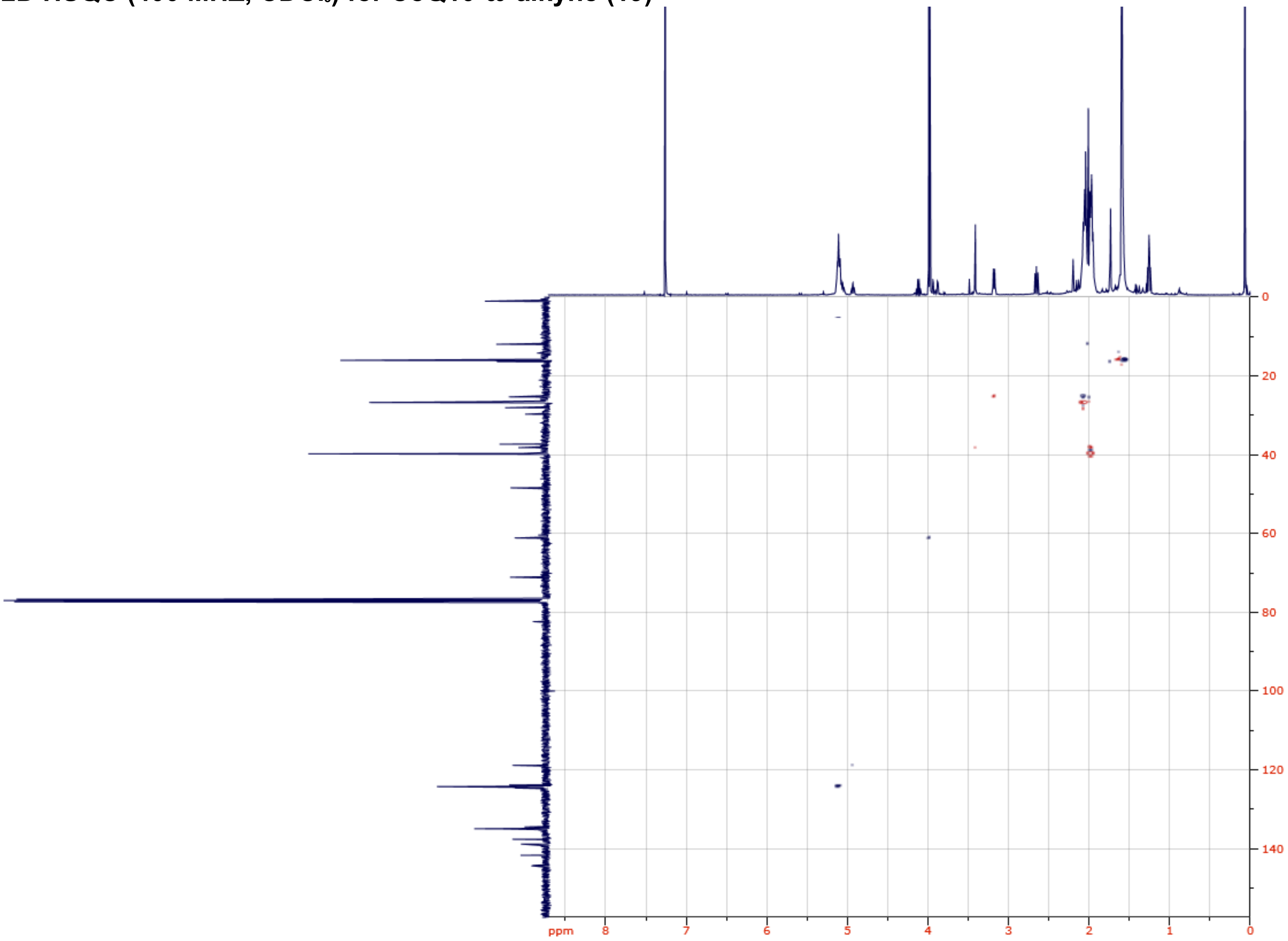

2D HMBC (400 MHZ, $CDCl_3$) for CoQ10-ω-alkyne (19)

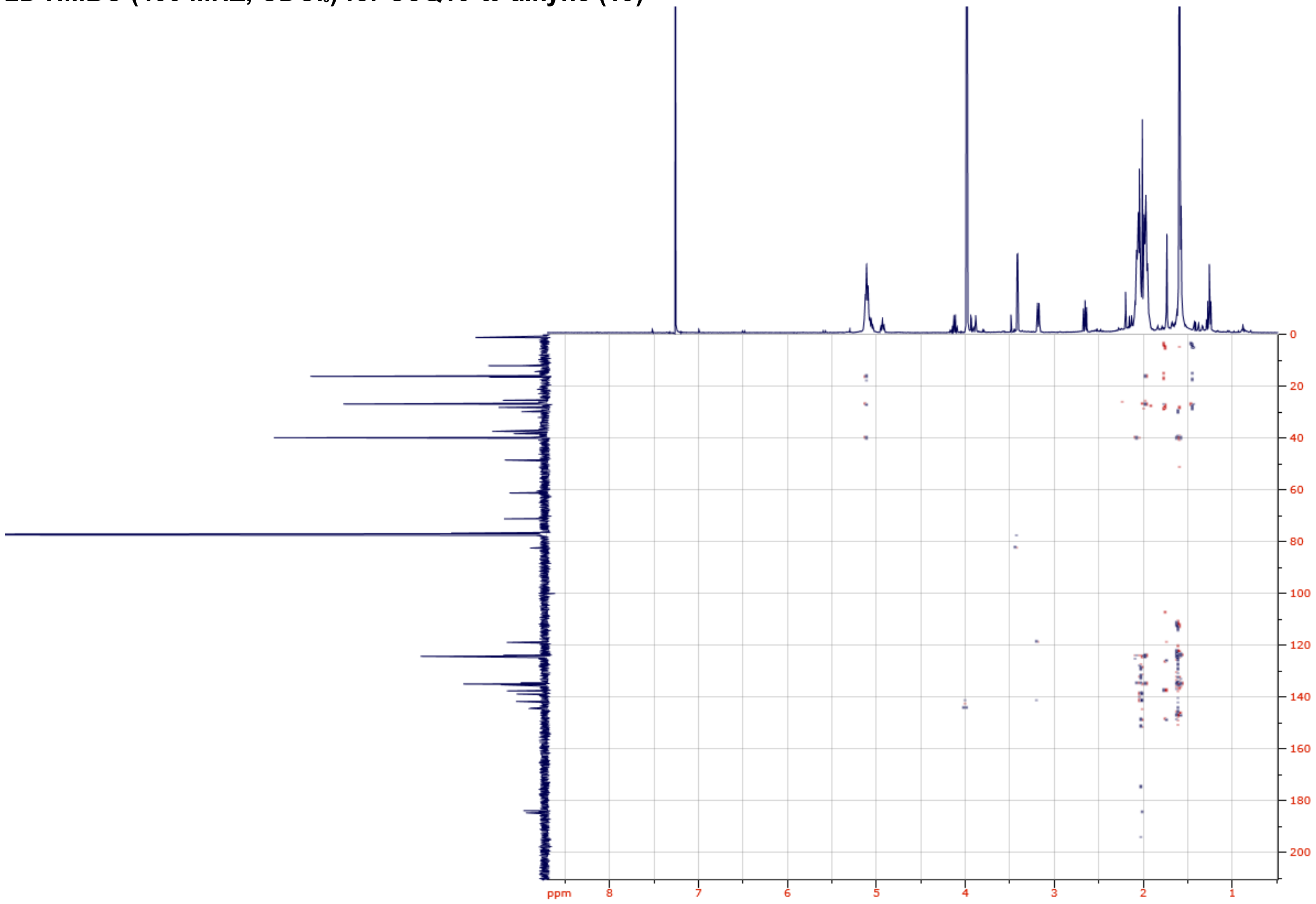

^{1}H NMR (400 MHZ, $CDCl_3$) for CoQ10-ω-CF_3 (20)

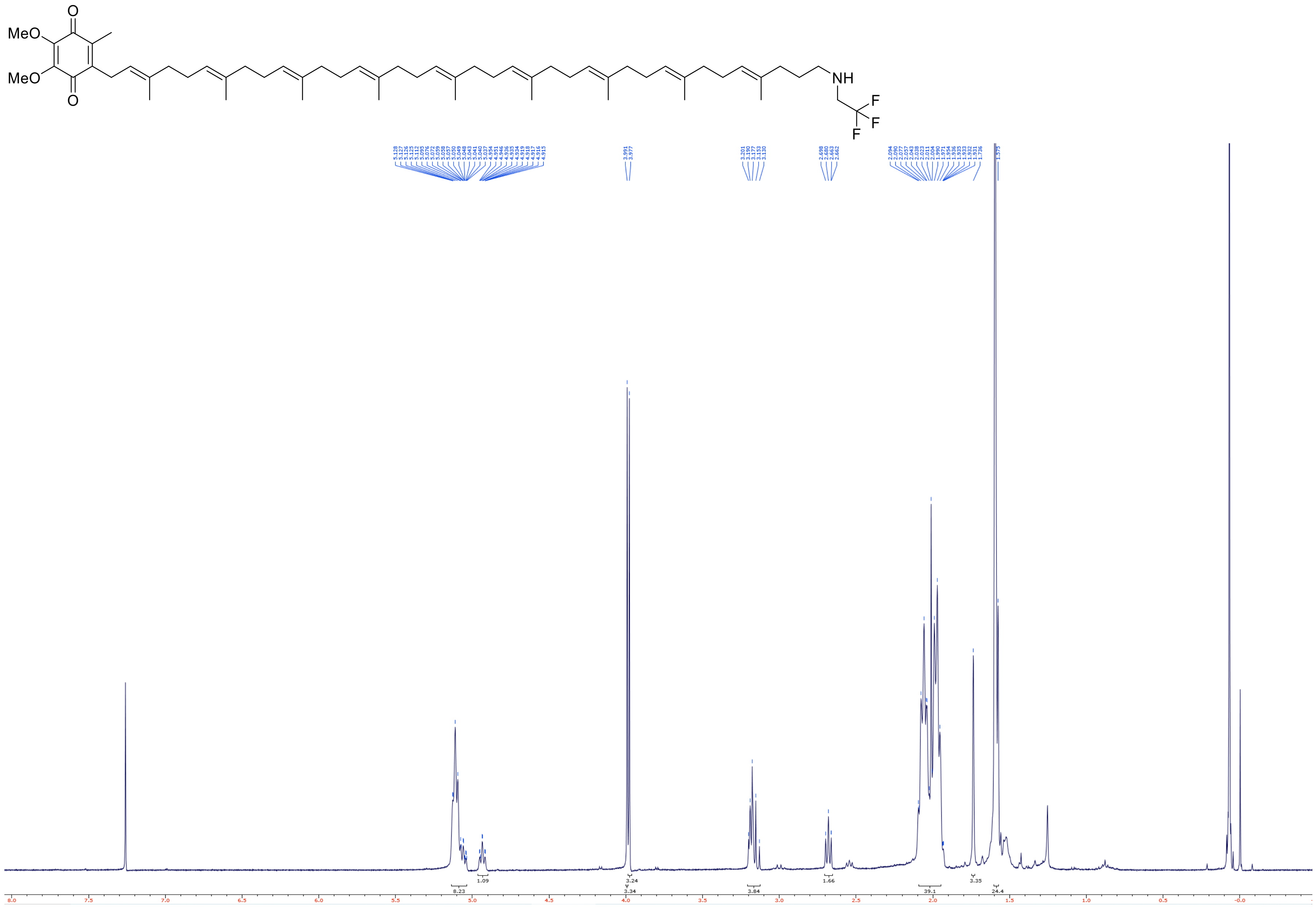

^{13}C NMR (101 MHZ, $CDCl_3$) for CoQ10-ω-CF_3 (20)

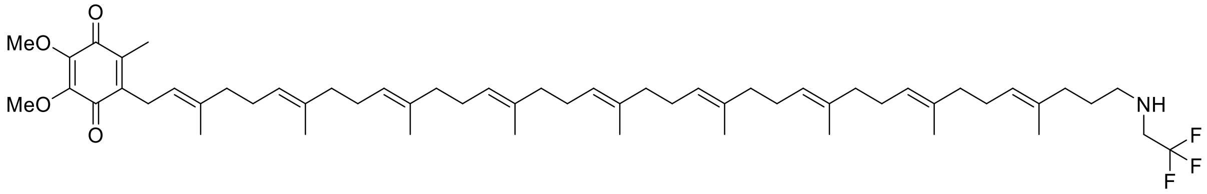

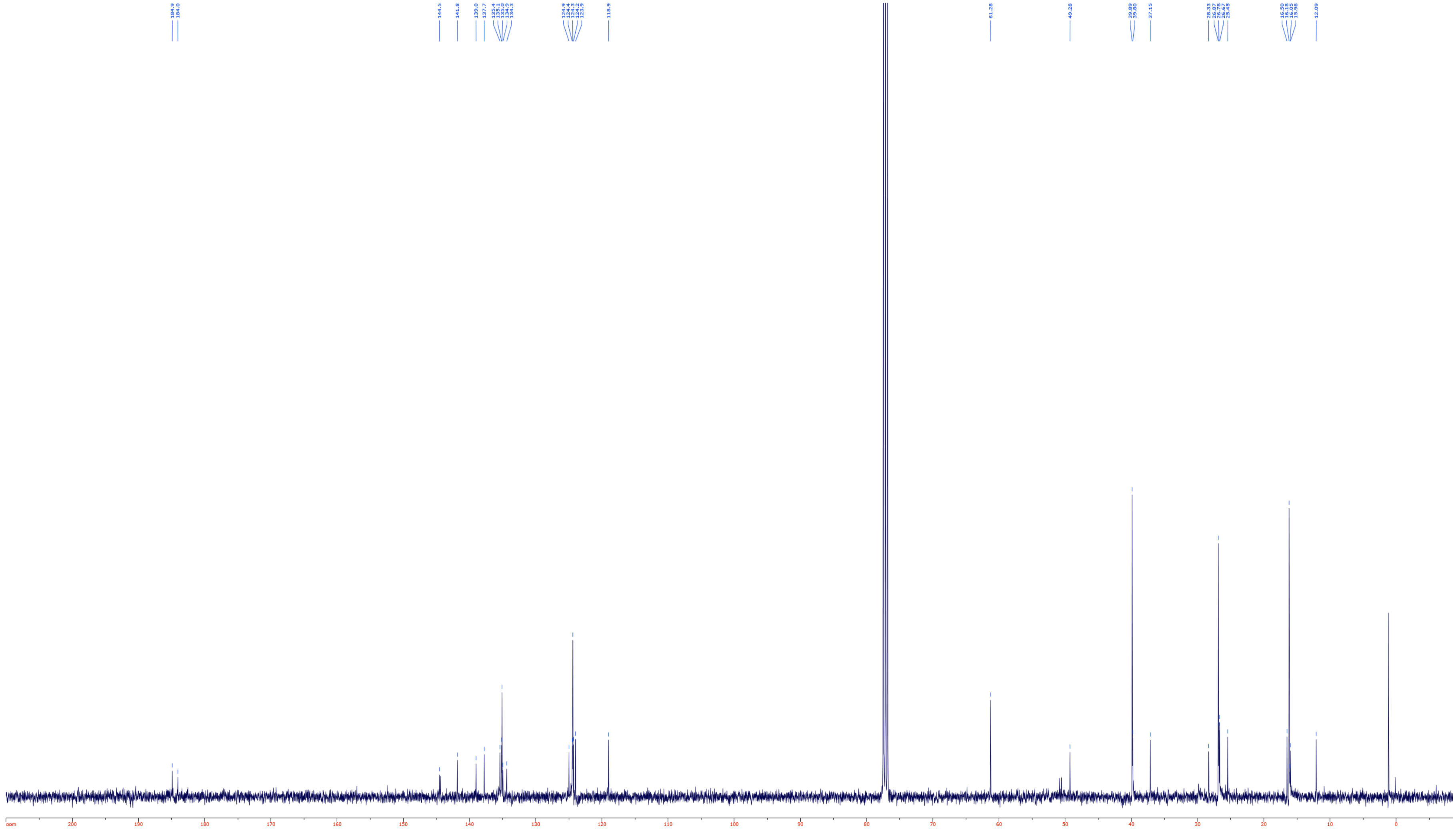

2D COSY (400 MHZ, $CDCl_3$) for CoQ10-ω-CF_3 (20)

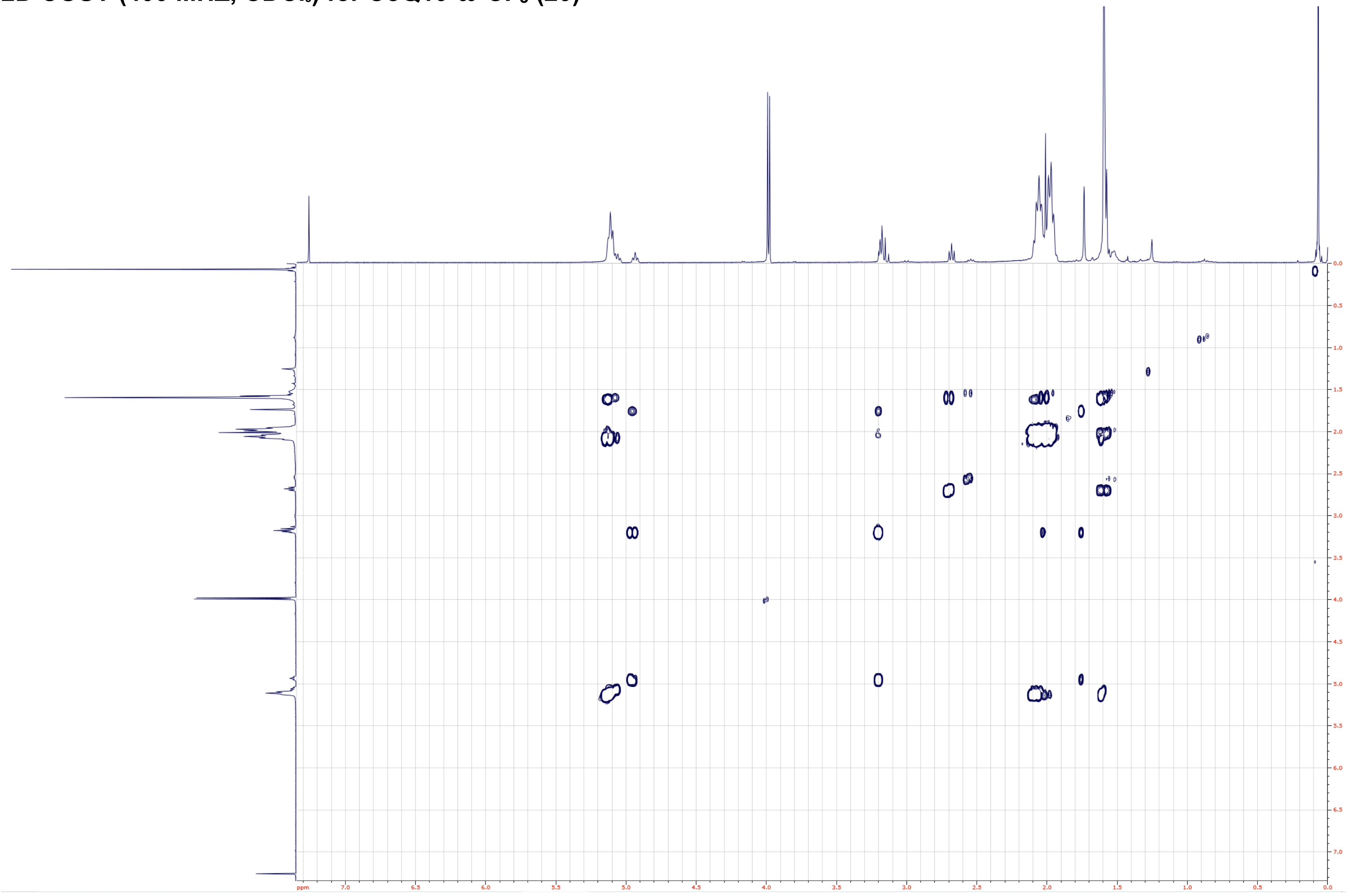

2D HSQC (400 MHZ, $CDCl_3$) for CoQ10-ω-CF_3 (20)

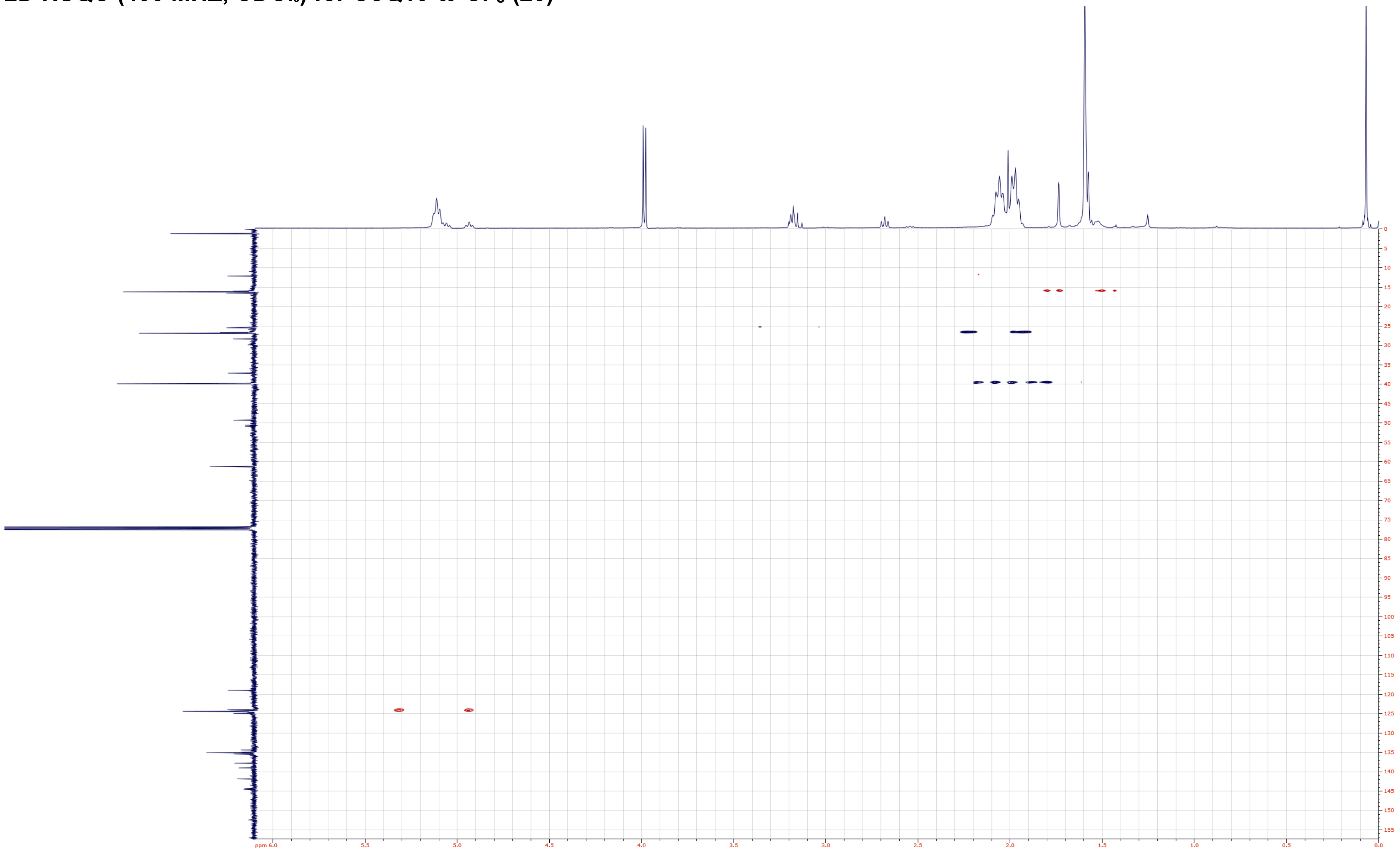

2D HMBC (400 MHZ, $CDCl_3$) for CoQ10-ω-CF_3 (20)

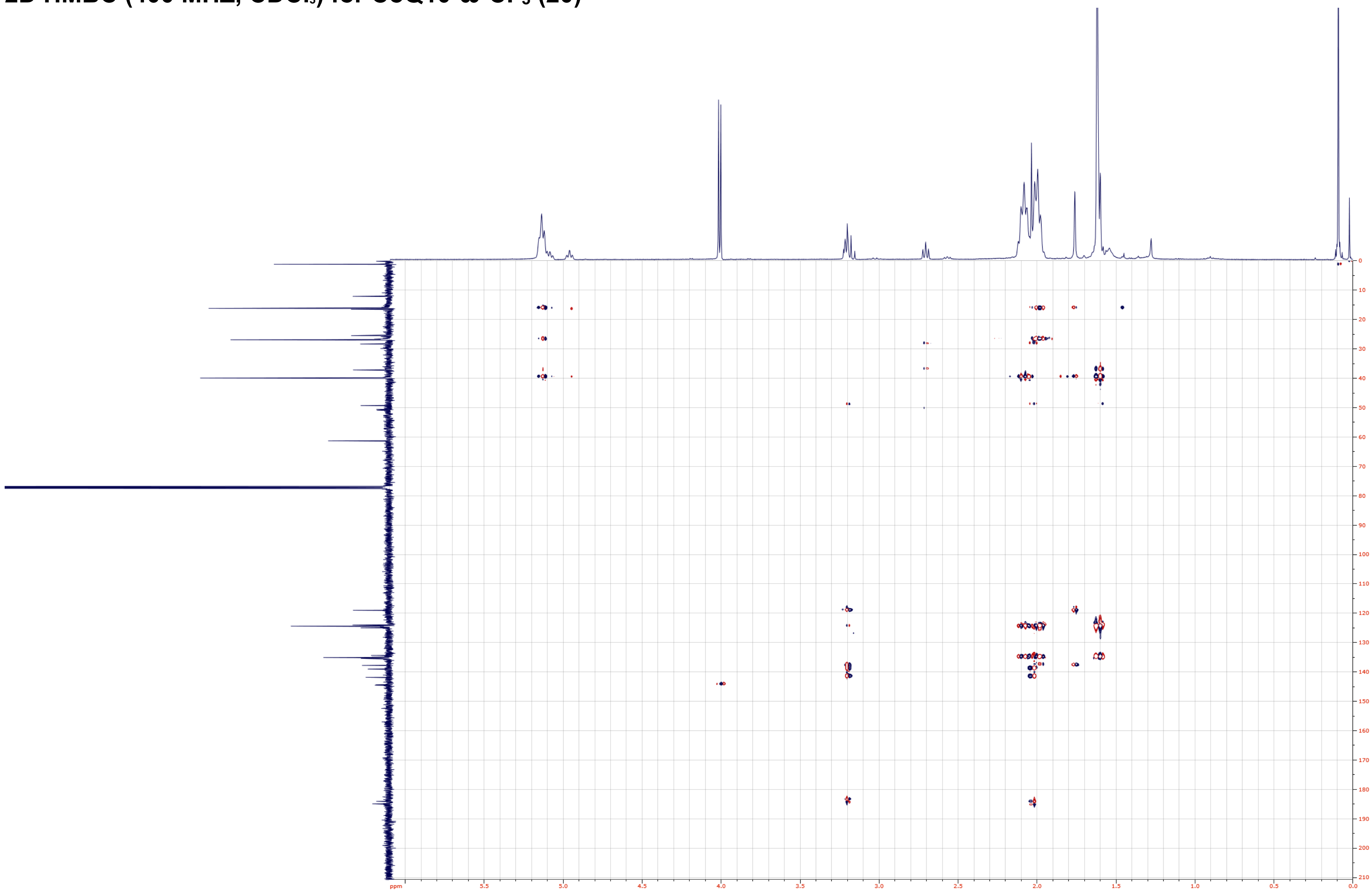

^{19}F NMR (376 MHZ, $CDCl_3$) for CoQ10-ω-CF_3 (20)

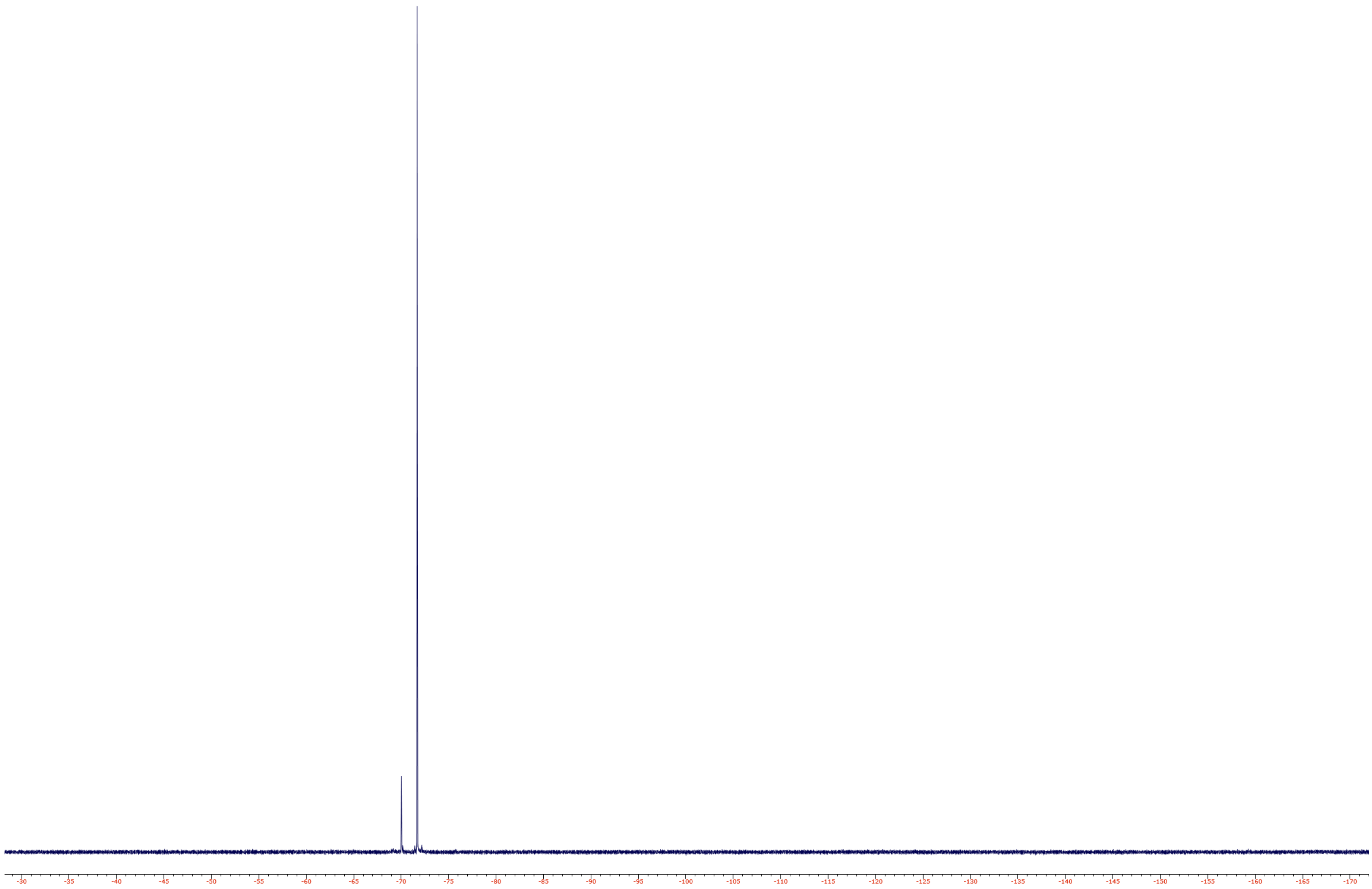

^{1}H NMR (400 MHZ, $CDCl_3$) for CoQ10-ω-pyrene (21)

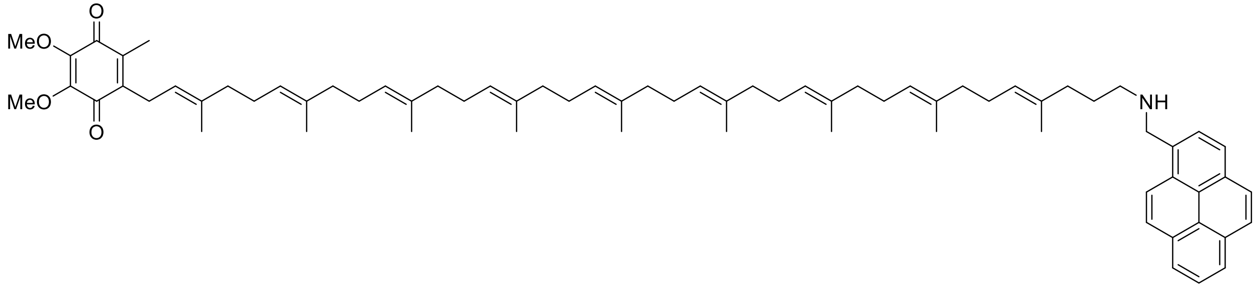

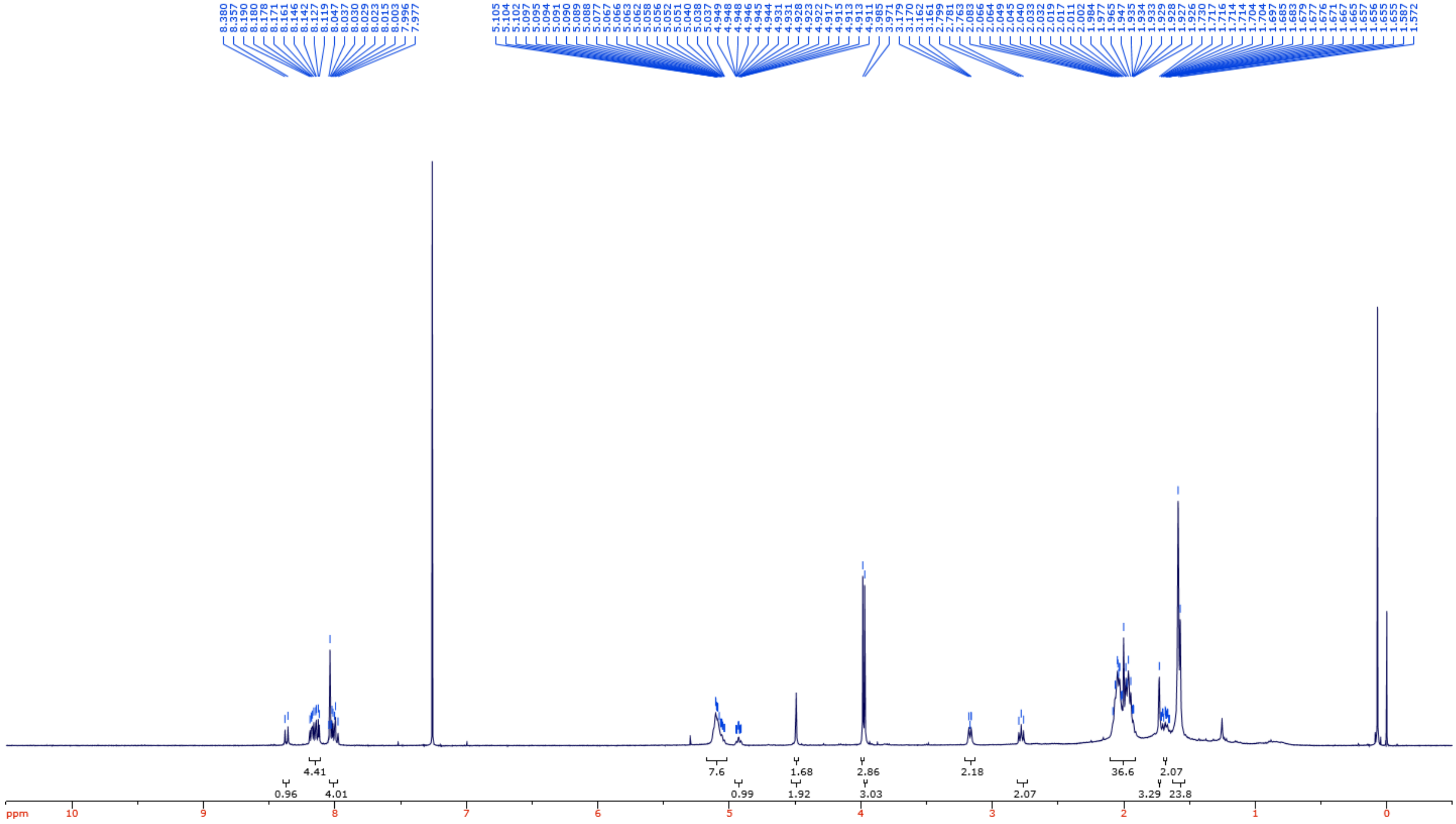

^{13}C NMR (101 MHZ, $CDCl_3$) for CoQ10-ω-pyrene (21)

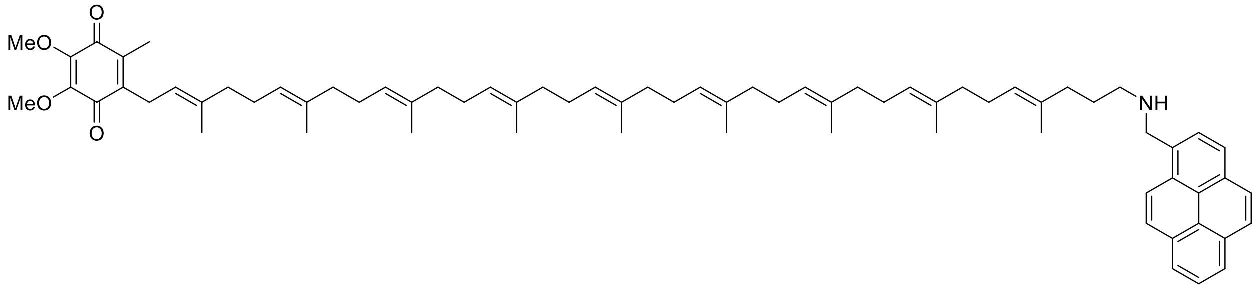

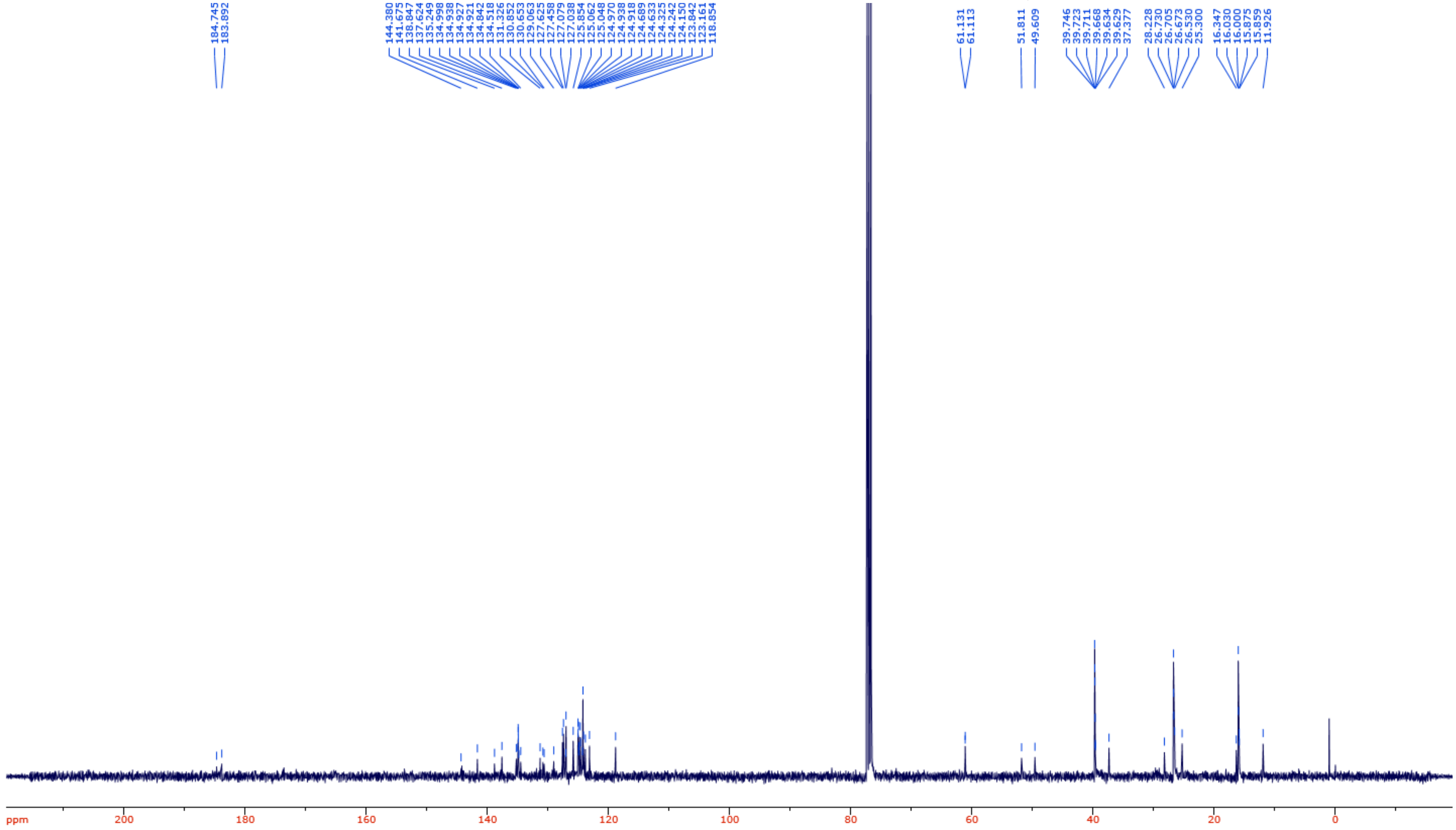

2D COSY (400 MHZ, $CDCl_3$) for CoQ10-ω-pyrene (21)

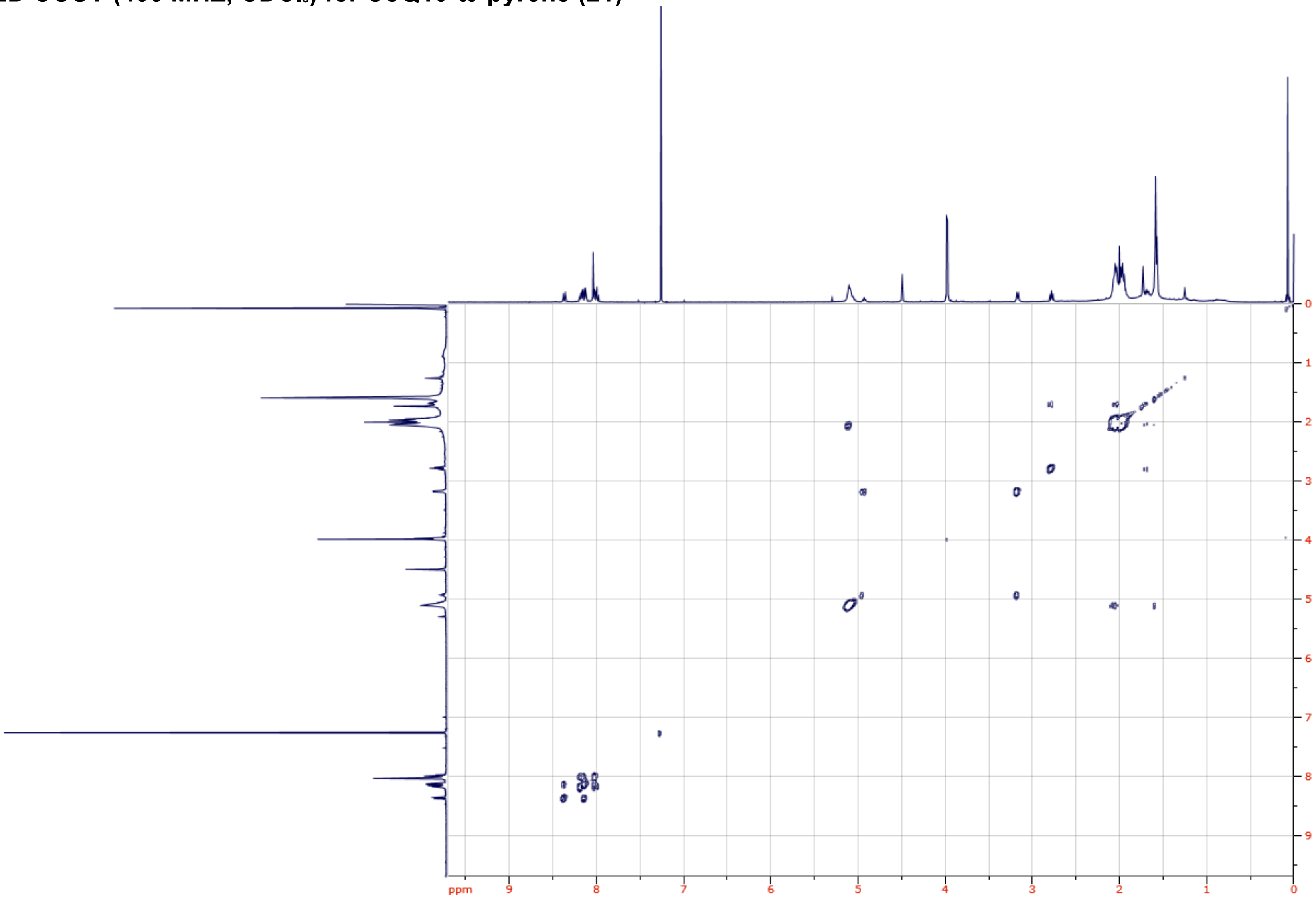

2D HSQC (400 MHZ, CDCl$_3$) for CoQ10-ω-pyrene (21)

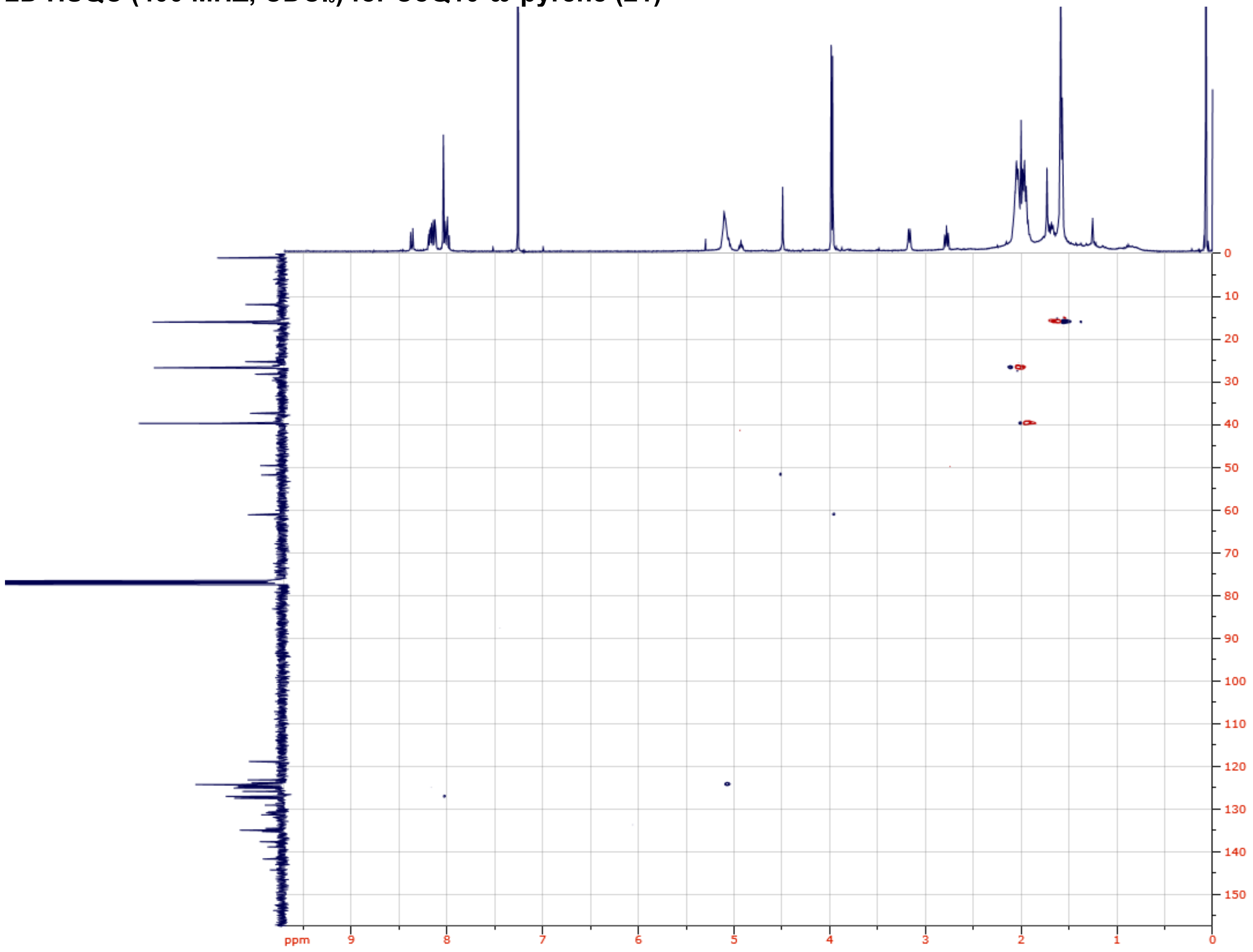

2D HMBC (400 MHZ, $CDCl_3$) for CoQ10-ω-pyrene (21)

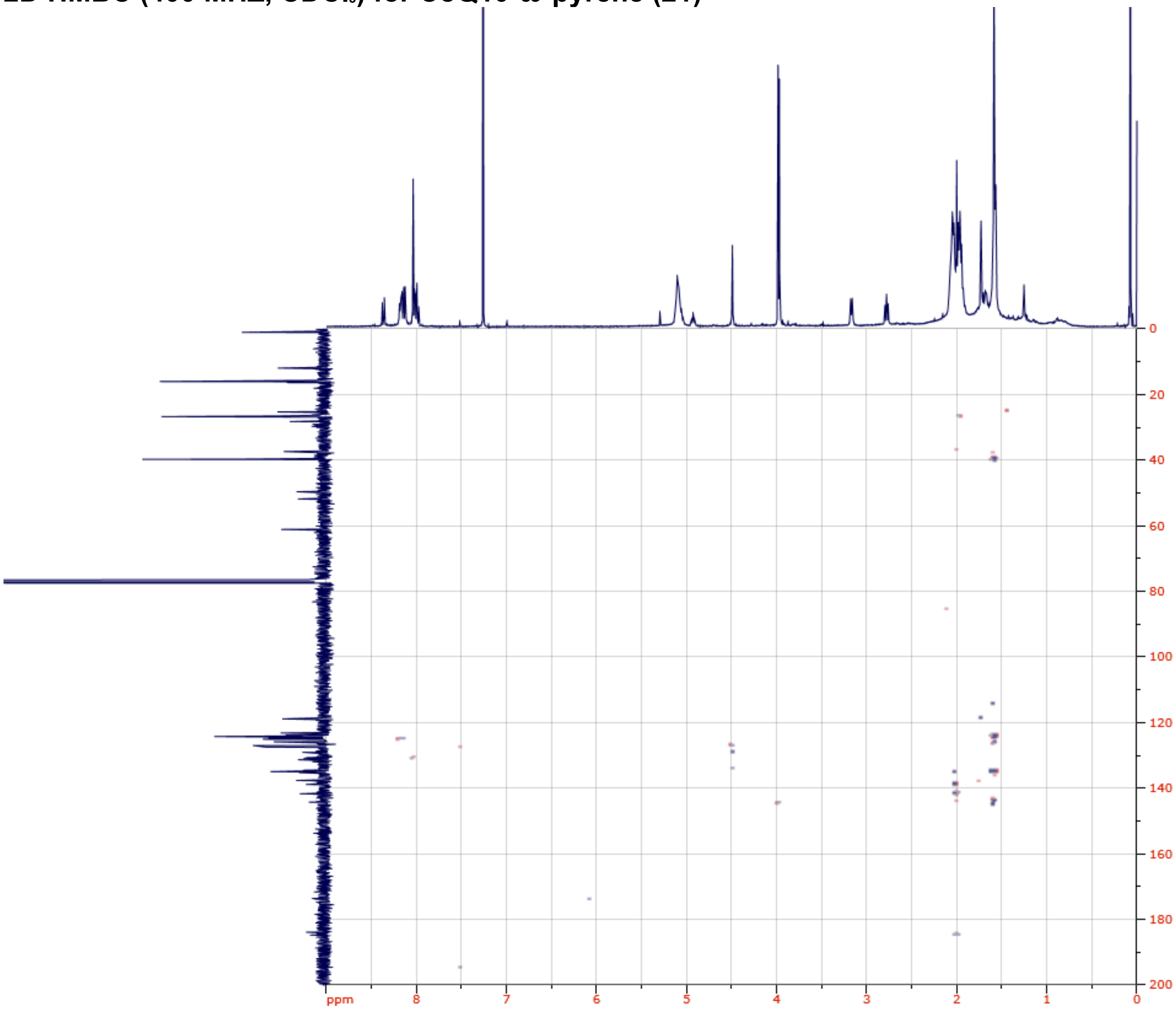

^{1}H NMR (400 MHZ, $CDCl_3$) for CoQ10-ω-PNB (22)

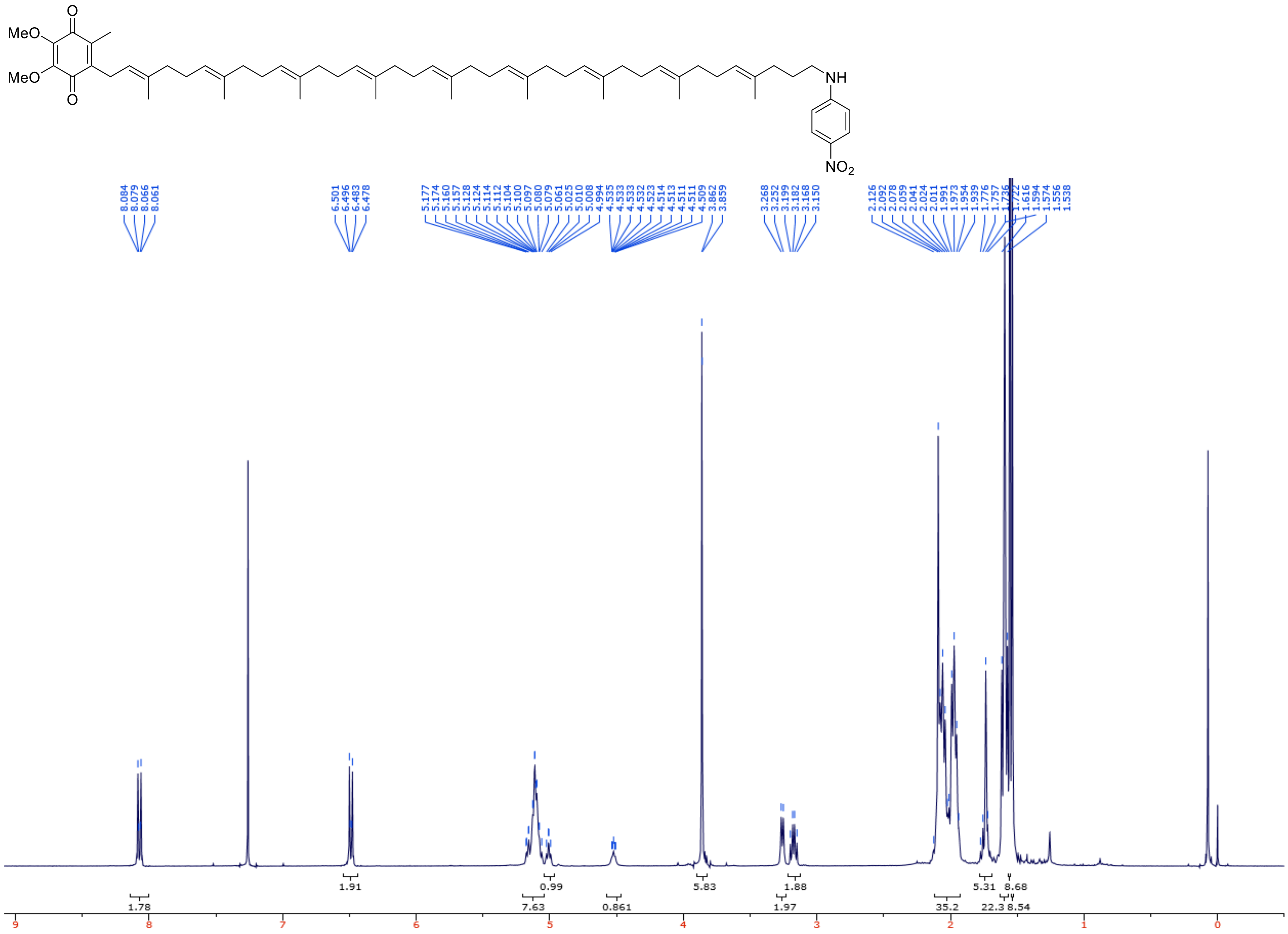

^{13}C NMR (400 MHZ, $CDCl_3$) for CoQ10-ω-PNB (22)

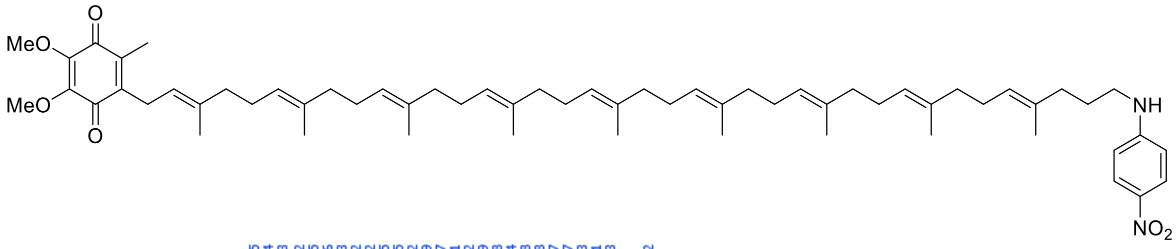

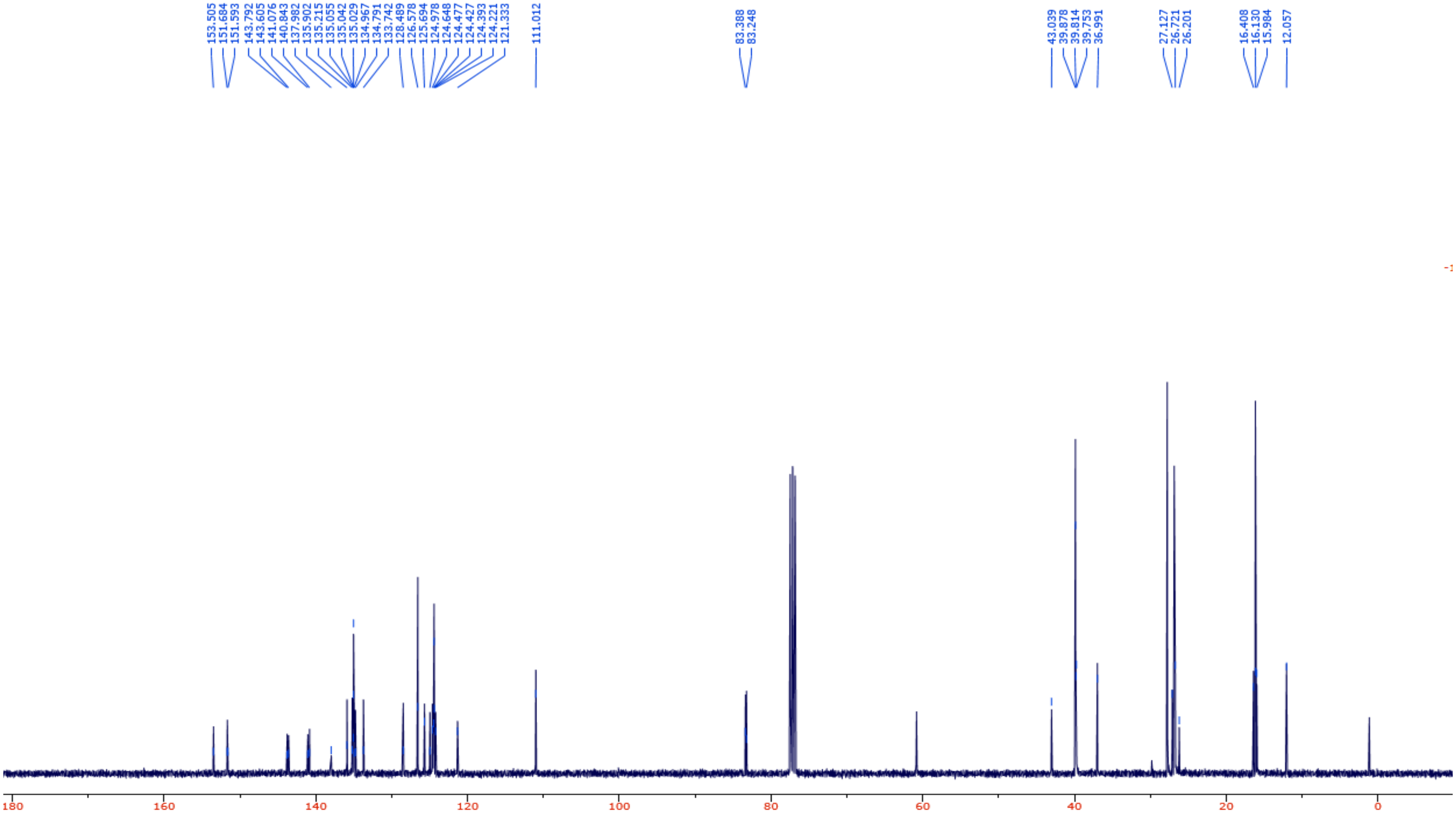

2D COSY (400 MHZ, $CDCl_3$) for CoQ10-ω-PNB (22)

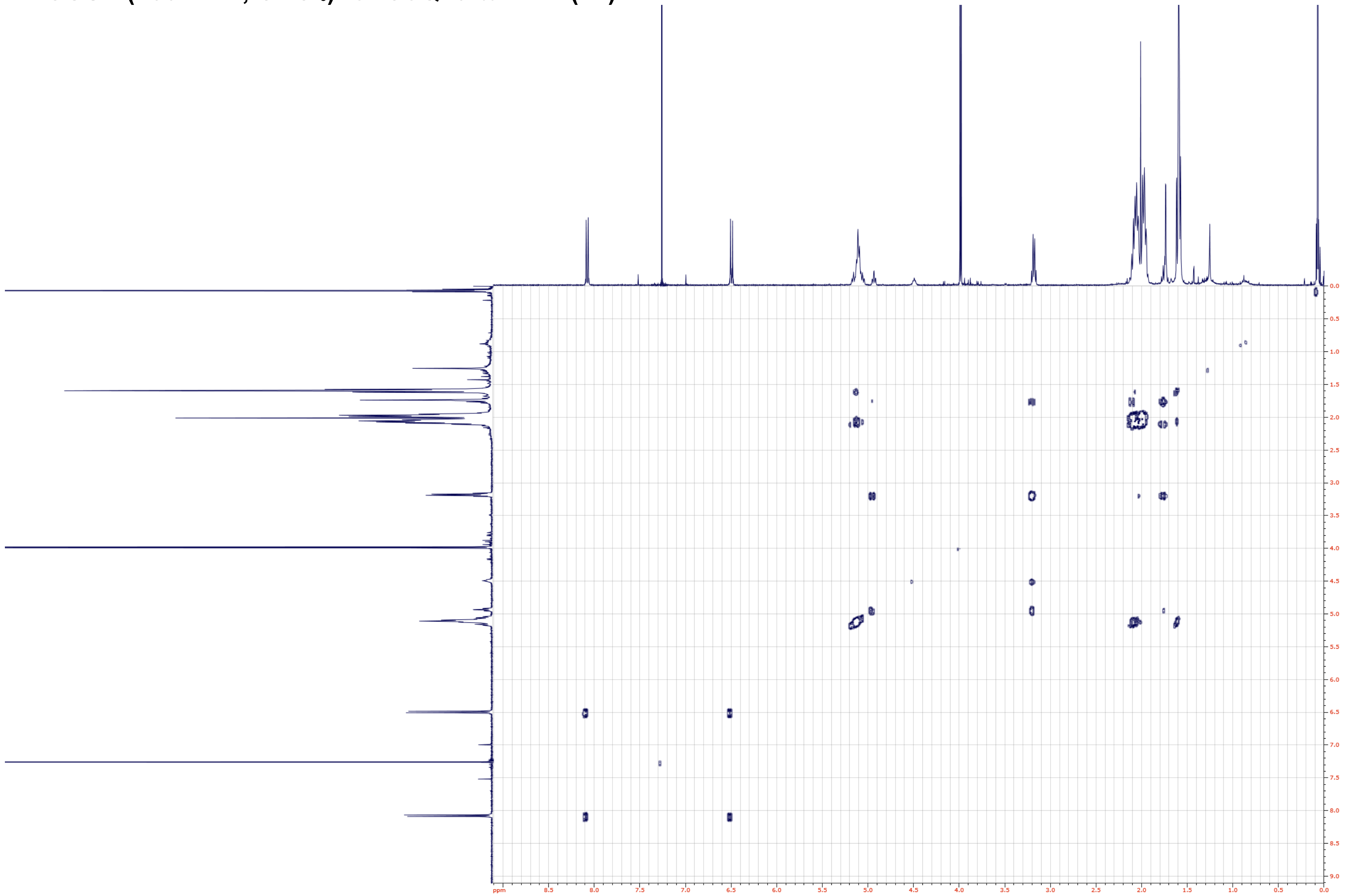

2D HSQC (400 MHZ, $CDCl_3$) for CoQ10-ω-PNB (22)

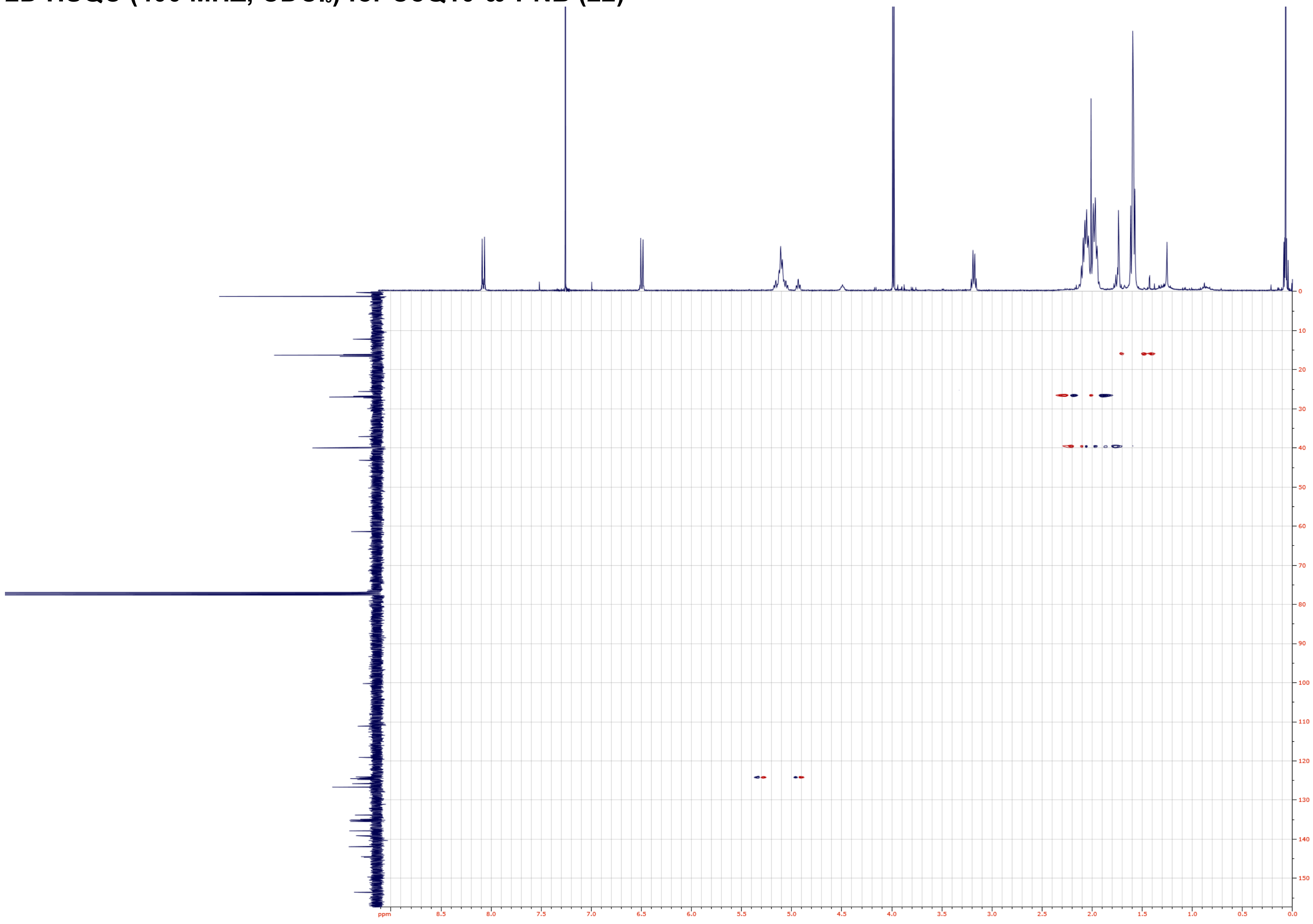

2D HMBC (400 MHZ, CDCl$_3$) for CoQ10-ω-PNB (22)

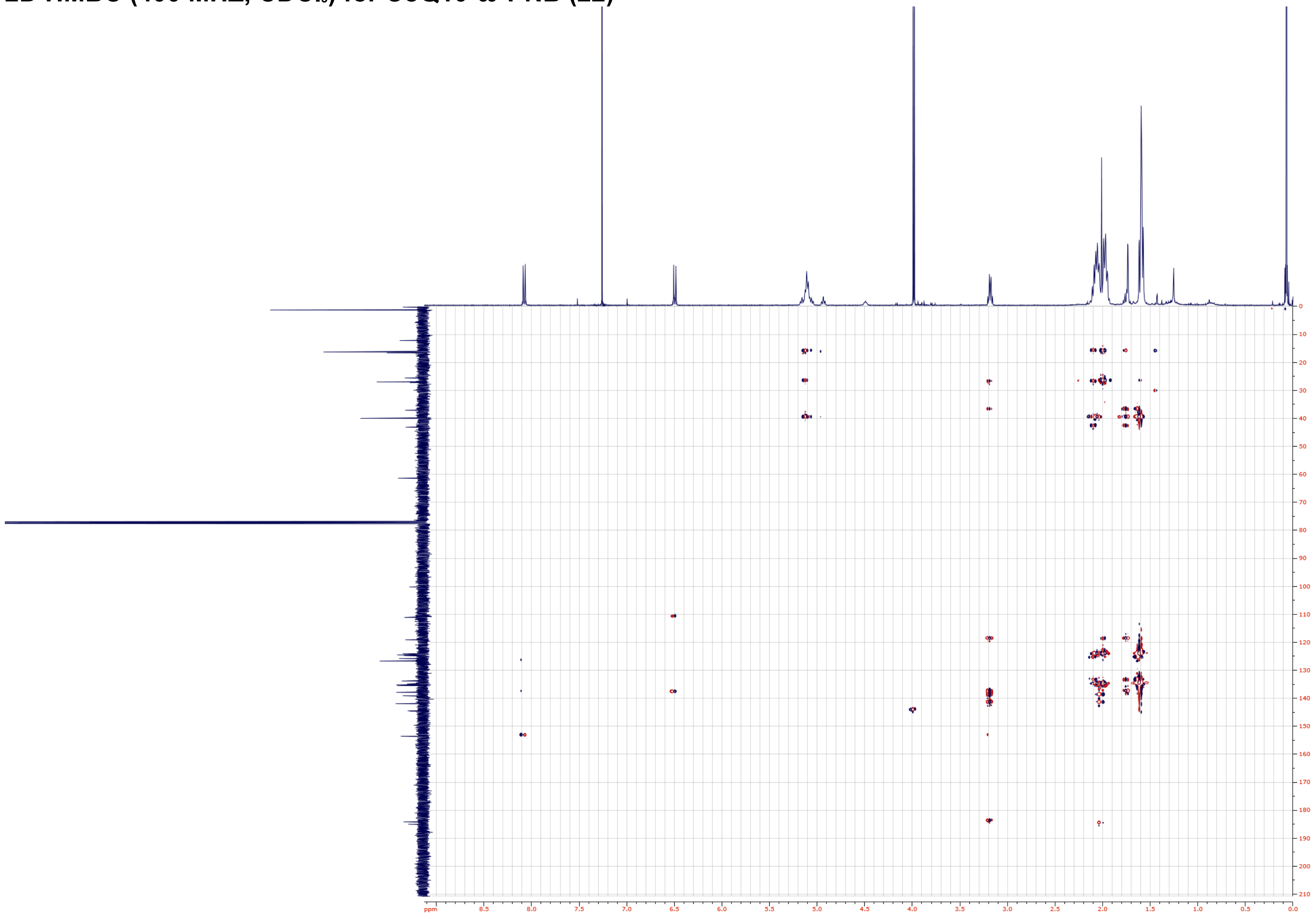

^{1}H NMR (400 MHZ, $CDCl_3$) for CoQ10-ω-diazirine (23)

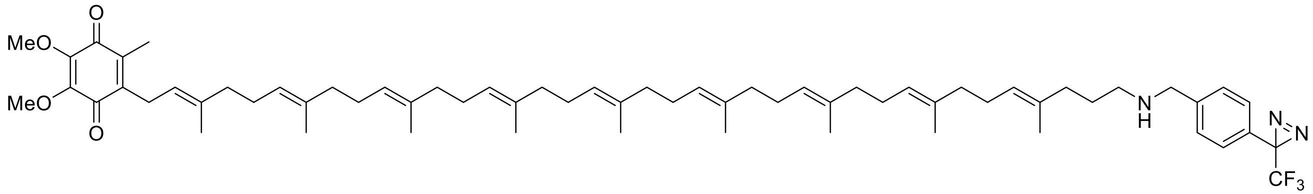

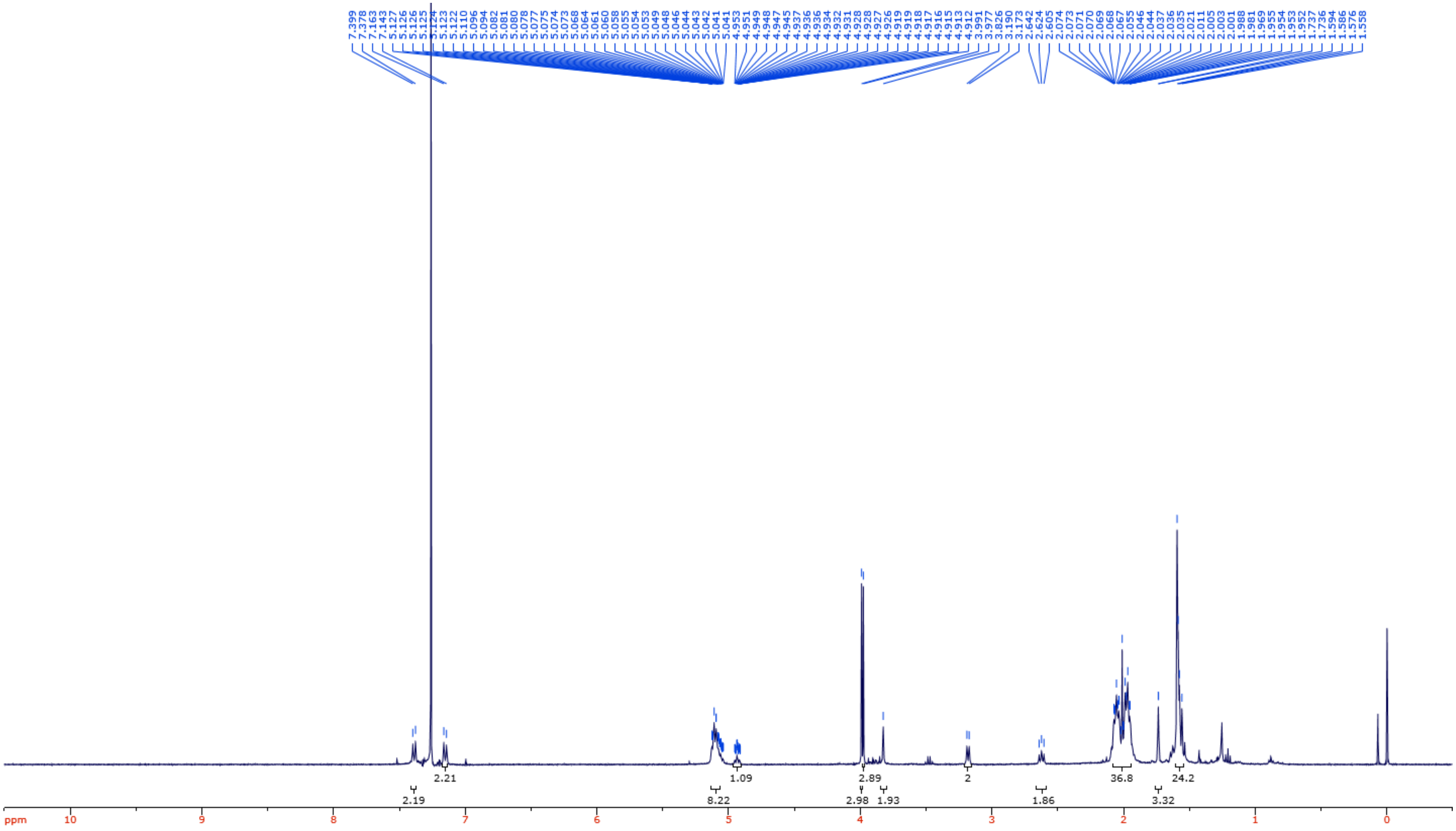

^{13}C NMR (101 MHZ, $CDCl_3$) for CoQ10-ω-diazirine (23)

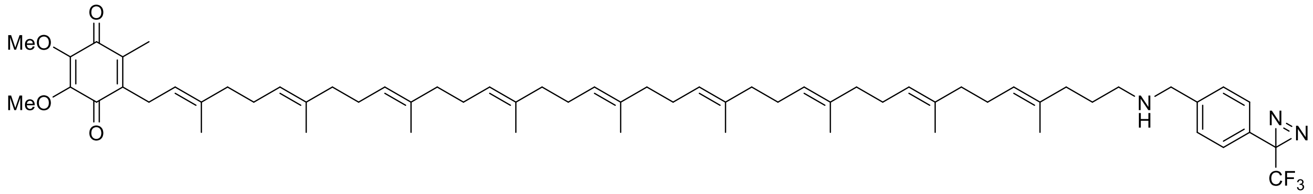

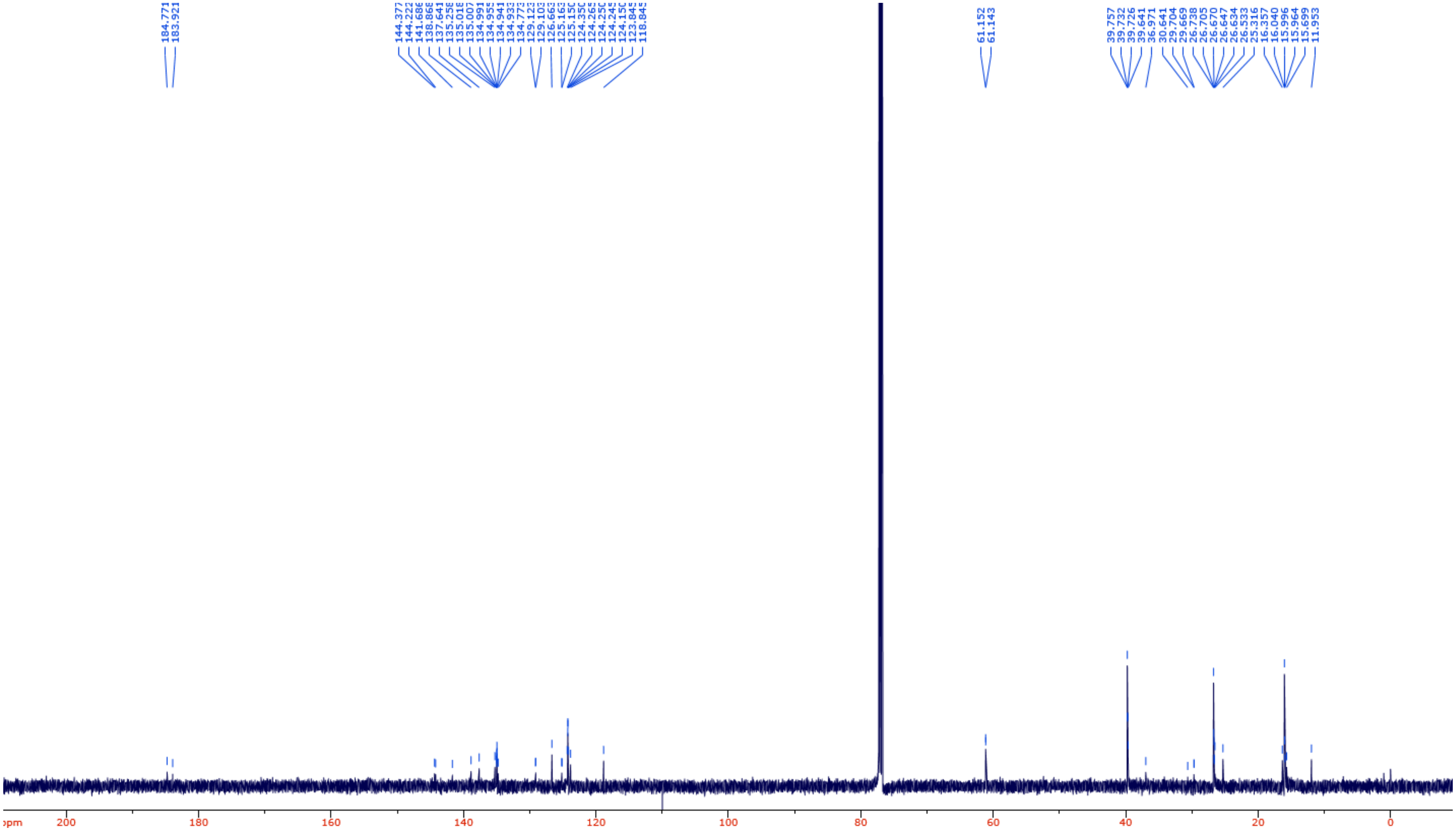

2D COSY (400 MHZ, $CDCl_3$) for CoQ10-ω-diazirine (23)

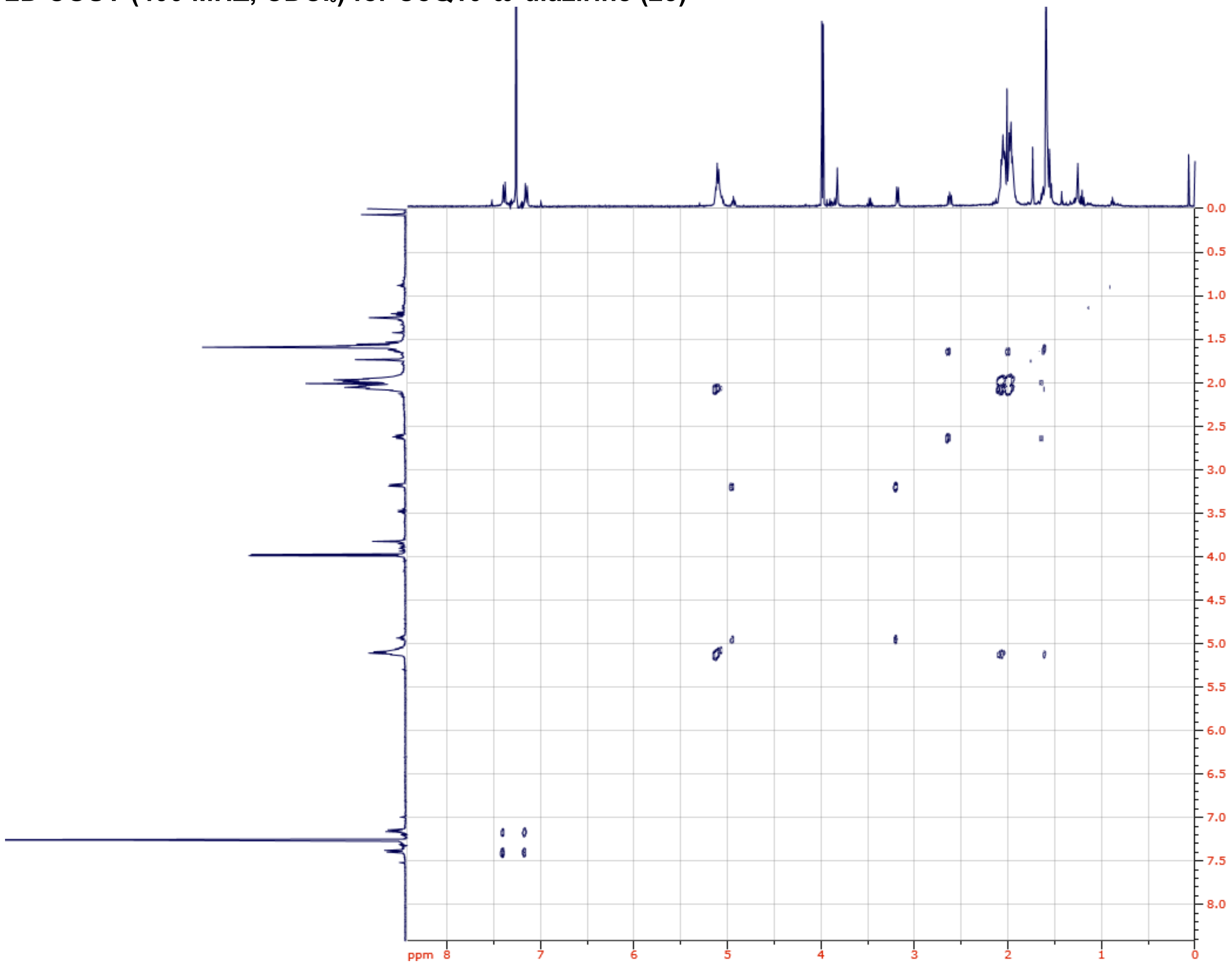

2D HSQC (400 MHZ, $CDCl_3$) for CoQ10-ω-diazirine (23)

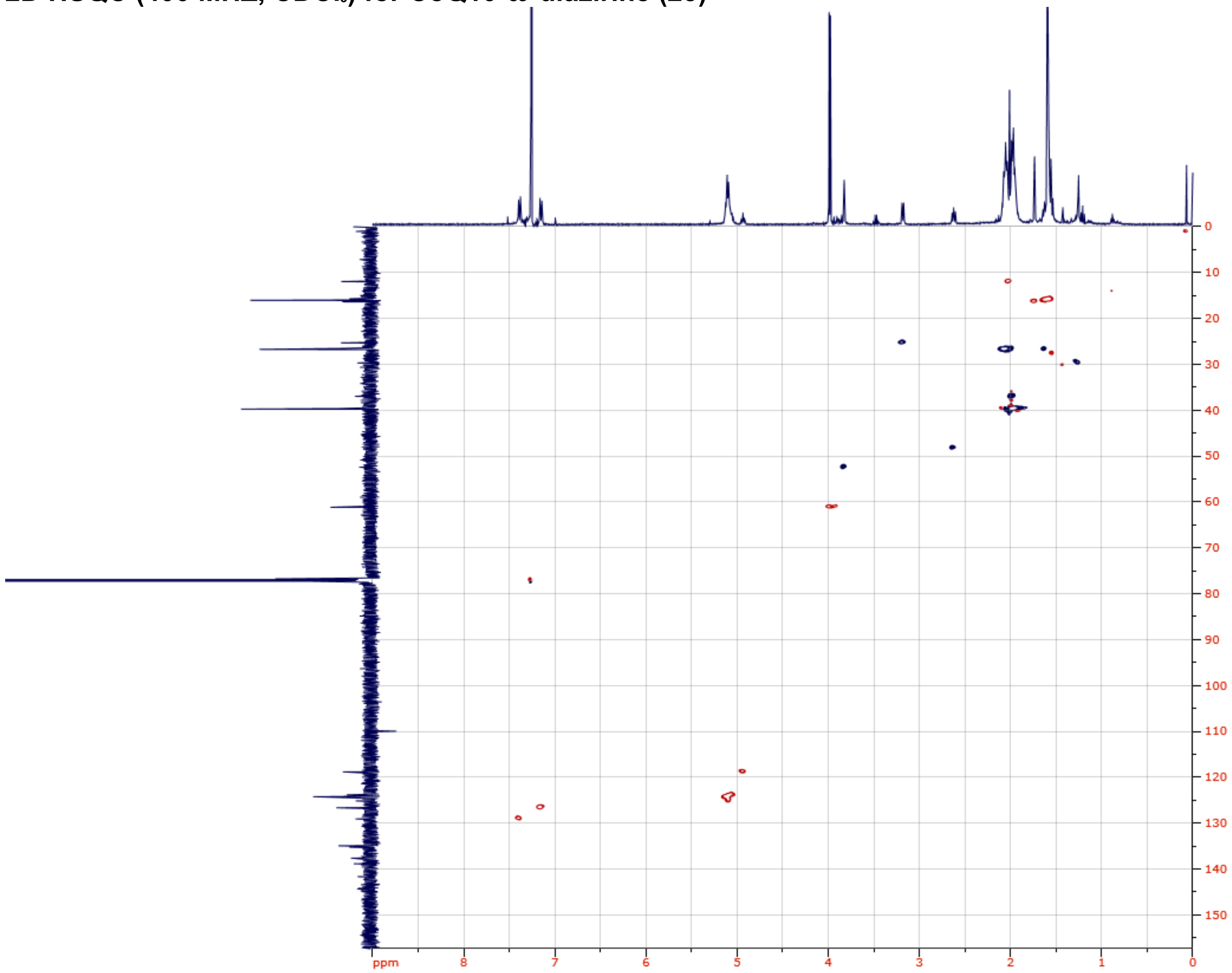

2D HMBC (400 MHZ, $CDCl_3$) for CoQ10-ω-diazirine (23)

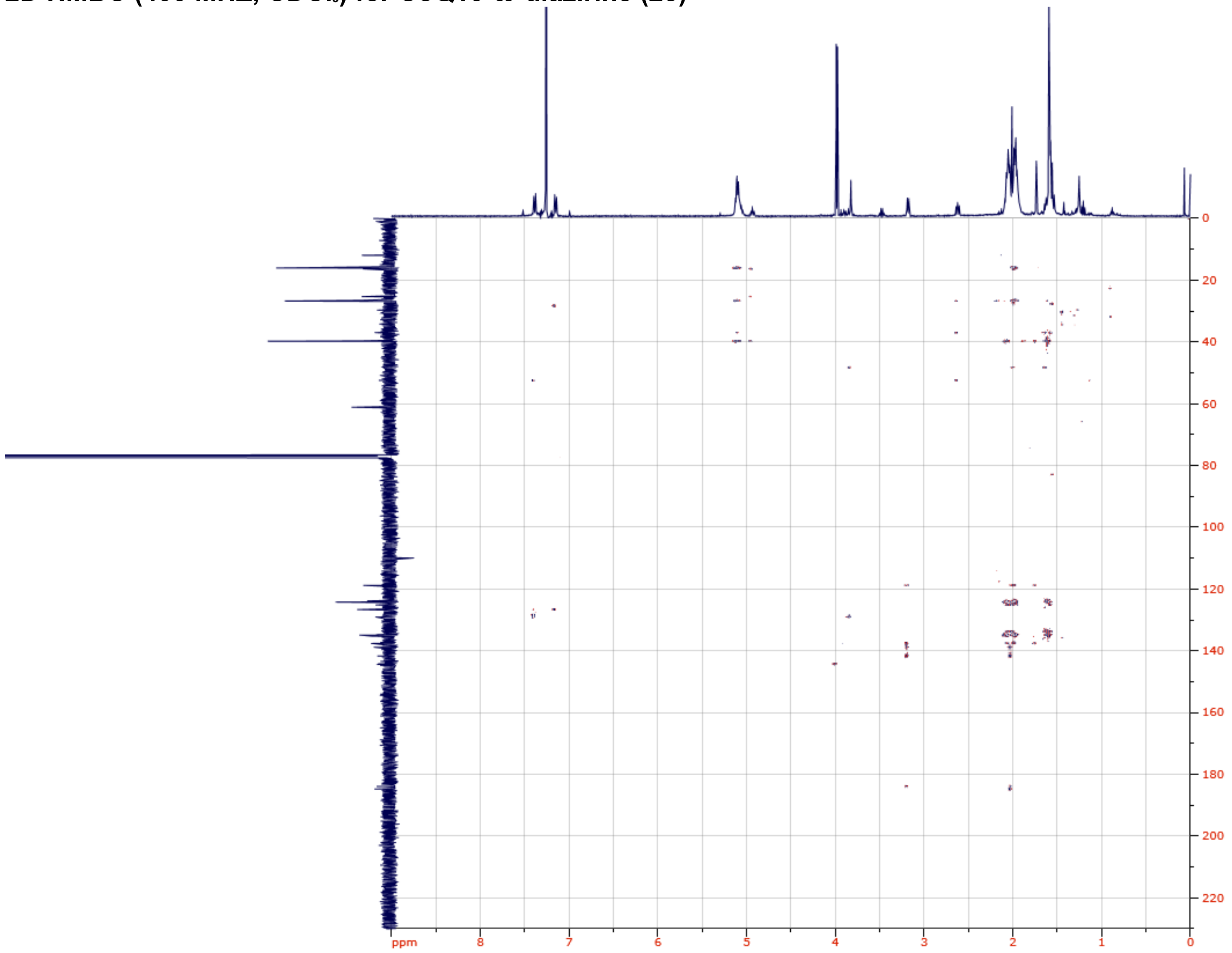

^{19}F-NMR (565 MHz, $CDCl_3$) for CoQ10-ω-diazirine (23)

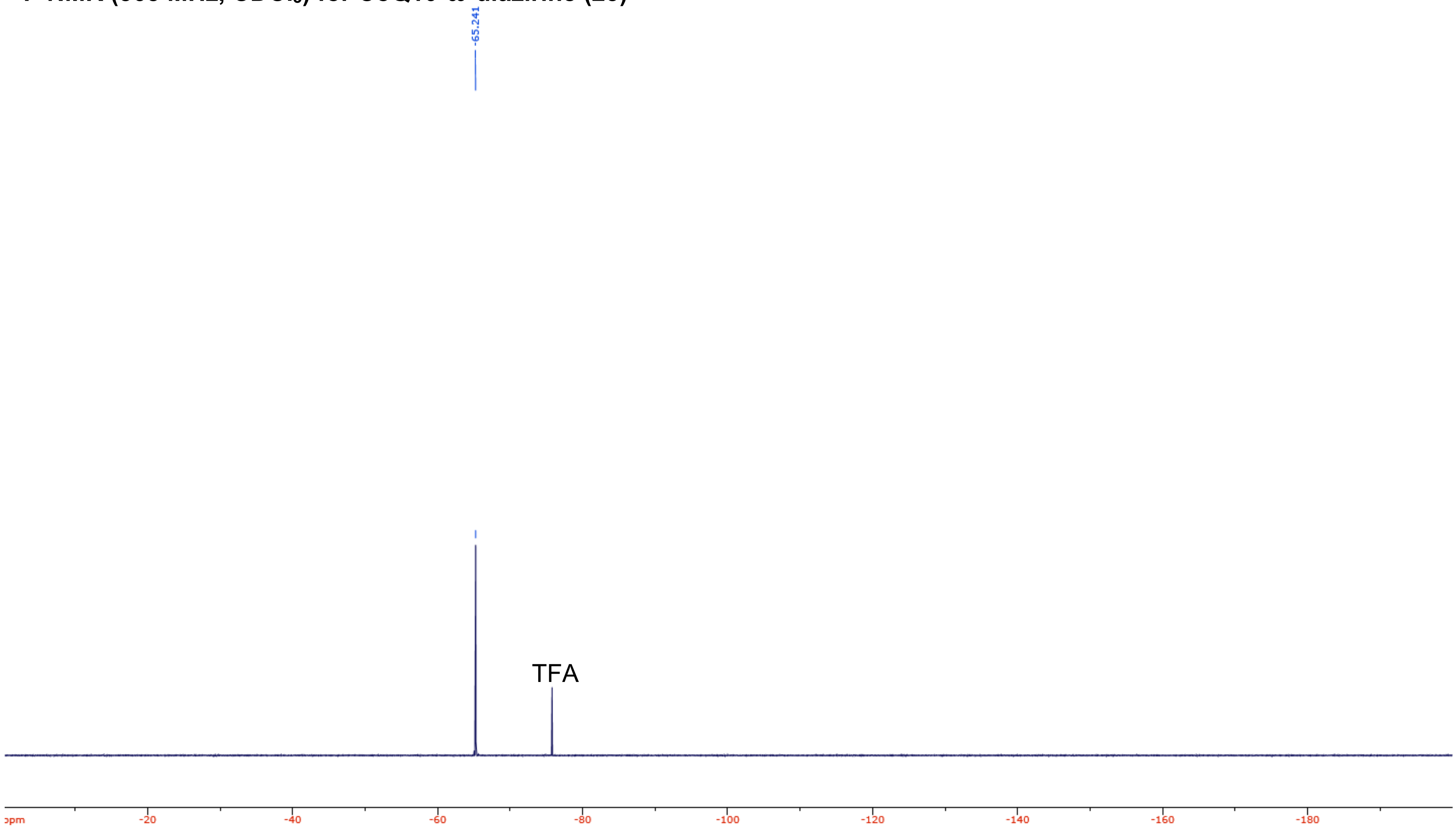

^{1}H NMR (400 MHZ, $CDCl_3$) for Boc_2-CoQ10-ω-OTs (24)

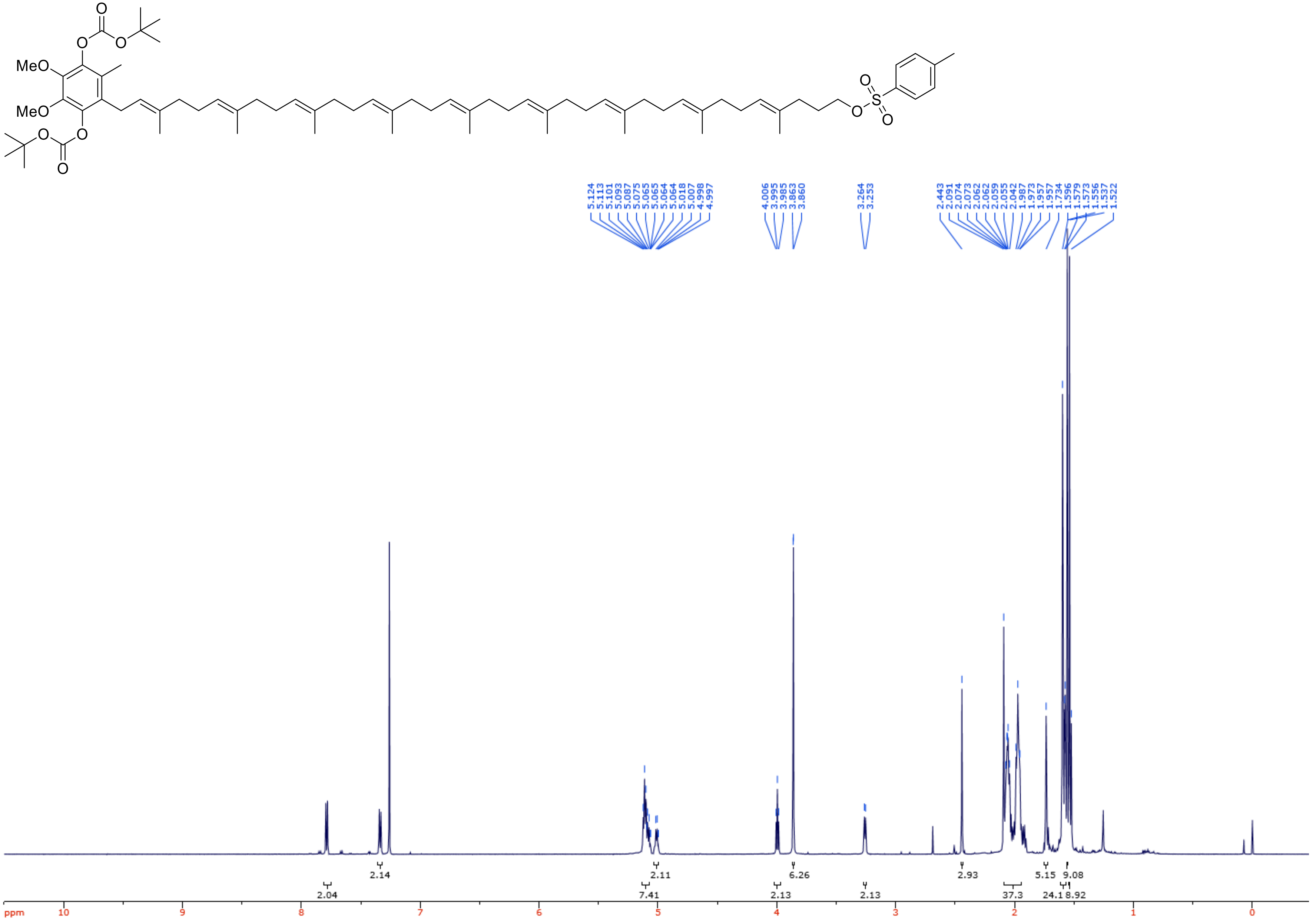

^{13}C NMR (101 MHZ, $CDCl_3$) for Boc$_2$-CoQ10-ω-OTs (24)

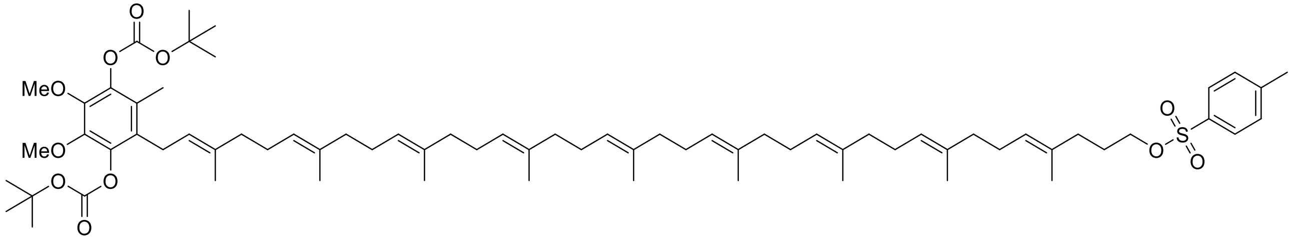

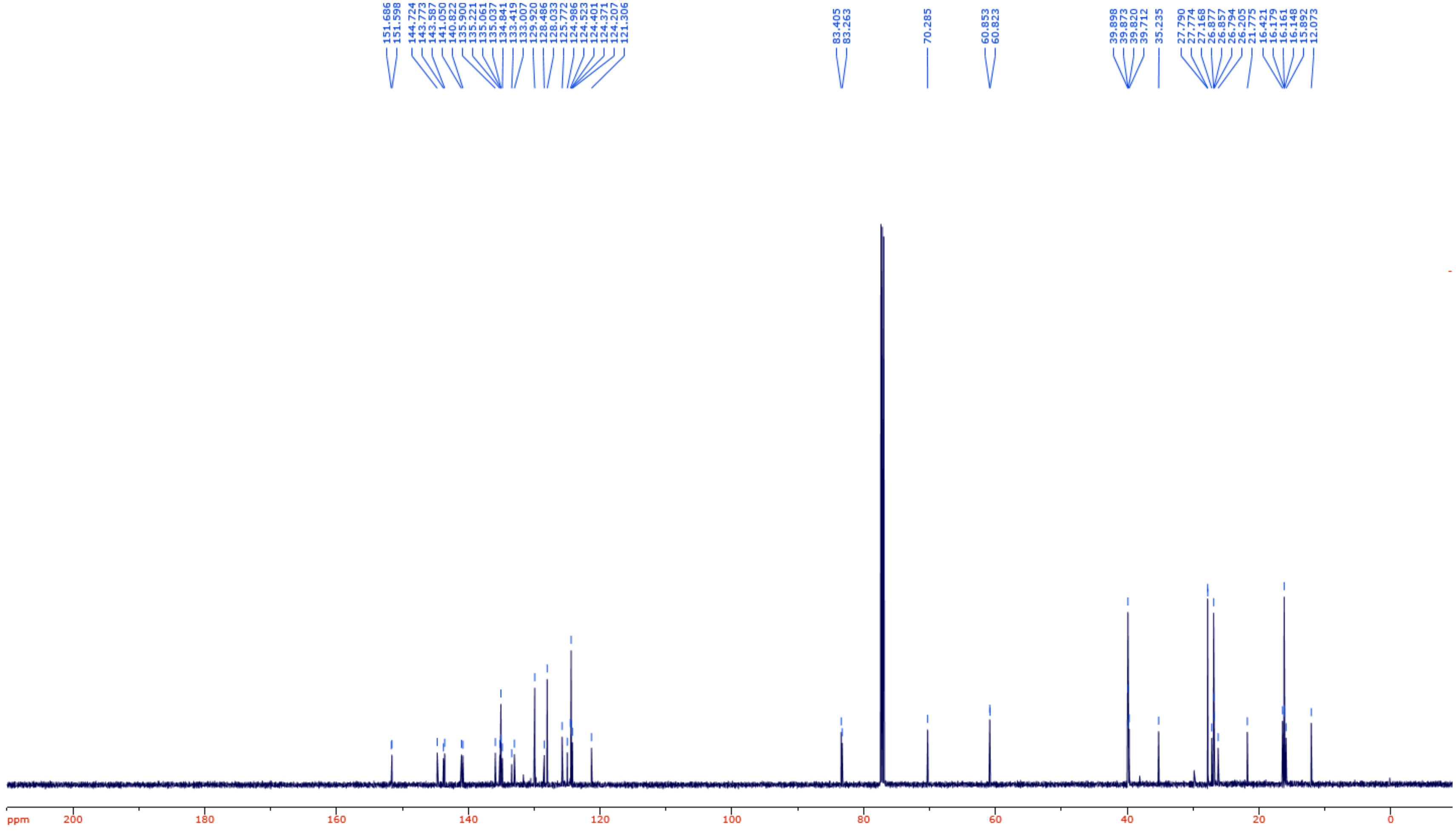

2D COSY (400 MHZ, $CDCl_3$) for Boc_2-CoQ10-ω-OTs (24)

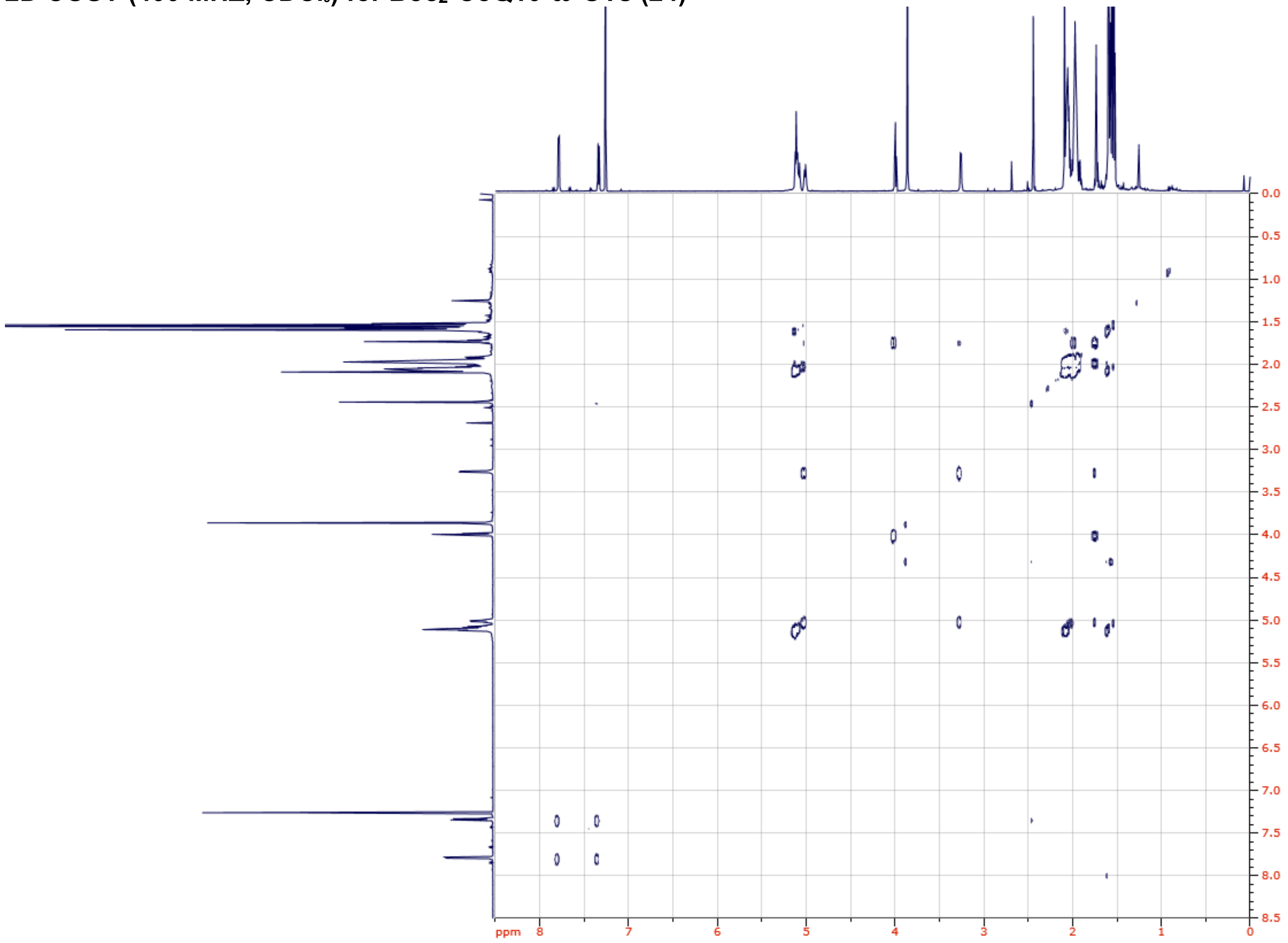

2D HSQC (400 MHZ, $CDCl_3$) for Boc_2-CoQ10-ω-OTs (24)

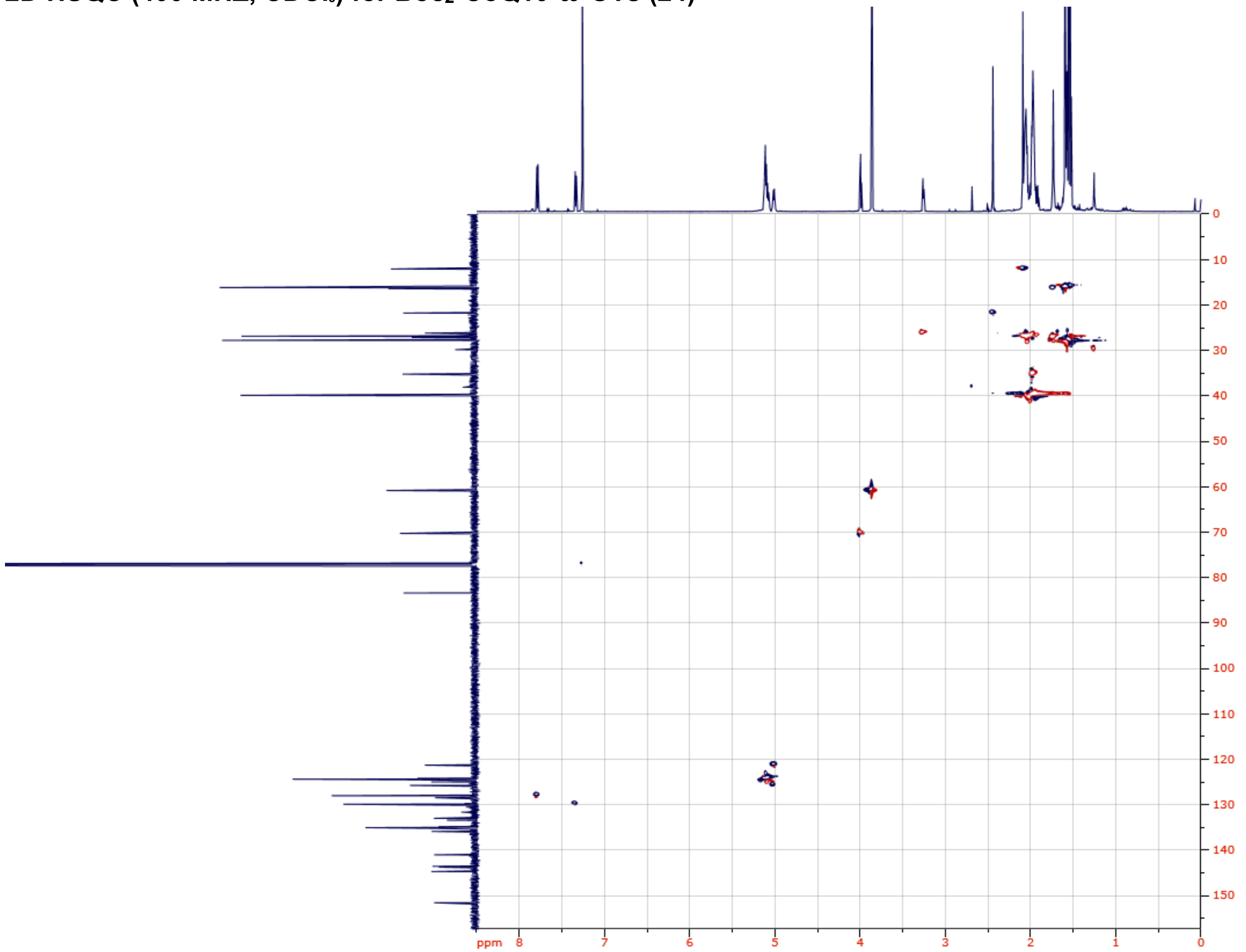

2D HMBC (400 MHZ, $CDCl_3$) for Boc_2-CoQ10-ω-OTs (24)

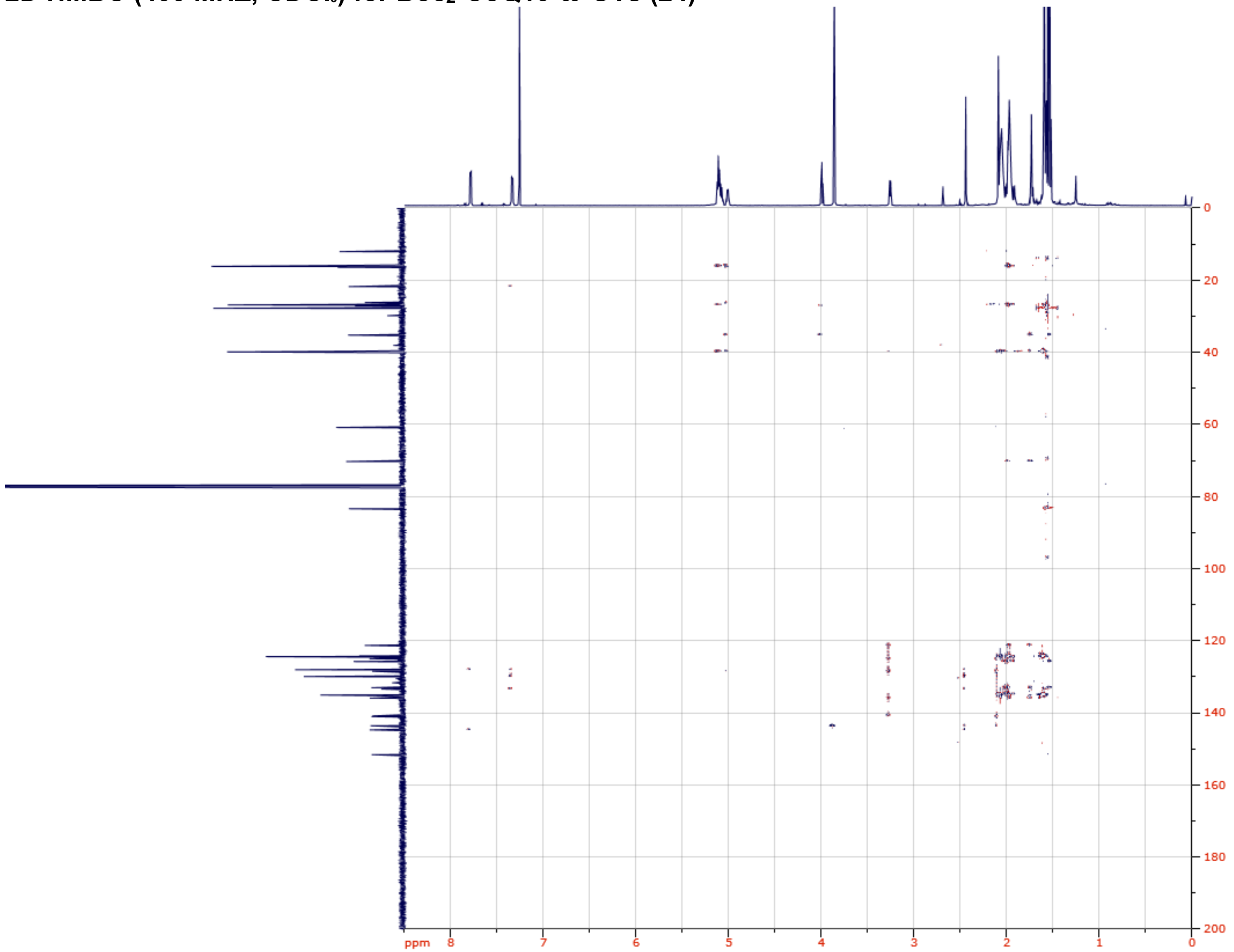

^{1}H NMR (400 MHZ, $CDCl_3$) for Boc_2-MK-9-ω-OTs (25)

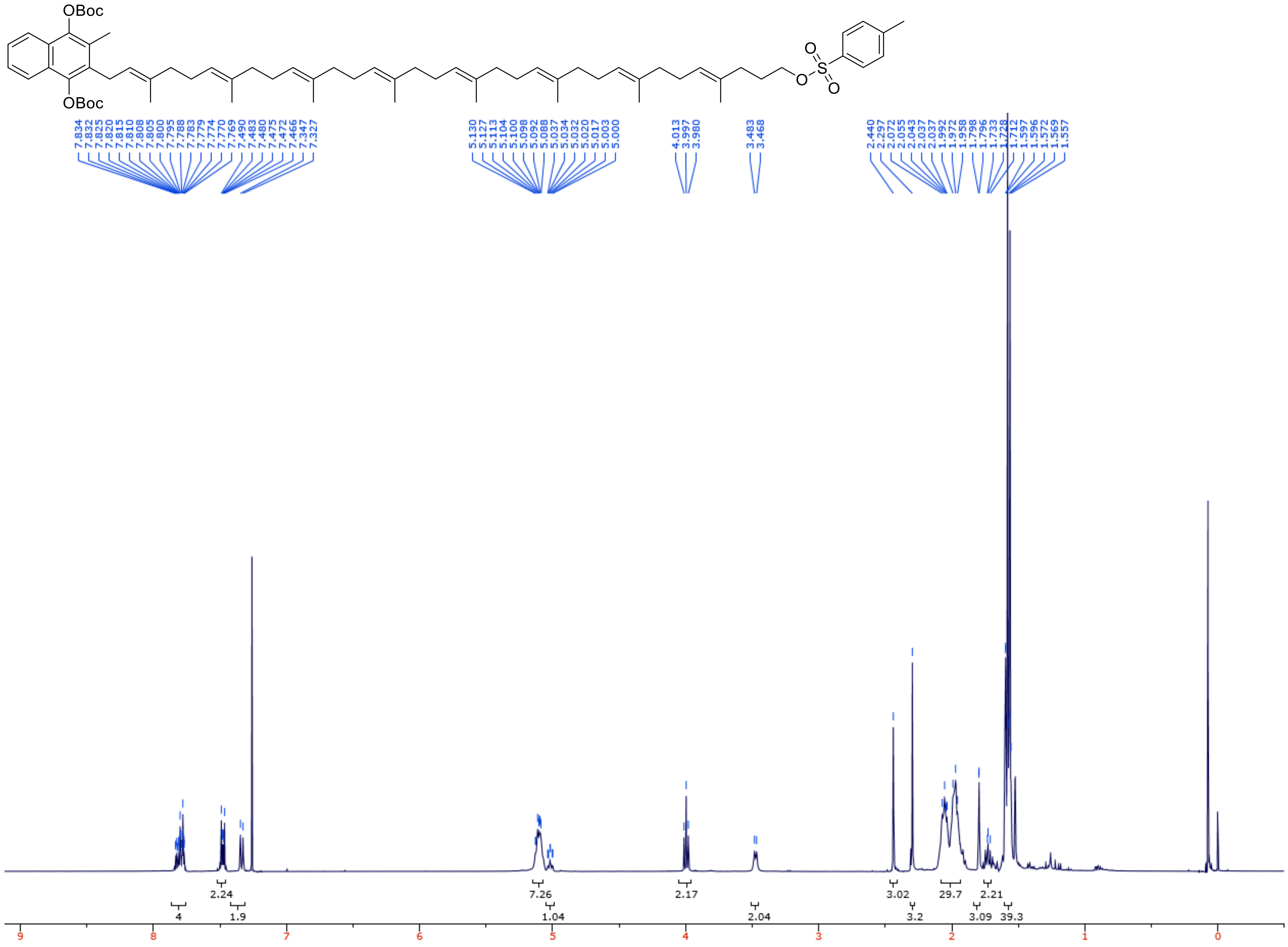

^{13}C NMR (101 MHZ, $CDCl_3$) for Boc_2-MK-9-ω-OTs (25)

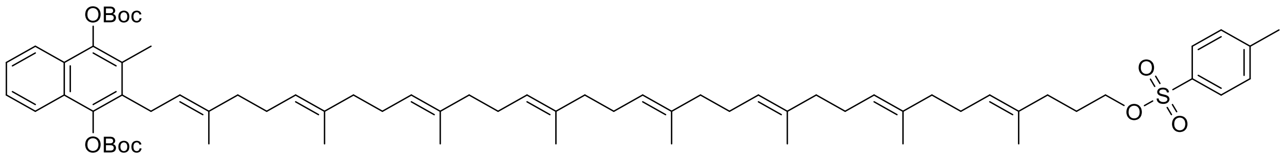

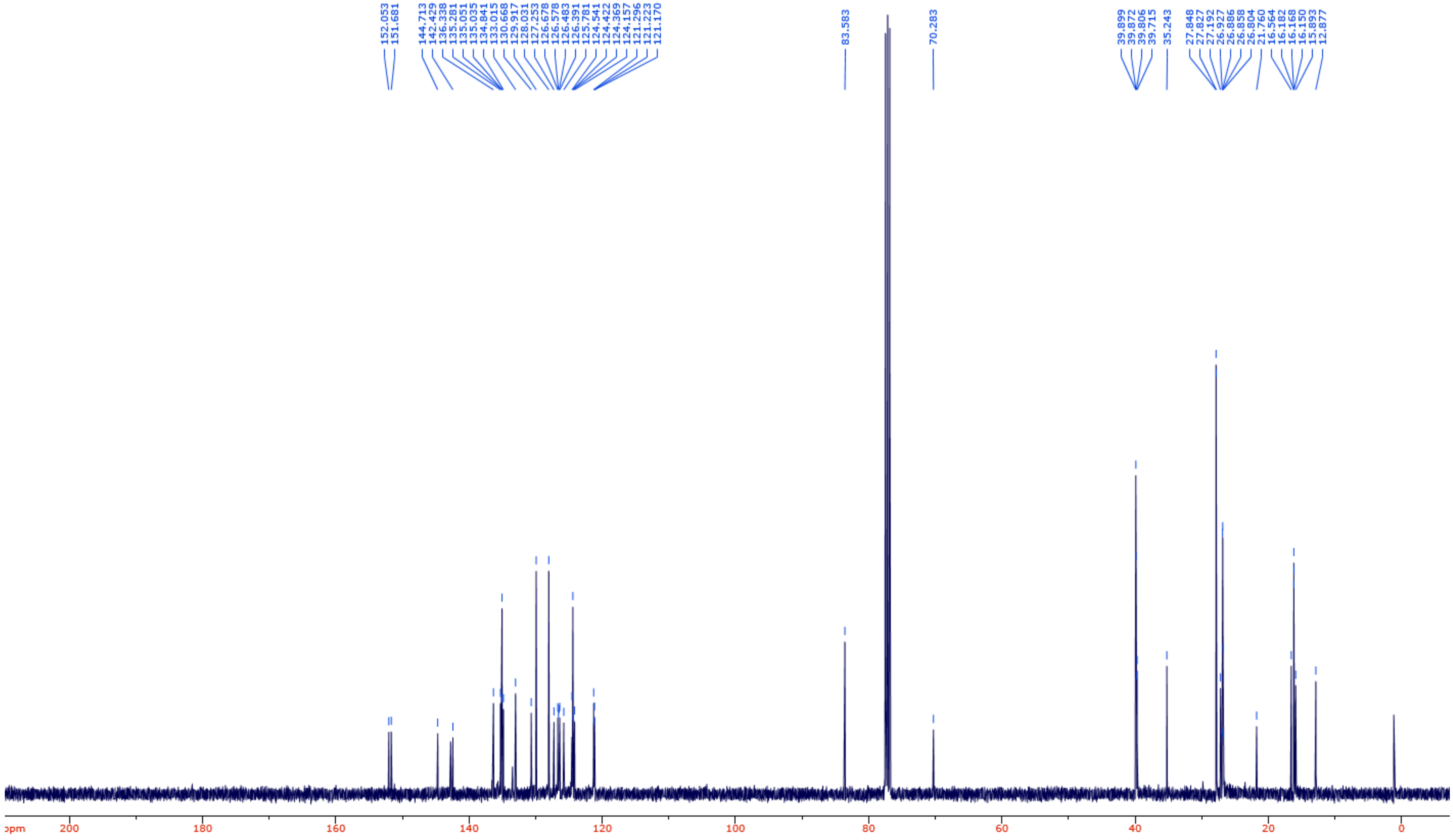

2D COSY (400 MHZ, $CDCl_3$) for Boc_2-MK-9-ω-OTs (25)

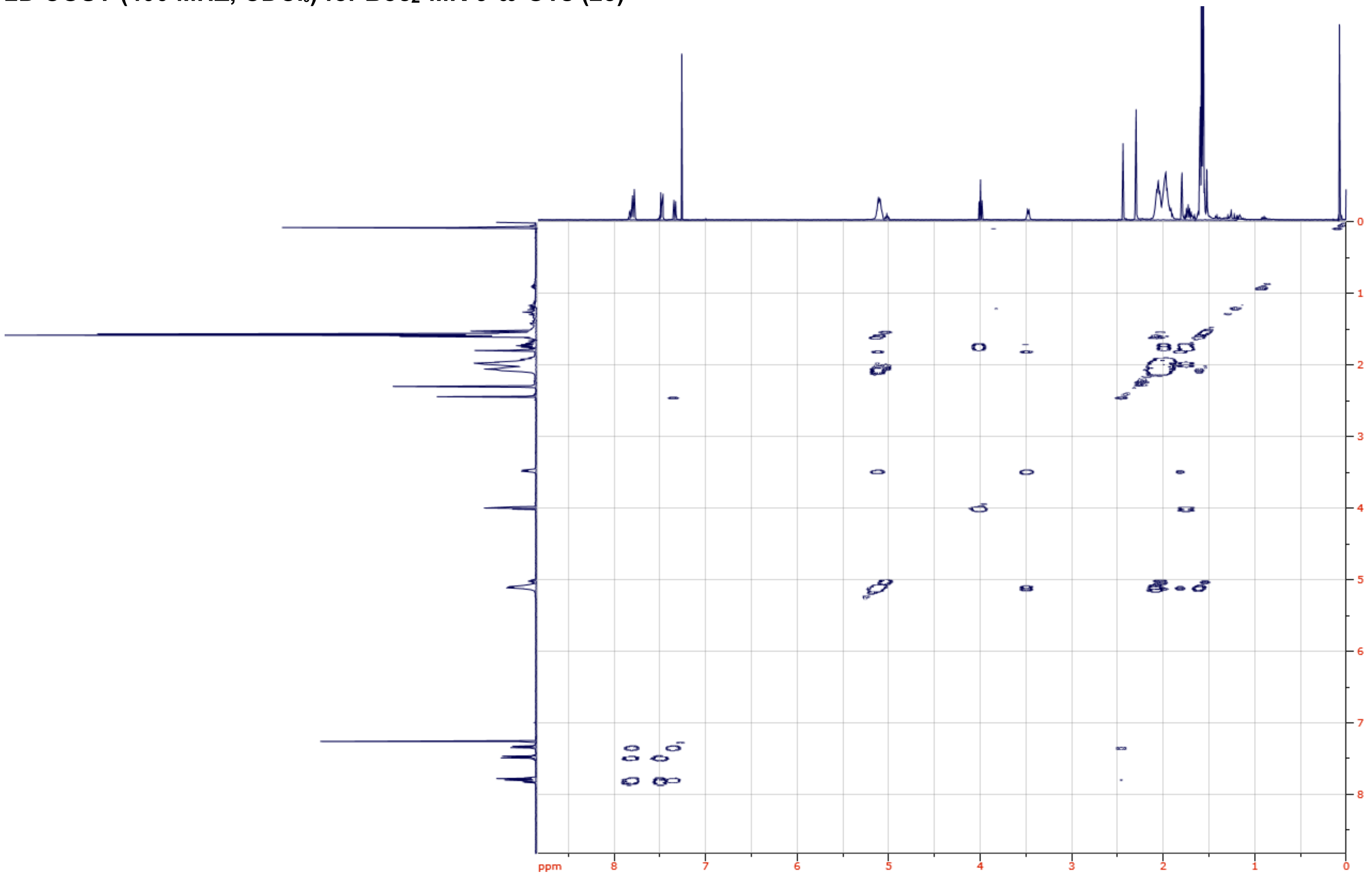

2D HSQC (400 MHZ, $CDCl_3$) for Boc_2-MK-9-ω-OTs (25)

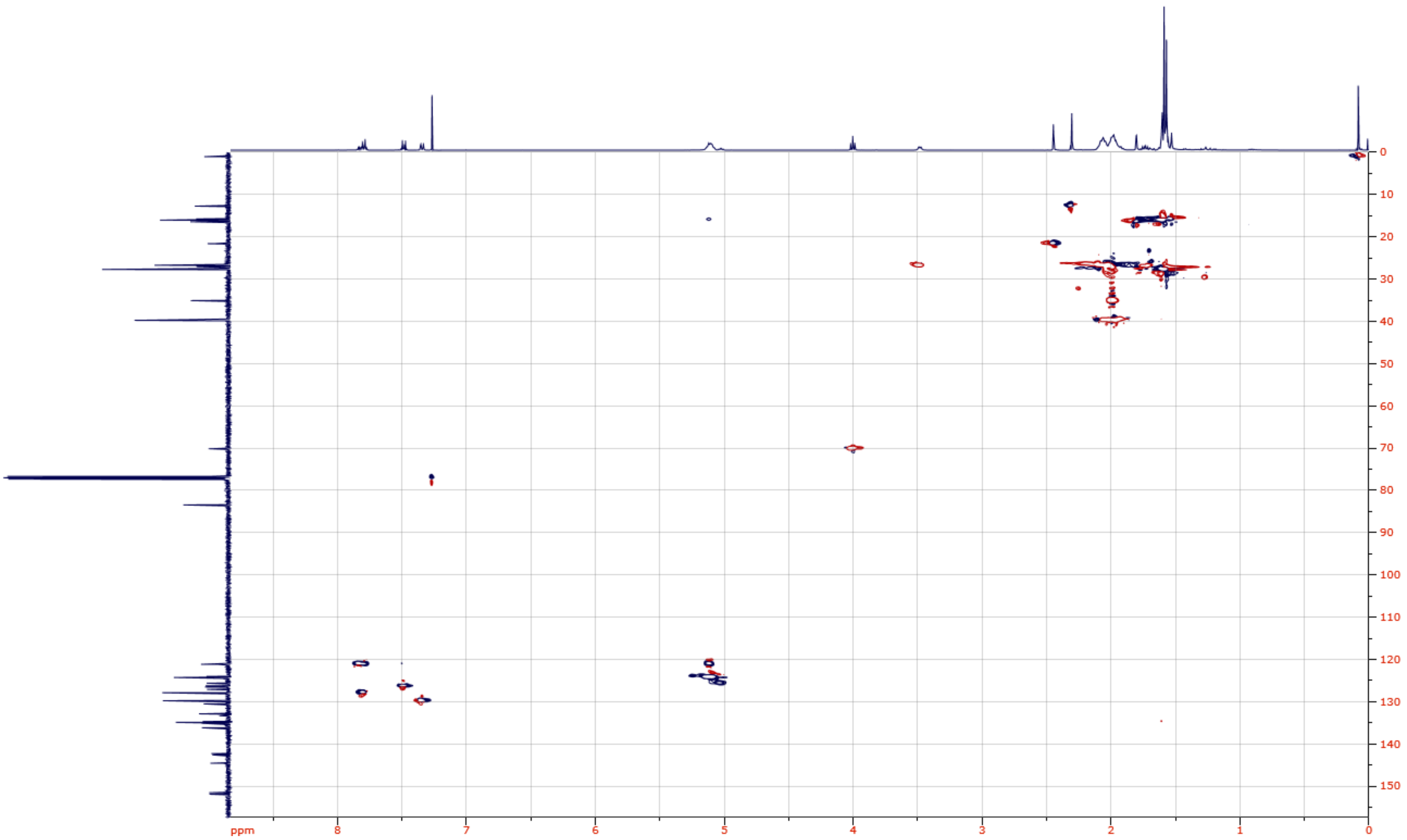

2D HMBC (400 MHZ, $CDCl_3$) for Boc_2-MK-9-ω-OTs (25)

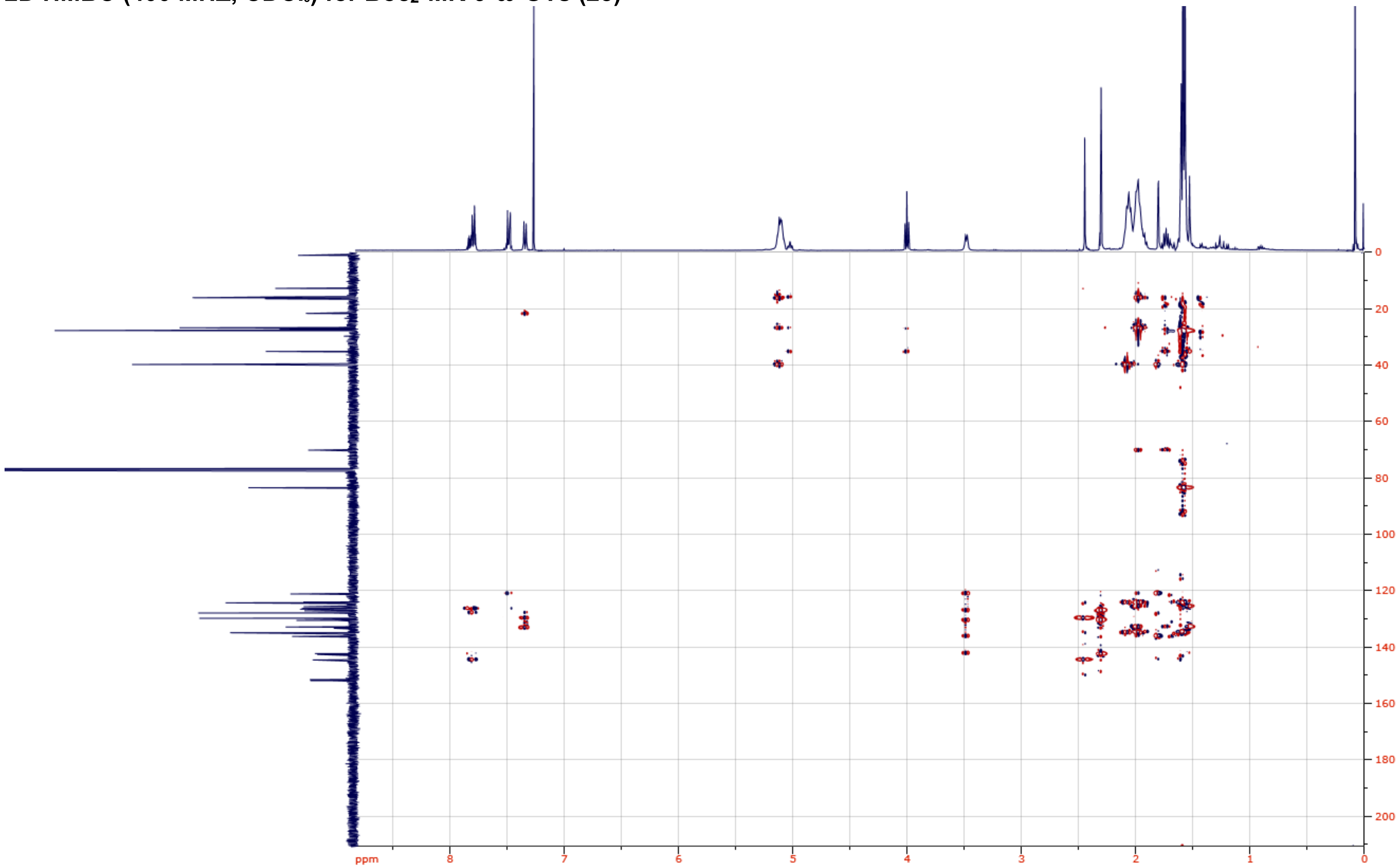

^{1}H NMR (400 MHZ, $CDCl_3$) for Boc_2-CoQ10-ω-azide (26)

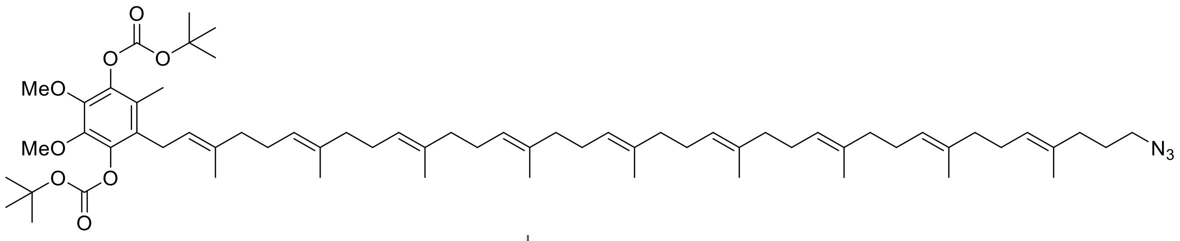

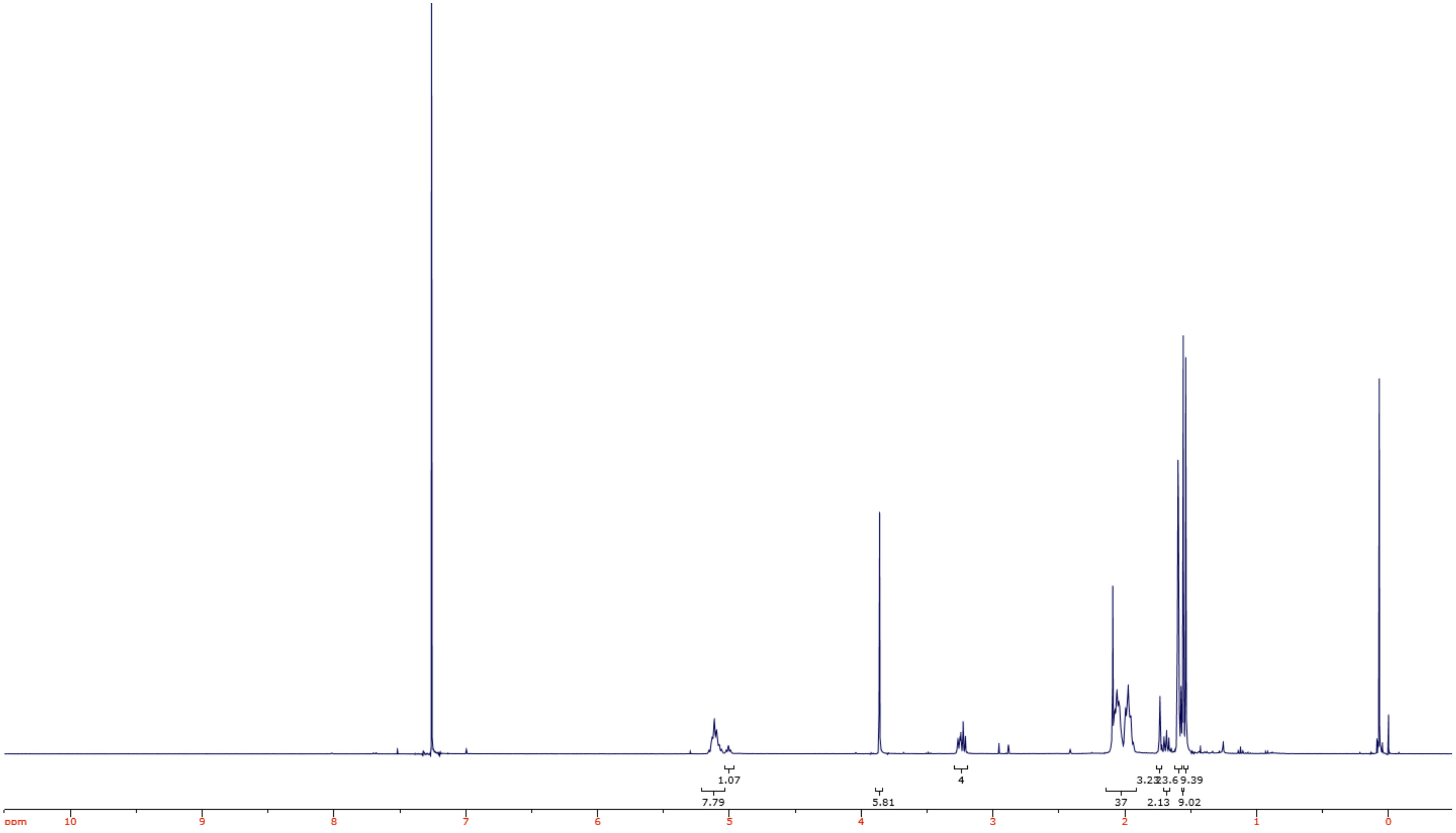

^{13}C NMR (101 MHZ, $CDCl_3$) for Boc$_2$-CoQ10-ω-azide (26)

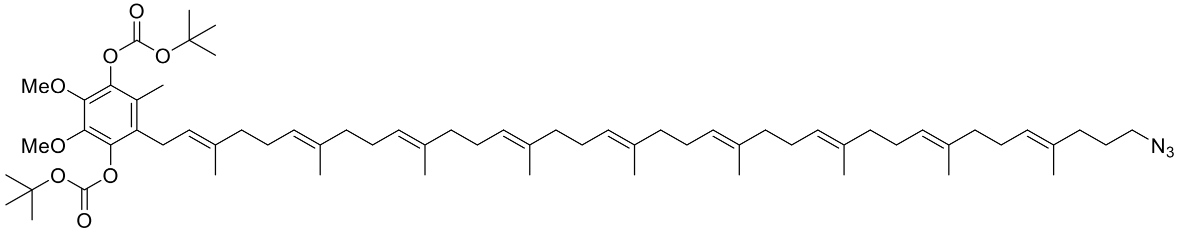

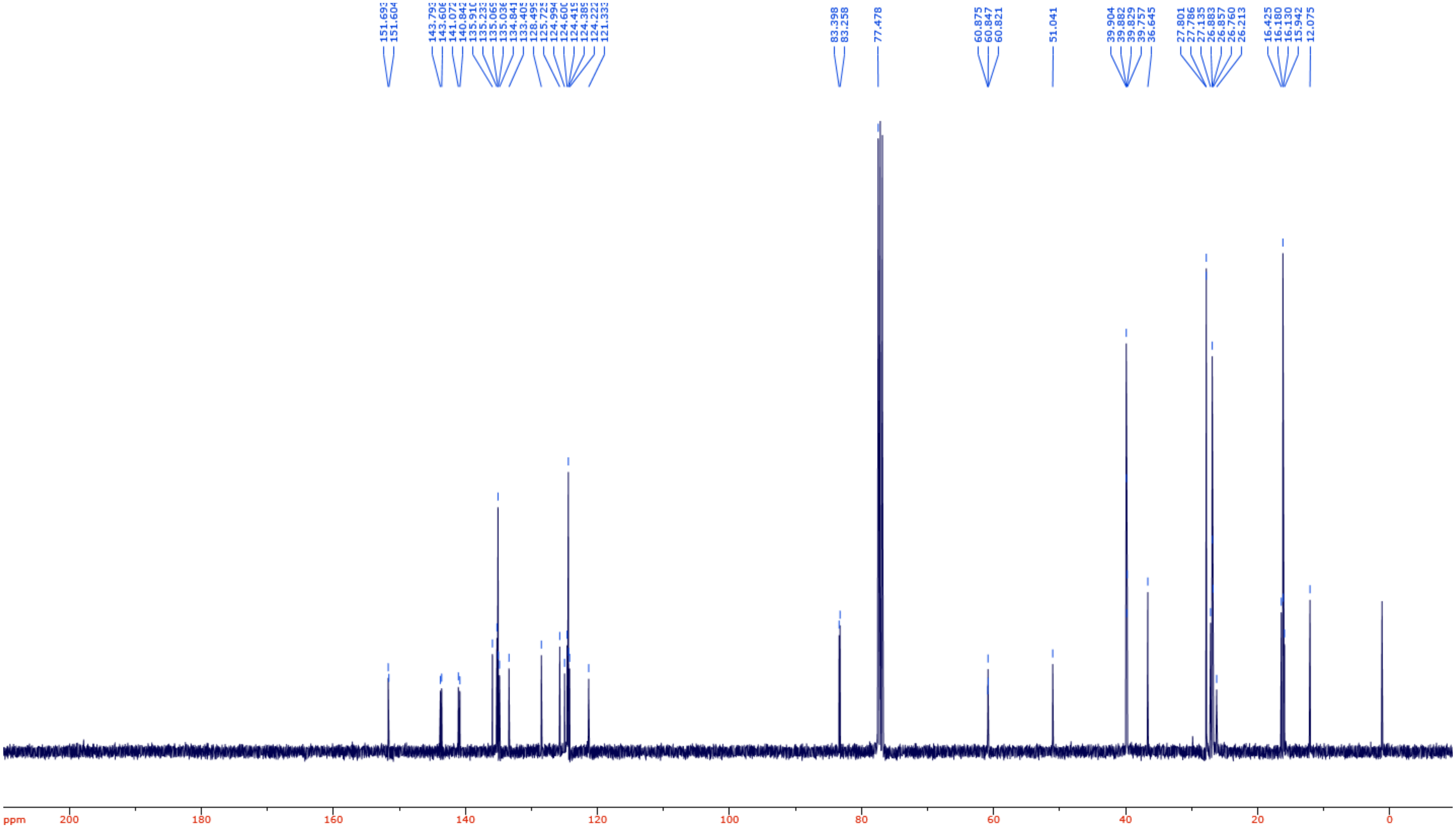

2D COSY (400 MHZ, $CDCl_3$) for Boc_2-CoQ10-ω-azide (26)

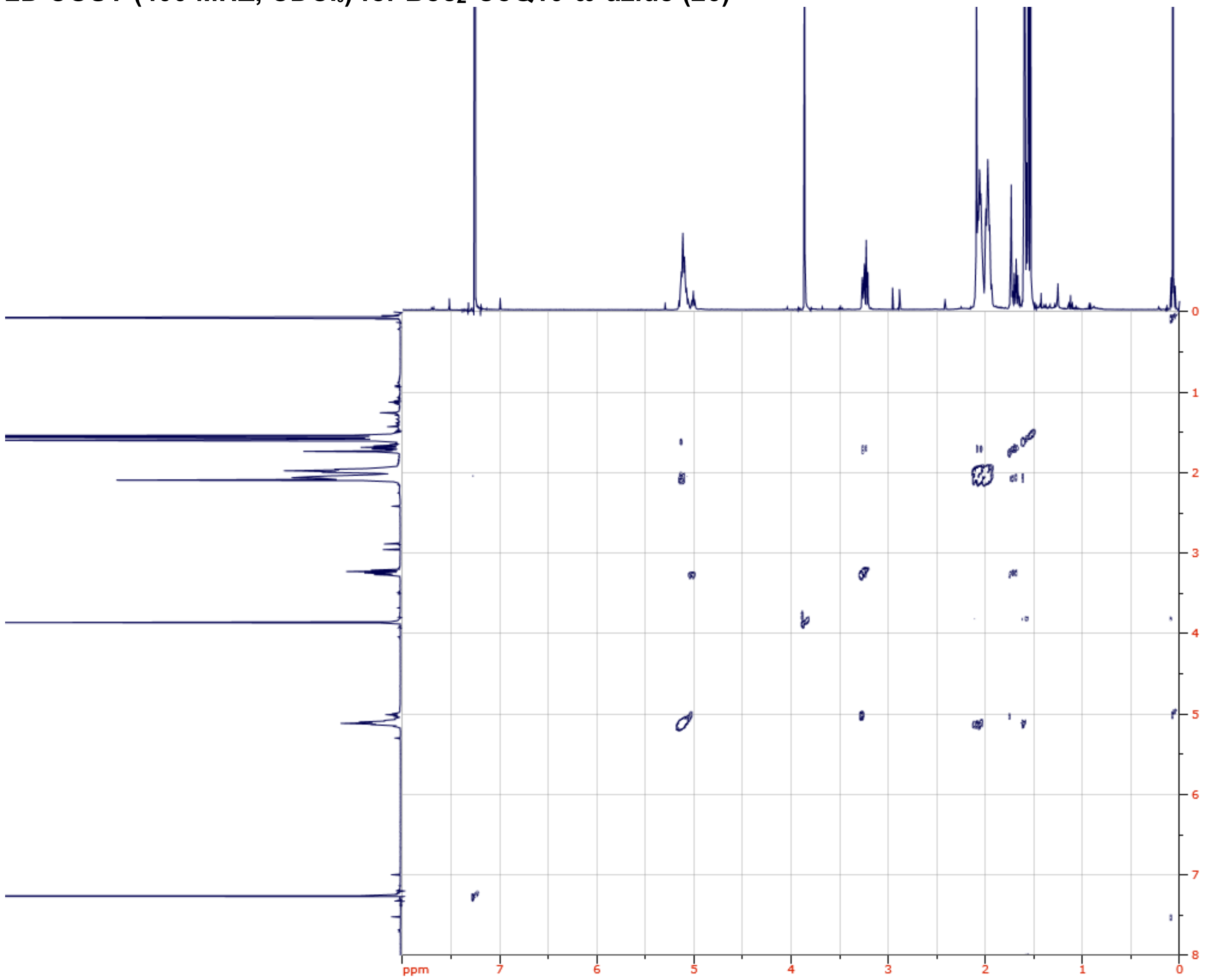

2D HSQC (400 MHZ, $CDCl_3$) for Boc_2-CoQ10-ω-azide (26)

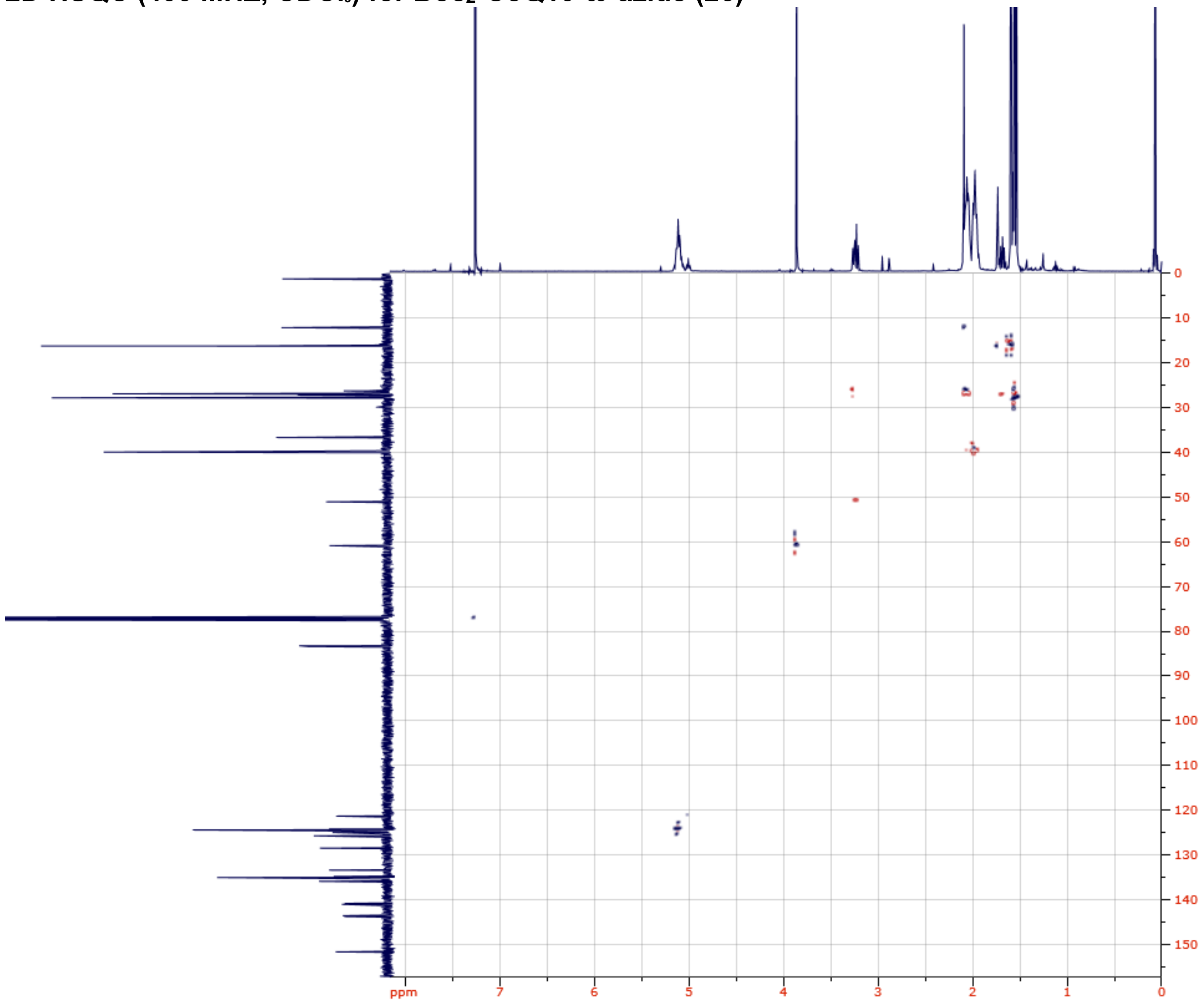

2D HMBC (400 MHZ, $CDCl_3$) for Boc_2-CoQ10-ω-azide (26)

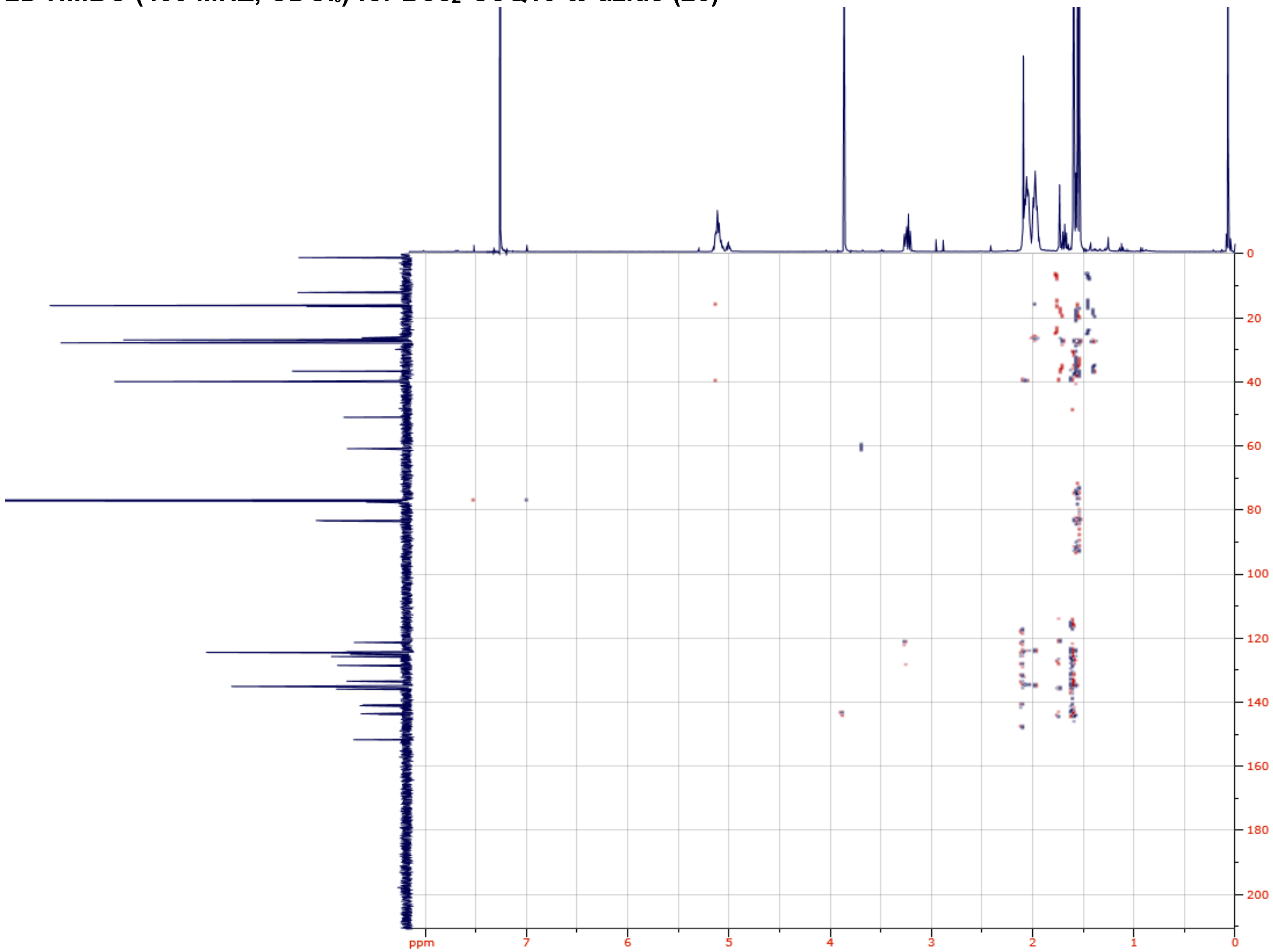

^{1}H NMR (400 MHZ, $CDCl_3$) for Boc_2-MK9-ω-azide (27)

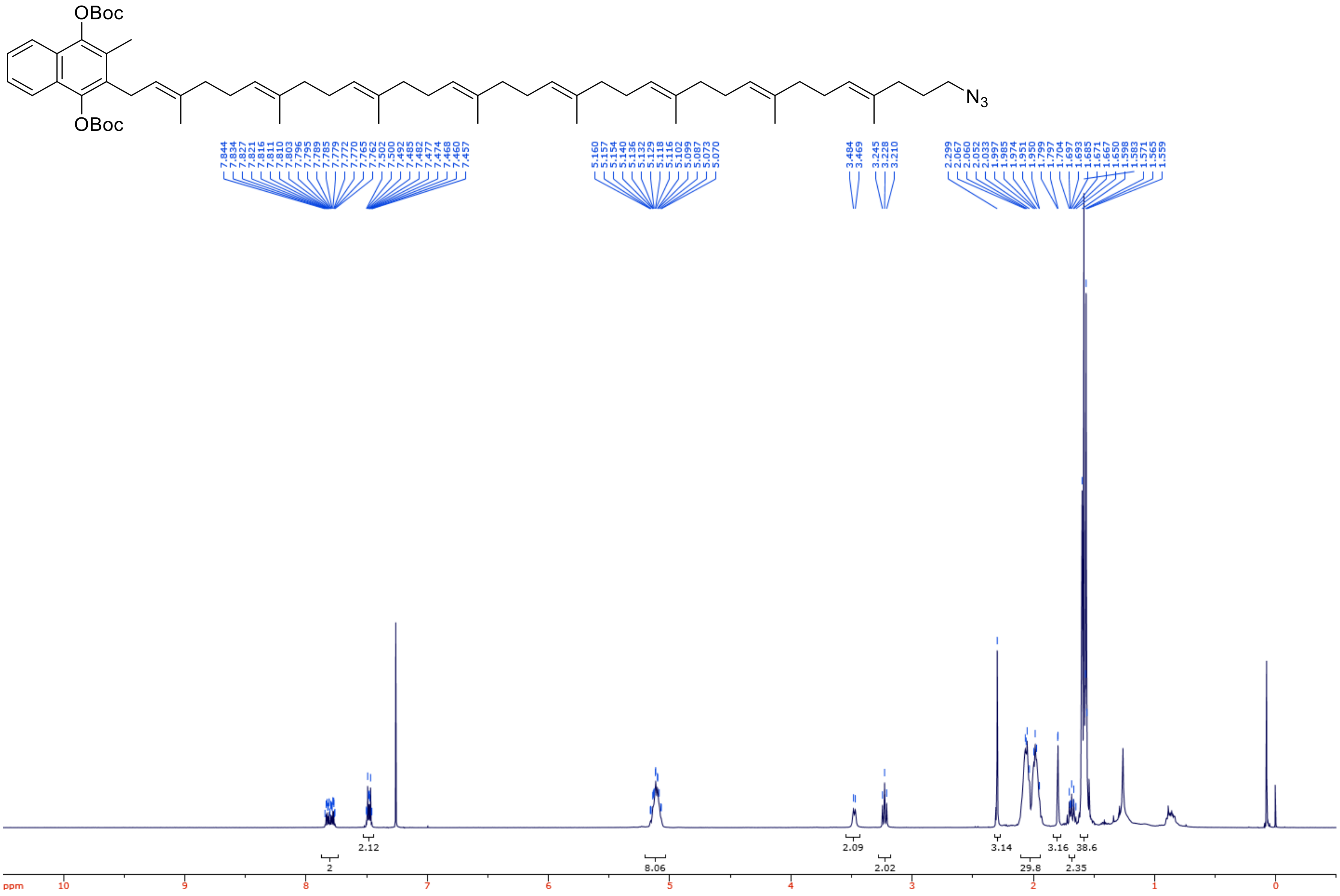

^{13}C NMR (101 MHz, $CDCl_3$) for Boc_2-MK9-ω-azide (27)

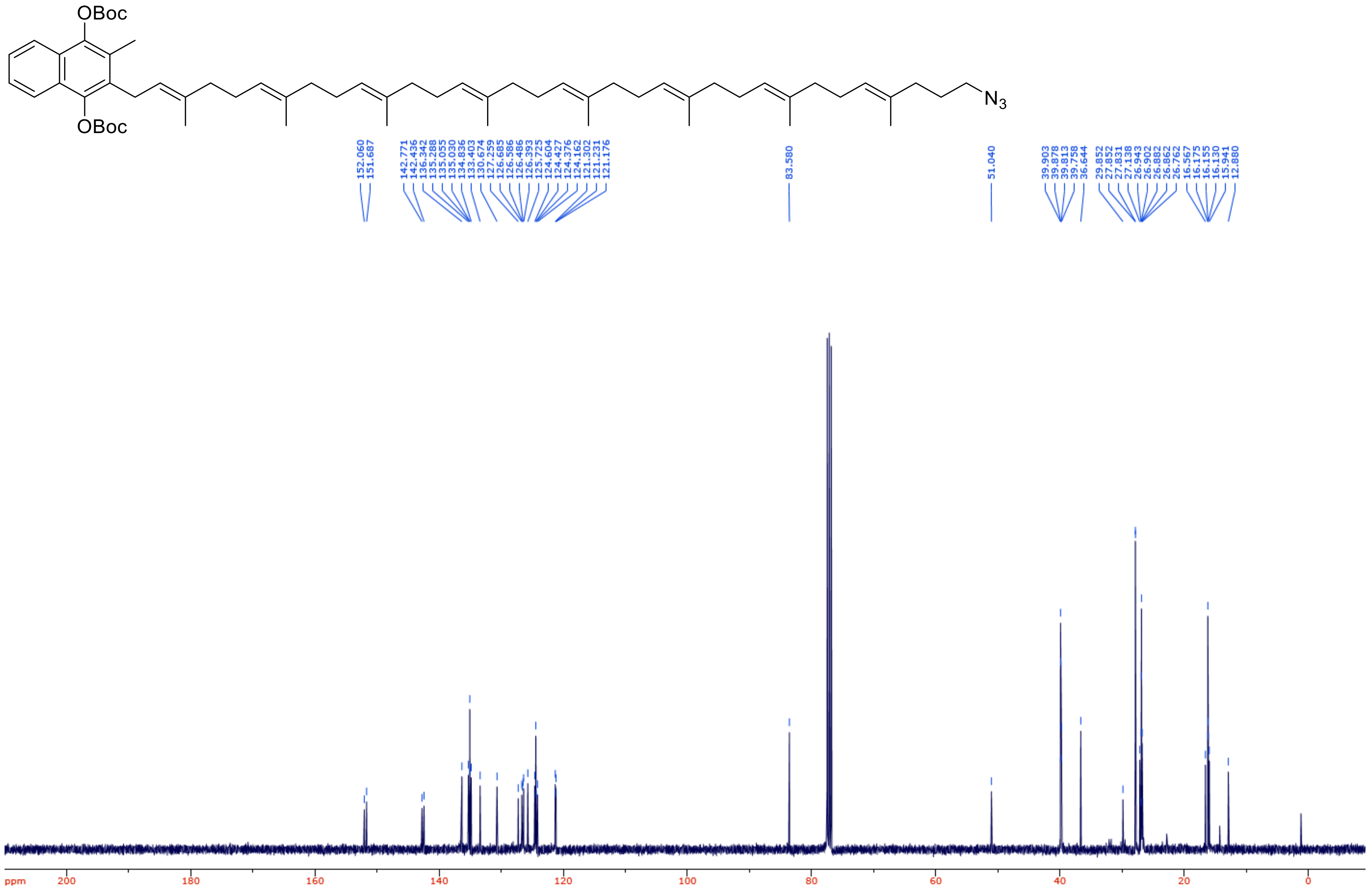

2D COSY (400 MHZ, $CDCl_3$) for Boc_2-MK9-ω-azide (27)

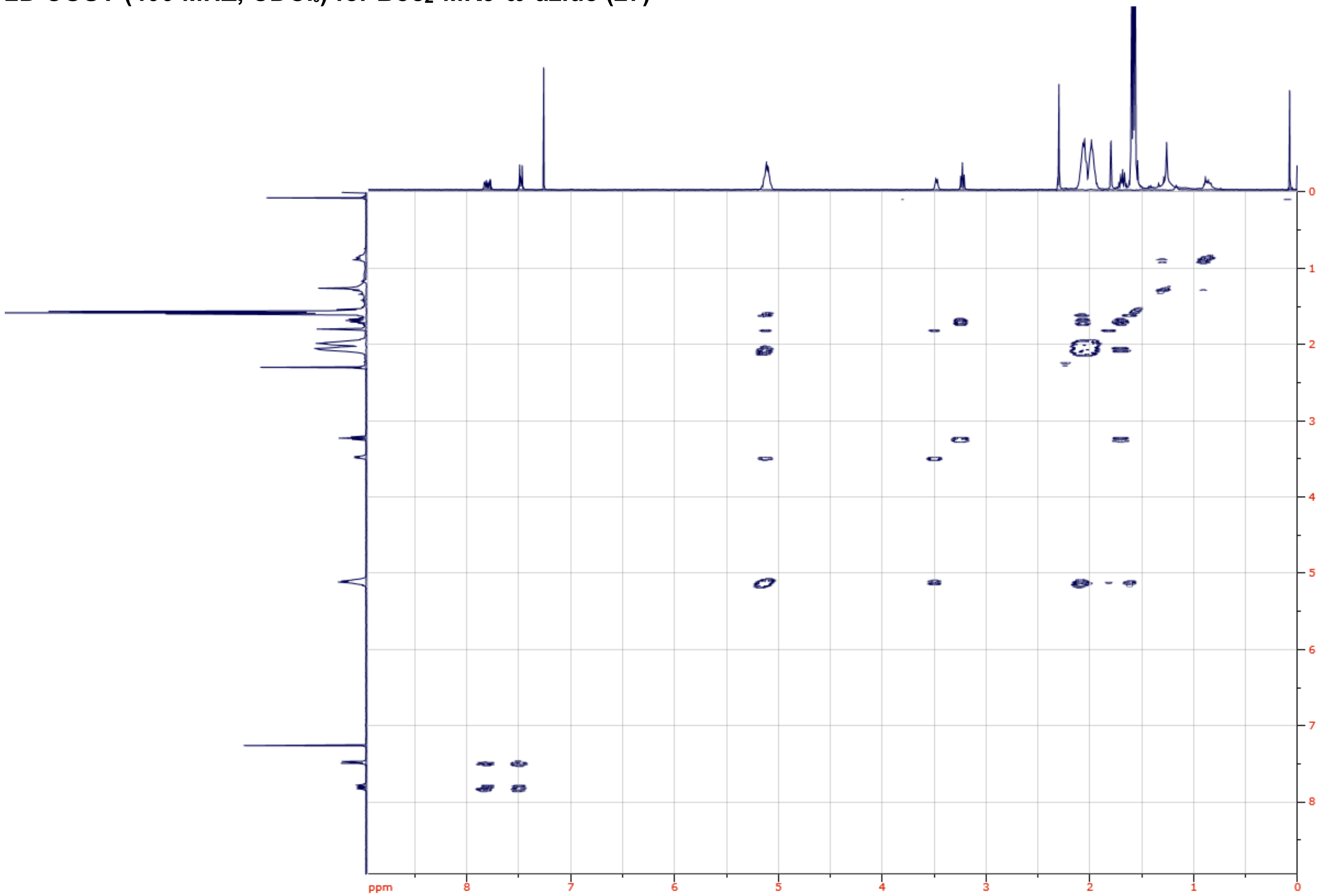

2D HSQC (400 MHZ, $CDCl_3$) for Boc_2-MK9-ω-azide (27)

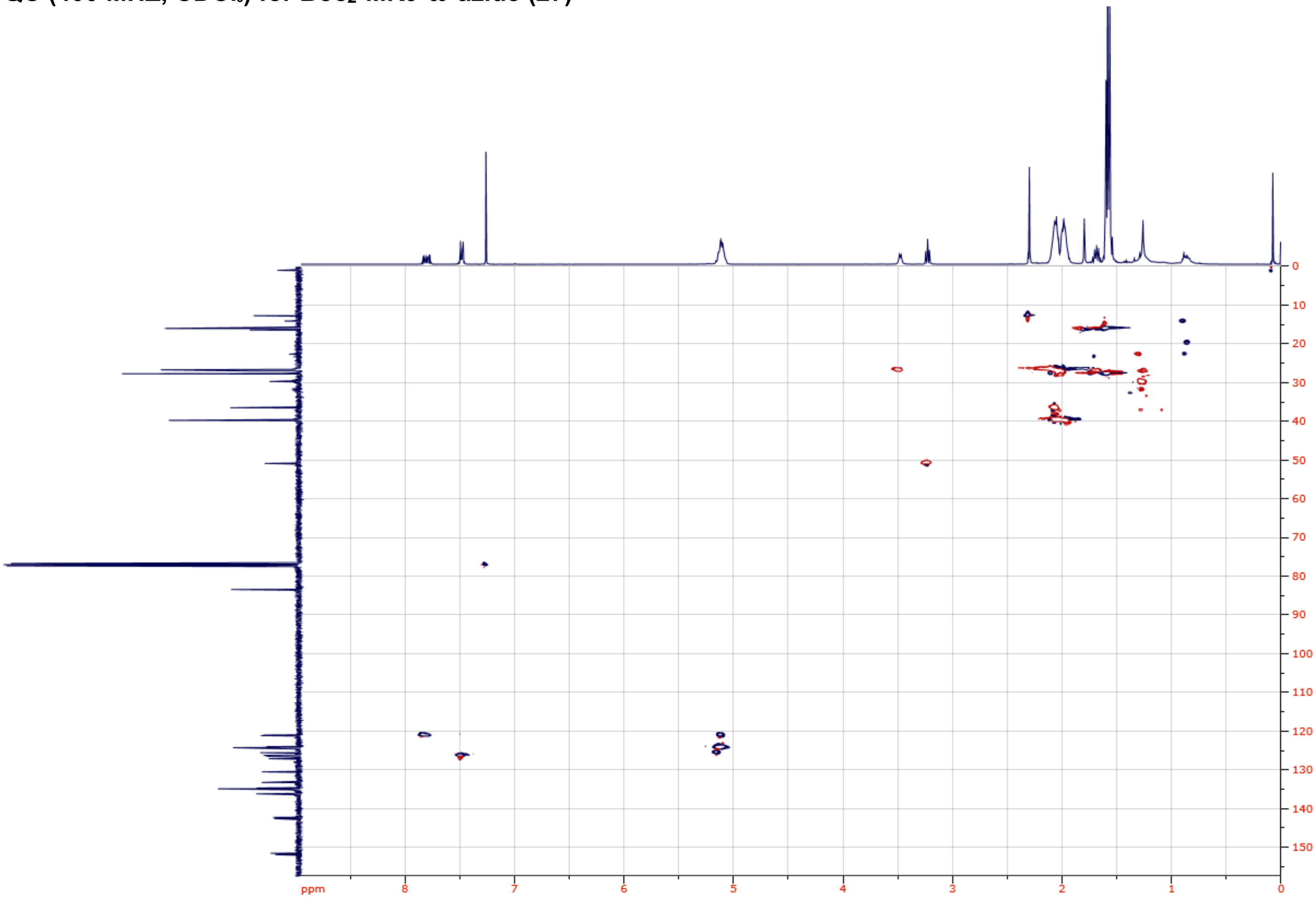

2D HMBC (400 MHZ, $CDCl_3$) for Boc_2-MK9-ω-azide (27)

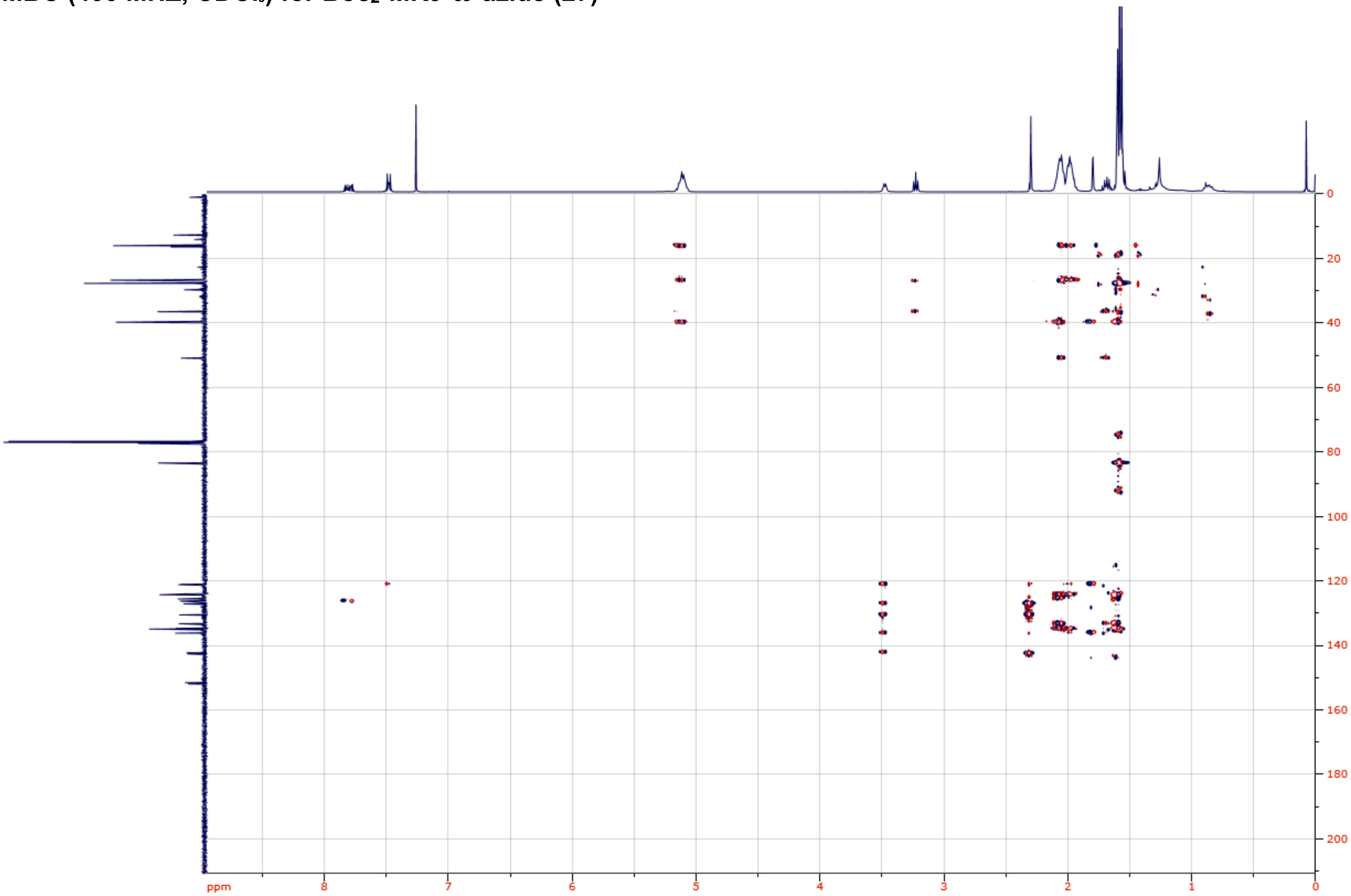

^{1}H NMR (400 MHZ, $CDCl_3$) for CoQ10-ω-azide (28)

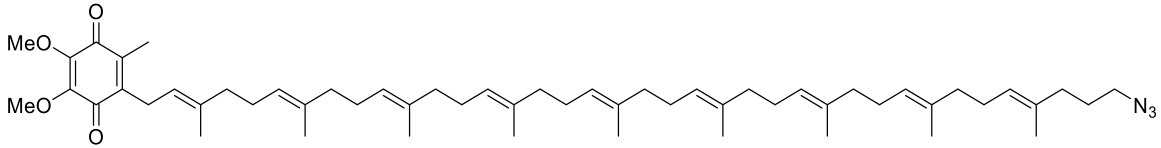

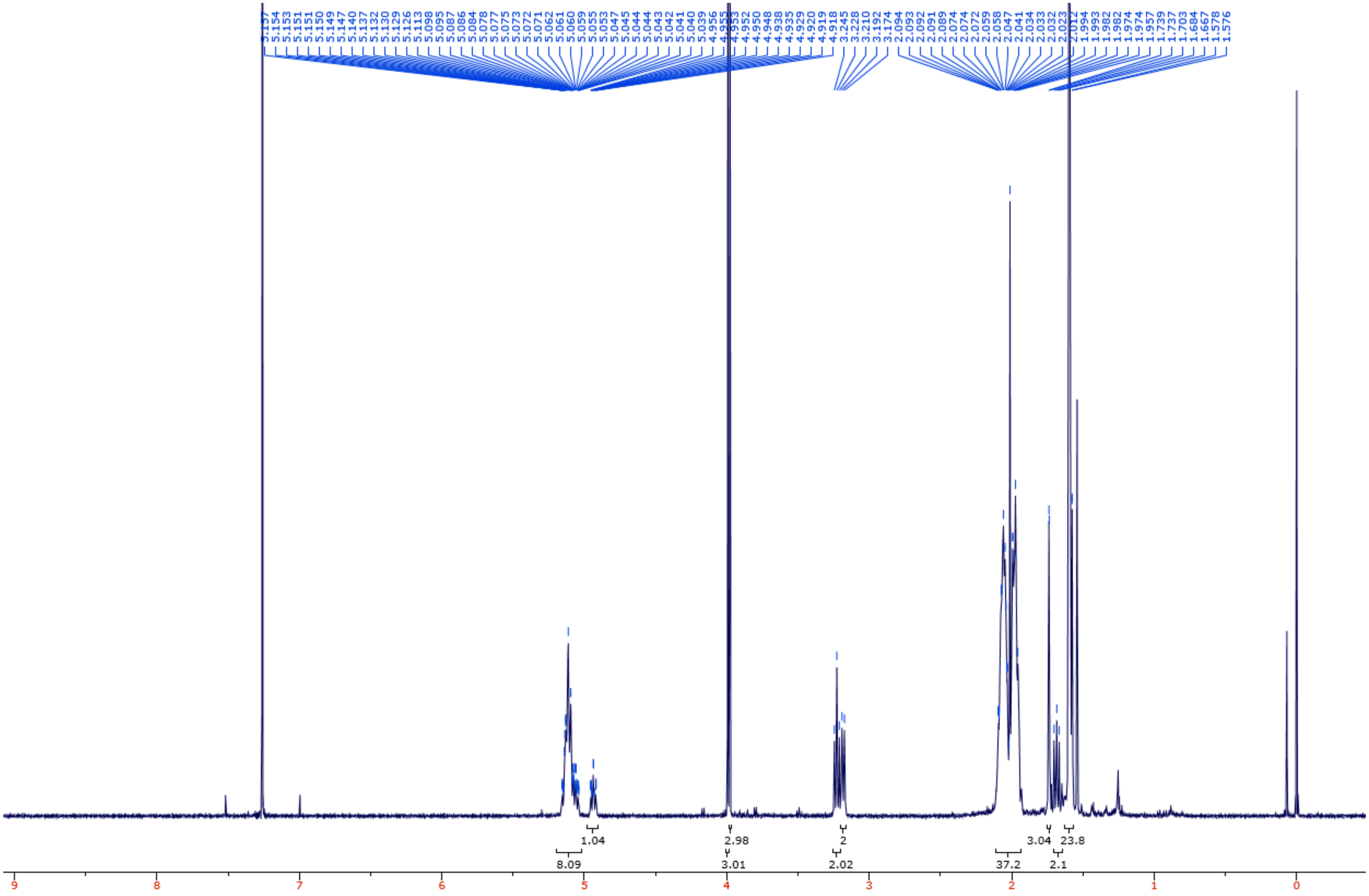

^{13}C NMR (101 MHZ, CDCl$_3$) for CoQ10-ω-azide (28)

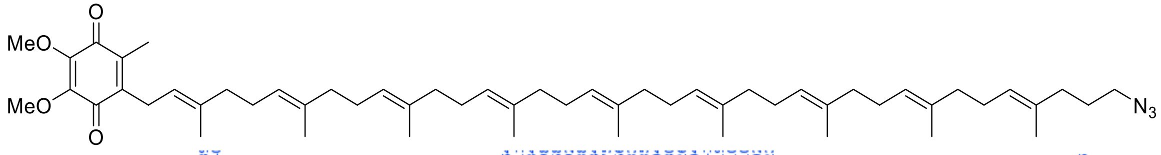

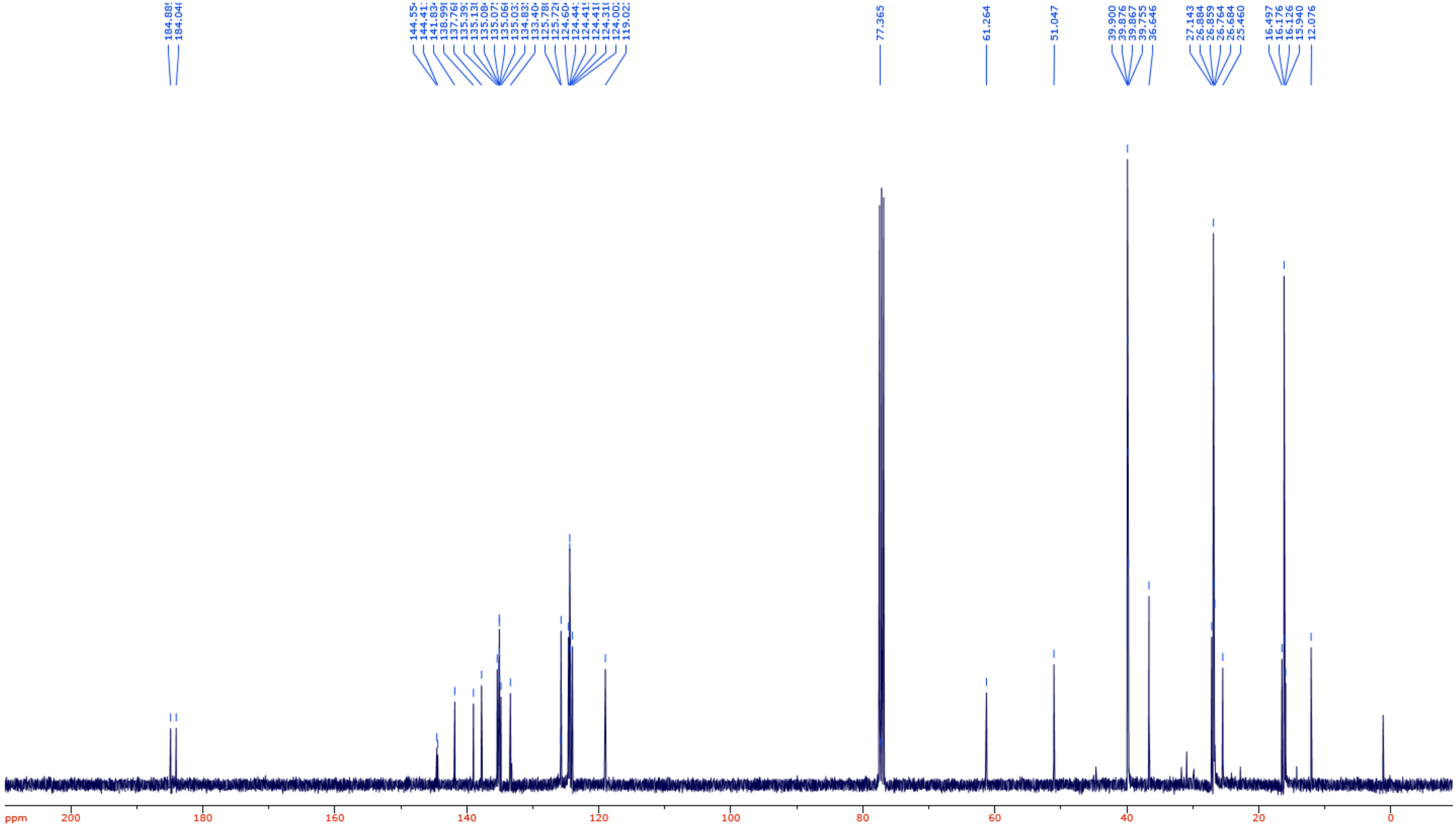

2D COSY (400 MHZ, $CDCl_3$) for CoQ10-ω-azide (28)

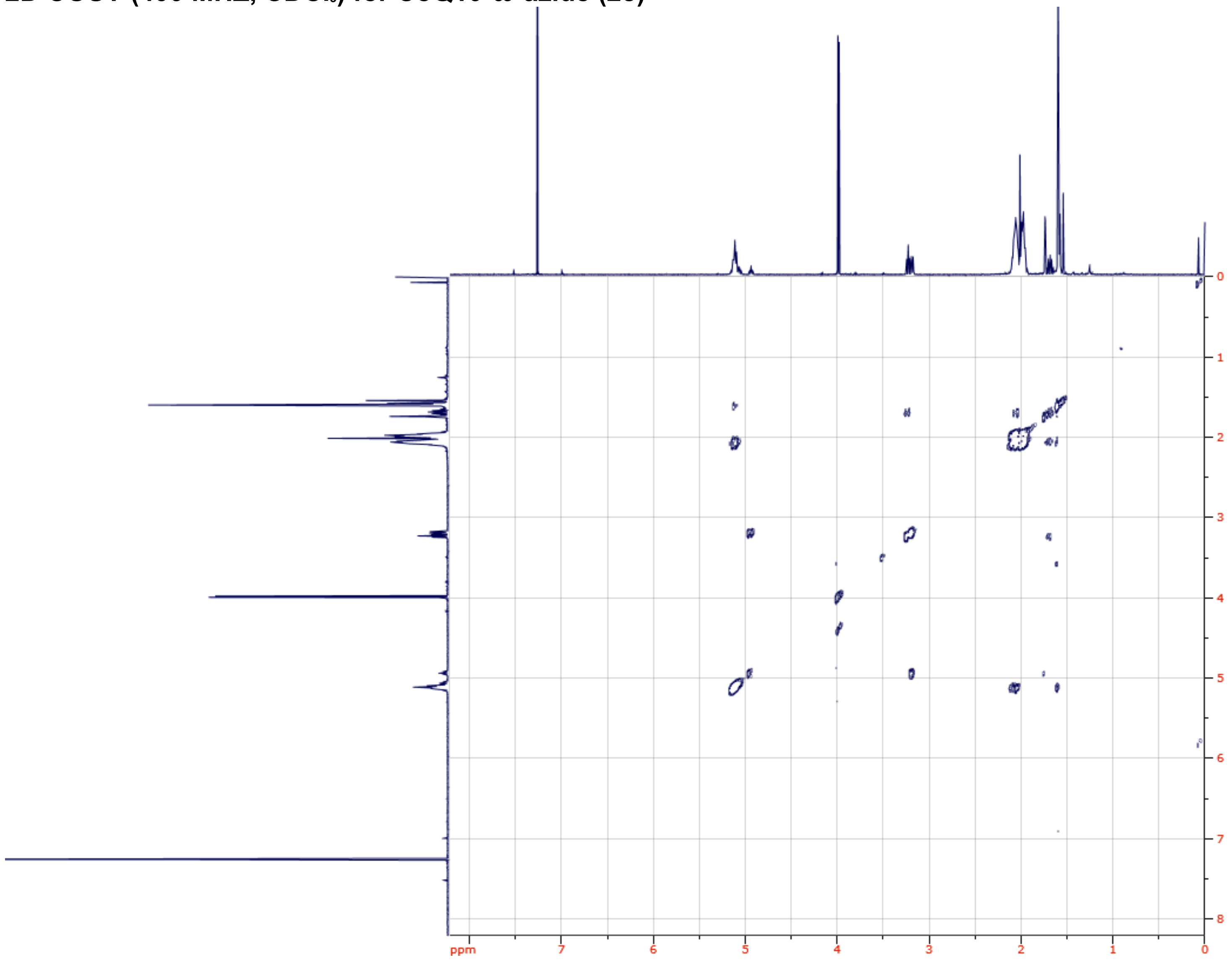

2D HSQC (400 MHZ, $CDCl_3$) for CoQ10-ω-azide (28)

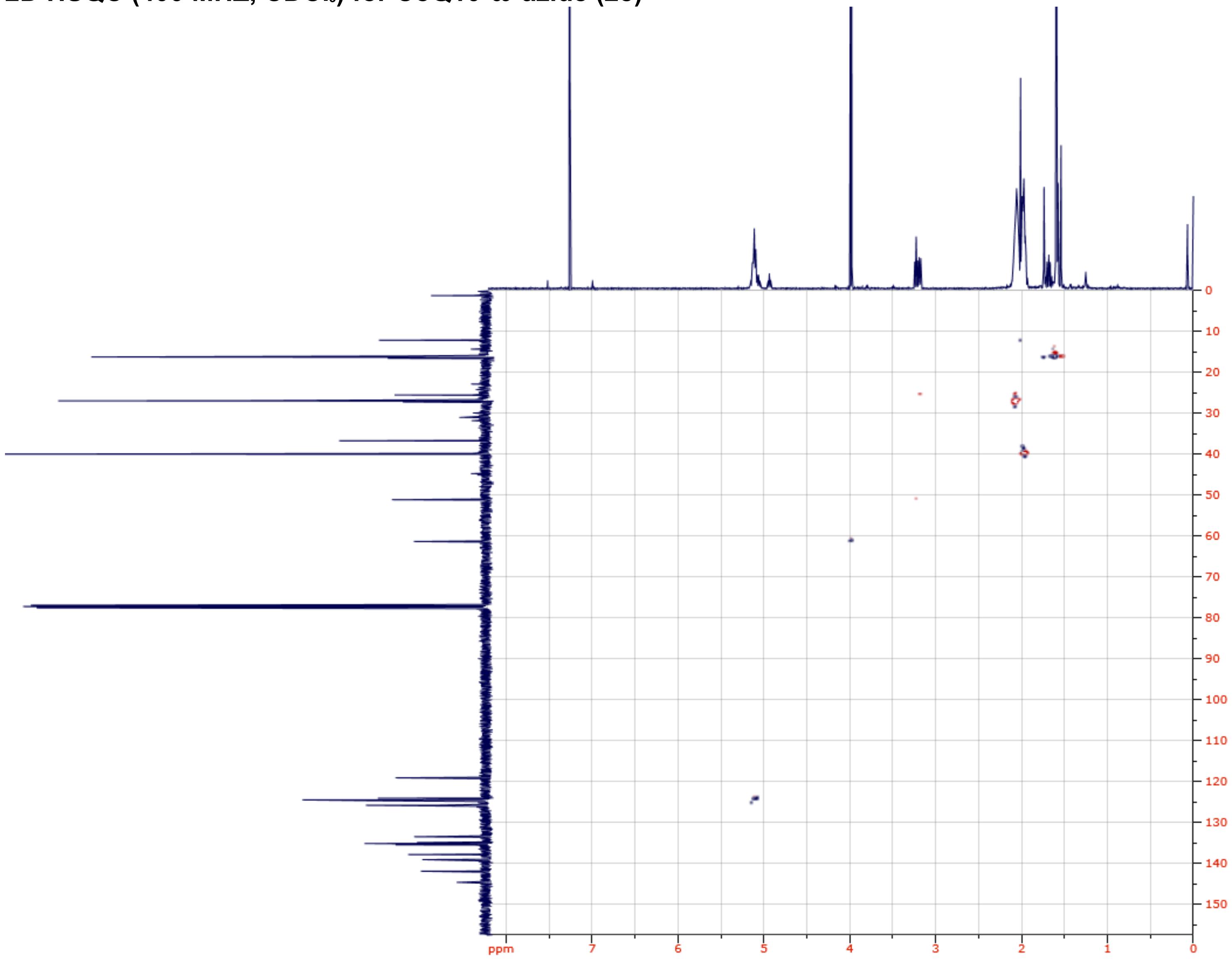

2D HMBC (400 MHZ, $CDCl_3$) for CoQ10-ω-azide (28)

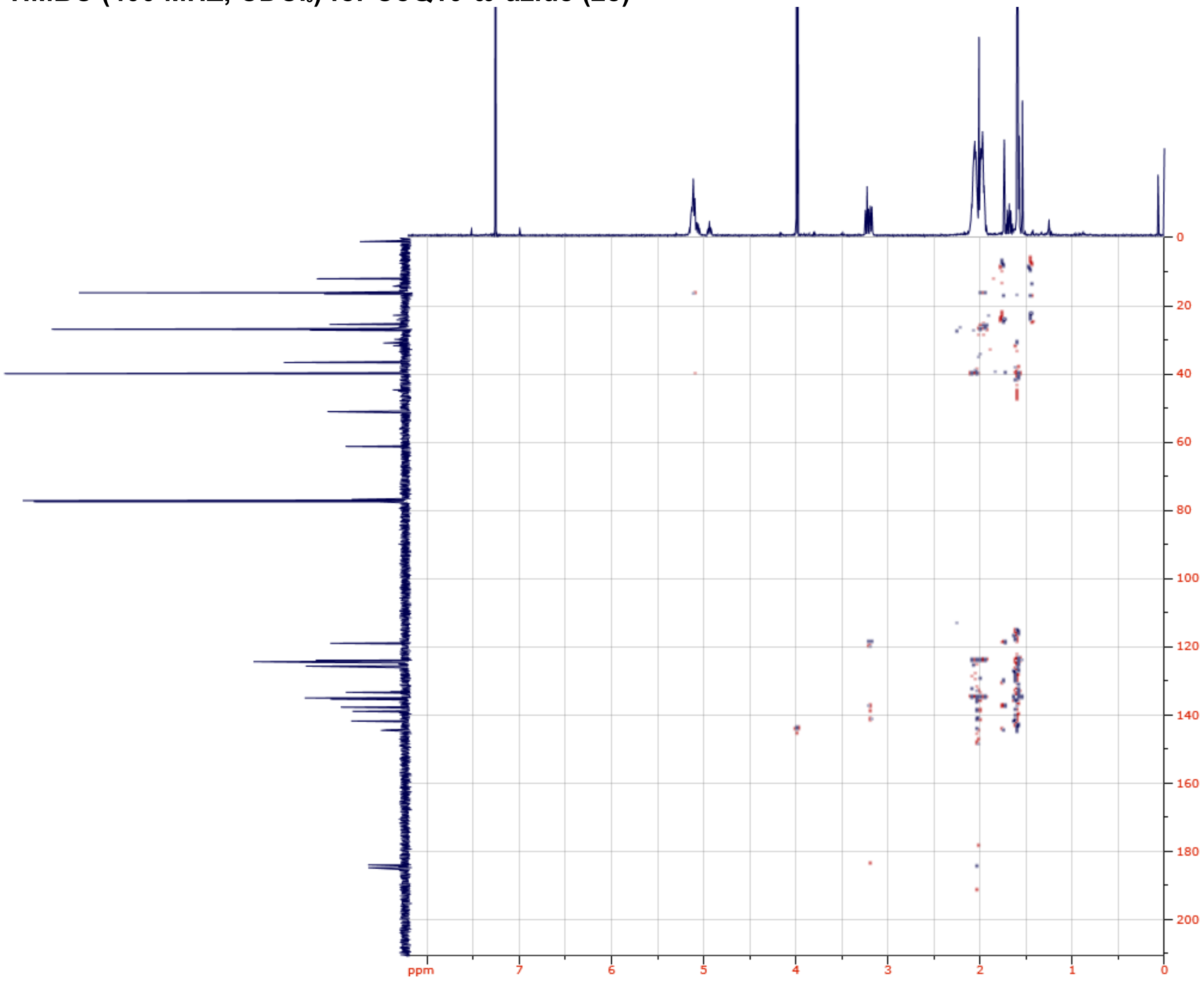

^{1}H NMR (400 MHZ, $CDCl_3$) for MK9-ω-azide (29)

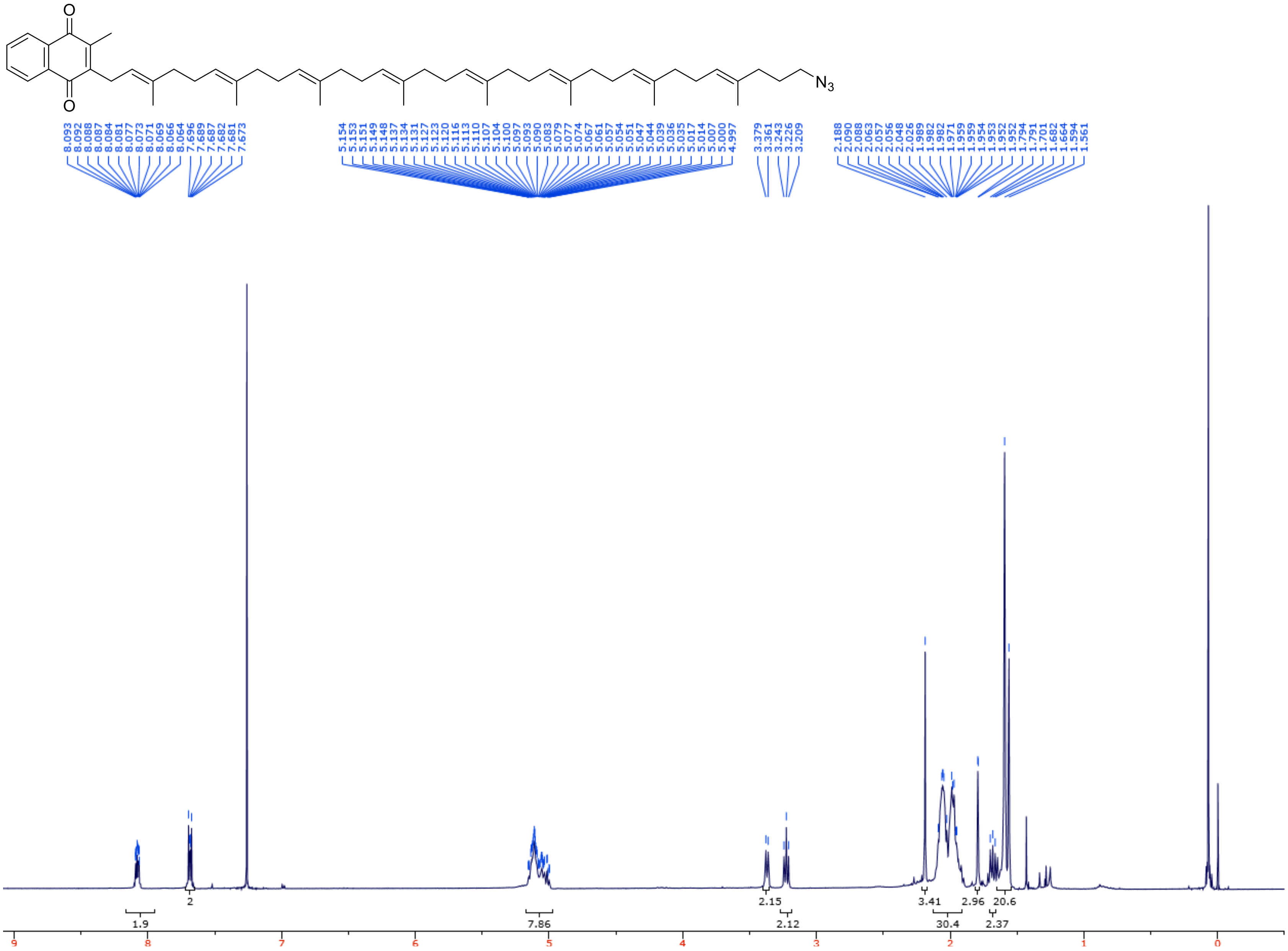

^{13}C NMR (101 MHZ, $CDCl_3$) for MK9-ω-azide (29)

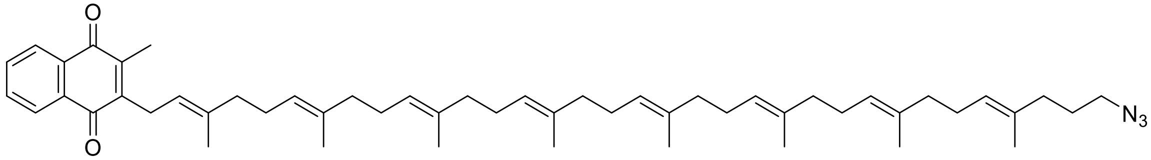

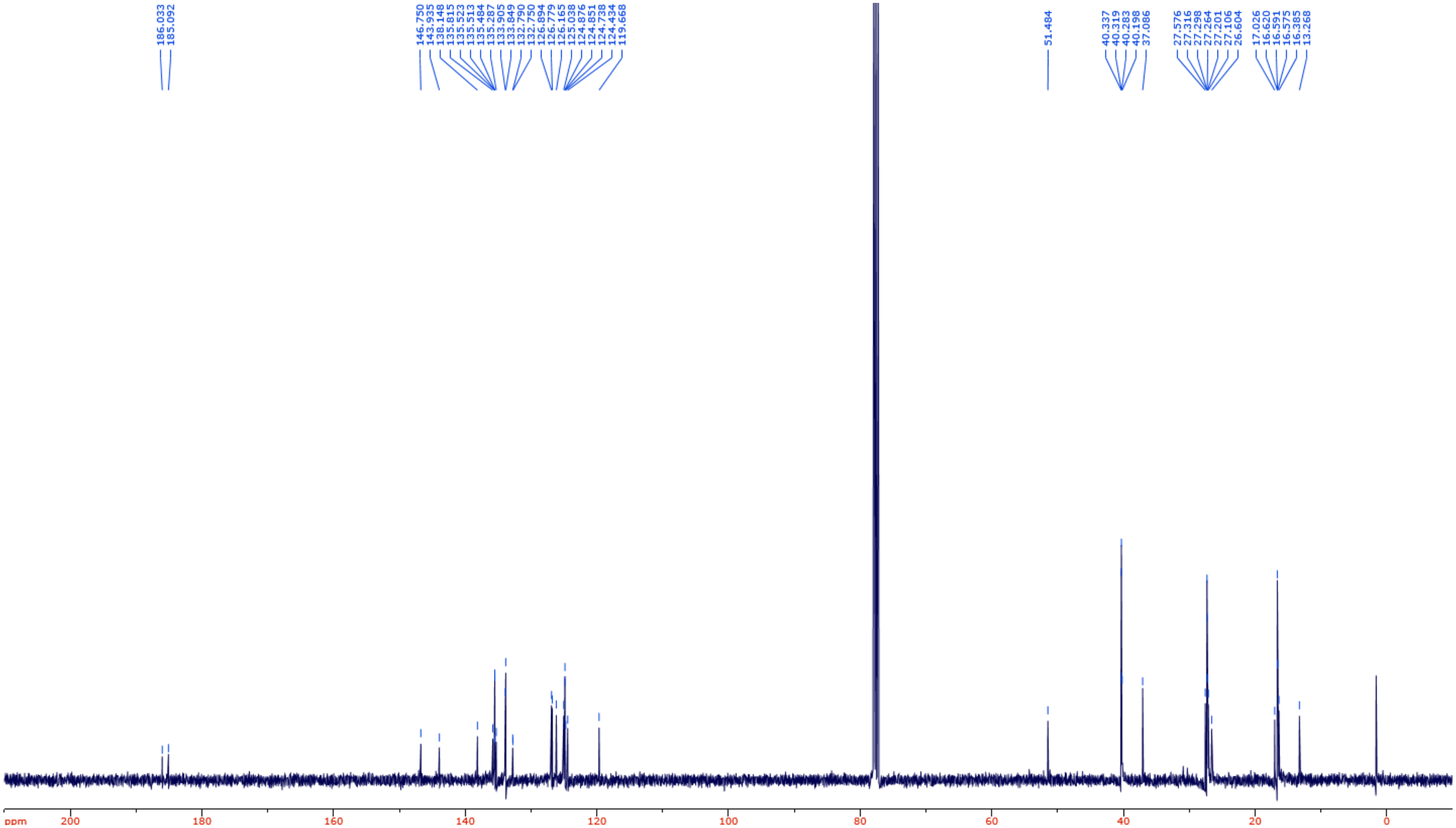

2D COSY (400 MHZ, $CDCl_3$) for MK9-ω-azide (29)

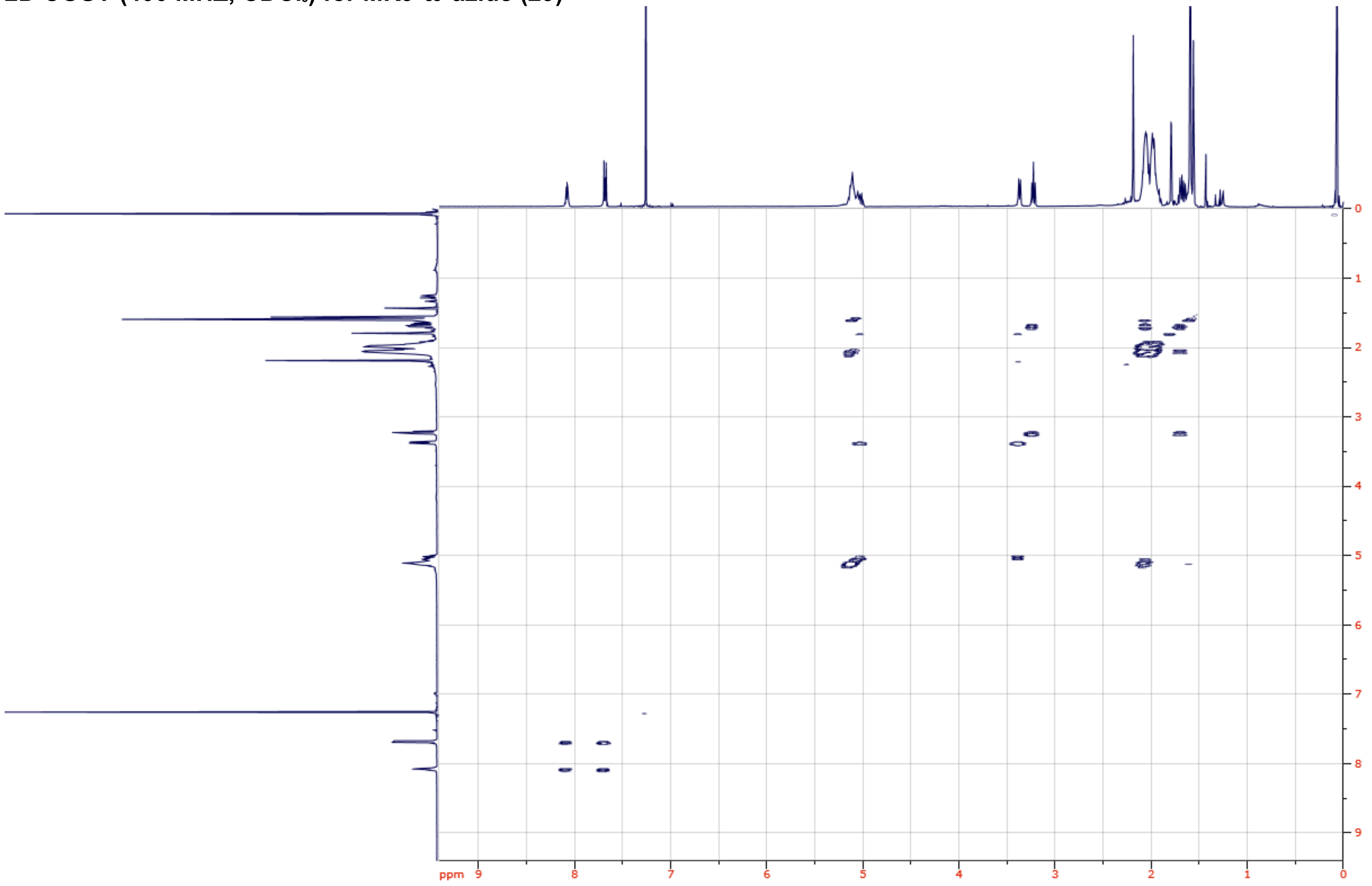

2D HSQC (400 MHZ, $CDCl_3$) for MK9-ω-azide (29)

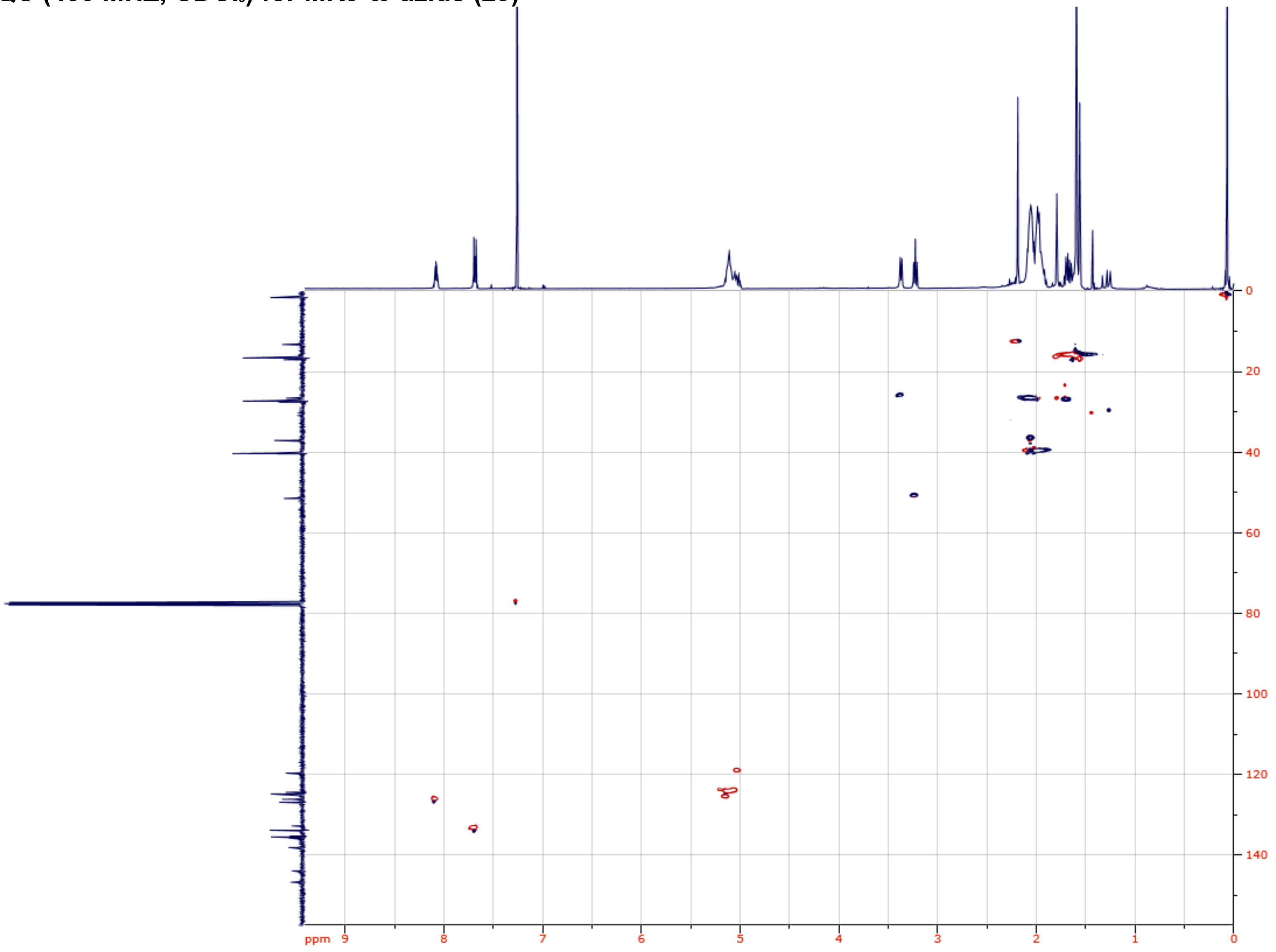

2D HSQC (400 MHZ, $CDCl_3$) for MK9-ω-azide (29)

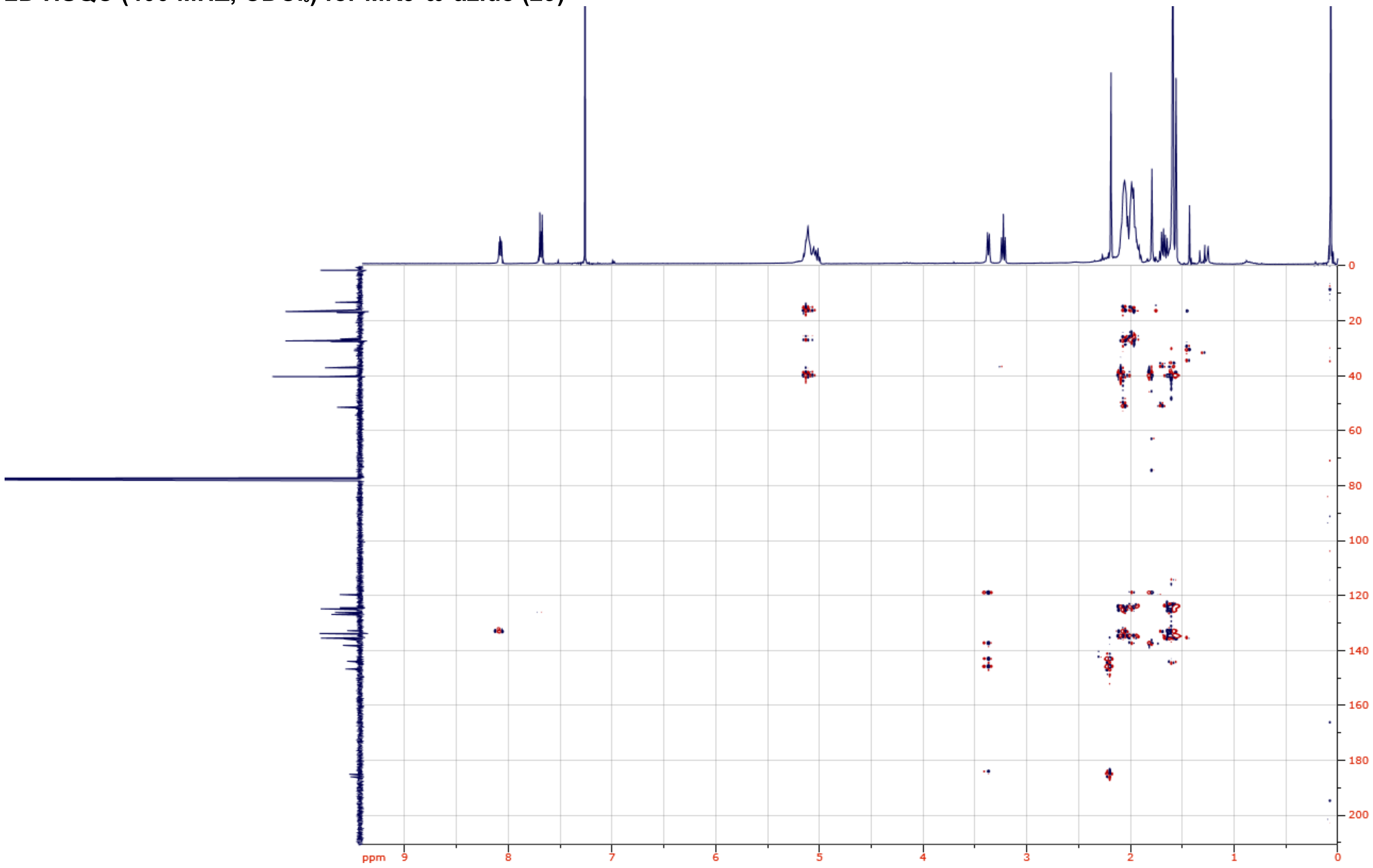

^{1}H NMR (400 MHZ, DMSO-d_6) for NBD-alkyne (39)

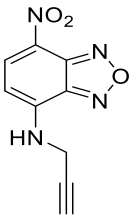

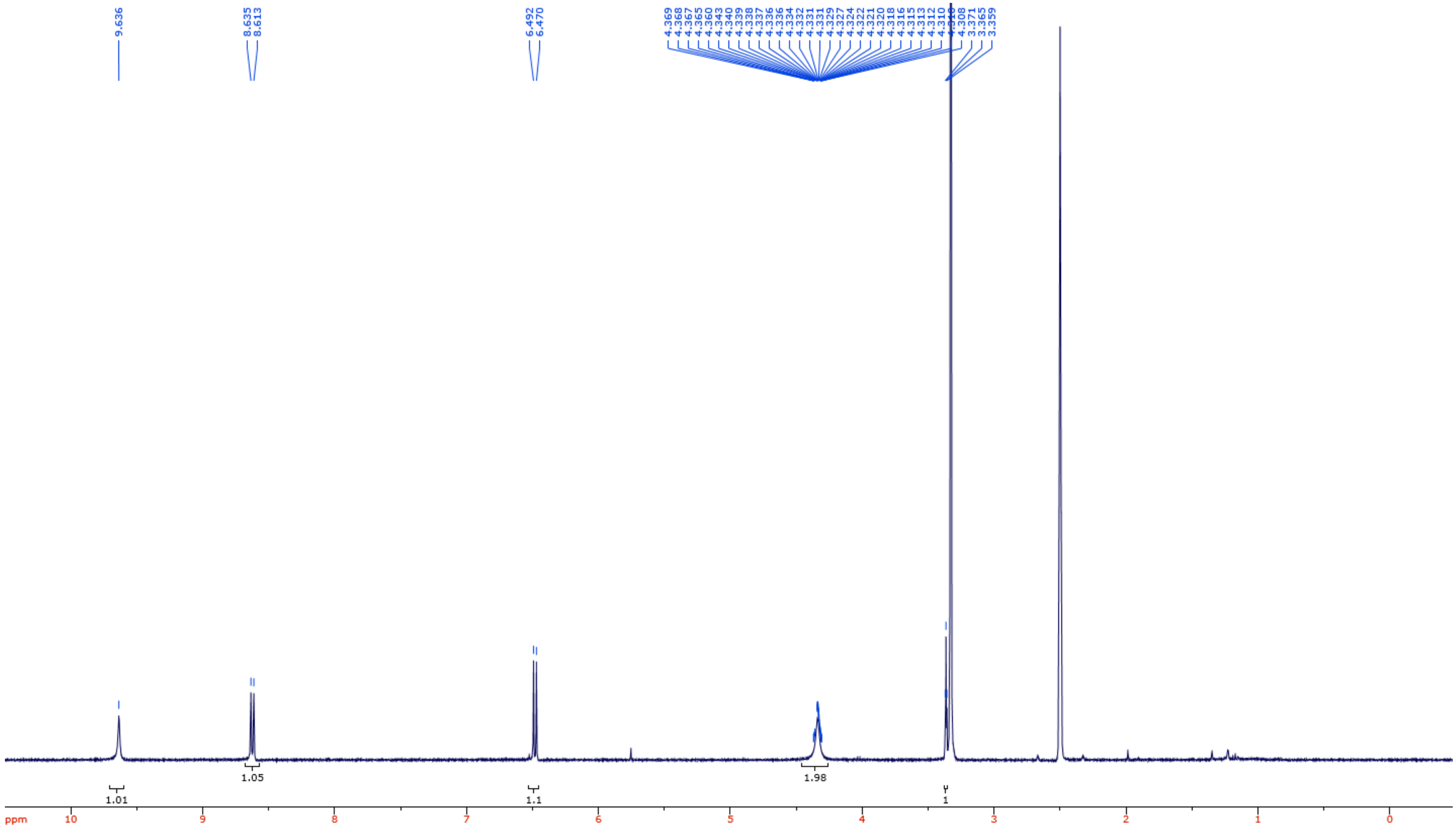

^{13}C NMR (101 MHZ, DMSO-d_6) for for NBD-alkyne (39)

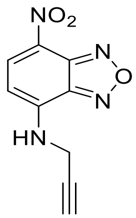

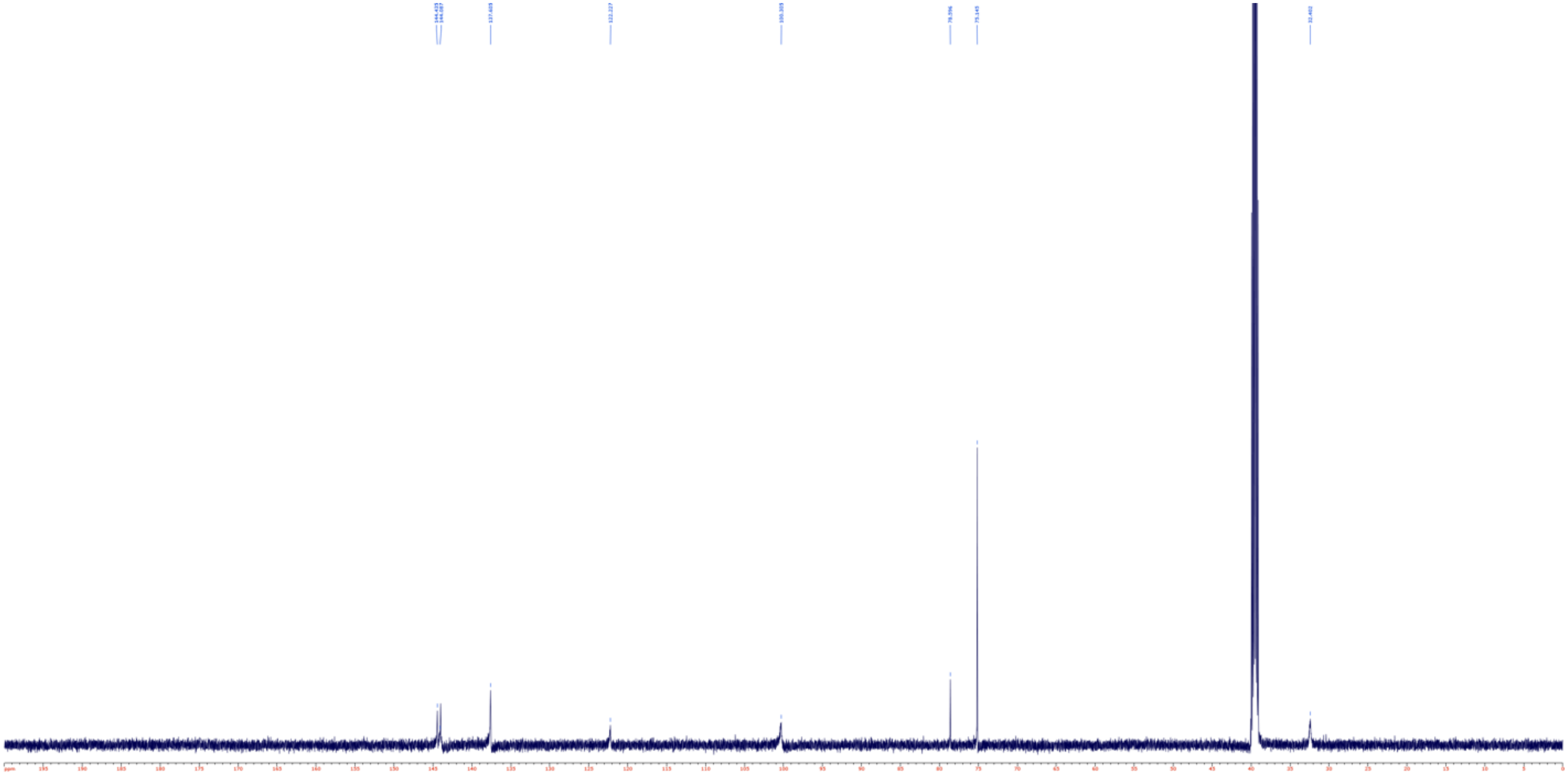

^{1}H NMR (400 MHz, DMSO-d_6) for DNB-alkyne (40)

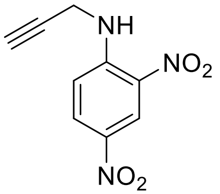

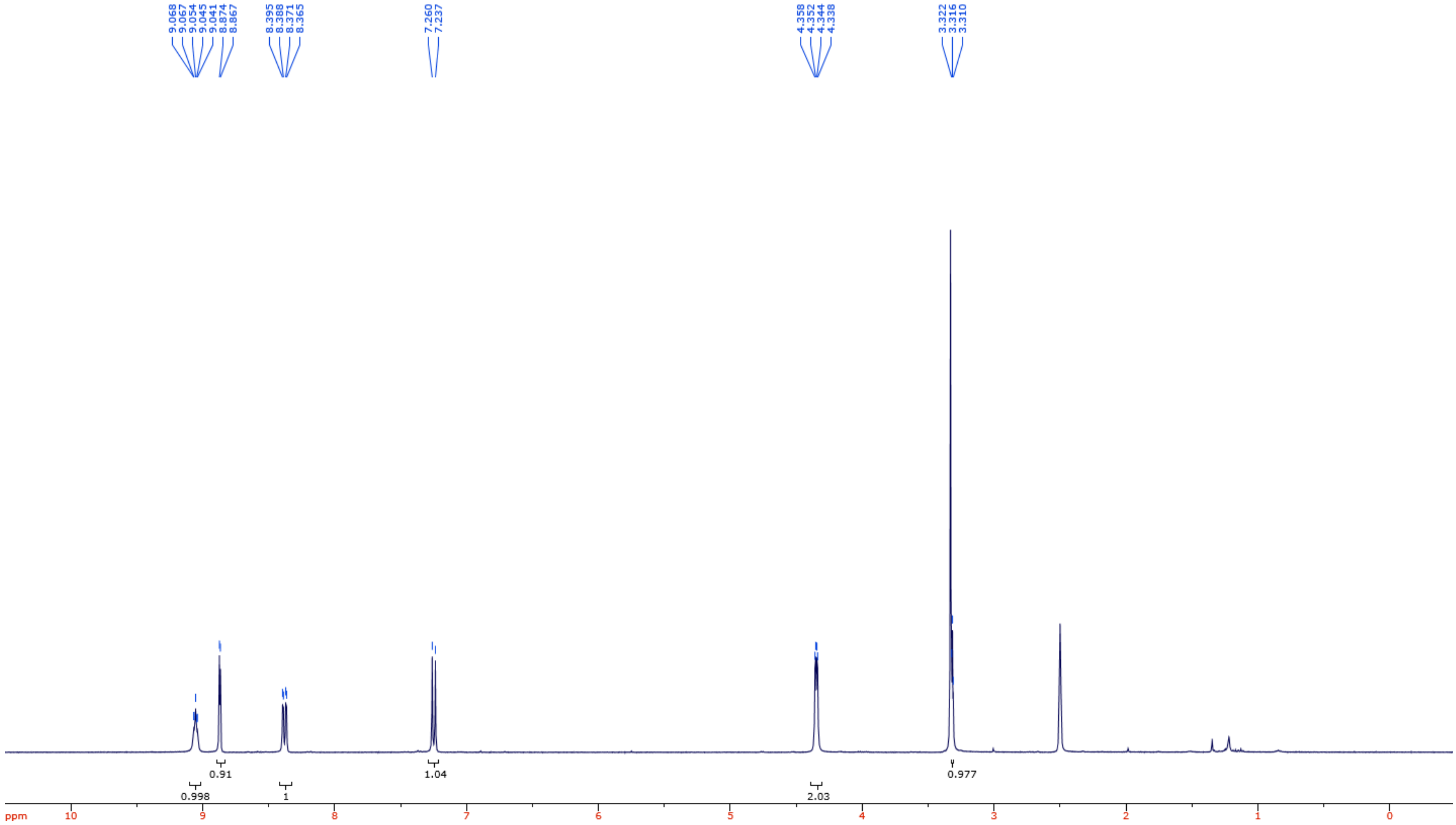

^{13}C NMR (101 MHz, DMSO-d$_6$) for DNB-alkyne (40)

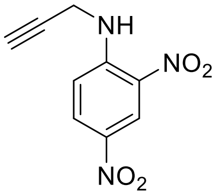

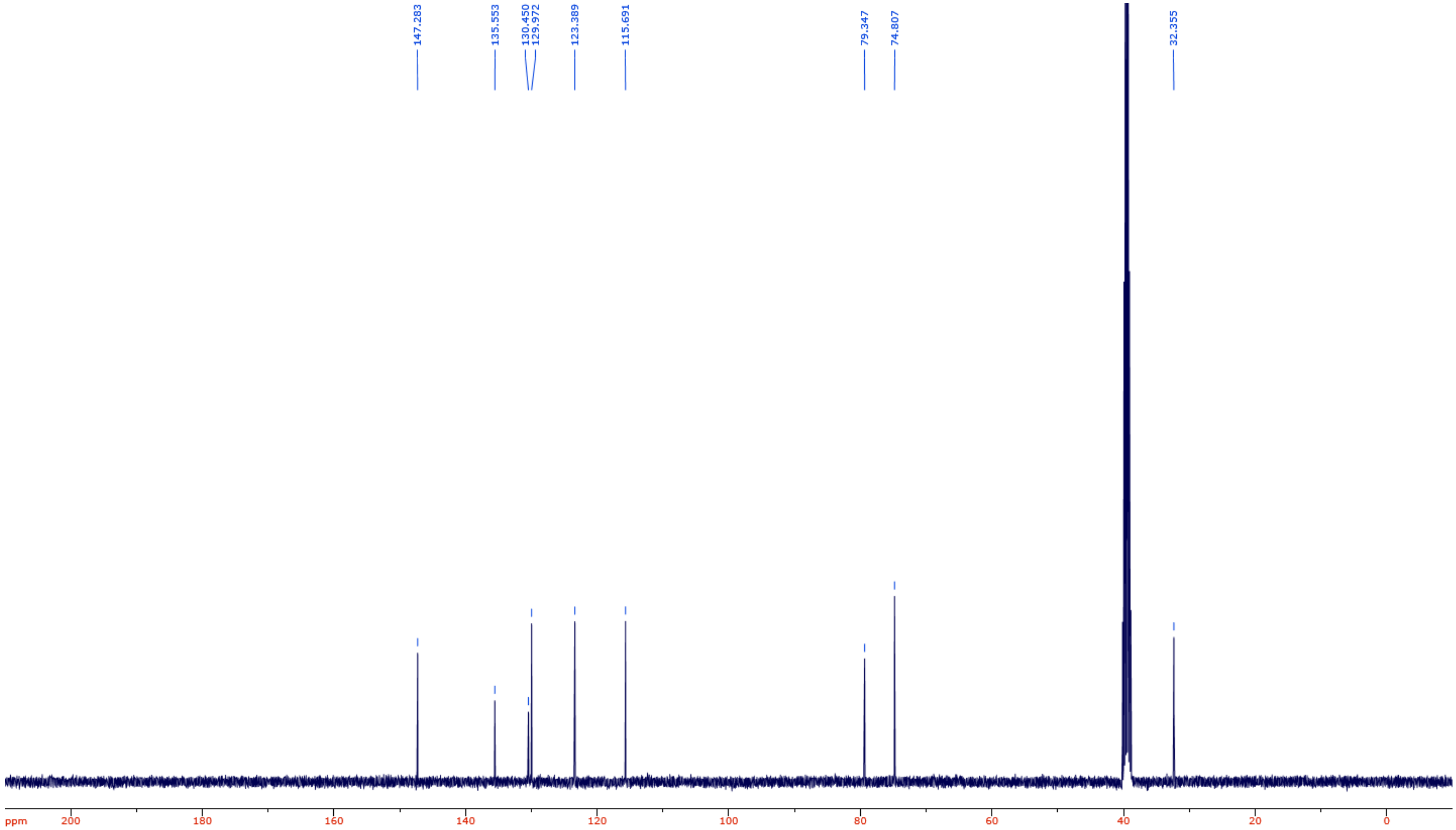

^{1}H NMR (400 MHz, DMSO-d_6) for benzyl (2E)-3-(1*H*-pyrrol-2-yl)prop-2-enoate (44)

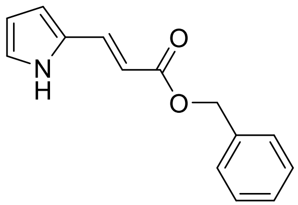

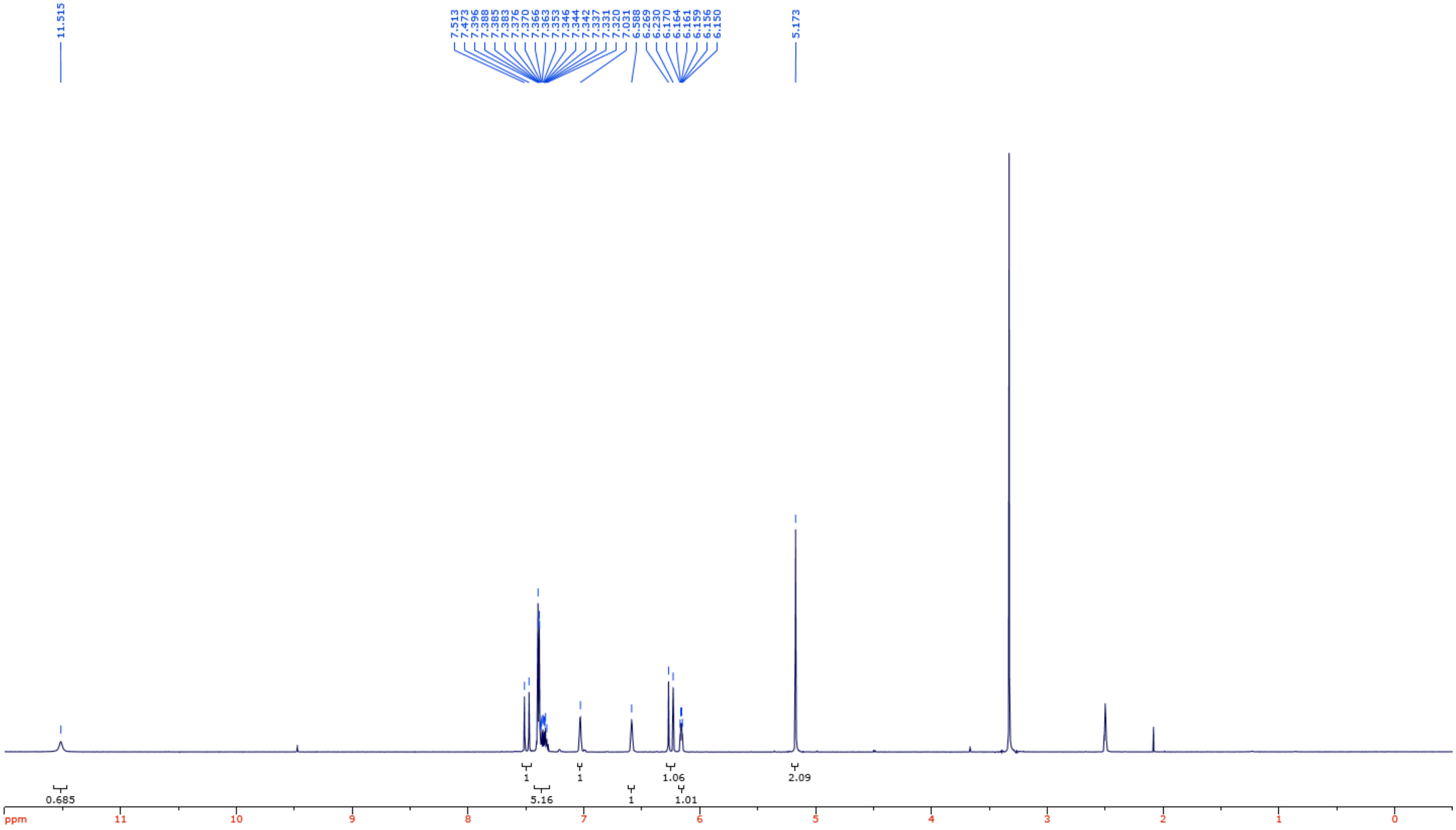

^{13}C NMR (101 MHz, DMSO-d_6) for benzyl (2E)-3-(1H-pyrrol-2-yl)prop-2-enoate (44)

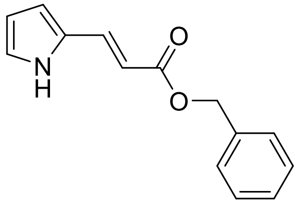

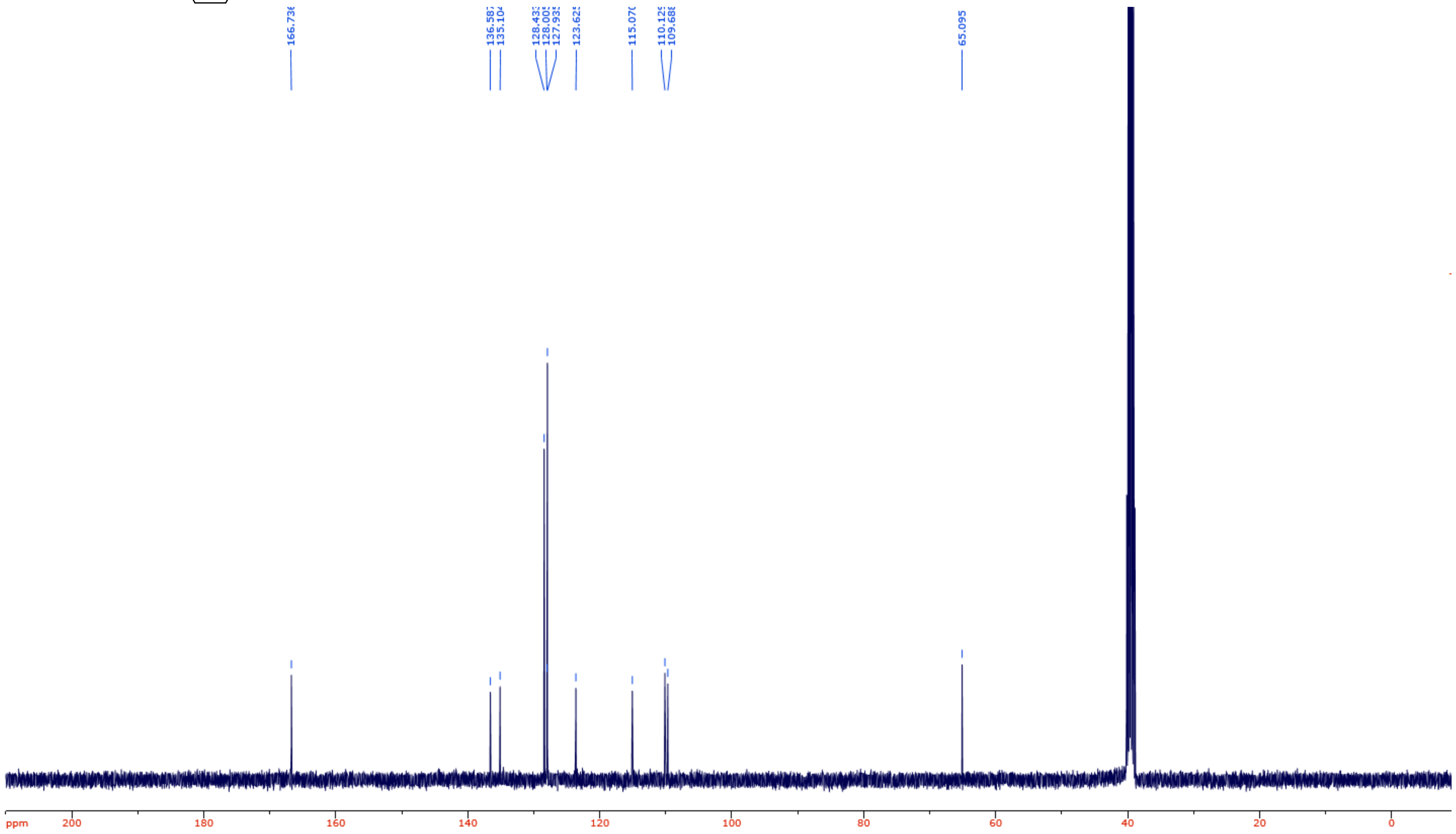

^{1}H NMR (400 MHz, DMSO-d$_6$) for benzyl 3-(1*H*-pyrrol-2-yl)propanoate (45)

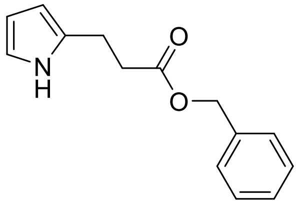

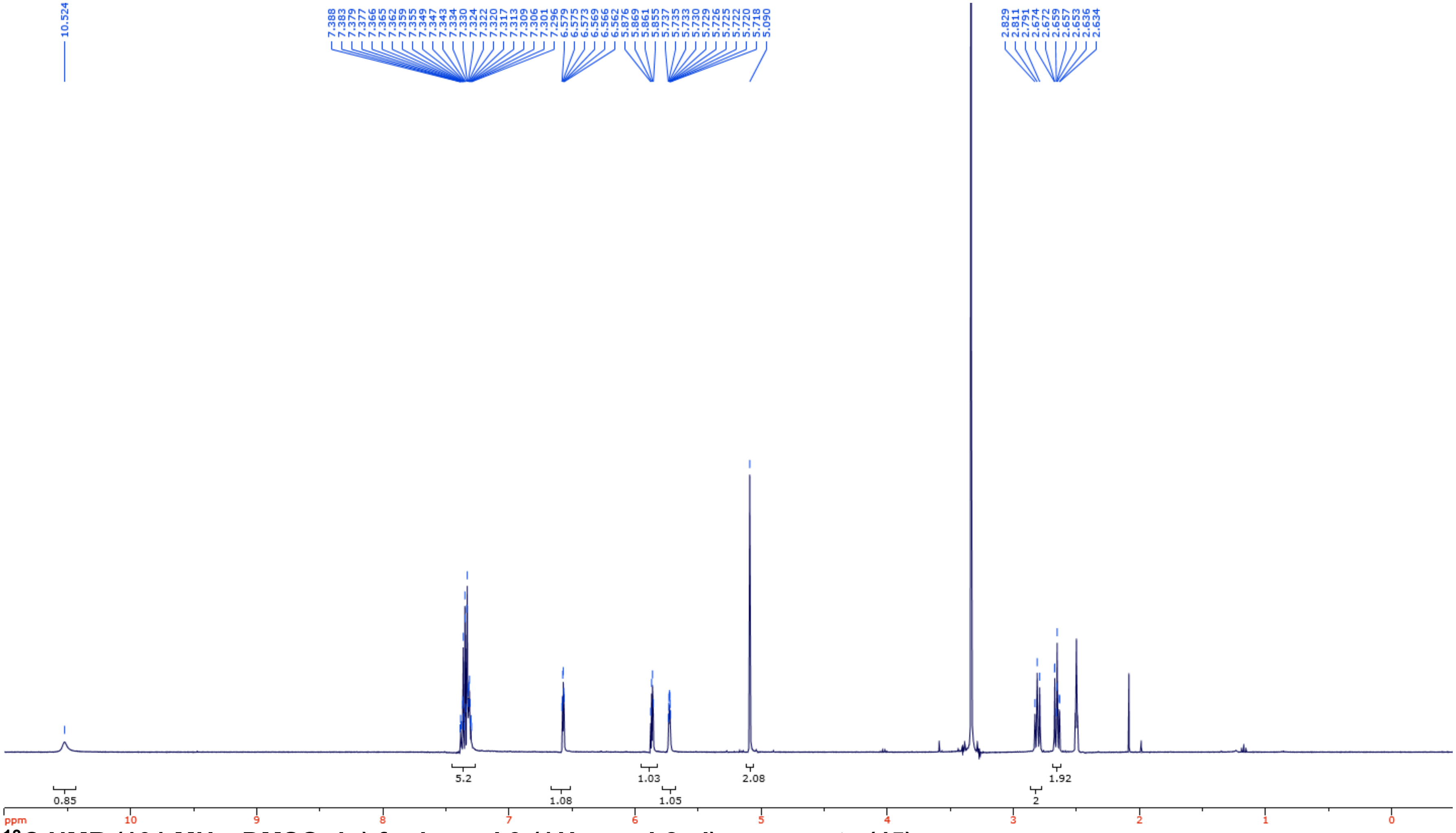

^{13}C NMR (101 MHz, DMSO-d$_6$) for benzyl 3-(1*H*-pyrrol-2-yl)propanoate (45)

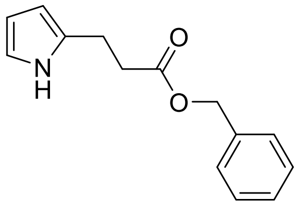

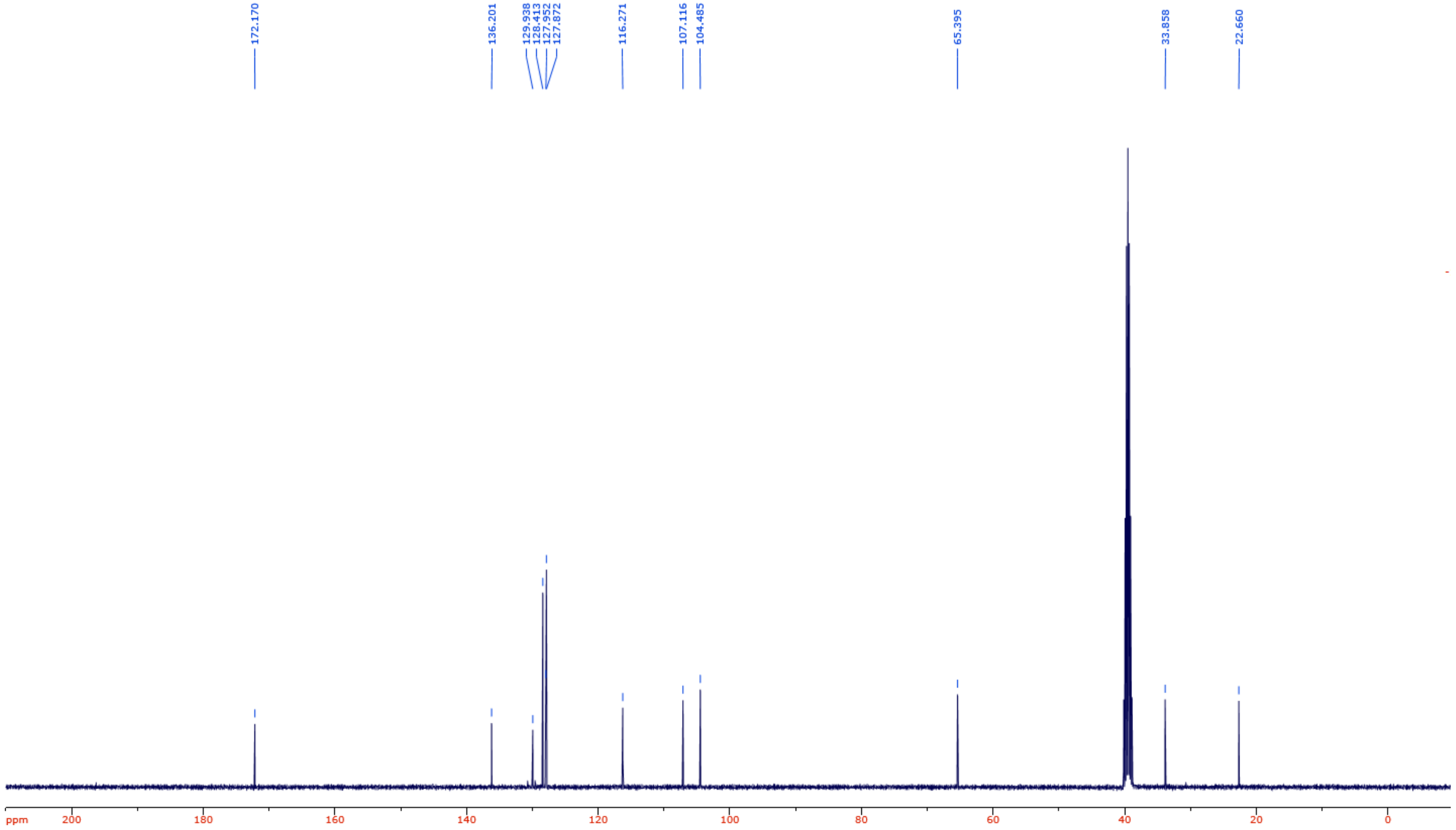

^{1}H NMR (400 MHz, DMSO-d$_6$) for BODIPY acid (47)

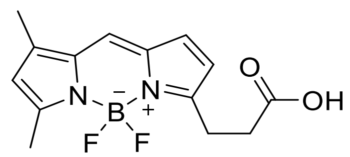

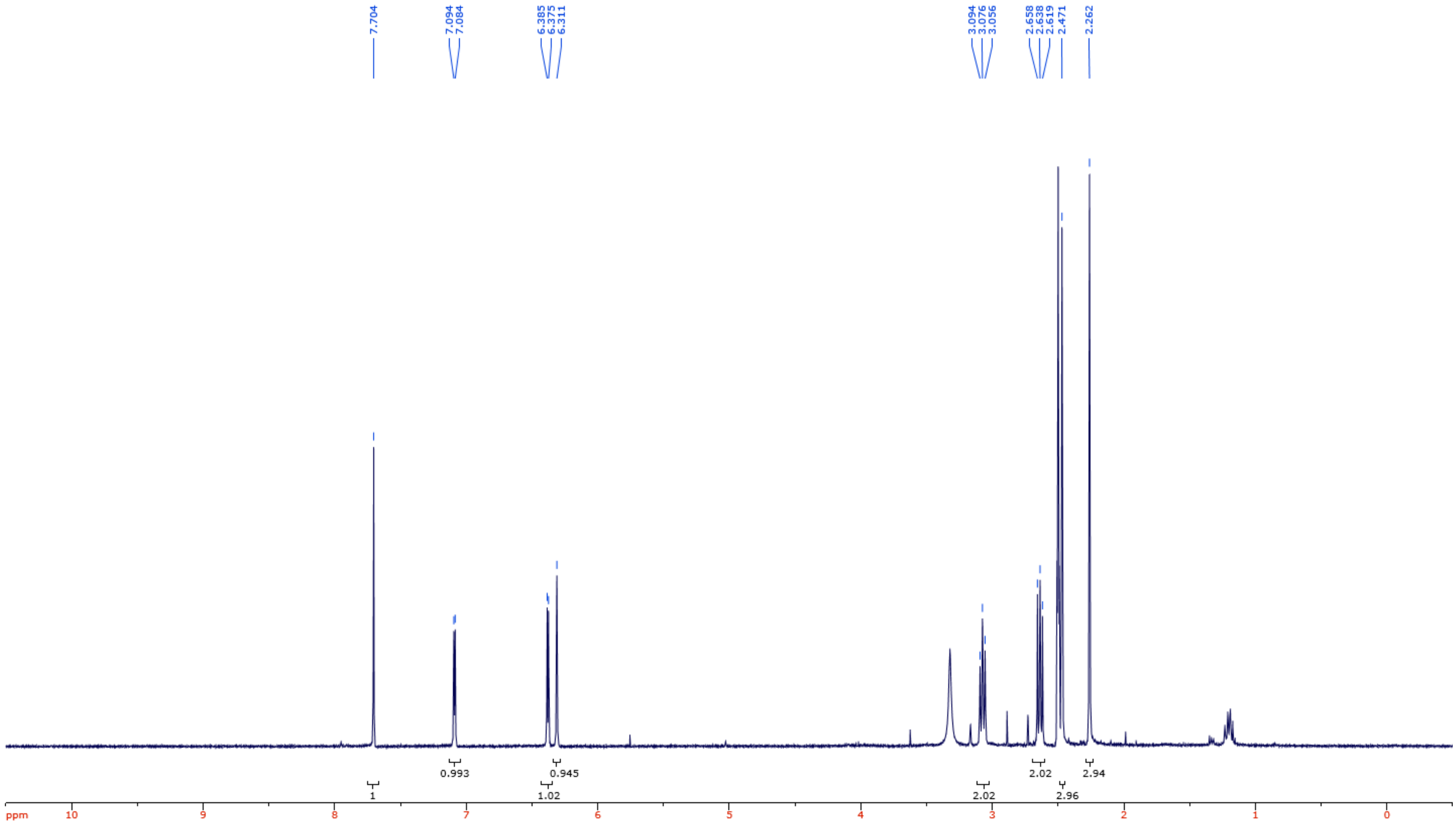

^{13}C NMR (101 MHz, DMSO-d_6) for BODIPY acid (47)

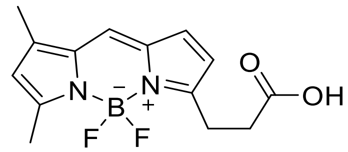

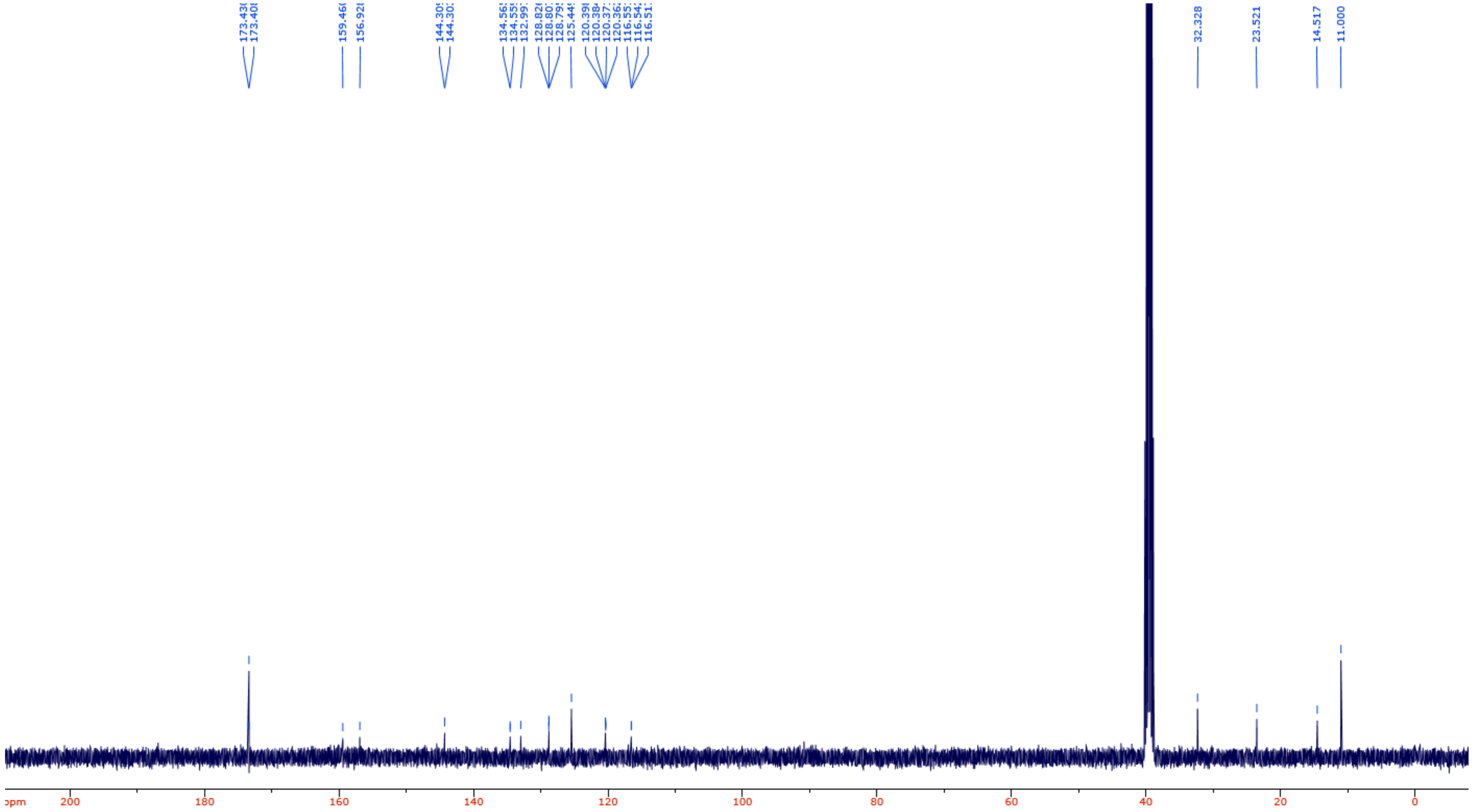

^{1}H NMR (400 MHz, $CDCl_3$) for BODIPY-alkyne (41)

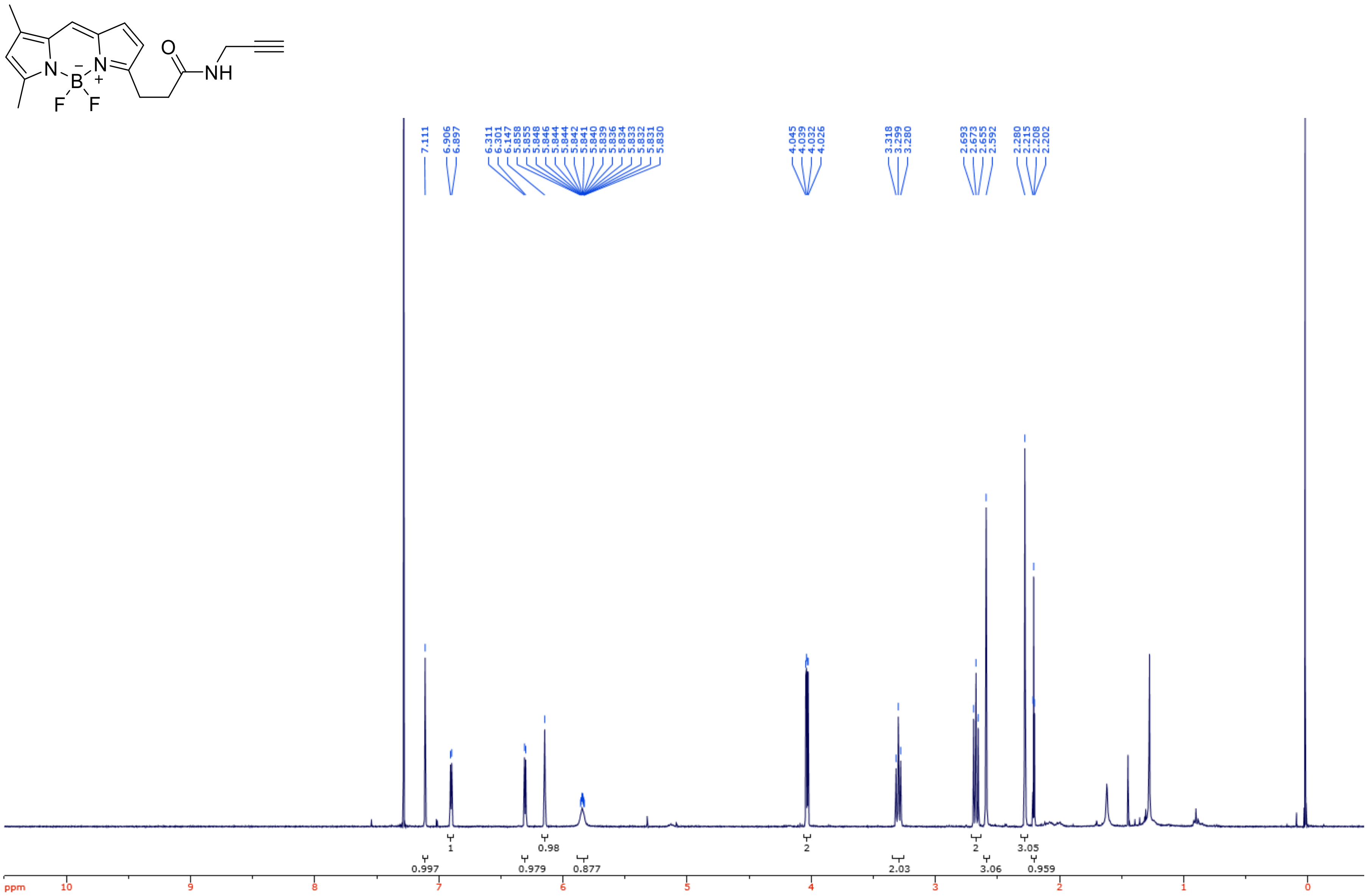
O
NH
N
B
N
F
F
7.111
6.906
6.897
6.311
6.301
6.147
5.858
5.855
5.848
5.846
5.844
5.844
5.842
5.841
5.840
5.839
5.836
5.834
5.833
5.832
5.831
5.830
4.045
4.039
4.032
4.026
3.318
3.299
3.280
2.693
2.673
2.655
2.592
2.280
2.215
2.208
2.202
1
0.98
2
2
3.05
0.997
0.979
0.877
2.03
3.06
0.959
ppm
10
9
8
7
6
5
4
3
2
1
0

^{13}C NMR (101 MHz, $CDCl_3$) for BODIPY-alkyne (41)

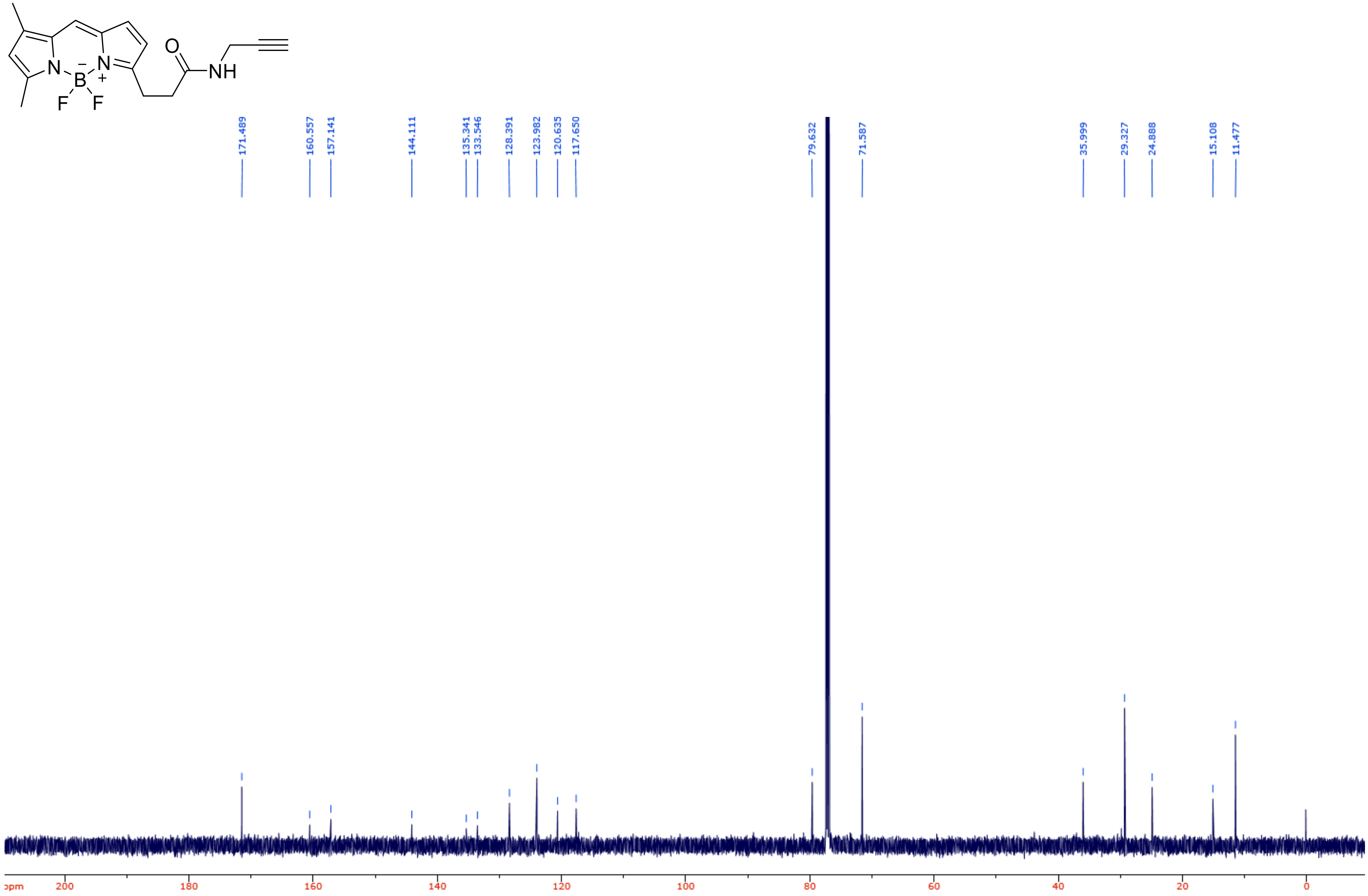

2D COSY NMR (400 MHz, $CDCl_3$) for BODIPY-alkyne (41)

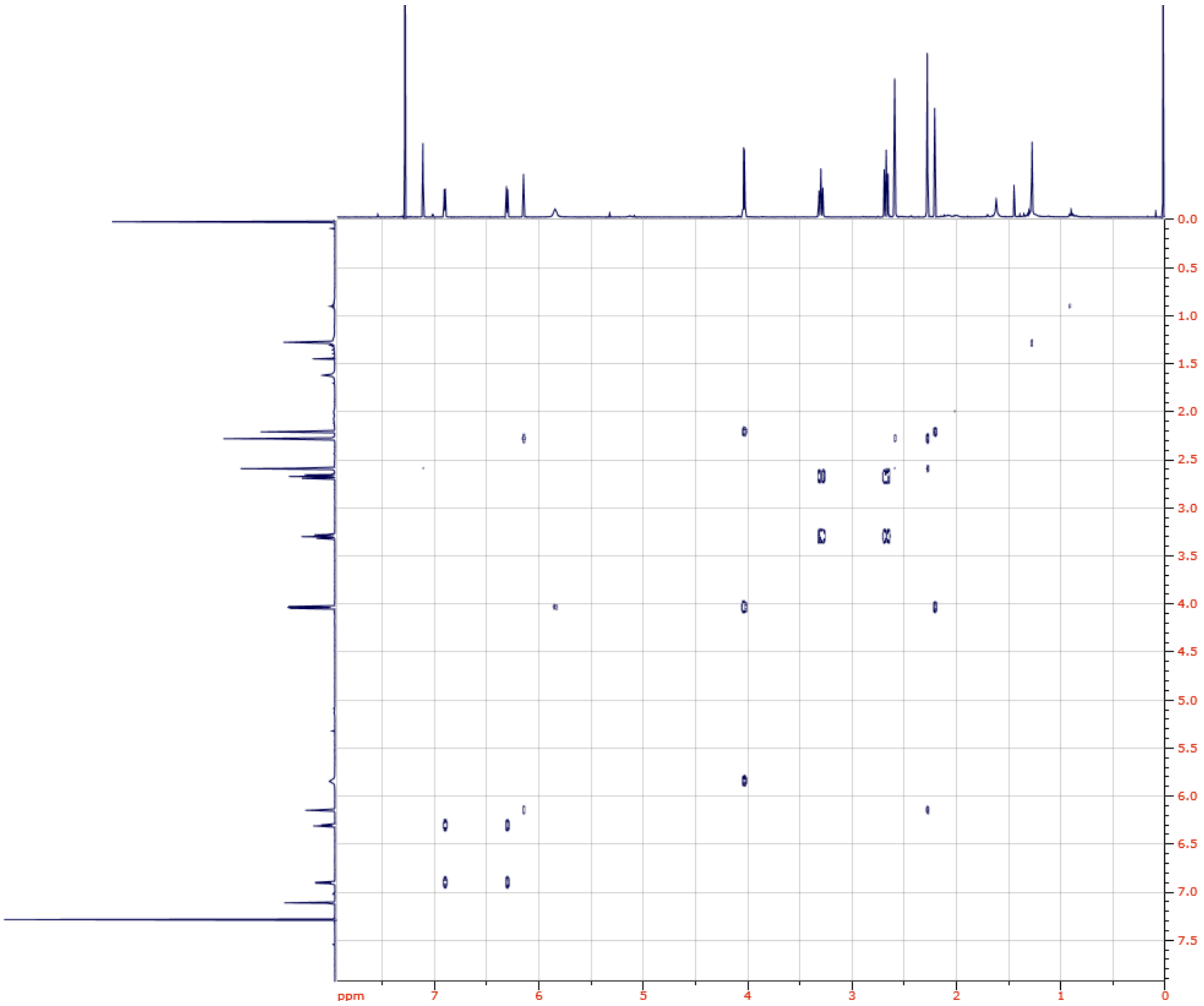

2D HSQC NMR (400 MHz, $CDCl_3$) for BODIPY-alkyne (41)

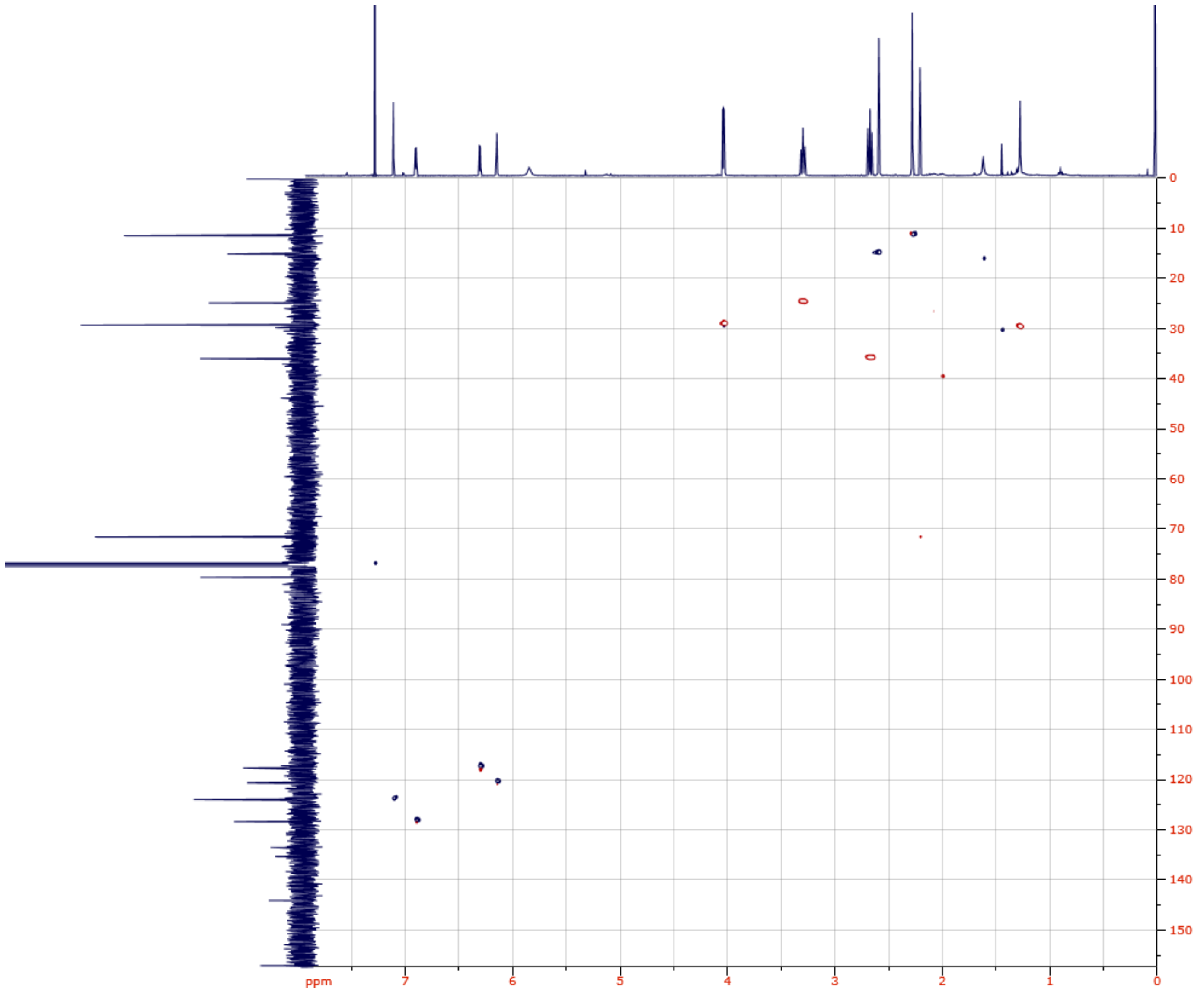

2D HMBC NMR (400 MHz, $CDCl_3$) for BODIPY-alkyne (41)

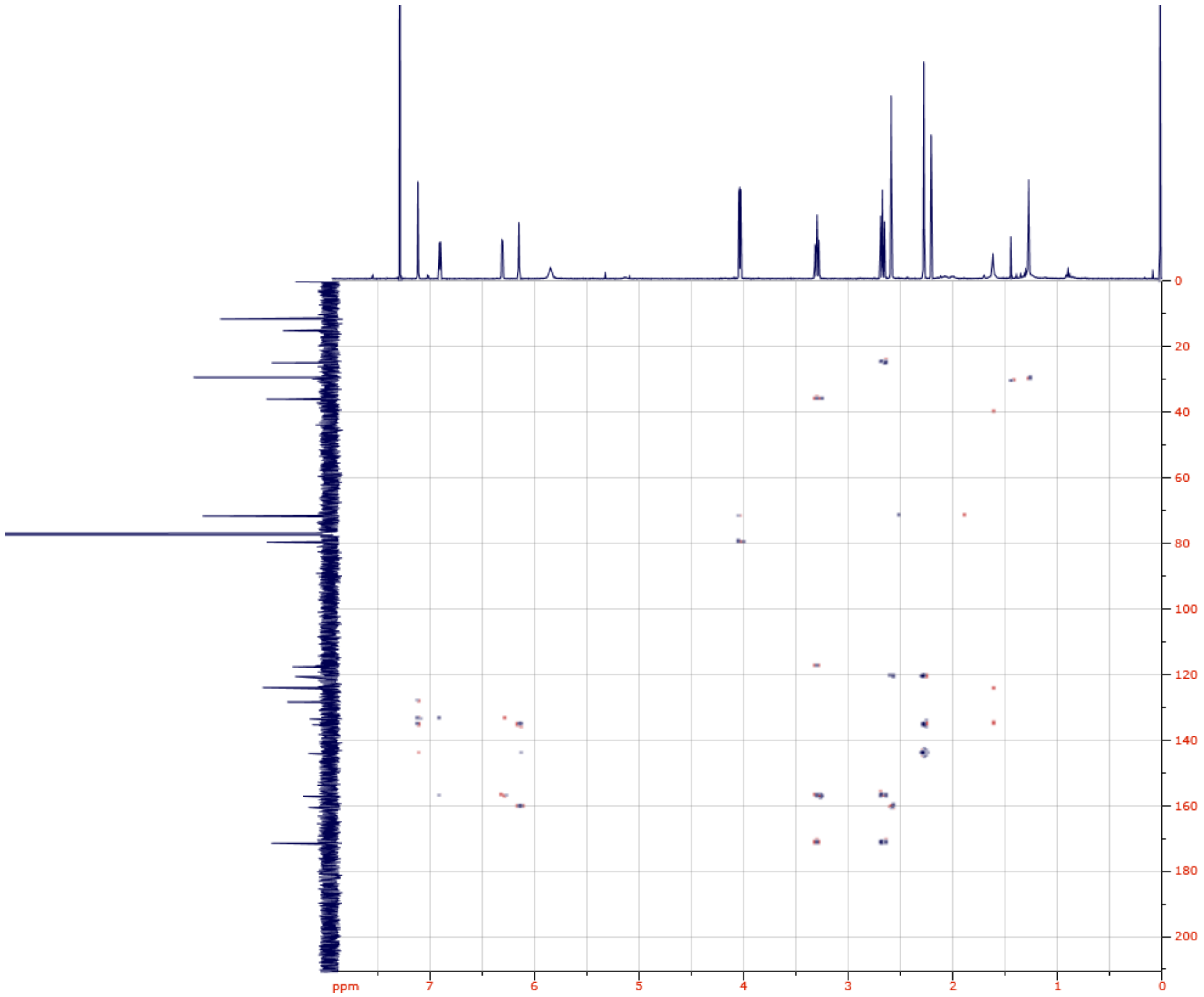

^{19}F NMR (376 MHz, $CDCl_3$) for BODIPY-alkyne (41)

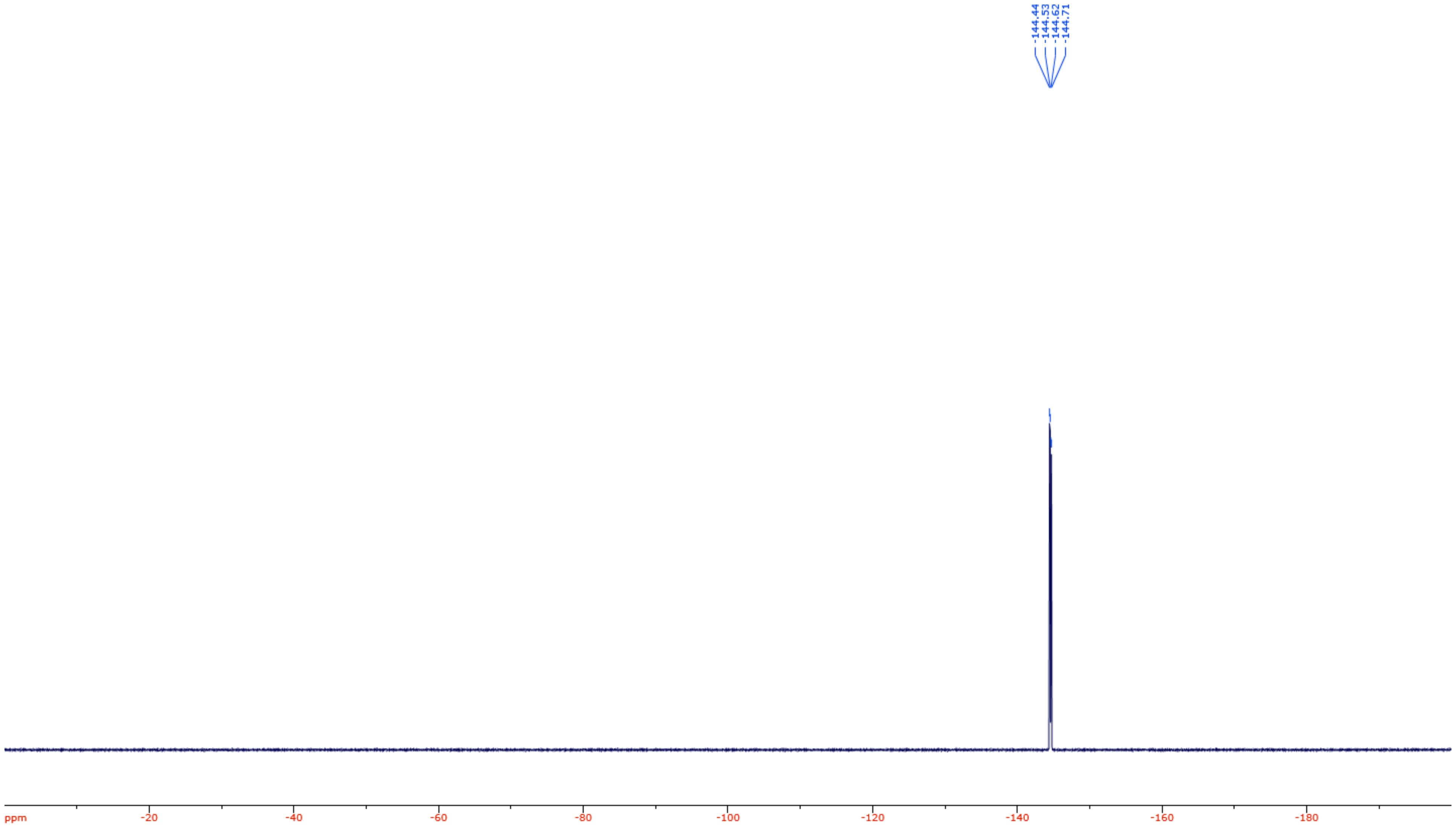
-144.44
-144.53
-144.62
-144.71
ppm
-20
-40
-60
-80
-100
-120
-140
-160
-180

^{1}H NMR (400 MHZ, DMSO-d_6) for Biotin-NHS ester (49)

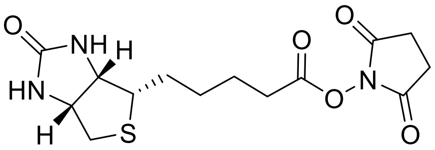

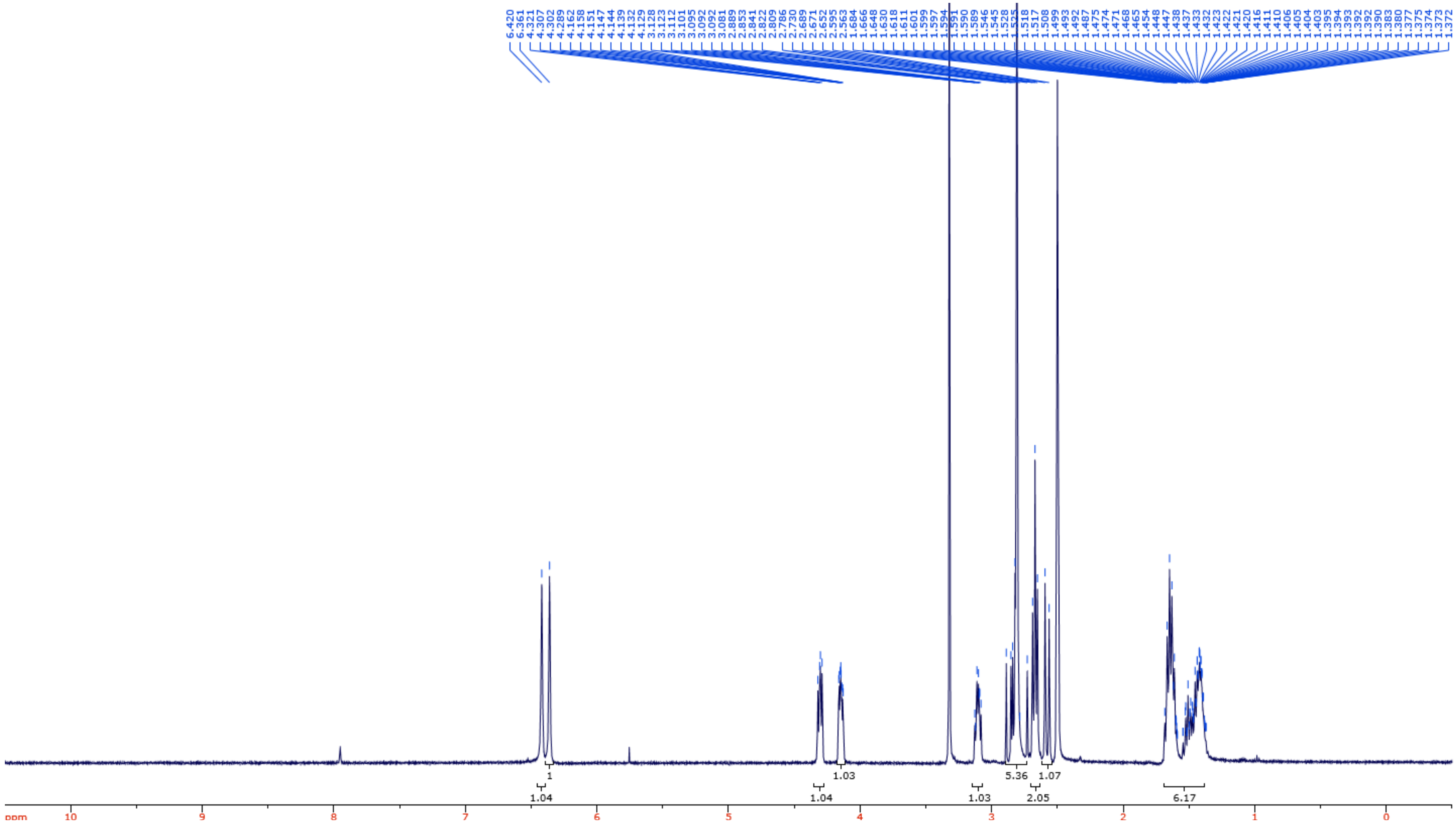

^{13}C NMR (101 MHZ, DMSO-d$_6$) for Biotin-NHS ester (49)

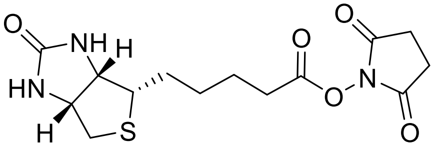

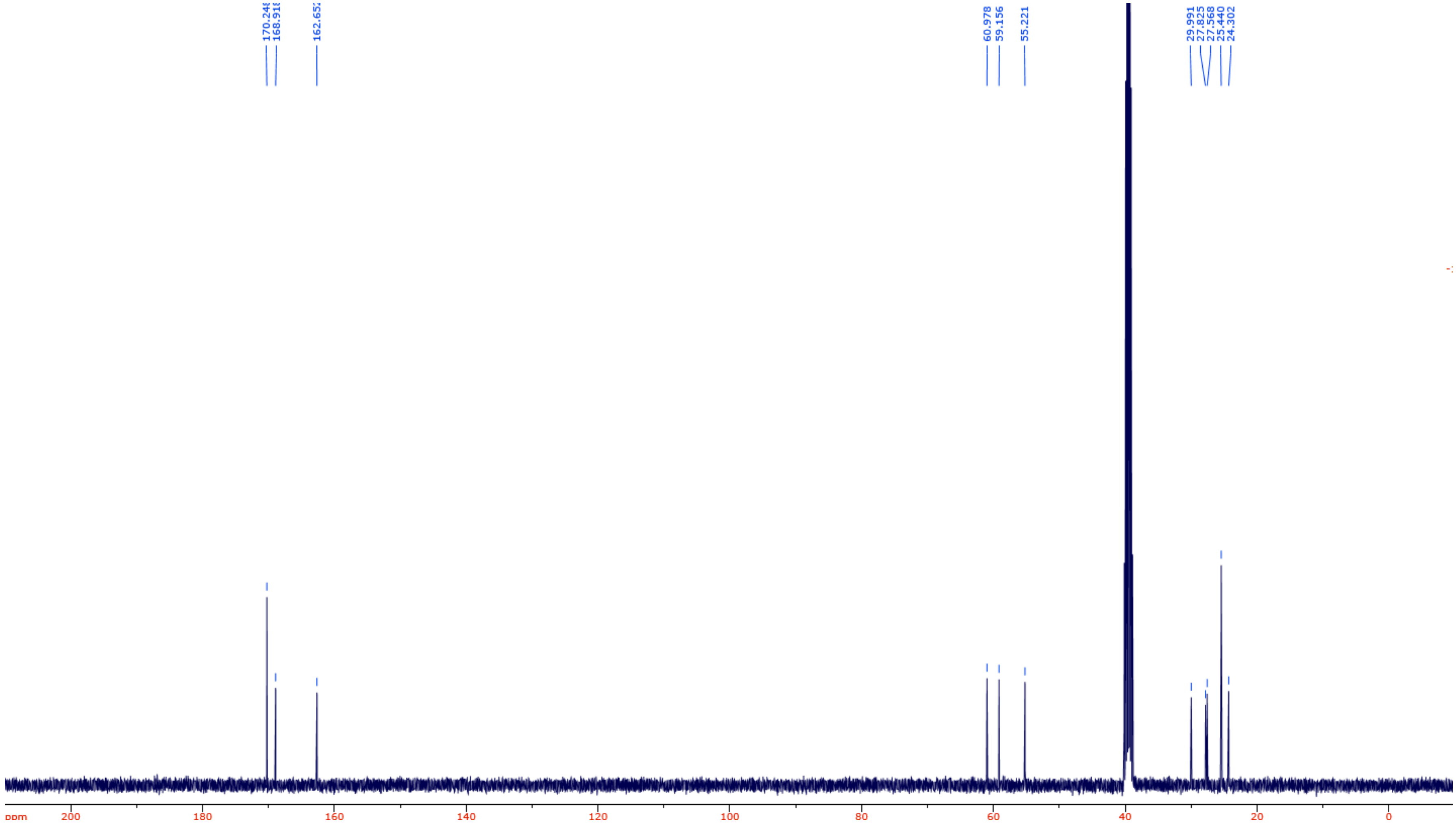

^{1}H NMR (400 MHZ, DMSO-d_6) for biotin alkyne (48)

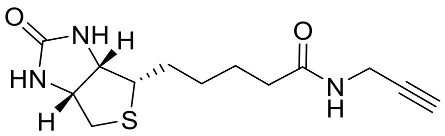

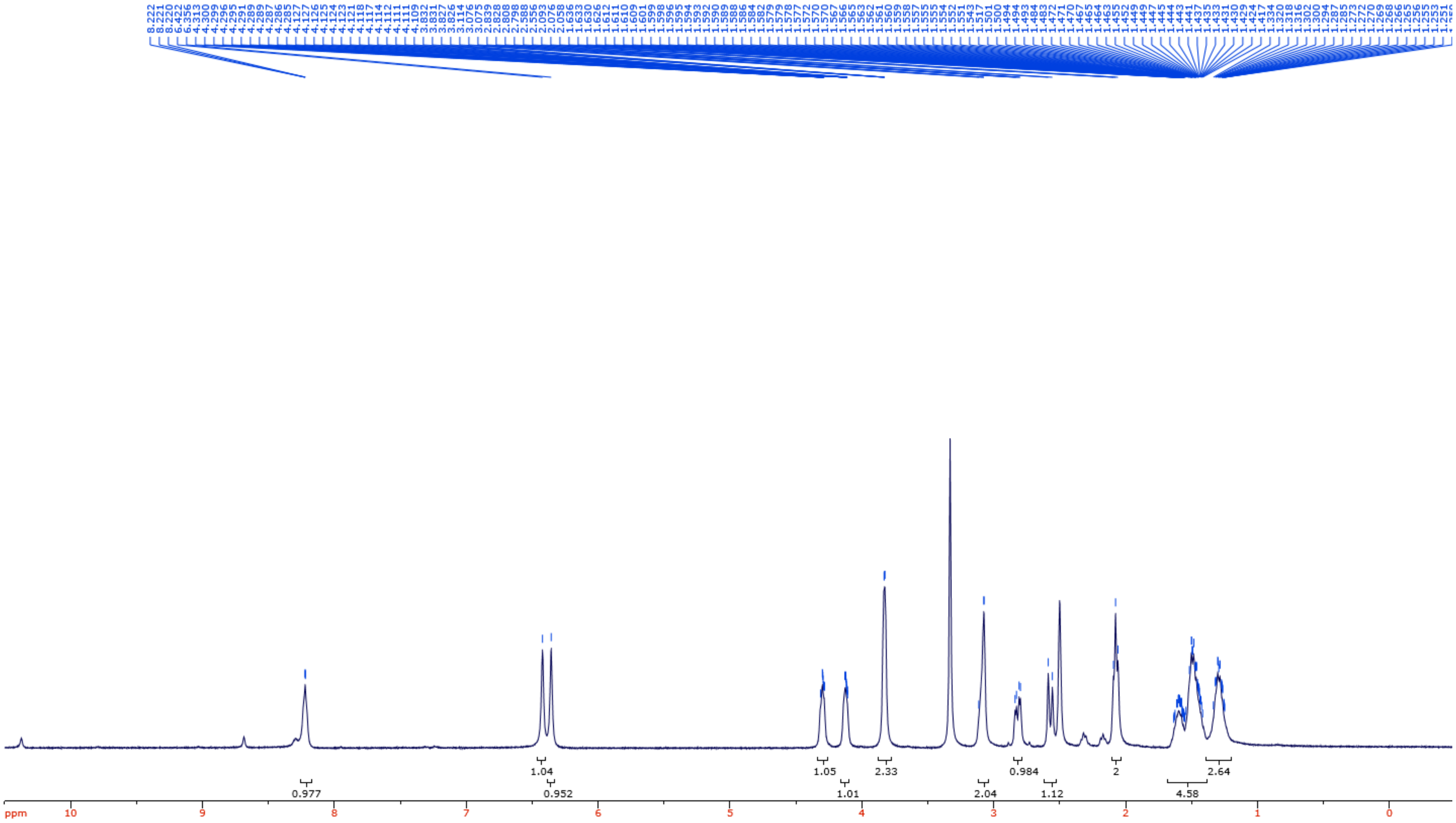

^{13}C NMR (101 MHZ, DMSO-d_6) for biotin alkyne (48)

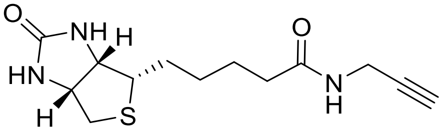

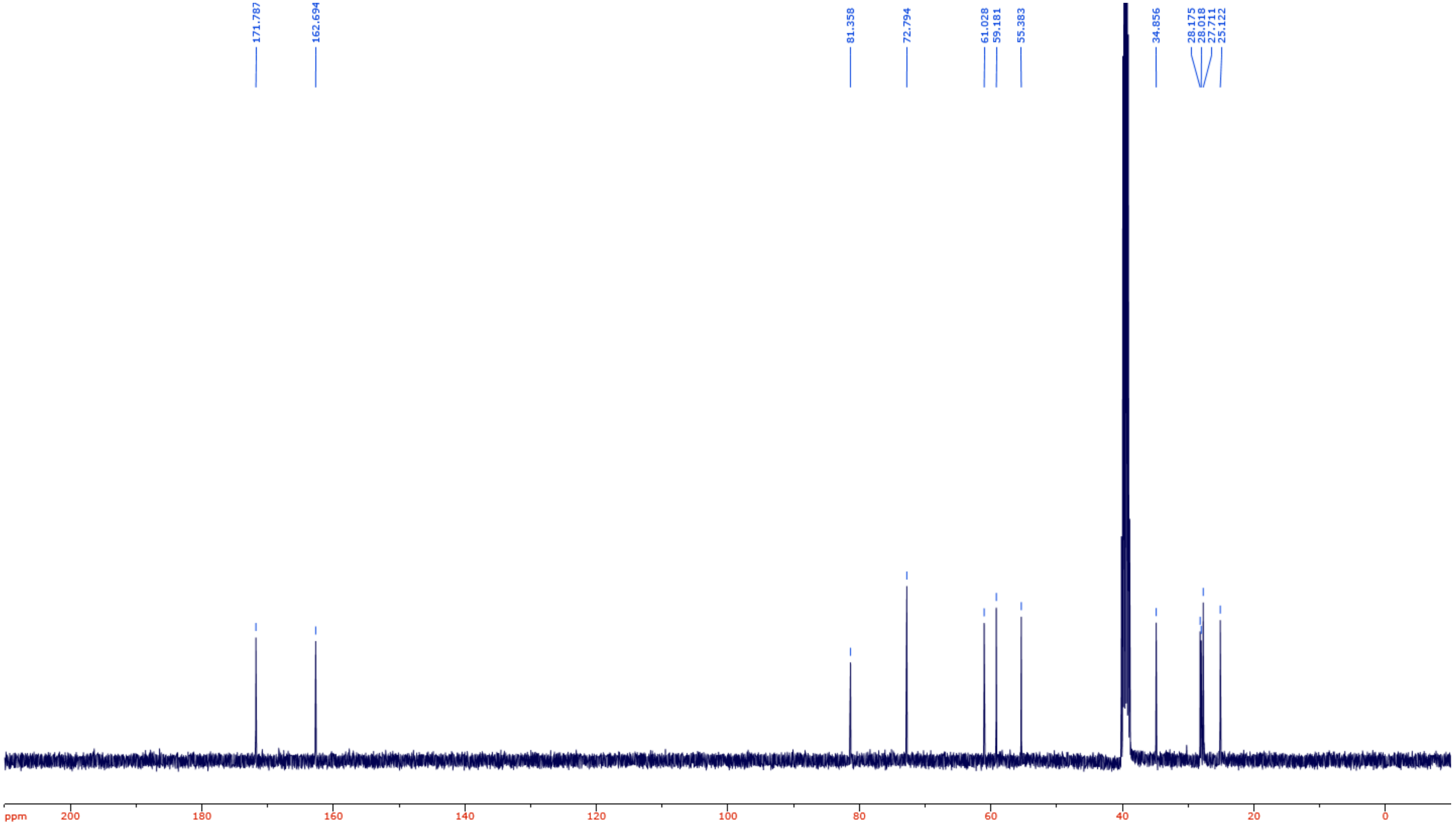

^{1}H NMR (400 MHz, $CDCl_3$) for CoQ$_{10}$-ω-NBD (30)

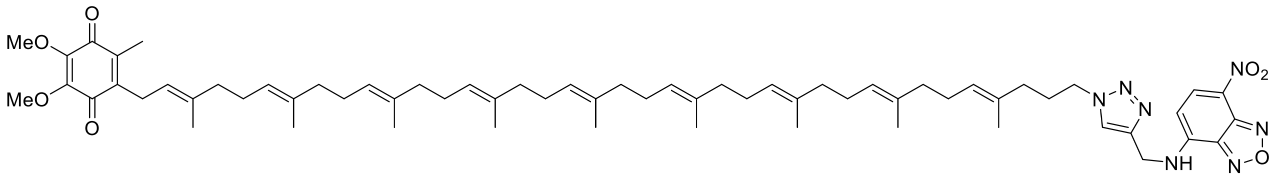

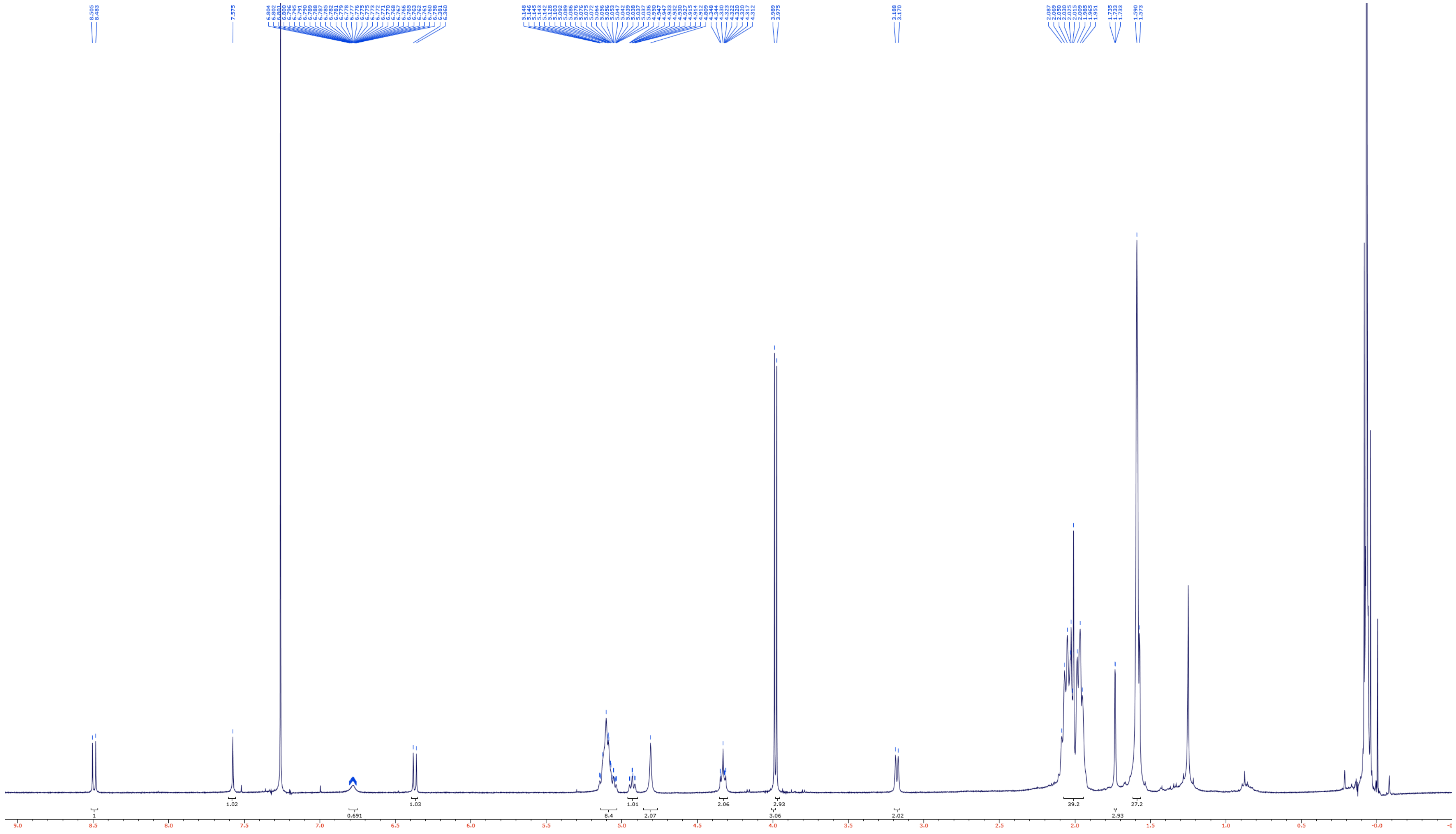

^{13}C NMR (400 MHz, $CDCl_3$) for CoQ_{10}-ω-NBD (30)

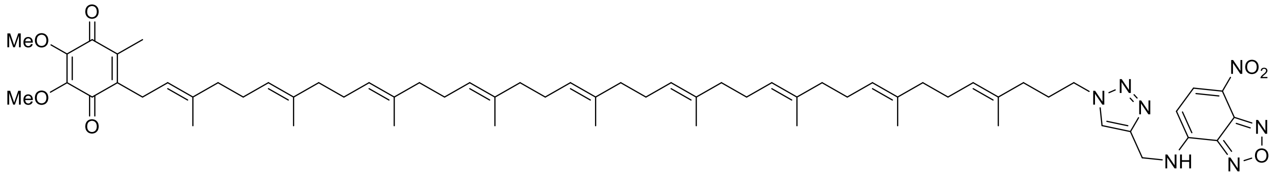

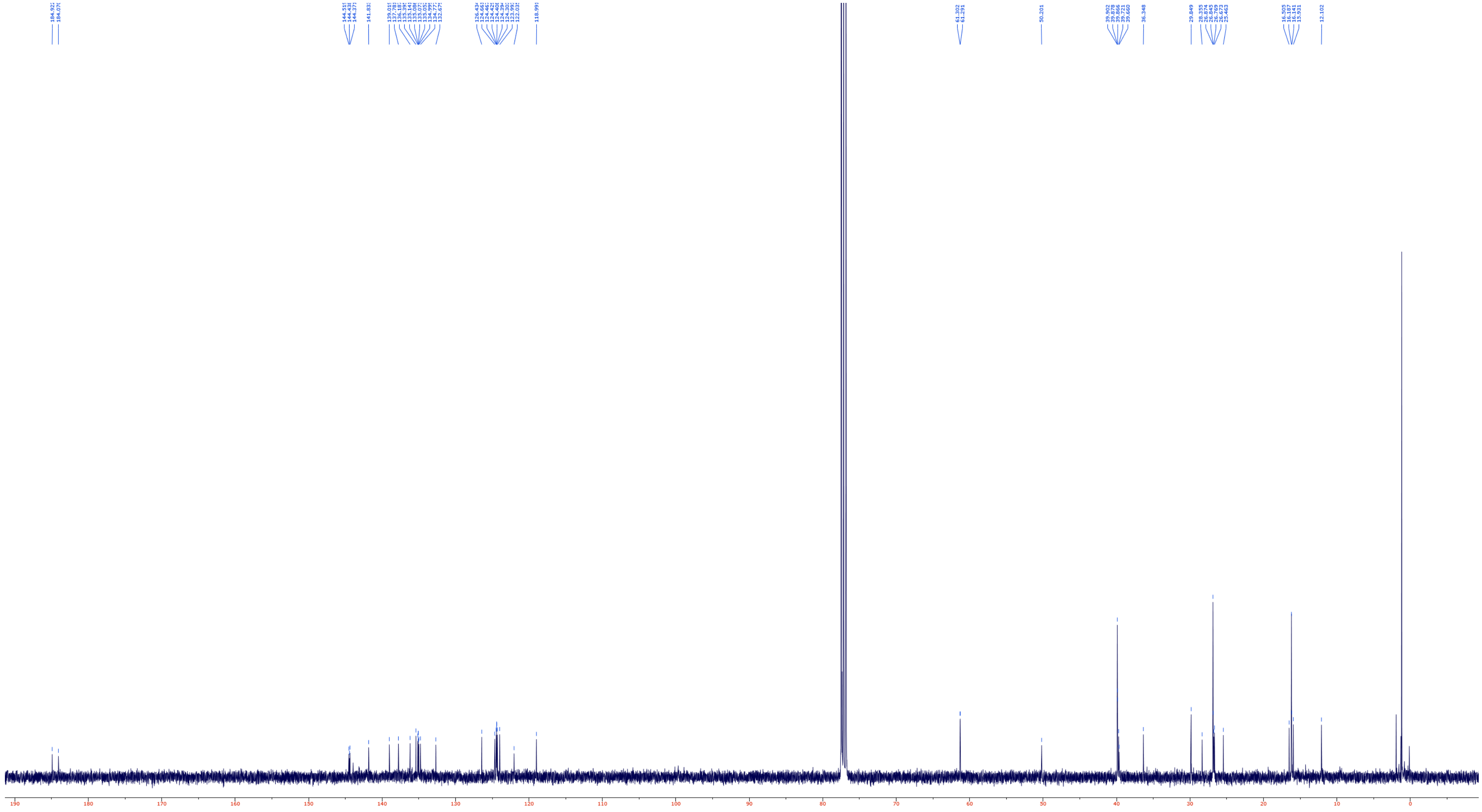

2D COSY (400 MHz, $CDCl_3$) for CoQ_{10}-ω-NBD (30)

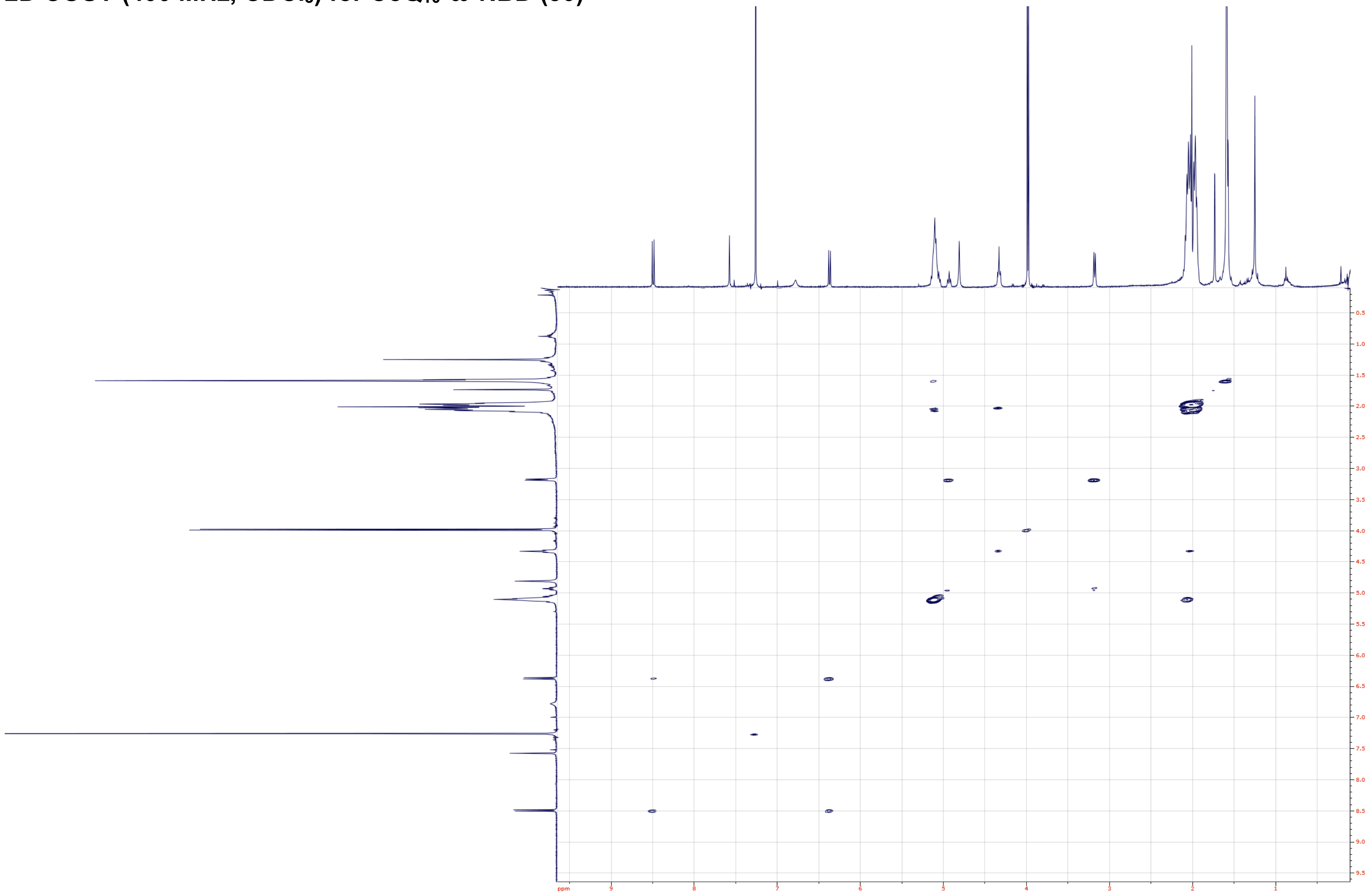

2D HSQC (400 MHz, $CDCl_3$) for CoQ_{10}-ω-NBD (30)

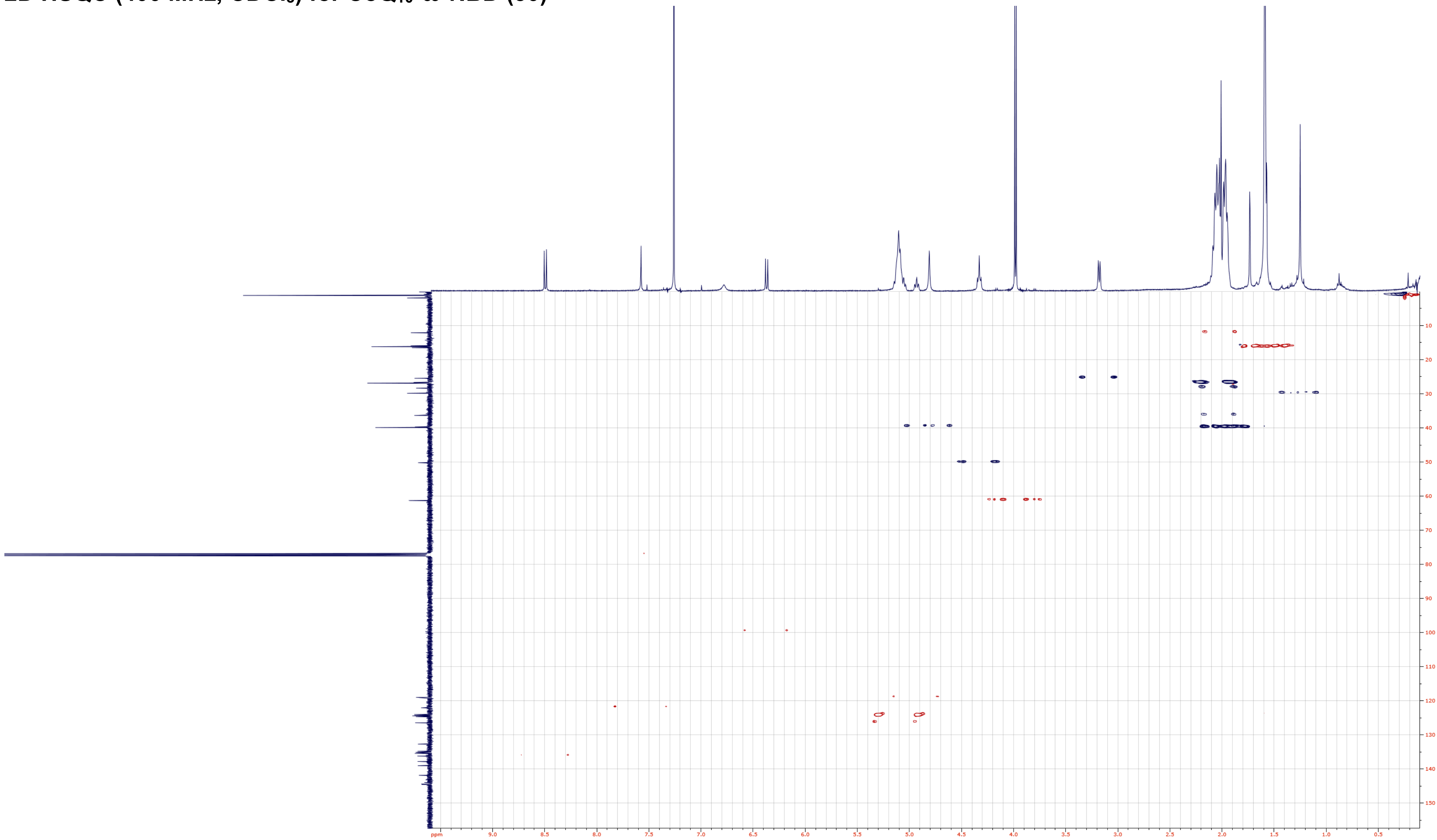

2D HMBC (400 MHz, $CDCl_3$) for CoQ_{10}-ω-NBD (30)

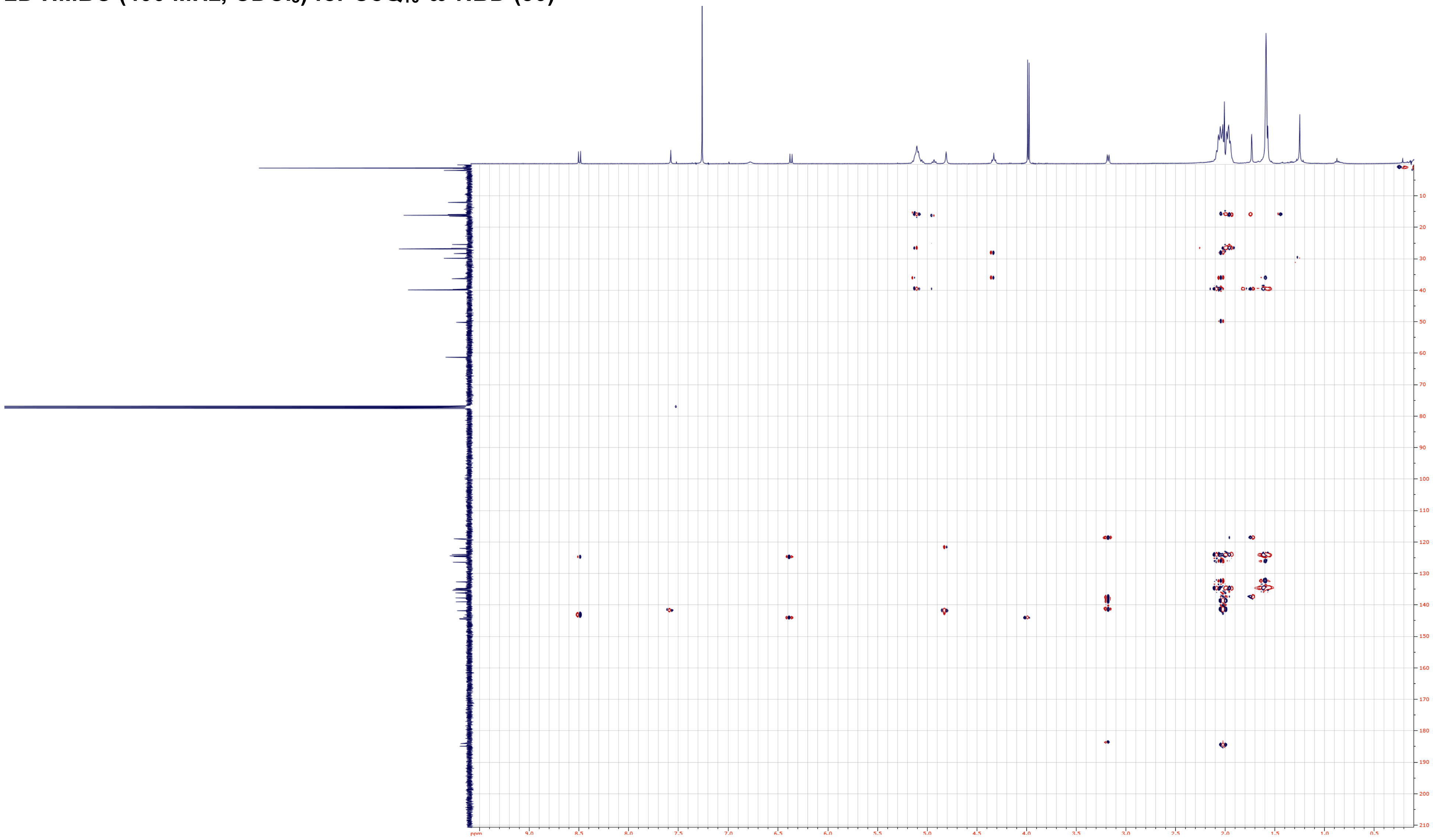

^{1}H NMR (400 MHz, $CDCl_3$) for MK9-ω-NBD (31)

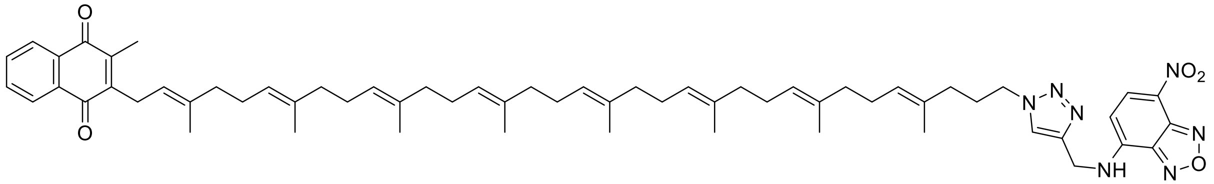

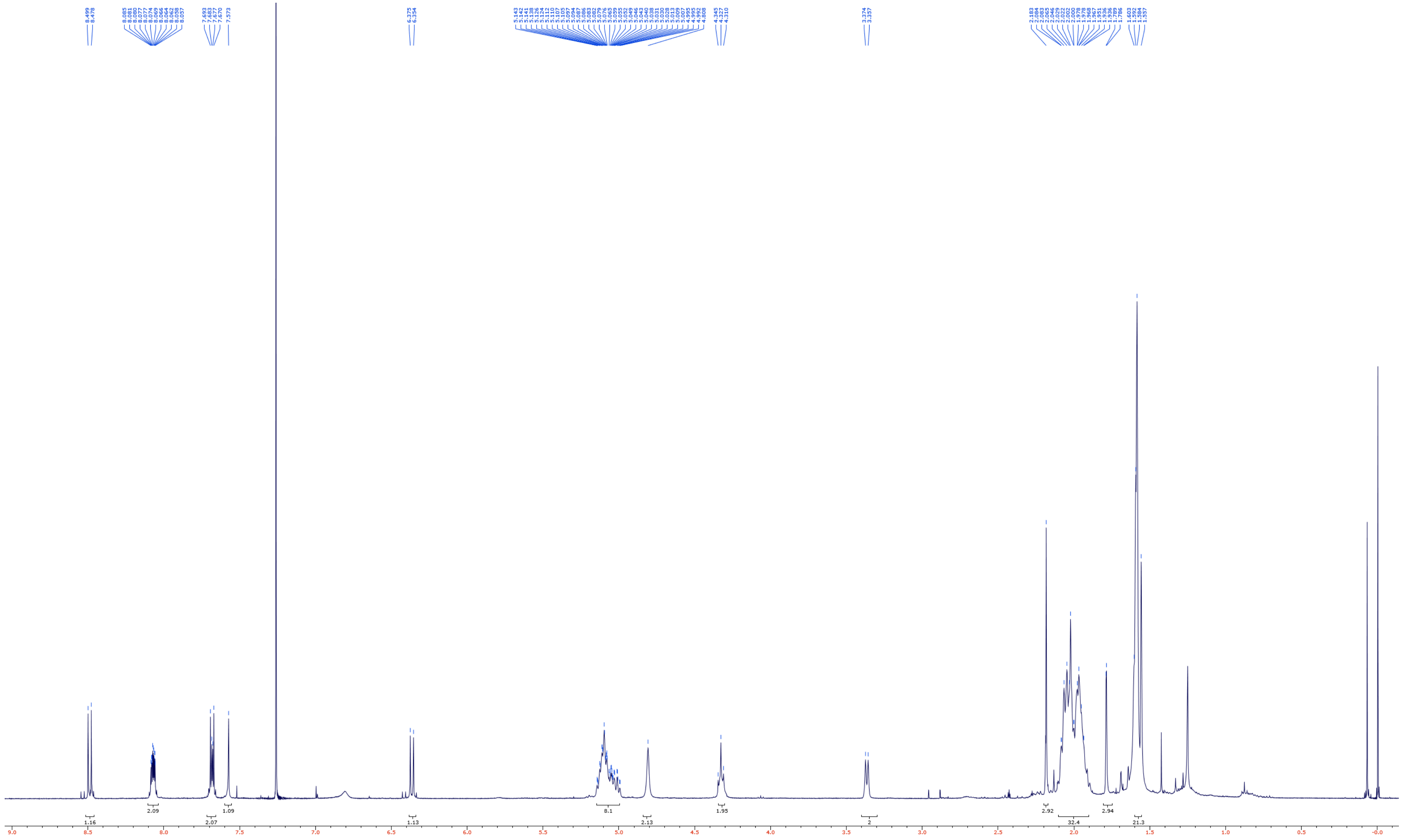

^{13}C NMR (101 MHz, $CDCl_3$) for MK9-ω-NBD (31)

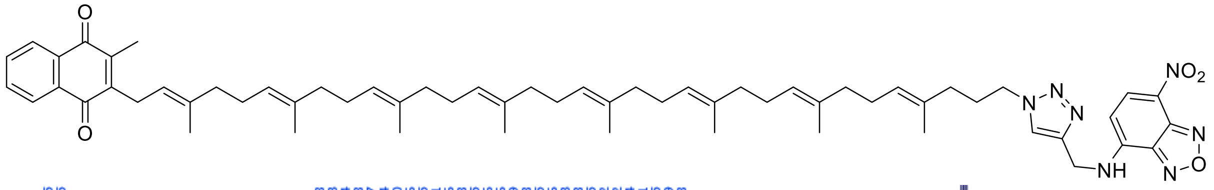

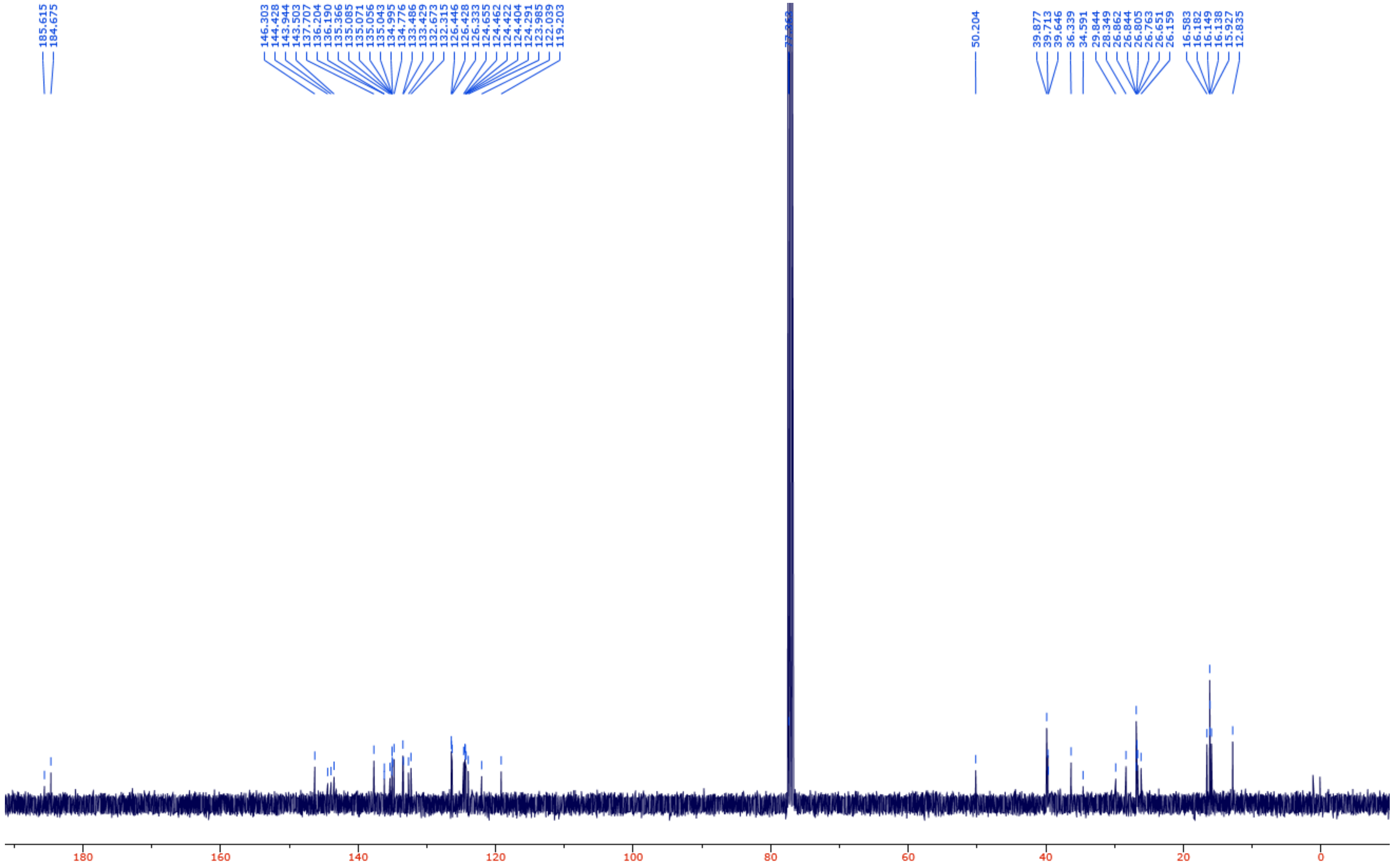

2D COSY (400 MHz, CDCl$_3$) for MK9-ω-NBD (31)

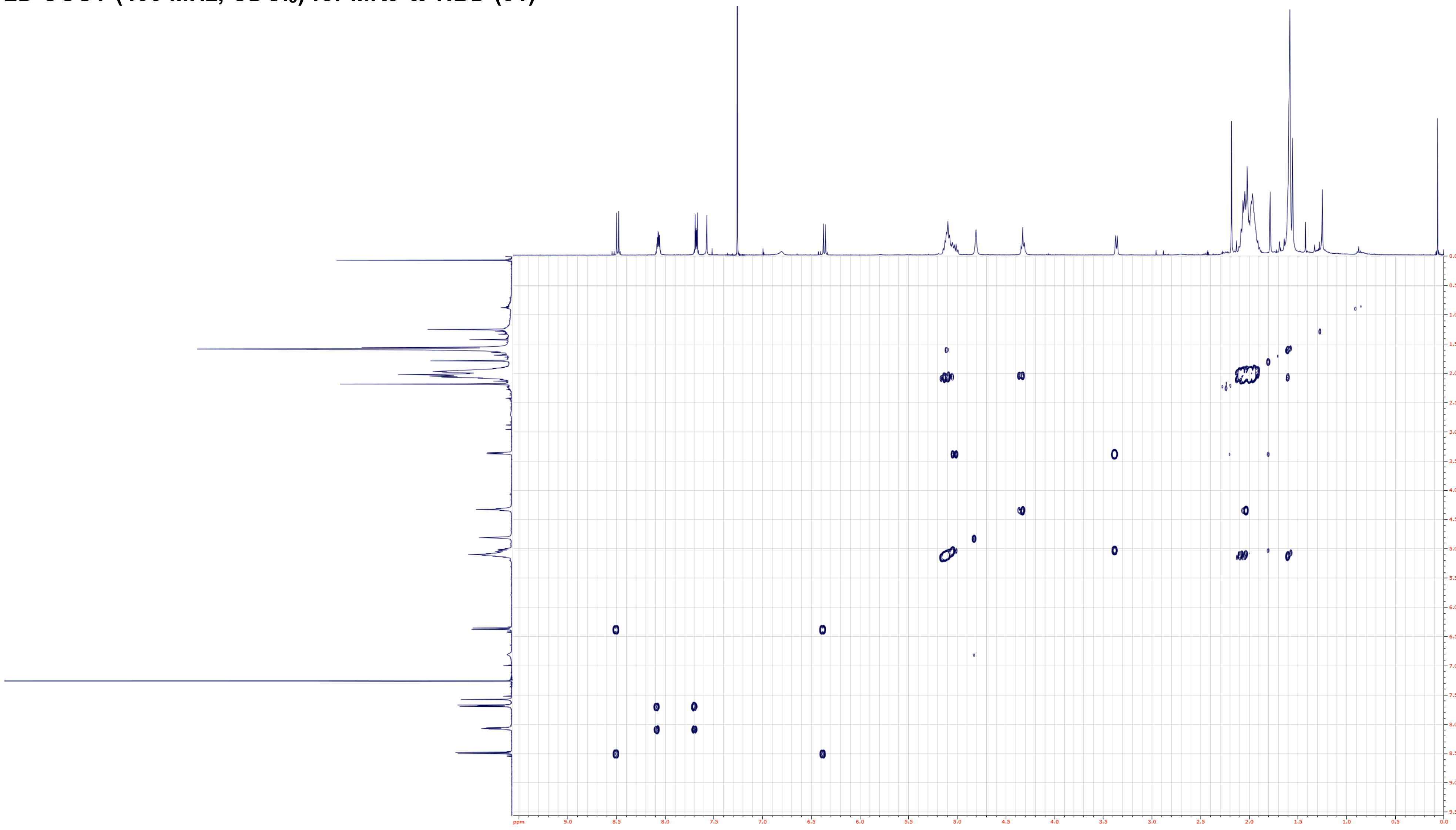

2D HSQC (400 MHz, $CDCl_3$) for MK9-ω-NBD (31)

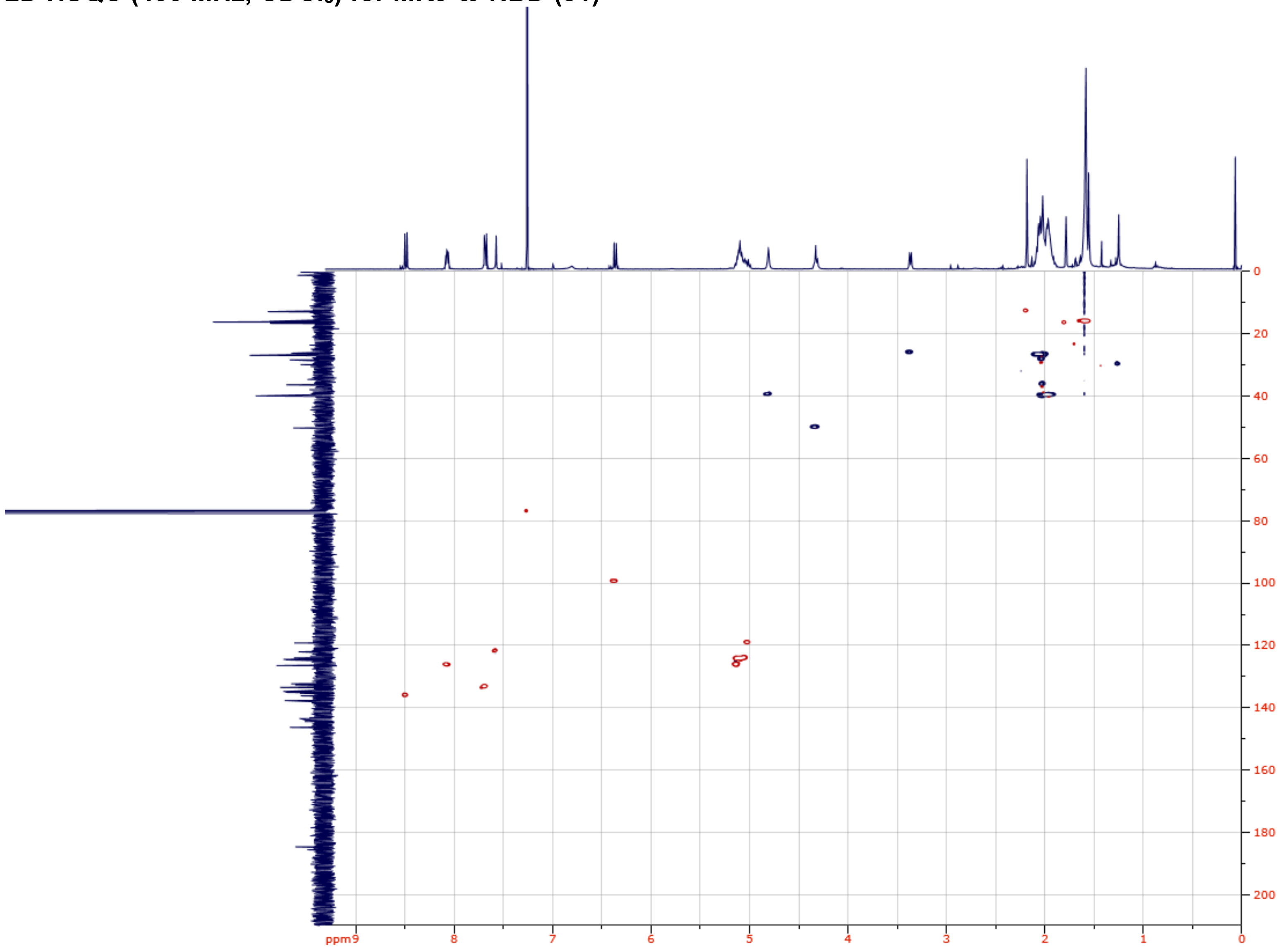

2D HMBC (400 MHz, $CDCl_3$) for MK9-ω-NBD (31)

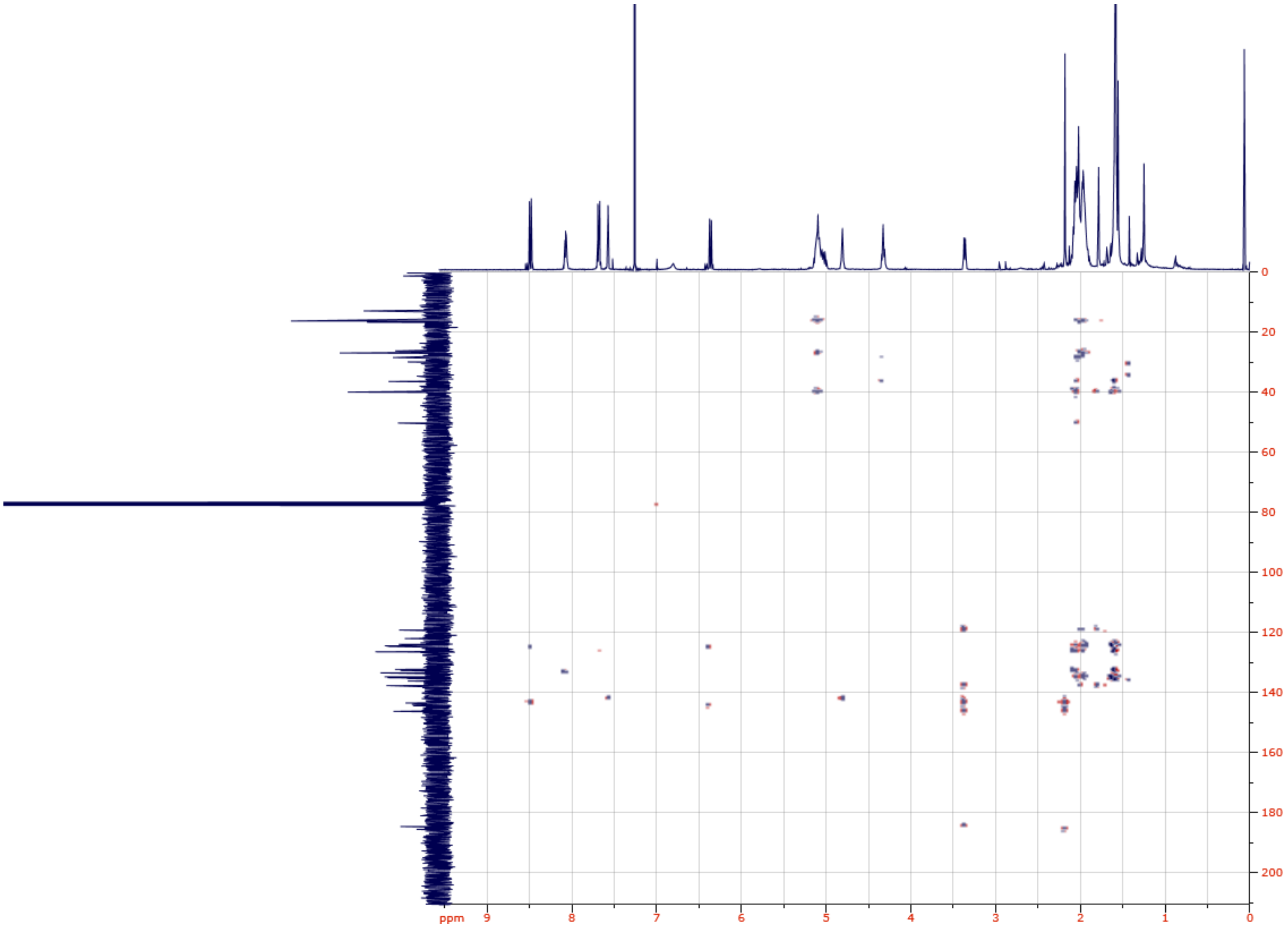

^{1}H NMR (400 MHz, $CDCl_3$) for CoQ10-ω-DNB (32)

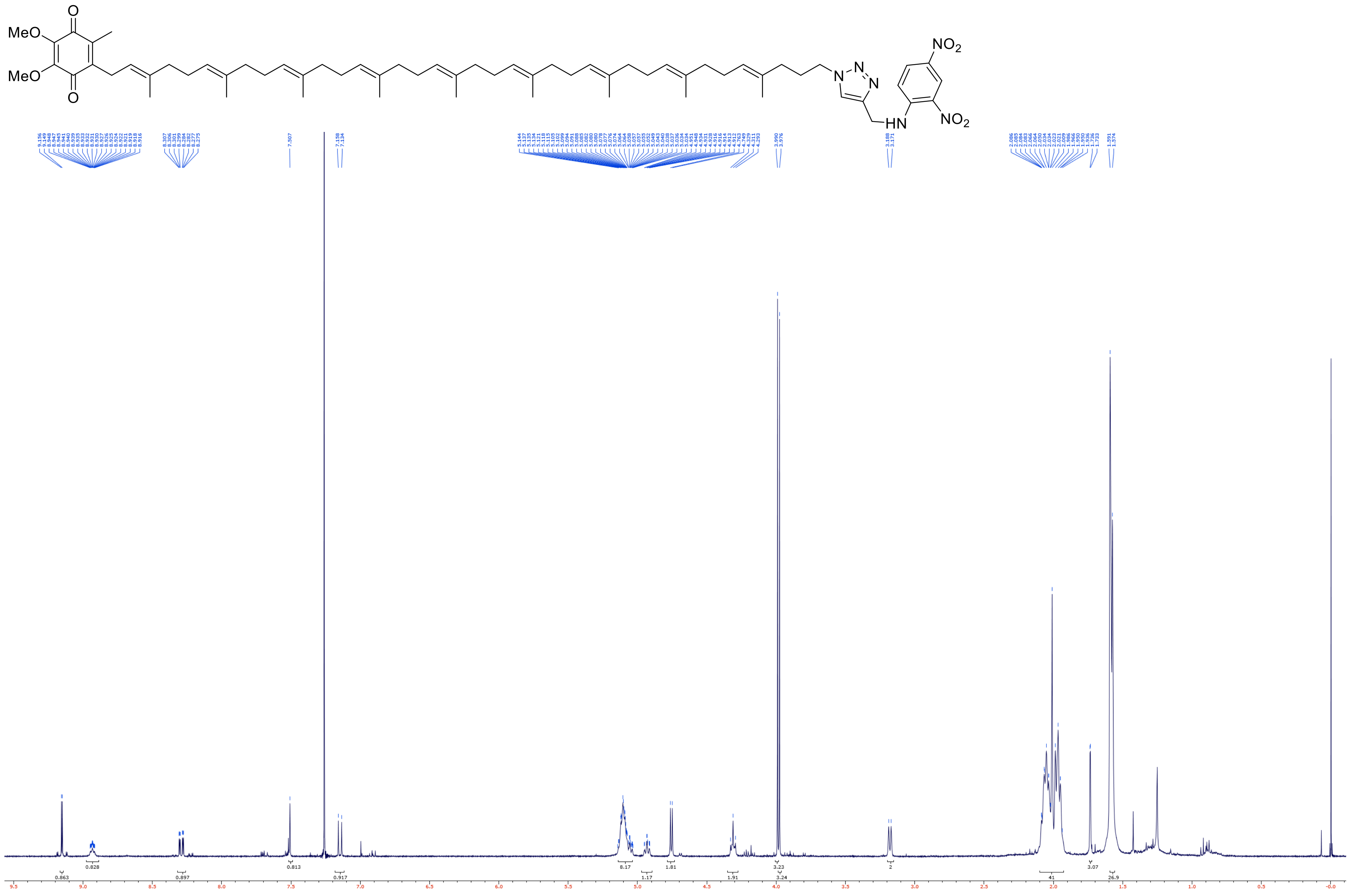

^{13}C NMR (101 MHz, $CDCl_3$) for CoQ10-ω-DNB (32)

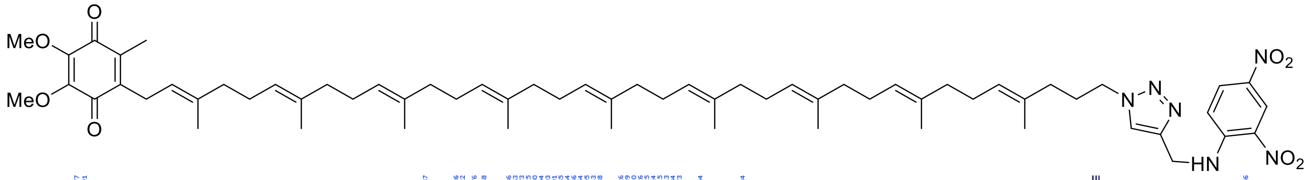

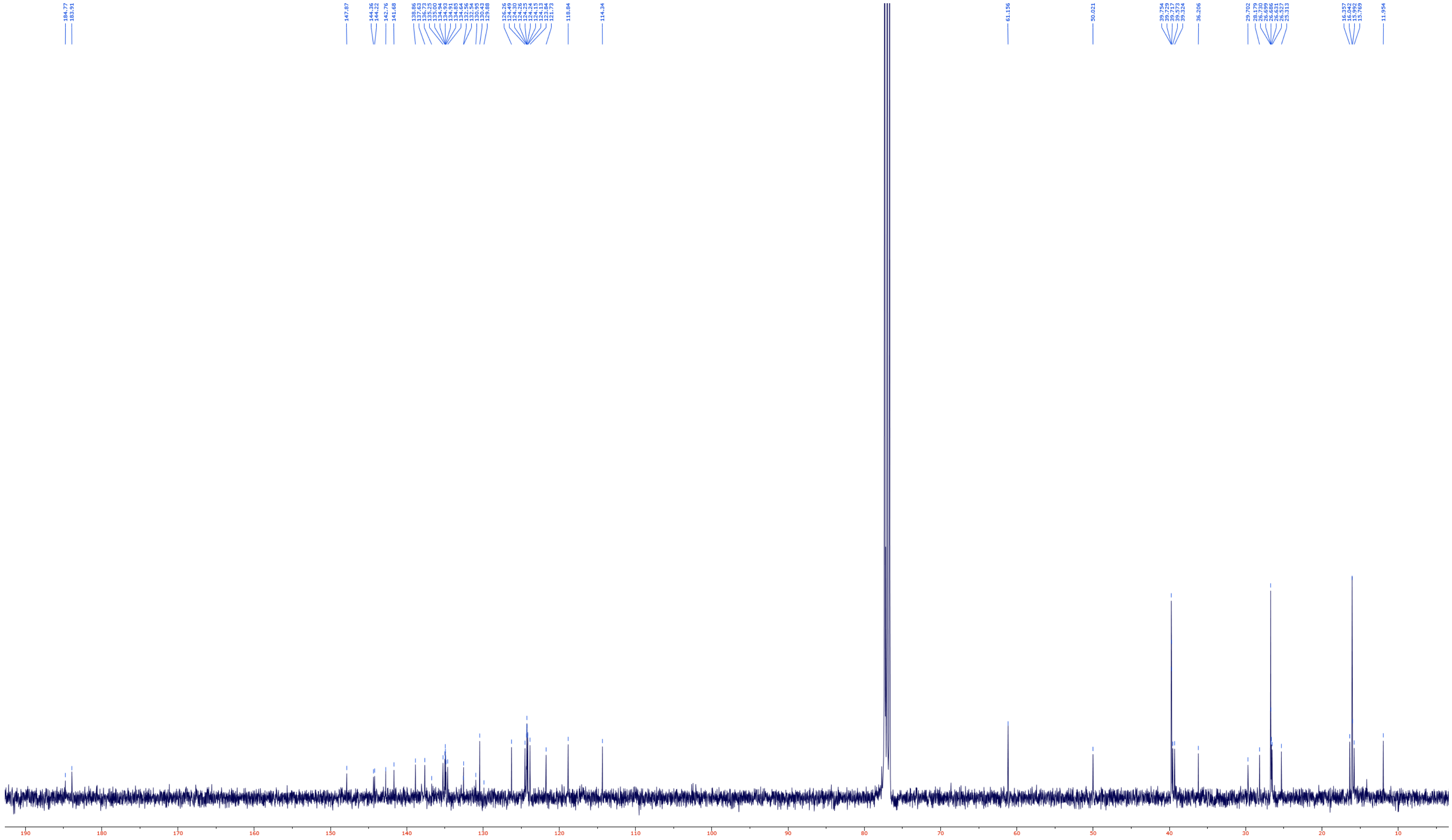

2D HSQC (400 MHz, $CDCl_3$) for CoQ10-ω-DNB (32)

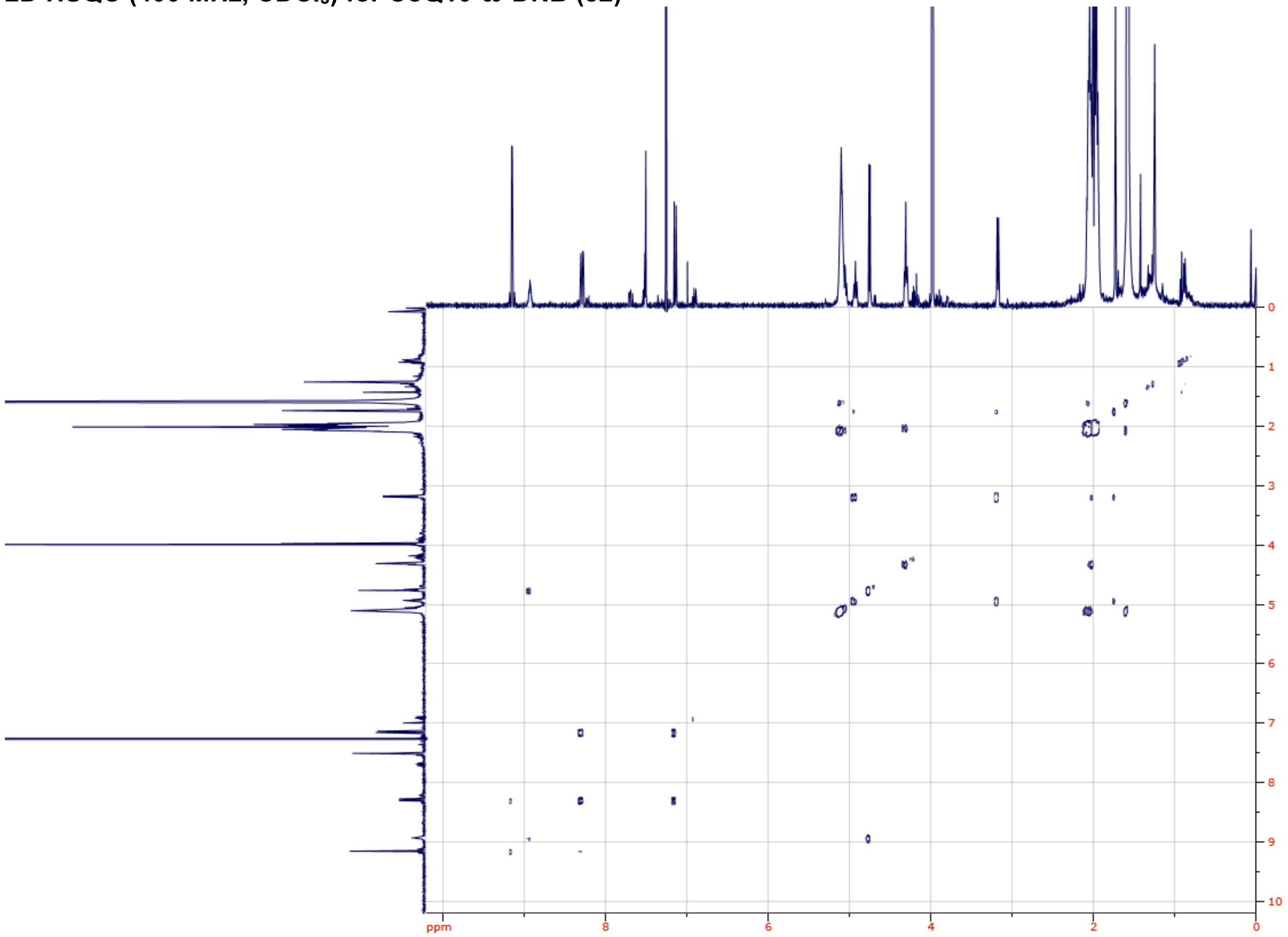

2D HSQC (400 MHz, $CDCl_3$) for CoQ10-ω-DNB (32)

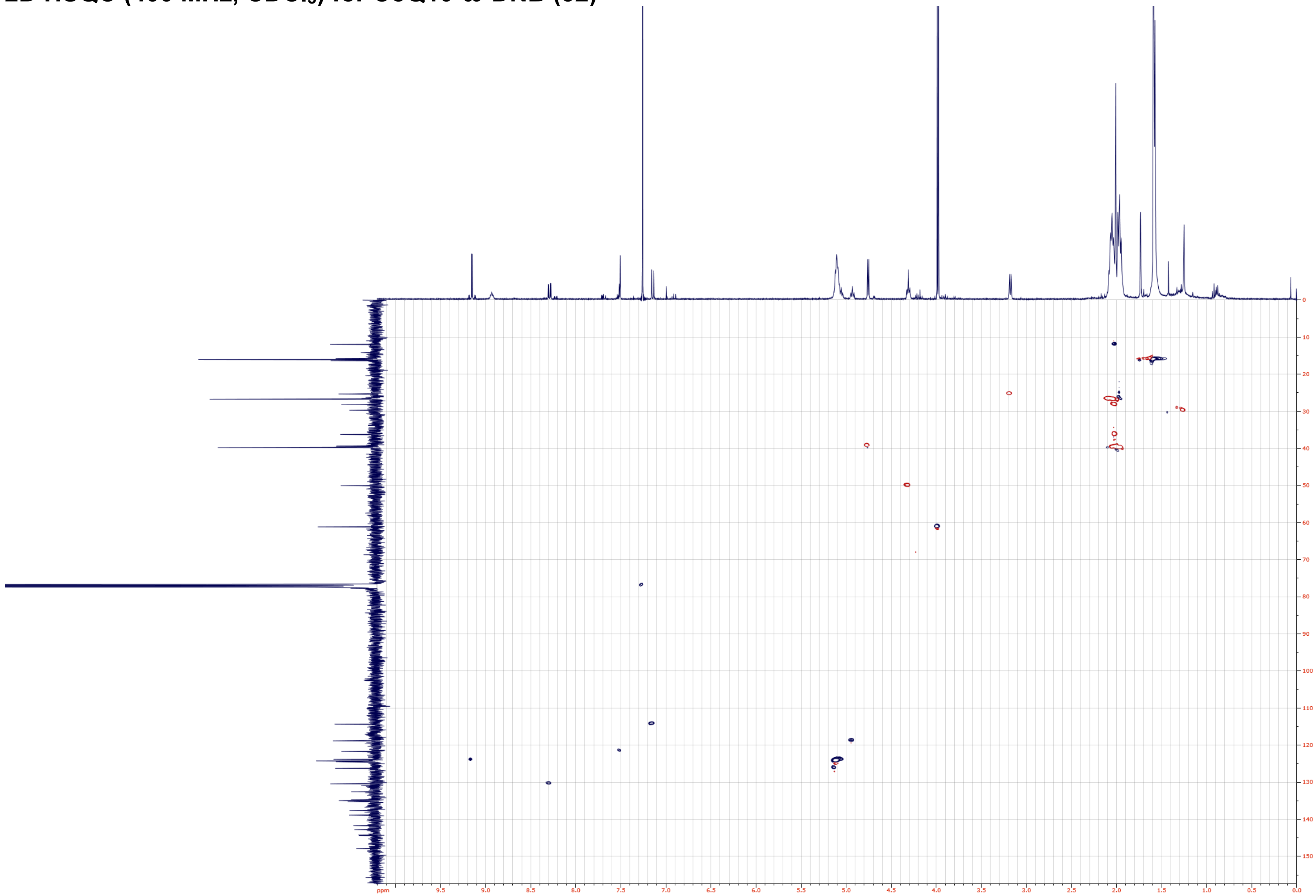

2D HMBC (400 MHz, $CDCl_3$) for CoQ10-ω-DNB (32)

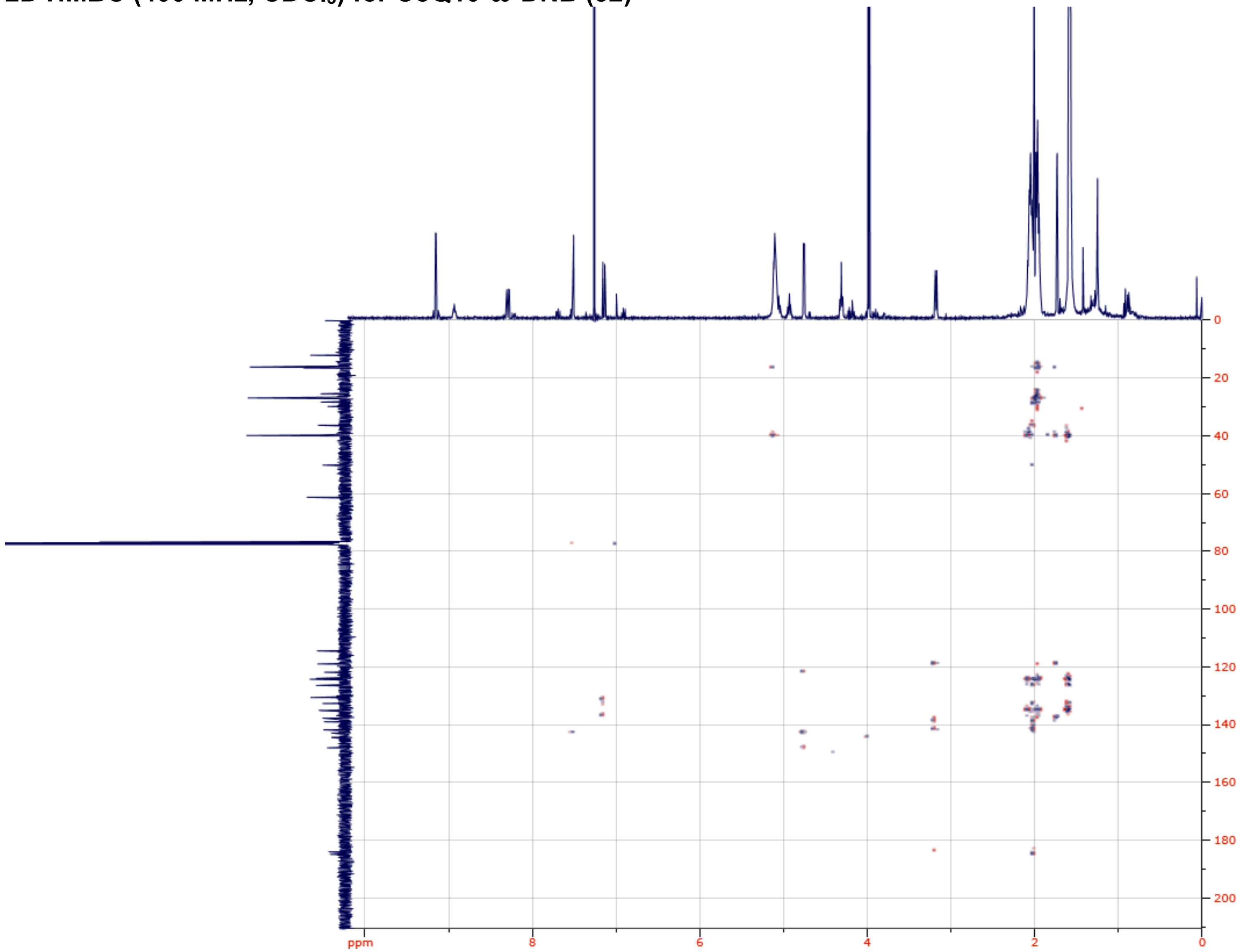

^{1}H NMR (600 MHz, $CDCl_3$) for MK9-ω-DNB (33)

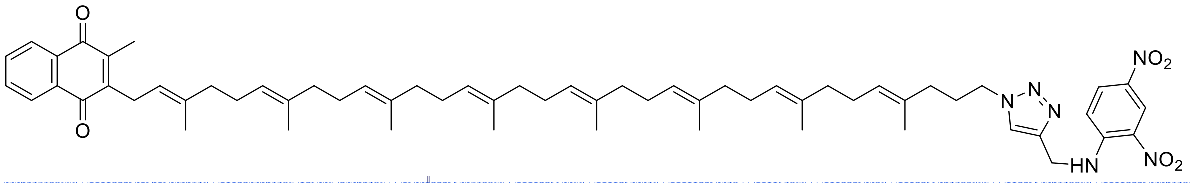

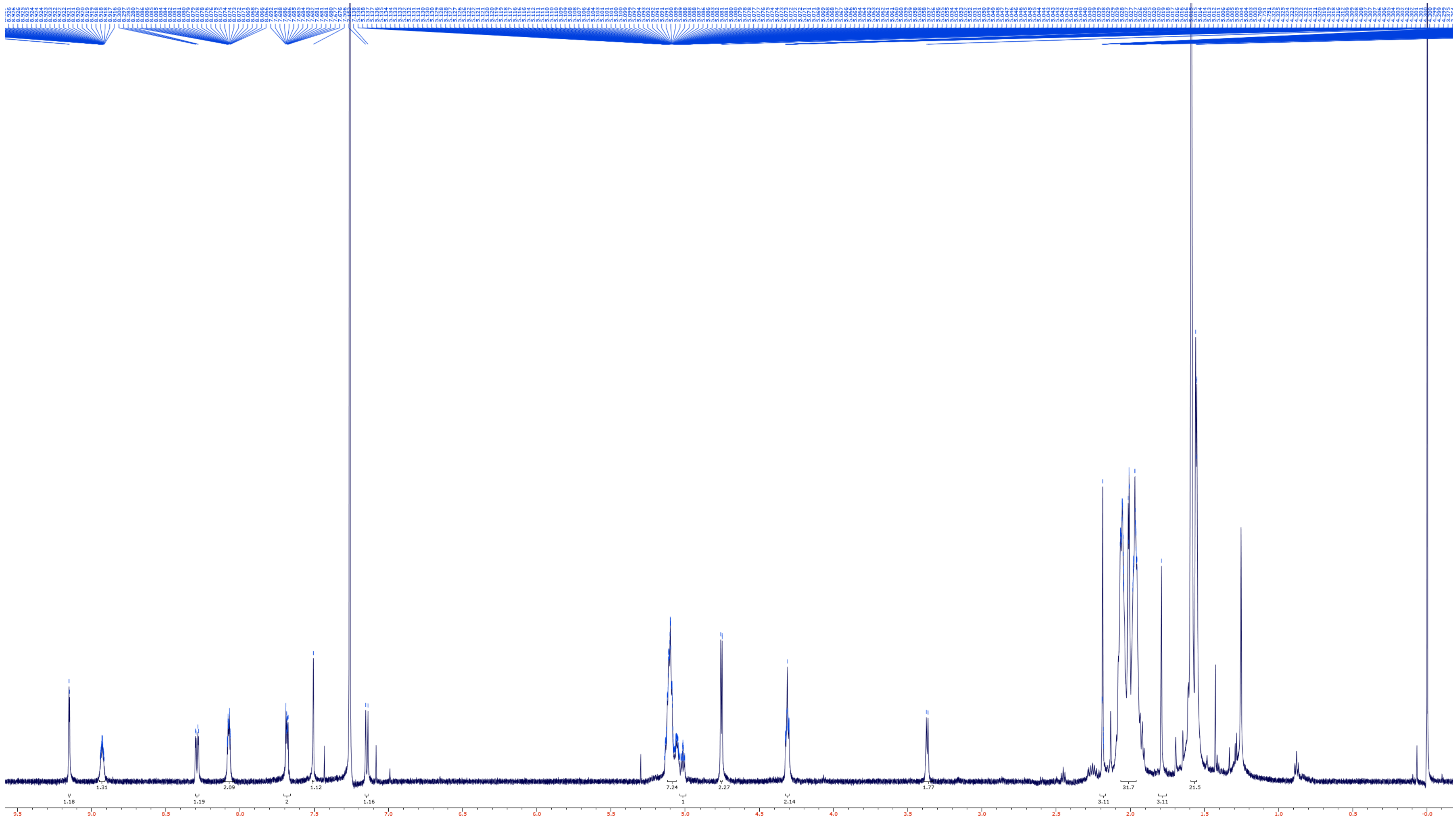

^{13}C NMR (151 MHz, $CDCl_3$) for MK9-ω-DNB (33)

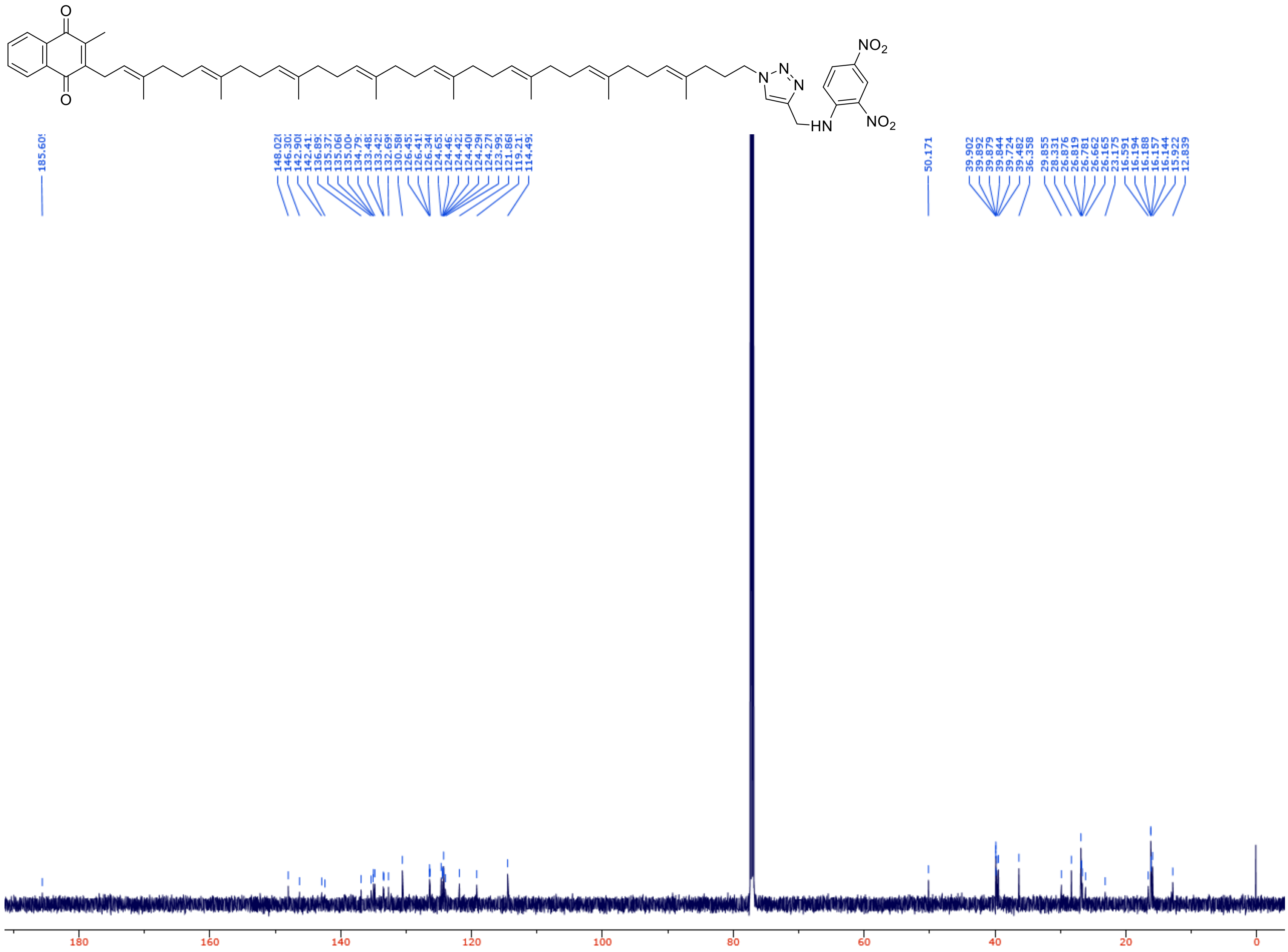

2D COSY NMR (600 MHz, $CDCl_3$) for MK9-ω-DNB (33)

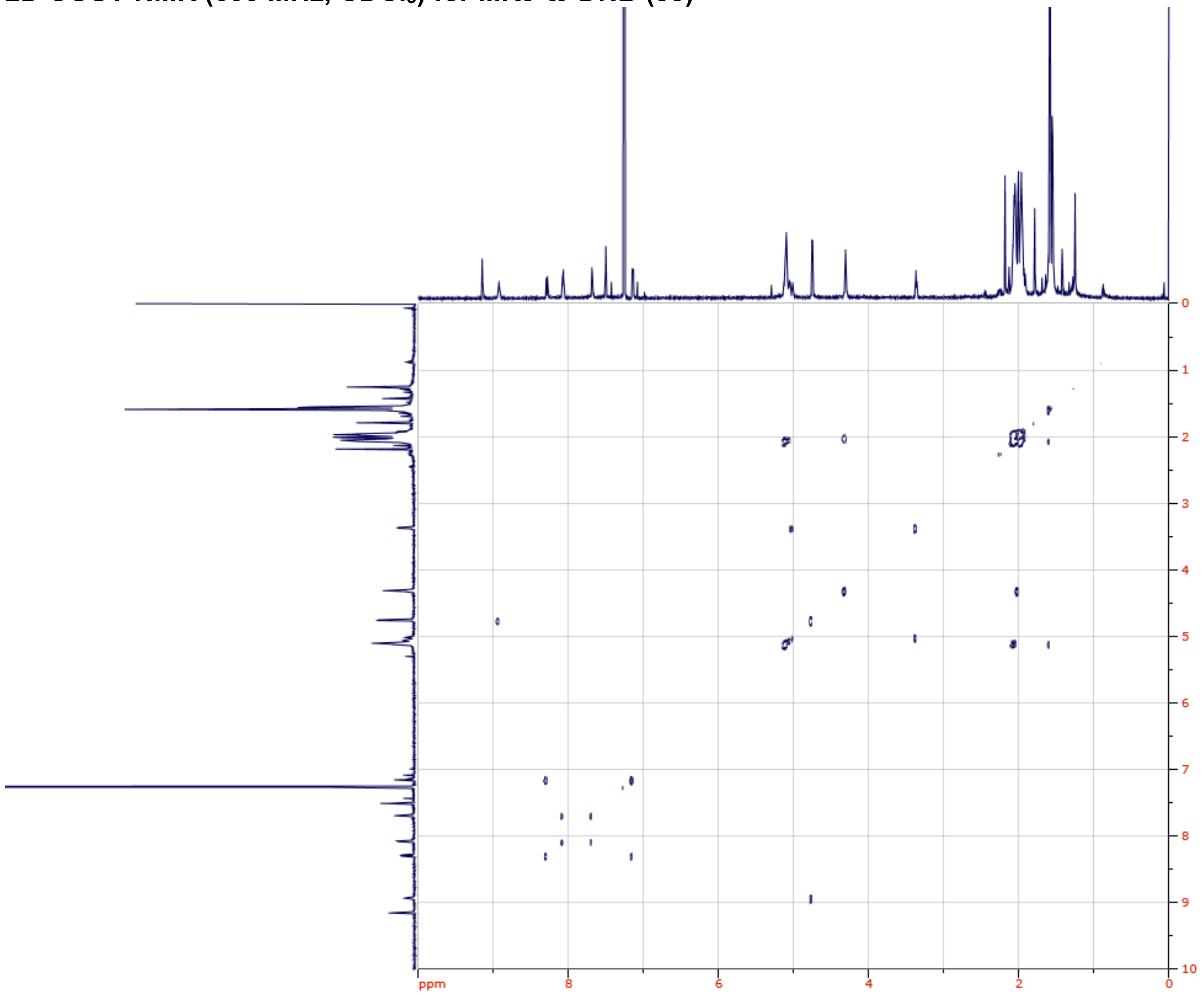

2D HSQC NMR (600 MHz, $CDCl_3$) for MK9-ω-DNB (33)

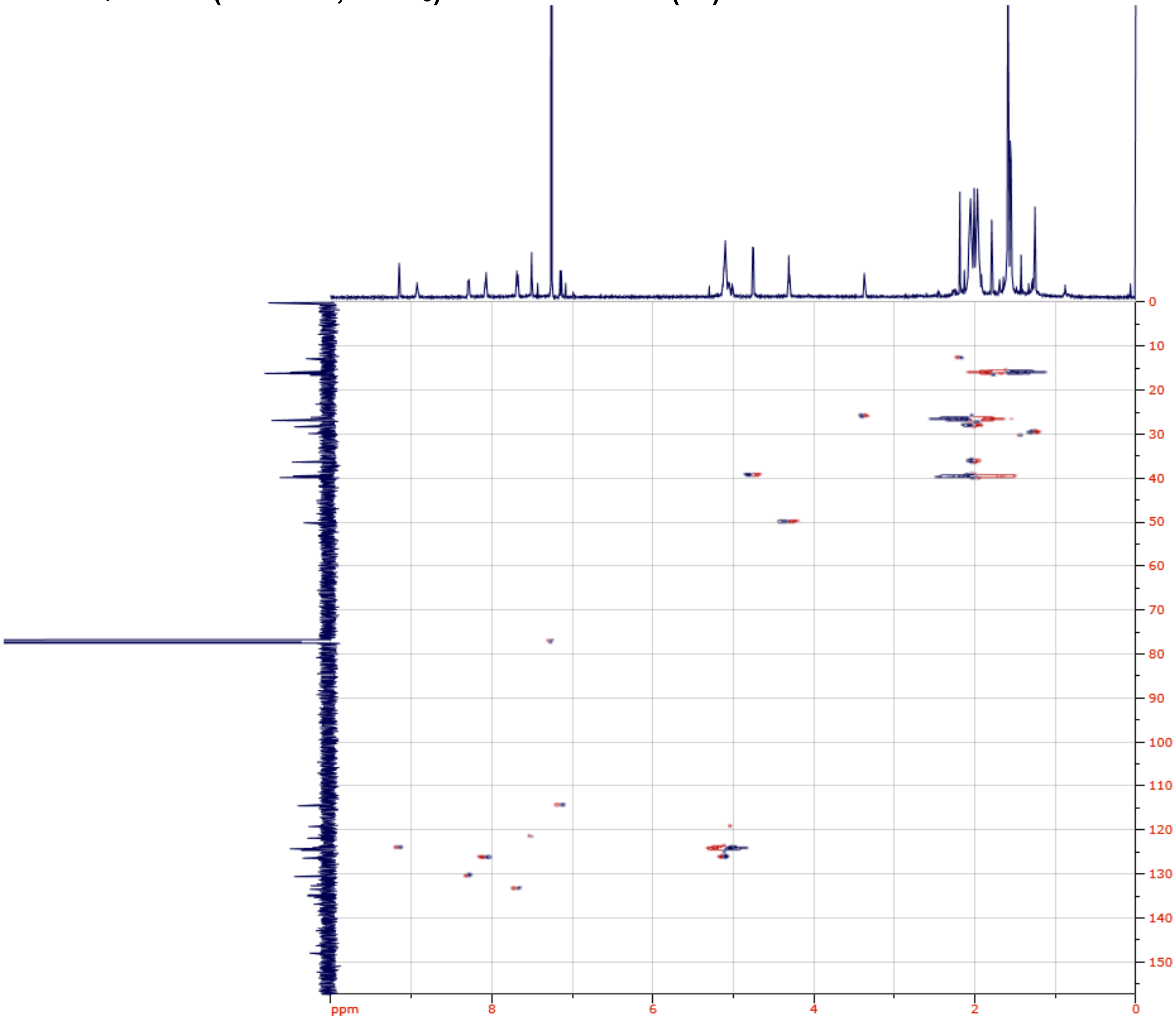

2D HMBC NMR (600 MHz, $CDCl_3$) for MK9-ω-DNB (33)

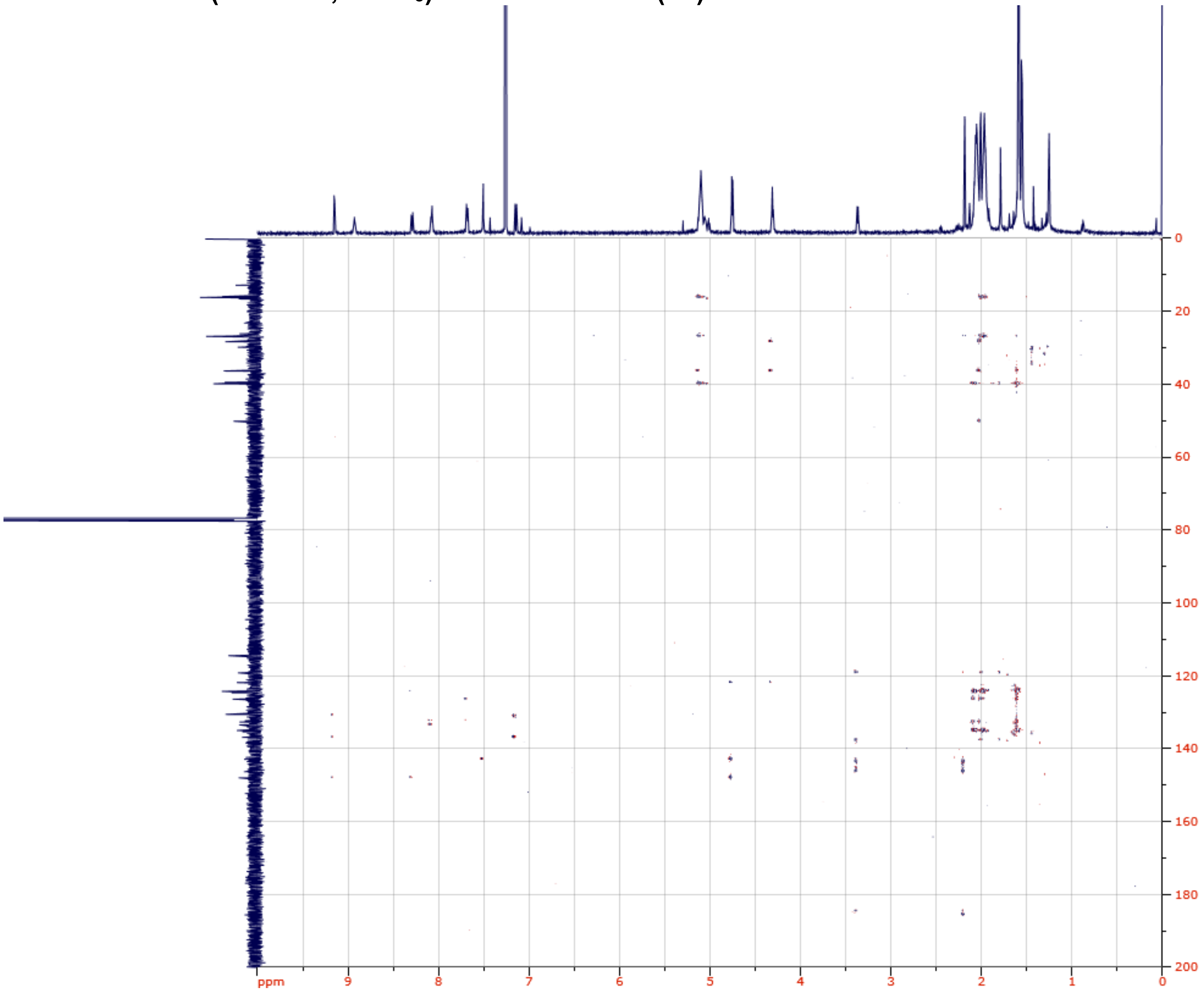

^{1}H NMR (400 MHz, $CDCl_3$) for CoQ10-ω-BODIPY (34)

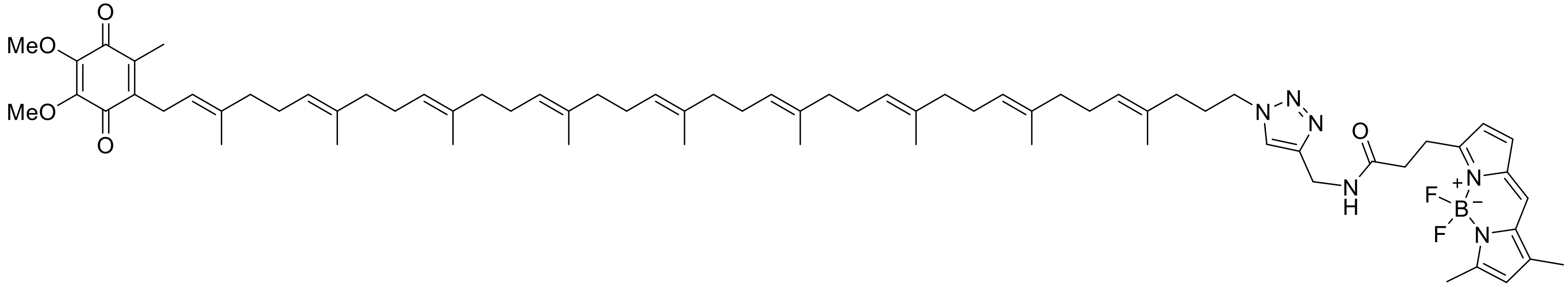

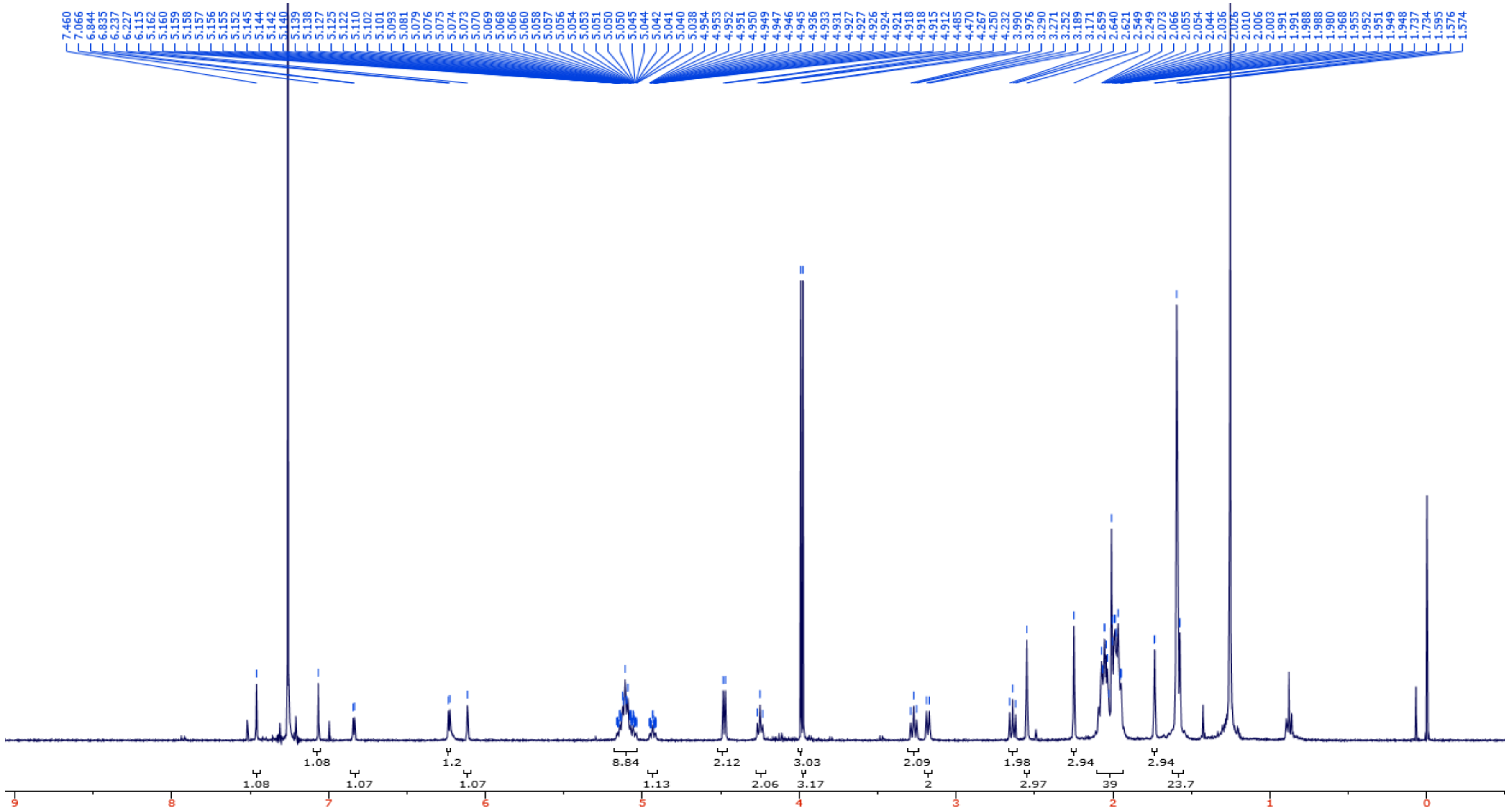

^{13}C NMR (151 MHz, $CDCl_3$) for CoQ10-ω-BODIPY (34)

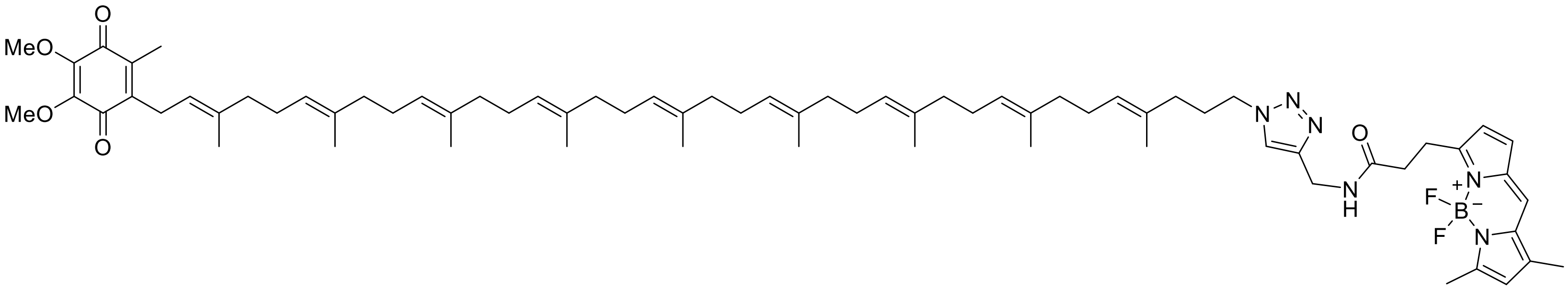

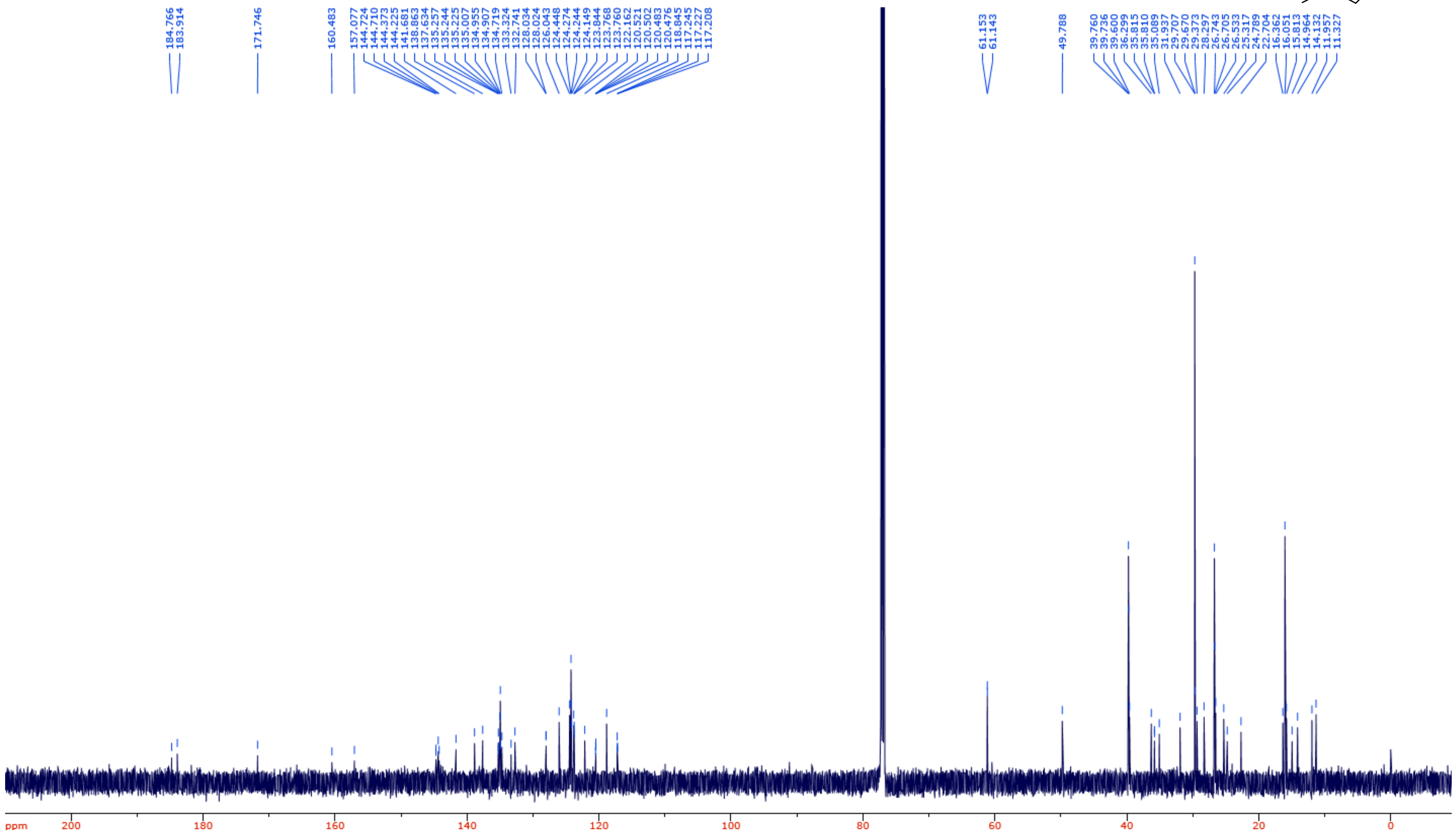

2D COSY (600 MHz, $CDCl_3$) for CoQ10-ω-BODIPY (34)

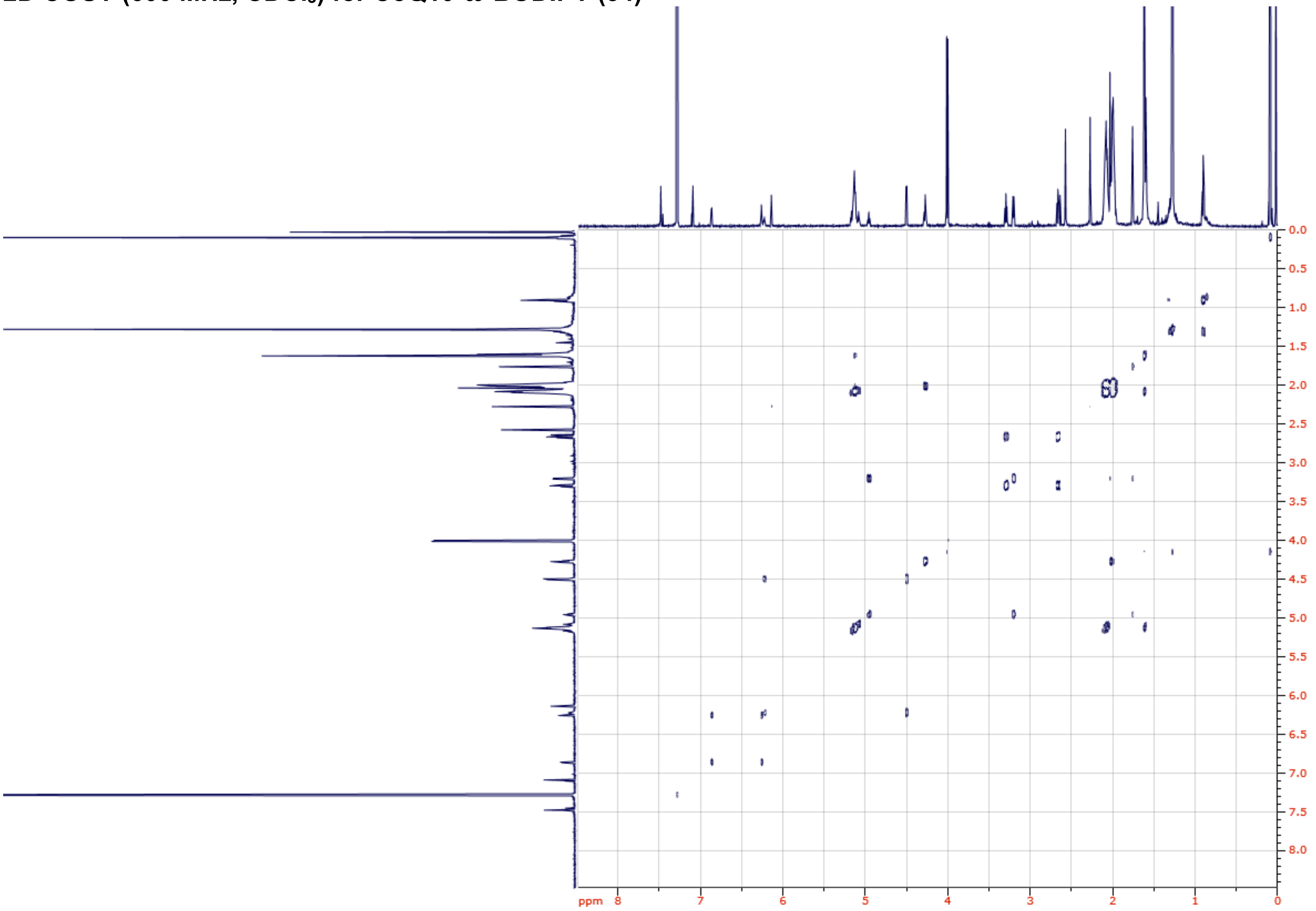

2D HSQC (600 MHz, $CDCl_3$) for CoQ10-ω-BODIPY (34)

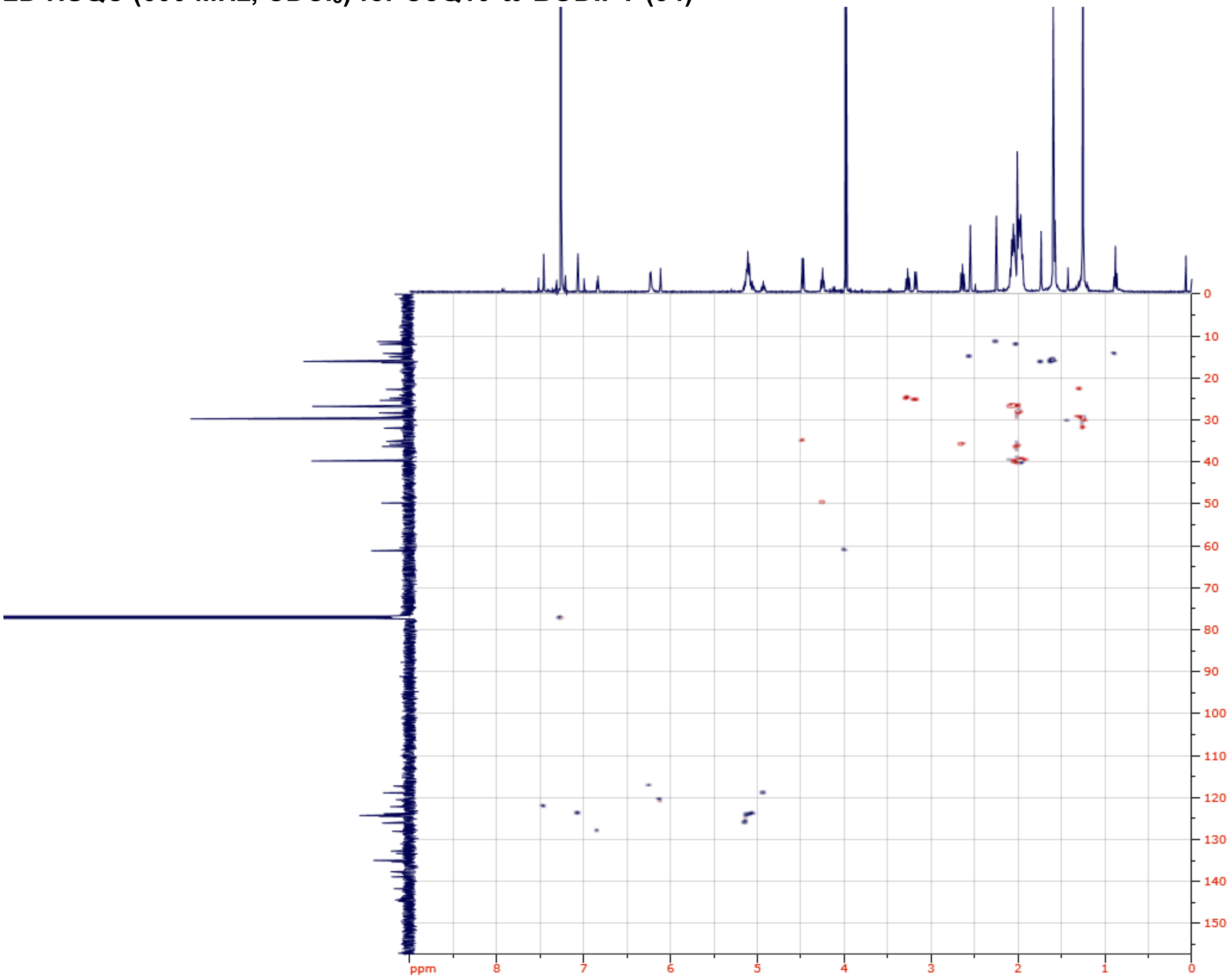

2D HMBC (600 MHz, $CDCl_3$) for CoQ10-ω-BODIPY (34)

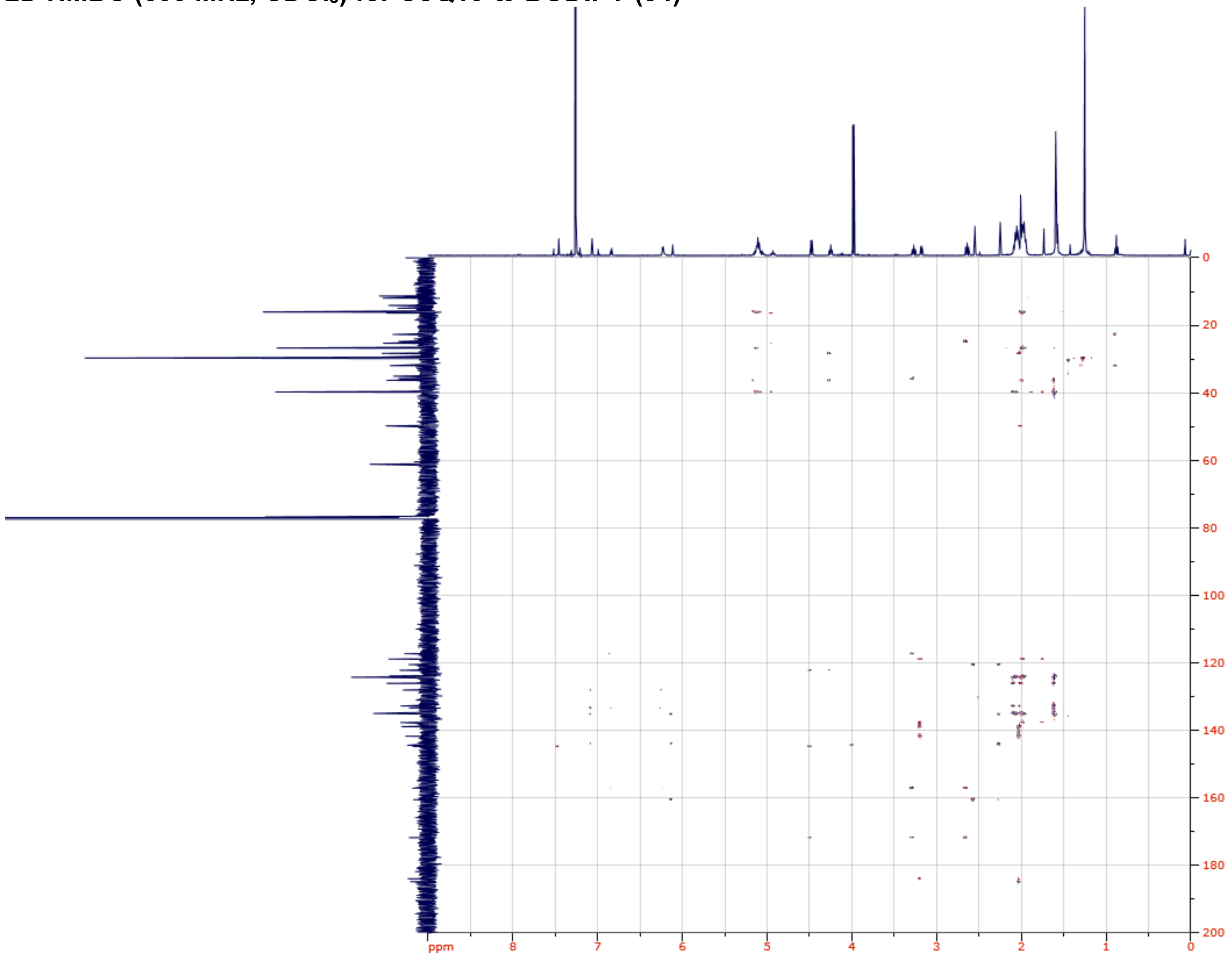

^{19}F NMR (565 MHz, $CDCl_3$) for CoQ10-ω-BODIPY (34)

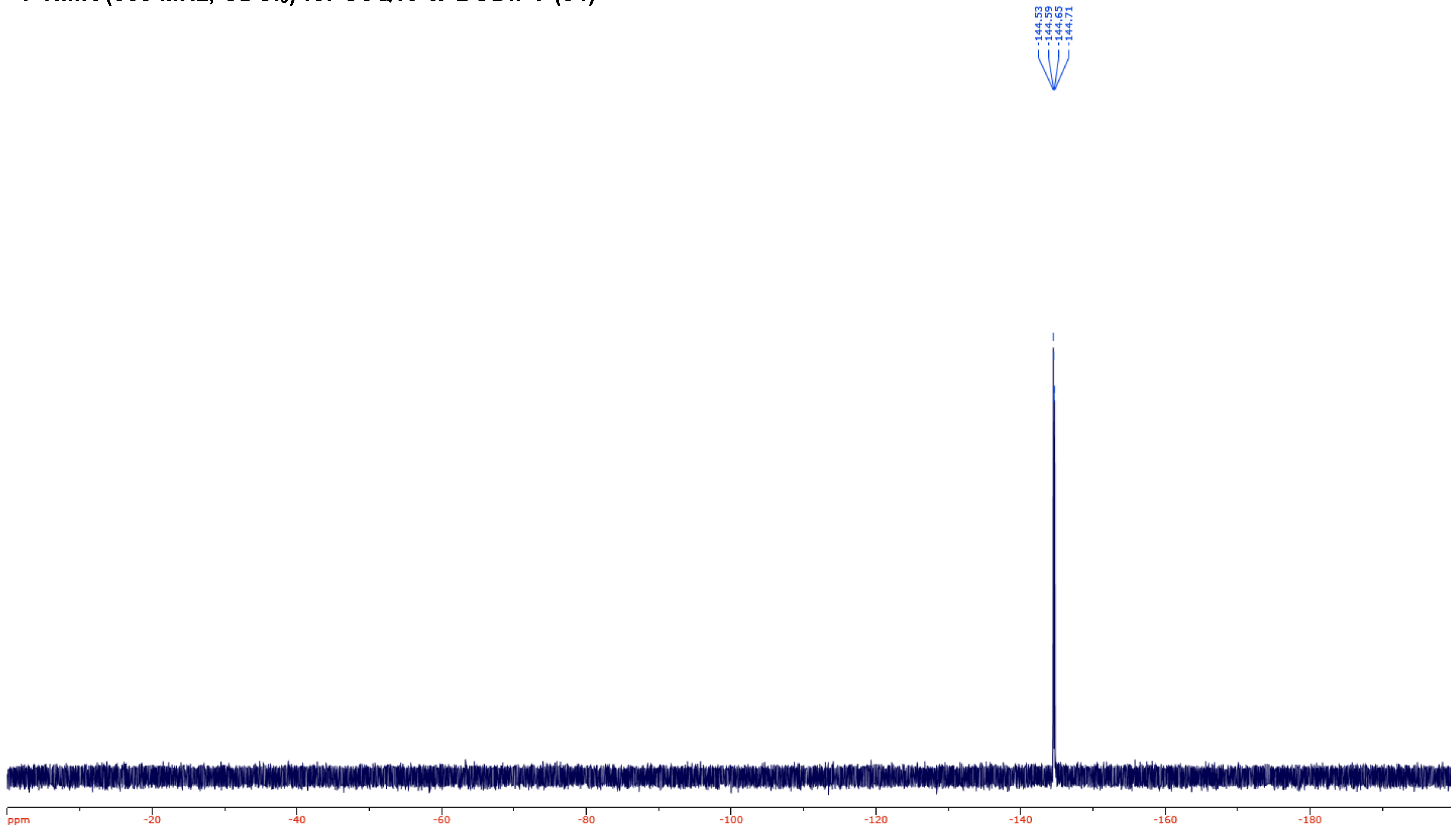

^{1}H NMR (400 MHz, $CDCl_3$) for MK9-ω-BODIPY (35)

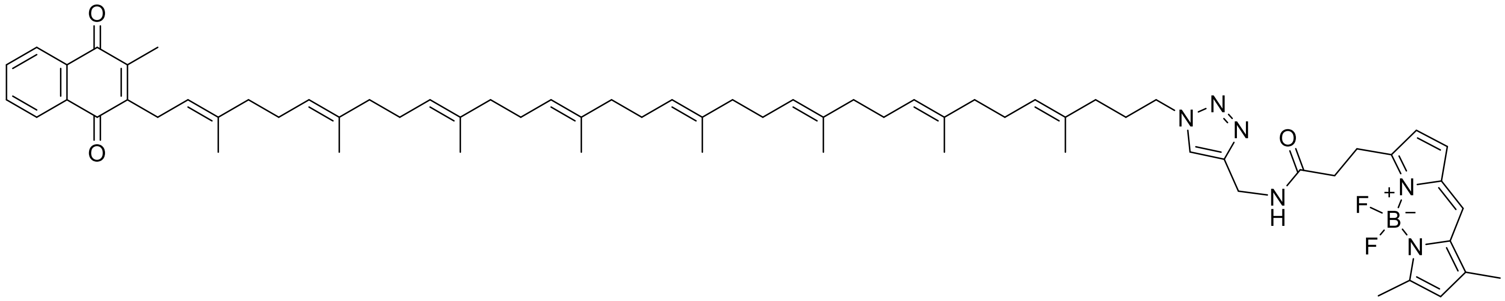

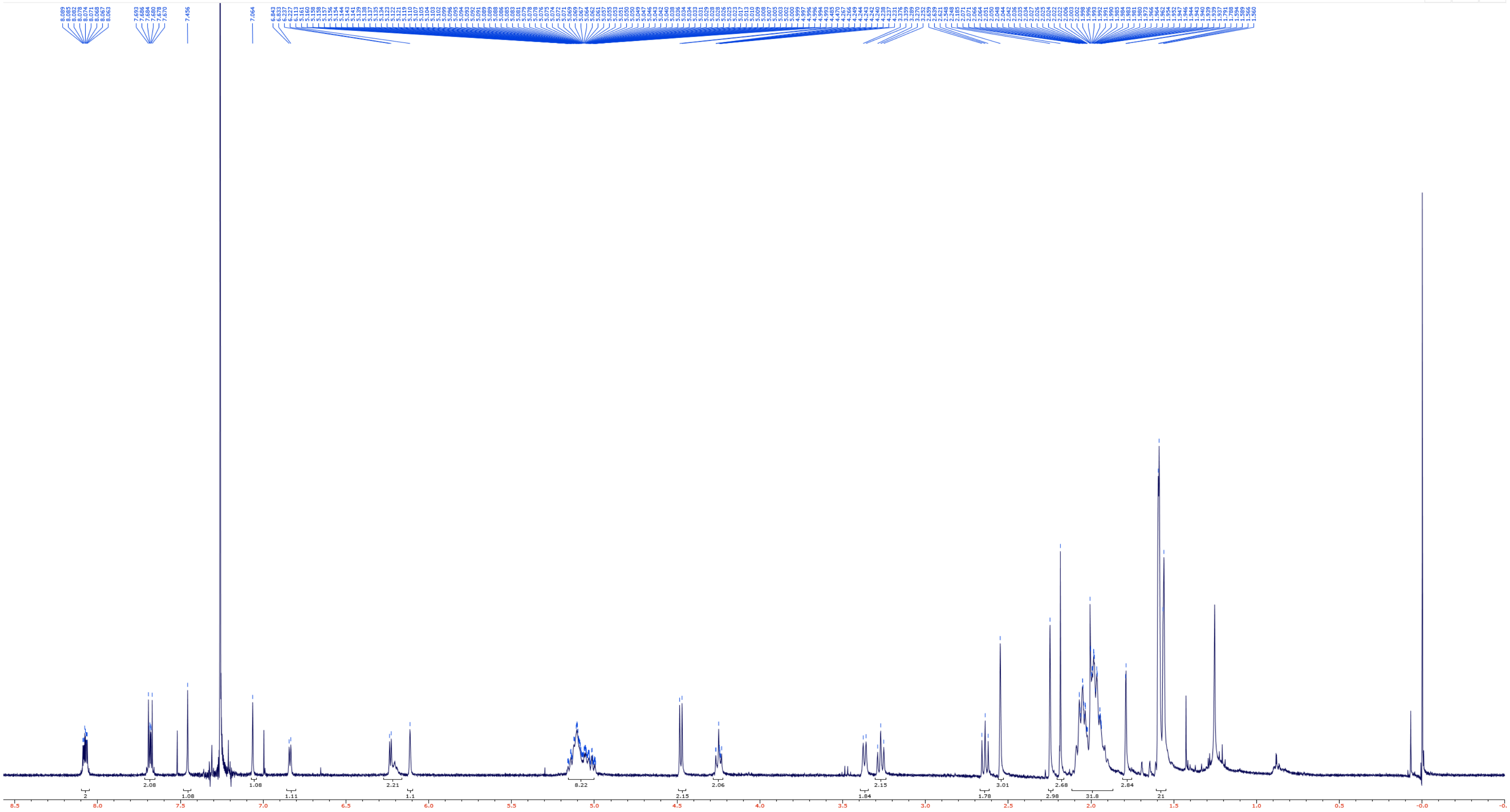

^{13}C NMR (151 MHz, $CDCl_3$) for MK9-ω-BODIPY (35)

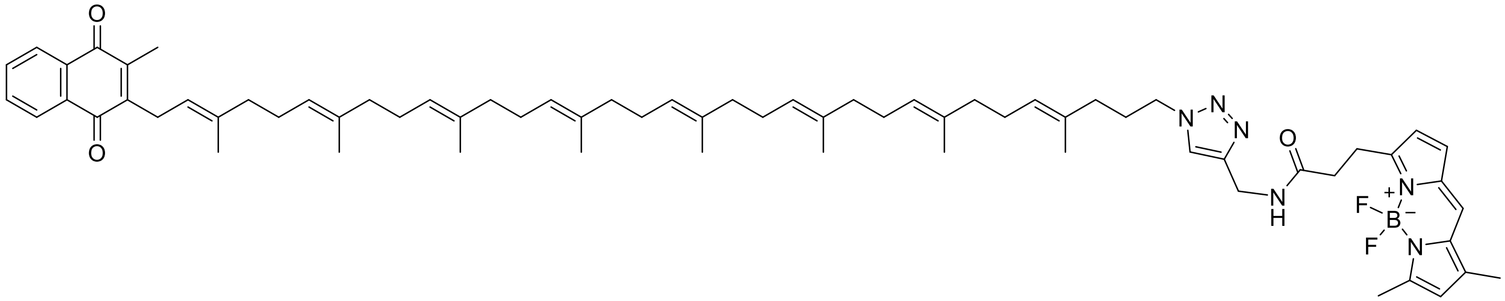

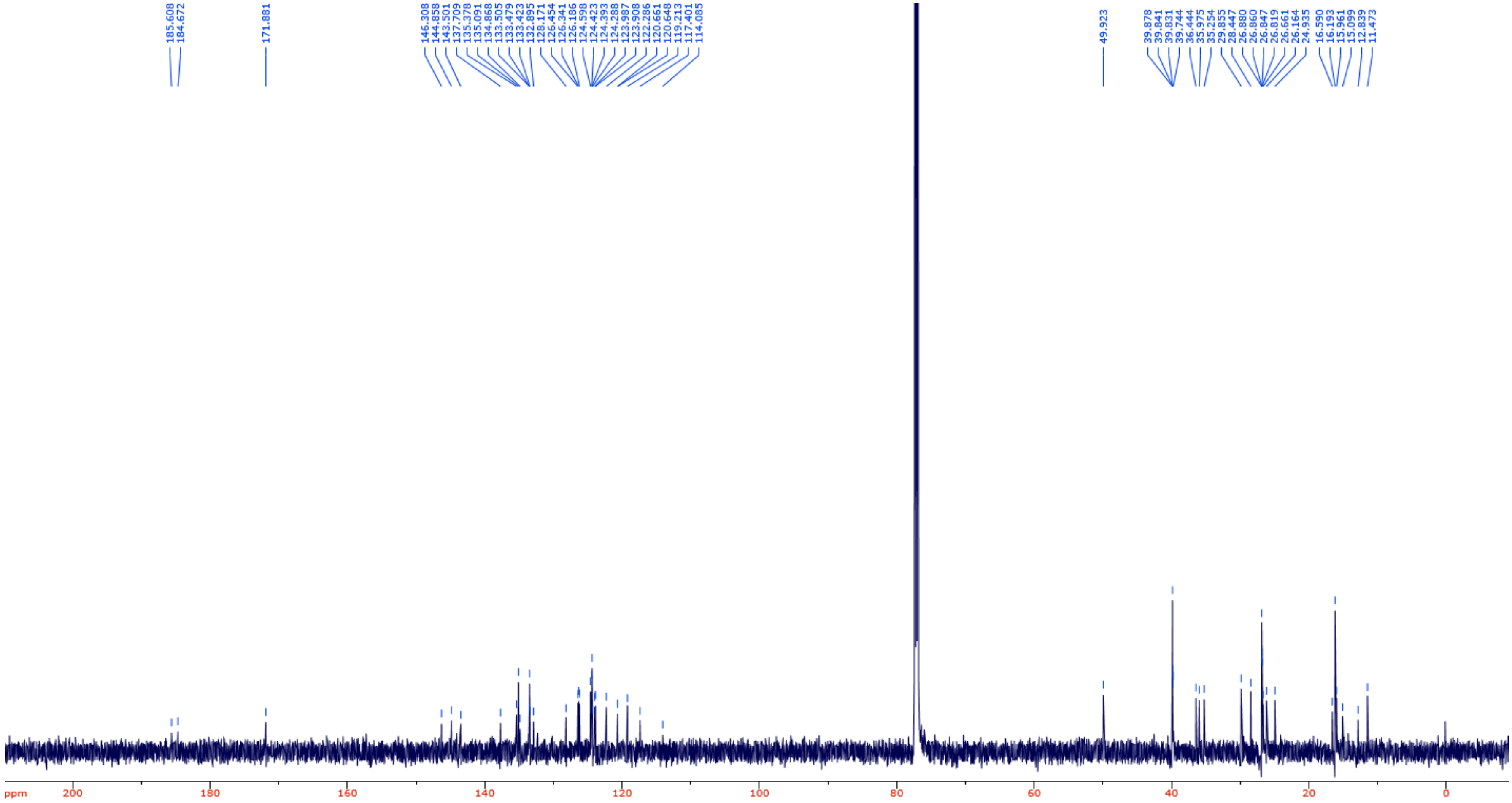

2D COSY (600 MHz, $CDCl_3$) for MK9-ω-BODIPY (35)

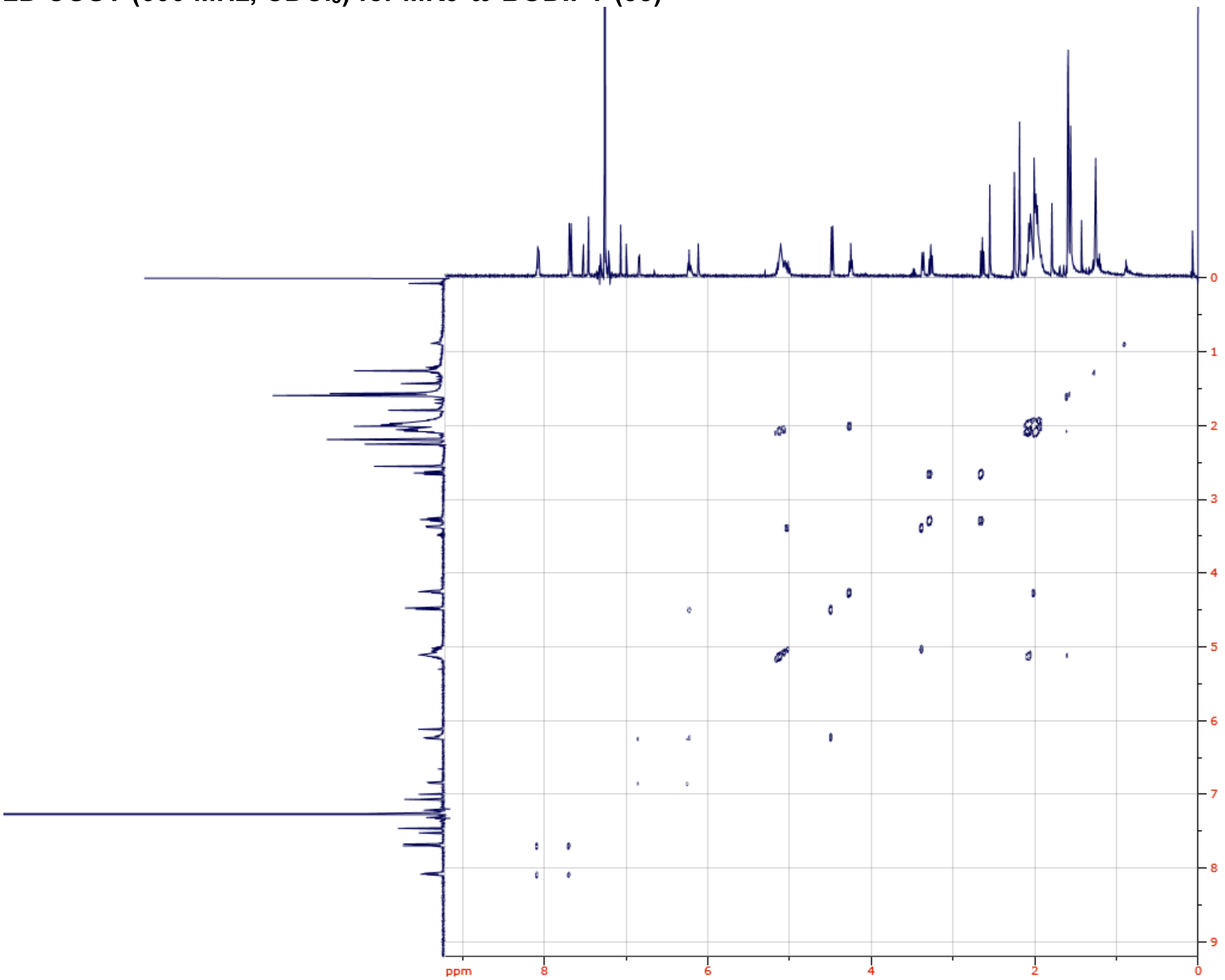

2D HSQC (600 MHz, $CDCl_3$) for MK9-ω-BODIPY (35)

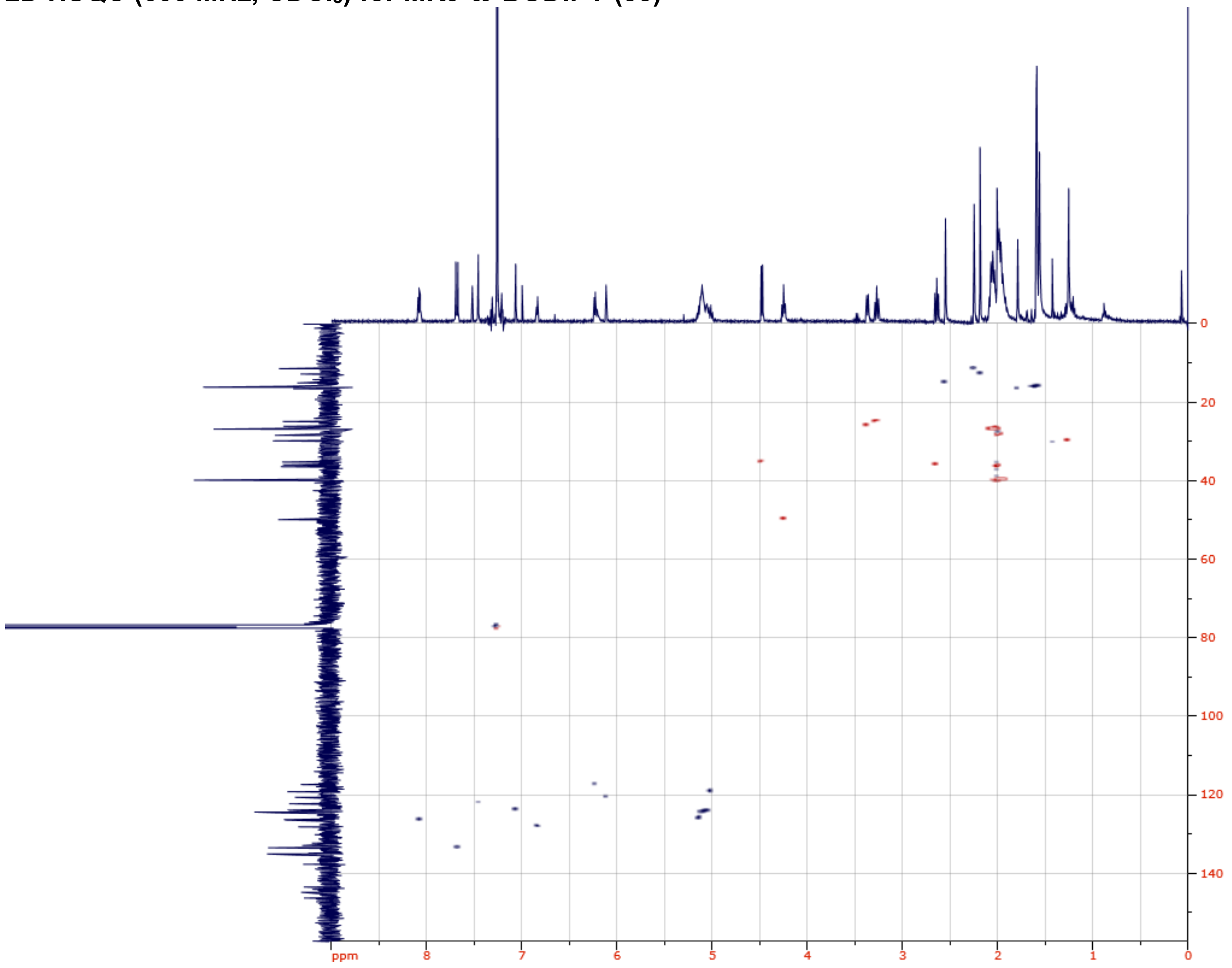

2D HMBC (600 MHz, $CDCl_3$) for MK9-ω-BODIPY (35)

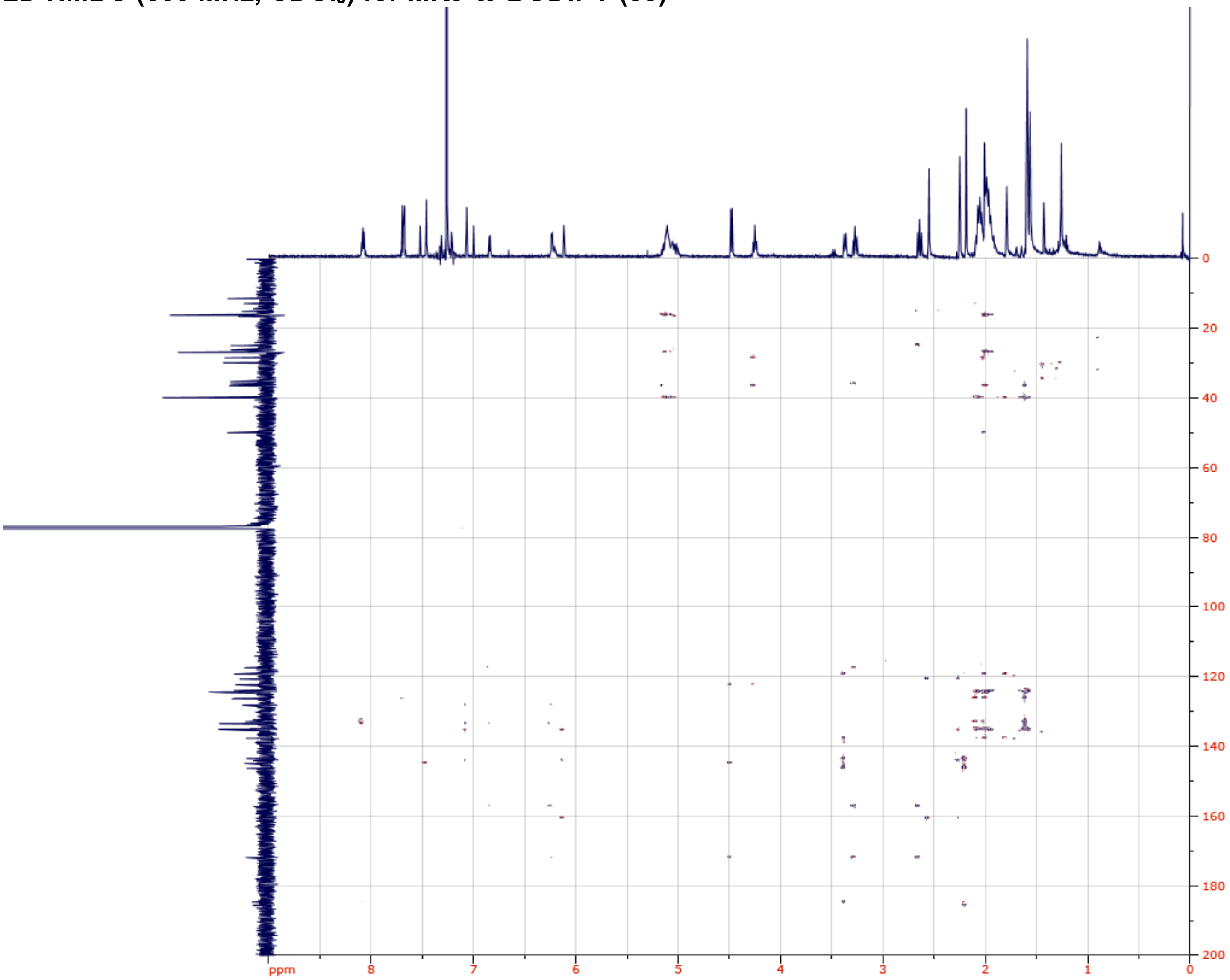

^{19}F NMR (565 MHz, $CDCl_3$) for MK9-ω-BODIPY (35)

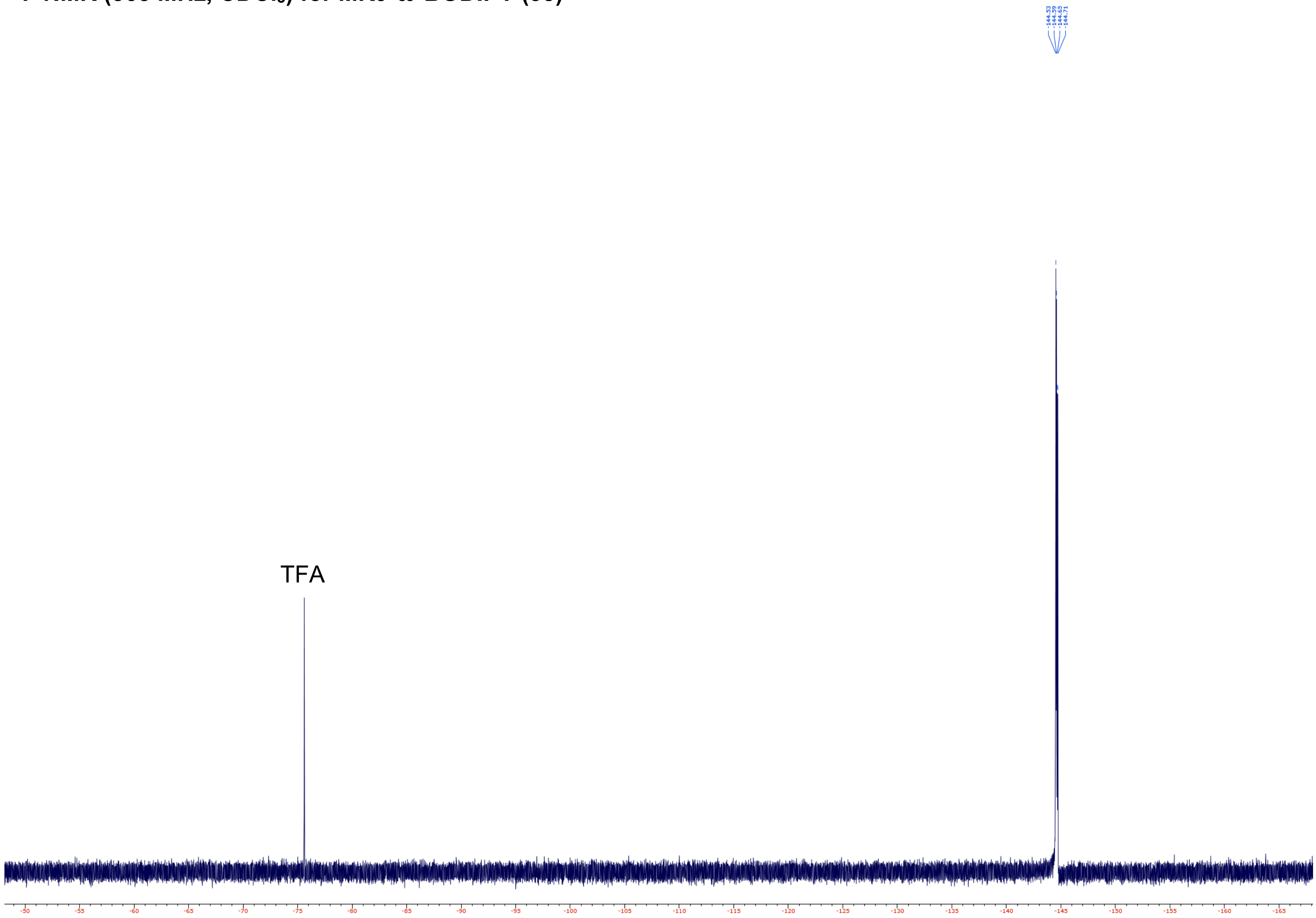

^{1}H NMR (400 MHz, $CDCl_3$) for CoQ10-ω-biotin (36)

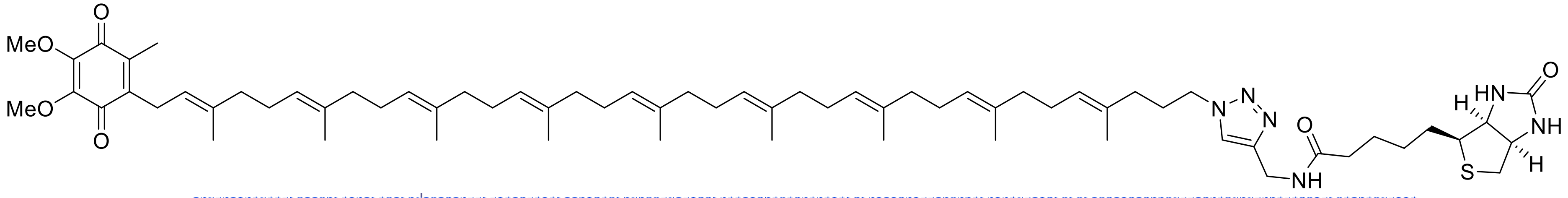

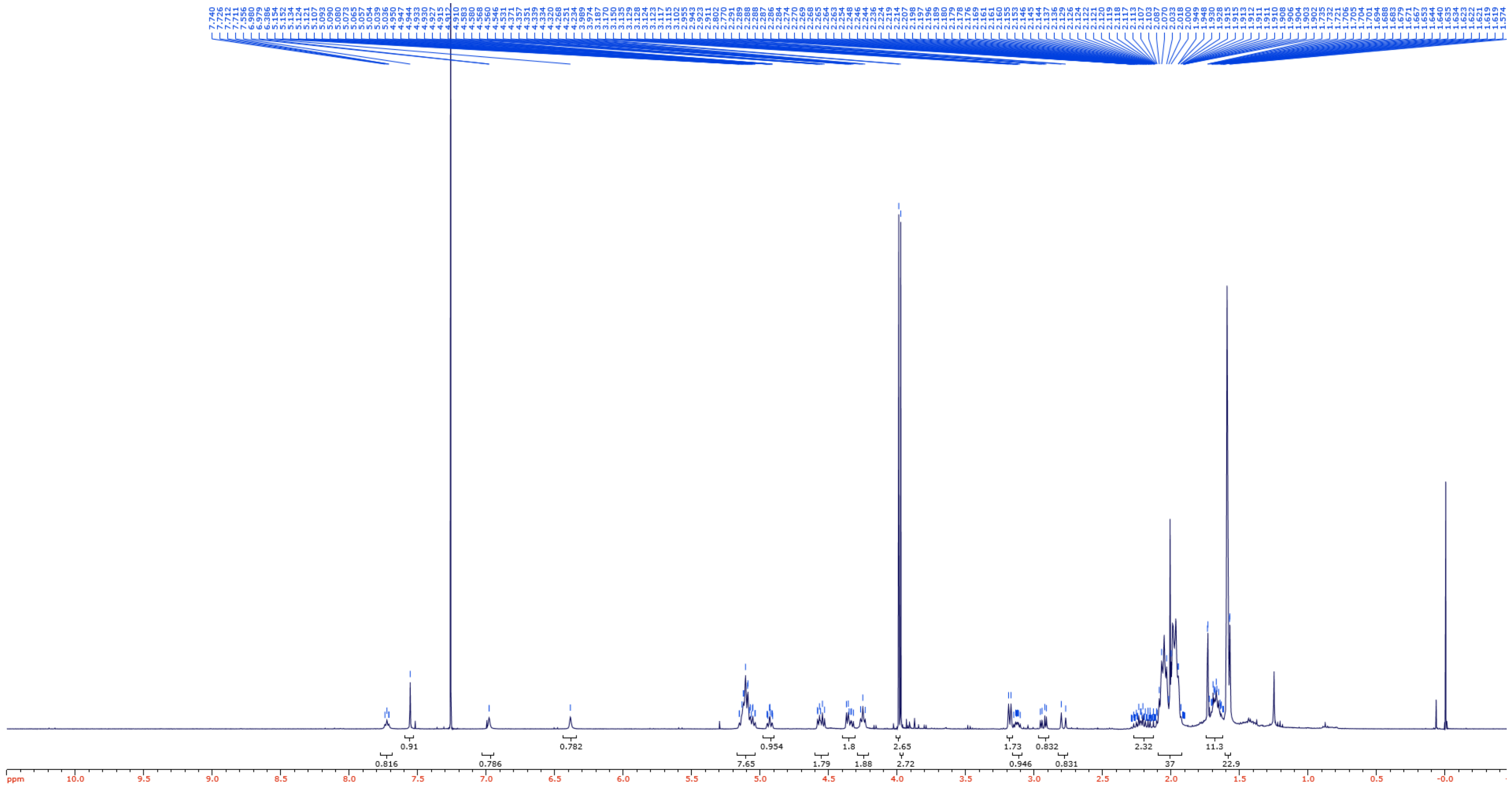

^{13}C NMR (400 MHz, CDCl$_3$) for CoQ10-ω-biotin (36)

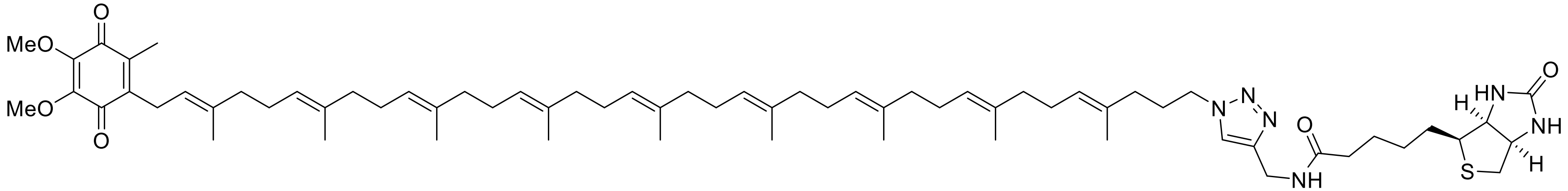

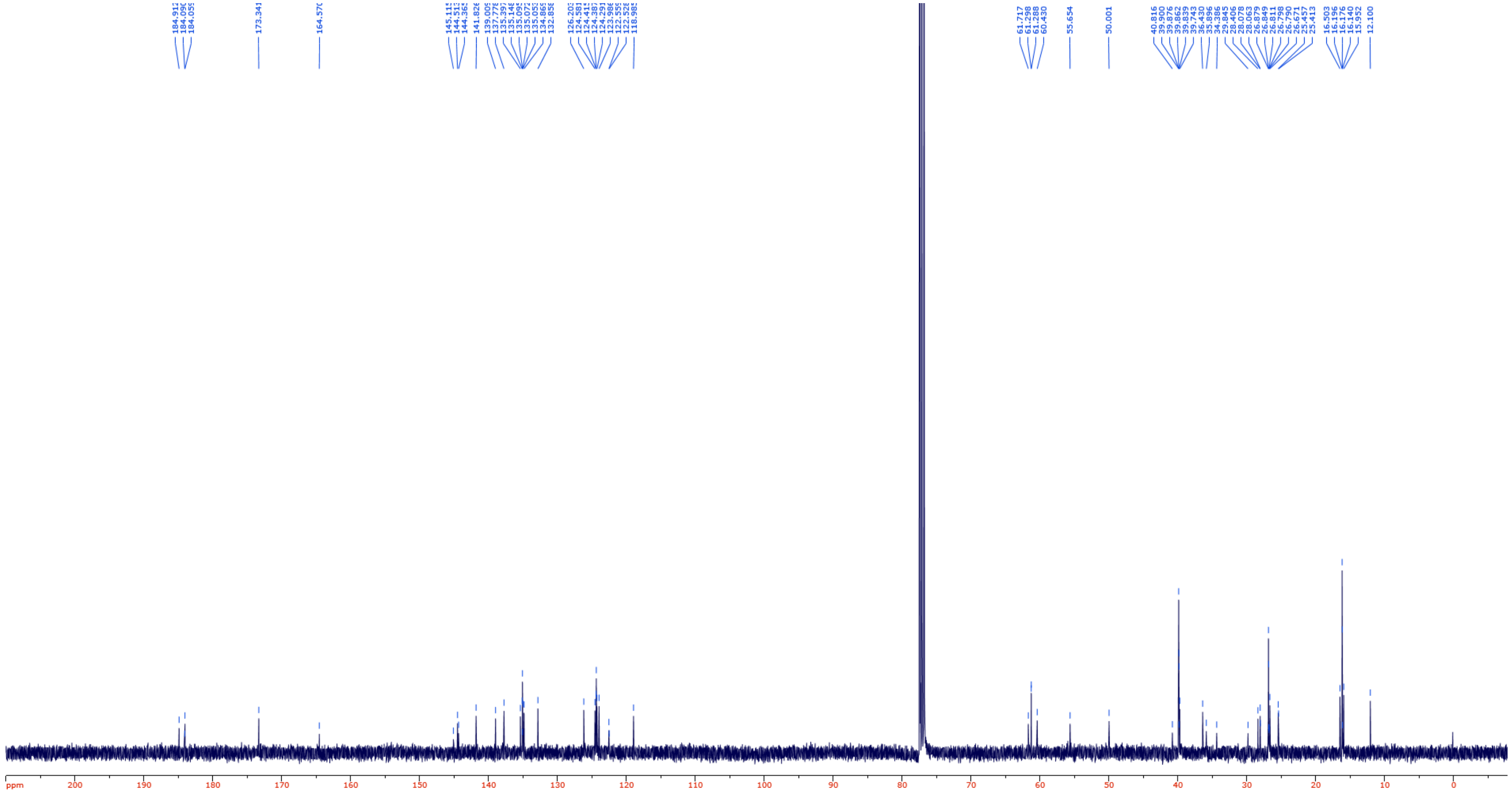

2D COSY (400 MHz, $CDCl_3$) for CoQ10-ω-biotin (36)

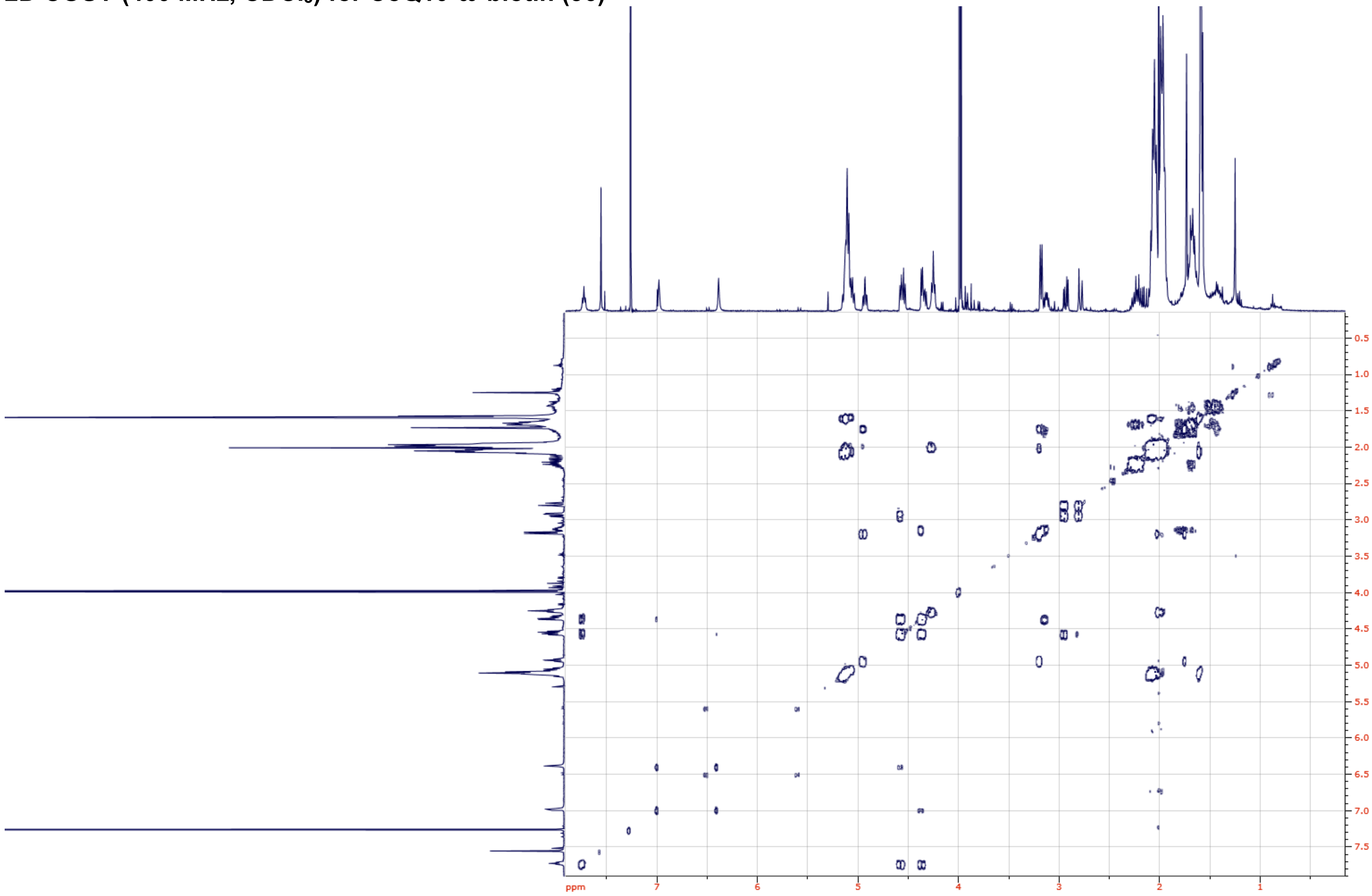

2D HSQC (400 MHz, $CDCl_3$) for CoQ10-ω-biotin (36)

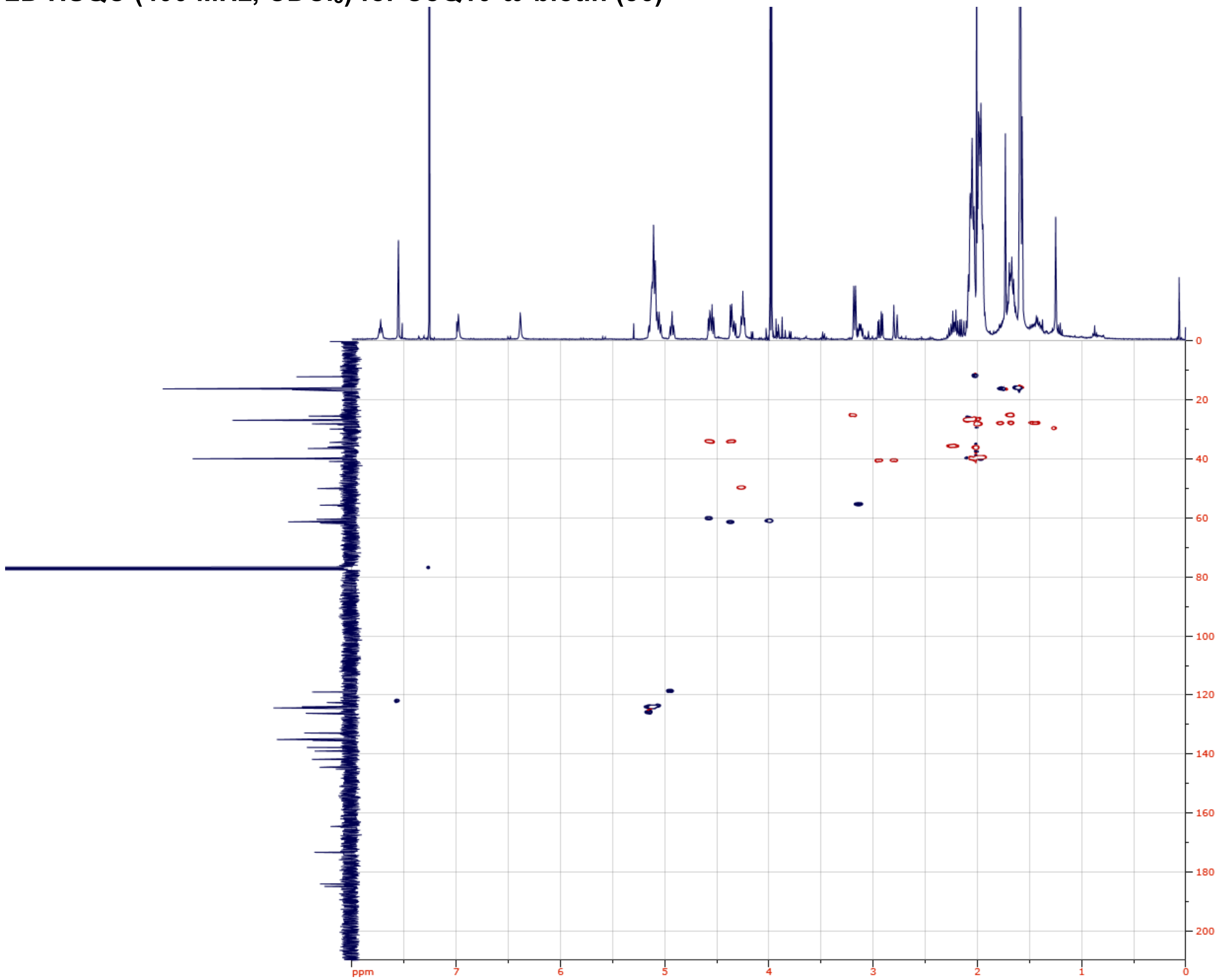

2D HMBC (400 MHz, $CDCl_3$) for CoQ10-ω-biotin (36)

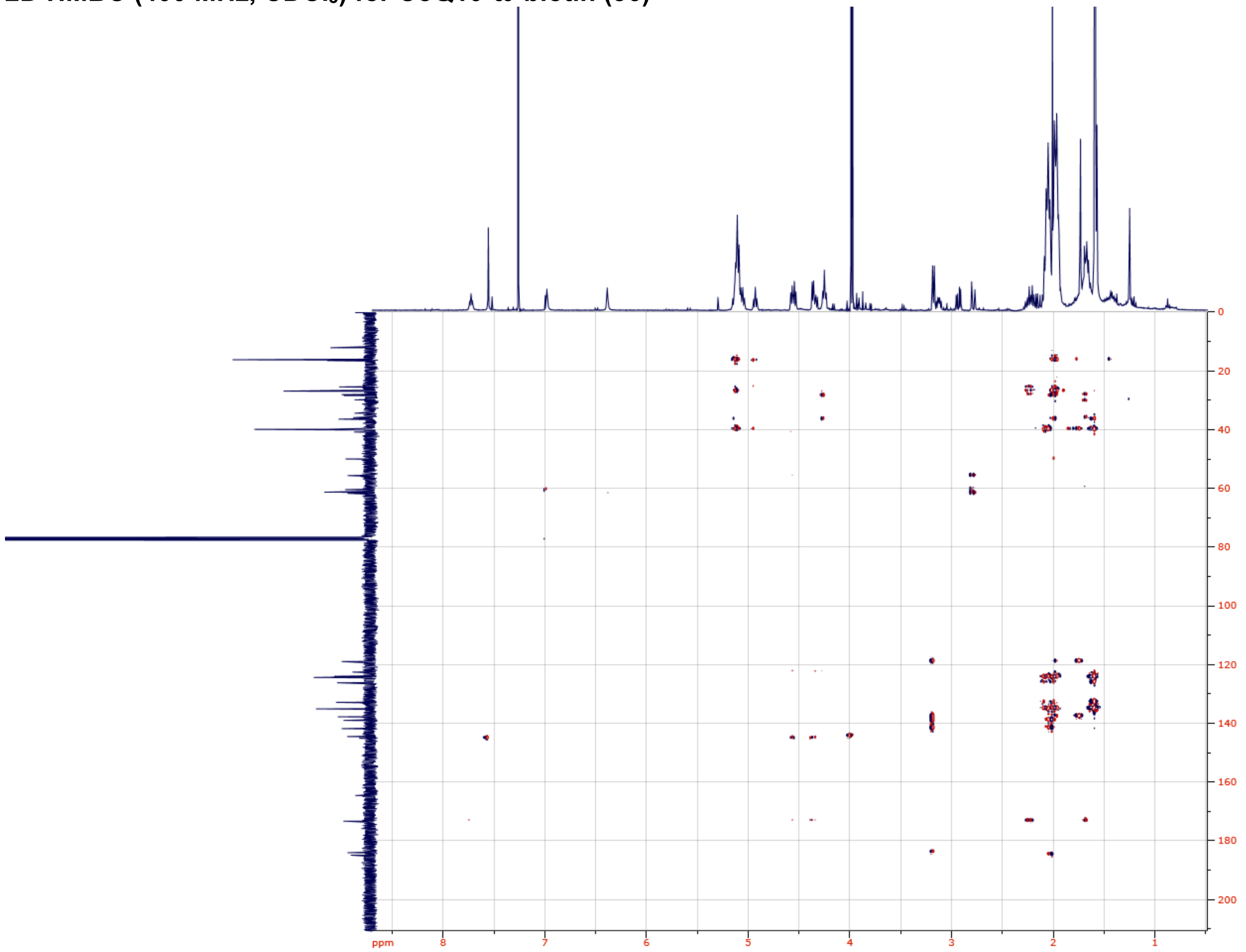

Analytical HPLC Traces

Hex-LysE (37)

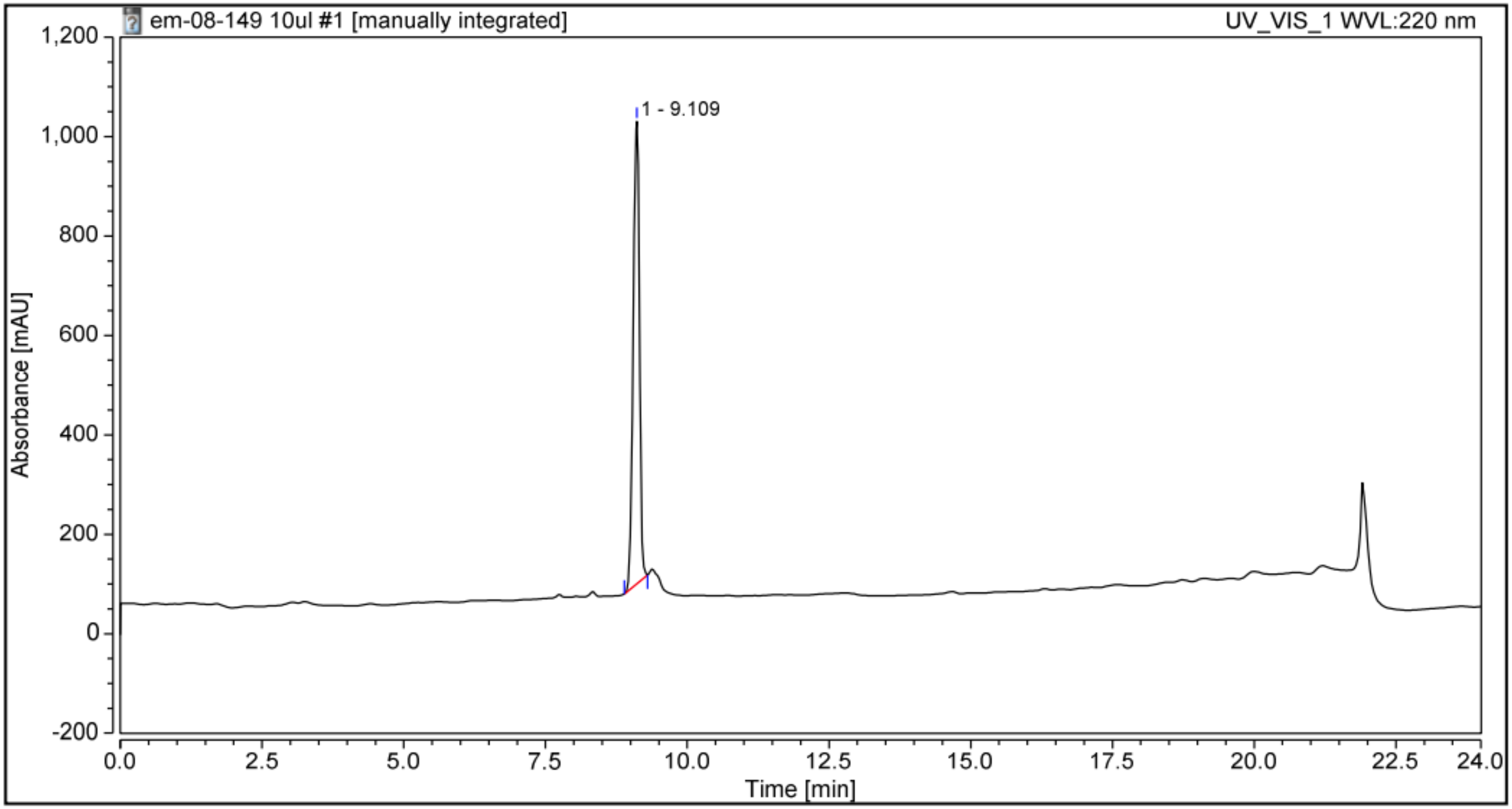

Hex-MBA2 (38)

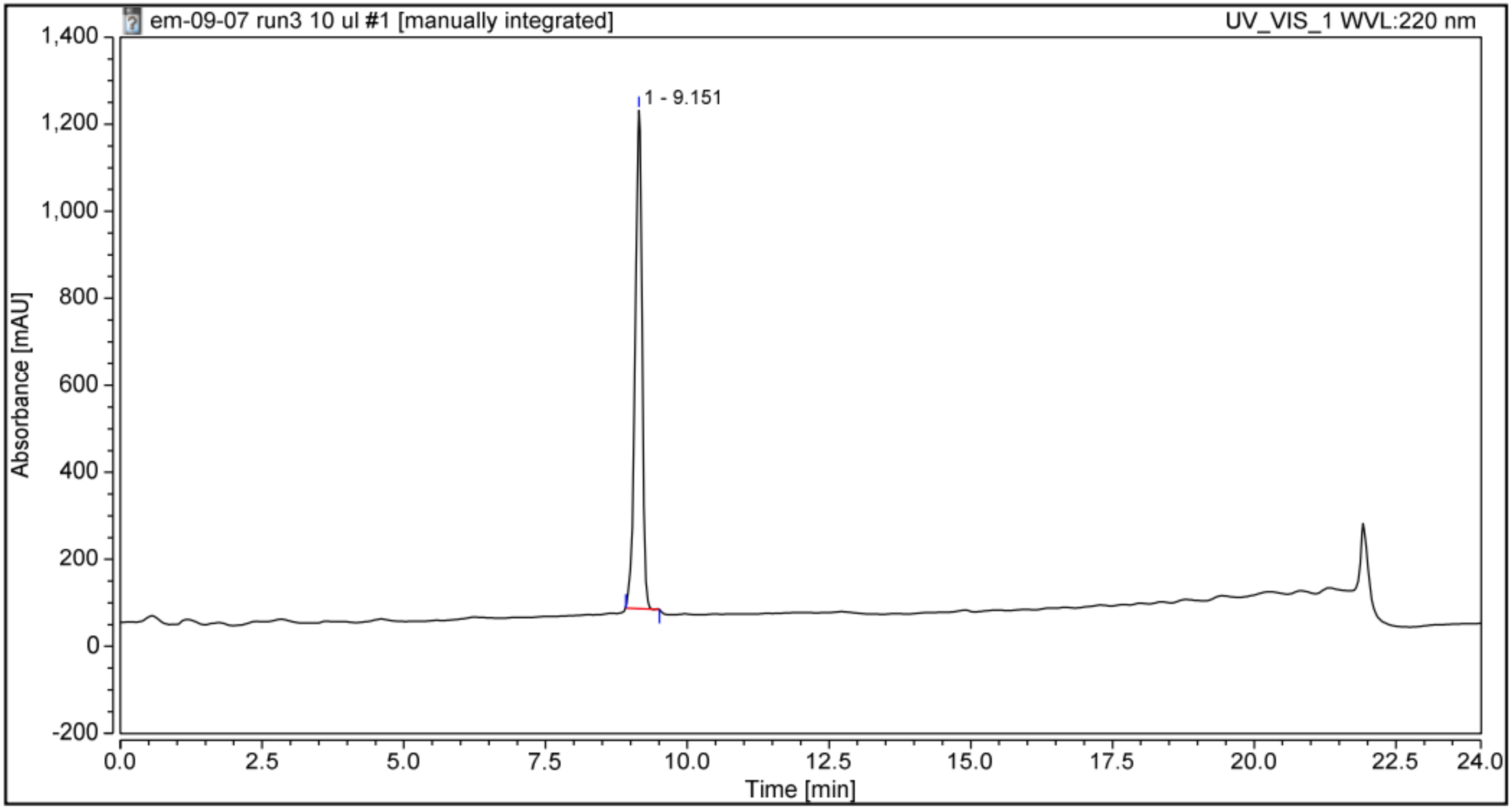
em-09-07 run3 10 ul #1 [manually integrated]
UV_VIS_1 WVL:220 nm
1 - 9.151
Absorbance [mAU]
1,400
1,200
1,000
800
600
400
200
0
-200
0.0
2.5
5.0
7.5
10.0
12.5
15.0
17.5
20.0
22.5
24.0
Time [min]

IR Spectra

Boc$_2$-CoQ10 (5)

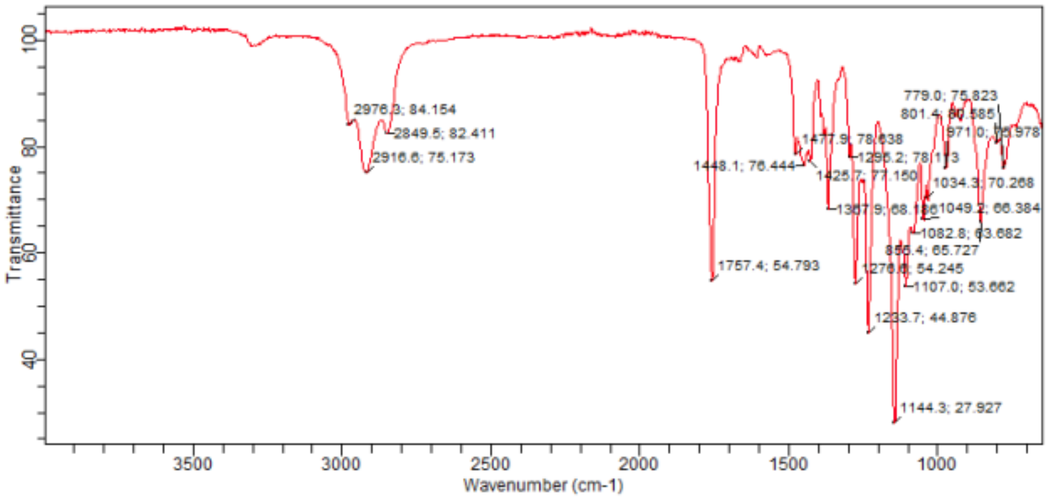

Boc$_2$-MK4 (6)

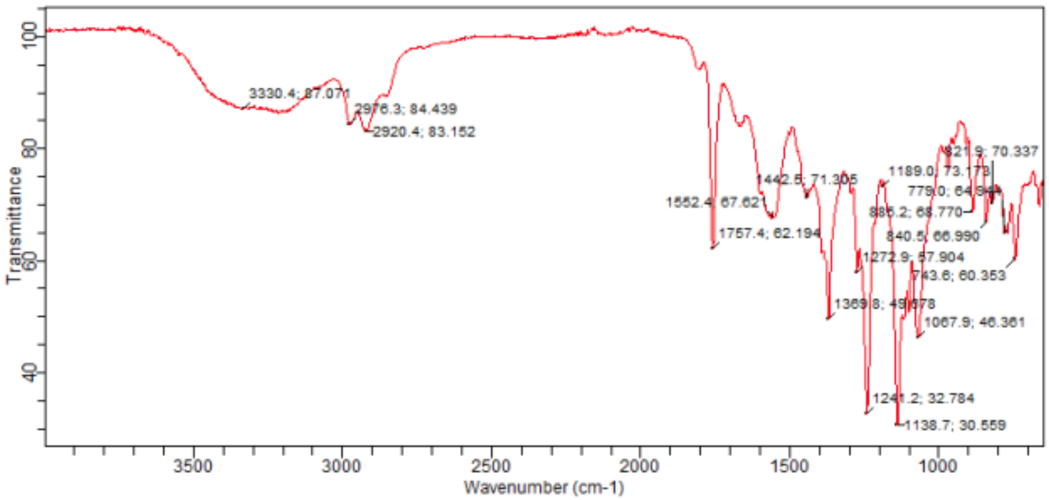

Boc$_2$-MK9 (7)

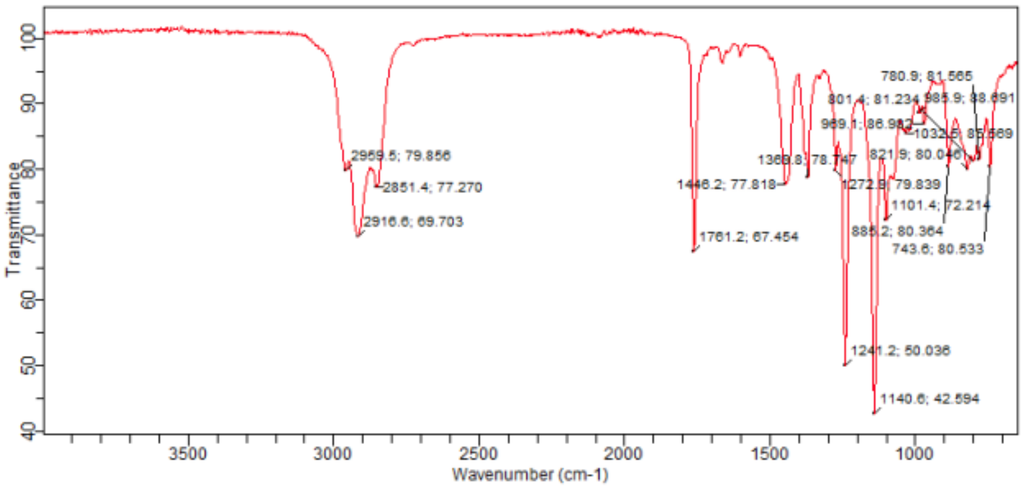

Me$_2$-CoQ10-ω-epoxide (8)

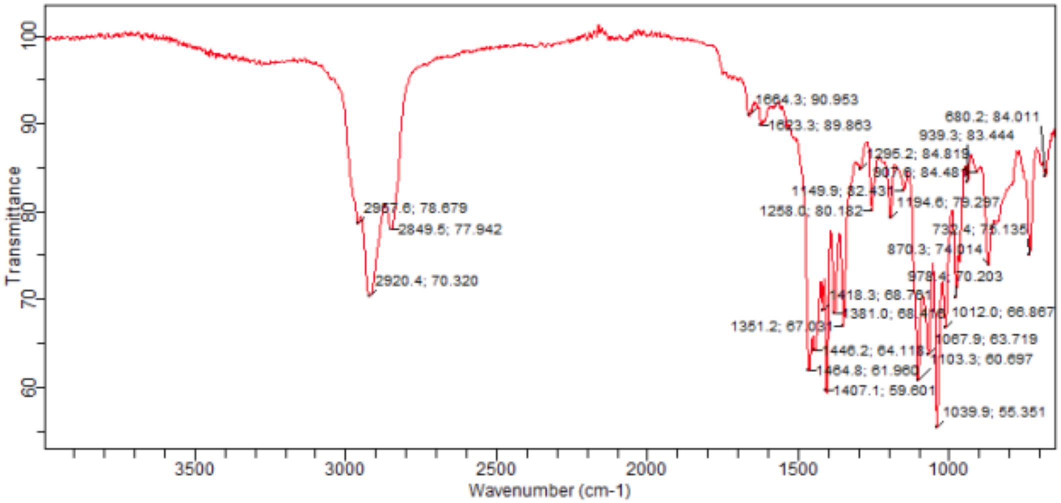

Boc$_2$-CoQ10-ω-epoxide (9)

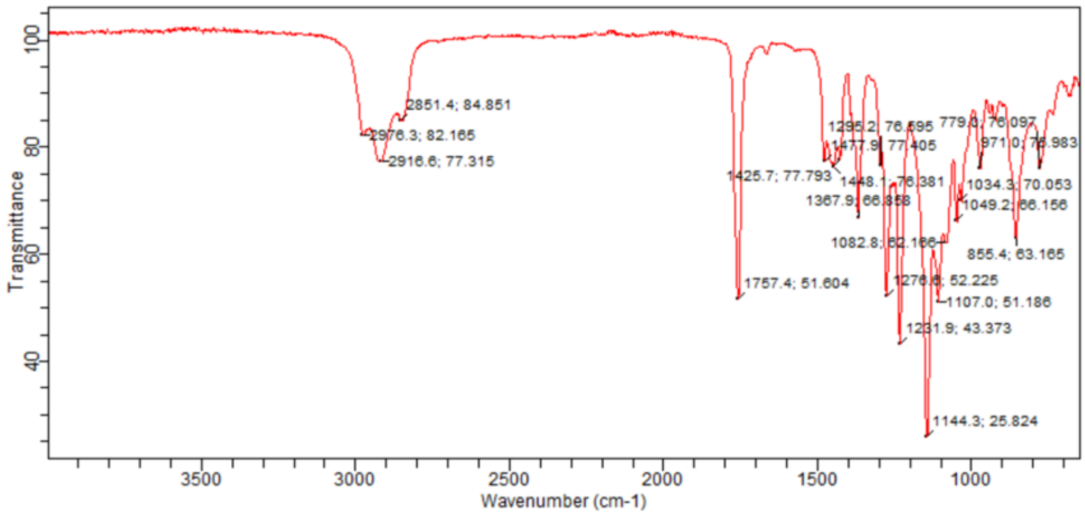

Boc$_2$-MK4-ω-epoxide (10)

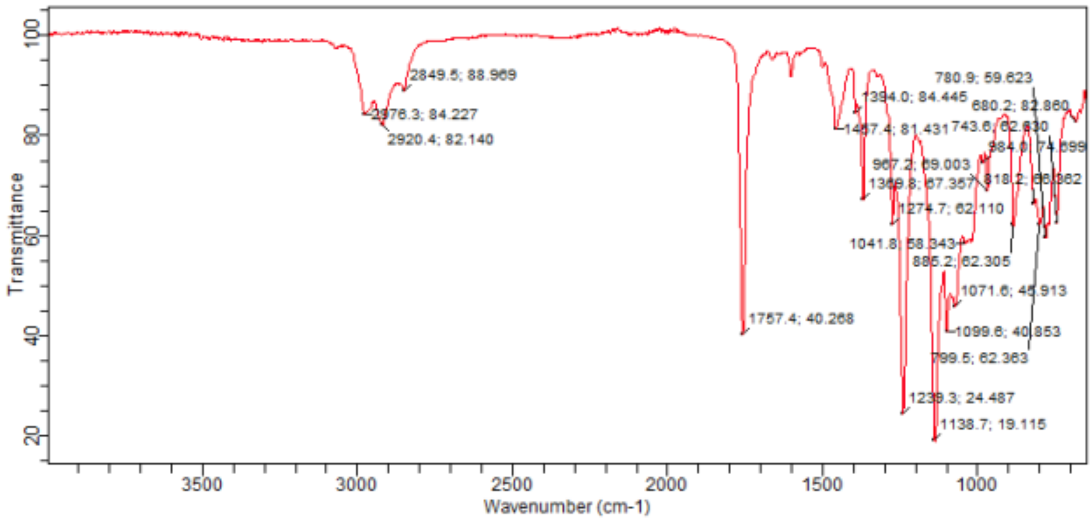

Boc$_2$-MK9-ω-epoxide (11)

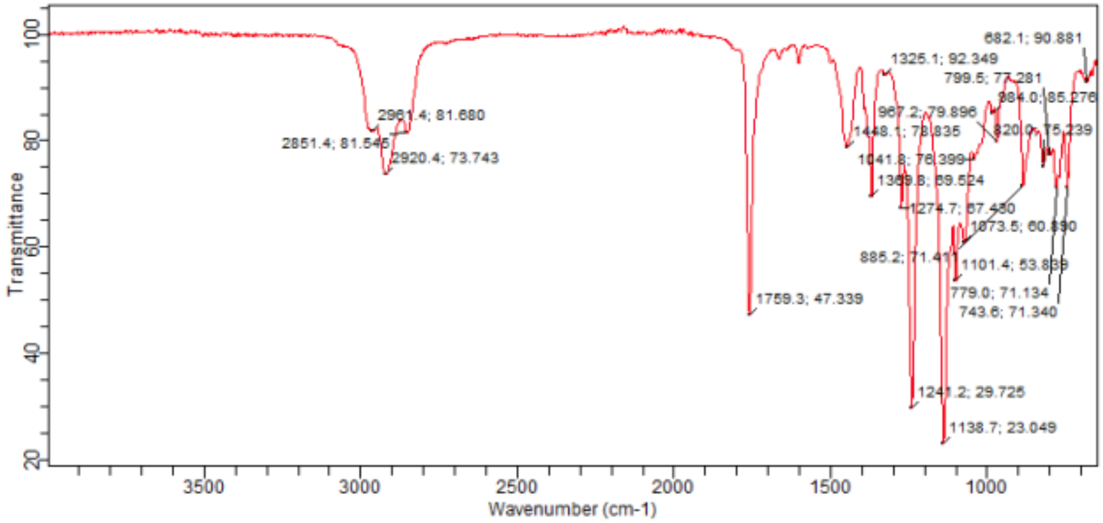

Me$_2$-CoQ10-ω-2AB (12)

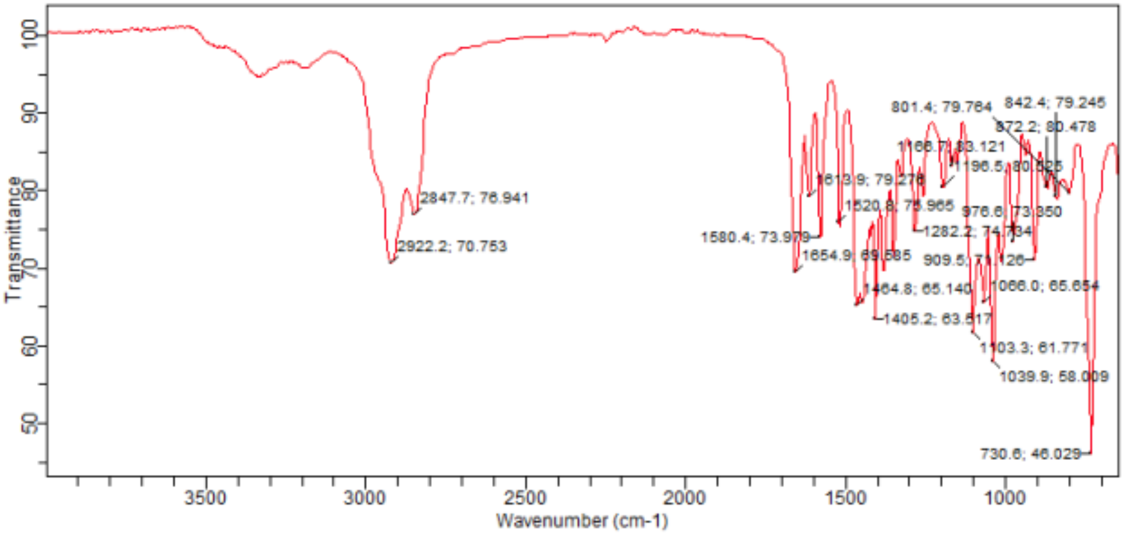

Boc$_2$-CoQ10-ω-2AB (13)

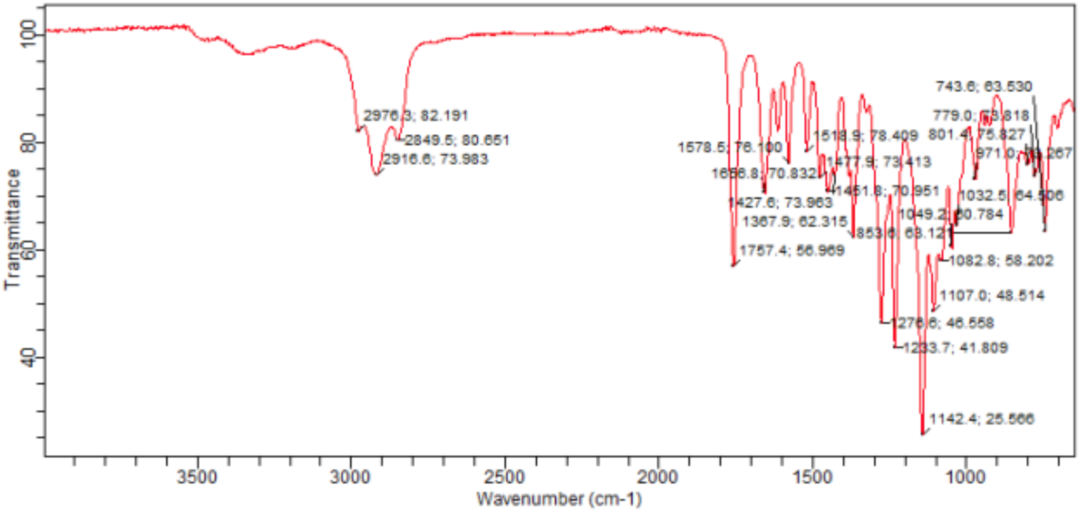

Boc$_2$-MK4-ω-2AB (14)

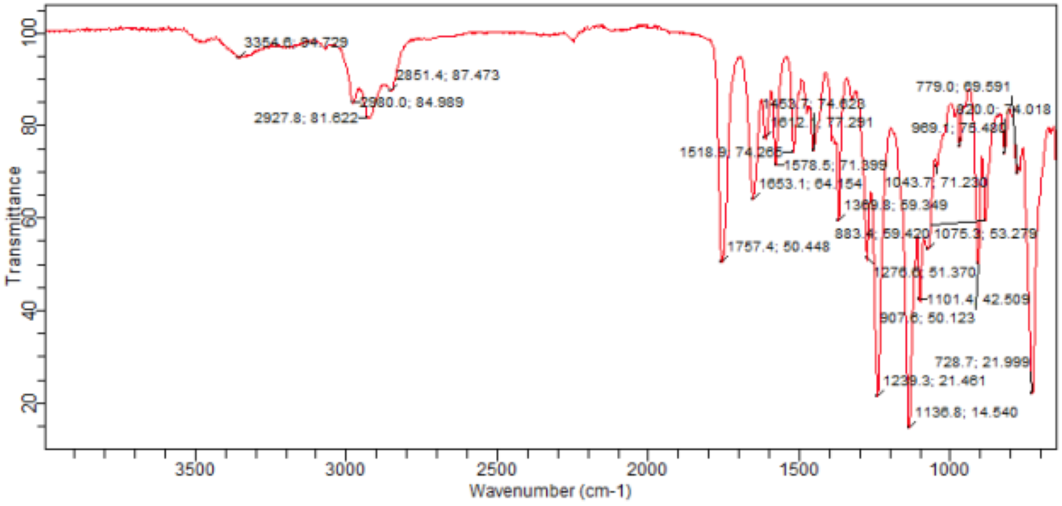

Boc$_2$-MK9-ω-2AB (15)

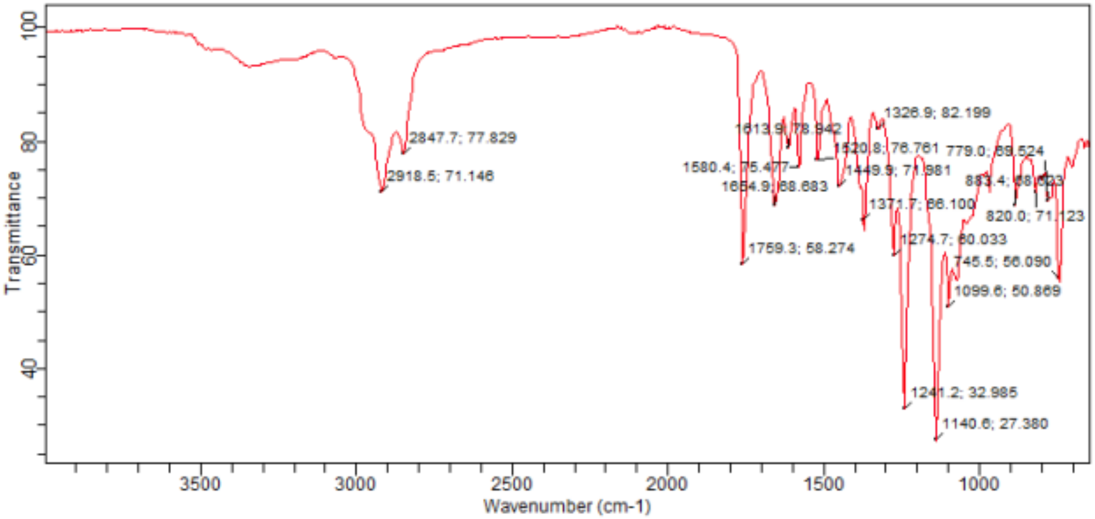

CoQ10-ω-2-AB (16)

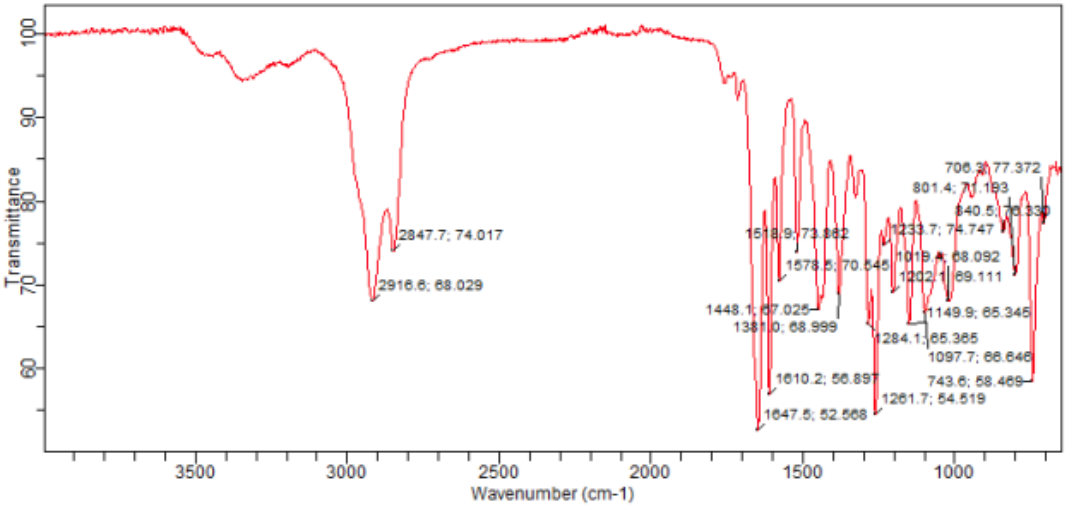

MK4-ω-2-AB (17)

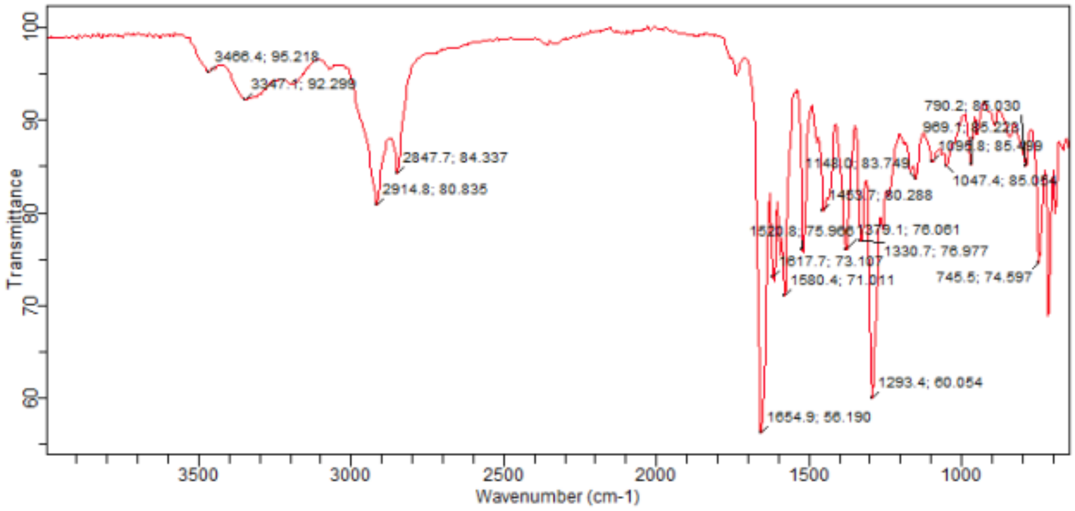

MK-9-ω-2-AB (18)

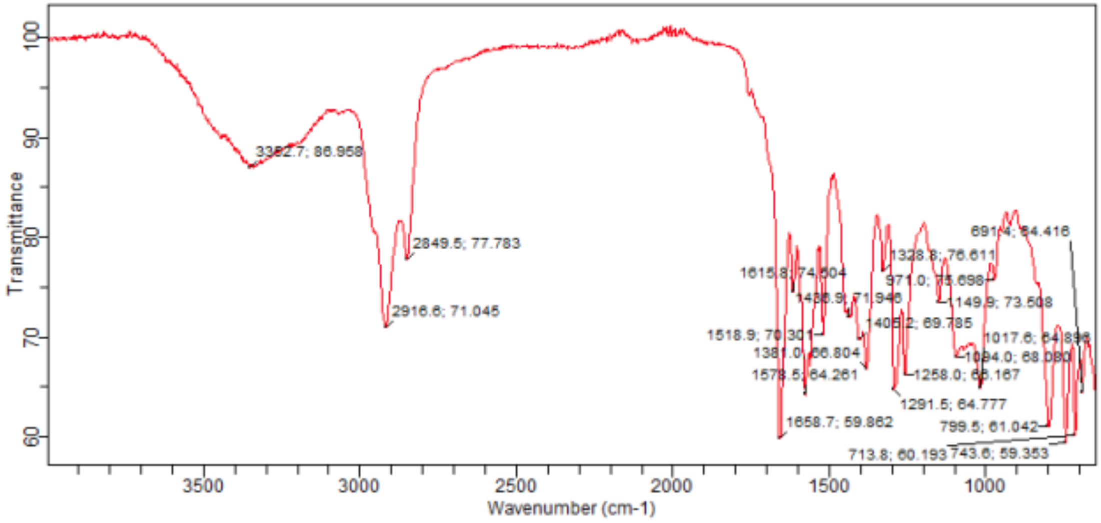

Boc$_2$-CoQ10-ω-alkyne (19a)

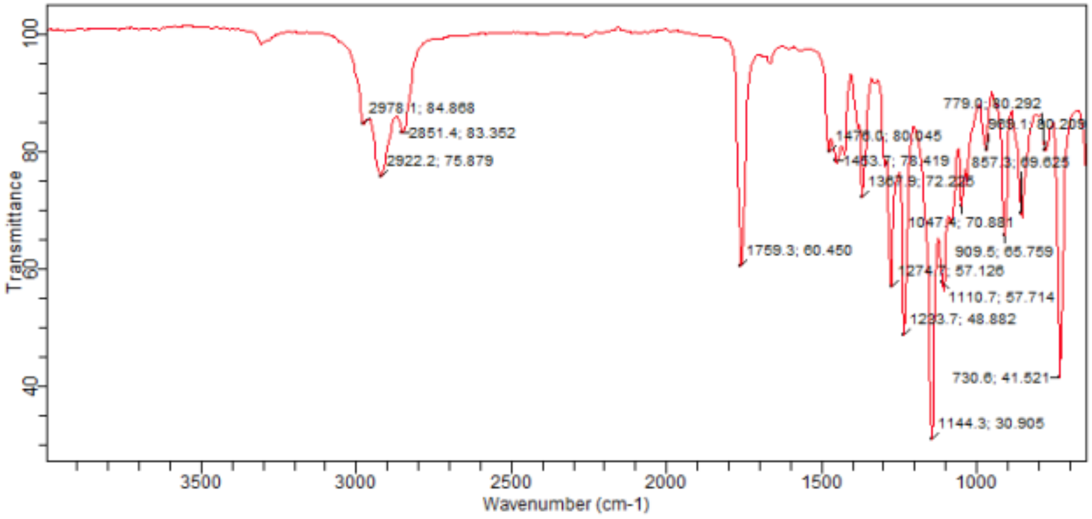

Boc$_2$-CoQ10-ω-CF$_3$ (20a)

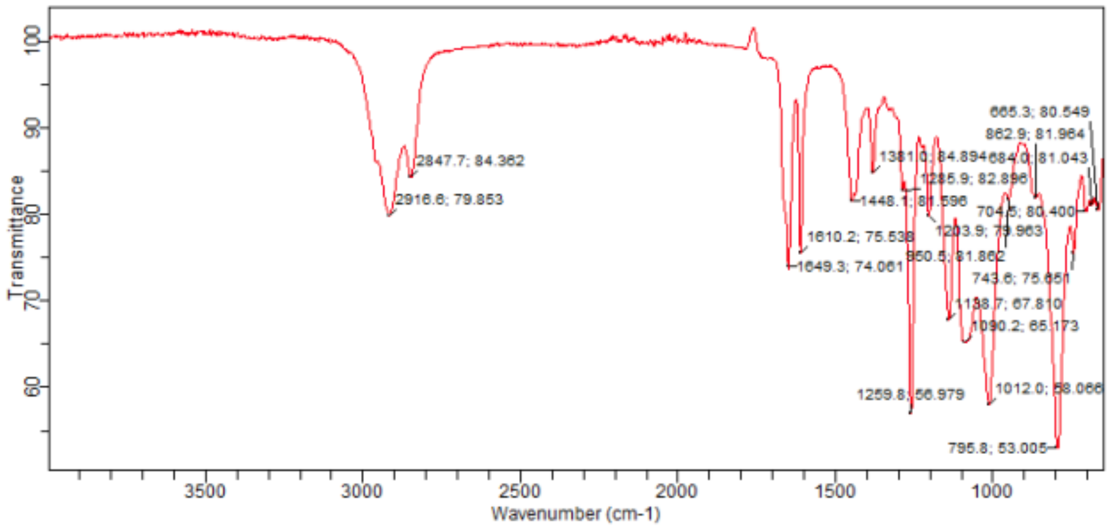

Boc$_2$-CoQ10-ω-pyrene (21a)

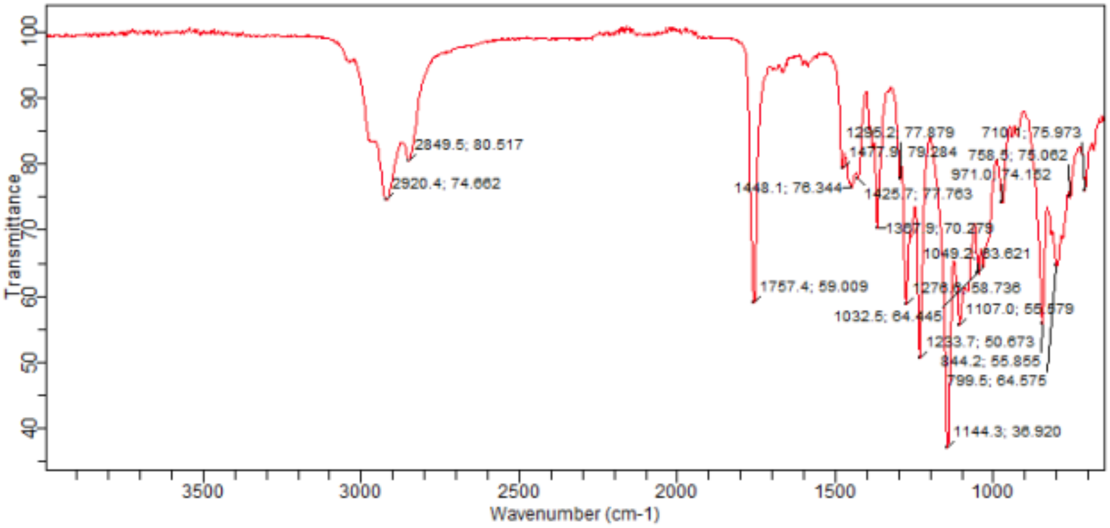

Boc$_2$-CoQ10-ω-PNB (22a)

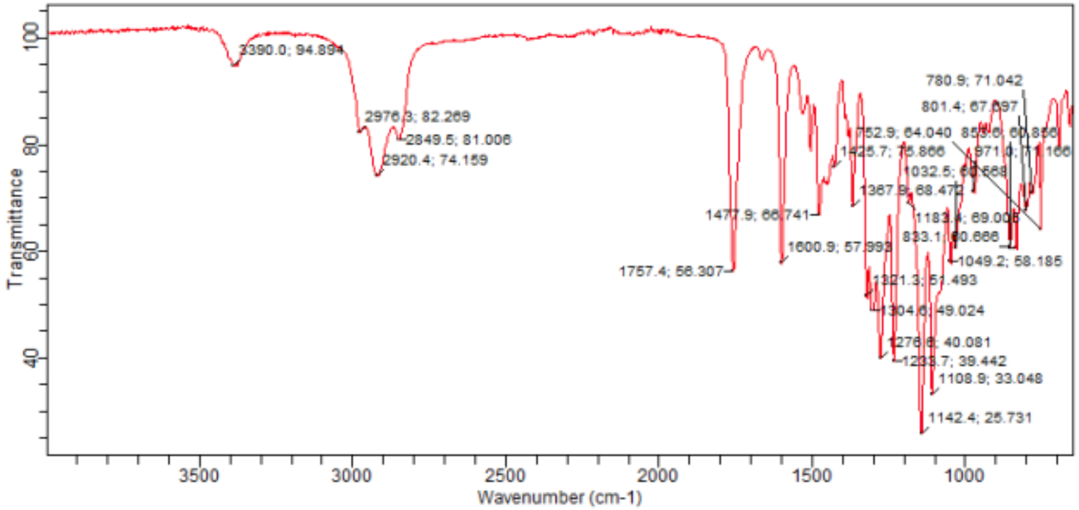

Boc_2-CoQ10-ω-diazirine (23a)

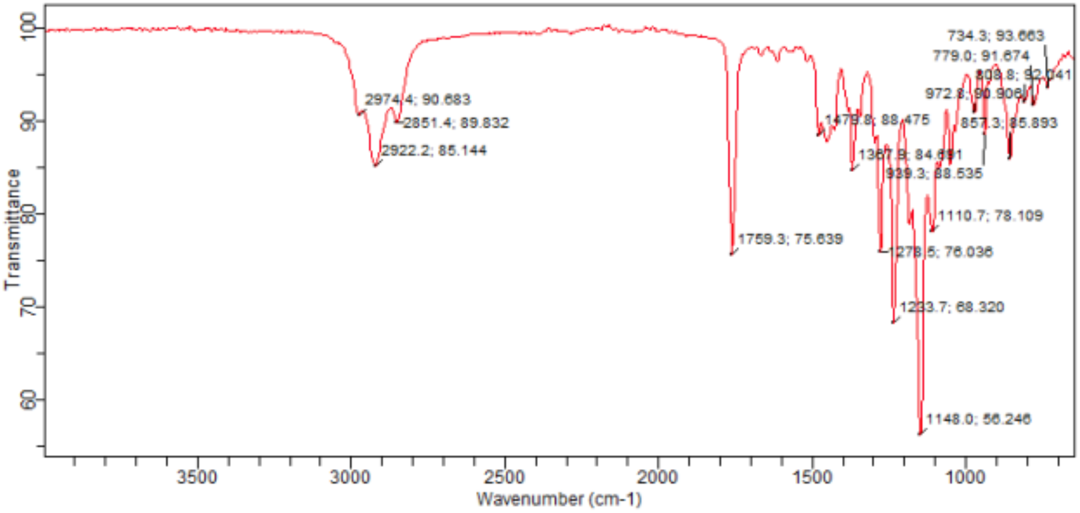

CoQ10-ω-alkyne (19)

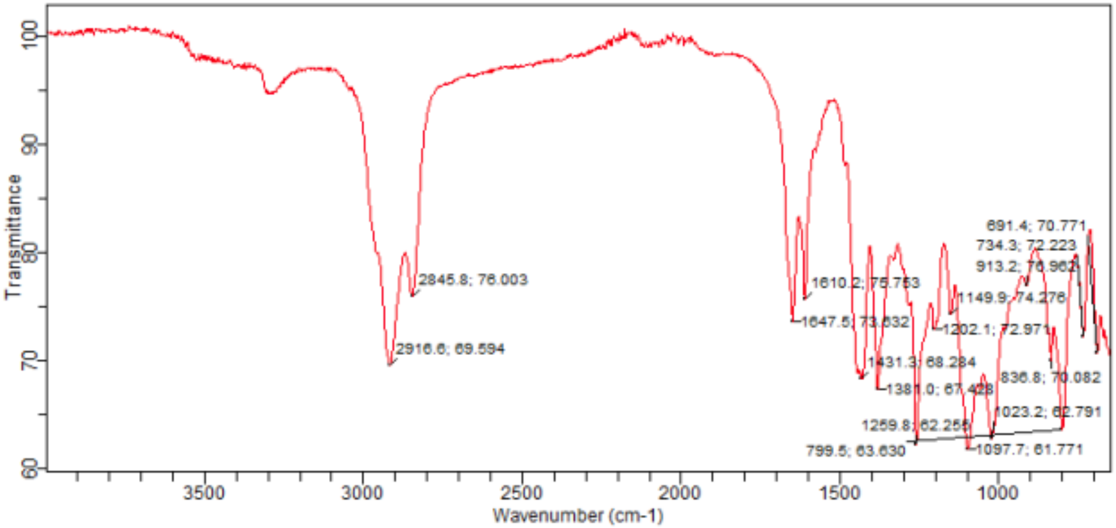

CoQ10-ω-CF_3 (20)

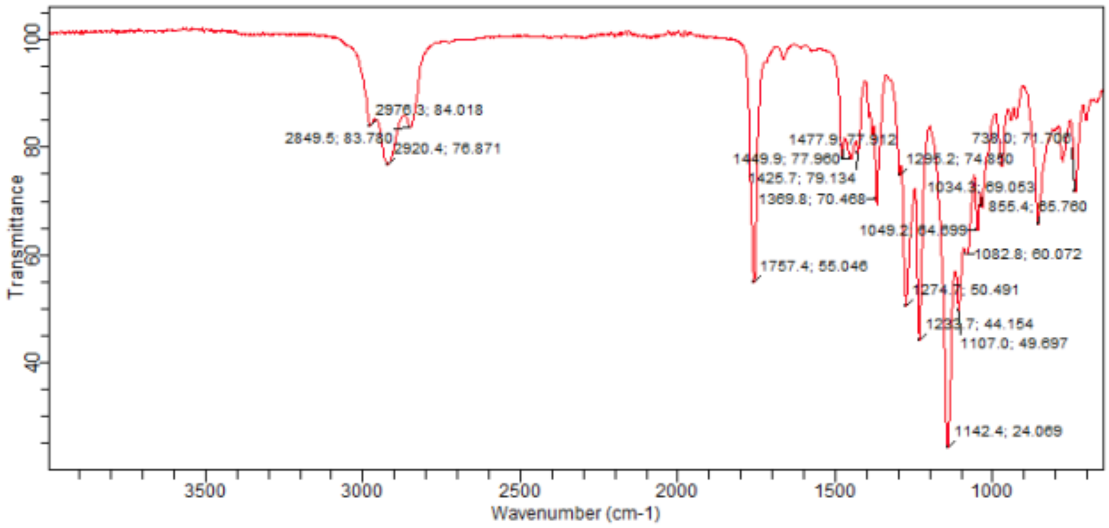

CoQ10-ω-pyrene (21)

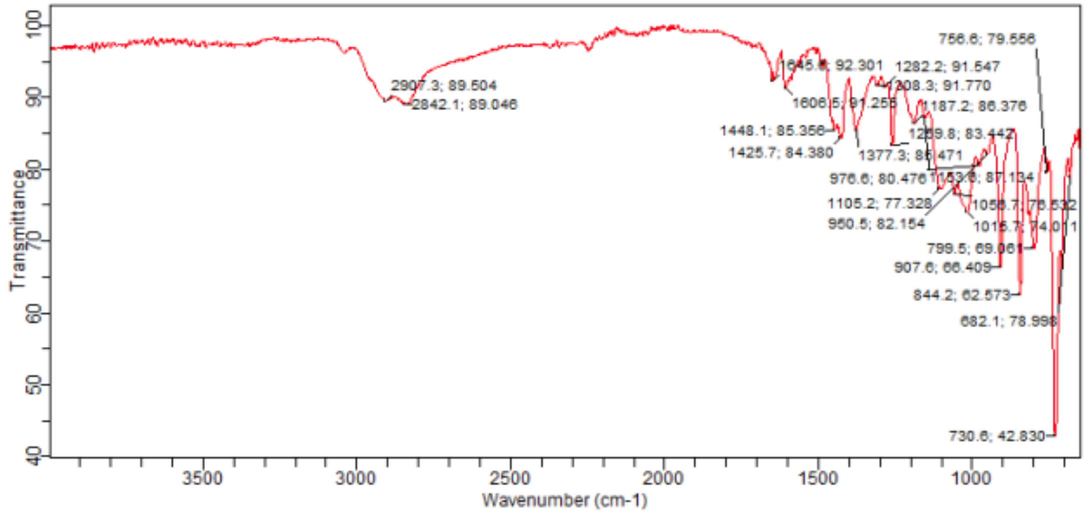

CoQ10-ω-PNB (22)

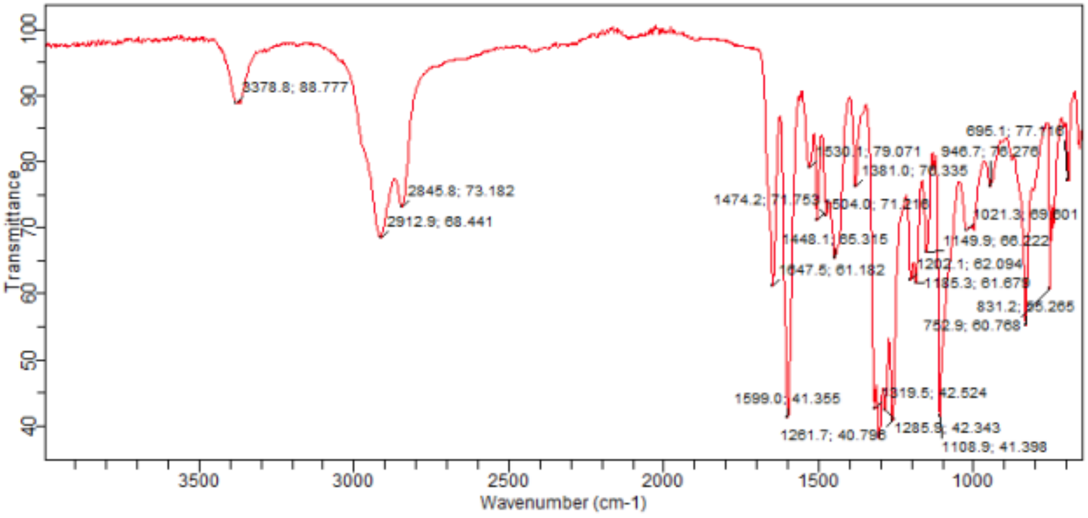

CoQ10-ω-diazirine (23)

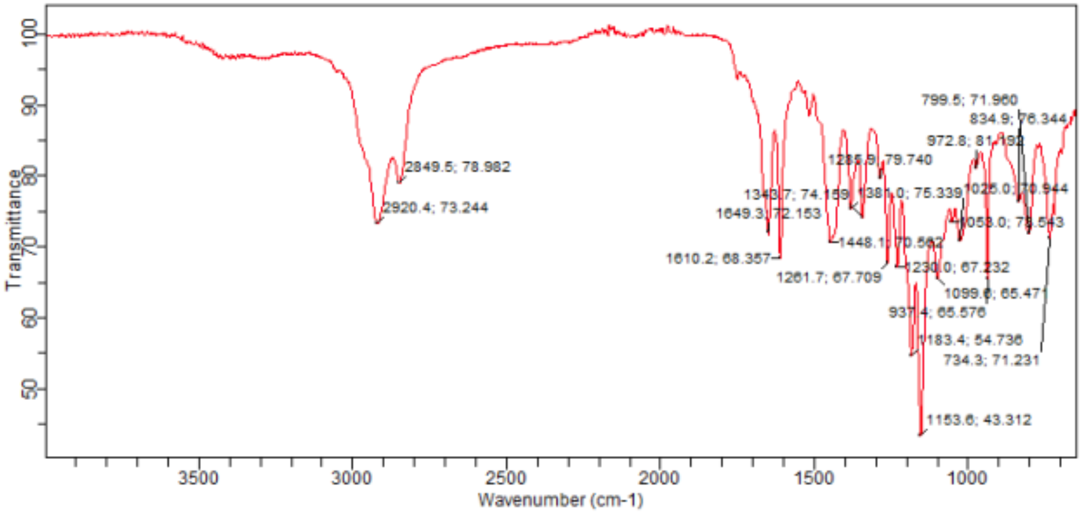

Boc₂-CoQ10-ω-OTs (24)

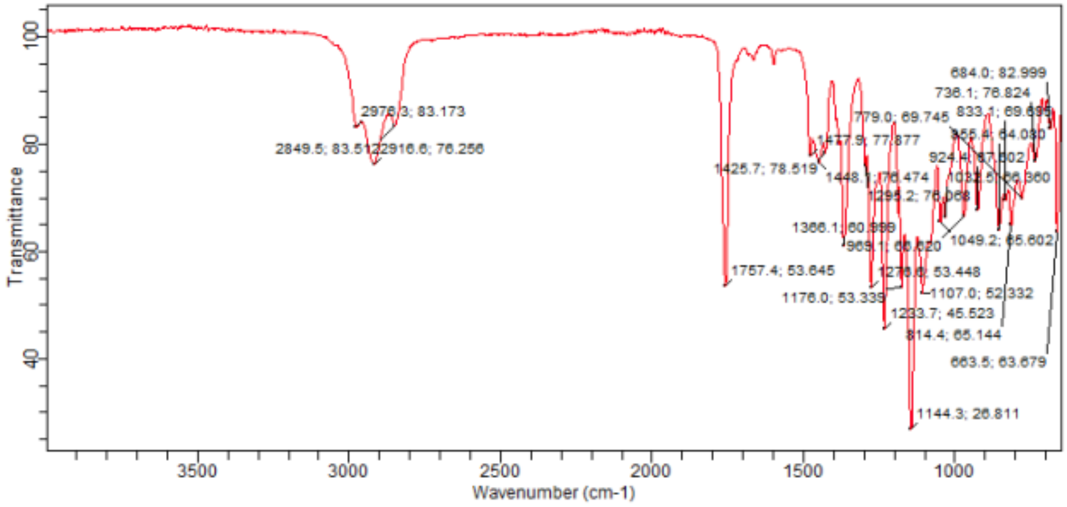

Boc₂-MK9-ω-OTs (25)

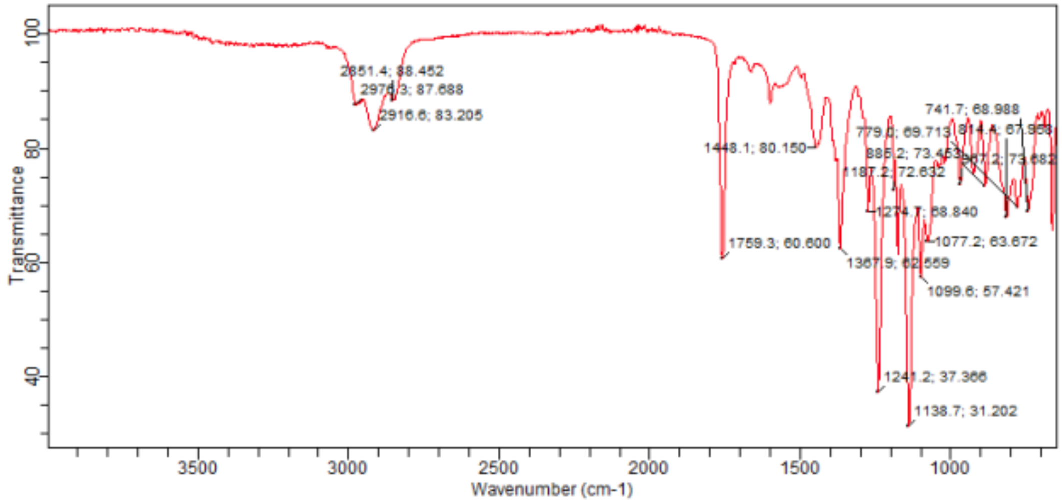

Boc$_2$-CoQ10-ω-azide (26)

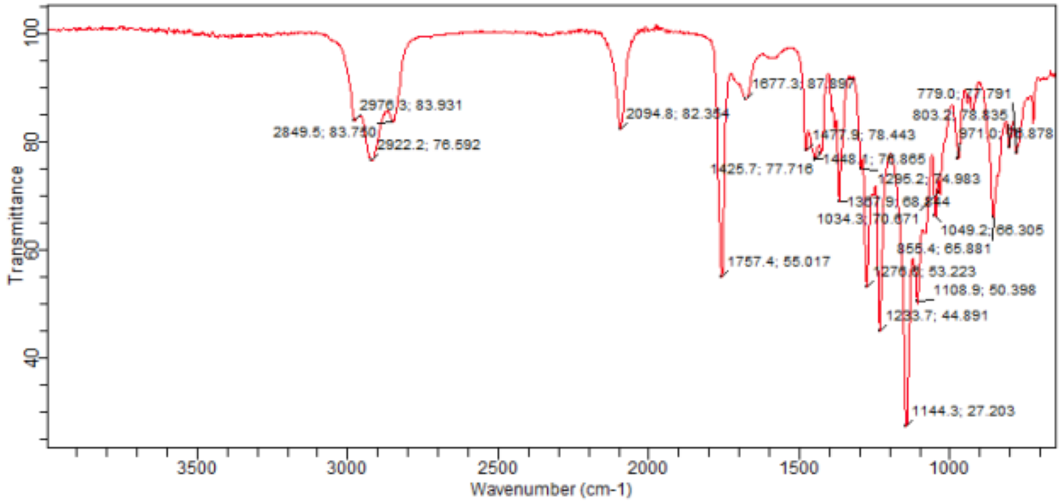

Boc$_2$-MK9-ω-azide (27)

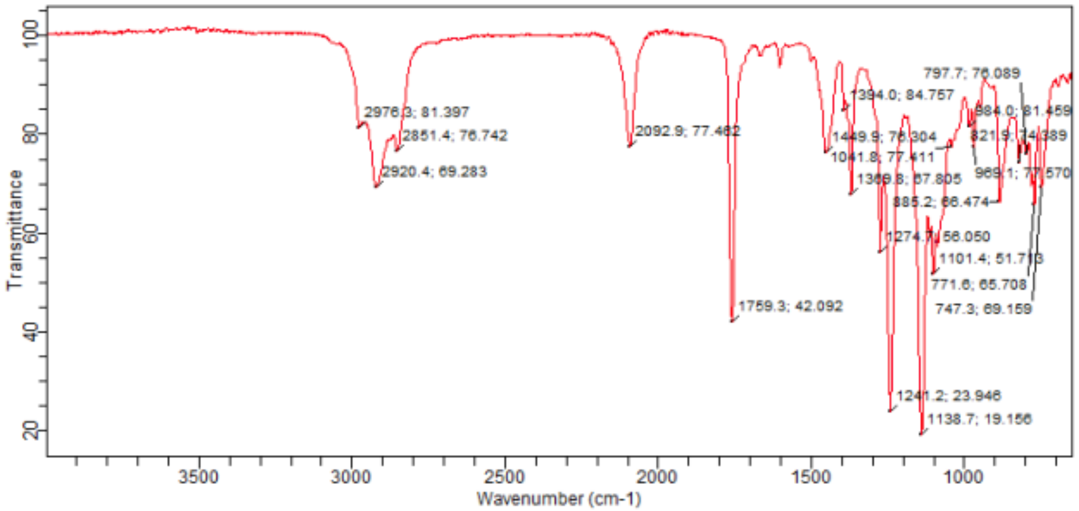

CoQ10-ω-azide (28)

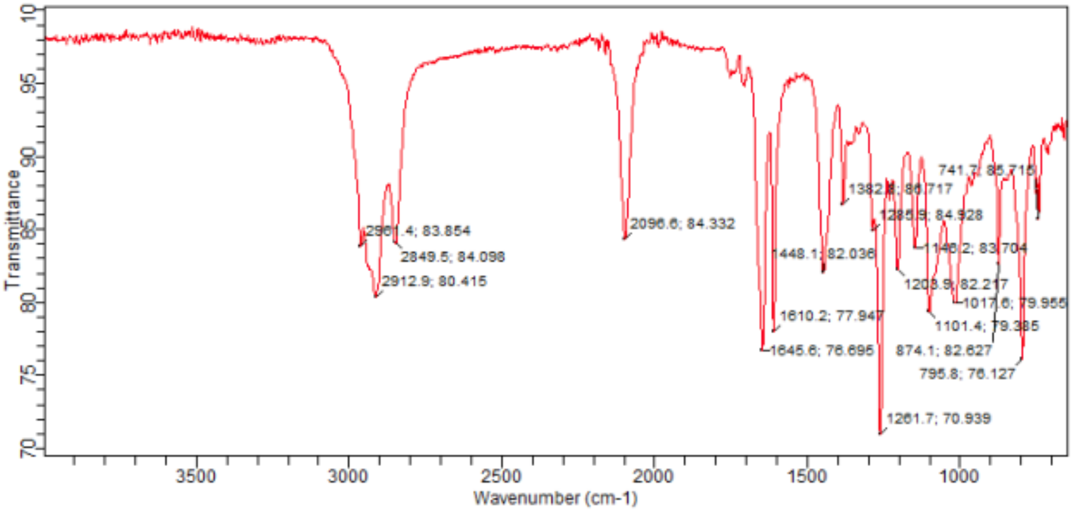

MK9-ω-azide (29)

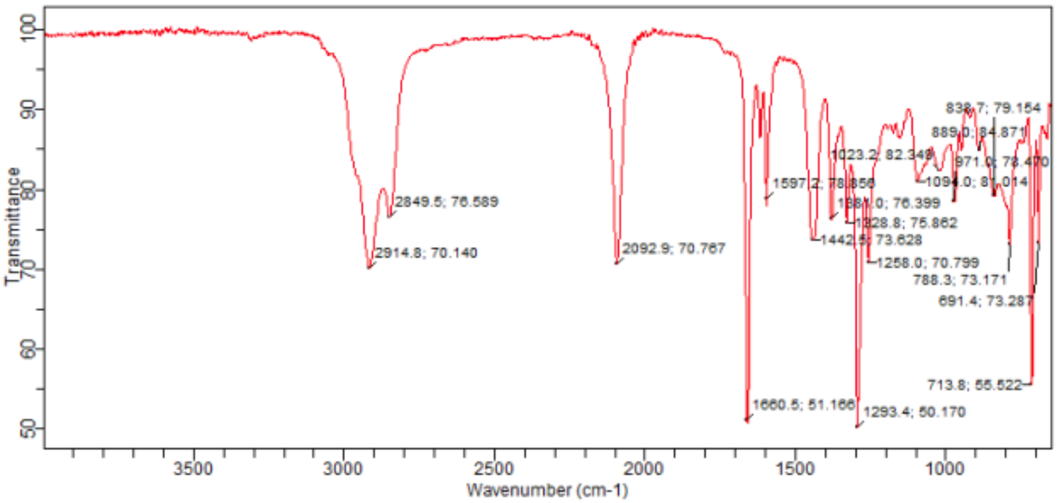

CoQ10-ω-NBD (30)

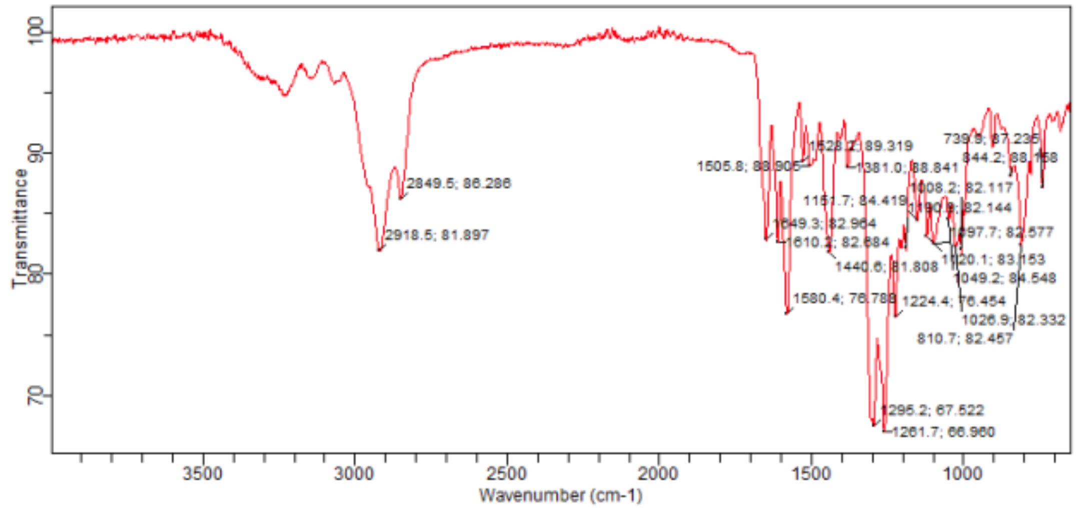

MK9-ω-NBD (31)

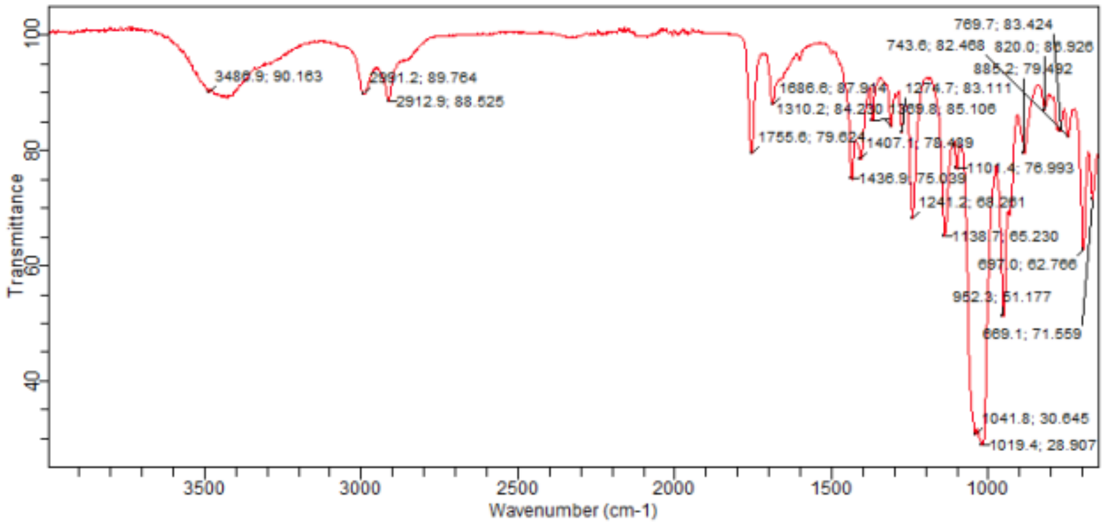

CoQ10-ω-DNB (32)

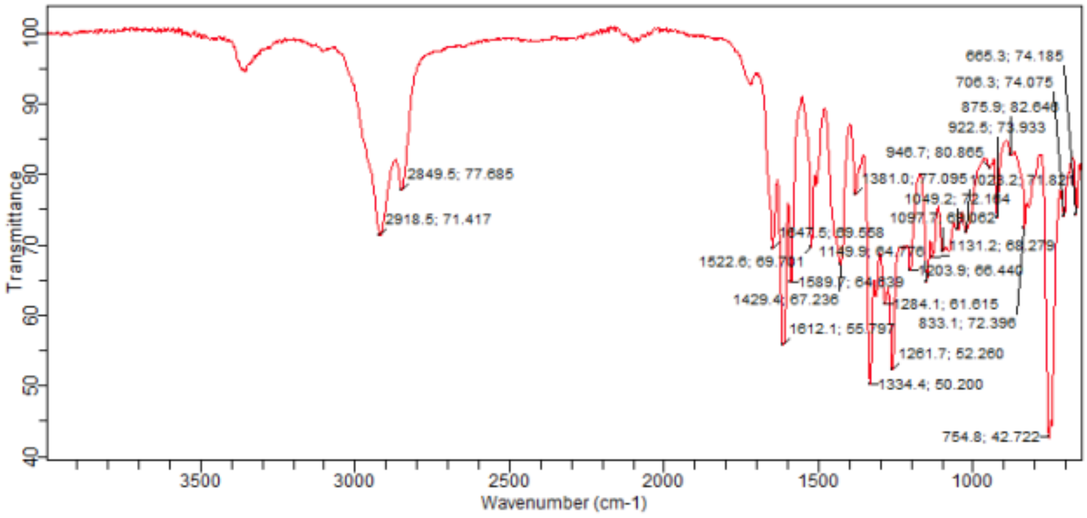

MK9-ω-DNB (33)

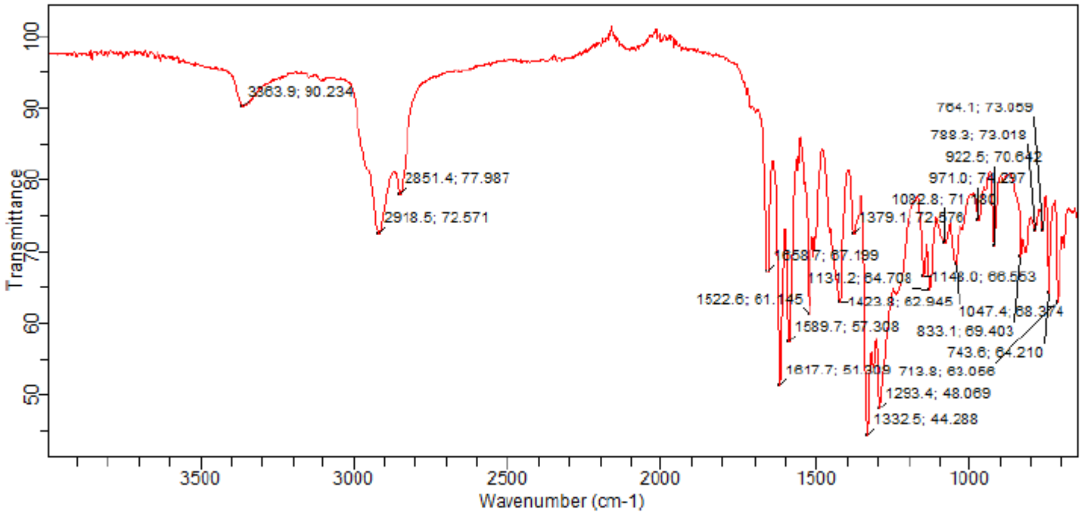

CoQ10-ω-BODIPY (34)

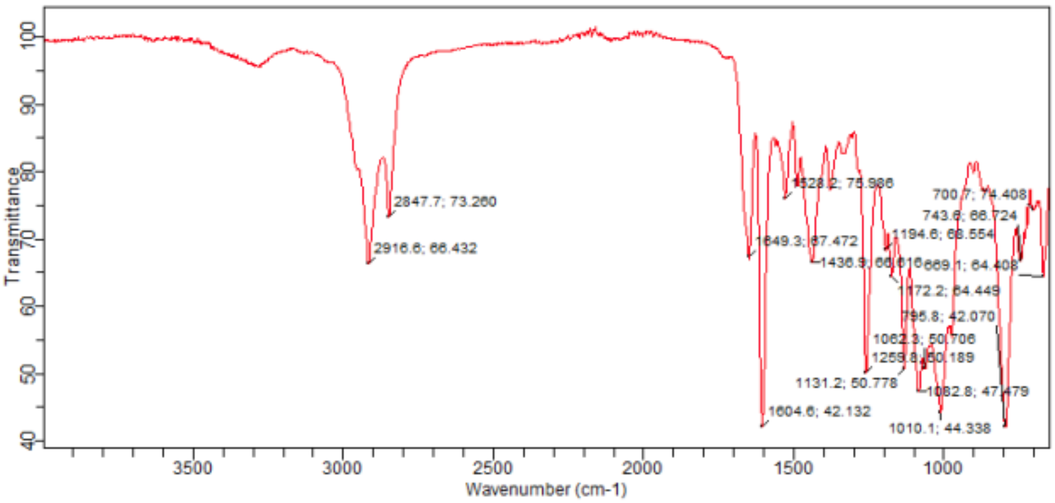

MK9-ω-BODIPY (35)

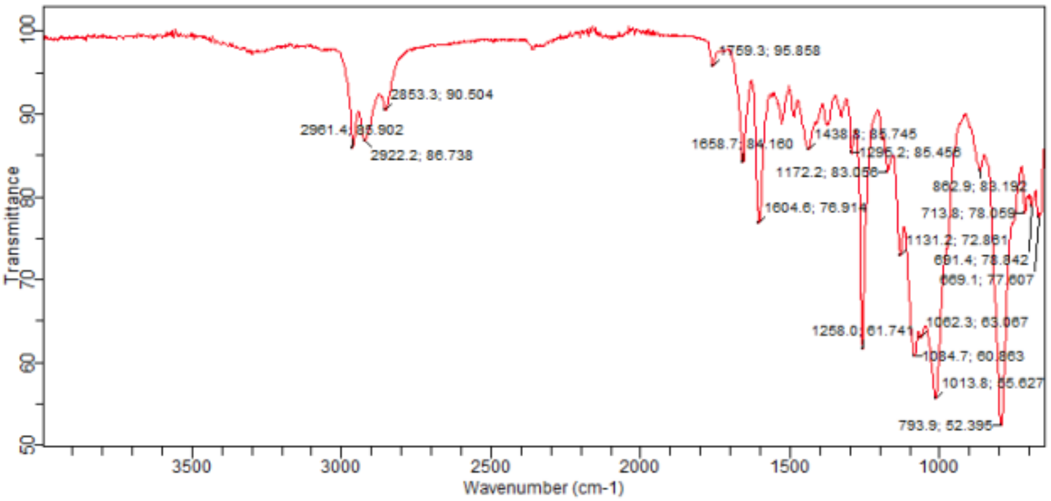

CoQ10-ω-biotin (36)

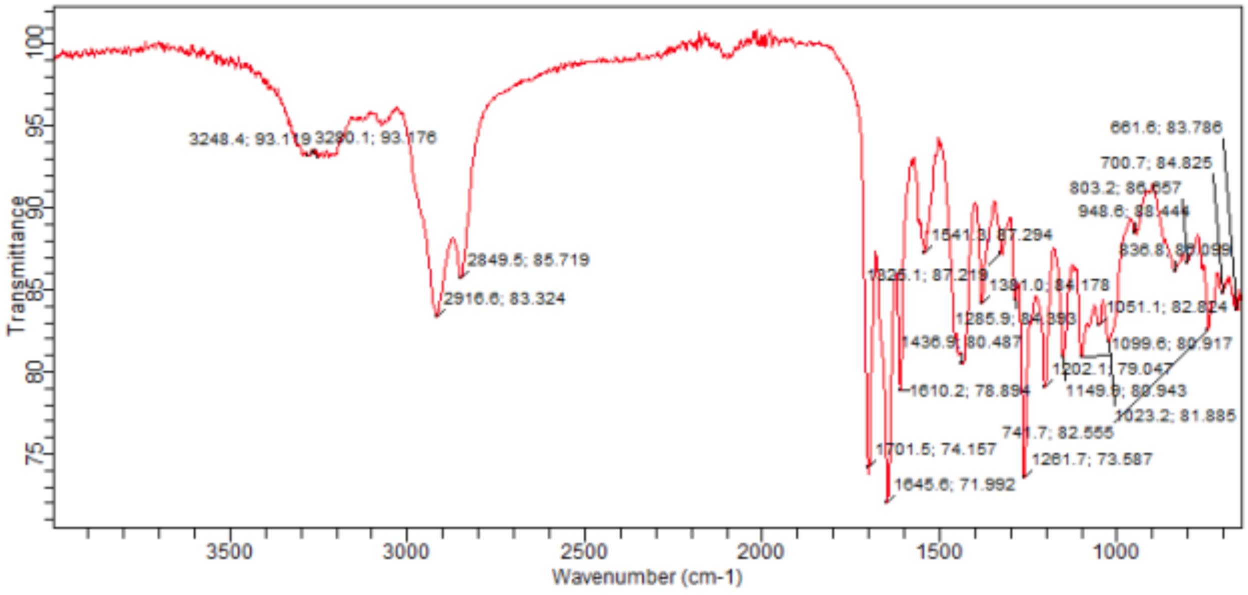

Supplement: SC-016-D5SC03363B-s001 [file SC-016-D5SC03363B-s001.pdf]
